# Diversity of macaque microbiota compared to the human counterparts

**DOI:** 10.1038/s41598-018-33950-6

**Published:** 2018-10-22

**Authors:** Zigui Chen, Yun Kit Yeoh, Mamie Hui, Po Yee Wong, Martin C. W. Chan, Margaret Ip, Jun Yu, Robert D. Burk, Francis K. L. Chan, Paul K. S. Chan

**Affiliations:** 1 Department of Microbiology, Faculty of Medicine, The Chinese University of Hong Kong, Hong Kong, China; 2 Centre for Gut Microbiota Research, The Chinese University of Hong Kong, Hong Kong, China; 3 Department of Medicine and Therapeutics, Faculty of Medicine, The Chinese University of Hong Kong, Hong Kong, China; 4 State Key Laboratory of Digestive Disease, Institute of Digestive Disease, Li Ka Shing Institute of Health Sciences, and CUHK Shenzhen Research Institute, The Chinese University of Hong Kong, Hong Kong, China; 5 Departments of Pediatrics, Microbiology and Immunology, Epidemiology and Population Health, and Obstetrics, Gynecology and Woman’s Health, Albert Einstein College of Medicine, Bronx, NY USA

## Abstract

Studies on the microbial communities in non-human primate hosts provide unique insights in both evolution and function of microbes related to human health and diseases. Using 16S rRNA gene amplicon profiling, we examined the oral, anal and vaginal microbiota in a group of non-captive rhesus macaques (N = 116) and compared the compositions with the healthy communities from Human Microbiome Project. The macaque microbiota was dominated by Bacteroidetes, Firmicutes and Proteobacteria; however, there were marked differences in phylotypes enriched across body sites indicative of strong niche specialization. Compared to human gut microbiota where *Bacteroides* predominately enriched, the surveyed macaque anal community exhibited increased abundance of *Prevotella*. In contrast to the conserved human vaginal microbiota extremely dominated by *Lactobacillus*, the macaque vaginal microbial composition was highly diverse while lactobacilli were rare. A constant decrease of the vaginal microbiota diversity was observed among macaque samples from juvenile, adult without tubectomy, and adult with tubectomy, with the most notable distinction being the enrichment of *Halomonas* in juvenile and *Saccharofermentans* in contracepted adults. Both macaque and human oral microbiota were colonized with three most common oral bacterial genera: *Streptococcus*, *Haemophilus* and *Veillonella*, and shared relatively conserved communities to each other. A number of bacteria related to human pathogens were consistently detected in macaques. The findings delineate the range of structure and diversity of microbial communities in a wild macaque population, and enable the application of macaque as an animal model for future characterization of microbes in transmission, genomics and function.

## Introduction

The human body harbours a diverse indigenous microbial community (microbiota) that interacts with host environment and supplies crucial ecosystem services to benefit the healthy individuals^1–3^. Interruption of symbiotic balances of microbiota in component and abundance of distinct organisms has been linked to several human diseases, such as bacterial vaginosis^4^, carcinogenesis^5^, diabetes^6^, inflammatory bowel disease (IBD)^7^ and obesity^8^. Restoration of a beneficial microbial structure or function provides emerging therapy for certain diseases. For example, fecal microbiota transplantation (FMT) has provided a much-improved outcome changed the standard of care for recurrent *Clostridium difficile* colitis^9^. Ongoing efforts in identifying the genome and function of individual species have particular health benefits. Besides, studies on microbe-microbe and microbe-host interactions in light of evolutionary and ecological theory provide unique insight in understanding the role of microbes and the benefits they provide to hosts^3,10,11^.

Non-human primates (NHPs) are often used as an animal model in medical research because they share various behavioural, physiological and genetic similarities with humans^12,13^. These similarities extend to the gut, skin and genital microbiota with surveys indicating shared microbial components common to non-human primates and humans, but also the co-speciation of (gut) microbiota with their respective hosts^14–18^. Hence, NHP-associated microbiome differs from the human counterparts in various ways. For example, the typical gut microbiota in rhesus macaques and baboons resembles a diseased state in humans as it contains higher relative abundances of Proteobacteria as opposed to the more dominant Bacteroidetes and Firmicutes in healthy humans^19–21^. Similarly, vaginal communities in a number of surveyed non-human mammals do not have comparable abundances of *Lactobacillus* as do their human counterparts^22^ but usually harbour microorganisms associated with bacterial vaginosis in humans^23^, although low pH conditions and bacterial diversity in the human vagina may be an exception rather than the norm in other mammals^24^.

A recent survey comparing the gut bacterial communities of wild, semi-captive and captive doucs and howler monkeys with humans indicated that captivity biased the gut microbiota composition in these non-human primates towards a ‘humanized’ profile^25^. For a better understanding of the indigenous microbiota in non-human primate animals, in this study, we collected paired oral, anal and vaginal swabs from a wild macaque population inhabiting a protected nature reserve in Hong Kong and profiled their microbial communities using 16S rRNA gene amplicon sequencing. The data were compared to their human counterparts from the Human Microbiome Project to define the similarity and difference across body sites and host species. Since these animals were part of a contraceptive operation project on the macaques, and 79% of adult female animals had already undergone tubectomy before they were re-captured for the veterinary inspection, the changes of macaque vaginal microbiota in response to contraception were also compared.

## Results

### Diversity of microbial composition in healthy macaques and humans

Oral, perianal and vaginal (female only) swabs were collected from 117 rhesus macaques (84 females and 33 males) (Tables 1 and S1). Samples from one female animal in pregnancy was excluded from this study. All surveyed animals appeared healthy according to the body condition and temperature measured by veterinarians. We performed amplicon sequencing on the V4 region of the 16S rRNA gene, producing 13,583,251 high-quality sequences from a total of 318 samples using an Illumina MiSeq sequencing (mean 42,715 ± 15,848 reads per sample). In order to describe the surveyed macaque-associated microbial communities in the context of the human counterparts, we downloaded 2,689,425 16S rRNA gene V3-V5 sequences from the Human Microbiome Project (HMP) (SRA accession PRJNA48489) (Table S2). These HMP 16S sequences were generated by 454 pyrosequencing from 420 samples (mean 5,110 ± 2,652 reads per sample) consisting of paired saliva, stool and vaginal (female only) sites from 173 healthy human individuals (74 females and 99 males)^3,26^. All samples with high-quality reads less than 2,000 were removed from the further analysis (Table S1).

**Table 1:** Breakdown of number of samples included in this study.

Cohort	NCBI SRA	16S region	Subject #	Sample #	Anal		Oral		Vaginal
					Female	Male	Female	Male	Female
Macaques	PRJNA411767	V4	116	314	83	32	83	33	83
Humans	PRJNA48489	V3-V5	173	399	70	88	72	96	73
Total			290	713	153	120	155	129	156

**Figure 1:**
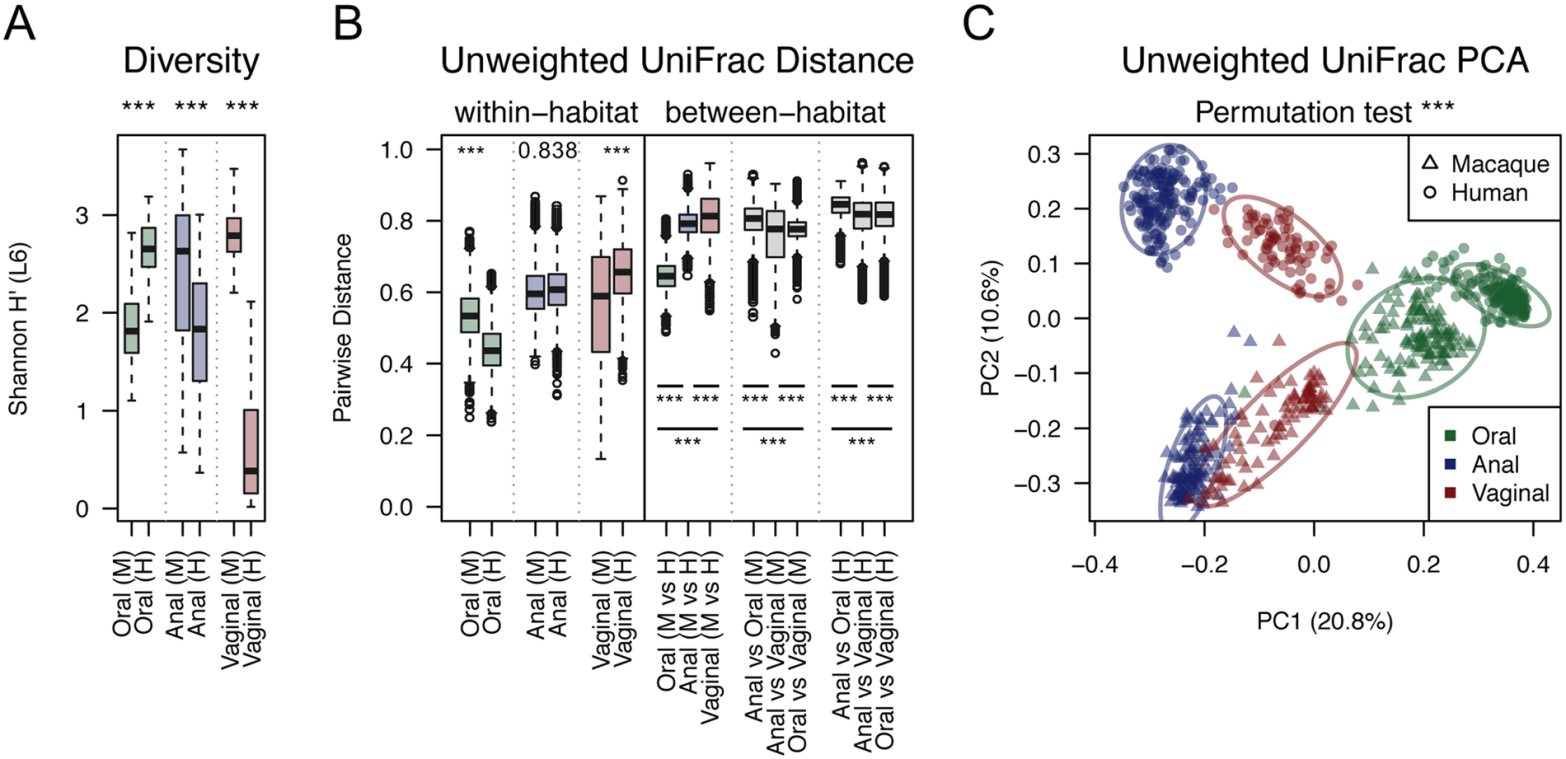
Diversity of macaque microbiome comparing with their human counterparts. (**A**) Alpha diversity of microbiota as measured using the relative inverse Shannon index of genus level 16S rRNA gene phylotypes between macaque and human body sites. (**B**) Beta diversity of microbiota within and between body sites based on unweighted UniFrac pairwise distances. (**C**) Principal coordinate plot of unweighted UniFrac distances showing ecological clustering of microbiota by body sites and host species. Asterisks denote significance at ^*^P < 0.05, ^**^P < 0.01 and ^***^P < 0.001.

Using a threshold of 97% sequence similarity, we identified 755 and 1,644 operational taxonomic units (OTUs) from the human and macaque cohorts, respectively. The samples showed variable species richness when comparing the three body sites (rarefiled to 2,000 reads per samples), but the variability was independent of host species indicating there was unlikely obvious batch bias associated with these two datasets (Fig. S1). Microbial community composition was represented using unweighted UniFrac distances as this metric resulted in more distinct clustering of samples by body site and host species in ordination analysis when compared to the weighted UniFrac distances (entire variance, 94.886 *vs* 83.761) (Fig. S2). A principal component ordination indicated that the sample clustering was driven more by the presence/absence of bacterial OTUs (unweighted) rather than the proportion of microbial community members (weighted). Overall, the microbial community diversities were compositionally distinct with respect to body sites and host species (Fig. 1A). For example, human vaginal samples had the lowest OTU richness and Shannon diversity (at the genus level) among habitats due to a dominance of *Lactobacillus* (mean abundance of 78.65 ± 3.36%) which was rare in the macaque vagina (0.35 ± 0.07%). Macaque anal microbiota was significantly more diverse when compared to the human stool community (mean Shannon index of 2.42 vs 1.78, p < 0.001). In contrast, the surveyed macaques shown less diversity of the oral microbial community to the humans (1.85 vs 2.65, p < 0.001). From the principal component ordination, we observed that oral microbiota showed the least within-host and between-host variability when compared to anal or vaginal communities (green boxplots in Fig. 1B), implying relatively higher similarity of oral microbiota composition between macaque and human hosts. Using the *adonis* function in R’s package ‘*vegan*’, a permutational multivariate analysis of variance (PERMANOVA) based on unweighted UniFrac distances indicated that approximately 46% of variation in microbial community composition could be attributed to body site (Df = 2, R^2^ = 0.2627, pseudo *F* = 170.8, p < 0.001), host species (Df = 1, R^2^ = 0.0864, pseudo *F* = 112.3, p < 0.001) and the combination of site and host (Df = 2, R^2^ = 0.1071, pseudo *F* = 69.6, p < 0.001), which supported the observations from the principal component ordination that microbial variation was mainly associated with both site and host species (Fig. 1C). Gender was not significantly associated with the composition of oral and anal microbial communities (R^2^ = 0.0022, p = 0.167) (Fig. S3).

### Site-by-site genus-level comparison of microbial communities between hosts

**Figure 2:**
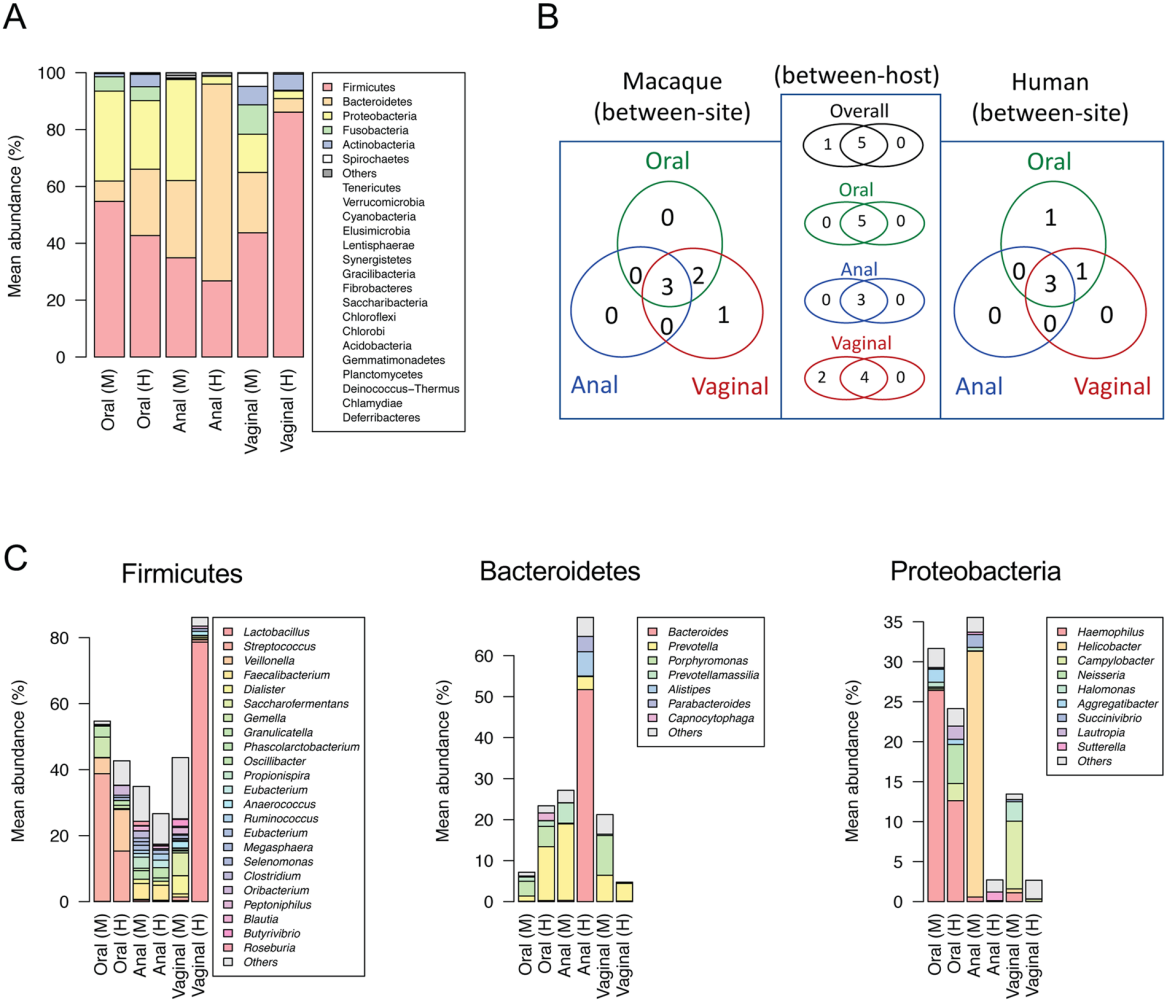
Distribution of microbial taxa between macaque and human body sites. (**A**) Distribution summarized at the phylum level. The less dominant phyla with mean abundance <1% in any of the respective habitats were combined. (**B**) Venn diagram showing the number of phyla present in unique or multiple habitat(s). The six phyla with mean abundance >1% in any of respective habitats were counted. (**C**) Distribution of genera among OTUs within each of three most predominant phyla Firmicutes, Bacteroidetes and Proteobacteria. Genera with <1% mean abundance in the respective habitats are represented by the single group “Others”.

At the phylum level, composition of microbial communities associated with the surveyed macaques largely resembled those in the corresponding body sites of their human counterparts. The macaque microbiota was predominantly composed of Firmicutes, Bacteroidetes and Proteobacteria which collectively accounted for 93.5% mean abundance in oral cavity, 97.6% in anus and 78.4% in vagina (Table 2, Fig. 2A,B). Particularly, Firmicutes accounted for 43.6% of overall 16S sequence reads and were detected in every surveyed macaque and human samples across the body sites (taking into account OTUs with >1% relative abundance in at least one sample). Proteobacteria and Bacteroidetes were commonly present in macaque samples (>98% and >94% prevalence at >1% relative abundance, respectively) but they were less prevalent in human vagina (44% and 37% prevalence at >1% relative abundance, respectively). Besides, Fusobacteria and Actinobacteria were relatively common in the oral cavity and vagina but rare in anus samples. Spirochaetes seems to be unique to macaques and were found in 60% of vagina samples (at >1% relative abundance). Members of other phyla, such as Tenericutes, Verrucomicrobia and Saccharibacteria (TM7) were less prevalent and detected at relatively lower abundances (combined mean abundance <0.5%) (Fig. 2A).

**Table 2:** Relative abundance of bacteria taxa at the phylum level at the respective body site of macaques and humans.

Body site	Phylym	Macaque		Human		MW test (p)	LDA score		
		Abundance^$^ (%)	Prevalence* (%)	Abundance^$^ (%)	Prevalence* (%)		Macaque	Human	p value
Oral	Actinobacteria	1.11 ± 0.14	27.6	4.36 ± 0.23	94.6	1.03E-30	—	4.2202	1.41E-30
	Bacteroidetes	7.16 ± 0.60	94.0	23.37 ± 0.67	100.0	1.31E-35	—	4.9048	1.78E-35
	Firmicutes	54.71 ± 1.21	100.0	42.71 ± 0.85	100.0	1.04E-13	4.7883	—	7.84E-14
	Fusobacteria	5.04 ± 0.43	89.7	4.88 ± 0.28	94.6	3.80E-01	—	—	—
	Proteobacteria	31.66 ± 1.10	100.0	24.12 ± 0.87	100.0	4.57E-08	4.5592	—	6.36E-08
	Spirochaetes	0.19 ± 0.04	2.6	0.34 ± 0.05	6.5	1.16E-02	—	2.9318	8.79E-03
	Tenericutes	0.04 ± 0.01	0.0	0.19 ± 0.04	5.4	4.57E-01	—	—	—
Anal	Actinobacteria	0.37 ± 0.04	4.3	0.15 ± 0.03	2.5	2.14E-19	3.3448	—	3.01E-19
	Bacteroidetes	27.13 ± 1.55	97.4	69.22 ± 1.40	100.0	3.95E-37	—	5.3210	5.45E-37
	Cyanobacteria	0.17 ± 0.03	3.5	0.06 ± 0.03	2.5	3.88E-30	3.1827	—	6.03E-30
	Elusimicrobia	0.08 ± 0.06	1.7	0.00 ± 0.00	0.0	1.64E-10	3.3329	—	1.84E-10
	Firmicutes	34.92 ± 1.61	100.0	26.77 ± 1.35	100.0	3.55E-05	4.5929	—	4.00E-05
	Fusobacteria	0.23 ± 0.11	3.5	0.06 ± 0.04	1.9	2.49E-24	3.2631	—	3.47E-24
	Proteobacteria	35.55 ± 2.84	100.0	2.72 ± 0.26	59.5	7.59E-37	5.2210	—	6.37E-37
	Spirochaetes	0.76 ± 0.07	29.6	0.00 ± 0.00	0.0	6.45E-55	3.6808	—	1.12E-54
	Tenericutes	0.55 ± 0.11	14.8	0.62 ± 0.14	15.8	1.07E-10	—	3.3040	5.32E-11
	Verrucomicrobia	0.19 ± 0.03	5.2	0.38 ± 0.12	8.9	1.01E-10	—	3.3977	1.45E-10
Vaginal	Actinobacteria	6.53 ± 0.78	95.2	5.66 ± 1.58	30.1	1.28E-09	3.8415	—	2.17E-09
	Bacteroidetes	21.22 ± 1.10	100.0	4.75 ± 1.14	37.0	4.71E-19	4.9075	—	7.85E-19
	Cyanobacteria	0.03 ± 0.01	0.0	0.08 ± 0.02	1.4	5.92E-01	—	2.2814	4.53E-01
	Firmicutes	43.68 ± 1.55	100.0	86.12 ± 2.38	100.0	2.51E-20	—	5.3250	4.13E-20
	Fusobacteria	10.33 ± 1.20	84.3	0.27 ± 0.14	5.5	3.03E-22	4.6957	—	5.11E-22
	Proteobacteria	13.45 ± 1.07	97.6	2.70 ± 0.54	43.8	1.35E-17	4.7479	—	2.28E-17
	Spirochaetes	4.56 ± 0.61	60.2	0.00 ± 0.00	0.0	3.16E-27	4.3492	—	5.60E-27
	Synergistetes	0.00 ± 0.00	0.0	0.01 ± 0.01	1.4	2.92E-01	—	2.0346	2.87E-01
	Tenericutes	0.15 ± 0.03	3.6	0.40 ± 0.19	6.8	2.37E-03	—	2.9840	1.28E-03

^$^Mean ± s.e.m.

^*^Individual samples with >1% abundance were counted.

**Figure 3:**
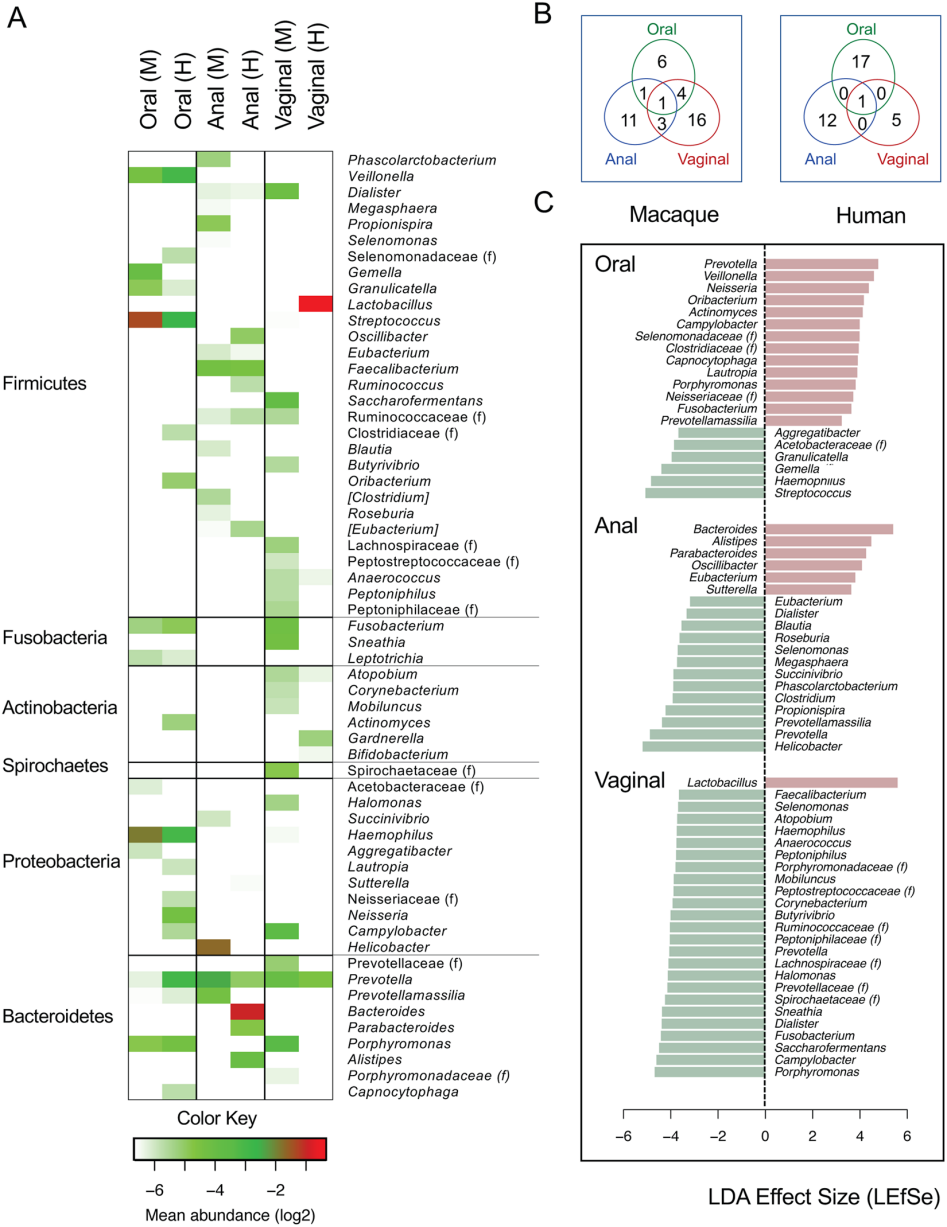
The “core” dominant bacterial taxa in each body site of macaques and humans. (**A**) Heat map showing relative abundances of the dominant taxa in each body site in macaques and humans. Using OTUs summarized at the genus level, a total of 59 “core” bacterial taxa (at the genus, family or higher levels) comprising >1% mean abundance in any of the respective habitats were included. Scale bar represents log2 transformed relative abundance values, with highest value of −0.34 (a log2 of 79%) in red and lowest value of −6.64 (a log2 of 1%) in blue. (**B**) Venn diagram showing the number of “core” bacterial taxa in each body site of the surveyed macaques (left) and humans (right). (**C**) Linear discriminant analysis (LDA) for effect size (LEfSe) listing bacterial taxa that best discriminated macaque- (minus values in green) or human-associated microbial communities (positive values in red). A higher LDA score reflects higher relative abundances.

The majority of bacteria belong to three phyla (Firmicutes, Bacteroidetes and Proteobacteria); however, the microbial components at the genus level were highly diverse, composed of distinct taxa shaping the differences of communities by habitats (Table S3, Fig. 2C). Using OTUs summarized at the genus and higher taxonomic levels (QIIME L6), we defined 59 “core” bacterial taxa comprising >1% mean abundance in any of respective habitats (e.g., oral, perianal or vaginal) surveyed in this study (Fig. 3A, Table S3). Each community was predominantly represented by one or a few of bacterial taxa distinguishing from other habitats. For example, *Bacteroides* was detected in all human stool samples (100% prevalence of >1% relative abundance) with a mean relative abundance of 51.1 ± 1.8%, while *Helicobacter* was the most dominant anal bacteria in the surveyed macaques, accounting for 30.9 ± 2.9% mean relative abundance, and was detectable in 97% of animals (>1% relative abundance) (Figs 3A and S4). The oral cavity of both macaques and humans mainly harboured *Streptococcus* and *Haemophilus*, but the composition of less dominant taxa was different: *Gemella* was more common in macaques (mean abundances of 6.2% vs 1.0%, p < 0.001), and *Prevotella* (1.3% vs 13.1%, p < 0.001) in humans. Some taxa could be found in multiple habitats (Fig. 3B, Table S3). For example, *Prevotella* was present in all surveyed communities, although the relative abundance within each habitat was variable. There were four “core” bacterial taxa shared between macaque oral and vaginal sites (*Porphyromonas*, *Streptococcus*, *Fusobacterium* and *Haemophilus*), three between anal and vaginal sites (Ruminococcaceae, *Selenomonas* and *Dialister*), and one between oral and anal sites (*Prevotellamassilia*). In contrast, the human microbiota from different communities were less overlapped (Fig. 3B).

#### Oral cavity

There were 12 and 18 “core” OTUs enriched in macaque and human oral communities, respectively, with nine shared between host species (Tables S3 and S4, Figs 3, S4 and S5). The most predominant oral bacteria were *Streptococcus*, with relative abundances of 4.5–87.9% (mean abundance of 38.7 ± 1.4%) in macaques, and 2.4–47.3% (15.3 ± 0.7%) in humans. *Haemophilus* and *Veillonella* were the second and third most common genera found in >99.4% and >78% oral samples (at >1% relative abundance), respectively, constituting the mean relative abundances of 26% and 5% in macaques, and 13% and 13% in humans. Using a linear discriminant analysis (LDA) for effect size (LEfSe), we found 20 “core” oral bacteria discriminatively associated with hosts, including 6 subsets predominant in macaques (*Streptococcus*, *Haemophilus*, *Granulicatella*, *Gemella*, Acetobacteraceae and *Aggregatibacter*), and 14 in humans (*Prevotella*, *Veillonella*, *Neisseria*, *Oribacterium*, *Actinomyces*, *Campylobacter*, Clostridiaceae, Selenomonadaceae, *Capnocytophaga*, *Lautropia*, *Porphyromonas*, Neisseriaceae, *Fusobacterium* and *Prevotellamassilia*) (Fig. 3C). *Streptococcus* was negatively correlated with other “core” taxa indicative of strong depression in the overgrowth of *Streptococcus* linked to the presence of other bacteria (Fig. S6).

#### Anus

At the phylum level, human stool communities were dominated by Bacteroidetes (69% mean abundance) and Firmicutes (27%) whereas macaque anal samples had approximately equal relative abundances of Bacteroidetes (27%), Firmicutes (35%) and Proteobacteria (36%) (Table 2, Fig. 2). A large proportion of genera belonging to the phylum Firmicutes, such as *Dialister*, *Eubacterium*, *Faecalibacterium* and *Phascolarctobacterium*, were detected in both macaques and humans (Tables S3 and S5, Figs 3A, S4 and S5). In contrast, members of Bacteroidetes were differentially enriched between two hosts. We detected higher relative abundances of *Prevotella* and *Prevotellamassilia* in macaques in contrast to the human counterparts where *Bacteroides*, *Alistipes* and *Parabacteroides* were largely enriched. One striking feature of the macaque anal microbiota was its high predominance of proteobacterial OTUs. *Helicobacter* accounted for 87% of proteobacterial sequences in macaque anal samples and was found in 97% samples (at > 1% relative abundance), but were extremely rare in healthy human stool samples. Using LEfSe, we classified 19 “core” bacterial genera that discriminated macaque and human anal communities, with *Helicobacter* dominating in macaques (31% mean relative abundance) and *Bacteroides* (51%) in humans (Fig. 3C). These two bacteria were negatively correlated with other “core” taxa in the relevant host habitats (Fig. S6).

#### Vagina

The human vaginal microbiota was highly enriched by *Lactobacillus* (79% mean abundance), a striking contrast to the macaque vagina which was not obviously dominated by any single genus (Figs 2, 3, S4 and S5). The macaque vaginal microbiota was extremely more diverse than their human counterparts (p < 0.001) (Fig. 1A) and was represented by at least 24 discriminative taxa belonging to 6 phyla (Table S3 and S6). More than 50% of 16S sequence reads from macaque vaginal samples were assigned to *Porphyromonas*, *Campylobacter*, *Saccharofermentans*, *Prevotella*, *Dialister*, *Fusobacterium* and *Sneathia* that collectively comprised 4.9% to 9.6% mean abundance. The macaque and human vaginal communities showed a limited degree of overlap to each other, with notable distinctions being the enrichment of *Prevotella* (mean abundance of 6.7% vs. 4.3%, p < 0.001), *Atopobium* (2.1% vs. 1.2%, p < 0.001), and *Anaerococcus* (1.9% vs. 1.2%, p < 0.001) in macaque relative to human.

### OTU-level segregation of pathogenic microbes in macaques

Several bacteria detected in macaque samples are potentially designated as known human pathogens, the most notable being *Fusobacterium* in oral cavity, *Helicobacter* in anus and *Gardnerella* in vagina. In order to determine whether the macaque OTUs represented opportunistic “pathogens” define by the Pathosystems Resource Integration Center (PATRIC), we aligned their 16S as well as sequences from the human samples to the species classification. In total, 40 of 461 PATRIC pathogens were detected at >1% prevalence of >0.1% relative abundance in the surveyed human and macaque samples (Table S7). Class A-C pathogens defined by the National Institute of Allergy and Infectious Diseases (NIAID) such as *Bacillus anthracis*, *Clostridium botulinum*, *Francisella tularensis* and *Yersinia pestis* recorded less than 0.1% relative abundance in any surveyed macaque and human individuals. Most of detectable pathogens were niche-specific and broadly distributed in healthy human subjects, and some taxa could be found in both hosts with low relative abundance. For example, *Bacteroides vulgatus* was detected in 4 macaque anal samples while this taxon was nearly ubiquitous in humans (prevalence of 94%, 21% and 15% in human stool, oral and vaginal samples at >0.1% relative abundance, respectively). *Helicobacter* was the most dominant genus in the surveyed macaque anal community, but these OTUs showed <97% similar to *H. pylori* based on the alignment of the 16S V4 region and were mostly identical to *H. fennelliae* (NCBI accession number of NG_042880) and *H. macacae* (NG_042884). In vaginal samples, 22% and 28% of macaque and human subjects (at >0.1% relative abundance) contained 16S sequences >97% identical to *Gardnerella vaginalis*, a species of anaerobic bacteria associated with bacterial vaginosis and disruption of the normal human vaginal microflora. *Campylobacter ureolyticus* and *Streptococcus agalactiae* were two other pathogens found in macaque and human vaginal samples. Six macaque oral pathogens were potentially zoonotic, including *Aggregatibacter actinomycetemcomitans*, *Capnocytophaga gingivalis*, *Porphyromonas gingivalis*, *Rothia mucilaginosa*, *Tannerella forsythia* and *Treponema denticola*. *Streptococcus pseudopneumoniae* were common in macaque oral cavities (>82% prevalence with >0.1% relative abundance) but nearly absent in healthy human individuals. It is important to note that these inferences are based on the V4 region of the 16S gene, which has limited resolution in determining taxonomy at species level. Ideally, presence of these putative pathogens in macaque samples should be verified with full length 16S rRNA gene sequences.

### Functional profile of the macaque microbiota

**Figure 4:**
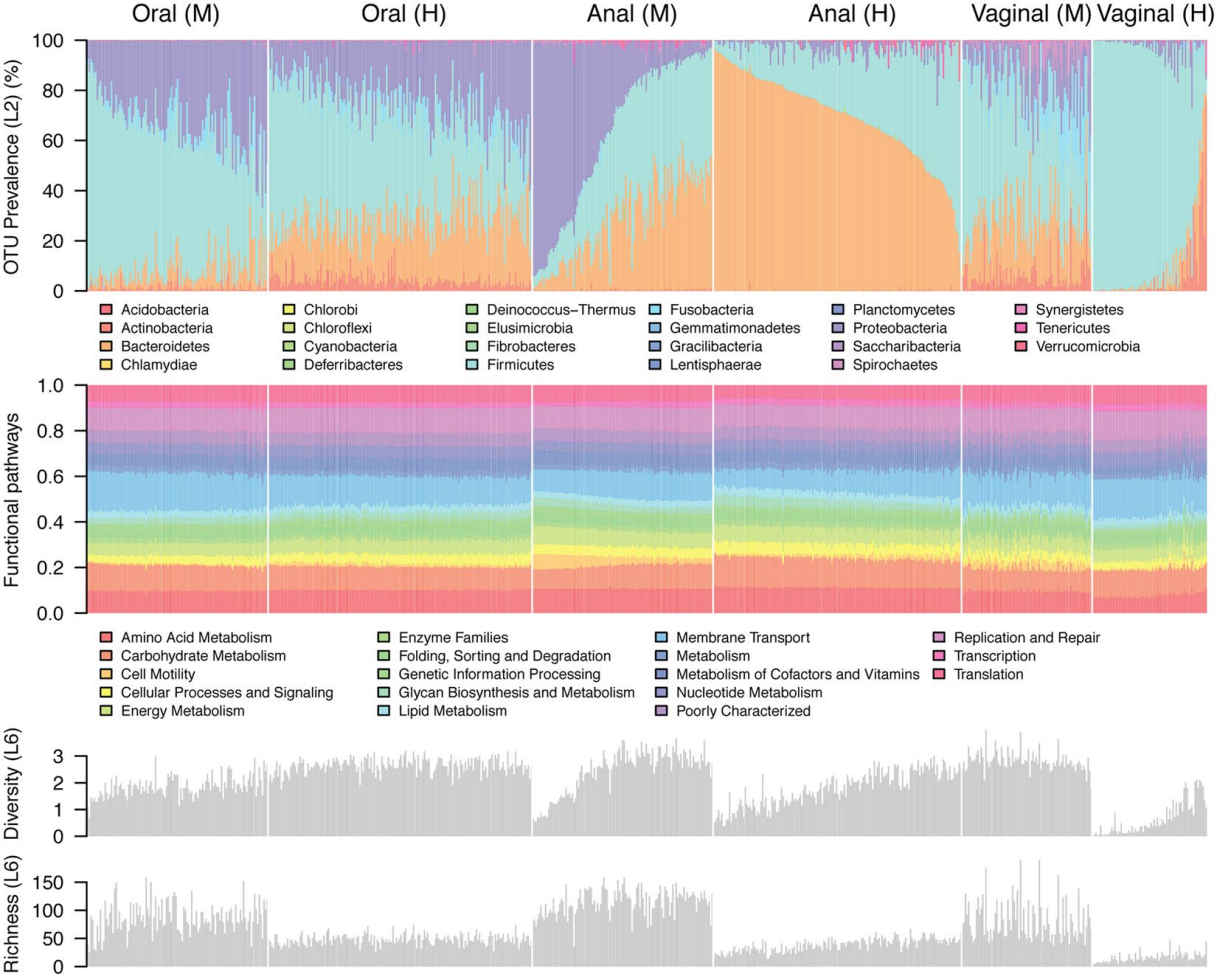
Microbial community composition varies between body sites and hosts while predicted metabolic pathways remain stable. Vertical bars represent the relative abundances of microbial taxa summarized at phylum level (denoted by colours) or PICRUSt-predicted KO pathway. Shannon diversity and richness metrics were calculated based on OTUs summarized at the genus level.

Since the microbial community compositions of the surveyed macaques and humans were most heavily influenced by body site, we hypothesized that physiological similarities between the same body sites between macaque and human may enrich for functionally similar microbial communities. Hence, we used Phylogenetic Investigation of Communities by Reconstruction of Unobserved States (PICRUSt) to predict functional pathways based on the composition of the microbial communities and produced Kyoto Encyclopaedia of Genes and Genomes (KEGG) Orthology (KO) abundance profiles for each sample. These KO abundance values were then summarized into KO pathways based on their involvement in metabolic pathways as described in the KEGG database. A PERMANOVA performed on variance stabilized-transformed KO profiles indicated that body site had the largest influence on predicted KO abundance followed by host species in descending size of effect (p < 0.001), supporting our hypothesis that body sites selected for functionally similar communities irrespective of host species (albeit in humans and macaques which share many physiological similarities). The hypothesis was also supported by KO profiles between samples showing less variability compared to community composition (Fig. 4). Similar to community composition, KO profiles were not associated with gender in oral cavity and anal sites when vagina samples were excluded from the analysis (p = 0.519). Results of the summarized KO profiles were supported by sparse partial least squares discriminant analysis (sPLSDA) using the first three ordination components that show clustering of samples mainly by body sites (Fig. S7).

Next, we attempted to identify the metabolic functions that discriminated between communities of the body sites. When comparing corresponding body sites between macaque and human hosts, there was consistently increased metabolism associated with plant fiber degradation (e.g., butanoate, glycerophospholipid, propanoate and pyruvate) in macaque relative to human, which was supported by the more relatively abundant chloroplast sequences observed in macaque 16S sequences (Fig. S8). This observation suggested that the surveyed macaques primarily consumed fiber-rich plant material in contrast to humans with more diversified diets. Other pathways related to amino acid and lipid biosynthesis/metabolism and energy production such as glycolysis, pentose phosphate pathway and the citrate cycle, however, showed small and varying associations with hosts, suggesting macaques and humans may share highly similar microbial metabolism in the same body sites (Tables S8–S10). We suspect the limited resolution of partial 16S rRNA gene in discriminating bacterial phylotypes, as well as a possible lack of macaque-specific PICRUSt reference microbial genomes may have restricted the utility of this analysis, as relatively higher scores of the weighted Nearest Sequenced Taxon Index (NSTI) were observed in macaque datasets when compared to the human datasets (oral: mean of 0.0484 *vs* 0.0408, p = 0.7783; anal: 0.0864 *vs* 0.0799, p = 0.0172; vaginal: 0.0834 *vs* 0.0240, p < 0.001) (Table S11).

### Macaque microbiota composition affected by age and tubectomy

**Figure 5:**
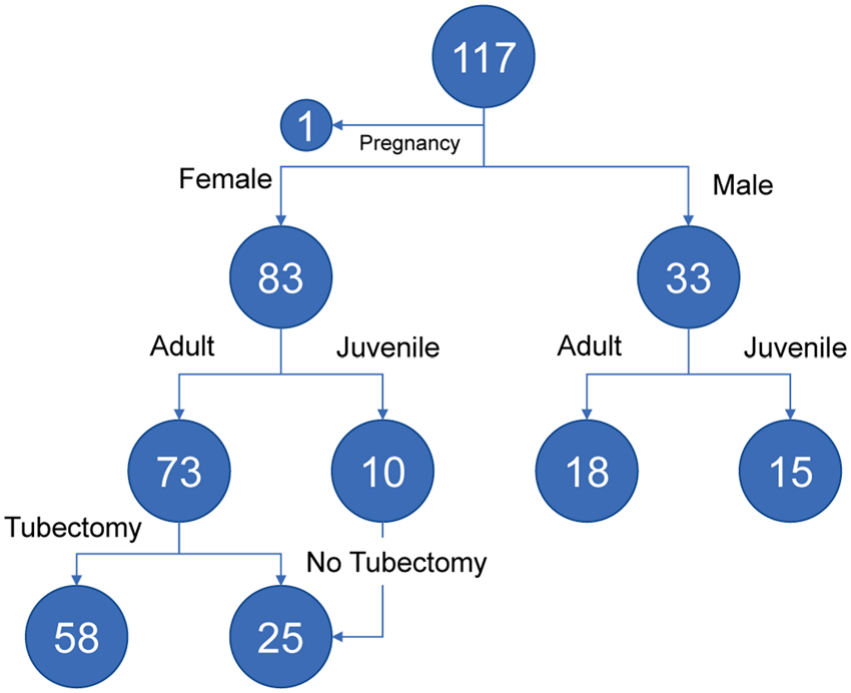
Distribution of macaque specimens according to gender, age and contraception.

**Figure 6:**
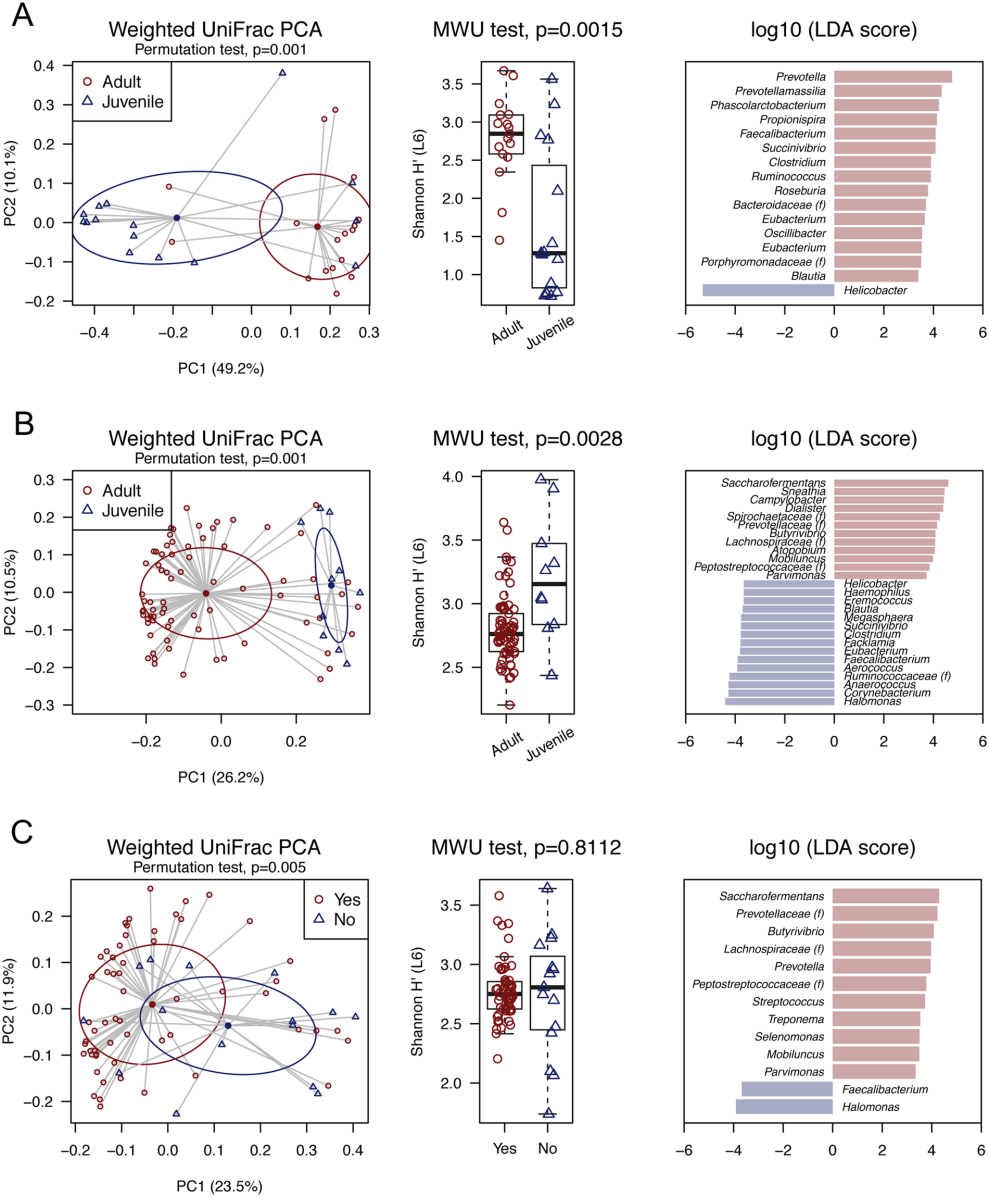
Comparison of macaque microbial communities showing the difference of (**A**) anal microbiota between juvenile and adult male macaques, (**B**) vaginal microbiota between juvenile and adult female macaques, and (**C**) vaginal microbiota between adult femal macaque with and without tubectomy. (left) Permutational multivariate analysis of variance (PERMANOVA) using a weighted UniFrac distance, (middle) beta diversity between compared groups, and (right) linear discriminant analysis (LDA) for effect size (LEfSe) listing bacterial taxa that best discriminated defined groups. MWU test, Mann–Whitney U test.

The surveyed macaque animals were grouped into adult and juvenile, based on body weight (female: mean of 6.0 kg *vs* 3.5 kg; male: 7.7 kg *vs* 4.4 kg) and length (female: mean of 43.9 cm *vs* 36.4 cm; male: 47.5 cm *vs* 38.5 cm) (see Fig. 5 for the sample size). There was no significant difference of the composition of oral microbiota between juvenile and adult macaques (data not shown). However, a dramatically decreased anal microbiota diversity was observed in juvenile male macaques compared to their adult counterparts (Shannon index, mean of 1.65 *vs* 2.79, p = 0.0015) (Fig. 6A), which inversely was not detected between female age groups (2.53 *vs* 2.48, p = 0.8631). Consistent with the distinct clustering of anal microbial communities between juvenile and adult male macaques (using weighted UniFrac distance, Df = 1, R^2^ = 0.1838, pseudo *F* = 7.0, p = 0.003), the young animals were predominantly colonized with *Helicobacter* (mean relative abundance of 59% *vs* 12%) though this bacterium was extremely common in both age groups (93% and 88% prevalence at >1% relative abundance) (Tables S5 and S12).

Similarly, significant differences of the vaginal microbiota diversity (3.21 *vs* 2.77, p = 0.0028) and community composition (Df = 1, R^2^ = 0.0907, pseudo *F* = 8.1, p = 0.001) between juvenile and adult (female) macaques were observed (Fig. 6B), with 15 and 12 bacteria taxa (>1% mean relative abundance in any of age groups) discriminating juvenile and adult animals, respectively (Table S13). Since these adult animals were part of a contraceptive operation project on the macaques, we hypothesize that the altered estrogen hormone may affect the vaginal microbial composition. 79% (58/73) of adult female macaques had already undergone tubectomy before they were re-captured for the veterinary inspection and sample collection (Fig. 5). Interestingly, PERMANOVA test using a weighted UniFrac distance well supported the separation of vaginal microbiota communities between adult macaques with and without tubectomy (Df = 1, R^2^ = 0.0441, pseudo *F* = 3.3, p = 0.005) (Fig. 6C). We also found a relatively decreased vaginal microbiota diversity in the contracepted animals (2.75 *vs* 2.81, p = 0.8112), although the statistical test was not significant. When vaginal microbial communities from juvenile macaques, adult without tubectomy, and adult with tubectomy were compared, a number of bacteria taxa showed constant increase (N = 7) or decrease (N = 5) of the relative abundances across groups (>1% mean relative abundance in any of three categorized groups), with the most notable distinctions being the enrichment of *Saccharofermentans* (0.01%, 4.62%, and 8.68%, p < 0.001) in contracepted adults, and *Halomonas* (7.33%, 3.05% and 1.43%, p < 0.001) in juvenile females (Tables 3, S13 and S14).

**Table 3:** Relative abundance of macaque vaginal bacteria between juvenile, adult without tubectomy, and adult with tubectomy.

	Juvenile (N = 10)		Adult (no tubectomy) (N = 15)		Adult (tubectomy) (N = 58)		Adult (total) (N = 73)		MWU test, p value		Kruskal test
	Abundance^$^ (%)	Prevalence* (%)	Abundance^$^ (%)	Prevalence* (%)	Abundance^$^ (%)	Prevalence* (%)	Abundance^$^ (%)	Prevalence* (%)	Age^^^	Tubectomy^#^	p value^&^
*Saccharofermentans*	0.01 ± 0.01	0.0	4.62 ± 1.79	46.7	8.68 ± 0.76	82.8	7.85 ± 0.73	75.3	0.0000	0.0167	0.0000
Prevotellaceae (f)	0.28 ± 0.12	10.0	0.57 ± 0.28	13.3	3.82 ± 0.45	75.9	3.15 ± 0.39	63.0	0.0014	0.0000	0.0000
Lachnospiraceae (f)	0.43 ± 0.15	30.0	1.30 ± 0.55	26.7	3.34 ± 0.47	67.2	2.92 ± 0.40	58.9	0.0091	0.0086	0.0008
*Butyrivibrio*	0.03 ± 0.01	0.0	0.67 ± 0.45	13.3	2.80 ± 0.58	56.9	2.36 ± 0.48	47.9	0.0052	0.0013	0.0001
Peptostreptococcaceae (f)	0.26 ± ± 0.13	10.0	0.73 ± 0.26	20.0	2.03 ± 0.33	55.2	1.76 ± 0.27	47.9	0.0010	0.0055	0.0001
*Mobiluncus*	0.02 ± 0.02	0.0	1.54 ± 0.67	40.0	2.02 ± 0.25	72.4	1.92 ± 0.24	65.8	0.0000	0.0282	0.0000
*Parvimonas*	0.00 ± 0.00	0.0	0.85 ± 0.39	26.7	1.21 ± 0.18	39.7	1.14 ± 0.16	37.0	0.0001	0.0420	0.0000
*Eremococcus*	1.10 ± 0.90	10.0	0.29 ± 0.17	13.3	0.06 ± 0.03	3.4	0.11 ± 0.04	5.5	0.0006	0.0030	0.0001
*Blautia*	1.23 ± 0.27	70.0	0.50 ± 0.19	26.7	0.24 ± 0.09	8.6	0.29 ± 0.08	12.3	0.0002	0.0321	0.0001
*Eubacterium*	1.37 ± 0.34	50.0	0.50 ± 0.23	13.3	0.24 ± 0.09	5.2	0.30 ± 0.08	6.8	0.0000	0.0474	0.0000
*Faecalibacterium*	2.11 ± 0.56	70.0	1.53 ± 0.84	26.7	0.56 ± 0.26	10.3	0.76 ± 0.27	13.7	0.0001	0.0308	0.0001
*Halomonas*	7.33 ± 2.77	80.0	3.05 ± 1.21	46.7	1.43 ± 0.64	13.8	1.77 ± 0.57	20.5	0.0003	0.0127	0.0001

^$^mean ± s.e.m.

^*^Individual samples with > 1% abundance were counted.

^^^Mann–Whitney U test between juvenile and adult (total) groups.

^#^Mann–Whitney U test between adult (no tubectomy) and adult (tubectomy) groups.

^&^Kruskal test test among juvenile, adult (no tubectomy) and adult (tubectomy) groups.

## Discussion

In this study, we surveyed the microbiota of paired oral, anal and vaginal swab samples of 116 non-captive healthy macaques and compared their compositions to the communities of the corresponding body sites of their human counterparts. Overall, the macaque-associated microbial communities were more diverse than humans which was consistent with other surveys of primate rectal swabs^25,27,28^, skin^17^ and vaginal microbial communities^22^. The observations of higher diverse of microbiota in macaques could be attributed to a mix of evolutionary histories and lifestyles in which humans have altered their exposure to microorganisms through means such as diet, medicine, hygiene and lifestyle. For example, human guts show a predominance of *Bacteroides* whereas the surveyed macaque anal samples exhibited increased relative abundances of *Prevotella*. The dominance of *Bacteroides* in humans is often associated with a diet high in protein and fat^29^, whereas *Prevotella* is associated with carbohydrate and fibre^30^. Although we did not have information regarding the specific diet of the surveyed macaques, the dominance of *Prevotella* is in line with a plant-based dietary preference in macaques^31^. This observation was also supported by the increased proportion of fibrolytic genes detected in the surveyed macaques. It was previously inferred that doucs and howler monkeys recruited *Bacteroides* and *Prevotella* into their gut microbiota as a result of reduced dietary plant fibre associated with captivity^25^, suggesting that dietary perturbation could be one of major drivers altering the stool microbial composition.

Another notable observation in the macaque anal samples was the dominance of proteobacterial taxa, namely *Helicobacter*. Using serological assays, Blaser’s group found that Rhesus macaques have widespread exposure to *H. pylori*^32^. Surveys of intestinal microbial communities in healthy captive macaques also indicated the presence of *Helicobacteraceae* OTUs^21,33^, which together with our data suggest that *Helicobacter* are a feature of macaque gut microbial communities. Interestingly, a higher abundance of *Helicobacter* was observed in juvenile male macaque anal samples, in line with the colonization in human beings that *H. pylori* is predominantly acquired during childhood^34^. *H. pylori* in populations in developed countries is disappearing due in large part to changing hygienic standards and antibiotic treatments^35^. Although *Helicobacter* colonization is often linked to diseases in humans, such as peptic ulcer disease, gastric cancer, and mucosa-associated lymphoid tissue lymphoma^36^, reverse associations of *H. pylori* with early-onset asthma^37^ and gastrointestinal infections^38^ demonstrate also beneficial protection of this late-in-life pathogen in early life. The amplified 16S rRNA *Helicobacter* OTUs in macaque anal samples in the current work were most identical to *H. fennelliae* and *H. macacae*; both of them are categorized as enterohepatic helicobacters and have been linked to diarrhoea, bacteraemia and colitis in macaques^39,40^, which may prove useful in understanding the aetiology and pathogenesis of gastrointestinal diseases in humans. As an example of how the gut microbiota composition differs between humans and macaques, however, the dominance of *Helicobacter* in macaque guts emphasises the need to be careful when interpreting findings from macaque animal models. For example, *Helicobacter pylori* are suspected to dampen the effects of a drug used for treating Parkinson’s disease in humans^41^. Although of different species, there is a possibility that the *Helicobacter* in macaques could also influence the outcome of animal trials testing efficacy of medical drugs.

Similar to the anal microbiota, vaginal communities in the surveyed macaques were highly diverse compared to their human counterparts. The finding was consistent with surveys of vaginal microbial communities in a number of non-human mammals, in which the dominance of *Lactobacillus* in the human vagina appears to be an exception rather than the norm^22,24,42^. A proposed mechanism behind the dominance of *Lactobacillus* in the human vagina is through increased glycogen availability, which functions as an energy source for lactobacilli, thereby promoting a low pH environment through lactobacilli-driven lactic acid production^43,44^. In contrast, the macaque lower genital tract is characterized by low levels of glycogen and lactic acid^45^, and previous study of short chain fatty acid content in the vagina of rhesus macaques reported that the main acid constituent is acetate^46^. Based on this finding, we infer that vaginal acidity in the surveyed macaques could be maintained by a variety of bacteria such as *Fastidiosipila*, *Murdochiella*, *Peptoniphilus* and *Dialister* by producing acetic acid to lower pH environment and protect from invasive pathogens. The presence of fermentative bacteria in the macaque vagina could be conserved in NHPs but not humans as the volatile short chain fatty acids produced can be used as scents for communication, social interaction and other olfactory-driven behaviour^47^, which arguably is less important in humans. Alternatively, a shift towards high starch diets of human beings associated with modernisation is probably responsible for lactobacilli dominance through increased vaginal glycogen content, resulting in low pH conditions in their reproductive physiology^24^. The exact reason behind the low microbial diversity in human vaginas, however, are still not well defined. We found reverse association of macaque vaginal microbiota diversity with increasing age and tubectomy contraception. Similarly, studies in pregnant women also demonstrated significantly decreased vaginal microbiota richness and diversity when that of nonpregnant women was compared, supporting potential influence of hormonal changes on the vaginal microbiome^48,49^.

Interestingly, the macaques harboured a group of anaerobic bacteria associated with bacterial vaginosis in woman, such as *Gardnerella* and Peptostreptococcaceae^50,51^. The role of *Prevotella* in the vagina is less understood; however, it has been reported that *Prevotella* can assist the growth of *Gardnerella vaginalis* and *Peptostreptococcus anaerobius* by producing key nutrients such as ammonia and amino acids for these pathogens^52,53^. Another notable observation was the dispersal of periodontal bacteria, such as *Fusobacterium* and *Porphyromonas* in macaque vaginal microbial community. Several pathogens within *Fusobacterium* and *Porphyromonas* genera have been associated with a variety of oral diseases even cancers in humans^54^. Although a higher diversity of vaginal microbiota may enhance the buffer to protect host, macaques may also suffer bacterial vaginosis or other diseases if these opportunistic “pathogens” dominate the community. In contrast, the lactobacilli dominance, in turn, the low diversity of vaginal microbial community appears to be unique to humans and is hypothesized to benefit women by producing lactic acid to reduce pathogenic invasion^55^. Continuing investigation on non-human vaginal microbial communities may improve our understanding of the “uniqueness” of human vaginal microbiota on both the physiological mechanisms and the broad evolutionary processes.

Both macaque oral swabs and human saliva samples shared the same three most predominant taxa: *Streptococcus*, *Haemophilus* and *Veillonella*. The lack of comparable distinction in oral microbial communities between macaque and human relative to the anal or vaginal site could indicate an evolutionary origin that oral microbiota has experienced similar selective pressures since the split of macaques and humans from their most recent common ancestor during million years of evolution^56^. Since the oral cavity functions as the first step in food digestion and is constantly exposed to the environmental selection, the microbiota at this site could be more influenced by generic saccharolytic and/or proteolytic microorganisms that respond to perturbations such as diet patterns, oral hygiene or foreign invasion. For example, the surveyed animals in this study had the chance to obtain food from household waste or tourists, resulting in accompanied oral microbiota responses to diet shift as that of humans. In contrast, host physiological and biological factors, such as body temperature, immunology and salivary content, likely do not exert significant selective forces on the oral microbiota for colonization and diversification compared to flora of the gastrointestinal or genital tracts. This hypothesis could be tested by comparing the oral microbiota in a larger cohort of animal species to determine whether oral microbial communities share more convergent lineages than other body sites. Nevertheless, interplays of oral microbiota with host immunodeficiency, lifestyle and other pathogens may also have detrimental consequences for the homeostasis of community, resulting in differential enrichment of certain bacteria between hosts. For example, there was a marked absence of actinobacterial lineages such as *Actinomyces* in the macaque oral swabs, which based on human data^57,58^ would suggest that they suffer from periodontal disease.

These observations collectively indicate that rhesus macaques can be used as an animal model to study microbiota-associated human diseases; however, experimental outcomes will have to be interpreted with caution as the resident macaque microbiota is dramatically different as their human counterparts. With the growing appreciation for viral-bacterial interactions in the context of human health^59^, precautions should also apply to the study of simian/human immunodeficiency virus in rhesus macaques as they are commonly used as the animal model for research into acquired immune deficiency syndrome^60^.

One of the shortcomings of this study is the lack of metagenomic data to predict the comprehensive metabolisms and functions, although our observation based on the limited reference supports the findings from human and other macaque-associated microbial community surveys. Another challenge in comparing the microbiota between macaques and humans in this study is the data sources with different processing methods, which may present batch effects. In order to minimize study bias, we used a closed-reference approach to cluster 16S reads from different datasets separately, and applied a maximum likelihood phylogenetic algorithm to assign bacteria taxonomy. The rarefaction curve also indicated that the variability was less relevant to batch bias. Indeed, the macaque microbial communities characterized in this study are largely concordant with previous reports from a number of macaque animal groups. The third caveat of the present study is the use of slight different body locations, for example, perianal swabs versus stool samples, to compare anal microbial communities between macaque and human. However, micro-niches from the same body site usually harbour stable microbial communities composed of predominant taxa consistent in healthy individuals^21^. Nevertheless, the present study is to our knowledge the first and largest survey of microbiota on non-captive macaque with samples from paired body sites from the same individuals. Our findings elucidate the diversity and structure of microbiota in an apparently healthy macaque population and defined the “core” indigenous bacterial taxa relevant to body sites and hosts species, which forms a basis for further investigation into the host-microorganism co-evolution and the causality of bacterial pathogens in diseases. The presence of microorganisms related to human pathogens in primate animals suggest that they are a native feature; distinction of the predominant microbiota between macaque and human highlights the need to interpret outcomes of animal model studies with caution.

## Methods

### Ethics approval and consent to participate

The programme has been approved by the AFCD and presented to the Animal Welfare Advisory Group (AWAG). All experiments were performed in accordance with the relevant guidelines and regulations.

### Macaque sample collection, DNA extraction and sequencing

Macaque samples were collected from Kam Shan Country Park, Hong Kong (22° 21′ 51.0″N, 114° 9′ 1.0″E), a 339 hectare protected area inhabited by breeding heterosexual troops of rhesus macaques (*Macaca mulatta*) and hybrids between rhesus and long-tail macaques (*M. fascicularis*) consisting of an estimated 1,000 individuals. To control the population of macaques, the Agriculture, Fisheries and Conservation Department (AFCD) of the Government of the Hong Kong Special Administrative Region, China regularly conducts contraceptive operations on the macaque population. Between August and December 2016, paired swab samples from oral, perianal and vaginal (female only) sites of 117 wild macaques (84 females and 33 males) were collected in 2 ml specimen transport medium (STM) (Hank’s BSS (10×), 5% Bovine Albumin (BA), Gentamycin (4 mg/ml), Pen/Strept (50,000 μg/ml), Fungizone (1 mg/ml), and NaHCO_3_ 7.5%) when the animals were anesthetised for the veterinary and/or contraceptive treatment. Basic animal information including gender, estimated age, body weight, body condition and temperature were recorded. After swab collection, animals were vaccinated with parasiticide (Ivomec), antibiotics (Amoxycillin) and a nonsteroidal anti-inflammatory drug (Rimadyl) before release. The programme has been approved by the AFCD and presented to the Animal Welfare Advisory Group (AWAG).

Swab samples were transported to the laboratory in an ice box cooler within 2 hours and stored at −20 °C immediately. For each sample, 200 µl STM were used for DNA extraction using the Qiagen DNA Mini Kit (Qiagen, USA). Purified DNA was eluted in 200 μl elution buffer (pH 8.0) and PCR amplified using primers 515F and 806R, resulting in an approximately 250 bp long amplicon spanning the V4 variable region of the 16S rRNA gene^61^. A pair of dual 12-bp barcodes was introduced to the PCR amplicons from each sample through the forward and reverse primers according to the protocol from the Earth Microbiome Project^62^. Successful amplicons were pooled at approximately equal molar DNA concentrations and sequenced on an Illumina MiSeq (Illumina Inc., USA) at the Weill Cornell Medicine Genomics Resources Core Facility, New York, USA, using paired-end reads. The DNA extraction was processed in a BioSafety Cabinet in a laboratory physically separated from where the PCR amplification was performed to minimize contamination. Negative controls, including STM, DNA extraction, and PCR master mixture were set. All sequence data generated from this survey were deposited in the Sequence Read Archive with accession number PRJNA411767.

### Human Microbiome Project (HMP) 16S rRNA gene sequence dataset

To compare the macaque-associated microbial community compositions with their human counterparts, 16S V3-V5 region 454 pyrosequencing data of paired saliva, stool and vaginal (female only) samples from 290 healthy human subjects (158 females and 132 males) were downloaded from the NIH Human Microbiome Project 16S dataset (PRJNA48489) (https://www.hmpdacc.org/hmp/HMR16S/)^3^.

### Sequence data processing and community composition statistical analyses

Demultiplexed raw sequences were quality-filtered and clustered into operational taxonomic units (OTUs) using an established bioinformatics pipeline which includes QIIME (MacQIIME 1.9.1-20150604)^63^, PPLACER v1.1.alpha17^64^, USEARCH v9.2.64^65^ and in-house developed scripts^66^. Sequences were clustered into OTUs based on a threshold of 97% nucleotide similarity using UPARSE^67^ and then assigned taxonomic identities using RDP classifier^68^ with a bacterial 16S reference database optimized from SILVA (release 128)^69^, HOMD^70^ and NCBI 16S Microbial (ftp://ftp.ncbi.nlm.nih.gov/blast/db/16SMicrobial.tar.gz). The resulting OTU tables were summarized at the phylum (L2) and genus (L6) levels for alpha and beta diversity analyses using QIIME and R v3.4.0^71^. In order to minimize potential sequence bias due to different lengths and depths, we used a closed-reference approach in QIIME to pick up OTUs for macaque and human bacterial reads separately. Two reference databases dereplicate from V3–V5 reads and V4 reads, including all novel OTUs at 97% dissimilarity, were pre-assigned using UPARSE. These two OTU tables were then merged and summarized at species level and/or above. Singleton reads were removed from the OTU tables.

We used a UniFrac algorithm in ‘*GUniFrac*’ R package^72^ to calculate unweighted and weighted (alpha = 0.5) pairwise Kantorovich-Rubinstein (KR) distances between samples. The OTU phylogenetic tree was constructed using PPLACER by placing the 16S sequences on a bacterial 16S reference tree to maximize phylogenetic likelihood inferred from a 16S reference alignment. Differences in community composition were assessed using permutational multivariate analysis of variance (PERMANOVA) in the ‘*vegan*’ R package and Jensen-Shannon divergence^73^. Principal component ordination analysis (PCA) was used to visualise associations between community composition and experimental factors. In addition, we used a linear discriminant analysis (LDA) for effect size (LEfSe) to determine characteristic microbiota between host species and body sites^74^. Higher LDA scores reflect significantly higher abundances of certain bacteria within the groups of samples. Spearman’s rank correlation was used to evaluate co-occurrence of taxa based on relative abundances. Comparisons of characteristics OTUs between sample groups were performed using non-parametric Mann-Whitney Wilcoxon rank-sum test or chi-square test. A two-sided p value of < 0.05 was considered statistically significant.

### Functional prediction based on community composition

Functional profiles of the macaque and human microbial communities were predicted using PICRUSt v1.1.1^75^ based on community composition and represented as KEGG Orthology (KO) counts^76^. These counts were summarized into KO hierarchies and then normalized for library size and variance stabilization-transformed using ‘*DESeq. 2*’ R package^77^. Differences in transformed counts between sample groups were analysed using *STAMP*^78^. Metabolic pathways that discriminated between host species and/or body sites were identified using sparse partial least squares discriminant analysis (sPLSDA) implemented in the ‘*mixOmics*’ R package^79^.

## Electronic supplementary material

Supplementary information

## Data Availability

All 16S sequence data generated from this survey were deposited in the Sequence Read Archive with accession number PRJNA411767. The human microbiota 16S data were downloaded from the NIH Human Microbiome Project (PRJNA48489) (https://www.hmpdacc.org/hmp/HMR16S/).

## References

[1] Grice EA, Segre JA (2012). The human microbiome: our second genome. Annual review of genomics and human genetics.

[2] Lynch SV, Pedersen O (2016). The Human Intestinal Microbiome in Health and Disease. N Engl J Med.

[3] Human Microbiome Project, C. Structure, function and diversity of the healthy human microbiome. *Nature* **486**, 207–214, 10.1038/nature11234 (2012). 10.1038/nature11234 PMC3564958 22699609

[4] Fredricks DN, Fiedler TL, Marrazzo JM (2005). Molecular identification of bacteria associated with bacterial vaginosis. N Engl J Med.

[5] Garrett WS (2015). Cancer and the microbiota. Science.

[6] Qin J (2012). A metagenome-wide association study of gut microbiota in type 2 diabetes. Nature.

[7] Pickard JM, Zeng MY, Caruso R, Nunez G (2017). Gut microbiota: Role in pathogen colonization, immune responses, and inflammatory disease. Immunol Rev.

[8] Ridaura VK (2013). Gut microbiota from twins discordant for obesity modulate metabolism in mice. Science.

[9] Allegretti J (2017). The Current Landscape and Lessons from Fecal Microbiota Transplantation for Inflammatory Bowel Disease: Past, Present, and Future. Inflamm Bowel Dis.

[10] Foster KR, Schluter J, Coyte KZ, Rakoff-Nahoum S (2017). The evolution of the host microbiome as an ecosystem on a leash. Nature.

[11] Gonzalez A (2011). Our microbial selves: what ecology can teach us. EMBO Rep.

[12] Carlsson HE, Schapiro SJ, Farah I, Hau J (2004). Use of primates in research: a global overview. Am J Primatol.

[13] Kostic AD, Howitt MR, Garrett WS (2013). Exploring host-microbiota interactions in animal models and humans. Genes Dev.

[14] Ochman H (2010). Evolutionary relationships of wild hominids recapitulated by gut microbial communities. PLoS Biol.

[15] Yildirim S (2010). Characterization of the fecal microbiome from non-human wild primates reveals species specific microbial communities. PLoS ONE.

[16] Moeller AH (2016). Cospeciation of gut microbiota with hominids. Science.

[17] Council Sarah E., Savage Amy M., Urban Julie M., Ehlers Megan E., Skene J. H. Pate, Platt Michael L., Dunn Robert R., Horvath Julie E. (2016). Diversity and evolution of the primate skin microbiome. Proceedings of the Royal Society B: Biological Sciences.

[18] McCord AI (2014). Fecal microbiomes of non-human primates in Western Uganda reveal species-specific communities largely resistant to habitat perturbation. Am J Primatol.

[19] Shin NR, Whon TW, Bae JW (2015). Proteobacteria: microbial signature of dysbiosis in gut microbiota. Trends in biotechnology.

[20] Tung J (2015). Social networks predict gut microbiome composition in wild baboons. Elife.

[21] Yasuda K (2015). Biogeography of the intestinal mucosal and lumenal microbiome in the rhesus macaque. Cell host & microbe.

[22] Yildirim S (2014). Primate vaginal microbiomes exhibit species specificity without universal Lactobacillus dominance. Isme J.

[23] Spear GT (2010). Identification of rhesus macaque genital microbiota by 16S pyrosequencing shows similarities to human bacterial vaginosis: implications for use as an animal model for HIV vaginal infection. AIDS Res Hum Retroviruses.

[24] Miller EA, Beasley DE, Dunn RR, Archie EA (2016). Lactobacilli Dominance and Vaginal pH: Why Is the Human Vaginal Microbiome Unique?. Front Microbiol.

[25] Clayton JB (2016). Captivity humanizes the primate microbiome. Proc Natl Acad Sci USA.

[26] Gevers D (2012). The Human Microbiome Project: a community resource for the healthy human microbiome. PLoS Biol.

[27] Amato KR (2015). Variable responses of human and non-human primate gut microbiomes to a Western diet. Microbiome.

[28] Angelakis E (2016). Gut microbiome and dietary patterns in different Saudi populations and monkeys. Scientific reports.

[29] Muegge BD (2011). Diet drives convergence in gut microbiome functions across mammalian phylogeny and within humans. Science.

[30] Wu GD (2011). Linking long-term dietary patterns with gut microbial enterotypes. Science.

[31] Richard AF, Goldstein SJ, Dewar RE (1989). Weed macaques: The evolutionary implications of macaque feeding ecology. International Journal of Primatology.

[32] Kienesberger S (2012). Serologic host response to Helicobacter pylori and Campylobacter jejuni in socially housed Rhesus macaques (Macaca mulatta). Gut pathogens.

[33] McKenna P (2008). The macaque gut microbiome in health, lentiviral infection, and chronic enterocolitis. PLoS Pathog.

[34] Kumagai T (1998). Acquisition versus loss of Helicobacter pylori infection in Japan: results from an 8-year birth cohort study. J Infect Dis.

[35] Blaser, M. J. *Missing microbes: how the overuse of antibiotics is fueling our modern plagues*. First edition. edn, (Henry Holt and Company, 2014). 10.1096/fj.14-0901ufm PMC4139909 29405744

[36] Polk DB, Peek RM (2010). Helicobacter pylori: gastric cancer and beyond. Nat Rev Cancer.

[37] Chen Y, Blaser MJ (2008). Helicobacter pylori colonization is inversely associated with childhood asthma. J Infect Dis.

[38] Rothenbacher D, Blaser MJ, Bode G, Brenner H (2000). Inverse relationship between gastric colonization of Helicobacter pylori and diarrheal illnesses in children: results of a population-based cross-sectional study. J Infect Dis.

[39] Fox JG (2001). Novel Helicobacter species isolated from rhesus monkeys with chronic idiopathic colitis. J Med Microbiol.

[40] Schauer, D. B. In *Helicobacter pylori: Physiology and Genetics* (eds Mobley, H. L. T., Mendz, G. L. & Hazell, S. L. (2001). 21290711

[41] Pierantozzi M (2006). Helicobacter pylori eradication and l-dopa absorption in patients with PD and motor fluctuations. Neurology.

[42] Stumpf RM (2013). The primate vaginal microbiome: comparative context and implications for human health and disease. Am J Phys Anthropol.

[43] Mirmonsef P (2014). Free glycogen in vaginal fluids is associated with Lactobacillus colonization and low vaginal pH. PLoS ONE.

[44] Ravel J (2011). Vaginal microbiome of reproductive-age women. Proceedings of the National Academy of Sciences of the United States of America.

[45] Mirmonsef P (2012). A comparison of lower genital tract glycogen and lactic acid levels in women and macaques: implications for HIV and SIV susceptibility. AIDS Res Hum Retroviruses.

[46] Bonsall RW, Michael RP (1980). The externalization of vaginal fatty acids by the female rhesus monkey. Journal of Chemical Ecology.

[47] Henkel S, Lambides AR, Berger A, Thomsen R, Widdig A (2015). Rhesus macaques (Macaca mulatta) recognize group membership via olfactory cues alone. Behavioral ecology and sociobiology.

[48] Freitas AC (2017). The vaginal microbiome of pregnant women is less rich and diverse, with lower prevalence of Mollicutes, compared to non-pregnant women. Scientific reports.

[49] Aagaard K (2012). A metagenomic approach to characterization of the vaginal microbiome signature in pregnancy. PLoS ONE.

[50] Ling Z (2010). Molecular analysis of the diversity of vaginal microbiota associated with bacterial vaginosis. BMC Genomics.

[51] Srinivasan S (2012). Bacterial communities in women with bacterial vaginosis: high resolution phylogenetic analyses reveal relationships of microbiota to clinical criteria. PLoS ONE.

[52] Pybus V, Onderdonk AB (1997). Evidence for a commensal, symbiotic relationship between Gardnerella vaginalis and Prevotella bivia involving ammonia: potential significance for bacterial vaginosis. J Infect Dis.

[53] Pybus V, Onderdonk AB (1998). A commensal symbiosis between Prevotella bivia and Peptostreptococcus anaerobius involves amino acids: potential significance to the pathogenesis of bacterial vaginosis. FEMS immunology and medical microbiology.

[54] Gholizadeh P (2016). Role of oral microbiome on oral cancers, a review. Biomed Pharmacother.

[55] Ma B, Forney LJ, Ravel J (2012). Vaginal microbiome: rethinking health and disease. Annu Rev Microbiol.

[56] Gibbs RA (2007). Evolutionary and biomedical insights from the rhesus macaque genome. Science.

[57] Abusleme L (2013). The subgingival microbiome in health and periodontitis and its relationship with community biomass and inflammation. Isme J.

[58] Liu B (2012). Deep sequencing of the oral microbiome reveals signatures of periodontal disease. PLoS ONE.

[59] Mirzaei MK, Maurice CF (2017). Menage a trois in the human gut: interactions between host, bacteria and phages. Nat Rev Micro.

[60] Shedlock DJ, Silvestri G, Weiner DB (2009). Monkeying around with HIV vaccines: using rhesus macaques to define ‘gatekeepers’ for clinical trials. Nat Rev Immunol.

[61] Wang Y, Qian PY (2009). Conservative fragments in bacterial 16S rRNA genes and primer design for 16S ribosomal DNA amplicons in metagenomic studies. PLoS ONE.

[62] Gilbert JA, Jansson JK, Knight R (2014). The Earth Microbiome project: successes and aspirations. BMC Biol.

[63] Caporaso JG (2010). QIIME allows analysis of high-throughput community sequencing data. Nat Methods.

[64] Matsen FA, Kodner RB, Armbrust E (2010). V. pplacer: linear time maximum-likelihood and Bayesian phylogenetic placement of sequences onto a fixed reference tree. BMC Bioinformatics.

[65] Edgar RC (2010). Search and clustering orders of magnitude faster than BLAST. Bioinformatics.

[66] Smith BC (2012). The cervical microbiome over 7 years and a comparison of methodologies for its characterization. PLoS ONE.

[67] Edgar RC (2013). UPARSE: highly accurate OTU sequences from microbial amplicon reads. Nat Methods.

[68] Vilo C, Dong Q (2012). Evaluation of the RDP Classifier Accuracy using 16S rRNA Gene Variable Regions. Metagenomics.

[69] Quast C (2013). The SILVA ribosomal RNA gene database project: improved data processing and web-based tools. Nucleic Acids Res.

[70] Chen T (2010). The Human Oral Microbiome Database: a web accessible resource for investigating oral microbe taxonomic and genomic information. Database (Oxford).

[71] Team, R. C. (ISBN 3-900051-07-0, 2014).

[72] Hamady M, Lozupone C, Knight R (2010). Fast UniFrac: facilitating high-throughput phylogenetic analyses of microbial communities including analysis of pyrosequencing and PhyloChip data. Isme J.

[73] Lozupone CA, Knight R (2008). Species divergence and the measurement of microbial diversity. FEMS Microbiol Rev.

[74] Segata N (2011). Metagenomic biomarker discovery and explanation. Genome Biol.

[75] Langille MG (2013). Predictive functional profiling of microbial communities using 16S rRNA marker gene sequences. Nature biotechnology.

[76] Kanehisa M, Furumichi M, Tanabe M, Sato Y, Morishima K (2017). KEGG: new perspectives on genomes, pathways, diseases and drugs. Nucleic Acids Res.

[77] Love MI, Huber W, Anders S (2014). Moderated estimation of fold change and dispersion for RNA-seq data with DESeq2. Genome Biol.

[78] Parks DH, Tyson GW, Hugenholtz P, Beiko RG (2014). STAMP: statistical analysis of taxonomic and functional profiles. Bioinformatics.

[79] Rohart F, Eslami A, Matigian N, Bougeard S, Le Cao KA (2017). MINT: a multivariate integrative method to identify reproducible molecular signatures across independent experiments and platforms. BMC Bioinformatics.

