# Supplementary material for: Diversity of macaque microbiota compared to the human counterparts
Source: Sci Rep. 2018 Oct 22;8:15573. doi: 10.1038/s41598-018-33950-6 (PMC6197227; doi:10.1038/s41598-018-33950-6)
Supplement: Supplementary file 1 — Supplementary information [file 41598_2018_33950_MOESM1_ESM.pdf]

Diversity of macaque microbiota compared to the human counterparts

Zigui Chen [1,2], Yun Kit Yeoh [1,2], Mamie Hui [1,2], Po Yee Wong [1], Martin C.W. Chan [1], Margaret Ip [1,2], Jun Yu [2,3,4], Robert D. Burk [5], Francis L.K. Chan [2,3] & Paul K.S. Chan [1,2 *]

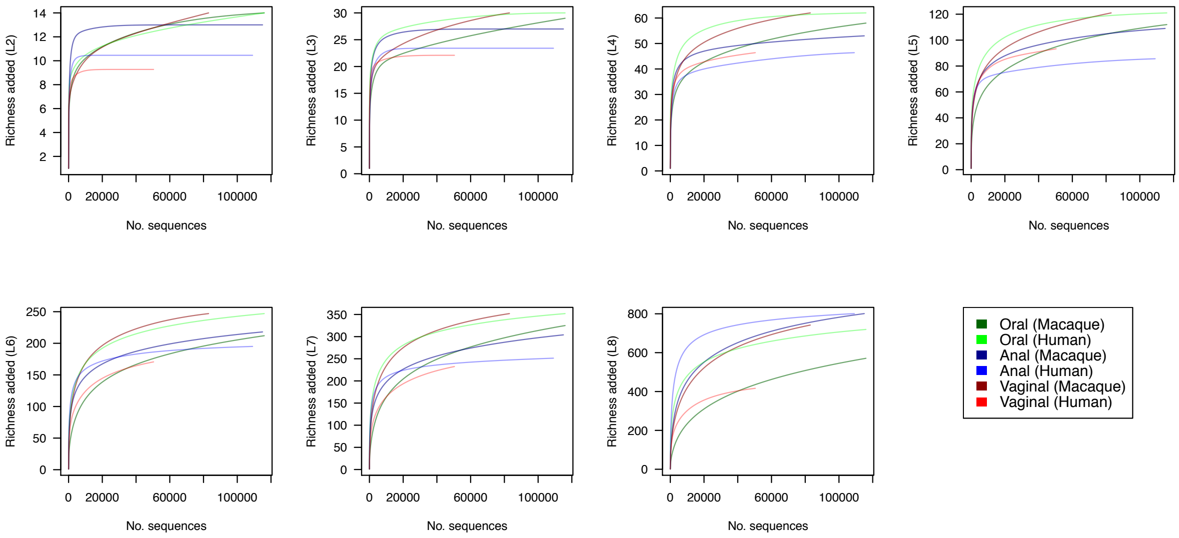

Figure S1. Rarefaction curves of species richness. Rarefaction was performed for OTUs summarized at various taxonomic ranks: L2, phylum, L3, order, L4, class, L5, family, L6, genus, L7, species and L8, OTU. The 16S rRNA sequences were rarefied to 1,000 reads per samples.

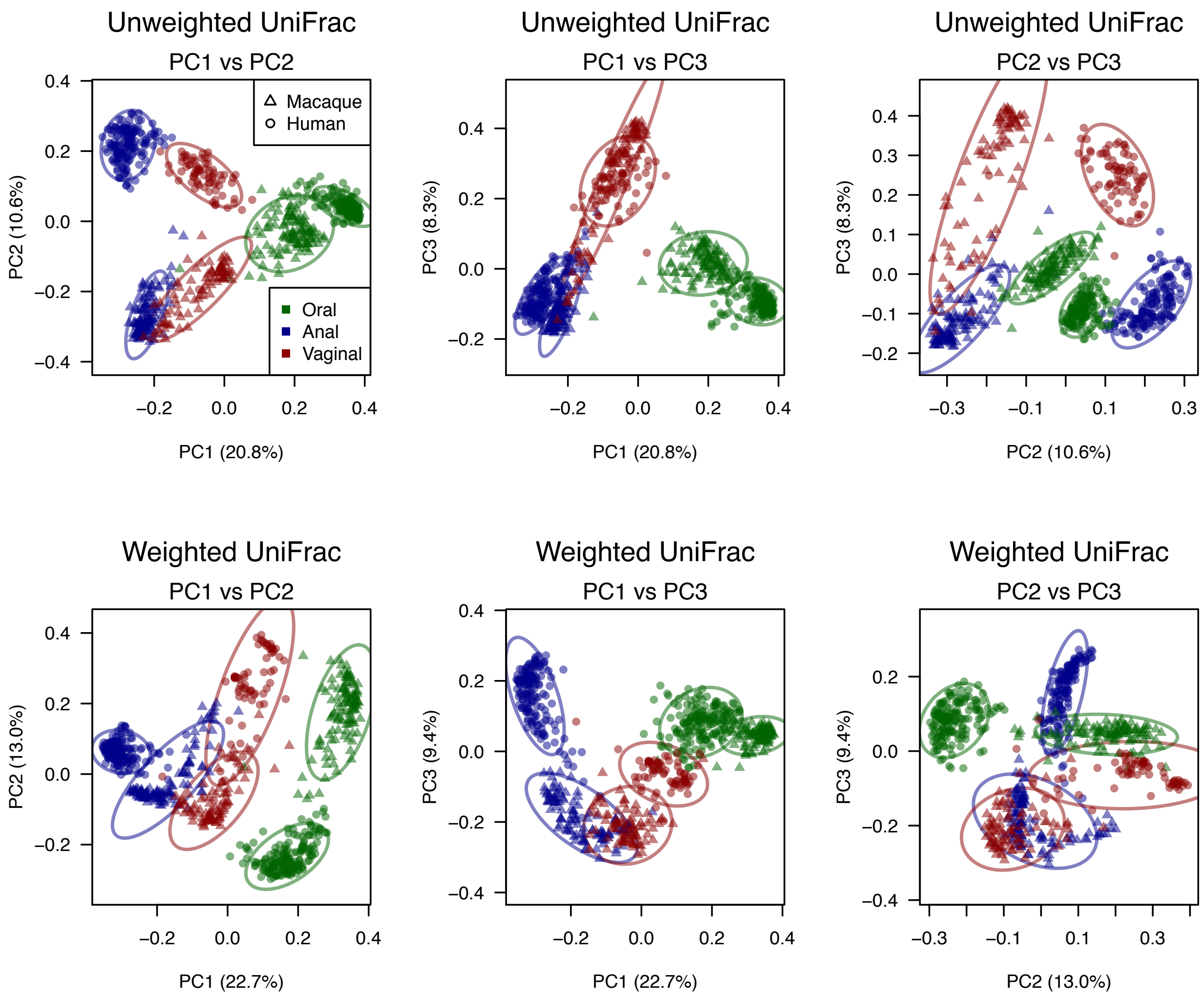

Figure S2. Diversity of primate microbiome based on unweighted and weighted UniFrac distances.

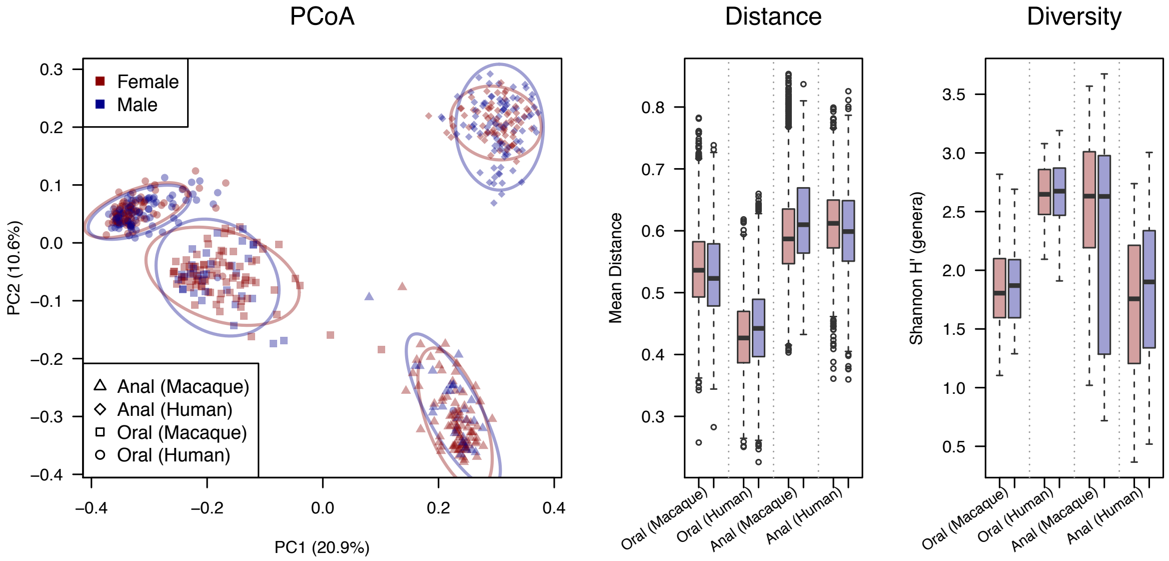

Figure S3. Comparison of primate microbiome diversity by gender based on unweighted UniFrac distances.

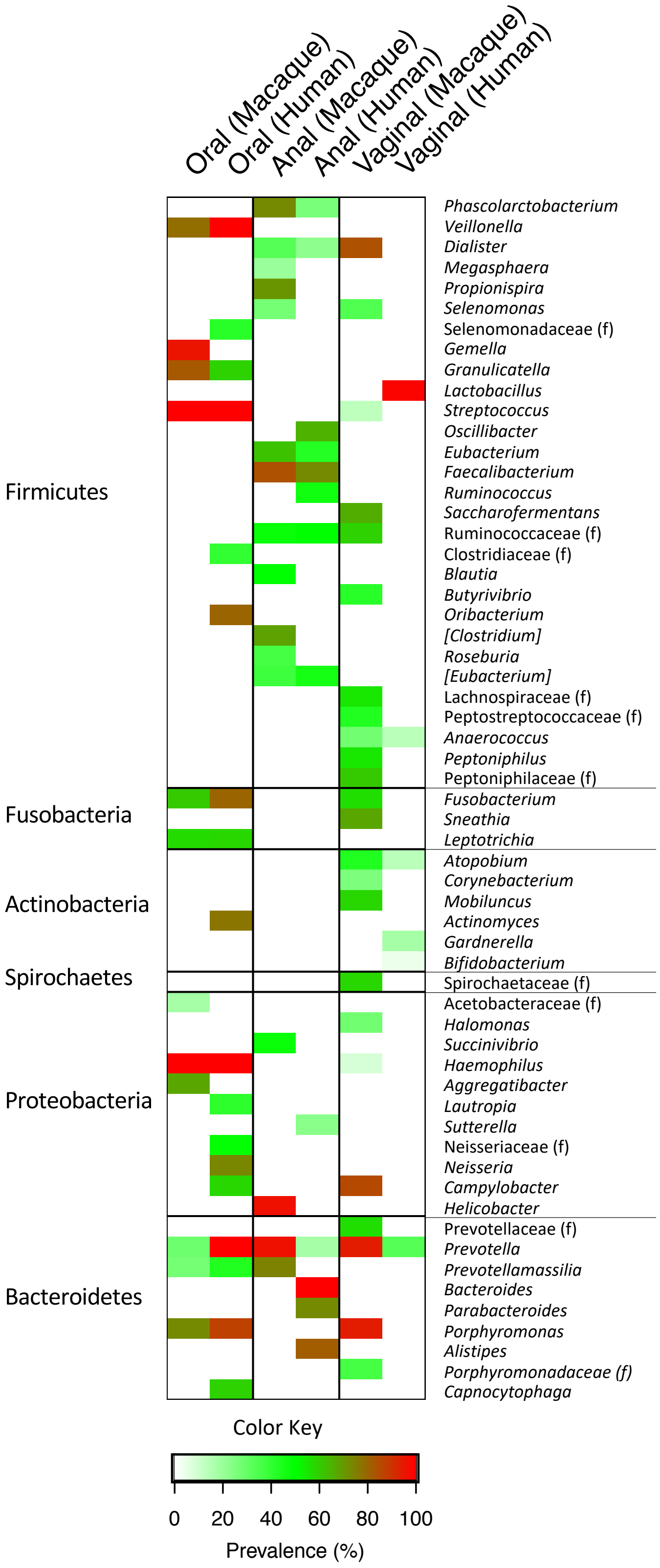

Figure S4. A heat map showing the prevalence of "core" dominant bacterial taxa in each body site of macaques and humans.

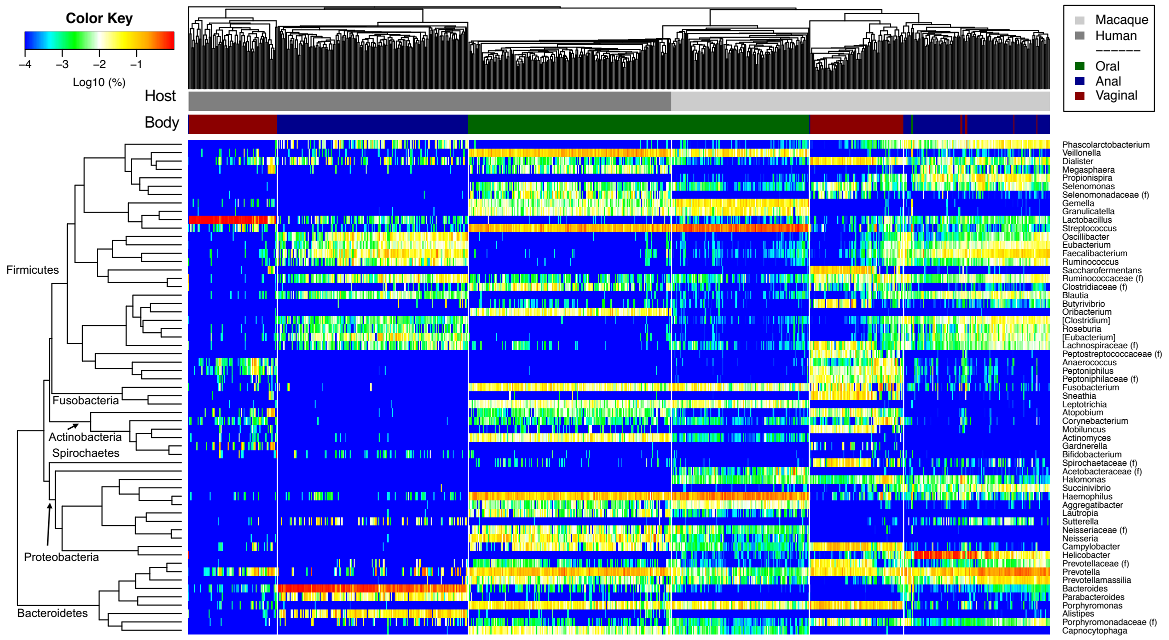

Figure S5. Heat map showing the proportional abundances of "core" bacterial taxa in the respective body sites of the surveyed macaques and humans. Each column represents individuals with the proportional abundance on a base 10 logarithmic scale, with 0 (a log10 of 100%) in red and -4 (a log10 of 0.01%) in blue. Each raw represents the "core" bacteria, with the phylogeny on left based on the complete 16S rRNA gene sequences. The clustering of individuals on the top of were based on the unweighted GUniFrac distance.

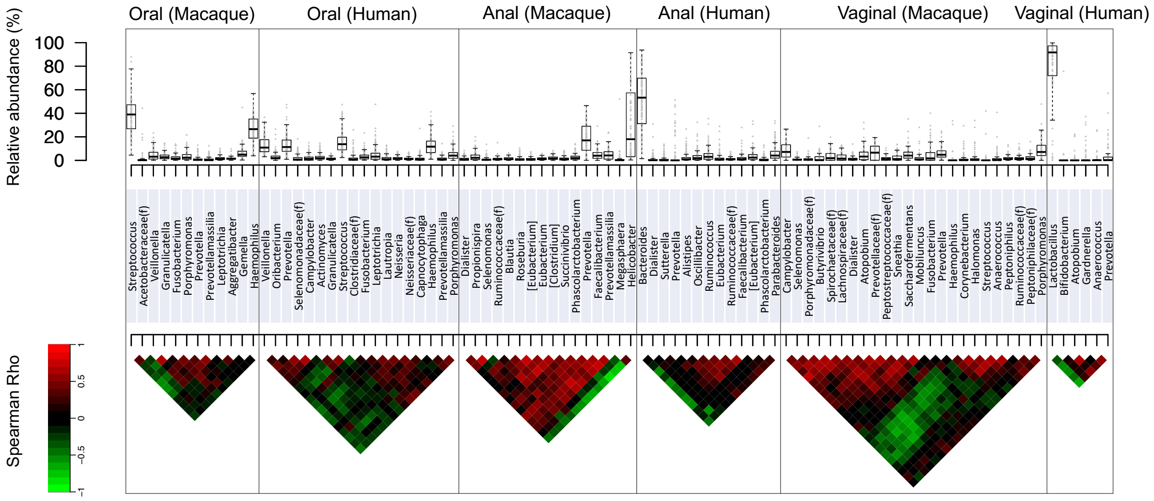

Figure S6. Co-occurrence among the "core" bacterial taxa within the respective body sites (taxa with mean abundance of >1%), as determined by Spearman's rank correlation analysis. The taxa were clustered using UPGMA (Unweighted Pair Group Method with Arithmetic Mean) hierarchical clustering based on correlation distance matrices produced using Spearman's rank correlation.

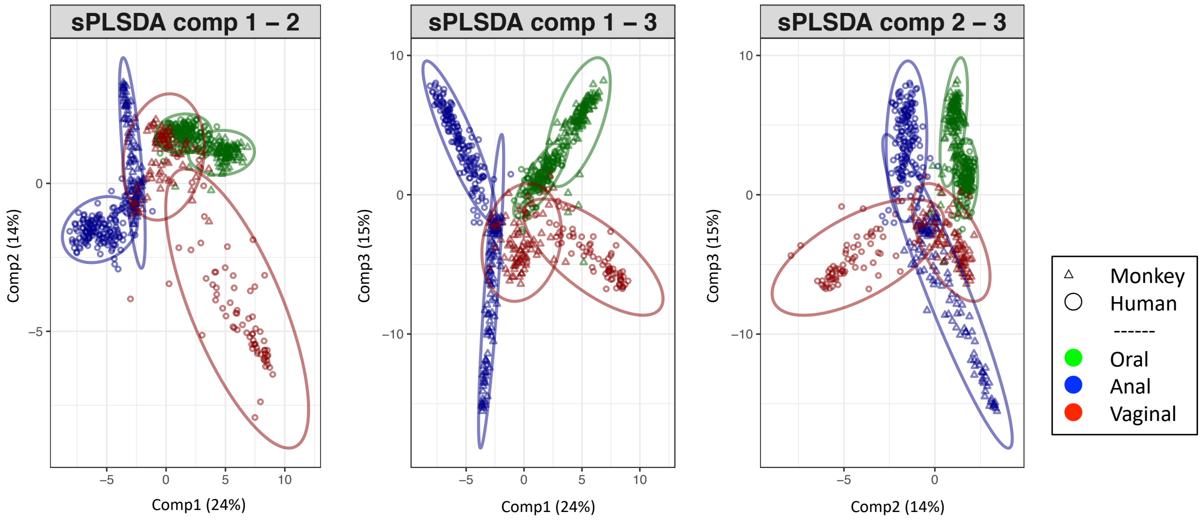

Figure S7. Principal component ordination of variance stabilization-transformed KEGG metabolic pathway abundances predicted by PICRUSt. The clustering was supported by a sparse partial least squares discriminant analysis (sPLSDA) using the first three components.

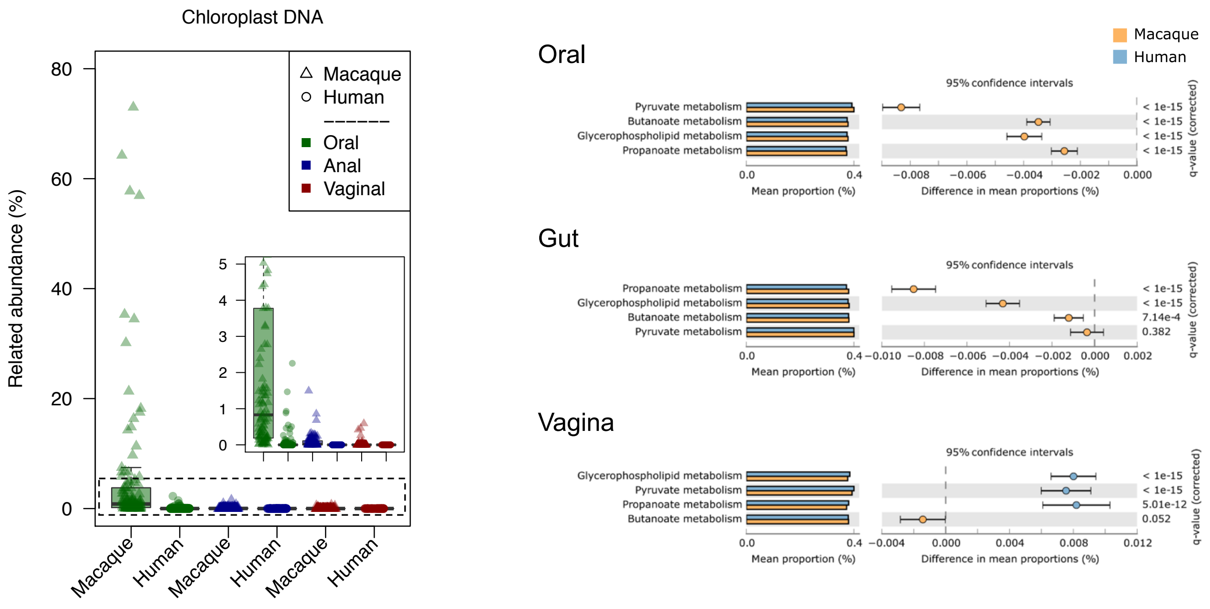

Figure S8. Relative abundances of chloroplast sequences detected in the 16S sequences in each body site of macaques and humans. Higher relative abundances of chloroplast sequences in the macaques were consistent with increased metabolism associated with plant fiber degradation (e.g., butanoate, glycerophospholipid, propanoate and pyruvate) in macaques relative to humans**.** Pathways in each panel are ordered from top to bottom according to increasing adjusted p values, listed on the right of each bar. Pathways associated with human samples are coloured blue, macaques are coloured orange. The reported p values on the right are Bonferroni-corrected for multiple comparisons.

Table S1. Macaque individual collected in this study.

Subject_ID	Sample_ID	Gender	Body_site	16S_region	Paried 1 file name	Paried 2 file name	16S V4 read (raw)	16S V4 read (QC)
PM001	PM001_S1	M	Oral	16SV4	PM001_S1_L001_R1.fastq	PM001_S1_L001_R2.fastq	65427	57109
PM001	PM001_S2	M	Anal	16SV4	PM001_S2_L001_R1.fastq	PM001_S2_L001_R2.fastq	**55432**	**42761**
PM002	PM002_S1	F	Oral	16SV4	PM002_S1_L001_R1.fastq	PM002_S1_L001_R2.fastq	65933	59359
PM002	PM002_S2	F	Anal	16SV4	PM002_S2_L001_R1.fastq	PM002_S2_L001_R2.fastq	66260	53471
PM002	PM002_S3	F	Vaginal	16SV4	PM002_S3_L001_R1.fastq	PM002_S3_L001_R2.fastq	70508	52821
PM003	PM003_S1	F	Oral	16SV4	PM003_S1_L001_R1.fastq	PM003_S1_L001_R2.fastq	18444	16617
PM003	PM003_S2	F	Anal	16SV4	PM003_S2_L001_R1.fastq	PM003_S2_L001_R2.fastq	49797	38173
PM003	PM003_S3	F	Vaginal	16SV4	PM003_S3_L001_R1.fastq	PM003_S3_L001_R2.fastq	19897	18264
PM004	PM004_S1	F	Oral	16SV4	PM004_S1_L001_R1.fastq	PM004_S1_L001_R2.fastq	69280	60119
PM004	PM004_S2	F	Anal	16SV4	PM004_S2_L001_R1.fastq	PM004_S2_L001_R2.fastq	72257	57996
PM004	PM004_S3	F	Vaginal	16SV4	PM004_S3_L001_R1.fastq	PM004_S3_L001_R2.fastq	54873	42651
PM005	PM005_S1	M	Oral	16SV4	PM005_S1_L001_R1.fastq	PM005_S1_L001_R2.fastq	61835	55917
PM005	PM005_S2	M	Anal	16SV4	PM005_S2_L001_R1.fastq	PM005_S2_L001_R2.fastq	75017	66722
PM006	PM006_S1	F	Oral	16SV4	PM006_S1_L001_R1.fastq	PM006_S1_L001_R2.fastq	66852	59058
PM006	PM006_S2	F	Anal	16SV4	PM006_S2_L001_R1.fastq	PM006_S2_L001_R2.fastq	63149	54206
PM006	PM006_S3	F	Vaginal	16SV4	PM006_S3_L001_R1.fastq	PM006_S3_L001_R2.fastq	42541	37496
PM007	PM007_S1	M	Oral	16SV4	PM007_S1_L001_R1.fastq	PM007_S1_L001_R2.fastq	54089	47364
PM007	PM007_S2	M	Anal	16SV4	PM007_S2_L001_R1.fastq	PM007_S2_L001_R2.fastq	53325	48522
PM008	PM008_S1	F	Oral	16SV4	PM008_S1_L001_R1.fastq	PM008_S1_L001_R2.fastq	62849	55887
PM008	PM008_S2	F	Anal	16SV4	PM008_S2_L001_R1.fastq	PM008_S2_L001_R2.fastq	56009	33784
PM008	PM008_S3	F	Vaginal	16SV4	PM008_S3_L001_R1.fastq	PM008_S3_L001_R2.fastq	49138	41320
PM009	PM009_S1	F	Oral	16SV4	PM009_S1_L001_R1.fastq	PM009_S1_L001_R2.fastq	25677	19754
PM009	PM009_S2	F	Anal	16SV4	PM009_S2_L001_R1.fastq	PM009_S2_L001_R2.fastq	38804	33468
PM009	PM009_S3	F	Vaginal	16SV4	PM009_S3_L001_R1.fastq	PM009_S3_L001_R2.fastq	11374	5325
PM010	PM010_S1	F	Oral	16SV4	PM010_S1_L001_R1.fastq	PM010_S1_L001_R2.fastq	60061	51686
PM010	PM010_S2	F	Anal	16SV4	PM010_S2_L001_R1.fastq	PM010_S2_L001_R2.fastq	13246	11381
PM010	PM010_S3	F	Vaginal	16SV4	PM010_S3_L001_R1.fastq	PM010_S3_L001_R2.fastq	23026	19714
PM011	PM011_S1	M	Oral	16SV4	PM011_S1_L001_R1.fastq	PM011_S1_L001_R2.fastq	31873	28244
PM011	PM011_S2	M	Anal	16SV4	PM011_S2_L001_R1.fastq	PM011_S2_L001_R2.fastq	57304	50296
PM012	PM012_S1	F	Oral	16SV4	PM012_S1_L001_R1.fastq	PM012_S1_L001_R2.fastq	67714	59856
PM012	PM012_S2	F	Anal	16SV4	PM012_S2_L001_R1.fastq	PM012_S2_L001_R2.fastq	66938	53396
PM012	PM012_S3	F	Vaginal	16SV4	PM012_S3_L001_R1.fastq	PM012_S3_L001_R2.fastq	59117	48656
PM013	PM013_S1	M	Oral	16SV4	PM013_S1_L001_R1.fastq	PM013_S1_L001_R2.fastq	67641	60133
PM013	PM013_S2	M	Anal	16SV4	PM013_S2_L001_R1.fastq	PM013_S2_L001_R2.fastq	76306	59818
PM014	PM014_S1	F	Oral	16SV4	PM014_S1_L001_R1.fastq	PM014_S1_L001_R2.fastq	68655	60502
PM014	PM014_S2	F	Anal	16SV4	PM014_S2_L001_R1.fastq	PM014_S2_L001_R2.fastq	52500	46854
PM014	PM014_S3	F	Vaginal	16SV4	PM014_S3_L001_R1.fastq	PM014_S3_L001_R2.fastq	30743	27740
PM015	PM015_S1	F	Oral	16SV4	PM015_S1_L001_R1.fastq	PM015_S1_L001_R2.fastq	62245	55105
PM015	PM015_S2	F	Anal	16SV4	PM015_S2_L001_R1.fastq	PM015_S2_L001_R2.fastq	70864	64026
PM015	PM015_S3	F	Vaginal	16SV4	PM015_S3_L001_R1.fastq	PM015_S3_L001_R2.fastq	63157	47544
PM016	PM016_S1	F	Oral	16SV4	PM016_S1_L001_R1.fastq	PM016_S1_L001_R2.fastq	12417	8672
PM016	PM016_S2	F	Anal	16SV4	PM016_S2_L001_R1.fastq	PM016_S2_L001_R2.fastq	13598	6690
PM016	PM016_S3	F	Vaginal	16SV4	PM016_S3_L001_R1.fastq	PM016_S3_L001_R2.fastq	8408	4646
PM017	PM017_S1	F	Oral	16SV4	PM017_S1_L001_R1.fastq	PM017_S1_L001_R2.fastq	59992	52411
PM017	PM017_S2	F	Anal	16SV4	PM017_S2_L001_R1.fastq	PM017_S2_L001_R2.fastq	68224	60029
PM017	PM017_S3	F	Vaginal	16SV4	PM017_S3_L001_R1.fastq	PM017_S3_L001_R2.fastq	59171	46445
PM018	PM018_S1	M	Oral	16SV4	PM018_S1_L001_R1.fastq	PM018_S1_L001_R2.fastq	40728	33580
PM018	PM018_S2	M	Anal	16SV4	PM018_S2_L001_R1.fastq	PM018_S2_L001_R2.fastq	42982	31967
PM019	PM019_S1	M	Oral	16SV4	PM019_S1_L001_R1.fastq	PM019_S1_L001_R2.fastq	36435	31995
PM019	PM019_S2	M	Anal	16SV4	PM019_S2_L001_R1.fastq	PM019_S2_L001_R2.fastq	65837	54120
PM020	PM020_S1	M	Oral	16SV4	PM020_S1_L001_R1.fastq	PM020_S1_L001_R2.fastq	65223	57732
PM020	PM020_S2	M	Anal	16SV4	PM020_S2_L001_R1.fastq	PM020_S2_L001_R2.fastq	64685	59345
PM021	PM021_S1	M	Oral	16SV4	PM021_S1_L001_R1.fastq	PM021_S1_L001_R2.fastq	57795	51543
PM021	PM021_S2	M	Anal	16SV4	PM021_S2_L001_R1.fastq	PM021_S2_L001_R2.fastq	53310	48904
PM022	PM022_S1	F	Oral	16SV4	PM022_S1_L001_R1.fastq	PM022_S1_L001_R2.fastq	50717	43524
PM022	PM022_S2	F	Anal	16SV4	PM022_S2_L001_R1.fastq	PM022_S2_L001_R2.fastq	20488	16886
PM022	PM022_S3	F	Vaginal	16SV4	PM022_S3_L001_R1.fastq	PM022_S3_L001_R2.fastq	23689	20584
PM023	PM023_S1	M	Oral	16SV4	PM023_S1_L001_R1.fastq	PM023_S1_L001_R2.fastq	70102	60461
PM023	PM023_S2	M	Anal	16SV4	PM023_S2_L001_R1.fastq	PM023_S2_L001_R2.fastq	56124	44124
PM024	PM024_S1	F	Oral	16SV4	PM024_S1_L001_R1.fastq	PM024_S1_L001_R2.fastq	61296	53779
PM024	PM024_S2	F	Anal	16SV4	PM024_S2_L001_R1.fastq	PM024_S2_L001_R2.fastq	57742	51287
PM024	PM024_S3	F	Vaginal	16SV4	PM024_S3_L001_R1.fastq	PM024_S3_L001_R2.fastq	59114	45641
PM025	PM025_S1	F	Oral	16SV4	PM025_S1_L001_R1.fastq	PM025_S1_L001_R2.fastq	58911	52541
PM025	PM025_S2	F	Anal	16SV4	PM025_S2_L001_R1.fastq	PM025_S2_L001_R2.fastq	50102	45585
PM025	PM025_S3	F	Vaginal	16SV4	PM025_S3_L001_R1.fastq	PM025_S3_L001_R2.fastq	57323	41886
PM026	PM026_S1	F	Oral	16SV4	PM026_S1_L001_R1.fastq	PM026_S1_L001_R2.fastq	62501	55643

PM026	PM026_S2	F	Anal	16SV4	PM026_S2_L001_R1.fastq	PM026_S2_L001_R2.fastq	60311	56017
PM026	PM026_S3	F	Vaginal	16SV4	PM026_S3_L001_R1.fastq	PM026_S3_L001_R2.fastq	69240	52618
PM027	PM027_S1	F	Oral	16SV4	PM027_S1_L001_R1.fastq	PM027_S1_L001_R2.fastq	63047	56773
PM027	PM027_S2	F	Anal	16SV4	PM027_S2_L001_R1.fastq	PM027_S2_L001_R2.fastq	61963	56254
PM027	PM027_S3	F	Vaginal	16SV4	PM027_S3_L001_R1.fastq	PM027_S3_L001_R2.fastq	13644	12107
PM028	PM028_S1	M	Oral	16SV4	PM028_S1_L001_R1.fastq	PM028_S1_L001_R2.fastq	59933	53541
PM028	PM028_S2	M	Anal	16SV4	PM028_S2_L001_R1.fastq	PM028_S2_L001_R2.fastq	63356	49688
PM029	PM029_S1	M	Oral	16SV4	PM029_S1_L001_R1.fastq	PM029_S1_L001_R2.fastq	10410	6668
PM029	PM029_S2	M	Anal	16SV4	PM029_S2_L001_R1.fastq	PM029_S2_L001_R2.fastq	6591	1301
PM030	PM030_S1	F	Oral	16SV4	PM030_S1_L001_R1.fastq	PM030_S1_L001_R2.fastq	67621	60182
PM030	PM030_S2	F	Anal	16SV4	PM030_S2_L001_R1.fastq	PM030_S2_L001_R2.fastq	70813	64687
PM030	PM030_S3	F	Vaginal	16SV4	PM030_S3_L001_R1.fastq	PM030_S3_L001_R2.fastq	63707	49360
PM031	PM031_S1	F	Oral	16SV4	PM031_S1_L001_R1.fastq	PM031_S1_L001_R2.fastq	58908	52510
PM031	PM031_S2	F	Anal	16SV4	PM031_S2_L001_R1.fastq	PM031_S2_L001_R2.fastq	48877	42308
PM031	PM031_S3	F	Vaginal	16SV4	PM031_S3_L001_R1.fastq	PM031_S3_L001_R2.fastq	53090	44586
PM032	PM032_S1	M	Oral	16SV4	PM032_S1_L001_R1.fastq	PM032_S1_L001_R2.fastq	62067	55645
PM032	PM032_S2	M	Anal	16SV4	PM032_S2_L001_R1.fastq	PM032_S2_L001_R2.fastq	67719	53895
PM033	PM033_S1	F	Oral	16SV4	PM033_S1_L001_R1.fastq	PM033_S1_L001_R2.fastq	69638	61800
PM033	PM033_S2	F	Anal	16SV4	PM033_S2_L001_R1.fastq	PM033_S2_L001_R2.fastq	57629	51427
PM033	PM033_S3	F	Vaginal	16SV4	PM033_S3_L001_R1.fastq	PM033_S3_L001_R2.fastq	49459	31166
PM034	PM034_S1	M	Oral	16SV4	PM034_S1_L001_R1.fastq	PM034_S1_L001_R2.fastq	68126	61032
PM034	PM034_S2	M	Anal	16SV4	PM034_S2_L001_R1.fastq	PM034_S2_L001_R2.fastq	68376	62479
PM035	PM035_S1	M	Oral	16SV4	PM035_S1_L001_R1.fastq	PM035_S1_L001_R2.fastq	61983	54795
PM035	PM035_S2	M	Anal	16SV4	PM035_S2_L001_R1.fastq	PM035_S2_L001_R2.fastq	47418	43397
PM036	PM036_S1	M	Oral	16SV4	PM036_S1_L001_R1.fastq	PM036_S1_L001_R2.fastq	64184	57856
PM036	PM036_S2	M	Anal	16SV4	PM036_S2_L001_R1.fastq	PM036_S2_L001_R2.fastq	62428	57221
PM037	PM037_S1	M	Oral	16SV4	PM037_S1_L001_R1.fastq	PM037_S1_L001_R2.fastq	78364	66060
PM037	PM037_S2	M	Anal	16SV4	PM037_S2_L001_R1.fastq	PM037_S2_L001_R2.fastq	61692	52056
PM038	PM038_S1	M	Oral	16SV4	PM038_S1_L001_R1.fastq	PM038_S1_L001_R2.fastq	63244	55502
PM038	PM038_S2	M	Anal	16SV4	PM038_S2_L001_R1.fastq	PM038_S2_L001_R2.fastq	65732	57930
PM039	PM039_S1	F	Oral	16SV4	PM039_S1_L001_R1.fastq	PM039_S1_L001_R2.fastq	58065	52012
PM039	PM039_S2	F	Anal	16SV4	PM039_S2_L001_R1.fastq	PM039_S2_L001_R2.fastq	51808	48088
PM039	PM039_S3	F	Vaginal	16SV4	PM039_S3_L001_R1.fastq	PM039_S3_L001_R2.fastq	46481	37863
PM040	PM040_S1	M	Oral	16SV4	PM040_S1_L001_R1.fastq	PM040_S1_L001_R2.fastq	69753	62529
PM040	PM040_S2	M	Anal	16SV4	PM040_S2_L001_R1.fastq	PM040_S2_L001_R2.fastq	67998	55728
PM041	PM041_S1	F	Oral	16SV4	PM041_S1_L001_R1.fastq	PM041_S1_L001_R2.fastq	72270	64240
PM041	PM041_S2	F	Anal	16SV4	PM041_S2_L001_R1.fastq	PM041_S2_L001_R2.fastq	76370	61565
PM041	PM041_S3	F	Vaginal	16SV4	PM041_S3_L001_R1.fastq	PM041_S3_L001_R2.fastq	75335	46048
PM042	PM042_S1	F	Oral	16SV4	PM042_S1_L001_R1.fastq	PM042_S1_L001_R2.fastq	64111	57230
PM042	PM042_S2	F	Anal	16SV4	PM042_S2_L001_R1.fastq	PM042_S2_L001_R2.fastq	66153	57395
PM042	PM042_S3	F	Vaginal	16SV4	PM042_S3_L001_R1.fastq	PM042_S3_L001_R2.fastq	66992	53781
PM043	PM043_S1	F	Oral	16SV4	PM043_S1_L001_R1.fastq	PM043_S1_L001_R2.fastq	56971	50188
PM043	PM043_S2	F	Anal	16SV4	PM043_S2_L001_R1.fastq	PM043_S2_L001_R2.fastq	63303	58072
PM043	PM043_S3	F	Vaginal	16SV4	PM043_S3_L001_R1.fastq	PM043_S3_L001_R2.fastq	76317	60198
PM044	PM044_S1	F	Oral	16SV4	PM044_S1_L001_R1.fastq	PM044_S1_L001_R2.fastq	53966	48863
PM044	PM044_S2	F	Anal	16SV4	PM044_S2_L001_R1.fastq	PM044_S2_L001_R2.fastq	49725	44033
PM044	PM044_S3	F	Vaginal	16SV4	PM044_S3_L001_R1.fastq	PM044_S3_L001_R2.fastq	20833	16123
PM045	PM045_S1	F	Oral	16SV4	PM045_S1_L001_R1.fastq	PM045_S1_L001_R2.fastq	63052	55432
PM045	PM045_S2	F	Anal	16SV4	PM045_S2_L001_R1.fastq	PM045_S2_L001_R2.fastq	63681	55548
PM045	PM045_S3	F	Vaginal	16SV4	PM045_S3_L001_R1.fastq	PM045_S3_L001_R2.fastq	68123	58893
PM046	PM046_S1	M	Oral	16SV4	PM046_S1_L001_R1.fastq	PM046_S1_L001_R2.fastq	38352	33911
PM046	PM046_S2	M	Anal	16SV4	PM046_S2_L001_R1.fastq	PM046_S2_L001_R2.fastq	50697	39251
PM047	PM047_S1	F	Oral	16SV4	PM047_S1_L001_R1.fastq	PM047_S1_L001_R2.fastq	58421	52071
PM047	PM047_S2	F	Anal	16SV4	PM047_S2_L001_R1.fastq	PM047_S2_L001_R2.fastq	62433	50512
PM047	PM047_S3	F	Vaginal	16SV4	PM047_S3_L001_R1.fastq	PM047_S3_L001_R2.fastq	59664	44569
PM048	PM048_S1	F	Oral	16SV4	PM048_S1_L001_R1.fastq	PM048_S1_L001_R2.fastq	69559	62306
PM048	PM048_S2	F	Anal	16SV4	PM048_S2_L001_R1.fastq	PM048_S2_L001_R2.fastq	57344	52323
PM048	PM048_S3	F	Vaginal	16SV4	PM048_S3_L001_R1.fastq	PM048_S3_L001_R2.fastq	21640	19363
PM049	PM049_S1	F	Oral	16SV4	PM049_S1_L001_R1.fastq	PM049_S1_L001_R2.fastq	44444	39322
PM049	PM049_S2	F	Anal	16SV4	PM049_S2_L001_R1.fastq	PM049_S2_L001_R2.fastq	61327	56264
PM049	PM049_S3	F	Vaginal	16SV4	PM049_S3_L001_R1.fastq	PM049_S3_L001_R2.fastq	57955	33451
PM050	PM050_S1	F	Oral	16SV4	PM050_S1_L001_R1.fastq	PM050_S1_L001_R2.fastq	54041	48563
PM050	PM050_S2	F	Anal	16SV4	PM050_S2_L001_R1.fastq	PM050_S2_L001_R2.fastq	52364	44566
PM050	PM050_S3	F	Vaginal	16SV4	PM050_S3_L001_R1.fastq	PM050_S3_L001_R2.fastq	65476	52701
PM051	PM051_S1	F	Oral	16SV4	PM051_S1_L001_R1.fastq	PM051_S1_L001_R2.fastq	46528	41252
PM051	PM051_S2	F	Anal	16SV4	PM051_S2_L001_R1.fastq	PM051_S2_L001_R2.fastq	64034	45681
PM051	PM051_S3	F	Vaginal	16SV4	PM051_S3_L001_R1.fastq	PM051_S3_L001_R2.fastq	66930	50501
PM052	PM052_S1	F	Oral	16SV4	PM052_S1_L001_R1.fastq	PM052_S1_L001_R2.fastq	15678	10779
PM052	PM052_S2	F	Anal	16SV4	PM052_S2_L001_R1.fastq	PM052_S2_L001_R2.fastq	15392	9287

PM052	PM052_S3	F	Vaginal	16SV4	PM052_S3_L001_R1.fastq	PM052_S3_L001_R2.fastq	4239	2763
PM053	PM053_S1	F	Oral	16SV4	PM053_S1_L001_R1.fastq	PM053_S1_L001_R2.fastq	67561	61214
PM053	PM053_S2	F	Anal	16SV4	PM053_S2_L001_R1.fastq	PM053_S2_L001_R2.fastq	70427	57516
PM053	PM053_S3	F	Vaginal	16SV4	PM053_S3_L001_R1.fastq	PM053_S3_L001_R2.fastq	69045	60272
PM054	PM054_S1	F	Oral	16SV4	PM054_S1_L001_R1.fastq	PM054_S1_L001_R2.fastq	40139	32229
PM054	PM054_S2	F	Anal	16SV4	PM054_S2_L001_R1.fastq	PM054_S2_L001_R2.fastq	44310	31323
PM054	PM054_S3	F	Vaginal	16SV4	PM054_S3_L001_R1.fastq	PM054_S3_L001_R2.fastq	38739	22189
PM055	PM055_S1	M	Oral	16SV4	PM055_S1_L001_R1.fastq	PM055_S1_L001_R2.fastq	65837	58955
PM055	PM055_S2	M	Anal	16SV4	PM055_S2_L001_R1.fastq	PM055_S2_L001_R2.fastq	32311	29572
PM056	PM056_S1	F	Oral	16SV4	PM056_S1_L001_R1.fastq	PM056_S1_L001_R2.fastq	61165	54315
PM056	PM056_S2	F	Anal	16SV4	PM056_S2_L001_R1.fastq	PM056_S2_L001_R2.fastq	58142	51205
PM056	PM056_S3	F	Vaginal	16SV4	PM056_S3_L001_R1.fastq	PM056_S3_L001_R2.fastq	75577	63672
PM057	PM057_S1	F	Oral	16SV4	PM057_S1_L001_R1.fastq	PM057_S1_L001_R2.fastq	41662	38397
PM057	PM057_S2	F	Anal	16SV4	PM057_S2_L001_R1.fastq	PM057_S2_L001_R2.fastq	65769	52582
PM057	PM057_S3	F	Vaginal	16SV4	PM057_S3_L001_R1.fastq	PM057_S3_L001_R2.fastq	50123	43249
PM058	PM058_S1	F	Oral	16SV4	PM058_S1_L001_R1.fastq	PM058_S1_L001_R2.fastq	27709	24545
PM058	PM058_S2	F	Anal	16SV4	PM058_S2_L001_R1.fastq	PM058_S2_L001_R2.fastq	35315	27866
PM058	PM058_S3	F	Vaginal	16SV4	PM058_S3_L001_R1.fastq	PM058_S3_L001_R2.fastq	41527	32790
PM059	PM059_S1	F	Oral	16SV4	PM059_S1_L001_R1.fastq	PM059_S1_L001_R2.fastq	48686	44430
PM059	PM059_S2	F	Anal	16SV4	PM059_S2_L001_R1.fastq	PM059_S2_L001_R2.fastq	55975	44681
PM059	PM059_S3	F	Vaginal	16SV4	PM059_S3_L001_R1.fastq	PM059_S3_L001_R2.fastq	55106	47031
PM060	PM060_S1	F	Oral	16SV4	PM060_S1_L001_R1.fastq	PM060_S1_L001_R2.fastq	57339	48863
PM060	PM060_S2	F	Anal	16SV4	PM060_S2_L001_R1.fastq	PM060_S2_L001_R2.fastq	53509	41304
PM060	PM060_S3	F	Vaginal	16SV4	PM060_S3_L001_R1.fastq	PM060_S3_L001_R2.fastq	57917	45960
PM061	PM061_S1	F	Oral	16SV4	PM061_S1_L001_R1.fastq	PM061_S1_L001_R2.fastq	57243	50723
PM061	PM061_S2	F	Anal	16SV4	PM061_S2_L001_R1.fastq	PM061_S2_L001_R2.fastq	34660	30424
PM061	PM061_S3	F	Vaginal	16SV4	PM061_S3_L001_R1.fastq	PM061_S3_L001_R2.fastq	55958	44556
PM062	PM062_S1	F	Oral	16SV4	PM062_S1_L001_R1.fastq	PM062_S1_L001_R2.fastq	60821	55118
PM062	PM062_S2	F	Anal	16SV4	PM062_S2_L001_R1.fastq	PM062_S2_L001_R2.fastq	57738	49920
PM062	PM062_S3	F	Vaginal	16SV4	PM062_S3_L001_R1.fastq	PM062_S3_L001_R2.fastq	60145	47784
PM063	PM063_S1	F	Oral	16SV4	PM063_S1_L001_R1.fastq	PM063_S1_L001_R2.fastq	43158	37349
PM063	PM063_S2	F	Anal	16SV4	PM063_S2_L001_R1.fastq	PM063_S2_L001_R2.fastq	40046	32337
PM063	PM063_S3	F	Vaginal	16SV4	PM063_S3_L001_R1.fastq	PM063_S3_L001_R2.fastq	49213	38573
PM064	PM064_S1	F	Oral	16SV4	PM064_S1_L001_R1.fastq	PM064_S1_L001_R2.fastq	63851	56133
PM064	PM064_S2	F	Anal	16SV4	PM064_S2_L001_R1.fastq	PM064_S2_L001_R2.fastq	63634	53186
PM064	PM064_S3	F	Vaginal	16SV4	PM064_S3_L001_R1.fastq	PM064_S3_L001_R2.fastq	44355	36694
PM065	PM065_S1	F	Oral	16SV4	PM065_S1_L001_R1.fastq	PM065_S1_L001_R2.fastq	39826	32994
PM065	PM065_S2	F	Anal	16SV4	PM065_S2_L001_R1.fastq	PM065_S2_L001_R2.fastq	15591	12796
PM065	PM065_S3	F	Vaginal	16SV4	PM065_S3_L001_R1.fastq	PM065_S3_L001_R2.fastq	42048	33679
PM066	PM066_S1	F	Oral	16SV4	PM066_S1_L001_R1.fastq	PM066_S1_L001_R2.fastq	40094	34568
PM066	PM066_S2	F	Anal	16SV4	PM066_S2_L001_R1.fastq	PM066_S2_L001_R2.fastq	47880	40176
PM066	PM066_S3	F	Vaginal	16SV4	PM066_S3_L001_R1.fastq	PM066_S3_L001_R2.fastq	34500	25271
PM067	PM067_S1	F	Oral	16SV4	PM067_S1_L001_R1.fastq	PM067_S1_L001_R2.fastq	65887	58548
PM067	PM067_S2	F	Anal	16SV4	PM067_S2_L001_R1.fastq	PM067_S2_L001_R2.fastq	62014	54298
PM067	PM067_S3	F	Vaginal	16SV4	PM067_S3_L001_R1.fastq	PM067_S3_L001_R2.fastq	75697	57332
PM068	PM068_S1	M	Oral	16SV4	PM068_S1_L001_R1.fastq	PM068_S1_L001_R2.fastq	36654	30776
PM068	PM068_S2	M	Anal	16SV4	PM068_S2_L001_R1.fastq	PM068_S2_L001_R2.fastq	32180	23572
PM069	PM069_S1	F	Oral	16SV4	PM069_S1_L001_R1.fastq	PM069_S1_L001_R2.fastq	65912	58956
PM069	PM069_S2	F	Anal	16SV4	PM069_S2_L001_R1.fastq	PM069_S2_L001_R2.fastq	60722	50240
PM069	PM069_S3	F	Vaginal	16SV4	PM069_S3_L001_R1.fastq	PM069_S3_L001_R2.fastq	67984	58413
PM070	PM070_S1	F	Oral	16SV4	PM070_S1_L001_R1.fastq	PM070_S1_L001_R2.fastq	72487	66298
PM070	PM070_S2	F	Anal	16SV4	PM070_S2_L001_R1.fastq	PM070_S2_L001_R2.fastq	51676	47111
PM070	PM070_S3	F	Vaginal	16SV4	PM070_S3_L001_R1.fastq	PM070_S3_L001_R2.fastq	22339	20372
PM071	PM071_S1	F	Oral	16SV4	PM071_S1_L001_R1.fastq	PM071_S1_L001_R2.fastq	57868	51811
PM071	PM071_S2	F	Anal	16SV4	PM071_S2_L001_R1.fastq	PM071_S2_L001_R2.fastq	22542	20219
PM071	PM071_S3	F	Vaginal	16SV4	PM071_S3_L001_R1.fastq	PM071_S3_L001_R2.fastq	63524	56394
PM072	PM072_S1	F	Oral	16SV4	PM072_S1_L001_R1.fastq	PM072_S1_L001_R2.fastq	23533	20190
PM072	PM072_S2	F	Anal	16SV4	PM072_S2_L001_R1.fastq	PM072_S2_L001_R2.fastq	18201	14059
PM072	PM072_S3	F	Vaginal	16SV4	PM072_S3_L001_R1.fastq	PM072_S3_L001_R2.fastq	12684	8778
PM073	PM073_S1	F	Oral	16SV4	PM073_S1_L001_R1.fastq	PM073_S1_L001_R2.fastq	15607	11649
PM073	PM073_S2	F	Anal	16SV4	PM073_S2_L001_R1.fastq	PM073_S2_L001_R2.fastq	38056	27524
PM073	PM073_S3	F	Vaginal	16SV4	PM073_S3_L001_R1.fastq	PM073_S3_L001_R2.fastq	33487	21763
PM074	PM074_S1	M	Oral	16SV4	PM074_S1_L001_R1.fastq	PM074_S1_L001_R2.fastq	8641	6407
PM074	PM074_S2	M	Anal	16SV4	PM074_S2_L001_R1.fastq	PM074_S2_L001_R2.fastq	7259	3437
PM075	PM075_S1	F	Oral	16SV4	PM075_S1_L001_R1.fastq	PM075_S1_L001_R2.fastq	62542	56085
PM075	PM075_S2	F	Anal	16SV4	PM075_S2_L001_R1.fastq	PM075_S2_L001_R2.fastq	70630	58987
PM075	PM075_S3	F	Vaginal	16SV4	PM075_S3_L001_R1.fastq	PM075_S3_L001_R2.fastq	66516	53312
PM076	PM076_S1	F	Oral	16SV4	PM076_S1_L001_R1.fastq	PM076_S1_L001_R2.fastq	58361	51536
PM076	PM076_S2	F	Anal	16SV4	PM076_S2_L001_R1.fastq	PM076_S2_L001_R2.fastq	67054	55480

PM076	PM076_S3	F	Vaginal	16SV4	PM076_S3_L001_R1.fastq	PM076_S3_L001_R2.fastq	65888	52326
PM077	PM077_S1	F	Oral	16SV4	PM077_S1_L001_R1.fastq	PM077_S1_L001_R2.fastq	16906	14803
PM077	PM077_S2	F	Anal	16SV4	PM077_S2_L001_R1.fastq	PM077_S2_L001_R2.fastq	16463	13274
PM077	PM077_S3	F	Vaginal	16SV4	PM077_S3_L001_R1.fastq	PM077_S3_L001_R2.fastq	28300	24403
PM078	PM078_S1	F	Oral	16SV4	PM078_S1_L001_R1.fastq	PM078_S1_L001_R2.fastq	49547	44426
PM078	PM078_S2	F	Anal	16SV4	PM078_S2_L001_R1.fastq	PM078_S2_L001_R2.fastq	52209	43640
PM078	PM078_S3	F	Vaginal	16SV4	PM078_S3_L001_R1.fastq	PM078_S3_L001_R2.fastq	54225	47000
PM079	PM079_S1	F	Oral	16SV4	PM079_S1_L001_R1.fastq	PM079_S1_L001_R2.fastq	47348	41678
PM079	PM079_S2	F	Anal	16SV4	PM079_S2_L001_R1.fastq	PM079_S2_L001_R2.fastq	40960	35868
PM079	PM079_S3	F	Vaginal	16SV4	PM079_S3_L001_R1.fastq	PM079_S3_L001_R2.fastq	42573	32677
PM080	PM080_S1	F	Oral	16SV4	PM080_S1_L001_R1.fastq	PM080_S1_L001_R2.fastq	46704	41190
PM080	PM080_S2	F	Anal	16SV4	PM080_S2_L001_R1.fastq	PM080_S2_L001_R2.fastq	44092	31849
PM080	PM080_S3	F	Vaginal	16SV4	PM080_S3_L001_R1.fastq	PM080_S3_L001_R2.fastq	45410	37632
PM081	PM081_S1	F	Oral	16SV4	PM081_S1_L001_R1.fastq	PM081_S1_L001_R2.fastq	66853	60029
PM081	PM081_S2	F	Anal	16SV4	PM081_S2_L001_R1.fastq	PM081_S2_L001_R2.fastq	62041	51738
PM081	PM081_S3	F	Vaginal	16SV4	PM081_S3_L001_R1.fastq	PM081_S3_L001_R2.fastq	30145	25929
PM082	PM082_S1	F	Oral	16SV4	PM082_S1_L001_R1.fastq	PM082_S1_L001_R2.fastq	56689	49665
PM082	PM082_S2	F	Anal	16SV4	PM082_S2_L001_R1.fastq	PM082_S2_L001_R2.fastq	64254	51426
PM082	PM082_S3	F	Vaginal	16SV4	PM082_S3_L001_R1.fastq	PM082_S3_L001_R2.fastq	17604	15302
PM083	PM083_S1	M	Oral	16SV4	PM083_S1_L001_R1.fastq	PM083_S1_L001_R2.fastq	56629	49320
PM083	PM083_S2	M	Anal	16SV4	PM083_S2_L001_R1.fastq	PM083_S2_L001_R2.fastq	17390	11714
PM084	PM084_S1	M	Oral	16SV4	PM084_S1_L001_R1.fastq	PM084_S1_L001_R2.fastq	68321	62228
PM084	PM084_S2	M	Anal	16SV4	PM084_S2_L001_R1.fastq	PM084_S2_L001_R2.fastq	58818	49021
PM085	PM085_S1	F	Oral	16SV4	PM085_S1_L001_R1.fastq	PM085_S1_L001_R2.fastq	50023	45407
PM085	PM085_S2	F	Anal	16SV4	PM085_S2_L001_R1.fastq	PM085_S2_L001_R2.fastq	70044	61768
PM085	PM085_S3	F	Vaginal	16SV4	PM085_S3_L001_R1.fastq	PM085_S3_L001_R2.fastq	63131	53096
PM086	PM086_S1	F	Oral	16SV4	PM086_S1_L001_R1.fastq	PM086_S1_L001_R2.fastq	52814	47669
PM086	PM086_S2	F	Anal	16SV4	PM086_S2_L001_R1.fastq	PM086_S2_L001_R2.fastq	41138	36297
PM086	PM086_S3	F	Vaginal	16SV4	PM086_S3_L001_R1.fastq	PM086_S3_L001_R2.fastq	58529	47170
PM087	PM087_S1	F	Oral	16SV4	PM087_S1_L001_R1.fastq	PM087_S1_L001_R2.fastq	43581	37654
PM087	PM087_S2	F	Anal	16SV4	PM087_S2_L001_R1.fastq	PM087_S2_L001_R2.fastq	68160	62119
PM087	PM087_S3	F	Vaginal	16SV4	PM087_S3_L001_R1.fastq	PM087_S3_L001_R2.fastq	70109	57243
PM088	PM088_S1	M	Oral	16SV4	PM088_S1_L001_R1.fastq	PM088_S1_L001_R2.fastq	53901	46673
PM088	PM088_S2	M	Anal	16SV4	PM088_S2_L001_R1.fastq	PM088_S2_L001_R2.fastq	61022	54862
PM089	PM089_S1	F	Oral	16SV4	PM089_S1_L001_R1.fastq	PM089_S1_L001_R2.fastq	65952	59562
PM089	PM089_S2	F	Anal	16SV4	PM089_S2_L001_R1.fastq	PM089_S2_L001_R2.fastq	67636	55762
PM089	PM089_S3	F	Vaginal	16SV4	PM089_S3_L001_R1.fastq	PM089_S3_L001_R2.fastq	53651	35288
PM090	PM090_S1	F	Oral	16SV4	PM090_S1_L001_R1.fastq	PM090_S1_L001_R2.fastq	44979	38932
PM090	PM090_S2	F	Anal	16SV4	PM090_S2_L001_R1.fastq	PM090_S2_L001_R2.fastq	39558	34729
PM090	PM090_S3	F	Vaginal	16SV4	PM090_S3_L001_R1.fastq	PM090_S3_L001_R2.fastq	27609	23313
PM091	PM091_S1	M	Oral	16SV4	PM091_S1_L001_R1.fastq	PM091_S1_L001_R2.fastq	50689	44819
PM091	PM091_S2	M	Anal	16SV4	PM091_S2_L001_R1.fastq	PM091_S2_L001_R2.fastq	44341	39357
PM092	PM092_S1	F	Oral	16SV4	PM092_S1_L001_R1.fastq	PM092_S1_L001_R2.fastq	43584	37356
PM092	PM092_S2	F	Anal	16SV4	PM092_S2_L001_R1.fastq	PM092_S2_L001_R2.fastq	52250	47236
PM092	PM092_S3	F	Vaginal	16SV4	PM092_S3_L001_R1.fastq	PM092_S3_L001_R2.fastq	32087	20111
PM093	PM093_S1	F	Oral	16SV4	PM093_S1_L001_R1.fastq	PM093_S1_L001_R2.fastq	65213	57152
PM093	PM093_S2	F	Anal	16SV4	PM093_S2_L001_R1.fastq	PM093_S2_L001_R2.fastq	57241	50687
PM093	PM093_S3	F	Vaginal	16SV4	PM093_S3_L001_R1.fastq	PM093_S3_L001_R2.fastq	48078	37853
PM094	PM094_S1	F	Oral	16SV4	PM094_S1_L001_R1.fastq	PM094_S1_L001_R2.fastq	45648	40470
PM094	PM094_S2	F	Anal	16SV4	PM094_S2_L001_R1.fastq	PM094_S2_L001_R2.fastq	10503	3587
PM094	PM094_S3	F	Vaginal	16SV4	PM094_S3_L001_R1.fastq	PM094_S3_L001_R2.fastq	60359	52197
PM095	PM095_S1	F	Oral	16SV4	PM095_S1_L001_R1.fastq	PM095_S1_L001_R2.fastq	40385	36323
PM095	PM095_S2	F	Anal	16SV4	PM095_S2_L001_R1.fastq	PM095_S2_L001_R2.fastq	59943	49542
PM095	PM095_S3	F	Vaginal	16SV4	PM095_S3_L001_R1.fastq	PM095_S3_L001_R2.fastq	16845	14801
PM096	PM096_S1	F	Oral	16SV4	PM096_S1_L001_R1.fastq	PM096_S1_L001_R2.fastq	27842	25146
PM096	PM096_S2	F	Anal	16SV4	PM096_S2_L001_R1.fastq	PM096_S2_L001_R2.fastq	65893	59566
PM096	PM096_S3	F	Vaginal	16SV4	PM096_S3_L001_R1.fastq	PM096_S3_L001_R2.fastq	58103	44890
PM097	PM097_S1	F	Oral	16SV4	PM097_S1_L001_R1.fastq	PM097_S1_L001_R2.fastq	54078	46138
PM097	PM097_S2	F	Anal	16SV4	PM097_S2_L001_R1.fastq	PM097_S2_L001_R2.fastq	36270	31307
PM097	PM097_S3	F	Vaginal	16SV4	PM097_S3_L001_R1.fastq	PM097_S3_L001_R2.fastq	30897	28096
PM098	PM098_S1	F	Oral	16SV4	PM098_S1_L001_R1.fastq	PM098_S1_L001_R2.fastq	22881	17321
PM098	PM098_S2	F	Anal	16SV4	PM098_S2_L001_R1.fastq	PM098_S2_L001_R2.fastq	16150	13049
PM098	PM098_S3	F	Vaginal	16SV4	PM098_S3_L001_R1.fastq	PM098_S3_L001_R2.fastq	63328	48536
PM099	PM099_S1	F	Oral	16SV4	PM099_S1_L001_R1.fastq	PM099_S1_L001_R2.fastq	37944	34273
PM099	PM099_S2	F	Anal	16SV4	PM099_S2_L001_R1.fastq	PM099_S2_L001_R2.fastq	62061	52853
PM099	PM099_S3	F	Vaginal	16SV4	PM099_S3_L001_R1.fastq	PM099_S3_L001_R2.fastq	68118	56030
PM100	PM100_S1	M	Oral	16SV4	PM100_S1_L001_R1.fastq	PM100_S1_L001_R2.fastq	56750	50596
PM100	PM100_S2	M	Anal	16SV4	PM100_S2_L001_R1.fastq	PM100_S2_L001_R2.fastq	61519	50244
PM101	PM101_S1	M	Oral	16SV4	PM101_S1_L001_R1.fastq	PM101_S1_L001_R2.fastq	59103	52319

PM101	PM101_S2	M	Anal	16SV4	PM101_S2_L001_R1.fastq	PM101_S2_L001_R2.fastq	39815	35948
PM102	PM102_S1	M	Oral	16SV4	PM102_S1_L001_R1.fastq	PM102_S1_L001_R2.fastq	57127	51298
PM102	PM102_S2	M	Anal	16SV4	PM102_S2_L001_R1.fastq	PM102_S2_L001_R2.fastq	33238	52832
PM103	PM103_S1	F	Oral	16SV4	PM103_S1_L001_R1.fastq	PM103_S1_L001_R2.fastq	51831	45895
PM103	PM103_S2	F	Anal	16SV4	PM103_S2_L001_R1.fastq	PM103_S2_L001_R2.fastq	64349	53535
PM103	PM103_S3	F	Vaginal	16SV4	PM103_S3_L001_R1.fastq	PM103_S3_L001_R2.fastq	48354	39606
PM104	PM104_S1	F	Oral	16SV4	PM104_S1_L001_R1.fastq	PM104_S1_L001_R2.fastq	52398	47891
PM104	PM104_S2	F	Anal	16SV4	PM104_S2_L001_R1.fastq	PM104_S2_L001_R2.fastq	28326	23492
PM104	PM104_S3	F	Vaginal	16SV4	PM104_S3_L001_R1.fastq	PM104_S3_L001_R2.fastq	40358	20254
PM105	PM105_S1	F	Oral	16SV4	PM105_S1_L001_R1.fastq	PM105_S1_L001_R2.fastq	59534	50794
PM105	PM105_S2	F	Anal	16SV4	PM105_S2_L001_R1.fastq	PM105_S2_L001_R2.fastq	35611	27062
PM105	PM105_S3	F	Vaginal	16SV4	PM105_S3_L001_R1.fastq	PM105_S3_L001_R2.fastq	62303	55156
PM106	PM106_S1	F	Oral	16SV4	PM106_S1_L001_R1.fastq	PM106_S1_L001_R2.fastq	7210	4706
PM106	PM106_S2	F	Anal	16SV4	PM106_S2_L001_R1.fastq	PM106_S2_L001_R2.fastq	54617	44954
PM106	PM106_S3	F	Vaginal	16SV4	PM106_S3_L001_R1.fastq	PM106_S3_L001_R2.fastq	56759	46536
PM107	PM107_S1	F	Oral	16SV4	PM107_S1_L001_R1.fastq	PM107_S1_L001_R2.fastq	46715	42766
PM107	PM107_S2	F	Anal	16SV4	PM107_S2_L001_R1.fastq	PM107_S2_L001_R2.fastq	18198	12695
PM107	PM107_S3	F	Vaginal	16SV4	PM107_S3_L001_R1.fastq	PM107_S3_L001_R2.fastq	13350	11874
PM108	PM108_S1	F	Oral	16SV4	PM108_S1_L001_R1.fastq	PM108_S1_L001_R2.fastq	20408	18844
PM108	PM108_S2	F	Anal	16SV4	PM108_S2_L001_R1.fastq	PM108_S2_L001_R2.fastq	49965	42349
PM108	PM108_S3	F	Vaginal	16SV4	PM108_S3_L001_R1.fastq	PM108_S3_L001_R2.fastq	35430	17409
PM109	PM109_S1	F	Oral	16SV4	PM109_S1_L001_R1.fastq	PM109_S1_L001_R2.fastq	43135	38404
PM109	PM109_S2	F	Anal	16SV4	PM109_S2_L001_R1.fastq	PM109_S2_L001_R2.fastq	54700	49449
PM109	PM109_S3	F	Vaginal	16SV4	PM109_S3_L001_R1.fastq	PM109_S3_L001_R2.fastq	59081	45184
PM110	PM110_S1	F	Oral	16SV4	PM110_S1_L001_R1.fastq	PM110_S1_L001_R2.fastq	49865	45608
PM110	PM110_S2	F	Anal	16SV4	PM110_S2_L001_R1.fastq	PM110_S2_L001_R2.fastq	50355	39619
PM110	PM110_S3	F	Vaginal	16SV4	PM110_S3_L001_R1.fastq	PM110_S3_L001_R2.fastq	58267	48458
PM111	PM111_S1	M	Oral	16SV4	PM111_S1_L001_R1.fastq	PM111_S1_L001_R2.fastq	42157	37585
PM111	PM111_S2	M	Anal	16SV4	PM111_S2_L001_R1.fastq	PM111_S2_L001_R2.fastq	71891	60665
PM112	PM112_S1	F	Oral	16SV4	PM112_S1_L001_R1.fastq	PM112_S1_L001_R2.fastq	40784	36549
PM112	PM112_S2	F	Anal	16SV4	PM112_S2_L001_R1.fastq	PM112_S2_L001_R2.fastq	53947	47514
PM112	PM112_S3	F	Vaginal	16SV4	PM112_S3_L001_R1.fastq	PM112_S3_L001_R2.fastq	63041	38824
PM113	PM113_S1	F	Oral	16SV4	PM113_S1_L001_R1.fastq	PM113_S1_L001_R2.fastq	37873	33559
PM113	PM113_S2	F	Anal	16SV4	PM113_S2_L001_R1.fastq	PM113_S2_L001_R2.fastq	68225	54991
PM113	PM113_S3	F	Vaginal	16SV4	PM113_S3_L001_R1.fastq	PM113_S3_L001_R2.fastq	66351	56086
PM114	PM114_S1	M	Oral	16SV4	PM114_S1_L001_R1.fastq	PM114_S1_L001_R2.fastq	51137	46242
PM114	PM114_S2	M	Anal	16SV4	PM114_S2_L001_R1.fastq	PM114_S2_L001_R2.fastq	49146	37257
PM115	PM115_S1	F	Oral	16SV4	PM115_S1_L001_R1.fastq	PM115_S1_L001_R2.fastq	56412	51338
PM115	PM115_S2	F	Anal	16SV4	PM115_S2_L001_R1.fastq	PM115_S2_L001_R2.fastq	52109	45533
PM115	PM115_S3	F	Vaginal	16SV4	PM115_S3_L001_R1.fastq	PM115_S3_L001_R2.fastq	39025	34772
PM116	PM116_S1	M	Oral	16SV4	PM116_S1_L001_R1.fastq	PM116_S1_L001_R2.fastq	66377	59872
PM116	PM116_S2	M	Anal	16SV4	PM116_S2_L001_R1.fastq	PM116_S2_L001_R2.fastq	65675	52614
PM117	PM117_S1	F	Oral	16SV4	PM117_S1_L001_R1.fastq	PM117_S1_L001_R2.fastq	9745	6395
PM117	PM117_S2	F	Anal	16SV4	PM117_S2_L001_R1.fastq	PM117_S2_L001_R2.fastq	53748	44678
PM117	PM117_S3	F	Vaginal	16SV4	PM117_S3_L001_R1.fastq	PM117_S3_L001_R2.fastq	44973	32373

Table S2. Human individual from the HMP dataset.

SampleID	RSID	PSN	NAP	ExperimentAccession	RunID	SRS_SampleID	SeqID	Sex	HMPBodySubsite	HMPBodySite	Size	File_name	16S reads (raw)	16S reads (QC)
SRS011407.SRX020666.V35	159247771	700015991	700015993	SRX020666	SRR044929	SRS011407	SRS011407	female	Saliva	Oral	5368962	SRS011407.fna	8603	7024
SRS011482.SRX020660.V35	159085930	700016299	700016315	SRX020660	SRR044841	SRS011482	SRS011482	female	Saliva	Oral	5958472	SRS011482.fna	9697	8025
SRS011504.SRX020666.V35	159005010	700016344	700016356	SRX020666	SRR044961	SRS011504	SRS011504	female	Saliva	Oral	2351606	SRS011504.fna	3815	2969
SRS011588.SRX020666.V35	159733294	700033134	700033161	SRX020666	SRR044926	SRS011588	SRS011588	female	Saliva	Oral	2183484	SRS011588.fna	3531	2980
SRS011623.SRX020666.V35	159470302	700033448	700033450	SRX020666	SRR044984	SRS011623	SRS011623	female	Saliva	Oral	4451913	SRS011623.fna	7185	5382
SRS011655.SRX020660.V35	159713063	700033730	700033757	SRX020660	SRR044881	SRS011655	SRS011655	female	Saliva	Oral	7291159	SRS011655.fna	11875	9917
SRS013551.SRX020681.V35	159227541	700016546	700016572	SRX020681	SRR048265	SRS013551	SRS013551	female	Saliva	Oral	2694030	SRS013551.fna	4398	3891
SRS014556.SRX020555.V35	763638144	700023180	700023210	SRX020555	SRR045697	SRS014556	SRS014556	female	Saliva	Oral	3791024	SRS014556.fna	6186	5938
SRS014803.SRX020555.V35	763921366	700023437	700023457	SRX020555	SRR045684	SRS014803	SRS014803	female	Saliva	Oral	5509548	SRS014803.fna	8975	8311
SRS015113.SRX020555.V35	763901136	700023691	700023768	SRX020555	SRR049330	SRS015113	SRS015113	female	Saliva	Oral	4373120	SRS015113.fna	7101	6741
SRS015170.SRX020558.V35	763759525	700023805	700023825	SRX020558	SRR043737	SRS015170	SRS015170	female	Saliva	Oral	3459140	SRS015170.fna	5617	5338
SRS015227.SRX020535.V35	764143897	700023862	700023882	SRX020535	SRR044003	SRS015227	SRS015227	female	Saliva	Oral	3218375	SRS015227.fna	5202	4965
SRS015704.SRX020573.V35	764467579	700024339	700024359	SRX020573	SRR044273	SRS015704	SRS015704	female	Saliva	Oral	4572789	SRS015704.fna	7421	6928
SRS016170.SRX020547.V35	764366428	700024798	700024827	SRX020547	SRR045744	SRS016170	SRS016170	female	Saliva	Oral	2748006	SRS016170.fna	4508	4203
SRS016221.SRX020522.V35	764649650	700024855	700024884	SRX020522	SRR044443	SRS016221	SRS016221	female	Saliva	Oral	2018112	SRS016221.fna	3313	3250
SRS016642.SRX019691.V35	160036745	700032425	700032426	SRX019691	SRR041586	SRS016642	SRS016642	female	Saliva	Oral	2888013	SRS016642.fna	4679	3815
SRS016708.SRX019682.V35	160016515	700032640	700032653	SRX019682	SRR040957	SRS016708	SRS016708	female	Saliva	Oral	2512264	SRS016708.fna	4098	3781
SRS018258.SRX019687.V35	160137896	700035639	700035665	SRX019687	SRR041200	SRS018258	SRS018258	female	Saliva	Oral	2335284	SRS018258.fna	3784	3285
SRS018497.SRX019687.V35	160259276	700035935	700035961	SRX019687	SRR041347	SRS018497	SRS018497	female	Saliva	Oral	2949534	SRS018497.fna	4771	3965
SRS018735.SRX020522.V35	765013792	700037124	700037202	SRX020522	SRR044398	SRS018735	SRS018735	female	Saliva	Oral	3746761	SRS018735.fna	6133	5889
SRS019263.SRX020566.V35	765034022	700037698	700037734	SRX020566	SRR046169	SRS019263	SRS019263	female	Saliva	Oral	3251241	SRS019263.fna	5269	5206
SRS021117.SRX022230.V35	160744799	700096594	700096664	SRX022230	SRR058093	SRS021117	SRS021117	female	Saliva	Oral	2629338	SRS021117.fna	4292	3565
SRS021183.SRX022239.V35	161007791	700096704	700096730	SRX022239	SRR058109	SRS021183	SRS021183	female	Saliva	Oral	5153513	SRS021183.fna	8382	7709
SRS021249.SRX022239.V35	161230322	700096869	700096895	SRX022239	SRR058109	SRS021249	SRS021249	female	Saliva	Oral	2419761	SRS021249.fna	3918	3356
SRS021372.SRX022239.V35	161028021	700097004	700097030	SRX022239	SRR058109	SRS021372	SRS021372	female	Saliva	Oral	1493145	SRS021372.fna	2424	2029
SRS021609.SRX020679.V35	158013734	700097295	700097321	SRX020679	SRR048050	SRS021609	SRS021609	female	Saliva	Oral	2547973	SRS021609.fna	4184	3406
SRS021732.SRX022231.V35	161493313	700097419	700097445	SRX022231	SRR058120	SRS021732	SRS021732	female	Saliva	Oral	2829287	SRS021732.fna	4602	4131
SRS021798.SRX022235.V35	161473083	700097485	700097511	SRX022235	SRR058095	SRS021798	SRS021798	female	Saliva	Oral	1542551	SRS021798.fna	2516	1885
SRS021978.SRX019683.V35	158256496	700097692	700097718	SRX019683	SRR041136	SRS021978	SRS021978	female	Saliva	Oral	2329695	SRS021978.fna	3792	3239
SRS022101.SRX019690.V35	158337416	700097841	700097867	SRX019690	SRR041600	SRS022101	SRS022101	female	Saliva	Oral	1880599	SRS022101.fna	3085	2725
SRS022167.SRX022231.V35	638754422	700097910	700097936	SRX022231	SRR058120	SRS022167	SRS022167	female	Saliva	Oral	2147016	SRS022167.fna	3502	3038
SRS022299.SRX022231.V35	533247696	700098113	700098139	SRX022231	SRR058120	SRS022299	SRS022299	female	Saliva	Oral	2974434	SRS022299.fna	4895	4303
SRS022422.SRX020680.V35	158742018	700098271	700098297	SRX020680	SRR048194	SRS022422	SRS022422	female	Saliva	Oral	3117648	SRS022422.fna	5084	4674
SRS022488.SRX020680.V35	158458797	700098363	700098389	SRX020680	SRR048176	SRS022488	SRS022488	female	Saliva	Oral	3047094	SRS022488.fna	4935	4658
SRS022554.SRX022226.V35	809635352	700098433	700098459	SRX022226	SRR058089	SRS022554	SRS022554	female	Saliva	Oral	1594017	SRS022554.fna	2599	2289
SRS022677.SRX022226.V35	432193348	700098603	700098629	SRX022226	SRR058089	SRS022677	SRS022677	female	Saliva	Oral	3720686	SRS022677.fna	6121	4418
SRS022743.SRX022226.V35	370425937	700098673	700098699	SRX022226	SRR058089	SRS022743	SRS022743	female	Saliva	Oral	2560892	SRS022743.fna	4192	3116
SRS022866.SRX022223.V35	650853796	700098870	700098896	SRX022223	SRR058086	SRS022866	SRS022866	female	Saliva	Oral	1576160	SRS022866.fna	2585	2341
SRS022932.SRX020679.V35	158236265	700098936	700098962	SRX020679	SRR048100	SRS022932	SRS022932	female	Saliva	Oral	3318543	SRS022932.fna	5391	4649
SRS022995.SRX020679.V35	158114885	700099006	700099029	SRX020679	SRR048061	SRS022995	SRS022995	female	Saliva	Oral	3390678	SRS022995.fna	5567	4666
SRS023055.SRX020679.V35	158276726	700099063	700099089	SRX020679	SRR048115	SRS023055	SRS023055	female	Saliva	Oral	2601728	SRS023055.fna	4204	3727
SRS023114.SRX022226.V35	441369442	700099129	700099245	SRX022226	SRR058089	SRS023114	SRS023114	female	Saliva	Oral	1695380	SRS023114.fna	2744	2449
SRS023310.SRX022223.V35	206906765	700099446	700099472	SRX022223	SRR058086	SRS023310	SRS023310	female	Saliva	Oral	1996007	SRS023310.fna	3272	2989
SRS023373.SRX022223.V35	246515023	700099516	700099539	SRX022223	SRR058086	SRS023373	SRS023373	female	Saliva	Oral	3536129	SRS023373.fna	5795	5397
SRS023859.SRX020681.V35	158883629	700100231	700100257	SRX020681	SRR048212	SRS023859	SRS023859	female	Saliva	Oral	4054860	SRS023859.fna	6652	5801
SRS024273.SRX019692.V35	158944319	700101228	700101251	SRX019692	SRR041908	SRS024273	SRS024273	female	Saliva	Oral	5553261	SRS024273.fna	8982	7968
SRS042258.SRX020533.V35	553359145	700106494	700106522	SRX020533	SRR049446	SRS042258	SRS042258	female	Saliva	Oral	3393896	SRS042258.fna	5602	5273
SRS042441.SRX020688.V35	159915365	700108600	700108626	SRX020688	SRR048722	SRS042441	SRS042441	female	Saliva	Oral	3472728	SRS042441.fna	5641	4422
SRS042483.SRX022242.V35	937415040	700102523	700102549	SRX022242	SRR058098	SRS042483	SRS042483	female	Saliva	Oral	10364363	SRS042483.fna	16869	13404
SRS042504.SRX020563.V35	737052003	700106066	700106095	SRX020563	SRR047696	SRS042504	SRS042504	female	Saliva	Oral	5784322	SRS042504.fna	9413	8883
SRS042607.SRX022227.V35	970836795	700109111	700109137	SRX022227	SRR058090	SRS042607	SRS042607	female	Saliva	Oral	3873611	SRS042607.fna	6322	5264
SRS045145.SRX022222.V35	892969023	700110291	700110317	SRX022222	SRR058085	SRS045145	SRS045145	female	Saliva	Oral	3353294	SRS045145.fna	5445	4424
SRS045158.SRX022221.V35	160866180	700109321	700109348	SRX022221	SRR058084	SRS045158	SRS045158	female	Saliva	Oral	9706260	SRS045158.fna	15767	13886
SRS045517.SRX021470.V35	414519462	700102117	700102143	SRX021470	SRR052699	SRS045517	SRS045517	female	Saliva	Oral	3334022	SRS045517.fna	5493	5084
SRS046269.SRX020684.V35	159753524	700107379	700107405	SRX020684	SRR048482	SRS046269	SRS046269	female	Saliva	Oral	5534766	SRS046269.fna	8999	8198
SRS047001.SRX020687.V35	160704339	700108222	700108248	SRX020687	SRR048672	SRS047001	SRS047001	female	Saliva	Oral	7126048	SRS047001.fna	11621	10411
SRS050005.SRX020533.V35	612472597	700105154	700105183	SRX020533	SRR046858	SRS050005	SRS050005	female	Saliva	Oral	3928597	SRS050005.fna	6374	5914
SRS050119.SRX020687.V35	160643649	700110093	700110119	SRX020687	SRR048661	SRS050119	SRS050119	female	Saliva	Oral	4530513	SRS050119.fna	7441	6756
SRS050388.SRX020686.V35	160582958	700108534	700108560	SRX020686	SRR048634	SRS050388	SRS050388	female	Saliva	Oral	3557250	SRS050388.fna	5778	4991
SRS050971.SRX020665.V35	763456073	700105667	700105687	SRX020665	SRR046309	SRS050971	SRS050971	female	Saliva	Oral	2285099	SRS050971.fna	3741	3598
SRS054258.SRX020686.V35	160502038	700108099	700108125	SRX020686	SRR048616	SRS054258	SRS054258	female	Saliva	Oral	5217969	SRS054258.fna	8513	7802
SRS054953.SRX020685.V35	160319967	700109625	700109651	SRX020685	SRR048563	SRS054953	SRS054953	female	Saliva	Oral	4078170	SRS054953.fna	6657	5737
SRS055808.SRX020533.V35	208027353	700106552	700106581	SRX020533	SRR046692	SRS055808	SRS055808	female	Saliva	Oral	2447351	SRS055808.fna	4001	3773
SRS055987.SRX020665.V35	763820215	700105211	700105240	SRX020665	SRR046273	SRS055987	SRS055987	female	Saliva	Oral	3963196	SRS055987.fna	6498	6218
SRS057603.SRX021470.V35	514014184	700101775	700101801	SRX021470	SRR052699	SRS057603	SRS057603	female	Saliva	Oral	2882941	SRS057603.fna	4699	4405
SRS062620.SRX020579.V35	375450439	700114807	700114836	SRX020579	SRR047091	SRS062620	SRS062620	female	Saliva	Oral	2859808	SRS062620.fna	4658	4294
SRS062708.SRX020689.V35	763721271	700111300	700111326	SRX020689	SRR048837	SRS062708	SRS062708	female	Saliva	Oral	4690648	SRS062708.fna	7571	6385
SRS062784.SRX020665.V35	763840445	700114106	700114127	SRX020665	SRR046308	SRS062784	SRS062784	female	Saliva	Oral	4745235	SRS062784.fna	7728	7360
SRS063388.SRX022237.V35	937495960	700111578	700111604	SRX022237	SRR058114	SRS063388	SRS063388	female	Saliva	Oral	1131675	SRS063388.fna	1852	1048
SRS063697.SRX020548.V35	763719065	700113485	700113505	SRX020548	SRR050136	SRS063697	SRS063697	female	Saliva	Oral	3199986	SRS063697.fna	5240	4883
SRS063891.SRX020689.V35	866653606	700111226	700111252	SRX020689	SRR048801	SRS063891	SRS063891	female	Saliva	Oral	3600358	SRS063891.fna	5835	4947
SRS064038.SRX020579.V35	663835652	700114219	700114248	SRX020579	SRR046979	SRS064038	SRS064038	female	Saliva	Oral	3837797	SRS064038.fna	6242	5660
SRS065061.SRX020689.V35	872848689	700111749	700111775	SRX020689	SRR048819	SRS065061	SRS065061	female	Saliva	Oral	4319896	SRS065061.fna	6993	5558
SRS065577.SRX020689.V35	257905678	700111160	700111186	SRX020689	SRR048783	SRS065577	SRS065577	female	Saliva	Oral	4048847	SRS065577.fna	6514	5140
SRS011159.SRX020660.V35	158822939	700014965	700014968	SRX020660	SRR044847	SRS011159	SRS011159	male	Saliva	Oral	7454240	SRS011159.fna	12186	9860
SRS011273.SRX020666.V35	158802708	700015257	700015260	SRX020666	SRR044970	SRS011273	SRS011273	male	Saliva	Oral	3925069	SRS011273.fna	6323	5047
SRS011417.SRX020666.V35	158964549	700016036	700016059	SRX020666	SRR044943	SRS011417	SRS011417	male	Saliva	Oral	2663939	SRS011417.fna	4315	3340
SRS011454.SRX020660.V35	159146620	700016123	700016150	SRX020660	SRR044853	SRS011454	SRS011454	male	Saliva	Oral	8680321	SRS011454.fna	14039	11513
SRS011531.SRX020660.V35	159166850	700016620	700016622	SRX020660	SRR044905	SRS011531	SRS011531	male	Saliva	Oral	8015501	SRS011531.fna	13059	10396
SRS013185.SRX019681.V35	159369152	700015861	700015884	SRX019681	SRR040881	SRS013185	SRS013185	male	Saliva	Oral	1883866	SRS013185.fna	3095	2432
SRS013228.SRX019639.V35	159591683	700015934	700015935	SRX019639	SRR040728	SRS013228	SRS013228	male	Saliva	Oral	2497808	SRS013228.fna	4024	3214
SRS013456.SRX019692.V35	159328691	700016377	700016387	SRX019692	SRR041861	SRS013456	SRS013456	male	Saliva	Oral	5932498	SRS013456.fna	9695	8659
SRS013498.SRX019683.V35	159207311	700016460	700016478	SRX019683	SRR041025	SRS013498	SRS013498	male	Saliva	Oral	2023939	SRS013498.fna	3302	2890
SRS013699.SRX019690.V35	159268001	700016776	700016777	SRX019690	SRR041612	SRS013699	SRS013699	male	Saliva	Oral	2657012	SRS013699.fna	4327	3997
SRS014857.SRX020558.V35	764002286	700023494	700023511	SRX020558	SRR049815	SRS014857	SRS014857	male	Saliva	Oral	5575157	SRS014857.fna	9057	8793
SRS014950.SRX020530.V35	763880905	700023589	700023605	SRX020530	SRR045804	SRS014950	SRS014950	male	Saliva	Oral	2875787	SRS014950.fna	4692	4439
SRS015293.SRX020558.V35	763982056	700023925	700023948	SRX020558	SRR043753	SRS015293	SRS015293	male	Saliva	Oral	5742205	SRS015293.fna	9254	8974
SRS015454.SRX020538.V35	764083206	700024087	700024109	SRX020538	SRR044657	SRS015454	SRS015454	male	Saliva	Oral	6781853	SRS015454.fna	11106	10305
SRS015556.SRX020535.V35	764285508	700024190	700024211	SRX020535	SRR049851	SRS015556	SRS015556	male	Saliva	Oral	2858534	SRS015556.fna	4640	4377
SRS015665.SRX020573.V35	764487809	700024297	700024320	SRX020573	SRR044262	SRS015665	SRS015665	male	Saliva	Oral	3363880	SRS015665.fna	5479	5349
SRS015758.SRX020573.V35	764224817	700024396	700024413	SRX020573	SRR044261	SRS015758	SRS015758	male	Saliva	Oral	3552605	SRS015758.fna	5845	5624
SRS015817.SRX020562.V35	763435843	700024450	700024472	SRX020562	SRR044698	SRS015817	SRS015817	male	Saliva	Oral	5451261	SRS015817.fna	8832	8161
SRS015998.SRX020573.V35	764447348	700024636	700024653	SRX020573	SRR047418	SRS015998	SRS015998	male	Saliva	Oral	3352139	SRS015998.fna	5464	5324
SRS016285.SRX020535.V35	764669880	700024917	700024948	SRX020535	SRR043984	SRS016285	SRS016285	male	Saliva	Oral	2892683	SRS016285.fna	4735	4331
SRS016408.SRX019688.V35	159672603	700032072	700032095	SRX019688	SRR041373	SRS016408	SRS016408	male	Saliva	Oral	2255635	SRS016408.fna	3642	2398
SRS016449.SRX019681.V35	159429842	700032144	700032145	SRX019681	SRR040979	SRS016449	SRS016449	male	Saliva	Oral	2133447	SRS016449.fna	3461	2801
SRS016853.SRX019692.V35	159308461	700033213	700033214	SRX019692	SRR041870	SRS016853	SRS016853	male	Saliva	Oral	6508155	SRS016853.fna	10570	9603
SRS016924.SRX019681.V35	159450072	700033367	700033390	SRX019681	SRR040927	SRS016924	SRS016924	male	Saliva	Oral	1855536	SRS016924.fna	3026	2273
SRS017072.SRX019687.V35	159611913	700033563	700033585	SRX019687	SRR041306	SRS017072	SRS017072	male	Saliva	Oral	2650904	SRS017072.fna	4277	3816
SRS017115.SRX019684.V35	159551223	700033676	700033677	SRX019684	SRR041127	SRS017115	SRS017115	male	Saliva	Oral	3062339	SRS017115.fna	4978	4192
SRS017203.SRX019688.V35	159814214	700033808	700033809	SRX019688	SRR041430	SRS017203	SRS017203	male	Saliva	Oral	2015821	SRS017203.fna	3254	2024
SRS017402.SRX019683.V35	160481808	700034085	700034108	SRX019683	SRR041179	SRS017402	SRS017402	male	Saliva	Oral	2047397	SRS017402.fna	3332	2866
SRS017649.SRX019683.V35	160461578	700034626	700034649	SRX019683	SRR041058	SRS017649	SRS017649	male	Saliva	Oral	2159595	SRS017649.fna	3484	2960
SRS017772.SRX019639.V35	160542498	700034842	700034865	SRX019639	SRR040794	SRS017772	SRS017772	male	Saliva	Oral	1939933	SRS017772.fna	3148	2594
SRS017829.SRX019686.V35	160603188	700034911	700034934	SRX019686	SRR041345	SRS017829	SRS017829	male	Saliva	Oral	3811353	SRS017829.fna	6194	5503
SRS017886.SRX019688.V35	160441347	700035161	700035184	SRX019688	SRR041368	SRS017886	SRS017886	male	Saliva	Oral	2071905	SRS017886.fna	3318	2394
SRS017943.SRX019690.V35	158357646	700035241	700035264	SRX019690	SRR041602	SRS017943	SRS017943	male	Saliva	Oral	2460362	SRS017943.fna	4054	3716
SRS018005.SRX019639.V35	160239046	700035301	700035329	SRX019639	SRR040783	SRS018005	SRS018005	male	Saliva	Oral	2349339	SRS018005.fna	3810	2981

SRS018141.SRX019682.V35	160158126	700035518	700035541	SRX019682	SRR040910	SRS018141	SRS018141	male	Saliva	Oral	2088377	SRS018141.fna	3389	2630
SRS018587.SRX020522.V35	765094712	700037032	700037054	SRX020522	SRR044447	SRS018587	SRS018587	male	Saliva	Oral	3833368	SRS018587.fna	6193	6008
SRS018787.SRX020530.V35	765135172	700037237	700037254	SRX020530	SRR045799	SRS018787	SRS018787	male	Saliva	Oral	2744564	SRS018787.fna	4461	4057
SRS019011.SRX020510.V35	764305738	700037451	700037480	SRX020510	SRR044594	SRS019011	SRS019011	male	Saliva	Oral	4028560	SRS019011.fna	6597	6342
SRS019890.SRX020522.V35	764892411	700038344	700038366	SRX020522	SRR044401	SRS019890	SRS019890	male	Saliva	Oral	4621012	SRS019890.fna	7502	7256
SRS020127.SRX020680.V35	158398106	700095357	700095380	SRX020680	SRR048133	SRS020127	SRS020127	male	Saliva	Oral	3493336	SRS020127.fna	5668	4934
SRS020184.SRX020680.V35	158418336	700095414	700095437	SRX020680	SRR048148	SRS020184	SRS020184	male	Saliva	Oral	3094014	SRS020184.fna	5072	4789
SRS020298.SRX019684.V35	160663879	700095528	700095551	SRX019684	SRR041125	SRS020298	SRS020298	male	Saliva	Oral	2703651	SRS020298.fna	4355	3730
SRS020421.SRX019682.V35	160684109	700095721	700095744	SRX019682	SRR040922	SRS020421	SRS020421	male	Saliva	Oral	2454380	SRS020421.fna	3988	3510
SRS020592.SRX022230.V35	160906640	700095960	700095983	SRX022230	SRR058093	SRS020592	SRS020592	male	Saliva	Oral	2131449	SRS020592.fna	3491	2817
SRS020819.SRX022230.V35	160886410	700096271	700096293	SRX022230	SRR058093	SRS020819	SRS020819	male	Saliva	Oral	1704325	SRS020819.fna	2766	2275
SRS020934.SRX022239.V35	161068481	700096422	700096445	SRX022239	SRR058109	SRS020934	SRS020934	male	Saliva	Oral	2240230	SRS020934.fna	3643	3142
SRS021312.SRX022235.V35	161351702	700096947	700096970	SRX022235	SRR058095	SRS021312	SRS021312	male	Saliva	Oral	2513730	SRS021312.fna	4065	3460
SRS021492.SRX019690.V35	158479027	700097181	700097204	SRX019690	SRR041639	SRS021492	SRS021492	male	Saliva	Oral	2222726	SRS021492.fna	3615	3433
SRS021549.SRX020679.V35	158216035	700097238	700097261	SRX020679	SRR048088	SRS021549	SRS021549	male	Saliva	Oral	4090800	SRS021549.fna	6700	5818
SRS021672.SRX022235.V35	161331472	700097361	700097384	SRX022235	SRR058095	SRS021672	SRS021672	male	Saliva	Oral	1812613	SRS021672.fna	2948	2370
SRS021861.SRX022235.V35	161270782	700097574	700097597	SRX022235	SRR058095	SRS021861	SRS021861	male	Saliva	Oral	1589108	SRS021861.fna	2593	1965
SRS021918.SRX020679.V35	158155345	700097635	700097658	SRX020679	SRR048077	SRS021918	SRS021918	male	Saliva	Oral	3317887	SRS021918.fna	5367	4226
SRS022041.SRX022231.V35	161554003	700097758	700097781	SRX022231	SRR058120	SRS022041	SRS022041	male	Saliva	Oral	2187388	SRS022041.fna	3526	3128
SRS022362.SRX022226.V35	492786515	700098205	700098228	SRX022226	SRR058089	SRS022362	SRS022362	male	Saliva	Oral	1874050	SRS022362.fna	3065	2768
SRS022617.SRX019692.V35	158499257	700098546	700098569	SRX019692	SRR041926	SRS022617	SRS022617	male	Saliva	Oral	6001133	SRS022617.fna	9837	8734
SRS022806.SRX022223.V35	295137534	700098739	700098762	SRX022223	SRR058086	SRS022806	SRS022806	male	Saliva	Oral	2384868	SRS022806.fna	3918	3587
SRS023184.SRX019692.V35	158924089	700099292	700099315	SRX019692	SRR041710	SRS023184	SRS023184	male	Saliva	Oral	4500926	SRS023184.fna	7315	6805
SRS023250.SRX022229.V35	964271349	700099385	700099408	SRX022229	SRR058092	SRS023250	SRS023250	male	Saliva	Oral	2339148	SRS023250.fna	3842	3008
SRS023430.SRX020680.V35	158438567	700099594	700099617	SRX020680	SRR048162	SRS023430	SRS023430	male	Saliva	Oral	2329500	SRS023430.fna	3803	3644
SRS023742.SRX019681.V35	158721788	700100026	700100049	SRX019681	SRR040902	SRS023742	SRS023742	male	Saliva	Oral	2218778	SRS023742.fna	3615	3124
SRS023799.SRX021470.V35	336497421	700100169	700100192	SRX021470	SRR052699	SRS023799	SRS023799	male	Saliva	Oral	1898890	SRS023799.fna	3093	2741
SRS024102.SRX019686.V35	159510762	700100544	700100567	SRX019686	SRR041242	SRS024102	SRS024102	male	Saliva	Oral	4796745	SRS024102.fna	7838	7134
SRS024462.SRX019684.V35	159571453	700101585	700101608	SRX019684	SRR041166	SRS024462	SRS024462	male	Saliva	Oral	2552309	SRS024462.fna	4131	3466
SRS024519.SRX020680.V35	159389382	700101642	700101665	SRX020680	SRR048170	SRS024519	SRS024519	male	Saliva	Oral	3637622	SRS024519.fna	5933	5392
SRS024690.SRX021470.V35	368533040	700102047	700102070	SRX021470	SRR052699	SRS024690	SRS024690	male	Saliva	Oral	2682842	SRS024690.fna	4418	3638
SRS043192.SRX020685.V35	160178356	700109567	700109590	SRX020685	SRR048537	SRS043192	SRS043192	male	Saliva	Oral	2577442	SRS043192.fna	4201	3641
SRS044606.SRX020580.V35	863126187	700103564	700103587	SRX020580	SRR047191	SRS044606	SRS044606	male	Saliva	Oral	1141717	SRS044606.fna	1843	1002
SRS044938.SRX020533.V35	147406386	700106931	700106948	SRX020533	SRR046802	SRS044938	SRS044938	male	Saliva	Oral	2382837	SRS044938.fna	3904	3711
SRS045138.SRX020688.V35	160400887	700107763	700107786	SRX020688	SRR048739	SRS045138	SRS045138	male	Saliva	Oral	6381542	SRS045138.fna	10317	7883
SRS045314.SRX020531.V35	370027359	700038957	700038980	SRX020531	SRR046031	SRS045314	SRS045314	male	Saliva	Oral	6509039	SRS045314.fna	10583	8322
SRS046488.SRX020685.V35	160056975	700107934	700107957	SRX020685	SRR048525	SRS046488	SRS046488	male	Saliva	Oral	3067923	SRS046488.fna	5000	4424
SRS046893.SRX022236.V35	160967330	700108477	700108501	SRX022236	SRR058113	SRS046893	SRS046893	male	Saliva	Oral	9054807	SRS046893.fna	14750	10541
SRS047094.SRX020529.V35	861967750	700038855	700038872	SRX020529	SRR049438	SRS047094	SRS047094	male	Saliva	Oral	2287872	SRS047094.fna	3688	3476
SRS047701.SRX022036.V35	160987560	700108786	700108810	SRX022036	SRR057609	SRS047701	SRS047701	male	Saliva	Oral	1219860	SRS047701.fna	1998	1487
SRS048043.SRX020684.V35	159288231	700102852	700102875	SRX020684	SRR048453	SRS048043	SRS048043	male	Saliva	Oral	3586778	SRS048043.fna	5832	5452
SRS048044.SRX020685.V35	160218816	700108900	700108923	SRX020685	SRR048550	SRS048044	SRS048044	male	Saliva	Oral	5230798	SRS048044.fna	8571	7340
SRS049063.SRX019691.V35	159490532	700102360	700102383	SRX019691	SRR041637	SRS049063	SRS049063	male	Saliva	Oral	3034242	SRS049063.fna	4947	4233
SRS049248.SRX022036.V35	161311242	700107820	700107844	SRX022036	SRR057609	SRS049248	SRS049248	male	Saliva	Oral	3234235	SRS049248.fna	5277	4118
SRS050871.SRX022222.V35	686765762	700107193	700107216	SRX022222	SRR058085	SRS050871	SRS050871	male	Saliva	Oral	1805540	SRS050871.fna	2951	2438
SRS052982.SRX020686.V35	160421117	700108165	700108188	SRX020686	SRR048604	SRS052982	SRS052982	male	Saliva	Oral	3209535	SRS052982.fna	5233	4683
SRS053635.SRX020533.V35	668248235	700105883	700105906	SRX020533	SRR046667	SRS053635	SRS053635	male	Saliva	Oral	2279904	SRS053635.fna	3709	3519
SRS055003.SRX020533.V35	517810313	700106397	700106414	SRX020533	SRR046681	SRS055003	SRS055003	male	Saliva	Oral	3814887	SRS055003.fna	6198	5843
SRS055295.SRX020687.V35	160765029	700109177	700109200	SRX020687	SRR048698	SRS055295	SRS055295	male	Saliva	Oral	7252917	SRS055295.fna	11828	10170
SRS055847.SRX020687.V35	160845950	700109510	700109533	SRX020687	SRR048686	SRS055847	SRS055847	male	Saliva	Oral	6926262	SRS055847.fna	11304	10073
SRS056331.SRX020688.V35	159632143	700107493	700107516	SRX020688	SRR048717	SRS056331	SRS056331	male	Saliva	Oral	6383989	SRS056331.fna	10452	7354
SRS056362.SRX020685.V35	160380657	700107877	700107900	SRX020685	SRR048580	SRS056362	SRS056362	male	Saliva	Oral	3947291	SRS056362.fna	6416	5209
SRS056421.SRX021470.V35	604812005	700102303	700102326	SRX021470	SRR052699	SRS056421	SRS056421	male	Saliva	Oral	4414021	SRS056421.fna	7196	6718
SRS056509.SRX021470.V35	840279516	700102663	700102686	SRX021470	SRR052699	SRS056509	SRS056509	male	Saliva	Oral	3128637	SRS056509.fna	5146	4727
SRS056606.SRX022036.V35	160947100	700109811	700109835	SRX022036	SRR057609	SRS056606	SRS056606	male	Saliva	Oral	2553797	SRS056606.fna	4170	3220
SRS057473.SRX020529.V35	765701615	700038393	700038416	SRX020529	SRR046522	SRS057473	SRS057473	male	Saliva	Oral	3652076	SRS057473.fna	5979	5701
SRS058293.SRX020688.V35	160097436	700107550	700107573	SRX020688	SRR048725	SRS058293	SRS058293	male	Saliva	Oral	6006136	SRS058293.fna	9787	7160
SRS058543.SRX022036.V35	161412393	700109991	700110015	SRX022036	SRR057609	SRS058543	SRS058543	male	Saliva	Oral	5869609	SRS058543.fna	9458	4882
SRS062410.SRX020579.V35	764953101	700114372	700114389	SRX020579	SRR047092	SRS062410	SRS062410	male	Saliva	Oral	3676951	SRS062410.fna	6069	5210
SRS062500.SRX020579.V35	764831721	700114707	700114729	SRX020579	SRR047078	SRS062500	SRS062500	male	Saliva	Oral	2366130	SRS062500.fna	3867	3349
SRS063416.SRX022222.V35	160825720	700108288	700111365	SRX022222	SRR058085	SRS063416	SRS063416	male	Saliva	Oral	1982912	SRS063416.fna	3221	2613
SRS063965.SRX020548.V35	764750800	700113014	700113037	SRX020548	SRR050112	SRS063965	SRS063965	male	Saliva	Oral	3167852	SRS063965.fna	5183	4833
SRS065156.SRX020665.V35	763395383	700114001	700114019	SRX020665	SRR047674	SRS065156	SRS065156	male	Saliva	Oral	5185781	SRS065156.fna	8507	7851
SRS065424.SRX020548.V35	289996019	700114168	700114191	SRX020548	SRR047348	SRS065424	SRS065424	male	Saliva	Oral	2730422	SRS065424.fna	4460	4084
SRS065675.SRX020548.V35	764872181	700113056	700113073	SRX020548	SRR047337	SRS065675	SRS065675	male	Saliva	Oral	3480271	SRS065675.fna	5666	5353
SRS011405.SRX020666.V35	159247771	700015979	700015981	SRX020666	SRR044948	SRS011405	SRS011405	female	Stool	Gastrointestinal_tract	3748103	SRS011405.fna	6198	3569
SRS011413.SRX020666.V35	159005010	700016004	700016021	SRX020666	SRR049235	SRS011413	SRS011413	female	Stool	Gastrointestinal_tract	5939189	SRS011413.fna	9755	5781
SRS011415.SRX020666.V35	159085930	700016008	700016029	SRX020666	SRR044930	SRS011415	SRS011415	female	Stool	Gastrointestinal_tract	4675859	SRS011415.fna	7602	5444
SRS011586.SRX020666.V35	159733294	700033130	700033153	SRX020666	SRR044971	SRS011586	SRS011586	female	Stool	Gastrointestinal_tract	5860237	SRS011586.fna	9583	5541
SRS011621.SRX020666.V35	159470302	700033436	700033438	SRX020666	SRR044922	SRS011621	SRS011621	female	Stool	Gastrointestinal_tract	5666715	SRS011621.fna	9375	5609
SRS011653.SRX020666.V35	159713063	700033726	700033749	SRX020666	SRR044915	SRS011653	SRS011653	female	Stool	Gastrointestinal_tract	4931818	SRS011653.fna	8120	4536
SRS012191.SRX020679.V35	158013734	700013549	700013596	SRX020679	SRR048044	SRS012191	SRS012191	female	Stool	Gastrointestinal_tract	4563870	SRS012191.fna	7558	5253
SRS013543.SRX020681.V35	159227541	700016542	700016564	SRX020681	SRR048260	SRS013543	SRS013543	female	Stool	Gastrointestinal_tract	2773165	SRS013543.fna	4618	3366
SRS014572.SRX020555.V35	763638144	700023188	700023226	SRX020555	SRR045726	SRS014572	SRS014572	female	Stool	Gastrointestinal_tract	2515895	SRS014572.fna	4201	3688
SRS014823.SRX020555.V35	763921366	700023447	700023477	SRX020555	SRR045736	SRS014823	SRS014823	female	Stool	Gastrointestinal_tract	5430118	SRS014823.fna	8866	4781
SRS015133.SRX020555.V35	763901136	700023701	700023788	SRX020555	SRR045722	SRS015133	SRS015133	female	Stool	Gastrointestinal_tract	5366719	SRS015133.fna	8739	5777
SRS015190.SRX020558.V35	763759525	700023815	700023845	SRX020558	SRR047400	SRS015190	SRS015190	female	Stool	Gastrointestinal_tract	5391757	SRS015190.fna	8980	6777
SRS015247.SRX020535.V35	764143897	700023872	700023902	SRX020535	SRR044113	SRS015247	SRS015247	female	Stool	Gastrointestinal_tract	3477953	SRS015247.fna	5701	4479
SRS015724.SRX020573.V35	764467579	700024349	700024379	SRX020573	SRR044209	SRS015724	SRS015724	female	Stool	Gastrointestinal_tract	6045494	SRS015724.fna	9939	7199
SRS016152.SRX020573.V35	764366428	700024789	700024809	SRX020573	SRR044258	SRS016152	SRS016152	female	Stool	Gastrointestinal_tract	6335510	SRS016152.fna	10461	7937
SRS016203.SRX020522.V35	764649650	700024846	700024866	SRX020522	SRR044423	SRS016203	SRS016203	female	Stool	Gastrointestinal_tract	4526830	SRS016203.fna	7527	5303
SRS016630.SRX019691.V35	160036745	700032413	700032414	SRX019691	SRR041505	SRS016630	SRS016630	female	Stool	Gastrointestinal_tract	3019835	SRS016630.fna	4960	3604
SRS018068.SRX019682.V35	160016515	700035369	700035392	SRX019682	SRR040884	SRS018068	SRS018068	female	Stool	Gastrointestinal_tract	2229589	SRS018068.fna	3661	2867
SRS018250.SRX019687.V35	160137896	700035635	700035657	SRX019687	SRR041356	SRS018250	SRS018250	female	Stool	Gastrointestinal_tract	3042261	SRS018250.fna	4980	4045
SRS018489.SRX019687.V35	160259276	700035931	700035953	SRX019687	SRR041239	SRS018489	SRS018489	female	Stool	Gastrointestinal_tract	3123514	SRS018489.fna	5082	4362
SRS018733.SRX020522.V35	765013792	700037123	700037200	SRX020522	SRR047434	SRS018733	SRS018733	female	Stool	Gastrointestinal_tract	5408731	SRS018733.fna	8741	7227
SRS019267.SRX020522.V35	765034022	700037700	700037738	SRX020522	SRR044439	SRS019267	SRS019267	female	Stool	Gastrointestinal_tract	4808154	SRS019267.fna	8003	6249
SRS021109.SRX022230.V35	160744799	700096590	700096656	SRX022230	SRR058093	SRS021109	SRS021109	female	Stool	Gastrointestinal_tract	1390282	SRS021109.fna	2294	1456
SRS021175.SRX022239.V35	161007791	700096700	700096722	SRX022239	SRR058109	SRS021175	SRS021175	female	Stool	Gastrointestinal_tract	2169287	SRS021175.fna	3586	2810
SRS021241.SRX022239.V35	161230322	700096865	700096887	SRX022239	SRR058109	SRS021241	SRS021241	female	Stool	Gastrointestinal_tract	2295659	SRS021241.fna	3817	2548
SRS021364.SRX022239.V35	161028021	700097000	700097022	SRX022239	SRR058109	SRS021364	SRS021364	female	Stool	Gastrointestinal_tract	2212201	SRS021364.fna	3626	2706
SRS021724.SRX022231.V35	161493313	700097415	700097437	SRX022231	SRR058120	SRS021724	SRS021724	female	Stool	Gastrointestinal_tract	1993049	SRS021724.fna	3284	2527
SRS021790.SRX022235.V35	161473083	700097481	700097503	SRX022235	SRR058095	SRS021790	SRS021790	female	Stool	Gastrointestinal_tract	2074485	SRS021790.fna	3399	2187
SRS021970.SRX019689.V35	158256496	700097688	700097710	SRX019689	SRR041390	SRS021970	SRS021970	female	Stool	Gastrointestinal_tract	5972578	SRS021970.fna	9743	4096
SRS022093.SRX019690.V35	158337416	700097837	700097859	SRX019690	SRR041491	SRS022093	SRS022093	female	Stool	Gastrointestinal_tract	2854487	SRS022093.fna	4709	3429
SRS022159.SRX022231.V35	638754422	700097906	700097928	SRX022231	SRR058120	SRS022159	SRS022159	female	Stool	Gastrointestinal_tract	2261198	SRS022159.fna	3735	2944
SRS022291.SRX022231.V35	533247696	700098109	700098131	SRX022231	SRR058120	SRS022291	SRS022291	female	Stool	Gastrointestinal_tract	2314285	SRS022291.fna	3833	2948
SRS022414.SRX020680.V35	158742018	700098267	700098289	SRX020680	SRR048189	SRS022414	SRS022414	female	Stool	Gastrointestinal_tract	3867605	SRS022414.fna	6383	5218
SRS022480.SRX020680.V35	158458797	700098359	700098381	SRX020680	SRR048171	SRS022480	SRS022480	female	Stool	Gastrointestinal_tract	4118503	SRS022480.fna	6773	4992
SRS022546.SRX022226.V35	809635352	700098429	700098451	SRX022226	SRR058089	SRS022546	SRS022546	female	Stool	Gastrointestinal_tract	2280748	SRS022546.fna	3743	3003
SRS022669.SRX022226.V35	432193348	700098599	700098621	SRX022226	SRR058089	SRS022669	SRS022669	female	Stool	Gastrointestinal_tract	1826144	SRS022669.fna	3016	2459
SRS022735.SRX022231.V35	370425937	700098669	700098691	SRX022231	SRR058120	SRS022735	SRS022735	female	Stool	Gastrointestinal_tract	2522849	SRS022735.fna	4170	3220
SRS022858.SRX022226.V35	650853796	700098866	700098888	SRX022226	SRR058089	SRS022858	SRS022858	female	Stool	Gastrointestinal_tract	2038835	SRS022858.fna	3378	2620
SRS022924.SRX020679.V35	158236265	700098932	700098954	SRX020679	SRR048095	SRS022924	SRS022924	female	Stool	Gastrointestinal_tract	4380723	SRS022924.fna	7259	4534
SRS022987.SRX020679.V35	158114885	700099002	700099021	SRX020679	SRR048059	SRS022987	SRS022987	female	Stool	Gastrointestinal_tract	4468037	SRS022987.fna	7435	5491
SRS023047.SRX020679.V35	158276726	700099059	700099081	SRX020679	SRR048110	SRS023047	SRS023047	female	Stool	Gastrointestinal_tract	3339705	SRS023047.fna	5493	3589
SRS023106.SRX022226.V35	441369442	700099125	700099237	SRX022226	SRR058089	SRS023106	SRS023106	female	Stool	Gastrointestinal_tract	2654477	SRS023106.fna	4404	3184
SRS023302.SRX022223.V35	206906765	700099442	700099464	SRX022223	SRR058086	SRS023302	SRS023302	female	Stool	Gastrointestinal_tract	1890051	SRS023302.fna	3152	2745
SRS023365.SRX022223.V35	246515023	700099512	700099531	SRX022223	SRR058086	SRS023365	SRS023365	female	Stool	Gastrointestinal_tract	2545215	SRS023365.fna	4217	3635
SRS023851.SRX020680.V35	158883629	700100227	700100249	SRX020680	SRR048207	SRS023851	SRS023851	female	Stool	Gastrointestinal_tract	4028909	SRS023851.fna	6657	5364
SRS024265.SRX019692.V35	158944319	700101224	700101243	SRX019692	SRR041911	SRS024265	SRS024265	female	Stool	Gastrointestinal_tract	6016832	SRS024265.fna	9858	8082

SRS044146.SRX021470.V35	937415040	700102519	700102541	SRX021470	SRR052699	SRS044146	SRS044146	female	Stool	Gastrointestinal_tract	2888312	SRS044146.fna	4725	3809
SRS045414.SRX020518.V35	737052003	700106065	700106093	SRX020518	SRR049611	SRS045414	SRS045414	female	Stool	Gastrointestinal_tract	6983254	SRS045414.fna	11439	8109
SRS045493.SRX020685.V35	160319967	700109621	700109643	SRX020685	SRR048558	SRS045493	SRS045493	female	Stool	Gastrointestinal_tract	4260641	SRS045493.fna	7011	4869
SRS045526.SRX020665.V35	763456073	700105685	700105723	SRX020665	SRR046319	SRS045526	SRS045526	female	Stool	Gastrointestinal_tract	5846305	SRS045526.fna	9696	6400
SRS045613.SRX020523.V35	553359145	700106504	700106542	SRX020523	SRR046419	SRS045613	SRS045613	female	Stool	Gastrointestinal_tract	1099528	SRS045613.fna	1821	520
SRS047561.SRX020686.V35	160582958	700108530	700108552	SRX020686	SRR048629	SRS047561	SRS047561	female	Stool	Gastrointestinal_tract	3576232	SRS047561.fna	5907	4400
SRS048008.SRX021470.V35	514014184	700101771	700101793	SRX021470	SRR052699	SRS048008	SRS048008	female	Stool	Gastrointestinal_tract	2681233	SRS048008.fna	4439	3931
SRS048722.SRX020523.V35	208027353	700106551	700106579	SRX020523	SRR046409	SRS048722	SRS048722	female	Stool	Gastrointestinal_tract	1102061	SRS048722.fna	1801	969
SRS049157.SRX021470.V35	414519462	700102113	700102135	SRX021470	SRR052699	SRS049157	SRS049157	female	Stool	Gastrointestinal_tract	3021002	SRS049157.fna	5007	3719
SRS049982.SRX020686.V35	160643649	700110089	700110111	SRX020686	SRR048657	SRS049982	SRS049982	female	Stool	Gastrointestinal_tract	4077885	SRS049982.fna	6668	4944
SRS050141.SRX020688.V35	160704339	700108218	700108240	SRX020688	SRR048760	SRS050141	SRS050141	female	Stool	Gastrointestinal_tract	8412953	SRS050141.fna	13631	8335
SRS050374.SRX022227.V35	970836795	700109107	700109129	SRX022227	SRR058090	SRS050374	SRS050374	female	Stool	Gastrointestinal_tract	6545458	SRS050374.fna	10738	4636
SRS052326.SRX020665.V35	763820215	700105210	700105238	SRX020665	SRR046272	SRS052326	SRS052326	female	Stool	Gastrointestinal_tract	5496291	SRS052326.fna	9150	6910
SRS055482.SRX020514.V35	612472597	700105153	700105181	SRX020514	SRR049504	SRS055482	SRS055482	female	Stool	Gastrointestinal_tract	7605522	SRS055482.fna	12457	9778
SRS055934.SRX020684.V35	159915365	700108596	700108618	SRX020684	SRR048492	SRS055934	SRS055934	female	Stool	Gastrointestinal_tract	6056400	SRS055934.fna	9899	7741
SRS056255.SRX020686.V35	160502038	700108095	700108117	SRX020686	SRR048611	SRS056255	SRS056255	female	Stool	Gastrointestinal_tract	2093487	SRS056255.fna	3463	2889
SRS056505.SRX020684.V35	159753524	700107375	700107397	SRX020684	SRR048477	SRS056505	SRS056505	female	Stool	Gastrointestinal_tract	10366820	SRS056505.fna	17119	13609
SRS057258.SRX022221.V35	160866180	700109317	700109340	SRX022221	SRR058084	SRS057258	SRS057258	female	Stool	Gastrointestinal_tract	13312698	SRS057258.fna	21896	18017
SRS062610.SRX020689.V35	763721271	700111296	700111318	SRX020689	SRR048832	SRS062610	SRS062610	female	Stool	Gastrointestinal_tract	4723783	SRS062610.fna	7706	5090
SRS063068.SRX020689.V35	866653606	700111222	700111244	SRX020689	SRR048796	SRS063068	SRS063068	female	Stool	Gastrointestinal_tract	3920196	SRS063068.fna	6467	4573
SRS063138.SRX020689.V35	257905678	700111156	700111178	SRX020689	SRR048778	SRS063138	SRS063138	female	Stool	Gastrointestinal_tract	6532145	SRS063138.fna	10780	6670
SRS063275.SRX020579.V35	375450439	700114798	700114818	SRX020579	SRR049559	SRS063275	SRS063275	female	Stool	Gastrointestinal_tract	4047841	SRS063275.fna	6704	4986
SRS063307.SRX022237.V35	937495960	700111574	700111596	SRX022237	SRR058114	SRS063307	SRS063307	female	Stool	Gastrointestinal_tract	1381196	SRS063307.fna	2265	1235
SRS063524.SRX020689.V35	872848689	700111745	700111767	SRX020689	SRR048814	SRS063524	SRS063524	female	Stool	Gastrointestinal_tract	3532802	SRS063524.fna	5823	3400
SRS063921.SRX020528.V35	763719065	700113503	700113541	SRX020528	SRR046760	SRS063921	SRS063921	female	Stool	Gastrointestinal_tract	4821243	SRS063921.fna	8001	6159
SRS064276.SRX020665.V35	763840445	700114105	700114125	SRX020665	SRR046311	SRS064276	SRS064276	female	Stool	Gastrointestinal_tract	7146490	SRS064276.fna	11763	7969
SRS065466.SRX020579.V35	663835652	700114218	700114246	SRX020579	SRR049589	SRS065466	SRS065466	female	Stool	Gastrointestinal_tract	5533507	SRS065466.fna	9137	5866
SRS065500.SRX022221.V35	892969023	700110287	700110309	SRX022221	SRR058084	SRS065500	SRS065500	female	Stool	Gastrointestinal_tract	11484733	SRS065500.fna	18934	14889
SRS011157.SRX020666.V35	158822939	700014954	700014956	SRX020666	SRR044955	SRS011157	SRS011157	male	Stool	Gastrointestinal_tract	6194163	SRS011157.fna	10235	6199
SRS011271.SRX020666.V35	158802708	700015245	700015250	SRX020666	SRR044974	SRS011271	SRS011271	male	Stool	Gastrointestinal_tract	5763680	SRS011271.fna	9573	5492
SRS011452.SRX020666.V35	159146620	700016136	700016142	SRX020666	SRR044944	SRS011452	SRS011452	male	Stool	Gastrointestinal_tract	3800609	SRS011452.fna	6275	3531
SRS011529.SRX020666.V35	159166850	700016608	700016610	SRX020666	SRR044912	SRS011529	SRS011529	male	Stool	Gastrointestinal_tract	4982557	SRS011529.fna	8251	4781
SRS013177.SRX019681.V35	159369152	700015857	700015876	SRX019681	SRR040857	SRS013177	SRS013177	male	Stool	Gastrointestinal_tract	1665233	SRS013177.fna	2779	2114
SRS013216.SRX019639.V35	159591683	700015922	700015923	SRX019639	SRR040793	SRS013216	SRS013216	male	Stool	Gastrointestinal_tract	1857637	SRS013216.fna	3068	1908
SRS013386.SRX019692.V35	159328691	700016210	700016214	SRX019692	SRR041993	SRS013386	SRS013386	male	Stool	Gastrointestinal_tract	5898089	SRS013386.fna	9694	7652
SRS013490.SRX019683.V35	159207311	700016456	700016470	SRX019683	SRR041145	SRS013490	SRS013490	male	Stool	Gastrointestinal_tract	2322798	SRS013490.fna	3806	2897
SRS013687.SRX019690.V35	159268001	700016764	700016765	SRX019690	SRR041527	SRS013687	SRS013687	male	Stool	Gastrointestinal_tract	3265298	SRS013687.fna	5381	4314
SRS014885.SRX020558.V35	764002286	700023509	700023539	SRX020558	SRR049220	SRS014885	SRS014885	male	Stool	Gastrointestinal_tract	9941027	SRS014885.fna	16292	11651
SRS014978.SRX020530.V35	763880905	700023603	700023633	SRX020530	SRR045796	SRS014978	SRS014978	male	Stool	Gastrointestinal_tract	11261118	SRS014978.fna	18430	15596
SRS015281.SRX020558.V35	763982056	700023919	700023936	SRX020558	SRR047403	SRS015281	SRS015281	male	Stool	Gastrointestinal_tract	6131507	SRS015281.fna	10085	7540
SRS015452.SRX020538.V35	764083206	700024086	700024107	SRX020538	SRR044648	SRS015452	SRS015452	male	Stool	Gastrointestinal_tract	11516155	SRS015452.fna	18945	12902
SRS015578.SRX020535.V35	764285508	700024201	700024233	SRX020535	SRR043983	SRS015578	SRS015578	male	Stool	Gastrointestinal_tract	4214870	SRS015578.fna	6922	5188
SRS015782.SRX020573.V35	764224817	700024408	700024437	SRX020573	SRR044216	SRS015782	SRS015782	male	Stool	Gastrointestinal_tract	6382968	SRS015782.fna	10530	7211
SRS015815.SRX020562.V35	763435843	700024449	700024470	SRX020562	SRR044705	SRS015815	SRS015815	male	Stool	Gastrointestinal_tract	11996262	SRS015815.fna	19723	14277
SRS016018.SRX020573.V35	764447348	700024646	700024673	SRX020573	SRR044251	SRS016018	SRS016018	male	Stool	Gastrointestinal_tract	4537699	SRS016018.fna	7503	5873
SRS016267.SRX020535.V35	764669880	700024908	700024930	SRX020535	SRR044091	SRS016267	SRS016267	male	Stool	Gastrointestinal_tract	2686254	SRS016267.fna	4508	3338
SRS016400.SRX019688.V35	159672603	700032068	700032087	SRX019688	SRR041382	SRS016400	SRS016400	male	Stool	Gastrointestinal_tract	2083431	SRS016400.fna	3399	1663
SRS016437.SRX019681.V35	159429842	700032132	700032133	SRX019681	SRR040966	SRS016437	SRS016437	male	Stool	Gastrointestinal_tract	1905138	SRS016437.fna	3157	2368
SRS016841.SRX019692.V35	159308461	700033201	700033202	SRX019692	SRR041889	SRS016841	SRS016841	male	Stool	Gastrointestinal_tract	6758749	SRS016841.fna	11092	8762
SRS016916.SRX019681.V35	159450072	700033363	700033382	SRX019681	SRR040953	SRS016916	SRS016916	male	Stool	Gastrointestinal_tract	1762169	SRS016916.fna	2923	2152
SRS016954.SRX019687.V35	159611913	700033559	700033435	SRX019687	SRR041333	SRS016954	SRS016954	male	Stool	Gastrointestinal_tract	3224177	SRS016954.fna	5283	3644
SRS017103.SRX019684.V35	159551223	700033664	700033665	SRX019684	SRR041032	SRS017103	SRS017103	male	Stool	Gastrointestinal_tract	3232125	SRS017103.fna	5321	3467
SRS017191.SRX019688.V35	159814214	700033796	700033797	SRX019688	SRR041435	SRS017191	SRS017191	male	Stool	Gastrointestinal_tract	2530015	SRS017191.fna	4118	1791
SRS017394.SRX019683.V35	160481808	700034081	700034100	SRX019683	SRR041039	SRS017394	SRS017394	male	Stool	Gastrointestinal_tract	1791196	SRS017394.fna	2960	2069
SRS017641.SRX019683.V35	160461578	700034622	700034641	SRX019683	SRR041130	SRS017641	SRS017641	male	Stool	Gastrointestinal_tract	2116571	SRS017641.fna	3496	2627
SRS017764.SRX019639.V35	160542498	700034838	700034857	SRX019639	SRR040696	SRS017764	SRS017764	male	Stool	Gastrointestinal_tract	2290655	SRS017764.fna	3738	2657
SRS017821.SRX019686.V35	160603188	700034907	700034926	SRX019686	SRR041310	SRS017821	SRS017821	male	Stool	Gastrointestinal_tract	4041968	SRS017821.fna	6623	5142
SRS017878.SRX019688.V35	160441347	700035157	700035176	SRX019688	SRR041476	SRS017878	SRS017878	male	Stool	Gastrointestinal_tract	2435422	SRS017878.fna	3976	1672
SRS017935.SRX019692.V35	158357646	700035237	700035256	SRX019692	SRR041737	SRS017935	SRS017935	male	Stool	Gastrointestinal_tract	5204480	SRS017935.fna	8548	7173
SRS017997.SRX019639.V35	160239046	700035297	700035321	SRX019639	SRR040698	SRS017997	SRS017997	male	Stool	Gastrointestinal_tract	1908394	SRS017997.fna	3138	2796
SRS018133.SRX019682.V35	160158126	700035514	700035533	SRX019682	SRR040925	SRS018133	SRS018133	male	Stool	Gastrointestinal_tract	2623021	SRS018133.fna	4315	3466
SRS018427.SRX019684.V35	160218816	700035842	700035861	SRX019684	SRR041118	SRS018427	SRS018427	male	Stool	Gastrointestinal_tract	3298965	SRS018427.fna	5375	3968
SRS018607.SRX020530.V35	765094712	700037042	700037074	SRX020530	SRR045872	SRS018607	SRS018607	male	Stool	Gastrointestinal_tract	3967159	SRS018607.fna	6613	4606
SRS018817.SRX020530.V35	765135172	700037252	700037284	SRX020530	SRR045867	SRS018817	SRS018817	male	Stool	Gastrointestinal_tract	3747355	SRS018817.fna	6205	4609
SRS019013.SRX020510.V35	764305738	700037453	700037484	SRX020510	SRR044597	SRS019013	SRS019013	male	Stool	Gastrointestinal_tract	5290670	SRS019013.fna	8816	6360
SRS019910.SRX020522.V35	764892411	700038354	700038386	SRX020522	SRR044428	SRS019910	SRS019910	male	Stool	Gastrointestinal_tract	5610920	SRS019910.fna	9249	6803
SRS020119.SRX020680.V35	158398106	700095353	700095372	SRX020680	SRR048128	SRS020119	SRS020119	male	Stool	Gastrointestinal_tract	3975099	SRS020119.fna	6598	4982
SRS020176.SRX020680.V35	158418336	700095410	700095429	SRX020680	SRR048143	SRS020176	SRS020176	male	Stool	Gastrointestinal_tract	5558802	SRS020176.fna	9212	6587
SRS020290.SRX019684.V35	160663879	700095524	700095543	SRX019684	SRR041047	SRS020290	SRS020290	male	Stool	Gastrointestinal_tract	2962549	SRS020290.fna	4868	3688
SRS020413.SRX019682.V35	160684109	700095717	700095736	SRX019682	SRR040994	SRS020413	SRS020413	male	Stool	Gastrointestinal_tract	2654678	SRS020413.fna	4349	2781
SRS020584.SRX022230.V35	160906640	700095956	700095975	SRX022230	SRR058093	SRS020584	SRS020584	male	Stool	Gastrointestinal_tract	1681707	SRS020584.fna	2752	1815
SRS020811.SRX022230.V35	160886410	700096267	700096285	SRX022230	SRR058093	SRS020811	SRS020811	male	Stool	Gastrointestinal_tract	2473151	SRS020811.fna	4092	2941
SRS020926.SRX022239.V35	161068481	700096418	700096437	SRX022239	SRR058109	SRS020926	SRS020926	male	Stool	Gastrointestinal_tract	2292986	SRS020926.fna	3778	2879
SRS021304.SRX022235.V35	161351702	700096943	700096962	SRX022235	SRR058095	SRS021304	SRS021304	male	Stool	Gastrointestinal_tract	2034999	SRS021304.fna	3357	2064
SRS021484.SRX019690.V35	158479027	700097177	700097196	SRX019690	SRR041529	SRS021484	SRS021484	male	Stool	Gastrointestinal_tract	2616867	SRS021484.fna	4336	3621
SRS021541.SRX020679.V35	158216035	700097234	700097253	SRX020679	SRR048083	SRS021541	SRS021541	male	Stool	Gastrointestinal_tract	3176762	SRS021541.fna	5268	3455
SRS021664.SRX022235.V35	161331472	700097357	700097376	SRX022235	SRR058095	SRS021664	SRS021664	male	Stool	Gastrointestinal_tract	1812158	SRS021664.fna	2995	1981
SRS021853.SRX022235.V35	161270782	700097570	700097589	SRX022235	SRR058095	SRS021853	SRS021853	male	Stool	Gastrointestinal_tract	2842212	SRS021853.fna	4670	2882
SRS021910.SRX020679.V35	158155345	700097631	700097650	SRX020679	SRR048072	SRS021910	SRS021910	male	Stool	Gastrointestinal_tract	4325798	SRS021910.fna	7074	3945
SRS022033.SRX022231.V35	161554003	700097754	700097773	SRX022231	SRR058120	SRS022033	SRS022033	male	Stool	Gastrointestinal_tract	2381877	SRS022033.fna	3885	2712
SRS022354.SRX022226.V35	492786515	700098201	700098220	SRX022226	SRR058089	SRS022354	SRS022354	male	Stool	Gastrointestinal_tract	2009865	SRS022354.fna	3318	2662
SRS022609.SRX019692.V35	158499257	700098542	700098561	SRX019692	SRR041777	SRS022609	SRS022609	male	Stool	Gastrointestinal_tract	8143588	SRS022609.fna	13412	10060
SRS022798.SRX022223.V35	295137534	700098735	700098754	SRX022223	SRR058086	SRS022798	SRS022798	male	Stool	Gastrointestinal_tract	1928490	SRS022798.fna	3191	2731
SRS023176.SRX019692.V35	158924089	700099288	700099307	SRX019692	SRR041890	SRS023176	SRS023176	male	Stool	Gastrointestinal_tract	4915596	SRS023176.fna	8158	6675
SRS023242.SRX022223.V35	964271349	700099381	700099400	SRX022223	SRR058086	SRS023242	SRS023242	male	Stool	Gastrointestinal_tract	3505425	SRS023242.fna	5849	5122
SRS023422.SRX020680.V35	158438567	700099590	700099609	SRX020680	SRR048157	SRS023422	SRS023422	male	Stool	Gastrointestinal_tract	4149887	SRS023422.fna	6914	6291
SRS023488.SRX020681.V35	158964549	700099746	700099765	SRX020681	SRR048223	SRS023488	SRS023488	male	Stool	Gastrointestinal_tract	6029889	SRS023488.fna	10018	7690
SRS023734.SRX019681.V35	158721788	700100022	700100041	SRX019681	SRR040976	SRS023734	SRS023734	male	Stool	Gastrointestinal_tract	2521788	SRS023734.fna	4176	3036
SRS023791.SRX022223.V35	336497421	700100165	700100184	SRX022223	SRR058086	SRS023791	SRS023791	male	Stool	Gastrointestinal_tract	3296614	SRS023791.fna	5494	4920
SRS024454.SRX019684.V35	159571453	700101581	700101600	SRX019684	SRR041114	SRS024454	SRS024454	male	Stool	Gastrointestinal_tract	3209874	SRS024454.fna	5251	3558
SRS024511.SRX020680.V35	159389382	700101638	700101657	SRX020680	SRR048158	SRS024511	SRS024511	male	Stool	Gastrointestinal_tract	3418101	SRS024511.fna	5684	5051
SRS024682.SRX021470.V35	368533040	700102043	700102062	SRX021470	SRR052699	SRS024682	SRS024682	male	Stool	Gastrointestinal_tract	2569192	SRS024682.fna	4263	3540
SRS042387.SRX020523.V35	863126187	700103558	700103575	SRX020523	SRR046416	SRS042387	SRS042387	male	Stool	Gastrointestinal_tract	1266627	SRS042387.fna	2112	537
SRS042415.SRX020523.V35	517810313	700106412	700106930	SRX020523	SRR046407	SRS042415	SRS042415	male	Stool	Gastrointestinal_tract	1195798	SRS042415.fna	1977	684
SRS042843.SRX020685.V35	160097436	700107546	700107565	SRX020685	SRR048509	SRS042843	SRS042843	male	Stool	Gastrointestinal_tract	4247262	SRS042843.fna	7060	4804
SRS043769.SRX022036.V35	160987560	700108782	700108802	SRX022036	SRR057609	SRS043769	SRS043769	male	Stool	Gastrointestinal_tract	5445485	SRS043769.fna	8984	2757
SRS044088.SRX022036.V35	161412393	700109987	700110007	SRX022036	SRR057609	SRS044088	SRS044088	male	Stool	Gastrointestinal_tract	6489757	SRS044088.fna	10658	4343
SRS045350.SRX020688.V35	160421117	700108161	700108180	SRX020688	SRR048744	SRS045350	SRS045350	male	Stool	Gastrointestinal_tract	4661971	SRS045350.fna	7683	4610
SRS045627.SRX020685.V35	160056975	700107930	700107949	SRX020685	SRR048520	SRS045627	SRS045627	male	Stool	Gastrointestinal_tract	3434234	SRS045627.fna	5680	4600
SRS045910.SRX022221.V35	160825720	700108284	700108304	SRX022221	SRR058084	SRS045910	SRS045910	male	Stool	Gastrointestinal_tract	9482519	SRS045910.fna	15736	11484
SRS046216.SRX022036.V35	161311242	700107816	700107836	SRX022036	SRR057609	SRS046216	SRS046216	male	Stool	Gastrointestinal_tract	5627383	SRS046216.fna	9276	3493
SRS046349.SRX020531.V35	370027359	700038956	700038978	SRX020531	SRR046051	SRS046349	SRS046349	male	Stool	Gastrointestinal_tract	4330755	SRS046349.fna	7137	4615
SRS047642.SRX022222.V35	686765762	700107189	700107208	SRX022222	SRR058085	SRS047642	SRS047642	male	Stool	Gastrointestinal_tract	1503169	SRS047642.fna	2470	1374
SRS047967.SRX020687.V35	160765029	700109173	700109192	SRX020687	SRR048693	SRS047967	SRS047967	male	Stool	Gastrointestinal_tract	8086838	SRS047967.fna	13290	9507
SRS048299.SRX020685.V35	160380657	700107873	700107892	SRX020685	SRR048575	SRS048299	SRS048299	male	Stool	Gastrointestinal_tract	4276568	SRS048299.fna	7041	4993
SRS048853.SRX021470.V35	840279516	700102659	700102678	SRX021470	SRR052699	SRS048853	SRS048853	male	Stool	Gastrointestinal_tract	3293543	SRS048853.fna	5506	4556
SRS049949.SRX022036.V35	160967330	700108473	700108493	SRX022036	SRR057609	SRS049949	SRS049949	male	Stool	Gastrointestinal_tract	7097082	SRS049949.fna	11620	4427
SRS049959.SRX020530.V35	765701615	700038392	700038414	SRX020530	SRR045933	SRS049959	SRS049959	male	Stool	Gastrointestinal_tract	4193104	SRS049959.fna	6873	4723
SRS051031.SRX020542.V35	764487809	700105355	700105372	SRX020542	SRR046376	SRS051031	SRS051031	male	Stool	Gastrointestinal_tract	3717733	SRS051031.fna	6143	3273
SRS051086.SRX022221.V35	160947100	700109807	700109827	SRX022221	SRR058084	SRS051086	SRS051086	male	Stool	Gastrointestinal_tract	10006093	SRS051086.fna	16588	11535
SRS051609.SRX020686.V35	160400887	700107759	700107778	SRX020686	SRR048590	SRS051609	SRS051609	male	Stool	Gastrointestinal_tract	3919649	SRS051609.fna	6465	3917

SRS052471.SRX020530.V35	861967750	700038870	700038902	SRX020530	SRR045938	SRS052471	SRS052471	male	Stool	Gastrointestinal_tract	5847780	SRS052471.fna	9672	7011
SRS053301.SRX020687.V35	160845950	700109506	700109525	SRX020687	SRR048681	SRS053301	SRS053301	male	Stool	Gastrointestinal_tract	6490579	SRS053301.fna	10803	9033
SRS054488.SRX020684.V35	159632143	700107489	700107508	SRX020684	SRR048464	SRS054488	SRS054488	male	Stool	Gastrointestinal_tract	5671868	SRS054488.fna	9320	7300
SRS055137.SRX020685.V35	160178356	700109563	700109582	SRX020685	SRR048532	SRS055137	SRS055137	male	Stool	Gastrointestinal_tract	4843107	SRS055137.fna	7991	6270
SRS055697.SRX022221.V35	604812005	700110419	700110438	SRX022221	SRR058084	SRS055697	SRS055697	male	Stool	Gastrointestinal_tract	11892542	SRS055697.fna	19707	14294
SRS056656.SRX020684.V35	159288231	700102848	700102867	SRX020684	SRR048448	SRS056656	SRS056656	male	Stool	Gastrointestinal_tract	5275309	SRS056656.fna	8708	7524
SRS057447.SRX020523.V35	668248235	700105882	700105904	SRX020523	SRR046411	SRS057447	SRS057447	male	Stool	Gastrointestinal_tract	1404431	SRS057447.fna	2309	948
SRS057901.SRX019691.V35	159490532	700102356	700102375	SRX019691	SRR041646	SRS057901	SRS057901	male	Stool	Gastrointestinal_tract	3678672	SRS057901.fna	6093	3930
SRS058416.SRX020523.V35	147406386	700106946	700106978	SRX020523	SRR047701	SRS058416	SRS058416	male	Stool	Gastrointestinal_tract	1534940	SRS058416.fna	2531	738
SRS062464.SRX020579.V35	764750800	700113013	700113035	SRX020579	SRR047080	SRS062464	SRS062464	male	Stool	Gastrointestinal_tract	3143493	SRS062464.fna	5197	3437
SRS062847.SRX020579.V35	289996019	700114162	700114179	SRX020579	SRR047097	SRS062847	SRS062847	male	Stool	Gastrointestinal_tract	3981438	SRS062847.fna	6576	5064
SRS063214.SRX020579.V35	764953101	700114387	700114419	SRX020579	SRR047081	SRS063214	SRS063214	male	Stool	Gastrointestinal_tract	4099479	SRS063214.fna	6723	4551
SRS063797.SRX020689.V35	159510762	700111985	700112004	SRX020689	SRR048853	SRS063797	SRS063797	male	Stool	Gastrointestinal_tract	5639010	SRS063797.fna	9235	4902
SRS063827.SRX020579.V35	764872181	700113066	700113093	SRX020579	SRR049581	SRS063827	SRS063827	male	Stool	Gastrointestinal_tract	3904661	SRS063827.fna	6459	5169
SRS063961.SRX020579.V35	764831721	700114717	700114749	SRX020579	SRR046985	SRS063961	SRS063961	male	Stool	Gastrointestinal_tract	4201714	SRS063961.fna	6990	5229
SRS065263.SRX020665.V35	763395383	700114000	700114017	SRX020665	SRR046316	SRS065263	SRS065263	male	Stool	Gastrointestinal_tract	6020760	SRS065263.fna	10097	7170
SRS011446.SRX020660.V35	159005010	700016055	700016097	SRX020660	SRR044890	SRS011446	SRS011446	female	Vaginal_introitus	Urogenital_tract	5281931	SRS011446.fna	8939	7776
SRS011523.SRX020660.V35	159085930	700016498	700016502	SRX020660	SRR044873	SRS011523	SRS011523	female	Vaginal_introitus	Urogenital_tract	5599253	SRS011523.fna	9482	8122
SRS011580.SRX020666.V35	159247771	700033036	700033038	SRX020666	SRR044945	SRS011580	SRS011580	female	Vaginal_introitus	Urogenital_tract	2810199	SRS011580.fna	4682	3875
SRS011615.SRX020666.V35	159733294	700033149	700033191	SRX020666	SRR044978	SRS011615	SRS011615	female	Vaginal_introitus	Urogenital_tract	3910504	SRS011615.fna	6545	5588
SRS011682.SRX020666.V35	159713063	700033745	700033787	SRX020666	SRR044908	SRS011682	SRS011682	female	Vaginal_introitus	Urogenital_tract	3442862	SRS011682.fna	5720	4730
SRS013581.SRX020681.V35	159227541	700016561	700016602	SRX020681	SRR048272	SRS013581	SRS013581	female	Vaginal_introitus	Urogenital_tract	2926969	SRS013581.fna	4741	4188
SRS014546.SRX020555.V35	763638144	700023175	700023200	SRX020555	SRR045714	SRS014546	SRS014546	female	Vaginal_introitus	Urogenital_tract	2796449	SRS014546.fna	4771	4561
SRS014835.SRX020555.V35	763921366	700023453	700023489	SRX020555	SRR045701	SRS014835	SRS014835	female	Vaginal_introitus	Urogenital_tract	4450897	SRS014835.fna	7511	7094
SRS015145.SRX020555.V35	763901136	700023707	700023800	SRX020555	SRR045678	SRS015145	SRS015145	female	Vaginal_introitus	Urogenital_tract	4107164	SRS015145.fna	6977	6622
SRS015202.SRX020558.V35	763759525	700023821	700023857	SRX020558	SRR043787	SRS015202	SRS015202	female	Vaginal_introitus	Urogenital_tract	3771767	SRS015202.fna	6371	6172
SRS015259.SRX020535.V35	764143897	700023878	700023914	SRX020535	SRR044020	SRS015259	SRS015259	female	Vaginal_introitus	Urogenital_tract	2785445	SRS015259.fna	4726	4620
SRS015736.SRX020573.V35	764467579	700024355	700024391	SRX020573	SRR044271	SRS015736	SRS015736	female	Vaginal_introitus	Urogenital_tract	4388778	SRS015736.fna	7358	7008
SRS016215.SRX020522.V35	764649650	700024852	700024878	SRX020522	SRR044438	SRS016215	SRS016215	female	Vaginal_introitus	Urogenital_tract	4173490	SRS016215.fna	7101	6829
SRS016687.SRX019691.V35	160036745	700032470	700032471	SRX019691	SRR041604	SRS016687	SRS016687	female	Vaginal_introitus	Urogenital_tract	2343417	SRS016687.fna	3926	3779
SRS016728.SRX019682.V35	160016515	700032650	700032673	SRX019682	SRR040876	SRS016728	SRS016728	female	Vaginal_introitus	Urogenital_tract	2103629	SRS016728.fna	3550	3294
SRS018288.SRX019687.V35	160137896	700035654	700035695	SRX019687	SRR041227	SRS018288	SRS018288	female	Vaginal_introitus	Urogenital_tract	2755344	SRS018288.fna	4616	4284
SRS018527.SRX019639.V35	160259276	700035950	700035991	SRX019639	SRR040715	SRS018527	SRS018527	female	Vaginal_introitus	Urogenital_tract	1961653	SRS018527.fna	3307	2879
SRS018765.SRX020522.V35	765013792	700037139	700037232	SRX020522	SRR044415	SRS018765	SRS018765	female	Vaginal_introitus	Urogenital_tract	4470371	SRS018765.fna	7516	7233
SRS019241.SRX020522.V35	765034022	700037687	700037712	SRX020522	SRR044402	SRS019241	SRS019241	female	Vaginal_introitus	Urogenital_tract	4195406	SRS019241.fna	7093	6878
SRS021147.SRX022230.V35	160744799	700096609	700096694	SRX022230	SRR058093	SRS021147	SRS021147	female	Vaginal_introitus	Urogenital_tract	2305992	SRS021147.fna	3929	3142
SRS021213.SRX022239.V35	161007791	700096719	700096760	SRX022239	SRR058109	SRS021213	SRS021213	female	Vaginal_introitus	Urogenital_tract	3946332	SRS021213.fna	6642	5933
SRS021279.SRX022235.V35	161230322	700096884	700096925	SRX022235	SRR058095	SRS021279	SRS021279	female	Vaginal_introitus	Urogenital_tract	2654201	SRS021279.fna	4459	3674
SRS021402.SRX022239.V35	161028021	700097019	700097060	SRX022239	SRR058109	SRS021402	SRS021402	female	Vaginal_introitus	Urogenital_tract	2700784	SRS021402.fna	4595	4026
SRS021639.SRX020679.V35	158013734	700097310	700097351	SRX020679	SRR048058	SRS021639	SRS021639	female	Vaginal_introitus	Urogenital_tract	3192359	SRS021639.fna	5168	4347
SRS021762.SRX022231.V35	161493313	700097434	700097475	SRX022231	SRR058120	SRS021762	SRS021762	female	Vaginal_introitus	Urogenital_tract	2397139	SRS021762.fna	4042	3659
SRS021828.SRX022231.V35	161473083	700097500	700097541	SRX022231	SRR058120	SRS021828	SRS021828	female	Vaginal_introitus	Urogenital_tract	1785835	SRS021828.fna	2969	2644
SRS022008.SRX019635.V35	158256496	700097707	700097748	SRX019635	SRR040685	SRS022008	SRS022008	female	Vaginal_introitus	Urogenital_tract	5085438	SRS022008.fna	8647	8166
SRS022131.SRX019690.V35	158337416	700097856	700097897	SRX019690	SRR041569	SRS022131	SRS022131	female	Vaginal_introitus	Urogenital_tract	2221542	SRS022131.fna	3815	3655
SRS022197.SRX022231.V35	638754422	700097925	700097966	SRX022231	SRR058120	SRS022197	SRS022197	female	Vaginal_introitus	Urogenital_tract	2685296	SRS022197.fna	4515	4081
SRS022329.SRX022231.V35	533247696	700098128	700098169	SRX022231	SRR058120	SRS022329	SRS022329	female	Vaginal_introitus	Urogenital_tract	2854729	SRS022329.fna	4813	4319
SRS022452.SRX020680.V35	158742018	700098286	700098327	SRX020680	SRR048206	SRS022452	SRS022452	female	Vaginal_introitus	Urogenital_tract	3168810	SRS022452.fna	5192	4506
SRS022518.SRX020680.V35	158458797	700098378	700098419	SRX020680	SRR048188	SRS022518	SRS022518	female	Vaginal_introitus	Urogenital_tract	2509736	SRS022518.fna	4245	4059
SRS022584.SRX022226.V35	809635352	700098448	700098489	SRX022226	SRR058089	SRS022584	SRS022584	female	Vaginal_introitus	Urogenital_tract	2984023	SRS022584.fna	5057	4802
SRS022707.SRX022226.V35	432193348	700098618	700098659	SRX022226	SRR058089	SRS022707	SRS022707	female	Vaginal_introitus	Urogenital_tract	2657546	SRS022707.fna	4447	4126
SRS022773.SRX022226.V35	370425937	700098688	700098729	SRX022226	SRR058089	SRS022773	SRS022773	female	Vaginal_introitus	Urogenital_tract	2771555	SRS022773.fna	4732	4459
SRS022896.SRX022223.V35	650853796	700098885	700098926	SRX022223	SRR058086	SRS022896	SRS022896	female	Vaginal_introitus	Urogenital_tract	3971104	SRS022896.fna	6723	6470
SRS022962.SRX020679.V35	158236265	700098951	700098992	SRX020679	SRR048109	SRS022962	SRS022962	female	Vaginal_introitus	Urogenital_tract	2376213	SRS022962.fna	4012	3732
SRS023085.SRX020680.V35	158276726	700099078	700099119	SRX020680	SRR048127	SRS023085	SRS023085	female	Vaginal_introitus	Urogenital_tract	2467178	SRS023085.fna	4203	4061
SRS023145.SRX022226.V35	441369442	700099285	700099276	SRX022226	SRR058089	SRS023145	SRS023145	female	Vaginal_introitus	Urogenital_tract	2973371	SRS023145.fna	5064	4692
SRS023217.SRX020679.V35	158114885	700099345	700099348	SRX020679	SRR048071	SRS023217	SRS023217	female	Vaginal_introitus	Urogenital_tract	4095599	SRS023217.fna	6995	6582
SRS023340.SRX022223.V35	206906765	700099461	700099502	SRX022223	SRR058086	SRS023340	SRS023340	female	Vaginal_introitus	Urogenital_tract	2451650	SRS023340.fna	4208	4047
SRS023461.SRX022223.V35	246515023	700099739	700099738	SRX022223	SRR058086	SRS023461	SRS023461	female	Vaginal_introitus	Urogenital_tract	2928638	SRS023461.fna	4849	4504
SRS023889.SRX020681.V35	158883629	700100246	700100287	SRX020681	SRR048222	SRS023889	SRS023889	female	Vaginal_introitus	Urogenital_tract	2191449	SRS023889.fna	3685	3524
SRS024306.SRX019692.V35	158944319	700101281	700101284	SRX019692	SRR041759	SRS024306	SRS024306	female	Vaginal_introitus	Urogenital_tract	4803186	SRS024306.fna	8104	7629
SRS042961.SRX022222.V35	892969023	700110306	700110347	SRX022222	SRR058085	SRS042961	SRS042961	female	Vaginal_introitus	Urogenital_tract	2559192	SRS042961.fna	4318	3523
SRS043066.SRX020518.V35	737052003	700106062	700106087	SRX020518	SRR049609	SRS043066	SRS043066	female	Vaginal_introitus	Urogenital_tract	3296576	SRS043066.fna	5561	5250
SRS044715.SRX022227.V35	970836795	700109126	700109167	SRX022227	SRR058090	SRS044715	SRS044715	female	Vaginal_introitus	Urogenital_tract	3705798	SRS044715.fna	6282	4842
SRS044750.SRX020665.V35	763456073	700105682	700105717	SRX020665	SRR046279	SRS044750	SRS044750	female	Vaginal_introitus	Urogenital_tract	3241914	SRS044750.fna	5368	5024
SRS044945.SRX020685.V35	160319967	700109640	700109681	SRX020685	SRR048574	SRS044945	SRS044945	female	Vaginal_introitus	Urogenital_tract	5147184	SRS044945.fna	8399	6293
SRS044948.SRX020688.V35	160643649	700110108	700110149	SRX020688	SRR048759	SRS044948	SRS044948	female	Vaginal_introitus	Urogenital_tract	5763550	SRS044948.fna	9583	7658
SRS045920.SRX020533.V35	208027353	700106548	700106573	SRX020533	SRR046591	SRS045920	SRS045920	female	Vaginal_introitus	Urogenital_tract	4063559	SRS045920.fna	6753	6114
SRS048275.SRX022242.V35	937415040	700102538	700102579	SRX022242	SRR058098	SRS048275	SRS048275	female	Vaginal_introitus	Urogenital_tract	14671513	SRS048275.fna	24845	20463
SRS049964.SRX020573.V35	764366428	700024805	700024841	SRX020573	SRR044242	SRS049964	SRS049964	female	Vaginal_introitus	Urogenital_tract	2983390	SRS049964.fna	5015	4828
SRS050081.SRX020686.V35	160502038	700108114	700108155	SRX020686	SRR048628	SRS050081	SRS050081	female	Vaginal_introitus	Urogenital_tract	4923910	SRS050081.fna	8308	7920
SRS051407.SRX020665.V35	763820215	700105207	700105232	SRX020665	SRR047667	SRS051407	SRS051407	female	Vaginal_introitus	Urogenital_tract	3856560	SRS051407.fna	6548	6348
SRS051970.SRX021470.V35	514014184	700101790	700101831	SRX021470	SRR052699	SRS051970	SRS051970	female	Vaginal_introitus	Urogenital_tract	3708790	SRS051970.fna	6341	5987
SRS052468.SRX020684.V35	159470302	700108360	700108401	SRX020684	SRR048463	SRS052468	SRS052468	female	Vaginal_introitus	Urogenital_tract	5999621	SRS052468.fna	10081	9369
SRS053469.SRX021470.V35	414519462	700102132	700102173	SRX021470	SRR052699	SRS053469	SRS053469	female	Vaginal_introitus	Urogenital_tract	3799267	SRS053469.fna	6449	6131
SRS053877.SRX020687.V35	160704339	700108237	700108278	SRX020687	SRR048680	SRS053877	SRS053877	female	Vaginal_introitus	Urogenital_tract	6523273	SRS053877.fna	10961	9798
SRS054549.SRX020533.V35	553359145	700106491	700106516	SRX020533	SRR049505	SRS054549	SRS054549	female	Vaginal_introitus	Urogenital_tract	3202899	SRS054549.fna	5480	5230
SRS056514.SRX020685.V35	159915365	700108615	700108656	SRX020685	SRR048508	SRS056514	SRS056514	female	Vaginal_introitus	Urogenital_tract	3046599	SRS056514.fna	5177	4667
SRS056561.SRX022222.V35	160866180	700109336	700109378	SRX022222	SRR058085	SRS056561	SRS056561	female	Vaginal_introitus	Urogenital_tract	2309127	SRS056561.fna	3887	3265
SRS056693.SRX020533.V35	612472597	700105150	700105175	SRX020533	SRR046587	SRS056693	SRS056693	female	Vaginal_introitus	Urogenital_tract	5172345	SRS056693.fna	8727	8476
SRS056854.SRX020684.V35	159753524	700107394	700107435	SRX020684	SRR048491	SRS056854	SRS056854	female	Vaginal_introitus	Urogenital_tract	4730427	SRS056854.fna	7976	7718
SRS057898.SRX020686.V35	160582958	700108549	700108590	SRX020686	SRR048644	SRS057898	SRS057898	female	Vaginal_introitus	Urogenital_tract	3506060	SRS057898.fna	5801	4765
SRS062445.SRX020689.V35	937495960	700111593	700111634	SRX020689	SRR048777	SRS062445	SRS062445	female	Vaginal_introitus	Urogenital_tract	3614895	SRS062445.fna	5780	5129
SRS062896.SRX020689.V35	872848689	700111764	700111805	SRX020689	SRR048831	SRS062896	SRS062896	female	Vaginal_introitus	Urogenital_tract	3230128	SRS062896.fna	5406	4481
SRS063522.SRX020579.V35	663835652	700114210	700114230	SRX020579	SRR047034	SRS063522	SRS063522	female	Vaginal_introitus	Urogenital_tract	3428745	SRS063522.fna	5784	5354
SRS064618.SRX020689.V35	763721271	700111315	700111356	SRX020689	SRR048849	SRS064618	SRS064618	female	Vaginal_introitus	Urogenital_tract	5516844	SRS064618.fna	9045	7338
SRS064648.SRX020579.V35	375450439	700114804	700114830	SRX020579	SRR047105	SRS064648	SRS064648	female	Vaginal_introitus	Urogenital_tract	2901138	SRS064648.fna	4912	4573
SRS064833.SRX020689.V35	866653606	700111241	700111282	SRX020689	SRR048813	SRS064833	SRS064833	female	Vaginal_introitus	Urogenital_tract	1430560	SRS064833.fna	2411	1927
SRS064882.SRX020665.V35	763840445	700114121	700114157	SRX020665	SRR047673	SRS064882	SRS064882	female	Vaginal_introitus	Urogenital_tract	3846835	SRS064882.fna	6542	6065
SRS065165.SRX020528.V35	763719065	700113500	700113535	SRX020528	SRR046736	SRS065165	SRS065165	female	Vaginal_introitus	Urogenital_tract	4365334	SRS065165.fna	7374	6818
SRS065619.SRX020689.V35	257905678	700111175	700111216	SRX020689	SRR048795	SRS065619	SRS065619	female	Vaginal_introitus	Urogenital_tract	3164688	SRS065619.fna	5333	4402

Table S3. Relative abundance of "core" bacteria taxa (L6, at the genus and above levels) at the respective body site of macaques and humans.

Core Taxa	Body site	Macaque		Human			LDA score		
		Abundance[$] (%)	Prevalence* (%)	Abundance[$] (%)	Prevalence* (%)	MW test (p)	Macaque	Human	p value
k__Bacteria;p__Actinobacteria;c__Actinobacteria;o__Actinomycetales;f__Actinomycetaceae;g__Actinomyces	Oral	0.08 ± 0.01	0.0	2.64 ± 0.19	76.8	2.84E-45	-	4.1126	3.65E-45
k__Bacteria;p__Actinobacteria;c__Actinobacteria;o__Actinomycetales;f__Actinomycetaceae;g__Actinomyces	Anal								
k__Bacteria;p__Actinobacteria;c__Actinobacteria;o__Actinomycetales;f__Actinomycetaceae;g__Actinomyces	Vaginal								
k__Bacteria;p__Actinobacteria;c__Actinobacteria;o__Actinomycetales;f__Actinomycetaceae;g__Mobiluncus	Oral								
k__Bacteria;p__Actinobacteria;c__Actinobacteria;o__Actinomycetales;f__Actinomycetaceae;g__Mobiluncus	Anal								
k__Bacteria;p__Actinobacteria;c__Actinobacteria;o__Actinomycetales;f__Actinomycetaceae;g__Mobiluncus	Vaginal	1.69 ± 0.22	57.8	0.09 ± 0.05	4.1	5.60E-15	3.8735	-	8.41E-15
k__Bacteria;p__Actinobacteria;c__Actinobacteria;o__Bifidobacteriales;f__Bifidobacteriaceae;g__Bifidobacterium	Oral								
k__Bacteria;p__Actinobacteria;c__Actinobacteria;o__Bifidobacteriales;f__Bifidobacteriaceae;g__Bifidobacterium	Anal								
k__Bacteria;p__Actinobacteria;c__Actinobacteria;o__Bifidobacteriales;f__Bifidobacteriaceae;g__Bifidobacterium	Vaginal	0.01 ± 0.01	0.0	1.18 ± 1.04	4.1	9.37E-01	-	-	-
k__Bacteria;p__Actinobacteria;c__Actinobacteria;o__Bifidobacteriales;f__Bifidobacteriaceae;g__Gardnerella	Oral								
k__Bacteria;p__Actinobacteria;c__Actinobacteria;o__Bifidobacteriales;f__Bifidobacteriaceae;g__Gardnerella	Anal								
k__Bacteria;p__Actinobacteria;c__Actinobacteria;o__Bifidobacteriales;f__Bifidobacteriaceae;g__Gardnerella	Vaginal	0.42 ± 0.19	9.6	2.62 ± 0.87	17.8	3.79E-01	-	-	-
k__Bacteria;p__Actinobacteria;c__Actinobacteria;o__Corynebacteriales;f__Corynebacteriaceae;g__Corynebacterium	Oral								
k__Bacteria;p__Actinobacteria;c__Actinobacteria;o__Corynebacteriales;f__Corynebacteriaceae;g__Corynebacterium	Anal								
k__Bacteria;p__Actinobacteria;c__Actinobacteria;o__Corynebacteriales;f__Corynebacteriaceae;g__Corynebacterium	Vaginal	1.81 ± 0.49	25.3	0.21 ± 0.10	1.4	7.36E-06	3.9218	-	1.11E-05
k__Bacteria;p__Actinobacteria;c__Coriobacteriia;o__Coriobacteriales;f__Atopobiaceae;g__Atopobium	Oral								
k__Bacteria;p__Actinobacteria;c__Coriobacteriia;o__Coriobacteriales;f__Atopobiaceae;g__Atopobium	Anal								
k__Bacteria;p__Actinobacteria;c__Coriobacteriia;o__Coriobacteriales;f__Atopobiaceae;g__Atopobium	Vaginal	2.13 ± 0.44	43.4	1.24 ± 0.51	13.7	1.17E-13	3.7299	-	2.06E-13
k__Bacteria;p__Bacteroidetes;c__Bacteroidia;o__Bacteroidales;f__Bacteroidaceae;g__Bacteroides	Oral								
k__Bacteria;p__Bacteroidetes;c__Bacteroidia;o__Bacteroidales;f__Bacteroidaceae;g__Bacteroides	Anal	0.29 ± 0.12	3.5	51.73 ± 1.90	100.0	5.04E-45	-	5.4040	6.53E-45
k__Bacteria;p__Bacteroidetes;c__Bacteroidia;o__Bacteroidales;f__Bacteroidaceae;g__Bacteroides	Vaginal								
k__Bacteria;p__Bacteroidetes;c__Bacteroidia;o__Bacteroidales;f__Porphyromonadaceae;g__	Oral								
k__Bacteria;p__Bacteroidetes;c__Bacteroidia;o__Bacteroidales;f__Porphyromonadaceae;g__	Anal								
k__Bacteria;p__Bacteroidetes;c__Bacteroidia;o__Bacteroidales;f__Porphyromonadaceae;g__	Vaginal	1.23 ± 0.27	36.1	0.02 ± 0.01	1.4	3.16E-26	3.7992	-	5.66E-26
k__Bacteria;p__Bacteroidetes;c__Bacteroidia;o__Bacteroidales;f__Porphyromonadaceae;g__Parabacteroides	Oral								
k__Bacteria;p__Bacteroidetes;c__Bacteroidia;o__Bacteroidales;f__Porphyromonadaceae;g__Parabacteroides	Anal	0.04 ± 0.02	0.9	3.73 ± 0.38	73.4	1.73E-31	-	4.2579	2.47E-31
k__Bacteria;p__Bacteroidetes;c__Bacteroidia;o__Bacteroidales;f__Porphyromonadaceae;g__Parabacteroides	Vaginal								
k__Bacteria;p__Bacteroidetes;c__Bacteroidia;o__Bacteroidales;f__Porphyromonadaceae;g__Porphyromonas	Oral	3.58 ± 0.34	73.3	4.94 ± 0.35	86.9	0.0012186	-	3.817242	0.000919
k__Bacteria;p__Bacteroidetes;c__Bacteroidia;o__Bacteroidales;f__Porphyromonadaceae;g__Porphyromonas	Anal								
k__Bacteria;p__Bacteroidetes;c__Bacteroidia;o__Bacteroidales;f__Porphyromonadaceae;g__Porphyromonas	Vaginal	9.71 ± 0.92	94.0	0.16 ± 0.08	4.1	5.09E-26	4.6789	-	8.62E-26
k__Bacteria;p__Bacteroidetes;c__Bacteroidia;o__Bacteroidales;f__Prevotellaceae;g__	Oral								
k__Bacteria;p__Bacteroidetes;c__Bacteroidia;o__Bacteroidales;f__Prevotellaceae;g__	Anal								
k__Bacteria;p__Bacteroidetes;c__Bacteroidia;o__Bacteroidales;f__Prevotellaceae;g__	Vaginal	2.81 ± 0.36	56.6	0.00 ± 0.00	0.0	1.70E-25	4.1432	-	2.92E-25
k__Bacteria;p__Bacteroidetes;c__Bacteroidia;o__Bacteroidales;f__Prevotellaceae;g__Prevotella	Oral	1.28 ± 0.21	28.4	13.13 ± 0.62	99.4	7.1512E-42	-	4.770178	9.45E-42
k__Bacteria;p__Bacteroidetes;c__Bacteroidia;o__Bacteroidales;f__Prevotellaceae;g__Prevotella	Anal	18.67 ± 1.15	96.5	3.17 ± 0.75	17.7	4.92E-34	4.8825	-	7.82E-34
k__Bacteria;p__Bacteroidetes;c__Bacteroidia;o__Bacteroidales;f__Prevotellaceae;g__Prevotella	Vaginal	6.40 ± 0.60	94.0	4.32 ± 1.13	32.9	1.68E-09	4.0628	-	2.85E-09
k__Bacteria;p__Bacteroidetes;c__Bacteroidia;o__Bacteroidales;f__Prevotellaceae;g__Prevotellamassilia	Oral	1.06 ± 0.18	26.7	1.37 ± 0.13	43.5	1.39E-05	-	3.2302	1.28E-05
k__Bacteria;p__Bacteroidetes;c__Bacteroidia;o__Bacteroidales;f__Prevotellaceae;g__Prevotellamassilia	Anal	4.92 ± 0.43	74.8	0.14 ± 0.09	1.9	1.83E-49	4.3695	-	3.11E-49
k__Bacteria;p__Bacteroidetes;c__Bacteroidia;o__Bacteroidales;f__Prevotellaceae;g__Prevotellamassilia	Vaginal								
k__Bacteria;p__Bacteroidetes;c__Bacteroidia;o__Bacteroidales;f__Rikenellaceae;g__Alistipes	Oral								
k__Bacteria;p__Bacteroidetes;c__Bacteroidia;o__Bacteroidales;f__Rikenellaceae;g__Alistipes	Anal	0.01 ± 0.01	0.0	5.92 ± 0.49	81.6	1.94E-35	-	4.4765	2.65E-35
k__Bacteria;p__Bacteroidetes;c__Bacteroidia;o__Bacteroidales;f__Rikenellaceae;g__Alistipes	Vaginal								
k__Bacteria;p__Bacteroidetes;c__Flavobacteriia;o__Flavobacteriales;f__Flavobacteriaceae;g__Capnocytophaga	Oral	0.23 ± 0.03	4.3	1.85 ± 0.12	58.9	2.61E-35	-	3.9092	1.24E-35
k__Bacteria;p__Bacteroidetes;c__Flavobacteriia;o__Flavobacteriales;f__Flavobacteriaceae;g__Capnocytophaga	Anal								
k__Bacteria;p__Bacteroidetes;c__Flavobacteriia;o__Flavobacteriales;f__Flavobacteriaceae;g__Capnocytophaga	Vaginal								
k__Bacteria;p__Firmicutes;c__Bacilli;o__Bacillales;f__Family_XI;g__Gemella	Oral	6.19 ± 0.47	95.7	0.96 ± 0.06	33.3	5.5218E-39	4.396451	-	6.38E-39
k__Bacteria;p__Firmicutes;c__Bacilli;o__Bacillales;f__Family_XI;g__Gemella	Anal								
k__Bacteria;p__Firmicutes;c__Bacilli;o__Bacillales;f__Family_XI;g__Gemella	Vaginal								
k__Bacteria;p__Firmicutes;c__Bacilli;o__Lactobacillales;f__Carnobacteriaceae;g__Granulicatella	Oral	3.35 ± 0.26	82.8	1.45 ± 0.08	58.9	2.79E-11	3.9638	-	1.49E-11
k__Bacteria;p__Firmicutes;c__Bacilli;o__Lactobacillales;f__Carnobacteriaceae;g__Granulicatella	Anal								
k__Bacteria;p__Firmicutes;c__Bacilli;o__Lactobacillales;f__Carnobacteriaceae;g__Granulicatella	Vaginal								
k__Bacteria;p__Firmicutes;c__Bacilli;o__Lactobacillales;f__Lactobacillaceae;g__Lactobacillus	Oral								
k__Bacteria;p__Firmicutes;c__Bacilli;o__Lactobacillales;f__Lactobacillaceae;g__Lactobacillus	Anal								
k__Bacteria;p__Firmicutes;c__Bacilli;o__Lactobacillales;f__Lactobacillaceae;g__Lactobacillus	Vaginal	0.35 ± 0.07	13.3	78.65 ± 3.36	98.6	8.96E-26	-	5.5900	1.37E-25
k__Bacteria;p__Firmicutes;c__Bacilli;o__Lactobacillales;f__Streptococcaceae;g__Streptococcus	Oral	38.71 ± 1.41	100.0	15.31 ± 0.67	100.0	7.40E-33	5.0712	-	9.00E-33
k__Bacteria;p__Firmicutes;c__Bacilli;o__Lactobacillales;f__Streptococcaceae;g__Streptococcus	Anal								
k__Bacteria;p__Firmicutes;c__Bacilli;o__Lactobacillales;f__Streptococcaceae;g__Streptococcus	Vaginal	1.05 ± 0.57	13.3	0.71 ± 0.26	15.1	1.67E-01	-	-	-
k__Bacteria;p__Firmicutes;c__Clostridia;o__Clostridiales;f__Clostridiaceae;g__	Oral	0.02 ± 0.00	0	1.88 ± 0.27	39.9	4.9223E-29	-	3.946619	1.7E-29
k__Bacteria;p__Firmicutes;c__Clostridia;o__Clostridiales;f__Clostridiaceae;g__	Anal								
k__Bacteria;p__Firmicutes;c__Clostridia;o__Clostridiales;f__Clostridiaceae;g__	Vaginal								
k__Bacteria;p__Firmicutes;c__Clostridia;o__Clostridiales;f__Eubacteriaceae;g__Eubacterium	Oral								
k__Bacteria;p__Firmicutes;c__Clostridia;o__Clostridiales;f__Eubacteriaceae;g__Eubacterium	Anal	1.49 ± 0.11	61.7	1.17 ± 0.10	42.4	2.25E-03	3.1842	-	2.29E-03
k__Bacteria;p__Firmicutes;c__Clostridia;o__Clostridiales;f__Eubacteriaceae;g__Eubacterium	Vaginal								

k__Bacteria;p__Firmicutes;c__Clostridia;o__Clostridiales;f__Lachnospiraceae;g__	Oral								
k__Bacteria;p__Firmicutes;c__Clostridia;o__Clostridiales;f__Lachnospiraceae;g__	Anal								
k__Bacteria;p__Firmicutes;c__Clostridia;o__Clostridiales;f__Lachnospiraceae;g__	Vaginal	2.62 ± 0.36	55.4	0.00 ± 0.00	0.0	1.63E-28	4.0975	-	2.96E-28
k__Bacteria;p__Firmicutes;c__Clostridia;o__Clostridiales;f__Lachnospiraceae;g__Blautia	Oral								
k__Bacteria;p__Firmicutes;c__Clostridia;o__Clostridiales;f__Lachnospiraceae;g__Blautia	Anal	1.47 ± 0.12	49.6	0.77 ± 0.10	19.0	9.82E-10	3.5451	-	8.34E-10
k__Bacteria;p__Firmicutes;c__Clostridia;o__Clostridiales;f__Lachnospiraceae;g__Blautia	Vaginal								
k__Bacteria;p__Firmicutes;c__Clostridia;o__Clostridiales;f__Lachnospiraceae;g__Butyrivibrio	Oral								
k__Bacteria;p__Firmicutes;c__Clostridia;o__Clostridiales;f__Lachnospiraceae;g__Butyrivibrio	Anal								
k__Bacteria;p__Firmicutes;c__Clostridia;o__Clostridiales;f__Lachnospiraceae;g__Butyrivibrio	Vaginal	2.08 ± 0.43	42.2	0.00 ± 0.00	0.0	3.66E-20	4.0086	-	5.71E-20
k__Bacteria;p__Firmicutes;c__Clostridia;o__Clostridiales;f__Lachnospiraceae;g__[Clostridium]	Oral								
k__Bacteria;p__Firmicutes;c__Clostridia;o__Clostridiales;f__Lachnospiraceae;g__[Clostridium]	Anal	2.13 ± 0.16	68.7	0.44 ± 0.05	11.4	1.25E-24	3.9203	-	1.02E-24
k__Bacteria;p__Firmicutes;c__Clostridia;o__Clostridiales;f__Lachnospiraceae;g__[Clostridium]	Vaginal								
k__Bacteria;p__Firmicutes;c__Clostridia;o__Clostridiales;f__Lachnospiraceae;g__[Eubacterium]	Oral								
k__Bacteria;p__Firmicutes;c__Clostridia;o__Clostridiales;f__Lachnospiraceae;g__[Eubacterium]	Anal	1.10 ± 0.12	38.3	2.29 ± 0.31	45.6	2.37E-02	-	3.8016	2.34E-02
k__Bacteria;p__Firmicutes;c__Clostridia;o__Clostridiales;f__Lachnospiraceae;g__[Eubacterium]	Vaginal								
k__Bacteria;p__Firmicutes;c__Clostridia;o__Clostridiales;f__Lachnospiraceae;g__Oribacterium	Oral	0.01 ± 0.00	0.0	2.96 ± 0.26	80.4	1.40E-46	-	4.1621	1.76E-46
k__Bacteria;p__Firmicutes;c__Clostridia;o__Clostridiales;f__Lachnospiraceae;g__Oribacterium	Anal								
k__Bacteria;p__Firmicutes;c__Clostridia;o__Clostridiales;f__Lachnospiraceae;g__Oribacterium	Vaginal								
k__Bacteria;p__Firmicutes;c__Clostridia;o__Clostridiales;f__Lachnospiraceae;g__Roseburia	Oral								
k__Bacteria;p__Firmicutes;c__Clostridia;o__Clostridiales;f__Lachnospiraceae;g__Roseburia	Anal	1.30 ± 0.18	36.5	0.40 ± 0.05	12.7	3.52E-07	3.6267	-	3.87E-07
k__Bacteria;p__Firmicutes;c__Clostridia;o__Clostridiales;f__Lachnospiraceae;g__Roseburia	Vaginal								
k__Bacteria;p__Firmicutes;c__Clostridia;o__Clostridiales;f__Oscillospiraceae;g__Oscillibacter	Oral								
k__Bacteria;p__Firmicutes;c__Clostridia;o__Clostridiales;f__Oscillospiraceae;g__Oscillibacter	Anal	0.71 ± 0.11	14.8	3.10 ± 0.35	65.2	5.86E-15	-	4.0853	8.16E-15
k__Bacteria;p__Firmicutes;c__Clostridia;o__Clostridiales;f__Oscillospiraceae;g__Oscillibacter	Vaginal								
k__Bacteria;p__Firmicutes;c__Clostridia;o__Clostridiales;f__Peptostreptococcaceae;g__	Oral								
k__Bacteria;p__Firmicutes;c__Clostridia;o__Clostridiales;f__Peptostreptococcaceae;g__	Anal								
k__Bacteria;p__Firmicutes;c__Clostridia;o__Clostridiales;f__Peptostreptococcaceae;g__	Vaginal	1.58 ± 0.25	43.4	0.00 ± 0.00	0.0	9.99E-25	3.8819	-	1.70E-24
k__Bacteria;p__Firmicutes;c__Clostridia;o__Clostridiales;f__Ruminococcaceae;g__	Oral								
k__Bacteria;p__Firmicutes;c__Clostridia;o__Clostridiales;f__Ruminococcaceae;g__	Anal	1.37 ± 0.16	47.8	1.98 ± 0.26	50.0	9.42E-01	-	-	-
k__Bacteria;p__Firmicutes;c__Clostridia;o__Clostridiales;f__Ruminococcaceae;g__	Vaginal	2.15 ± 0.29	59.0	0.00 ± 0.00	0.0	5.24E-29	4.0350	-	9.48E-29
k__Bacteria;p__Firmicutes;c__Clostridia;o__Clostridiales;f__Ruminococcaceae;g__Faecalibacterium	Oral								
k__Bacteria;p__Firmicutes;c__Clostridia;o__Clostridiales;f__Ruminococcaceae;g__Faecalibacterium	Anal	4.80 ± 0.40	84.3	4.55 ± 0.42	73.4	8.48E-02	-	-	-
k__Bacteria;p__Firmicutes;c__Clostridia;o__Clostridiales;f__Ruminococcaceae;g__Faecalibacterium	Vaginal								
k__Bacteria;p__Firmicutes;c__Clostridia;o__Clostridiales;f__Ruminococcaceae;g__Ruminococcus	Oral								
k__Bacteria;p__Firmicutes;c__Clostridia;o__Clostridiales;f__Ruminococcaceae;g__Ruminococcus	Anal	0.98 ± 0.12	31.3	1.86 ± 0.30	46.8	2.95E-01	-	-	-
k__Bacteria;p__Firmicutes;c__Clostridia;o__Clostridiales;f__Ruminococcaceae;g__Ruminococcus	Vaginal								
k__Bacteria;p__Firmicutes;c__Clostridia;o__Clostridiales;f__Ruminococcaceae;g__Saccharofermentans	Oral								
k__Bacteria;p__Firmicutes;c__Clostridia;o__Clostridiales;f__Ruminococcaceae;g__Saccharofermentans	Anal								
k__Bacteria;p__Firmicutes;c__Clostridia;o__Clostridiales;f__Ruminococcaceae;g__Saccharofermentans	Vaginal	6.91 ± 0.70	66.3	0.55 ± 0.31	6.8	1.03E-16	4.4911	-	1.62E-16
k__Bacteria;p__Firmicutes;c__Negativicutes;o__Acidaminococcales;f__Acidaminococcaceae;g__Phascolarctobacterium	Oral								
k__Bacteria;p__Firmicutes;c__Negativicutes;o__Acidaminococcales;f__Acidaminococcaceae;g__Phascolarctobacterium	Anal	2.61 ± 0.23	73.0	1.03 ± 0.16	25.9	4.48E-18	3.8915	-	6.15E-18
k__Bacteria;p__Firmicutes;c__Negativicutes;o__Acidaminococcales;f__Acidaminococcaceae;g__Phascolarctobacterium	Vaginal								
k__Bacteria;p__Firmicutes;c__Negativicutes;o__Selenomonadales;f__Selenomonadaceae;g__	Oral	0.00 ± 0.00	0	1.88 ± 0.22	41.7	3.2636E-40	-	3.982728	4.21E-40
k__Bacteria;p__Firmicutes;c__Negativicutes;o__Selenomonadales;f__Selenomonadaceae;g__	Anal								
k__Bacteria;p__Firmicutes;c__Negativicutes;o__Selenomonadales;f__Selenomonadaceae;g__	Vaginal								
k__Bacteria;p__Firmicutes;c__Negativicutes;o__Selenomonadales;f__Selenomonadaceae;g__Propionispira	Oral								
k__Bacteria;p__Firmicutes;c__Negativicutes;o__Selenomonadales;f__Selenomonadaceae;g__Propionispira	Anal	3.37 ± 0.35	70.4	0.00 ± 0.00	0.0	1.48E-53	4.2199	-	2.54E-53
k__Bacteria;p__Firmicutes;c__Negativicutes;o__Selenomonadales;f__Selenomonadaceae;g__Propionispira	Vaginal								
k__Bacteria;p__Firmicutes;c__Negativicutes;o__Selenomonadales;f__Selenomonadaceae;g__Selenomonas	Oral								
k__Bacteria;p__Firmicutes;c__Negativicutes;o__Selenomonadales;f__Selenomonadaceae;g__Selenomonas	Anal	1.09 ± 0.17	27.0	0.00 ± 0.00	0.0	3.25E-52	3.7064	-	5.52E-52
k__Bacteria;p__Firmicutes;c__Negativicutes;o__Selenomonadales;f__Selenomonadaceae;g__Selenomonas	Vaginal	1.01 ± 0.15	33.7	0.00 ± 0.00	0.0	1.74E-28	3.6912	-	3.15E-28
k__Bacteria;p__Firmicutes;c__Negativicutes;o__Veillonellales;f__Veillonellaceae;g__Dialister	Oral								
k__Bacteria;p__Firmicutes;c__Negativicutes;o__Veillonellales;f__Veillonellaceae;g__Dialister	Anal	1.29 ± 0.16	33.0	1.19 ± 0.26	22.2	6.46E-09	3.3307	-	9.29E-09
k__Bacteria;p__Firmicutes;c__Negativicutes;o__Veillonellales;f__Veillonellaceae;g__Dialister	Vaginal	5.53 ± 0.61	84.3	0.47 ± 0.15	12.3	1.18E-21	4.3828	-	2.03E-21
k__Bacteria;p__Firmicutes;c__Negativicutes;o__Veillonellales;f__Veillonellaceae;g__Megasphaera	Oral								
k__Bacteria;p__Firmicutes;c__Negativicutes;o__Veillonellales;f__Veillonellaceae;g__Megasphaera	Anal	1.11 ± 0.46	20.0	0.01 ± 0.01	0.0	3.57E-38	3.7326	-	5.60E-38
k__Bacteria;p__Firmicutes;c__Negativicutes;o__Veillonellales;f__Veillonellaceae;g__Megasphaera	Vaginal								
k__Bacteria;p__Firmicutes;c__Negativicutes;o__Veillonellales;f__Veillonellaceae;g__Veillonella	Oral	4.78 ± 0.43	78.4	12.58 ± 0.52	100.0	2.37E-24	-	4.5880	3.40E-24
k__Bacteria;p__Firmicutes;c__Negativicutes;o__Veillonellales;f__Veillonellaceae;g__Veillonella	Anal								
k__Bacteria;p__Firmicutes;c__Negativicutes;o__Veillonellales;f__Veillonellaceae;g__Veillonella	Vaginal								
k__Bacteria;p__Firmicutes;c__Tissierellia;o__Tissierellales;f__Peptoniphilaceae;g__	Oral								
k__Bacteria;p__Firmicutes;c__Tissierellia;o__Tissierellales;f__Peptoniphilaceae;g__	Anal								
k__Bacteria;p__Firmicutes;c__Tissierellia;o__Tissierellales;f__Peptoniphilaceae;g__	Vaginal	2.25 ± 0.28	60.2	0.08 ± 0.04	4.1	1.56E-24	4.0407	-	2.72E-24
k__Bacteria;p__Firmicutes;c__Tissierellia;o__Tissierellales;f__Peptoniphilaceae;g__Anaerococcus	Oral								
k__Bacteria;p__Firmicutes;c__Tissierellia;o__Tissierellales;f__Peptoniphilaceae;g__Anaerococcus	Anal								
k__Bacteria;p__Firmicutes;c__Tissierellia;o__Tissierellales;f__Peptoniphilaceae;g__Anaerococcus	Vaginal	1.95 ± 0.59	27.7	1.20 ± 0.50	13.7	1.24E-04	3.7581	-	1.34E-04
k__Bacteria;p__Firmicutes;c__Tissierellia;o__Tissierellales;f__Peptoniphilaceae;g__Peptoniphilus	Oral								

k__Bacteria;p__Firmicutes;c__Tissierellia;o__Tissierellales;f__Peptoniphilaceae;g__Peptoniphilus	Anal								
k__Bacteria;p__Firmicutes;c__Tissierellia;o__Tissierellales;f__Peptoniphilaceae;g__Peptoniphilus	Vaginal	1.97 ± 0.25	55.4	0.70 ± 0.20	16.4	3.06E-13	3.7732	-	4.85E-13
k__Bacteria;p__Fusobacteria;c__Fusobacteriia;o__Fusobacteriales;f__Fusobacteriaceae;g__Fusobacterium	Oral	2.55 ± 0.27	60.3	3.39 ± 0.24	79.8	2.80E-04	-	3.6346	2.83E-04
k__Bacteria;p__Fusobacteria;c__Fusobacteriia;o__Fusobacteriales;f__Fusobacteriaceae;g__Fusobacterium	Anal								
k__Bacteria;p__Fusobacteria;c__Fusobacteriia;o__Fusobacteriales;f__Fusobacteriaceae;g__Fusobacterium	Vaginal	5.35 ± 0.89	56.6	0.08 ± 0.05	2.7	4.02E-19	4.4177	-	6.52E-19
k__Bacteria;p__Fusobacteria;c__Fusobacteriia;o__Fusobacteriales;f__Leptotrichiaceae;g__Leptotrichia	Oral	1.86 ± 0.18	57.8	1.45 ± 0.09	58.3	0.46282095	-	-	-
k__Bacteria;p__Fusobacteria;c__Fusobacteriia;o__Fusobacteriales;f__Leptotrichiaceae;g__Leptotrichia	Anal								
k__Bacteria;p__Fusobacteria;c__Fusobacteriia;o__Fusobacteriales;f__Leptotrichiaceae;g__Leptotrichia	Vaginal								
k__Bacteria;p__Fusobacteria;c__Fusobacteriia;o__Fusobacteriales;f__Leptotrichiaceae;g__Sneathia	Oral								
k__Bacteria;p__Fusobacteria;c__Fusobacteriia;o__Fusobacteriales;f__Leptotrichiaceae;g__Sneathia	Anal								
k__Bacteria;p__Fusobacteria;c__Fusobacteriia;o__Fusobacteriales;f__Leptotrichiaceae;g__Sneathia	Vaginal	4.98 ± 0.66	67.5	0.19 ± 0.13	2.7	8.96E-21	4.3698	-	1.48E-20
k__Bacteria;p__Proteobacteria;c__Alphaproteobacteria;o__Rhodospirillales;f__Acetobacteraceae;g__	Oral	1.39 ± 0.48	17.2	0.00 ± 0.00	0	4.301E-56	3.860116	-	7.35E-56
k__Bacteria;p__Proteobacteria;c__Alphaproteobacteria;o__Rhodospirillales;f__Acetobacteraceae;g__	Anal								
k__Bacteria;p__Proteobacteria;c__Alphaproteobacteria;o__Rhodospirillales;f__Acetobacteraceae;g__	Vaginal								
k__Bacteria;p__Proteobacteria;c__Betaproteobacteria;o__Burkholderiales;f__Burkholderiaceae;g__Lautropia	Oral	0.17 ± 0.04	3.4	1.66 ± 0.21	41.1	2.74E-20	-	3.8870	9.07E-21
k__Bacteria;p__Proteobacteria;c__Betaproteobacteria;o__Burkholderiales;f__Burkholderiaceae;g__Lautropia	Anal								
k__Bacteria;p__Proteobacteria;c__Betaproteobacteria;o__Burkholderiales;f__Burkholderiaceae;g__Lautropia	Vaginal								
k__Bacteria;p__Proteobacteria;c__Betaproteobacteria;o__Burkholderiales;f__Sutterellaceae;g__Sutterella	Oral								
k__Bacteria;p__Proteobacteria;c__Betaproteobacteria;o__Burkholderiales;f__Sutterellaceae;g__Sutterella	Anal	0.29 ± 0.04	9.6	1.09 ± 0.20	23.4	2.21E-02	-	3.6299	1.66E-02
k__Bacteria;p__Proteobacteria;c__Betaproteobacteria;o__Burkholderiales;f__Sutterellaceae;g__Sutterella	Vaginal								
k__Bacteria;p__Proteobacteria;c__Betaproteobacteria;o__Neisseriales;f__Neisseriaceae;g__	Oral	0.74 ± 0.17	12.9	1.80 ± 0.18	48.8	7.87E-08	-	3.7138	4.22E-08
k__Bacteria;p__Proteobacteria;c__Betaproteobacteria;o__Neisseriales;f__Neisseriaceae;g__	Anal								
k__Bacteria;p__Proteobacteria;c__Betaproteobacteria;o__Neisseriales;f__Neisseriaceae;g__	Vaginal								
k__Bacteria;p__Proteobacteria;c__Betaproteobacteria;o__Neisseriales;f__Neisseriaceae;g__Neisseria	Oral	0.18 ± 0.03	3.4	4.89 ± 0.46	73.8	1.132E-37	-	4.372187	1.35E-37
k__Bacteria;p__Proteobacteria;c__Betaproteobacteria;o__Neisseriales;f__Neisseriaceae;g__Neisseria	Anal								
k__Bacteria;p__Proteobacteria;c__Betaproteobacteria;o__Neisseriales;f__Neisseriaceae;g__Neisseria	Vaginal								
k__Bacteria;p__Proteobacteria;c__Epsilonproteobacteria;o__Campylobacterales;f__Campylobacteraceae;g__Campylobacter	Oral	0.21 ± 0.03	5.2	2.14 ± 0.20	58.3	1.36E-13	-	3.9889	1.96E-13
k__Bacteria;p__Proteobacteria;c__Epsilonproteobacteria;o__Campylobacterales;f__Campylobacteraceae;g__Campylobacter	Anal								
k__Bacteria;p__Proteobacteria;c__Epsilonproteobacteria;o__Campylobacterales;f__Campylobacteraceae;g__Campylobacter	Vaginal	8.47 ± 0.75	85.5	0.28 ± 0.16	5.5	8.52E-25	4.6046	-	1.48E-24
k__Bacteria;p__Proteobacteria;c__Epsilonproteobacteria;o__Campylobacterales;f__Helicobacteraceae;g__Helicobacter	Oral								
k__Bacteria;p__Proteobacteria;c__Epsilonproteobacteria;o__Campylobacterales;f__Helicobacteraceae;g__Helicobacter	Anal	30.74 ± 2.90	96.5	0.00 ± 0.00	0.0	1.33E-55	5.1906	-	2.31E-55
k__Bacteria;p__Proteobacteria;c__Epsilonproteobacteria;o__Campylobacterales;f__Helicobacteraceae;g__Helicobacter	Vaginal								
k__Bacteria;p__Proteobacteria;c__Gammaproteobacteria;o__Aeromonadales;f__Succinivibrionaceae;g__Succinivibrio	Oral								
k__Bacteria;p__Proteobacteria;c__Gammaproteobacteria;o__Aeromonadales;f__Succinivibrionaceae;g__Succinivibrio	Anal	1.60 ± 0.17	47.8	0.03 ± 0.03	0.6	8.84E-53	3.8896	-	1.53E-52
k__Bacteria;p__Proteobacteria;c__Gammaproteobacteria;o__Aeromonadales;f__Succinivibrionaceae;g__Succinivibrio	Vaginal								
k__Bacteria;p__Proteobacteria;c__Gammaproteobacteria;o__Oceanospirillales;f__Halomonadaceae;g__Halomonas	Oral								
k__Bacteria;p__Proteobacteria;c__Gammaproteobacteria;o__Oceanospirillales;f__Halomonadaceae;g__Halomonas	Anal								
k__Bacteria;p__Proteobacteria;c__Gammaproteobacteria;o__Oceanospirillales;f__Halomonadaceae;g__Halomonas	Vaginal	2.44 ± 0.63	27.7	0.00 ± 0.00	0.0	6.95E-30	4.1239	-	1.29E-29
k__Bacteria;p__Proteobacteria;c__Gammaproteobacteria;o__Pasteurellales;f__Pasteurellaceae;g__Aggregatibacter	Oral	1.62 ± 0.12	67.2	0.64 ± 0.07	18.5	1.30E-20	3.6773	-	1.91E-20
k__Bacteria;p__Proteobacteria;c__Gammaproteobacteria;o__Pasteurellales;f__Pasteurellaceae;g__Aggregatibacter	Anal								
k__Bacteria;p__Proteobacteria;c__Gammaproteobacteria;o__Pasteurellales;f__Pasteurellaceae;g__Aggregatibacter	Vaginal								
k__Bacteria;p__Proteobacteria;c__Gammaproteobacteria;o__Pasteurellales;f__Pasteurellaceae;g__Haemophilus	Oral	26.42 ± 1.00	100	12.62 ± 0.60	99.4	2.0418E-23	4.835868	-	2.98E-23
k__Bacteria;p__Proteobacteria;c__Gammaproteobacteria;o__Pasteurellales;f__Pasteurellaceae;g__Haemophilus	Anal								
k__Bacteria;p__Proteobacteria;c__Gammaproteobacteria;o__Pasteurellales;f__Pasteurellaceae;g__Haemophilus	Vaginal	1.11 ± 0.55	8.4	0.02 ± 0.01	0.0	3.55E-11	3.7460	-	5.24E-11
k__Bacteria;p__Spirochaetes;c__Spirochaetia;o__Spirochaetales;f__Spirochaetaceae;g__	Oral								
k__Bacteria;p__Spirochaetes;c__Spirochaetia;o__Spirochaetales;f__Spirochaetaceae;g__	Anal								
k__Bacteria;p__Spirochaetes;c__Spirochaetia;o__Spirochaetales;f__Spirochaetaceae;g__	Vaginal	3.59 ± 0.48	57.8	0.00 ± 0.00	0.0	7.81E-23	4.2443	-	1.28E-22

$ mean ± s.e.m

* Individual samples with >1% abundance were counted.

Table S4. Relative abundance of bactarial taxa (L6, at the genus and above levels) at the oral site of macaques and humans.

Oral site	Macaque		Human			LDA score		
	Abundance$ (%)	Prevalence* (%)	Abundance$ (%)	Prevalence* (%)	MW test (p)	Macaque	Human	p value
k__Bacteria;p__Acidobacteria;c__Acidobacteriia;o__Acidobacteriales;f__Acidobacteriaceae;g__	0.00 ± 0.00	0.0	0.00 ± 0.00	0.0	NA	-	-	-
k__Bacteria;p__Acidobacteria;c__Acidobacteriia;o__Acidobacteriales;f__Acidobacteriaceae;g__Terriglobus	0.00 ± 0.00	0.0	0.00 ± 0.00	0.0	8.92E-02	-	-	-
k__Bacteria;p__Acidobacteria;c__Holophagae;o__;f__;g__	0.00 ± 0.00	0.0	0.00 ± 0.00	0.0	NA	-	-	-
k__Bacteria;p__Acidobacteria;c__Solibacteres;o__Solibacterales;f__Bryobacteraceae;g__Paludibaculum	0.00 ± 0.00	0.0	0.00 ± 0.00	0.0	2.32E-01	-	-	-
k__Bacteria;p__Actinobacteria;c__Actinobacteria;o__Actinomycetales;f__Actinomycetaceae;g__	0.00 ± 0.00	0.0	0.00 ± 0.00	0.0	2.32E-01	-	-	-
k__Bacteria;p__Actinobacteria;c__Actinobacteria;o__Actinomycetales;f__Actinomycetaceae;g__Actinobaculum	0.01 ± 0.00	0.0	0.00 ± 0.00	0.0	1.49E-14	-	-	1.74E-14
**k__Bacteria;p__Actinobacteria;c__Actinobacteria;o__Actinomycetales;f__Actinomycetaceae;g__Actinomyces**	**0.08 ± 0.01**	**0.0**	**2.64 ± 0.19**	**76.8**	**2.84E-45**	**-**	**4.1126**	**3.65E-45**
k__Bacteria;p__Actinobacteria;c__Actinobacteria;o__Actinomycetales;f__Actinomycetaceae;g__Actinotignum	0.01 ± 0.00	0.0	0.01 ± 0.00	0.0	7.88E-01	-	-	-
k__Bacteria;p__Actinobacteria;c__Actinobacteria;o__Actinomycetales;f__Actinomycetaceae;g__Arcanobacterium	0.00 ± 0.00	0.0	0.00 ± 0.00	0.0	NA	-	-	-
k__Bacteria;p__Actinobacteria;c__Actinobacteria;o__Actinomycetales;f__Actinomycetaceae;g__Flaviflexus	0.00 ± 0.00	0.0	0.00 ± 0.00	0.0	2.32E-01	-	-	-
k__Bacteria;p__Actinobacteria;c__Actinobacteria;o__Actinomycetales;f__Actinomycetaceae;g__Mobiluncus	0.00 ± 0.00	0.0	0.06 ± 0.01	1.8	1.26E-07	-	2.4894	1.12E-07
k__Bacteria;p__Actinobacteria;c__Actinobacteria;o__Actinomycetales;f__Actinomycetaceae;g__Trueperella	0.00 ± 0.00	0.0	0.00 ± 0.00	0.0	2.32E-01	-	-	-
k__Bacteria;p__Actinobacteria;c__Actinobacteria;o__Actinomycetales;f__Actinomycetaceae;g__Varibaculum	0.00 ± 0.00	0.0	0.00 ± 0.00	0.0	NA	-	-	-
k__Bacteria;p__Actinobacteria;c__Actinobacteria;o__Bifidobacteriales;f__Bifidobacteriaceae;g__Alloscardovia	0.80 ± 0.14	19.8	0.00 ± 0.00	0.0	7.20E-46	3.6032	-	1.16E-45
k__Bacteria;p__Actinobacteria;c__Actinobacteria;o__Bifidobacteriales;f__Bifidobacteriaceae;g__Bifidobacterium	0.00 ± 0.00	0.0	0.01 ± 0.00	0.0	3.21E-01	-	-	-
k__Bacteria;p__Actinobacteria;c__Actinobacteria;o__Bifidobacteriales;f__Bifidobacteriaceae;g__Gardnerella	0.00 ± 0.00	0.0	0.00 ± 0.00	0.0	4.10E-01	-	-	-
k__Bacteria;p__Actinobacteria;c__Actinobacteria;o__Bifidobacteriales;f__Bifidobacteriaceae;g__Scardovia	0.00 ± 0.00	0.0	0.00 ± 0.00	0.0	9.44E-05	-	-	9.97E-05
k__Bacteria;p__Actinobacteria;c__Actinobacteria;o__Corynebacteriales;f__Corynebacteriaceae;g__Corynebacterium	0.09 ± 0.01	0.0	0.21 ± 0.02	2.4	1.25E-04	-	2.7991	8.27E-05
k__Bacteria;p__Actinobacteria;c__Actinobacteria;o__Corynebacteriales;f__Dietziaceae;g__Dietzia	0.00 ± 0.00	0.0	0.00 ± 0.00	0.0	1.67E-01	-	-	-
k__Bacteria;p__Actinobacteria;c__Actinobacteria;o__Corynebacteriales;f__Mycobacteriaceae;g__Mycobacterium	0.00 ± 0.00	0.0	0.00 ± 0.00	0.0	2.32E-01	-	-	-
k__Bacteria;p__Actinobacteria;c__Actinobacteria;o__Corynebacteriales;f__Nocardiaceae;g__	0.00 ± 0.00	0.0	0.00 ± 0.00	0.0	NA	-	-	-
k__Bacteria;p__Actinobacteria;c__Actinobacteria;o__Frankiales;f__;g__	0.00 ± 0.00	0.0	0.00 ± 0.00	0.0	2.32E-01	-	-	-
k__Bacteria;p__Actinobacteria;c__Actinobacteria;o__Frankiales;f__Antricoccus;g__	0.00 ± 0.00	0.0	0.00 ± 0.00	0.0	NA	-	-	-
k__Bacteria;p__Actinobacteria;c__Actinobacteria;o__Frankiales;f__Frankiaceae;g__Jatrophihabitans	0.00 ± 0.00	0.0	0.00 ± 0.00	0.0	3.68E-02	2.5923	-	3.70E-02
k__Bacteria;p__Actinobacteria;c__Actinobacteria;o__Kineosporiales;f__Kineosporiaceae;g__Kineococcus	0.00 ± 0.00	0.0	0.00 ± 0.00	0.0	NA	-	-	-
k__Bacteria;p__Actinobacteria;c__Actinobacteria;o__Micrococcales;f__Bogoriellaceae;g__Bogoriella	0.00 ± 0.00	0.0	0.00 ± 0.00	0.0	NA	-	-	-
k__Bacteria;p__Actinobacteria;c__Actinobacteria;o__Micrococcales;f__Brevibacteriaceae;g__Brevibacterium	0.00 ± 0.00	0.0	0.00 ± 0.00	0.0	NA	-	-	-
k__Bacteria;p__Actinobacteria;c__Actinobacteria;o__Micrococcales;f__Cellulomonadaceae;g__Cellulomonas	0.00 ± 0.00	0.0	0.00 ± 0.00	0.0	2.32E-01	-	-	-
k__Bacteria;p__Actinobacteria;c__Actinobacteria;o__Micrococcales;f__Dermabacteraceae;g__Brachybacterium	0.00 ± 0.00	0.0	0.00 ± 0.00	0.0	1.57E-02	-	-	1.58E-02
k__Bacteria;p__Actinobacteria;c__Actinobacteria;o__Micrococcales;f__Dermatophilaceae;g__Dermatophilus	0.00 ± 0.00	0.0	0.00 ± 0.00	0.0	2.32E-01	-	-	-
k__Bacteria;p__Actinobacteria;c__Actinobacteria;o__Micrococcales;f__Dermatophilaceae;g__Kineosphaera	0.00 ± 0.00	0.0	0.00 ± 0.00	0.0	2.32E-01	-	-	-
k__Bacteria;p__Actinobacteria;c__Actinobacteria;o__Micrococcales;f__Dermatophilaceae;g__Piscicoccus	0.00 ± 0.00	0.0	0.00 ± 0.00	0.0	NA	-	-	-
k__Bacteria;p__Actinobacteria;c__Actinobacteria;o__Micrococcales;f__Intrasporangiaceae;g__Knoellia	0.00 ± 0.00	0.0	0.00 ± 0.00	0.0	1.57E-02	-	-	1.58E-02
k__Bacteria;p__Actinobacteria;c__Actinobacteria;o__Micrococcales;f__Intrasporangiaceae;g__Phycicoccus	0.00 ± 0.00	0.0	0.00 ± 0.00	0.0	NA	-	-	-
k__Bacteria;p__Actinobacteria;c__Actinobacteria;o__Micrococcales;f__Microbacteriaceae;g__	0.00 ± 0.00	0.0	0.00 ± 0.00	0.0	2.97E-03	-	-	3.02E-03
k__Bacteria;p__Actinobacteria;c__Actinobacteria;o__Micrococcales;f__Microbacteriaceae;g__Amnibacterium	0.00 ± 0.00	0.0	0.00 ± 0.00	0.0	6.99E-03	-	-	7.14E-03
k__Bacteria;p__Actinobacteria;c__Actinobacteria;o__Micrococcales;f__Microbacteriaceae;g__Leucobacter	0.00 ± 0.00	0.0	0.00 ± 0.00	0.0	2.32E-01	-	-	-
k__Bacteria;p__Actinobacteria;c__Actinobacteria;o__Micrococcales;f__Micrococcaceae;g__Arthrobacter	0.00 ± 0.00	0.0	0.00 ± 0.00	0.0	3.68E-02	-	-	3.70E-02
k__Bacteria;p__Actinobacteria;c__Actinobacteria;o__Micrococcales;f__Micrococcaceae;g__Glutamicibacter	0.00 ± 0.00	0.0	0.00 ± 0.00	0.0	8.92E-02	-	-	-
k__Bacteria;p__Actinobacteria;c__Actinobacteria;o__Micrococcales;f__Micrococcaceae;g__Kocuria	0.00 ± 0.00	0.0	0.00 ± 0.00	0.0	5.74E-04	-	-	5.90E-04
k__Bacteria;p__Actinobacteria;c__Actinobacteria;o__Micrococcales;f__Micrococcaceae;g__Micrococcus	0.00 ± 0.00	0.0	0.00 ± 0.00	0.0	1.57E-02	-	-	1.58E-02
k__Bacteria;p__Actinobacteria;c__Actinobacteria;o__Micrococcales;f__Micrococcaceae;g__Paenarthrobacter	0.00 ± 0.00	0.0	0.00 ± 0.00	0.0	2.32E-01	-	-	-
k__Bacteria;p__Actinobacteria;c__Actinobacteria;o__Micrococcales;f__Micrococcaceae;g__Rothia	0.07 ± 0.01	0.9	0.80 ± 0.07	26.8	5.78E-31	-	3.5726	7.65E-31
k__Bacteria;p__Actinobacteria;c__Actinobacteria;o__Micromonosporales;f__Micromonosporaceae;g__Catellatospora	0.00 ± 0.00	0.0	0.00 ± 0.00	0.0	2.32E-01	-	-	-
k__Bacteria;p__Actinobacteria;c__Actinobacteria;o__Nakamurellales;f__Nakamurellaceae;g__Nakamurella	0.00 ± 0.00	0.0	0.00 ± 0.00	0.0	8.92E-02	-	-	-
k__Bacteria;p__Actinobacteria;c__Actinobacteria;o__Propionibacteriales;f__Nocardioidaceae;g__Marmoricola	0.00 ± 0.00	0.0	0.00 ± 0.00	0.0	2.32E-01	-	-	-
k__Bacteria;p__Actinobacteria;c__Actinobacteria;o__Propionibacteriales;f__Propionibacteriaceae;g__Cutibacterium	0.00 ± 0.00	0.0	0.10 ± 0.05	1.2	1.59E-09	-	2.6707	2.28E-09
k__Bacteria;p__Actinobacteria;c__Actinobacteria;o__Propionibacteriales;f__Propionibacteriaceae;g__Propionimicrobium	0.00 ± 0.00	0.0	0.00 ± 0.00	0.0	NA	-	-	-
k__Bacteria;p__Actinobacteria;c__Actinobacteria;o__Propionibacteriales;f__Propionibacteriaceae;g__Pseudopropionibacterium	0.00 ± 0.00	0.0	0.02 ± 0.00	0.0	4.40E-08	-	-	3.95E-08
k__Bacteria;p__Actinobacteria;c__Actinobacteria;o__Pseudonocardiales;f__Pseudonocardiaceae;g__Actinomycetospora	0.00 ± 0.00	0.0	0.00 ± 0.00	0.0	6.79E-03	-	-	6.89E-03
k__Bacteria;p__Actinobacteria;c__Actinobacteria;o__Pseudonocardiales;f__Pseudonocardiaceae;g__Saccharopolyspora	0.00 ± 0.00	0.0	0.00 ± 0.00	0.0	NA	-	-	-
k__Bacteria;p__Actinobacteria;c__Actinobacteria;o__Streptosporangiales;f__Thermomonosporaceae;g__Actinoallomurus	0.00 ± 0.00	0.0	0.00 ± 0.00	0.0	NA	-	-	-
k__Bacteria;p__Actinobacteria;c__Coriobacteriia;o__Coriobacteriales;f__Atopobiaceae;g__	0.00 ± 0.00	0.0	0.00 ± 0.00	0.0	2.32E-01	-	-	-
k__Bacteria;p__Actinobacteria;c__Coriobacteriia;o__Coriobacteriales;f__Atopobiaceae;g__Atopobium	0.02 ± 0.01	0.0	0.48 ± 0.04	11.3	2.06E-34	-	3.3622	2.85E-34
k__Bacteria;p__Actinobacteria;c__Coriobacteriia;o__Coriobacteriales;f__Atopobiaceae;g__Olsenella	0.00 ± 0.00	0.0	0.00 ± 0.00	0.0	1.49E-14	-	-	1.74E-14
k__Bacteria;p__Actinobacteria;c__Coriobacteriia;o__Coriobacteriales;f__Coriobacteriaceae;g__	0.00 ± 0.00	0.0	0.00 ± 0.00	0.0	3.40E-10	-	-	3.77E-10
k__Bacteria;p__Actinobacteria;c__Coriobacteriia;o__Coriobacteriales;f__Coriobacteriaceae;g__Collinsella	0.00 ± 0.00	0.0	0.00 ± 0.00	0.0	3.33E-08	-	-	3.68E-08
k__Bacteria;p__Actinobacteria;c__Coriobacteriia;o__Coriobacteriales;f__Coriobacteriaceae;g__Parvibacter	0.00 ± 0.00	0.0	0.00 ± 0.00	0.0	NA	-	-	-

k__Bacteria;p__Actinobacteria;c__Coriobacteriia;o__Coriobacteriales;f__Coriobacteriaceae;g__Senegalimassilia	0.00 ± 0.00	0.0	0.00 ± 0.00	0.0	3.34E-07	-	-	3.57E-07
k__Bacteria;p__Actinobacteria;c__Coriobacteriia;o__Eggerthellales;f__Eggerthellaceae;g__	0.00 ± 0.00	0.0	0.00 ± 0.00	0.0	3.34E-07	-	-	3.57E-07
k__Bacteria;p__Actinobacteria;c__Coriobacteriia;o__Eggerthellales;f__Eggerthellaceae;g__Eggerthella	0.00 ± 0.00	0.0	0.00 ± 0.00	0.0	2.97E-03	-	-	3.02E-03
k__Bacteria;p__Actinobacteria;c__Coriobacteriia;o__Eggerthellales;f__Eggerthellaceae;g__Enterorhabdus	0.00 ± 0.00	0.0	0.00 ± 0.00	0.0	5.74E-04	-	-	5.90E-04
k__Bacteria;p__Actinobacteria;c__Coriobacteriia;o__Eggerthellales;f__Eggerthellaceae;g__Gordonibacter	0.00 ± 0.00	0.0	0.00 ± 0.00	0.0	NA	-	-	-
k__Bacteria;p__Actinobacteria;c__Coriobacteriia;o__Eggerthellales;f__Eggerthellaceae;g__Raoultibacter	0.00 ± 0.00	0.0	0.00 ± 0.00	0.0	2.32E-01	-	-	-
k__Bacteria;p__Actinobacteria;c__Coriobacteriia;o__Eggerthellales;f__Eggerthellaceae;g__Slackia	0.00 ± 0.00	0.0	0.00 ± 0.00	0.0	1.78E-06	-	-	1.89E-06
k__Bacteria;p__Actinobacteria;c__Thermoleophilia;o__Solirubrobacterales;f__Solirubrobacteraceae;g__Solirubrobacter	0.00 ± 0.00	0.0	0.00 ± 0.00	0.0	2.32E-01	-	-	-
k__Bacteria;p__Bacteroidetes;c__;o__;f__;g__	0.00 ± 0.00	0.0	0.00 ± 0.00	0.0	NA	-	-	-
k__Bacteria;p__Bacteroidetes;c__Bacteroidia;o__Bacteroidales;f__;g__	0.00 ± 0.00	0.0	0.00 ± 0.00	0.0	4.90E-05	-	-	5.10E-05
k__Bacteria;p__Bacteroidetes;c__Bacteroidia;o__Bacteroidales;f__Bacteroidaceae;g__	0.01 ± 0.00	0.0	0.00 ± 0.00	0.0	6.16E-34	-	-	8.78E-34
k__Bacteria;p__Bacteroidetes;c__Bacteroidia;o__Bacteroidales;f__Bacteroidaceae;g__Anaerorhabdus	0.00 ± 0.00	0.0	0.00 ± 0.00	0.0	1.11E-04	-	-	1.15E-04
k__Bacteria;p__Bacteroidetes;c__Bacteroidia;o__Bacteroidales;f__Bacteroidaceae;g__Bacteroides	0.09 ± 0.07	1.7	0.28 ± 0.07	6.0	1.37E-04	-	2.9582	1.05E-04
k__Bacteria;p__Bacteroidetes;c__Bacteroidia;o__Bacteroidales;f__Bacteroidales_RF16_group;g__	0.01 ± 0.00	0.0	0.00 ± 0.00	0.0	3.23E-16	-	-	3.84E-16
k__Bacteria;p__Bacteroidetes;c__Bacteroidia;o__Bacteroidales;f__Odoribacteraceae;g__Butyricimonas	0.00 ± 0.00	0.0	0.00 ± 0.00	0.0	3.48E-01	-	-	-
k__Bacteria;p__Bacteroidetes;c__Bacteroidia;o__Bacteroidales;f__Odoribacteraceae;g__Odoribacter	0.00 ± 0.00	0.0	0.00 ± 0.00	0.0	2.65E-02	-	-	2.58E-02
k__Bacteria;p__Bacteroidetes;c__Bacteroidia;o__Bacteroidales;f__Phocaeicola;g__abscessus	0.02 ± 0.01	0.9	0.00 ± 0.00	0.0	1.71E-17	-	-	2.06E-17
k__Bacteria;p__Bacteroidetes;c__Bacteroidia;o__Bacteroidales;f__Porphyromonadaceae;g__	0.09 ± 0.01	0.0	0.11 ± 0.02	1.2	5.97E-01	-	-	-
k__Bacteria;p__Bacteroidetes;c__Bacteroidia;o__Bacteroidales;f__Porphyromonadaceae;g__Barnesiella	0.00 ± 0.00	0.0	0.00 ± 0.00	0.0	6.18E-02	-	-	-
k__Bacteria;p__Bacteroidetes;c__Bacteroidia;o__Bacteroidales;f__Porphyromonadaceae;g__Coprobacter	0.00 ± 0.00	0.0	0.00 ± 0.00	0.0	2.41E-01	-	-	-
k__Bacteria;p__Bacteroidetes;c__Bacteroidia;o__Bacteroidales;f__Porphyromonadaceae;g__Muribaculum	0.00 ± 0.00	0.0	0.00 ± 0.00	0.0	8.18E-03	-	-	8.43E-03
k__Bacteria;p__Bacteroidetes;c__Bacteroidia;o__Bacteroidales;f__Porphyromonadaceae;g__Paludibacter	0.01 ± 0.00	0.0	0.00 ± 0.00	0.0	1.55E-12	-	-	1.77E-12
k__Bacteria;p__Bacteroidetes;c__Bacteroidia;o__Bacteroidales;f__Porphyromonadaceae;g__Parabacteroides	0.02 ± 0.02	0.9	0.04 ± 0.01	0.6	6.28E-04	-	2.0916	8.73E-04
k__Bacteria;p__Bacteroidetes;c__Bacteroidia;o__Bacteroidales;f__Porphyromonadaceae;g__Petrimonas	0.00 ± 0.00	0.0	0.00 ± 0.00	0.0	8.92E-02	-	-	-
**k__Bacteria;p__Bacteroidetes;c__Bacteroidia;o__Bacteroidales;f__Porphyromonadaceae;g__Porphyromonas**	**3.58 ± 0.34**	**73.3**	**4.94 ± 0.35**	**86.9**	**1.22E-03**	**-**	**3.8172**	**9.19E-04**
k__Bacteria;p__Bacteroidetes;c__Bacteroidia;o__Bacteroidales;f__Porphyromonadaceae;g__Proteiniphilum	0.00 ± 0.00	0.0	0.00 ± 0.00	0.0	NA	-	-	-
k__Bacteria;p__Bacteroidetes;c__Bacteroidia;o__Bacteroidales;f__Porphyromonadaceae;g__Tannerella	0.06 ± 0.01	0.0	0.07 ± 0.01	0.0	5.43E-01	-	-	-
k__Bacteria;p__Bacteroidetes;c__Bacteroidia;o__Bacteroidales;f__Prevotellaceae;g__	0.07 ± 0.03	2.6	0.21 ± 0.03	3.0	1.85E-07	-	2.8092	1.14E-07
k__Bacteria;p__Bacteroidetes;c__Bacteroidia;o__Bacteroidales;f__Prevotellaceae;g__Alloprevotella	0.04 ± 0.01	0.0	0.56 ± 0.06	19.6	8.37E-23	-	3.4021	3.05E-23
k__Bacteria;p__Bacteroidetes;c__Bacteroidia;o__Bacteroidales;f__Prevotellaceae;g__Paraprevotella	0.00 ± 0.00	0.0	0.00 ± 0.00	0.0	2.65E-02	-	-	2.58E-02
**k__Bacteria;p__Bacteroidetes;c__Bacteroidia;o__Bacteroidales;f__Prevotellaceae;g__Prevotella**	**1.28 ± 0.21**	**28.4**	**13.13 ± 0.62**	**99.4**	**7.15E-42**	**-**	**4.7702**	**9.45E-42**
**k__Bacteria;p__Bacteroidetes;c__Bacteroidia;o__Bacteroidales;f__Prevotellaceae;g__Prevotellamassilia**	**1.06 ± 0.18**	**26.7**	**1.37 ± 0.13**	**43.5**	**1.39E-05**	**-**	**3.2302**	**1.28E-05**
k__Bacteria;p__Bacteroidetes;c__Bacteroidia;o__Bacteroidales;f__Rikenellaceae;g__	0.01 ± 0.00	0.0	0.01 ± 0.01	0.6	4.14E-11	-	-	4.91E-11
k__Bacteria;p__Bacteroidetes;c__Bacteroidia;o__Bacteroidales;f__Rikenellaceae;g__Alistipes	0.00 ± 0.00	0.0	0.03 ± 0.01	0.0	5.55E-04	-	2.2012	5.22E-04
k__Bacteria;p__Bacteroidetes;c__Bacteroidia;o__Bacteroidales;f__Rikenellaceae;g__Rikenellaceae_RC9_gut_group	0.00 ± 0.00	0.0	0.00 ± 0.00	0.0	4.67E-09	-	-	5.11E-09
k__Bacteria;p__Bacteroidetes;c__Bacteroidia;o__Marinilabiliales;f__Marinilabiliaceae;g__	0.03 ± 0.01	0.0	0.00 ± 0.00	0.0	9.47E-23	2.0971	-	1.24E-22
k__Bacteria;p__Bacteroidetes;c__Bacteroidia;o__Marinilabiliales;f__Prolixibacteraceae;g__Mariniphaga	0.00 ± 0.00	0.0	0.00 ± 0.00	0.0	NA	-	-	-
k__Bacteria;p__Bacteroidetes;c__Chitinophagia;o__Chitinophagales;f__Chitinophagaceae;g__	0.02 ± 0.00	0.0	0.02 ± 0.01	0.6	4.71E-22	-	-	6.52E-22
k__Bacteria;p__Bacteroidetes;c__Chitinophagia;o__Chitinophagales;f__Chitinophagaceae;g__Chitinophaga	0.00 ± 0.00	0.0	0.00 ± 0.00	0.0	NA	-	-	-
k__Bacteria;p__Bacteroidetes;c__Chitinophagia;o__Chitinophagales;f__Chitinophagaceae;g__Filimonas	0.00 ± 0.00	0.0	0.00 ± 0.00	0.0	2.32E-01	-	-	-
k__Bacteria;p__Bacteroidetes;c__Chitinophagia;o__Chitinophagales;f__Chitinophagaceae;g__Flaviaesturariibacter	0.00 ± 0.00	0.0	0.00 ± 0.00	0.0	2.32E-01	-	-	-
k__Bacteria;p__Bacteroidetes;c__Chitinophagia;o__Chitinophagales;f__Chitinophagaceae;g__Flavihumibacter	0.00 ± 0.00	0.0	0.00 ± 0.00	0.0	NA	-	-	-
k__Bacteria;p__Bacteroidetes;c__Chitinophagia;o__Chitinophagales;f__Chitinophagaceae;g__Flavisolibacter	0.00 ± 0.00	0.0	0.00 ± 0.00	0.0	8.92E-02	-	-	-
k__Bacteria;p__Bacteroidetes;c__Chitinophagia;o__Chitinophagales;f__Chitinophagaceae;g__Heliimonas	0.00 ± 0.00	0.0	0.00 ± 0.00	0.0	2.32E-01	-	-	-
k__Bacteria;p__Bacteroidetes;c__Chitinophagia;o__Chitinophagales;f__Chitinophagaceae;g__Niabella	0.00 ± 0.00	0.0	0.00 ± 0.00	0.0	2.32E-01	-	-	-
k__Bacteria;p__Bacteroidetes;c__Chitinophagia;o__Chitinophagales;f__Chitinophagaceae;g__Sediminibacterium	0.00 ± 0.00	0.0	0.00 ± 0.00	0.0	2.32E-01	-	-	-
k__Bacteria;p__Bacteroidetes;c__Chitinophagia;o__Chitinophagales;f__Chitinophagaceae;g__Taibaiella	0.00 ± 0.00	0.0	0.00 ± 0.00	0.0	2.32E-01	-	-	-
k__Bacteria;p__Bacteroidetes;c__Chitinophagia;o__Chitinophagales;f__Chitinophagaceae;g__Terrimonas	0.00 ± 0.00	0.0	0.00 ± 0.00	0.0	2.32E-01	-	-	-
k__Bacteria;p__Bacteroidetes;c__Cytophagia;o__Cytophagales;f__Cytophagaceae;g__	0.00 ± 0.00	0.0	0.00 ± 0.00	0.0	2.32E-01	-	-	-
k__Bacteria;p__Bacteroidetes;c__Cytophagia;o__Cytophagales;f__Cytophagaceae;g__Spirosoma	0.00 ± 0.00	0.0	0.00 ± 0.00	0.0	8.92E-02	-	-	-
k__Bacteria;p__Bacteroidetes;c__Cytophagia;o__Cytophagales;f__Hymenobacteraceae;g__Siccationidurans	0.00 ± 0.00	0.0	0.00 ± 0.00	0.0	1.57E-02	-	-	1.58E-02
k__Bacteria;p__Bacteroidetes;c__Flavobacteriia;o__Flavobacteriales;f__Crocinitomicaceae;g__Fluviicola	0.00 ± 0.00	0.0	0.00 ± 0.00	0.0	NA	-	-	-
k__Bacteria;p__Bacteroidetes;c__Flavobacteriia;o__Flavobacteriales;f__Flavobacteriaceae;g__	0.00 ± 0.00	0.0	0.00 ± 0.00	0.0	6.79E-03	-	-	6.89E-03
k__Bacteria;p__Bacteroidetes;c__Flavobacteriia;o__Flavobacteriales;f__Flavobacteriaceae;g__Bergeyella	0.02 ± 0.00	0.0	0.05 ± 0.01	0.6	2.26E-03	-	2.1451	2.69E-03
**k__Bacteria;p__Bacteroidetes;c__Flavobacteriia;o__Flavobacteriales;f__Flavobacteriaceae;g__Capnocytophaga**	**0.23 ± 0.03**	**4.3**	**1.85 ± 0.12**	**58.9**	**2.61E-35**	**-**	**3.9092**	**1.24E-35**
k__Bacteria;p__Bacteroidetes;c__Flavobacteriia;o__Flavobacteriales;f__Flavobacteriaceae;g__Chryseobacterium	0.46 ± 0.04	10.3	0.44 ± 0.02	5.4	1.57E-01	-	-	-
k__Bacteria;p__Bacteroidetes;c__Flavobacteriia;o__Flavobacteriales;f__Flavobacteriaceae;g__Cloacibacterium	0.00 ± 0.00	0.0	0.21 ± 0.05	4.8	1.02E-08	-	3.0546	8.79E-09
k__Bacteria;p__Bacteroidetes;c__Flavobacteriia;o__Flavobacteriales;f__Flavobacteriaceae;g__Elizabethkingia	0.00 ± 0.00	0.0	0.00 ± 0.00	0.0	4.10E-01	-	-	-
k__Bacteria;p__Bacteroidetes;c__Flavobacteriia;o__Flavobacteriales;f__Flavobacteriaceae;g__Empedobacter	0.00 ± 0.00	0.0	0.00 ± 0.00	0.0	NA	-	-	-
k__Bacteria;p__Bacteroidetes;c__Flavobacteriia;o__Flavobacteriales;f__Flavobacteriaceae;g__Flavobacterium	0.00 ± 0.00	0.0	0.00 ± 0.00	0.0	5.74E-04	-	-	5.90E-04
k__Bacteria;p__Bacteroidetes;c__Flavobacteriia;o__Flavobacteriales;f__Flavobacteriaceae;g__Riemerella	0.00 ± 0.00	0.0	0.00 ± 0.00	0.0	1.57E-02	-	-	1.58E-02
k__Bacteria;p__Bacteroidetes;c__Flavobacteriia;o__Flavobacteriales;f__Flavobacteriaceae;g__Soonwooa	0.00 ± 0.00	0.0	0.00 ± 0.00	0.0	1.57E-02	-	-	1.58E-02
k__Bacteria;p__Bacteroidetes;c__Sphingobacteriia;o__Sphingobacteriales;f__Chitinophagaceae;g__Taibaiella	0.00 ± 0.00	0.0	0.00 ± 0.00	0.0	NA	-	-	-

k__Bacteria;p__Bacteroidetes;c__Sphingobacteriia;o__Sphingobacteriales;f__Lentimicrobiaceae;g__	0.02 ± 0.00	0.0	0.03 ± 0.01	0.0	2.45E-08	-	-	3.05E-08
k__Bacteria;p__Bacteroidetes;c__Sphingobacteriia;o__Sphingobacteriales;f__Sphingobacteriaceae;g__	0.02 ± 0.01	0.0	0.00 ± 0.00	0.0	8.51E-19	-	-	1.04E-18
k__Bacteria;p__Bacteroidetes;c__Sphingobacteriia;o__Sphingobacteriales;f__Sphingobacteriaceae;g__Mucilaginibacter	0.00 ± 0.00	0.0	0.00 ± 0.00	0.0	2.32E-01	-	-	-
k__Bacteria;p__Bacteroidetes;c__Sphingobacteriia;o__Sphingobacteriales;f__Sphingobacteriaceae;g__Pedobacter	0.00 ± 0.00	0.0	0.00 ± 0.00	0.0	NA	-	-	-
k__Bacteria;p__Bacteroidetes;c__Sphingobacteriia;o__Sphingobacteriales;f__Sphingobacteriaceae;g__Sphingobacterium	0.00 ± 0.00	0.0	0.00 ± 0.00	0.0	1.30E-03	-	-	1.33E-03
k__Bacteria;p__Chlamydiae;c__Chlamydiia;o__Parachlamydiales;f__;g__	0.00 ± 0.00	0.0	0.00 ± 0.00	0.0	NA	-	-	-
k__Bacteria;p__Chlamydiae;c__Chlamydiia;o__Parachlamydiales;f__Parachlamydiaceae;g__Protochlamydia	0.00 ± 0.00	0.0	0.00 ± 0.00	0.0	2.32E-01	-	-	-
k__Bacteria;p__Chlorobi;c__Chlorobia;o__Chlorobiales;f__OPB56;g__	0.00 ± 0.00	0.0	0.00 ± 0.00	0.0	3.34E-07	-	-	3.57E-07
k__Bacteria;p__Chloroflexi;c__Anaerolineae;o__Anaerolineales;f__Anaerolineaceae;g__	0.00 ± 0.00	0.0	0.00 ± 0.00	0.0	2.15E-05	-	-	2.25E-05
k__Bacteria;p__Chloroflexi;c__Thermomicrobia;o__Sphaerobacterales;f__Sphaerobacteraceae;g__	0.00 ± 0.00	0.0	0.00 ± 0.00	0.0	NA	-	-	-
k__Bacteria;p__Cyanobacteria;c__Cyanobacteria;o__SubsectionIII;f__FamilyI;g__	0.00 ± 0.00	0.0	0.00 ± 0.00	0.0	2.32E-01	-	-	-
k__Bacteria;p__Cyanobacteria;c__Cyanobacteria;o__SubsectionIV;f__FamilyI;g__Trichormus	0.00 ± 0.00	0.0	0.00 ± 0.00	0.0	NA	-	-	-
k__Bacteria;p__Cyanobacteria;c__Melainabacteria;o__Gastranaerophilales;f__;g__	0.00 ± 0.00	0.0	0.00 ± 0.00	0.0	1.21E-03	-	-	1.25E-03
k__Bacteria;p__Cyanobacteria;c__Melainabacteria;o__Obscuribacterales;f__;g__	0.00 ± 0.00	0.0	0.00 ± 0.00	0.0	4.10E-01	-	-	-
k__Bacteria;p__Deferribacteres;c__Deferribacteres;o__Deferribacterales;f__Deferribacteraceae;g__Seleniivibrio	0.00 ± 0.00	0.0	0.00 ± 0.00	0.0	2.32E-01	-	-	-
k__Bacteria;p__Deinococcus-Thermus;c__Deinococci;o__Deinococcales;f__Deinococcaceae;g__Deinococcus	0.00 ± 0.00	0.0	0.00 ± 0.00	0.0	NA	-	-	-
k__Bacteria;p__Elusimicrobia;c__Elusimicrobia;o__Elusimicrobiales;f__Elusimicrobiaceae;g__	0.00 ± 0.00	0.0	0.00 ± 0.00	0.0	3.68E-02	2.1028	-	3.70E-02
k__Bacteria;p__Elusimicrobia;c__Elusimicrobia;o__Elusimicrobiales;f__Elusimicrobiaceae;g__Elusimicrobium	0.00 ± 0.00	0.0	0.00 ± 0.00	0.0	2.97E-03	-	-	3.02E-03
k__Bacteria;p__Fibrobacteres;c__Fibrobacteria;o__Fibrobacterales;f__Fibrobacteraceae;g__Fibrobacter	0.00 ± 0.00	0.0	0.00 ± 0.00	0.0	8.92E-02	-	-	-
k__Bacteria;p__Firmicutes;c__Bacilli;o__Bacillales;f__Alicyclobacillaceae;g__Alicyclobacillus	0.00 ± 0.00	0.0	0.00 ± 0.00	0.0	3.23E-16	-	-	3.84E-16
k__Bacteria;p__Firmicutes;c__Bacilli;o__Bacillales;f__Bacillaceae;g__	0.00 ± 0.00	0.0	0.00 ± 0.00	0.0	4.37E-10	-	-	4.88E-10
k__Bacteria;p__Firmicutes;c__Bacilli;o__Bacillales;f__Bacillaceae;g__Anoxybacillus	0.00 ± 0.00	0.0	0.02 ± 0.02	0.6	7.55E-02	-	-	-
k__Bacteria;p__Firmicutes;c__Bacilli;o__Bacillales;f__Bacillaceae;g__Bacillus	0.00 ± 0.00	0.0	0.00 ± 0.00	0.0	7.55E-02	-	-	-
k__Bacteria;p__Firmicutes;c__Bacilli;o__Bacillales;f__Bacillaceae;g__Geobacillus	0.00 ± 0.00	0.0	0.01 ± 0.01	0.6	2.41E-01	-	-	-
k__Bacteria;p__Firmicutes;c__Bacilli;o__Bacillales;f__Bacillales;g__Thermicanus	0.00 ± 0.00	0.0	0.01 ± 0.01	0.6	8.00E-01	-	-	-
k__Bacteria;p__Firmicutes;c__Bacilli;o__Bacillales;f__Exiguobacterium;g__	0.00 ± 0.00	0.0	0.00 ± 0.00	0.0	2.32E-01	-	-	-
**k__Bacteria;p__Firmicutes;c__Bacilli;o__Bacillales;f__Family_XI;g__Gemella**	**6.19 ± 0.47**	**95.7**	**0.96 ± 0.06**	**33.3**	**5.52E-39**	**4.3965**	**-**	**6.38E-39**
k__Bacteria;p__Firmicutes;c__Bacilli;o__Bacillales;f__Paenibacillaceae;g__	0.00 ± 0.00	0.0	0.00 ± 0.00	0.0	3.34E-07	-	-	3.57E-07
k__Bacteria;p__Firmicutes;c__Bacilli;o__Bacillales;f__Paenibacillaceae;g__Cohnella	0.00 ± 0.00	0.0	0.00 ± 0.00	0.0	2.32E-01	-	-	-
k__Bacteria;p__Firmicutes;c__Bacilli;o__Bacillales;f__Paenibacillaceae;g__Paenibacillus	0.00 ± 0.00	0.0	0.00 ± 0.00	0.0	NA	-	-	-
k__Bacteria;p__Firmicutes;c__Bacilli;o__Bacillales;f__Planococcaceae;g__Kurthia	0.00 ± 0.00	0.0	0.00 ± 0.00	0.0	6.79E-03	-	-	6.89E-03
k__Bacteria;p__Firmicutes;c__Bacilli;o__Bacillales;f__Staphylococcaceae;g__Jeotgalicoccus	0.00 ± 0.00	0.0	0.00 ± 0.00	0.0	3.68E-02	2.0378	-	3.70E-02
k__Bacteria;p__Firmicutes;c__Bacilli;o__Bacillales;f__Staphylococcaceae;g__Macrococcus	0.00 ± 0.00	0.0	0.00 ± 0.00	0.0	1.57E-02	-	-	1.58E-02
k__Bacteria;p__Firmicutes;c__Bacilli;o__Bacillales;f__Staphylococcaceae;g__Staphylococcus	0.00 ± 0.00	0.0	0.02 ± 0.01	0.0	3.83E-01	-	-	-
k__Bacteria;p__Firmicutes;c__Bacilli;o__Bacillales;f__Thermoactinomycetaceae;g__	0.00 ± 0.00	0.0	0.00 ± 0.00	0.0	8.92E-02	-	-	-
k__Bacteria;p__Firmicutes;c__Bacilli;o__Lactobacillales;f__Aerococcaceae;g__	0.00 ± 0.00	0.0	0.00 ± 0.00	0.0	2.32E-01	-	-	-
k__Bacteria;p__Firmicutes;c__Bacilli;o__Lactobacillales;f__Aerococcaceae;g__Abiotrophia	0.02 ± 0.00	0.0	0.13 ± 0.02	1.2	9.53E-02	-	-	-
k__Bacteria;p__Firmicutes;c__Bacilli;o__Lactobacillales;f__Aerococcaceae;g__Aerococcus	0.00 ± 0.00	0.0	0.00 ± 0.00	0.0	1.11E-04	-	-	1.15E-04
k__Bacteria;p__Firmicutes;c__Bacilli;o__Lactobacillales;f__Aerococcaceae;g__Dolosicoccus	0.00 ± 0.00	0.0	0.00 ± 0.00	0.0	2.32E-01	-	-	-
k__Bacteria;p__Firmicutes;c__Bacilli;o__Lactobacillales;f__Aerococcaceae;g__Eremococcus	0.00 ± 0.00	0.0	0.00 ± 0.00	0.0	1.57E-02	-	-	1.58E-02
k__Bacteria;p__Firmicutes;c__Bacilli;o__Lactobacillales;f__Aerococcaceae;g__Facklamia	0.00 ± 0.00	0.0	0.00 ± 0.00	0.0	4.10E-06	-	-	4.33E-06
k__Bacteria;p__Firmicutes;c__Bacilli;o__Lactobacillales;f__Carnobacteriaceae;g__Alkalibacterium	0.00 ± 0.00	0.0	0.00 ± 0.00	0.0	NA	-	-	-
k__Bacteria;p__Firmicutes;c__Bacilli;o__Lactobacillales;f__Carnobacteriaceae;g__Atopostipes	0.00 ± 0.00	0.0	0.00 ± 0.00	0.0	1.57E-02	-	-	1.58E-02
k__Bacteria;p__Firmicutes;c__Bacilli;o__Lactobacillales;f__Carnobacteriaceae;g__Dolosigranulum	0.00 ± 0.00	0.0	0.00 ± 0.00	0.0	3.68E-02	-	-	3.70E-02
**k__Bacteria;p__Firmicutes;c__Bacilli;o__Lactobacillales;f__Carnobacteriaceae;g__Granulicatella**	**3.35 ± 0.26**	**82.8**	**1.45 ± 0.08**	**58.9**	**2.79E-11**	**3.9638**	**-**	**1.49E-11**
k__Bacteria;p__Firmicutes;c__Bacilli;o__Lactobacillales;f__Enterococcaceae;g__Enterococcus	0.00 ± 0.00	0.0	0.00 ± 0.00	0.0	1.67E-01	-	-	-
k__Bacteria;p__Firmicutes;c__Bacilli;o__Lactobacillales;f__Lactobacillaceae;g__Lactobacillus	0.08 ± 0.02	0.9	0.02 ± 0.01	0.6	1.86E-29	2.4818	-	2.77E-29
k__Bacteria;p__Firmicutes;c__Bacilli;o__Lactobacillales;f__Lactobacillaceae;g__Pediococcus	0.00 ± 0.00	0.0	0.00 ± 0.00	0.0	NA	-	-	-
k__Bacteria;p__Firmicutes;c__Bacilli;o__Lactobacillales;f__Leuconostocaceae;g__Leuconostoc	0.00 ± 0.00	0.0	0.00 ± 0.00	0.0	3.68E-02	-	-	3.70E-02
k__Bacteria;p__Firmicutes;c__Bacilli;o__Lactobacillales;f__Leuconostocaceae;g__Weissella	0.00 ± 0.00	0.0	0.00 ± 0.00	0.0	4.90E-05	-	-	5.10E-05
k__Bacteria;p__Firmicutes;c__Bacilli;o__Lactobacillales;f__Streptococcaceae;g__Lactococcus	0.00 ± 0.00	0.0	0.02 ± 0.01	0.6	8.01E-02	-	-	-
**k__Bacteria;p__Firmicutes;c__Bacilli;o__Lactobacillales;f__Streptococcaceae;g__Streptococcus**	**38.71 ± 1.41**	**100.0**	**15.31 ± 0.67**	**100.0**	**7.40E-33**	**5.0712**	**-**	**9.00E-33**
k__Bacteria;p__Firmicutes;c__Clostridia;o__Clostridiales;f__;g__	0.01 ± 0.00	0.0	0.11 ± 0.02	0.6	1.90E-05	-	2.7231	1.57E-05
k__Bacteria;p__Firmicutes;c__Clostridia;o__Clostridiales;f__Bacteroides;g__pectinophilus	0.00 ± 0.00	0.0	0.00 ± 0.00	0.0	2.32E-01	-	-	-
k__Bacteria;p__Firmicutes;c__Clostridia;o__Clostridiales;f__Caldicoprobacteraceae;g__	0.00 ± 0.00	0.0	0.00 ± 0.00	0.0	3.68E-01	-	-	-
k__Bacteria;p__Firmicutes;c__Clostridia;o__Clostridiales;f__Christensenellaceae;g__	0.00 ± 0.00	0.0	0.00 ± 0.00	0.0	1.10E-05	-	-	1.17E-05
k__Bacteria;p__Firmicutes;c__Clostridia;o__Clostridiales;f__Christensenellaceae;g__Christensenella	0.00 ± 0.00	0.0	0.00 ± 0.00	0.0	NA	-	-	-
k__Bacteria;p__Firmicutes;c__Clostridia;o__Clostridiales;f__Christensenellaceae;g__Christensenellaceae_R-7_group	0.01 ± 0.00	0.0	0.01 ± 0.01	0.0	1.73E-10	-	-	1.98E-10
**k__Bacteria;p__Firmicutes;c__Clostridia;o__Clostridiales;f__Clostridiaceae;g__**	**0.02 ± 0.00**	**0.0**	**1.88 ± 0.27**	**39.9**	**4.92E-29**	**-**	**3.9466**	**1.70E-29**
k__Bacteria;p__Firmicutes;c__Clostridia;o__Clostridiales;f__Clostridiaceae;g__Beduini	0.00 ± 0.00	0.0	0.00 ± 0.00	0.0	3.68E-02	-	-	3.70E-02
k__Bacteria;p__Firmicutes;c__Clostridia;o__Clostridiales;f__Clostridiaceae;g__Butyricicoccus	0.01 ± 0.00	0.0	0.00 ± 0.00	0.0	5.77E-11	-	-	6.46E-11
k__Bacteria;p__Firmicutes;c__Clostridia;o__Clostridiales;f__Clostridiaceae;g__Caloramator	0.00 ± 0.00	0.0	0.00 ± 0.00	0.0	NA	-	-	-
k__Bacteria;p__Firmicutes;c__Clostridia;o__Clostridiales;f__Clostridiaceae;g__Clostridium	0.03 ± 0.00	0.0	0.00 ± 0.00	0.0	7.97E-38	2.1374	-	1.18E-37

k__Bacteria;p__Firmicutes;c__Clostridia;o__Clostridiales;f__Clostridiaceae;g__Hungatella	0.04 ± 0.01	0.0	0.00 ± 0.00	0.0	3.18E-41	2.2945	-	4.88E-41
k__Bacteria;p__Firmicutes;c__Clostridia;o__Clostridiales;f__Clostridiaceae;g__Lactonifactor	0.01 ± 0.00	0.0	0.00 ± 0.00	0.0	2.52E-14	-	-	3.00E-14
k__Bacteria;p__Firmicutes;c__Clostridia;o__Clostridiales;f__Clostridiaceae;g__Oxobacter	0.00 ± 0.00	0.0	0.00 ± 0.00	0.0	2.32E-01	-	-	-
k__Bacteria;p__Firmicutes;c__Clostridia;o__Clostridiales;f__Clostridiales;g__	0.00 ± 0.00	0.0	0.03 ± 0.01	0.0	1.09E-01	-	-	-
k__Bacteria;p__Firmicutes;c__Clostridia;o__Clostridiales;f__Clostridiales;g__Anaerovorax	0.01 ± 0.00	0.0	0.04 ± 0.01	0.6	8.84E-05	-	2.1485	1.01E-04
k__Bacteria;p__Firmicutes;c__Clostridia;o__Clostridiales;f__Clostridiales;g__Casaltella	0.00 ± 0.00	0.0	0.00 ± 0.00	0.0	NA	-	-	-
k__Bacteria;p__Firmicutes;c__Clostridia;o__Clostridiales;f__Clostridiales;g__Emergencia	0.00 ± 0.00	0.0	0.00 ± 0.00	0.0	3.36E-02	-	-	3.41E-02
k__Bacteria;p__Firmicutes;c__Clostridia;o__Clostridiales;f__Clostridiales;g__Eubacterium	0.01 ± 0.00	0.0	0.14 ± 0.01	0.6	1.82E-16	-	2.8357	2.62E-16
k__Bacteria;p__Firmicutes;c__Clostridia;o__Clostridiales;f__Clostridiales;g__Ihubacter	0.00 ± 0.00	0.0	0.00 ± 0.00	0.0	6.99E-03	-	-	7.14E-03
k__Bacteria;p__Firmicutes;c__Clostridia;o__Clostridiales;f__Clostridiales;g__Mogibacterium	0.00 ± 0.00	0.0	0.09 ± 0.01	0.6	2.60E-16	-	2.6637	3.79E-16
k__Bacteria;p__Firmicutes;c__Clostridia;o__Clostridiales;f__Defluviitaleaceae;g__	0.00 ± 0.00	0.0	0.00 ± 0.00	0.0	2.32E-01	-	-	-
k__Bacteria;p__Firmicutes;c__Clostridia;o__Clostridiales;f__Defluviitaleaceae;g__Defluviitalea	0.01 ± 0.00	0.0	0.02 ± 0.01	0.0	4.55E-08	-	-	5.69E-08
k__Bacteria;p__Firmicutes;c__Clostridia;o__Clostridiales;f__Defluviitaleaceae;g__Vallitalea	0.00 ± 0.00	0.0	0.00 ± 0.00	0.0	NA	-	-	-
k__Bacteria;p__Firmicutes;c__Clostridia;o__Clostridiales;f__Eubacteriaceae;g__	0.00 ± 0.00	0.0	0.00 ± 0.00	0.0	NA	-	-	-
k__Bacteria;p__Firmicutes;c__Clostridia;o__Clostridiales;f__Eubacteriaceae;g__Anaerofustis	0.00 ± 0.00	0.0	0.00 ± 0.00	0.0	NA	-	-	-
k__Bacteria;p__Firmicutes;c__Clostridia;o__Clostridiales;f__Eubacteriaceae;g__Eubacterium	0.03 ± 0.01	0.0	0.00 ± 0.00	0.0	1.92E-26	2.0159	-	2.69E-26
k__Bacteria;p__Firmicutes;c__Clostridia;o__Clostridiales;f__Family_XI;g__Murdochiella	0.00 ± 0.00	0.0	0.00 ± 0.00	0.0	NA	-	-	-
k__Bacteria;p__Firmicutes;c__Clostridia;o__Clostridiales;f__Family_XIII;g__Family_XIII_UCG-001	0.00 ± 0.00	0.0	0.00 ± 0.00	0.0	NA	-	-	-
k__Bacteria;p__Firmicutes;c__Clostridia;o__Clostridiales;f__Fenollaria;g__massiliensis	0.00 ± 0.00	0.0	0.00 ± 0.00	0.0	2.32E-01	-	-	-
k__Bacteria;p__Firmicutes;c__Clostridia;o__Clostridiales;f__Flavonifractor;g__	0.00 ± 0.00	0.0	0.00 ± 0.00	0.0	2.32E-01	-	-	-
k__Bacteria;p__Firmicutes;c__Clostridia;o__Clostridiales;f__Flavonifractor;g__plautii	0.00 ± 0.00	0.0	0.00 ± 0.00	0.0	2.32E-01	-	-	-
k__Bacteria;p__Firmicutes;c__Clostridia;o__Clostridiales;f__Flintibacter;g__	0.00 ± 0.00	0.0	0.00 ± 0.00	0.0	4.90E-05	-	-	5.10E-05
k__Bacteria;p__Firmicutes;c__Clostridia;o__Clostridiales;f__Gracilibacteraceae;g__	0.00 ± 0.00	0.0	0.00 ± 0.00	0.0	1.01E-01	-	-	-
k__Bacteria;p__Firmicutes;c__Clostridia;o__Clostridiales;f__Gracilibacteraceae;g__Gracilibacter	0.00 ± 0.00	0.0	0.00 ± 0.00	0.0	3.68E-01	-	-	-
k__Bacteria;p__Firmicutes;c__Clostridia;o__Clostridiales;f__Heliobacteriaceae;g__	0.00 ± 0.00	0.0	0.00 ± 0.00	0.0	3.68E-02	-	-	3.70E-02
k__Bacteria;p__Firmicutes;c__Clostridia;o__Clostridiales;f__Howardella;g__	0.01 ± 0.00	0.0	0.00 ± 0.00	0.0	4.46E-19	-	-	5.57E-19
k__Bacteria;p__Firmicutes;c__Clostridia;o__Clostridiales;f__Howardella;g__ureilytica	0.00 ± 0.00	0.0	0.00 ± 0.00	0.0	2.32E-01	-	-	-
k__Bacteria;p__Firmicutes;c__Clostridia;o__Clostridiales;f__Intestinimonas;g__	0.00 ± 0.00	0.0	0.00 ± 0.00	0.0	8.48E-11	-	-	9.74E-11
k__Bacteria;p__Firmicutes;c__Clostridia;o__Clostridiales;f__Lachnospiraceae;g__	0.10 ± 0.03	2.6	0.04 ± 0.01	1.2	3.87E-17	2.4326	-	5.72E-17
k__Bacteria;p__Firmicutes;c__Clostridia;o__Clostridiales;f__Lachnospiraceae;g__Acetatifactor	0.00 ± 0.00	0.0	0.00 ± 0.00	0.0	NA	-	-	-
k__Bacteria;p__Firmicutes;c__Clostridia;o__Clostridiales;f__Lachnospiraceae;g__Acetitomaculum	0.00 ± 0.00	0.0	0.00 ± 0.00	0.0	NA	-	-	-
k__Bacteria;p__Firmicutes;c__Clostridia;o__Clostridiales;f__Lachnospiraceae;g__Anaerobium	0.00 ± 0.00	0.0	0.00 ± 0.00	0.0	6.79E-03	-	-	6.89E-03
k__Bacteria;p__Firmicutes;c__Clostridia;o__Clostridiales;f__Lachnospiraceae;g__Anaerocolumna	0.00 ± 0.00	0.0	0.00 ± 0.00	0.0	NA	-	-	-
k__Bacteria;p__Firmicutes;c__Clostridia;o__Clostridiales;f__Lachnospiraceae;g__Anaerosporobacter	0.00 ± 0.00	0.0	0.00 ± 0.00	0.0	2.15E-05	-	-	2.25E-05
k__Bacteria;p__Firmicutes;c__Clostridia;o__Clostridiales;f__Lachnospiraceae;g__Anaerostipes	0.01 ± 0.00	0.0	0.00 ± 0.00	0.0	5.00E-21	-	-	6.27E-21
k__Bacteria;p__Firmicutes;c__Clostridia;o__Clostridiales;f__Lachnospiraceae;g__Blautia	0.04 ± 0.01	0.0	0.00 ± 0.00	0.0	5.22E-32	2.2498	-	7.68E-32
k__Bacteria;p__Firmicutes;c__Clostridia;o__Clostridiales;f__Lachnospiraceae;g__Butyrivibrio	0.01 ± 0.00	0.0	0.04 ± 0.01	0.0	7.41E-01	-	-	-
k__Bacteria;p__Firmicutes;c__Clostridia;o__Clostridiales;f__Lachnospiraceae;g__Catonella	0.07 ± 0.02	1.7	0.53 ± 0.05	11.9	4.90E-29	-	3.3392	5.74E-29
k__Bacteria;p__Firmicutes;c__Clostridia;o__Clostridiales;f__Lachnospiraceae;g__Cellulosilyticum	0.00 ± 0.00	0.0	0.00 ± 0.00	0.0	2.32E-01	-	-	-
k__Bacteria;p__Firmicutes;c__Clostridia;o__Clostridiales;f__Lachnospiraceae;g__Clostridium	0.06 ± 0.02	0.9	0.00 ± 0.00	0.0	1.68E-33	2.4560	-	2.46E-33
k__Bacteria;p__Firmicutes;c__Clostridia;o__Clostridiales;f__Lachnospiraceae;g__Coprococcus	0.01 ± 0.00	0.0	0.00 ± 0.00	0.0	3.13E-10	-	-	3.62E-10
k__Bacteria;p__Firmicutes;c__Clostridia;o__Clostridiales;f__Lachnospiraceae;g__Cuneatibacter	0.00 ± 0.00	0.0	0.00 ± 0.00	0.0	1.57E-02	-	-	1.58E-02
k__Bacteria;p__Firmicutes;c__Clostridia;o__Clostridiales;f__Lachnospiraceae;g__Desulfotomaculum	0.00 ± 0.00	0.0	0.00 ± 0.00	0.0	4.71E-12	-	-	5.38E-12
k__Bacteria;p__Firmicutes;c__Clostridia;o__Clostridiales;f__Lachnospiraceae;g__Dorea	0.01 ± 0.00	0.0	0.00 ± 0.00	0.0	7.24E-23	-	-	9.25E-23
k__Bacteria;p__Firmicutes;c__Clostridia;o__Clostridiales;f__Lachnospiraceae;g__Eisenbergiella	0.00 ± 0.00	0.0	0.00 ± 0.00	0.0	1.54E-02	-	-	1.57E-02
k__Bacteria;p__Firmicutes;c__Clostridia;o__Clostridiales;f__Lachnospiraceae;g__Eubacterium	0.01 ± 0.00	0.0	0.01 ± 0.00	0.0	4.77E-10	-	-	5.66E-10
k__Bacteria;p__Firmicutes;c__Clostridia;o__Clostridiales;f__Lachnospiraceae;g__Frisingicoccus	0.00 ± 0.00	0.0	0.00 ± 0.00	0.0	NA	-	-	-
k__Bacteria;p__Firmicutes;c__Clostridia;o__Clostridiales;f__Lachnospiraceae;g__Fusicatenibacter	0.00 ± 0.00	0.0	0.00 ± 0.00	0.0	6.09E-05	-	-	6.51E-05
k__Bacteria;p__Firmicutes;c__Clostridia;o__Clostridiales;f__Lachnospiraceae;g__Hespellia	0.00 ± 0.00	0.0	0.00 ± 0.00	0.0	NA	-	-	-
k__Bacteria;p__Firmicutes;c__Clostridia;o__Clostridiales;f__Lachnospiraceae;g__Johnsonella	0.01 ± 0.00	0.0	0.00 ± 0.00	0.0	1.01E-25	-	-	1.33E-25
k__Bacteria;p__Firmicutes;c__Clostridia;o__Clostridiales;f__Lachnospiraceae;g__Lachnoanaerobaculum	0.02 ± 0.00	0.0	0.58 ± 0.03	12.5	1.71E-41	-	3.4324	2.27E-41
k__Bacteria;p__Firmicutes;c__Clostridia;o__Clostridiales;f__Lachnospiraceae;g__Lachnospira	0.00 ± 0.00	0.0	0.00 ± 0.00	0.0	1.67E-01	-	-	-
k__Bacteria;p__Firmicutes;c__Clostridia;o__Clostridiales;f__Lachnospiraceae;g__Lachnospiraceae_ND3007_group	0.00 ± 0.00	0.0	0.00 ± 0.00	0.0	8.92E-02	-	-	-
k__Bacteria;p__Firmicutes;c__Clostridia;o__Clostridiales;f__Lachnospiraceae;g__Moryella	0.02 ± 0.01	0.0	0.13 ± 0.02	2.4	8.26E-05	-	2.7621	6.83E-05
k__Bacteria;p__Firmicutes;c__Clostridia;o__Clostridiales;f__Lachnospiraceae;g__Muricomes	0.00 ± 0.00	0.0	0.00 ± 0.00	0.0	NA	-	-	-
k__Bacteria;p__Firmicutes;c__Clostridia;o__Clostridiales;f__Lachnospiraceae;g__Murimonas	0.00 ± 0.00	0.0	0.00 ± 0.00	0.0	3.41E-02	-	-	3.46E-02
**k__Bacteria;p__Firmicutes;c__Clostridia;o__Clostridiales;f__Lachnospiraceae;g__Oribacterium**	**0.01 ± 0.00**	**0.0**	**2.96 ± 0.26**	**80.4**	**1.40E-46**	**-**	**4.1621**	**1.76E-46**
k__Bacteria;p__Firmicutes;c__Clostridia;o__Clostridiales;f__Lachnospiraceae;g__Pseudobutyrivibrio	0.00 ± 0.00	0.0	0.00 ± 0.00	0.0	2.32E-01	-	-	-
k__Bacteria;p__Firmicutes;c__Clostridia;o__Clostridiales;f__Lachnospiraceae;g__Roseburia	0.01 ± 0.00	0.0	0.00 ± 0.00	0.0	2.04E-12	-	-	2.38E-12
k__Bacteria;p__Firmicutes;c__Clostridia;o__Clostridiales;f__Lachnospiraceae;g__Ruminococcus	0.00 ± 0.00	0.0	0.01 ± 0.00	0.0	1.15E-02	-	-	1.12E-02
k__Bacteria;p__Firmicutes;c__Clostridia;o__Clostridiales;f__Lachnospiraceae;g__Shuttleworthia	0.01 ± 0.00	0.0	0.02 ± 0.01	0.0	8.87E-01	-	-	-
k__Bacteria;p__Firmicutes;c__Clostridia;o__Clostridiales;f__Lachnospiraceae;g__Stomatobaculum	0.00 ± 0.00	0.0	0.49 ± 0.04	12.5	1.47E-40	-	3.3752	1.91E-40
k__Bacteria;p__Firmicutes;c__Clostridia;o__Clostridiales;f__Lachnospiraceae;g__Tyzzerella	0.00 ± 0.00	0.0	0.00 ± 0.00	0.0	NA	-	-	-

k__Bacteria;p__Firmicutes;c__Clostridia;o__Clostridiales;f__Levyella;g__	0.00 ± 0.00	0.0	0.00 ± 0.00	0.0	2.32E-01	-	-	-
k__Bacteria;p__Firmicutes;c__Clostridia;o__Clostridiales;f__Oscillospiraceae;g__	0.00 ± 0.00	0.0	0.00 ± 0.00	0.0	2.32E-01	-	-	-
k__Bacteria;p__Firmicutes;c__Clostridia;o__Clostridiales;f__Oscillospiraceae;g__Oscillibacter	0.02 ± 0.01	0.9	0.00 ± 0.00	0.0	4.23E-12	-	-	5.10E-12
k__Bacteria;p__Firmicutes;c__Clostridia;o__Clostridiales;f__Peptococcaceae;g__	0.00 ± 0.00	0.0	0.10 ± 0.02	1.8	8.71E-12	-	2.6997	6.56E-12
k__Bacteria;p__Firmicutes;c__Clostridia;o__Clostridiales;f__Peptococcaceae;g__Peptococcus	0.01 ± 0.00	0.0	0.04 ± 0.01	0.0	1.52E-01	-	-	-
k__Bacteria;p__Firmicutes;c__Clostridia;o__Clostridiales;f__Peptostreptococcaceae;g__	0.00 ± 0.00	0.0	0.00 ± 0.00	0.0	4.10E-06	-	-	4.33E-06
k__Bacteria;p__Firmicutes;c__Clostridia;o__Clostridiales;f__Peptostreptococcaceae;g__Clostridioides	0.00 ± 0.00	0.0	0.00 ± 0.00	0.0	NA	-	-	-
k__Bacteria;p__Firmicutes;c__Clostridia;o__Clostridiales;f__Peptostreptococcaceae;g__Eubacterium	0.06 ± 0.01	0.0	0.18 ± 0.02	2.4	4.93E-01	-	-	-
k__Bacteria;p__Firmicutes;c__Clostridia;o__Clostridiales;f__Peptostreptococcaceae;g__Filifactor	0.01 ± 0.00	0.0	0.03 ± 0.01	0.0	8.70E-05	-	-	1.01E-04
k__Bacteria;p__Firmicutes;c__Clostridia;o__Clostridiales;f__Peptostreptococcaceae;g__Intestinibacter	0.01 ± 0.00	0.0	0.00 ± 0.00	0.0	1.40E-10	-	-	1.57E-10
k__Bacteria;p__Firmicutes;c__Clostridia;o__Clostridiales;f__Peptostreptococcaceae;g__Peptostreptococcus	0.01 ± 0.00	0.0	0.35 ± 0.05	6.5	1.20E-24	-	3.2435	6.20E-25
k__Bacteria;p__Firmicutes;c__Clostridia;o__Clostridiales;f__Peptostreptococcaceae;g__Romboutsia	0.00 ± 0.00	0.0	0.00 ± 0.00	0.0	1.02E-01	-	-	-
k__Bacteria;p__Firmicutes;c__Clostridia;o__Clostridiales;f__Peptostreptococcaceae;g__Terrisporobacter	0.00 ± 0.00	0.0	0.00 ± 0.00	0.0	2.53E-04	-	-	2.61E-04
k__Bacteria;p__Firmicutes;c__Clostridia;o__Clostridiales;f__Pseudoflavonifractor;g__	0.00 ± 0.00	0.0	0.00 ± 0.00	0.0	8.92E-02	-	-	-
k__Bacteria;p__Firmicutes;c__Clostridia;o__Clostridiales;f__Pseudoflavonifractor;g__capillosus	0.00 ± 0.00	0.0	0.00 ± 0.00	0.0	6.79E-03	-	-	6.89E-03
k__Bacteria;p__Firmicutes;c__Clostridia;o__Clostridiales;f__Ruminococcaceae;g__	0.22 ± 0.04	4.3	0.19 ± 0.02	3.0	3.81E-01	-	-	-
k__Bacteria;p__Firmicutes;c__Clostridia;o__Clostridiales;f__Ruminococcaceae;g__Acetanaerobacterium	0.00 ± 0.00	0.0	0.00 ± 0.00	0.0	1.67E-01	-	-	-
k__Bacteria;p__Firmicutes;c__Clostridia;o__Clostridiales;f__Ruminococcaceae;g__Acetivibrio	0.00 ± 0.00	0.0	0.00 ± 0.00	0.0	1.67E-01	-	-	-
k__Bacteria;p__Firmicutes;c__Clostridia;o__Clostridiales;f__Ruminococcaceae;g__Acutalibacter	0.00 ± 0.00	0.0	0.00 ± 0.00	0.0	NA	-	-	-
k__Bacteria;p__Firmicutes;c__Clostridia;o__Clostridiales;f__Ruminococcaceae;g__Anaerobacterium	0.00 ± 0.00	0.0	0.00 ± 0.00	0.0	3.68E-02	-	-	3.70E-02
k__Bacteria;p__Firmicutes;c__Clostridia;o__Clostridiales;f__Ruminococcaceae;g__Anaerofilum	0.00 ± 0.00	0.0	0.00 ± 0.00	0.0	2.53E-04	-	-	2.61E-04
k__Bacteria;p__Firmicutes;c__Clostridia;o__Clostridiales;f__Ruminococcaceae;g__Anaeromassilibacillus	0.00 ± 0.00	0.0	0.00 ± 0.00	0.0	NA	-	-	-
k__Bacteria;p__Firmicutes;c__Clostridia;o__Clostridiales;f__Ruminococcaceae;g__Anaerotruncus	0.00 ± 0.00	0.0	0.00 ± 0.00	0.0	1.54E-02	-	-	1.57E-02
k__Bacteria;p__Firmicutes;c__Clostridia;o__Clostridiales;f__Ruminococcaceae;g__Caproiciproducens	0.00 ± 0.00	0.0	0.00 ± 0.00	0.0	1.57E-02	-	-	1.58E-02
k__Bacteria;p__Firmicutes;c__Clostridia;o__Clostridiales;f__Ruminococcaceae;g__Clostridium	0.01 ± 0.00	0.0	0.00 ± 0.00	0.0	6.10E-17	-	-	7.38E-17
k__Bacteria;p__Firmicutes;c__Clostridia;o__Clostridiales;f__Ruminococcaceae;g__Drancourtella	0.00 ± 0.00	0.0	0.00 ± 0.00	0.0	NA	-	-	-
k__Bacteria;p__Firmicutes;c__Clostridia;o__Clostridiales;f__Ruminococcaceae;g__Ethanoligenens	0.00 ± 0.00	0.0	0.00 ± 0.00	0.0	1.11E-04	-	-	1.15E-04
k__Bacteria;p__Firmicutes;c__Clostridia;o__Clostridiales;f__Ruminococcaceae;g__Eubacterium	0.00 ± 0.00	0.0	0.00 ± 0.00	0.0	1.98E-03	-	-	2.06E-03
k__Bacteria;p__Firmicutes;c__Clostridia;o__Clostridiales;f__Ruminococcaceae;g__Faecalibacterium	0.05 ± 0.01	0.0	0.03 ± 0.01	0.6	7.67E-25	2.1484	-	1.15E-24
k__Bacteria;p__Firmicutes;c__Clostridia;o__Clostridiales;f__Ruminococcaceae;g__Fastidiosipila	0.00 ± 0.00	0.0	0.00 ± 0.00	0.0	2.32E-01	-	-	-
k__Bacteria;p__Firmicutes;c__Clostridia;o__Clostridiales;f__Ruminococcaceae;g__Gemmiger	0.01 ± 0.01	0.0	0.00 ± 0.00	0.0	7.75E-10	-	-	8.89E-10
k__Bacteria;p__Firmicutes;c__Clostridia;o__Clostridiales;f__Ruminococcaceae;g__Neglecta	0.00 ± 0.00	0.0	0.00 ± 0.00	0.0	1.67E-01	-	-	-
k__Bacteria;p__Firmicutes;c__Clostridia;o__Clostridiales;f__Ruminococcaceae;g__Papillibacter	0.00 ± 0.00	0.0	0.00 ± 0.00	0.0	6.79E-03	2.0510	-	6.89E-03
k__Bacteria;p__Firmicutes;c__Clostridia;o__Clostridiales;f__Ruminococcaceae;g__Phocea	0.00 ± 0.00	0.0	0.00 ± 0.00	0.0	NA	-	-	-
k__Bacteria;p__Firmicutes;c__Clostridia;o__Clostridiales;f__Ruminococcaceae;g__Ruminiclostridium	0.00 ± 0.00	0.0	0.00 ± 0.00	0.0	8.92E-02	-	-	-
k__Bacteria;p__Firmicutes;c__Clostridia;o__Clostridiales;f__Ruminococcaceae;g__Ruminococcus	0.02 ± 0.00	0.0	0.00 ± 0.00	0.0	7.15E-17	-	-	9.15E-17
k__Bacteria;p__Firmicutes;c__Clostridia;o__Clostridiales;f__Ruminococcaceae;g__Saccharofermentans	0.00 ± 0.00	0.0	0.00 ± 0.00	0.0	3.34E-07	-	-	3.57E-07
k__Bacteria;p__Firmicutes;c__Clostridia;o__Clostridiales;f__Ruminococcaceae;g__Sporobacter	0.01 ± 0.00	0.0	0.00 ± 0.00	0.0	1.85E-20	-	-	2.32E-20
k__Bacteria;p__Firmicutes;c__Clostridia;o__Clostridiales;f__Ruminococcaceae;g__Subdoligranulum	0.00 ± 0.00	0.0	0.00 ± 0.00	0.0	2.32E-01	-	-	-
k__Bacteria;p__Firmicutes;c__Clostridia;o__Clostridiales;f__Syntrophomonadaceae;g__	0.00 ± 0.00	0.0	0.00 ± 0.00	0.0	2.32E-01	-	-	-
k__Bacteria;p__Firmicutes;c__Clostridia;o__Clostridiales;f__Syntrophomonadaceae;g__Pelospora	0.00 ± 0.00	0.0	0.00 ± 0.00	0.0	8.92E-02	-	-	-
k__Bacteria;p__Firmicutes;c__Clostridia;o__Clostridiales;f__Syntrophomonadaceae;g__Syntrophomonas	0.00 ± 0.00	0.0	0.00 ± 0.00	0.0	NA	-	-	-
k__Bacteria;p__Firmicutes;c__Clostridia;o__Thermoanaerobacterales;f__Thermoanaerobacteraceae;g__	0.00 ± 0.00	0.0	0.00 ± 0.00	0.0	NA	-	-	-
k__Bacteria;p__Firmicutes;c__Erysipelotrichia;o__Erysipelotrichales;f__Erysipelotrichaceae;g__	0.01 ± 0.00	0.0	0.00 ± 0.00	0.0	3.84E-07	-	-	4.13E-07
k__Bacteria;p__Firmicutes;c__Erysipelotrichia;o__Erysipelotrichales;f__Erysipelotrichaceae;g__Bulleidia	0.01 ± 0.00	0.0	0.00 ± 0.00	0.0	9.73E-14	-	-	1.13E-13
k__Bacteria;p__Firmicutes;c__Erysipelotrichia;o__Erysipelotrichales;f__Erysipelotrichaceae;g__Catenibacterium	0.02 ± 0.00	0.0	0.00 ± 0.00	0.0	1.01E-25	2.0266	-	1.33E-25
k__Bacteria;p__Firmicutes;c__Erysipelotrichia;o__Erysipelotrichales;f__Erysipelotrichaceae;g__Clostridium	0.00 ± 0.00	0.0	0.00 ± 0.00	0.0	8.21E-11	-	-	9.43E-11
k__Bacteria;p__Firmicutes;c__Erysipelotrichia;o__Erysipelotrichales;f__Erysipelotrichaceae;g__Coprobacillus	0.00 ± 0.00	0.0	0.00 ± 0.00	0.0	NA	-	-	-
k__Bacteria;p__Firmicutes;c__Erysipelotrichia;o__Erysipelotrichales;f__Erysipelotrichaceae;g__Dielma	0.00 ± 0.00	0.0	0.00 ± 0.00	0.0	NA	-	-	-
k__Bacteria;p__Firmicutes;c__Erysipelotrichia;o__Erysipelotrichales;f__Erysipelotrichaceae;g__Eggerthia	0.00 ± 0.00	0.0	0.00 ± 0.00	0.0	3.68E-02	-	-	3.70E-02
k__Bacteria;p__Firmicutes;c__Erysipelotrichia;o__Erysipelotrichales;f__Erysipelotrichaceae;g__Erysipelatoclostridium	0.00 ± 0.00	0.0	0.00 ± 0.00	0.0	NA	-	-	-
k__Bacteria;p__Firmicutes;c__Erysipelotrichia;o__Erysipelotrichales;f__Erysipelotrichaceae;g__Erysipelotrichaceae_UCG-004	0.00 ± 0.00	0.0	0.00 ± 0.00	0.0	NA	-	-	-
k__Bacteria;p__Firmicutes;c__Erysipelotrichia;o__Erysipelotrichales;f__Erysipelotrichaceae;g__Eubacterium	0.00 ± 0.00	0.0	0.00 ± 0.00	0.0	NA	-	-	-
k__Bacteria;p__Firmicutes;c__Erysipelotrichia;o__Erysipelotrichales;f__Erysipelotrichaceae;g__Faecalitalea	0.00 ± 0.00	0.0	0.00 ± 0.00	0.0	NA	-	-	-
k__Bacteria;p__Firmicutes;c__Erysipelotrichia;o__Erysipelotrichales;f__Erysipelotrichaceae;g__Holdemanella	0.00 ± 0.00	0.0	0.00 ± 0.00	0.0	2.45E-09	-	-	2.72E-09
k__Bacteria;p__Firmicutes;c__Erysipelotrichia;o__Erysipelotrichales;f__Erysipelotrichaceae;g__Holdemania	0.00 ± 0.00	0.0	0.01 ± 0.00	0.0	3.35E-01	-	-	-
k__Bacteria;p__Firmicutes;c__Erysipelotrichia;o__Erysipelotrichales;f__Erysipelotrichaceae;g__Longibaculum	0.00 ± 0.00	0.0	0.00 ± 0.00	0.0	2.53E-04	-	-	2.61E-04
k__Bacteria;p__Firmicutes;c__Erysipelotrichia;o__Erysipelotrichales;f__Erysipelotrichaceae;g__Longicatena	0.00 ± 0.00	0.0	0.00 ± 0.00	0.0	NA	-	-	-
k__Bacteria;p__Firmicutes;c__Erysipelotrichia;o__Erysipelotrichales;f__Erysipelotrichaceae;g__Massiliomicrobiota	0.00 ± 0.00	0.0	0.00 ± 0.00	0.0	NA	-	-	-
k__Bacteria;p__Firmicutes;c__Erysipelotrichia;o__Erysipelotrichales;f__Erysipelotrichaceae;g__Solobacterium	0.03 ± 0.01	0.0	0.01 ± 0.00	0.0	2.25E-07	2.0511	-	1.12E-07
k__Bacteria;p__Firmicutes;c__Erysipelotrichia;o__Erysipelotrichales;f__Erysipelotrichaceae;g__Turicibacter	0.00 ± 0.00	0.0	0.00 ± 0.00	0.0	2.32E-01	-	-	-
k__Bacteria;p__Firmicutes;c__Negativicutes;o__Acidaminococcales;f__Acidaminococcaceae;g__Acidaminococcus	0.00 ± 0.00	0.0	0.00 ± 0.00	0.0	NA	-	-	-
k__Bacteria;p__Firmicutes;c__Negativicutes;o__Acidaminococcales;f__Acidaminococcaceae;g__Phascolarctobacterium	0.04 ± 0.02	0.9	0.00 ± 0.00	0.0	1.00E-26	2.2757	-	1.41E-26

k__Bacteria;p__Firmicutes;c__Negativicutes;o__Acidaminococcales;f__Acidaminococcaceae;g__Succiniclasticum	0.00 ± 0.00	0.0	0.00 ± 0.00	0.0	4.10E-01	-	-	-
**k__Bacteria;p__Firmicutes;c__Negativicutes;o__Selenomonadales;f__Selenomonadaceae;g__**	**0.00 ± 0.00**	**0.0**	**1.88 ± 0.22**	**41.7**	**3.26E-40**	**-**	**3.9827**	**4.21E-40**
k__Bacteria;p__Firmicutes;c__Negativicutes;o__Selenomonadales;f__Selenomonadaceae;g__Megamonas	0.00 ± 0.00	0.0	0.00 ± 0.00	0.0	NA	-	-	-
k__Bacteria;p__Firmicutes;c__Negativicutes;o__Selenomonadales;f__Selenomonadaceae;g__Mitsuokella	0.00 ± 0.00	0.0	0.01 ± 0.00	0.0	4.03E-02	-	-	-
k__Bacteria;p__Firmicutes;c__Negativicutes;o__Selenomonadales;f__Selenomonadaceae;g__Pectinatus	0.00 ± 0.00	0.0	0.00 ± 0.00	0.0	4.10E-06	-	-	4.33E-06
k__Bacteria;p__Firmicutes;c__Negativicutes;o__Selenomonadales;f__Selenomonadaceae;g__Propionispira	0.02 ± 0.00	0.0	0.00 ± 0.00	0.0	3.32E-29	-	-	4.53E-29
k__Bacteria;p__Firmicutes;c__Negativicutes;o__Selenomonadales;f__Selenomonadaceae;g__Schwartzia	0.03 ± 0.01	0.9	0.10 ± 0.02	1.2	4.56E-04	-	2.5914	3.68E-04
k__Bacteria;p__Firmicutes;c__Negativicutes;o__Selenomonadales;f__Selenomonadaceae;g__Selenomonas	0.22 ± 0.09	3.4	0.70 ± 0.07	21.4	3.62E-16	-	3.3815	1.34E-16
k__Bacteria;p__Firmicutes;c__Negativicutes;o__Veillonellales;f__Veillonellaceae;g__Allisonella	0.00 ± 0.00	0.0	0.00 ± 0.00	0.0	8.92E-02	-	-	-
k__Bacteria;p__Firmicutes;c__Negativicutes;o__Veillonellales;f__Veillonellaceae;g__Anaeroglobus	0.02 ± 0.01	0.9	0.01 ± 0.00	0.0	1.04E-03	-	-	1.15E-03
k__Bacteria;p__Firmicutes;c__Negativicutes;o__Veillonellales;f__Veillonellaceae;g__Dialister	0.07 ± 0.01	0.9	0.29 ± 0.03	6.5	3.57E-10	-	3.0282	3.98E-10
k__Bacteria;p__Firmicutes;c__Negativicutes;o__Veillonellales;f__Veillonellaceae;g__Megasphaera	0.00 ± 0.00	0.0	0.90 ± 0.08	31.5	8.12E-36	-	3.6527	1.08E-35
**k__Bacteria;p__Firmicutes;c__Negativicutes;o__Veillonellales;f__Veillonellaceae;g__Veillonella**	**4.78 ± 0.43**	**78.4**	**12.58 ± 0.52**	**100.0**	**2.37E-24**	**-**	**4.5880**	**3.40E-24**
k__Bacteria;p__Firmicutes;c__Tissierellia;o__Tissierellales;f__Peptoniphilaceae;g__	0.00 ± 0.00	0.0	0.00 ± 0.00	0.0	1.43E-07	-	-	1.54E-07
k__Bacteria;p__Firmicutes;c__Tissierellia;o__Tissierellales;f__Peptoniphilaceae;g__Anaerococcus	0.01 ± 0.01	0.0	0.00 ± 0.00	0.0	3.40E-10	-	-	3.77E-10
k__Bacteria;p__Firmicutes;c__Tissierellia;o__Tissierellales;f__Peptoniphilaceae;g__Anaerosphaera	0.00 ± 0.00	0.0	0.00 ± 0.00	0.0	NA	-	-	-
k__Bacteria;p__Firmicutes;c__Tissierellia;o__Tissierellales;f__Peptoniphilaceae;g__Finegoldia	0.00 ± 0.00	0.0	0.00 ± 0.00	0.0	3.68E-02	-	-	3.70E-02
k__Bacteria;p__Firmicutes;c__Tissierellia;o__Tissierellales;f__Peptoniphilaceae;g__Gallicola	0.00 ± 0.00	0.0	0.00 ± 0.00	0.0	NA	-	-	-
k__Bacteria;p__Firmicutes;c__Tissierellia;o__Tissierellales;f__Peptoniphilaceae;g__Helcococcus	0.00 ± 0.00	0.0	0.00 ± 0.00	0.0	2.32E-01	-	-	-
k__Bacteria;p__Firmicutes;c__Tissierellia;o__Tissierellales;f__Peptoniphilaceae;g__Parvimonas	0.01 ± 0.00	0.0	0.16 ± 0.02	1.2	3.88E-18	-	2.8650	2.22E-18
k__Bacteria;p__Firmicutes;c__Tissierellia;o__Tissierellales;f__Peptoniphilaceae;g__Peptoniphilus	0.00 ± 0.00	0.0	0.00 ± 0.00	0.0	9.54E-07	-	-	1.03E-06
k__Bacteria;p__Firmicutes;c__Tissierellia;o__Tissierellales;f__Tissierellaceae;g__	0.00 ± 0.00	0.0	0.00 ± 0.00	0.0	2.32E-01	-	-	-
k__Bacteria;p__Firmicutes;c__Tissierellia;o__Tissierellales;f__Tissierellaceae;g__Tissierella	0.03 ± 0.01	0.0	0.00 ± 0.00	0.0	9.48E-31	2.1711	-	1.31E-30
k__Bacteria;p__Fusobacteria;c__Fusobacteriia;o__Fusobacteriales;f__Fusobacteriaceae;g__Cetobacterium	0.00 ± 0.00	0.0	0.00 ± 0.00	0.0	2.97E-03	-	-	3.02E-03
**k__Bacteria;p__Fusobacteria;c__Fusobacteriia;o__Fusobacteriales;f__Fusobacteriaceae;g__Fusobacterium**	**2.55 ± 0.27**	**60.3**	**3.39 ± 0.24**	**79.8**	**2.80E-04**	**-**	**3.6346**	**2.83E-04**
k__Bacteria;p__Fusobacteria;c__Fusobacteriia;o__Fusobacteriales;f__Leptotrichiaceae;g__Leptotrichia	1.86 ± 0.18	57.8	1.45 ± 0.09	58.3	4.63E-01	-	-	-
k__Bacteria;p__Fusobacteria;c__Fusobacteriia;o__Fusobacteriales;f__Leptotrichiaceae;g__Sneathia	0.00 ± 0.00	0.0	0.03 ± 0.02	1.2	8.04E-01	-	-	-
k__Bacteria;p__Fusobacteria;c__Fusobacteriia;o__Fusobacteriales;f__Leptotrichiaceae;g__Streptobacillus	0.63 ± 0.11	19.0	0.02 ± 0.00	0.0	7.95E-33	3.4880	-	1.21E-32
k__Bacteria;p__Gemmatimonadetes;c__;o__;f__;g__	0.00 ± 0.00	0.0	0.00 ± 0.00	0.0	NA	-	-	-
k__Bacteria;p__Gemmatimonadetes;c__Gemmatimonadetes;o__Gemmatimonadales;f__Gemmatimonadaceae;g__Gemmatirosa	0.00 ± 0.00	0.0	0.00 ± 0.00	0.0	NA	-	-	-
k__Bacteria;p__Gracilibacteria;c__;o__;f__;g__	0.04 ± 0.01	0.0	0.00 ± 0.00	0.0	3.99E-20	2.3076	-	4.95E-20
k__Bacteria;p__Lentisphaerae;c__Lentisphaeria;o__Victivallales;f__;g__	0.00 ± 0.00	0.0	0.00 ± 0.00	0.0	2.32E-01	-	-	-
k__Bacteria;p__Lentisphaerae;c__Lentisphaeria;o__Victivallales;f__Victivallaceae;g__	0.00 ± 0.00	0.0	0.00 ± 0.00	0.0	8.00E-01	-	-	-
k__Bacteria;p__Lentisphaerae;c__Lentisphaeria;o__Victivallales;f__Victivallaceae;g__Victivallis	0.00 ± 0.00	0.0	0.00 ± 0.00	0.0	7.87E-01	-	-	-
k__Bacteria;p__Lentisphaerae;c__Oligosphaeria;o__Oligosphaerales;f__Oligosphaeraceae;g__	0.00 ± 0.00	0.0	0.00 ± 0.00	0.0	3.68E-02	-	-	3.70E-02
k__Bacteria;p__Planctomycetes;c__Planctomycetia;o__Planctomycetales;f__Gemmataceae;g__	0.00 ± 0.00	0.0	0.00 ± 0.00	0.0	8.92E-02	-	-	-
k__Bacteria;p__Planctomycetes;c__Planctomycetia;o__Planctomycetales;f__Isosphaeraceae;g__Aquisphaera	0.00 ± 0.00	0.0	0.00 ± 0.00	0.0	NA	-	-	-
k__Bacteria;p__Proteobacteria;c__Alphaproteobacteria;o__Caulobacterales;f__Caulobacteraceae;g__	0.00 ± 0.00	0.0	0.00 ± 0.00	0.0	6.79E-03	-	-	6.89E-03
k__Bacteria;p__Proteobacteria;c__Alphaproteobacteria;o__Caulobacterales;f__Caulobacteraceae;g__Brevundimonas	0.00 ± 0.00	0.0	0.00 ± 0.00	0.0	6.79E-03	-	-	6.89E-03
k__Bacteria;p__Proteobacteria;c__Alphaproteobacteria;o__Caulobacterales;f__Caulobacteraceae;g__Caulobacter	0.00 ± 0.00	0.0	0.00 ± 0.00	0.0	4.10E-01	-	-	-
k__Bacteria;p__Proteobacteria;c__Alphaproteobacteria;o__Caulobacterales;f__Caulobacteraceae;g__Phenylobacterium	0.00 ± 0.00	0.0	0.01 ± 0.00	0.0	2.36E-01	-	-	-
k__Bacteria;p__Proteobacteria;c__Alphaproteobacteria;o__Rhizobiales;f__;g__	0.00 ± 0.00	0.0	0.00 ± 0.00	0.0	7.48E-11	-	-	8.43E-11
k__Bacteria;p__Proteobacteria;c__Alphaproteobacteria;o__Rhizobiales;f__Aurantimonadaceae;g__Aureimonas	0.00 ± 0.00	0.0	0.00 ± 0.00	0.0	2.32E-01	-	-	-
k__Bacteria;p__Proteobacteria;c__Alphaproteobacteria;o__Rhizobiales;f__Beijerinckiaceae;g__Camelimonas	0.00 ± 0.00	0.0	0.00 ± 0.00	0.0	NA	-	-	-
k__Bacteria;p__Proteobacteria;c__Alphaproteobacteria;o__Rhizobiales;f__Bradyrhizobiaceae;g__Bosea	0.00 ± 0.00	0.0	0.00 ± 0.00	0.0	NA	-	-	-
k__Bacteria;p__Proteobacteria;c__Alphaproteobacteria;o__Rhizobiales;f__Bradyrhizobiaceae;g__Bradyrhizobium	0.00 ± 0.00	0.0	0.00 ± 0.00	0.0	8.63E-01	-	-	-
k__Bacteria;p__Proteobacteria;c__Alphaproteobacteria;o__Rhizobiales;f__Brucellaceae;g__Pseudochrobactrum	0.00 ± 0.00	0.0	0.00 ± 0.00	0.0	2.32E-01	-	-	-
k__Bacteria;p__Proteobacteria;c__Alphaproteobacteria;o__Rhizobiales;f__Hyphomicrobiaceae;g__Devosia	0.00 ± 0.00	0.0	0.00 ± 0.00	0.0	2.32E-01	-	-	-
k__Bacteria;p__Proteobacteria;c__Alphaproteobacteria;o__Rhizobiales;f__Hyphomicrobiaceae;g__Hyphomicrobium	0.00 ± 0.00	0.0	0.00 ± 0.00	0.0	NA	-	-	-
k__Bacteria;p__Proteobacteria;c__Alphaproteobacteria;o__Rhizobiales;f__Hyphomicrobiaceae;g__Rhodomicrobium	0.00 ± 0.00	0.0	0.00 ± 0.00	0.0	2.32E-01	-	-	-
k__Bacteria;p__Proteobacteria;c__Alphaproteobacteria;o__Rhizobiales;f__Methylobacteriaceae;g__Methylobacterium	0.00 ± 0.00	0.0	0.01 ± 0.01	0.6	7.48E-11	-	-	8.43E-11
k__Bacteria;p__Proteobacteria;c__Alphaproteobacteria;o__Rhizobiales;f__Methylocystaceae;g__Methylosinus	0.00 ± 0.00	0.0	0.00 ± 0.00	0.0	1.57E-02	2.2291	-	1.58E-02
k__Bacteria;p__Proteobacteria;c__Alphaproteobacteria;o__Rhizobiales;f__Phyllobacteriaceae;g__Mesorhizobium	0.00 ± 0.00	0.0	0.00 ± 0.00	0.0	NA	-	-	-
k__Bacteria;p__Proteobacteria;c__Alphaproteobacteria;o__Rhizobiales;f__Rhizobiaceae;g__	0.00 ± 0.00	0.0	0.00 ± 0.00	0.0	1.57E-02	-	-	1.58E-02
k__Bacteria;p__Proteobacteria;c__Alphaproteobacteria;o__Rhizobiales;f__Rhizobiaceae;g__Rhizobium	0.00 ± 0.00	0.0	0.00 ± 0.00	0.0	8.92E-02	-	-	-
k__Bacteria;p__Proteobacteria;c__Alphaproteobacteria;o__Rhodobacterales;f__Hyphomonadaceae;g__	0.01 ± 0.00	0.0	0.00 ± 0.00	0.0	4.28E-16	-	-	5.13E-16
k__Bacteria;p__Proteobacteria;c__Alphaproteobacteria;o__Rhodobacterales;f__Rhodobacteraceae;g__	0.00 ± 0.00	0.0	0.00 ± 0.00	0.0	7.73E-07	-	-	8.24E-07
k__Bacteria;p__Proteobacteria;c__Alphaproteobacteria;o__Rhodobacterales;f__Rhodobacteraceae;g__Amaricoccus	0.00 ± 0.00	0.0	0.00 ± 0.00	0.0	NA	-	-	-
k__Bacteria;p__Proteobacteria;c__Alphaproteobacteria;o__Rhodobacterales;f__Rhodobacteraceae;g__Aquimixticola	0.00 ± 0.00	0.0	0.00 ± 0.00	0.0	3.68E-02	-	-	3.70E-02
k__Bacteria;p__Proteobacteria;c__Alphaproteobacteria;o__Rhodobacterales;f__Rhodobacteraceae;g__Gemmobacter	0.00 ± 0.00	0.0	0.00 ± 0.00	0.0	3.68E-02	-	-	3.70E-02
k__Bacteria;p__Proteobacteria;c__Alphaproteobacteria;o__Rhodobacterales;f__Rhodobacteraceae;g__Paracoccus	0.00 ± 0.00	0.0	0.00 ± 0.00	0.0	8.92E-02	-	-	-
k__Bacteria;p__Proteobacteria;c__Alphaproteobacteria;o__Rhodobacterales;f__Rhodobacteraceae;g__Rhodobacter	0.00 ± 0.00	0.0	0.00 ± 0.00	0.0	NA	-	-	-
k__Bacteria;p__Proteobacteria;c__Alphaproteobacteria;o__Rhodobacterales;f__Rhodobacteraceae;g__Rubellimicrobium	0.00 ± 0.00	0.0	0.00 ± 0.00	0.0	NA	-	-	-

**k__Bacteria;p__Proteobacteria;c__Alphaproteobacteria;o__Rhodospirillales;f__Acetobacteraceae;g__**	**1.39 ± 0.48**	**17.2**	**0.00 ± 0.00**	**0.0**	**4.30E-56**	**3.8601**	**-**	**7.35E-56**
k__Bacteria;p__Proteobacteria;c__Alphaproteobacteria;o__Rhodospirillales;f__Acetobacteraceae;g__Acidocella	0.00 ± 0.00	0.0	0.00 ± 0.00	0.0	2.32E-01	-	-	-
k__Bacteria;p__Proteobacteria;c__Alphaproteobacteria;o__Rhodospirillales;f__Acetobacteraceae;g__Belnapia	0.00 ± 0.00	0.0	0.00 ± 0.00	0.0	8.92E-02	-	-	-
k__Bacteria;p__Proteobacteria;c__Alphaproteobacteria;o__Rhodospirillales;f__Acetobacteraceae;g__Gluconobacter	0.00 ± 0.00	0.0	0.00 ± 0.00	0.0	8.92E-02	-	-	-
k__Bacteria;p__Proteobacteria;c__Alphaproteobacteria;o__Rhodospirillales;f__Acetobacteraceae;g__Paracraurococcus	0.00 ± 0.00	0.0	0.00 ± 0.00	0.0	8.92E-02	-	-	-
k__Bacteria;p__Proteobacteria;c__Alphaproteobacteria;o__Rhodospirillales;f__Acetobacteraceae;g__Rhodovastum	0.00 ± 0.00	0.0	0.00 ± 0.00	0.0	NA	-	-	-
k__Bacteria;p__Proteobacteria;c__Alphaproteobacteria;o__Rhodospirillales;f__Acetobacteraceae;g__Roseomonas	0.00 ± 0.00	0.0	0.00 ± 0.00	0.0	NA	-	-	-
k__Bacteria;p__Proteobacteria;c__Alphaproteobacteria;o__Rhodospirillales;f__Rhodospirillaceae;g__	0.01 ± 0.01	0.9	0.01 ± 0.00	0.0	2.84E-09	-	-	3.26E-09
k__Bacteria;p__Proteobacteria;c__Alphaproteobacteria;o__Rhodospirillales;f__Rhodospirillaceae;g__Ferrovibrio	0.00 ± 0.00	0.0	0.00 ± 0.00	0.0	NA	-	-	-
k__Bacteria;p__Proteobacteria;c__Alphaproteobacteria;o__Rhodospirillales;f__Rhodospirillaceae;g__Lacibacterium	0.00 ± 0.00	0.0	0.00 ± 0.00	0.0	NA	-	-	-
k__Bacteria;p__Proteobacteria;c__Alphaproteobacteria;o__Rhodospirillales;f__Rhodospirillaceae;g__Niveispirillum	0.00 ± 0.00	0.0	0.00 ± 0.00	0.0	2.32E-01	-	-	-
k__Bacteria;p__Proteobacteria;c__Alphaproteobacteria;o__Rhodospirillales;f__Rhodospirillaceae;g__Tistrella	0.00 ± 0.00	0.0	0.00 ± 0.00	0.0	8.92E-02	-	-	-
k__Bacteria;p__Proteobacteria;c__Alphaproteobacteria;o__Rickettsiales;f__;g__	0.00 ± 0.00	0.0	0.00 ± 0.00	0.0	1.11E-04	-	-	1.15E-04
k__Bacteria;p__Proteobacteria;c__Alphaproteobacteria;o__Sphingomonadales;f__Erythrobacteraceae;g__Altererythrobacter	0.00 ± 0.00	0.0	0.00 ± 0.00	0.0	2.32E-01	-	-	-
k__Bacteria;p__Proteobacteria;c__Alphaproteobacteria;o__Sphingomonadales;f__Sphingomonadaceae;g__	0.00 ± 0.00	0.0	0.00 ± 0.00	0.0	8.92E-02	-	-	-
k__Bacteria;p__Proteobacteria;c__Alphaproteobacteria;o__Sphingomonadales;f__Sphingomonadaceae;g__Novosphingobium	0.00 ± 0.00	0.0	0.00 ± 0.00	0.0	1.57E-02	-	-	1.58E-02
k__Bacteria;p__Proteobacteria;c__Alphaproteobacteria;o__Sphingomonadales;f__Sphingomonadaceae;g__Sphingobium	0.00 ± 0.00	0.0	0.00 ± 0.00	0.0	1.50E-01	-	-	-
k__Bacteria;p__Proteobacteria;c__Alphaproteobacteria;o__Sphingomonadales;f__Sphingomonadaceae;g__Sphingomonas	0.00 ± 0.00	0.0	0.01 ± 0.00	0.0	1.04E-03	-	-	4.80E-04
k__Bacteria;p__Proteobacteria;c__Alphaproteobacteria;o__Sphingomonadales;f__Sphingomonadaceae;g__Sphingopyxis	0.00 ± 0.00	0.0	0.00 ± 0.00	0.0	2.32E-01	-	-	-
k__Bacteria;p__Proteobacteria;c__Alphaproteobacteria;o__Sphingomonadales;f__Sphingomonadaceae;g__Sphingorhabdus	0.00 ± 0.00	0.0	0.00 ± 0.00	0.0	NA	-	-	-
k__Bacteria;p__Proteobacteria;c__Betaproteobacteria;o__;f__;g__	0.00 ± 0.00	0.0	0.01 ± 0.00	0.0	7.64E-03	-	-	7.40E-03
k__Bacteria;p__Proteobacteria;c__Betaproteobacteria;o__Burkholderiales;f__Alcaligenaceae;g__Achromobacter	0.00 ± 0.00	0.0	0.00 ± 0.00	0.0	8.92E-02	-	-	-
k__Bacteria;p__Proteobacteria;c__Betaproteobacteria;o__Burkholderiales;f__Alcaligenaceae;g__Alcaligenes	0.00 ± 0.00	0.0	0.00 ± 0.00	0.0	8.92E-02	-	-	-
k__Bacteria;p__Proteobacteria;c__Betaproteobacteria;o__Burkholderiales;f__Alcaligenaceae;g__Derxia	0.00 ± 0.00	0.0	0.00 ± 0.00	0.0	2.32E-01	-	-	-
k__Bacteria;p__Proteobacteria;c__Betaproteobacteria;o__Burkholderiales;f__Alcaligenaceae;g__Parapusillimonas	0.00 ± 0.00	0.0	0.00 ± 0.00	0.0	NA	-	-	-
k__Bacteria;p__Proteobacteria;c__Betaproteobacteria;o__Burkholderiales;f__Alcaligenaceae;g__Pelistega	0.00 ± 0.00	0.0	0.00 ± 0.00	0.0	2.32E-01	-	-	-
k__Bacteria;p__Proteobacteria;c__Betaproteobacteria;o__Burkholderiales;f__Burkholderiaceae;g__Burkholderia	0.00 ± 0.00	0.0	0.00 ± 0.00	0.0	1.21E-01	-	-	-
k__Bacteria;p__Proteobacteria;c__Betaproteobacteria;o__Burkholderiales;f__Burkholderiaceae;g__Cupriavidus	0.00 ± 0.00	0.0	0.00 ± 0.00	0.0	8.92E-02	-	-	-
**k__Bacteria;p__Proteobacteria;c__Betaproteobacteria;o__Burkholderiales;f__Burkholderiaceae;g__Lautropia**	**0.17 ± 0.04**	**3.4**	**1.66 ± 0.21**	**41.1**	**2.74E-20**	**-**	**3.8870**	**9.07E-21**
k__Bacteria;p__Proteobacteria;c__Betaproteobacteria;o__Burkholderiales;f__Burkholderiaceae;g__Limnobacter	0.00 ± 0.00	0.0	0.00 ± 0.00	0.0	NA	-	-	-
k__Bacteria;p__Proteobacteria;c__Betaproteobacteria;o__Burkholderiales;f__Burkholderiaceae;g__Paraburkholderia	0.00 ± 0.00	0.0	0.00 ± 0.00	0.0	NA	-	-	-
k__Bacteria;p__Proteobacteria;c__Betaproteobacteria;o__Burkholderiales;f__Burkholderiaceae;g__Ralstonia	0.00 ± 0.00	0.0	0.02 ± 0.01	0.6	2.47E-01	-	-	-
k__Bacteria;p__Proteobacteria;c__Betaproteobacteria;o__Burkholderiales;f__Comamonadaceae;g__	0.00 ± 0.00	0.0	0.00 ± 0.00	0.0	6.99E-03	-	-	7.14E-03
k__Bacteria;p__Proteobacteria;c__Betaproteobacteria;o__Burkholderiales;f__Comamonadaceae;g__Comamonas	0.00 ± 0.00	0.0	0.00 ± 0.00	0.0	1.96E-09	-	-	2.16E-09
k__Bacteria;p__Proteobacteria;c__Betaproteobacteria;o__Burkholderiales;f__Comamonadaceae;g__Delftia	0.00 ± 0.00	0.0	0.00 ± 0.00	0.0	1.45E-01	-	-	-
k__Bacteria;p__Proteobacteria;c__Betaproteobacteria;o__Burkholderiales;f__Comamonadaceae;g__Pelomonas	0.00 ± 0.00	0.0	0.02 ± 0.01	0.6	1.35E-01	-	-	-
k__Bacteria;p__Proteobacteria;c__Betaproteobacteria;o__Burkholderiales;f__Oxalobacteraceae;g__Duganella	0.00 ± 0.00	0.0	0.00 ± 0.00	0.0	2.32E-01	-	-	-
k__Bacteria;p__Proteobacteria;c__Betaproteobacteria;o__Burkholderiales;f__Oxalobacteraceae;g__Herbaspirillum	0.00 ± 0.00	0.0	0.00 ± 0.00	0.0	1.57E-02	-	-	1.58E-02
k__Bacteria;p__Proteobacteria;c__Betaproteobacteria;o__Burkholderiales;f__Oxalobacteraceae;g__Janthinobacterium	0.00 ± 0.00	0.0	0.00 ± 0.00	0.0	2.04E-01	-	-	-
k__Bacteria;p__Proteobacteria;c__Betaproteobacteria;o__Burkholderiales;f__Oxalobacteraceae;g__Massilia	0.00 ± 0.00	0.0	0.01 ± 0.01	0.0	1.54E-02	-	-	1.57E-02
k__Bacteria;p__Proteobacteria;c__Betaproteobacteria;o__Burkholderiales;f__Roseateles;g__	0.02 ± 0.02	0.9	0.00 ± 0.00	0.0	1.11E-04	2.0434	-	1.15E-04
k__Bacteria;p__Proteobacteria;c__Betaproteobacteria;o__Burkholderiales;f__Roseateles;g__aquatilis	0.00 ± 0.00	0.0	0.01 ± 0.00	0.0	5.12E-01	-	-	-
k__Bacteria;p__Proteobacteria;c__Betaproteobacteria;o__Burkholderiales;f__Rubrivivax;g__gelatinosus	0.00 ± 0.00	0.0	0.00 ± 0.00	0.0	1.57E-02	-	-	1.58E-02
k__Bacteria;p__Proteobacteria;c__Betaproteobacteria;o__Burkholderiales;f__Sutterellaceae;g__Parasutterella	0.00 ± 0.00	0.0	0.00 ± 0.00	0.0	1.15E-02	-	-	1.12E-02
k__Bacteria;p__Proteobacteria;c__Betaproteobacteria;o__Burkholderiales;f__Sutterellaceae;g__Sutterella	0.00 ± 0.00	0.0	0.01 ± 0.01	0.0	8.62E-02	-	-	-
k__Bacteria;p__Proteobacteria;c__Betaproteobacteria;o__Burkholderiales;f__Tepidimonas;g__fonticaldi	0.00 ± 0.00	0.0	0.00 ± 0.00	0.0	1.57E-02	-	-	1.58E-02
k__Bacteria;p__Proteobacteria;c__Betaproteobacteria;o__Hydrogenophilales;f__Hydrogenophilaceae;g__	0.00 ± 0.00	0.0	0.00 ± 0.00	0.0	5.74E-04	-	-	5.90E-04
k__Bacteria;p__Proteobacteria;c__Betaproteobacteria;o__Methylophilales;f__Methylophilaceae;g__	0.00 ± 0.00	0.0	0.00 ± 0.00	0.0	NA	-	-	-
**k__Bacteria;p__Proteobacteria;c__Betaproteobacteria;o__Neisseriales;f__Neisseriaceae;g__**	**0.74 ± 0.17**	**12.9**	**1.80 ± 0.18**	**48.8**	**7.87E-08**	**-**	**3.7138**	**4.22E-08**
k__Bacteria;p__Proteobacteria;c__Betaproteobacteria;o__Neisseriales;f__Neisseriaceae;g__Alysiella	0.02 ± 0.01	0.0	0.01 ± 0.00	0.0	2.25E-10	-	-	2.78E-10
k__Bacteria;p__Proteobacteria;c__Betaproteobacteria;o__Neisseriales;f__Neisseriaceae;g__Eikenella	0.01 ± 0.00	0.0	0.06 ± 0.01	0.0	2.17E-08	-	2.4017	1.66E-08
k__Bacteria;p__Proteobacteria;c__Betaproteobacteria;o__Neisseriales;f__Neisseriaceae;g__Kingella	0.00 ± 0.00	0.0	0.12 ± 0.02	1.2	1.08E-24	-	2.7620	1.51E-24
**k__Bacteria;p__Proteobacteria;c__Betaproteobacteria;o__Neisseriales;f__Neisseriaceae;g__Neisseria**	**0.18 ± 0.03**	**3.4**	**4.89 ± 0.46**	**73.8**	**1.13E-37**	**-**	**4.3722**	**1.35E-37**
k__Bacteria;p__Proteobacteria;c__Betaproteobacteria;o__Neisseriales;f__Neisseriaceae;g__Simonsiella	0.00 ± 0.00	0.0	0.01 ± 0.00	0.0	5.53E-02	-	-	-
k__Bacteria;p__Proteobacteria;c__Betaproteobacteria;o__Neisseriales;f__Neisseriaceae;g__Snodgrassella	0.00 ± 0.00	0.0	0.00 ± 0.00	0.0	4.10E-01	-	-	-
k__Bacteria;p__Proteobacteria;c__Betaproteobacteria;o__Rhodocyclales;f__Rhodocyclaceae;g__Azoarcus	0.00 ± 0.00	0.0	0.00 ± 0.00	0.0	NA	-	-	-
k__Bacteria;p__Proteobacteria;c__Betaproteobacteria;o__Rhodocyclales;f__Rhodocyclaceae;g__Propionivibrio	0.01 ± 0.00	0.0	0.00 ± 0.00	0.0	1.75E-21	-	-	2.21E-21
k__Bacteria;p__Proteobacteria;c__Deltaproteobacteria;o__Bradymonadales;f__;g__	0.00 ± 0.00	0.0	0.00 ± 0.00	0.0	4.10E-06	-	-	4.33E-06
k__Bacteria;p__Proteobacteria;c__Deltaproteobacteria;o__Desulfobacterales;f__Desulfobacteraceae;g__	0.00 ± 0.00	0.0	0.00 ± 0.00	0.0	1.78E-06	-	-	1.89E-06
k__Bacteria;p__Proteobacteria;c__Deltaproteobacteria;o__Desulfobacterales;f__Desulfobulbaceae;g__Desulfobulbus	0.00 ± 0.00	0.0	0.00 ± 0.00	0.0	1.11E-13	-	-	1.29E-13
k__Bacteria;p__Proteobacteria;c__Deltaproteobacteria;o__Desulfovibrionales;f__;g__	0.00 ± 0.00	0.0	0.00 ± 0.00	0.0	2.32E-01	-	-	-
k__Bacteria;p__Proteobacteria;c__Deltaproteobacteria;o__Desulfovibrionales;f__Desulfohalobiaceae;g__	0.00 ± 0.00	0.0	0.00 ± 0.00	0.0	NA	-	-	-
k__Bacteria;p__Proteobacteria;c__Deltaproteobacteria;o__Desulfovibrionales;f__Desulfomicrobiaceae;g__Desulfomicrobium	0.00 ± 0.00	0.0	0.00 ± 0.00	0.0	3.40E-10	-	-	3.77E-10

k__Bacteria;p__Proteobacteria;c__Deltaproteobacteria;o__Desulfovibrionales;f__Desulfovibrionaceae;g__	0.00 ± 0.00	0.0	0.00 ± 0.00	0.0	8.92E-02	-	-	-
k__Bacteria;p__Proteobacteria;c__Deltaproteobacteria;o__Desulfovibrionales;f__Desulfovibrionaceae;g__Desulfovibrio	0.01 ± 0.01	0.0	0.00 ± 0.00	0.0	9.40E-06	-	-	9.89E-06
k__Bacteria;p__Proteobacteria;c__Deltaproteobacteria;o__Desulfuromonadales;f__;g__	0.00 ± 0.00	0.0	0.00 ± 0.00	0.0	NA	-	-	-
k__Bacteria;p__Proteobacteria;c__Deltaproteobacteria;o__Desulfuromonadales;f__Desulfuromonadaceae;g__	0.00 ± 0.00	0.0	0.00 ± 0.00	0.0	NA	-	-	-
k__Bacteria;p__Proteobacteria;c__Deltaproteobacteria;o__Desulfuromonadales;f__Geobacteraceae;g__Geobacter	0.00 ± 0.00	0.0	0.00 ± 0.00	0.0	8.92E-02	-	-	-
k__Bacteria;p__Proteobacteria;c__Deltaproteobacteria;o__Myxococcales;f__Labilitrichaceae;g__Labilithrix	0.00 ± 0.00	0.0	0.00 ± 0.00	0.0	2.32E-01	-	-	-
k__Bacteria;p__Proteobacteria;c__Deltaproteobacteria;o__Myxococcales;f__Phaselicystidaceae;g__Phaselicystis	0.00 ± 0.00	0.0	0.00 ± 0.00	0.0	NA	-	-	-
k__Bacteria;p__Proteobacteria;c__Epsilonproteobacteria;o__Campylobacterales;f__Campylobacteraceae;g__	0.02 ± 0.00	0.0	0.00 ± 0.00	0.0	6.10E-22	-	-	7.73E-22
k__Bacteria;p__Proteobacteria;c__Epsilonproteobacteria;o__Campylobacterales;f__Campylobacteraceae;g__Arcobacter	0.00 ± 0.00	0.0	0.00 ± 0.00	0.0	2.32E-01	-	-	-
**k__Bacteria;p__Proteobacteria;c__Epsilonproteobacteria;o__Campylobacterales;f__Campylobacteraceae;g__Campylobacter**	**0.21 ± 0.03**	**5.2**	**2.14 ± 0.20**	**58.3**	**1.36E-13**	**-**	**3.9889**	**1.96E-13**
k__Bacteria;p__Proteobacteria;c__Epsilonproteobacteria;o__Campylobacterales;f__Campylobacteraceae;g__Sulfurospirillum	0.00 ± 0.00	0.0	0.00 ± 0.00	0.0	8.92E-02	-	-	-
k__Bacteria;p__Proteobacteria;c__Epsilonproteobacteria;o__Campylobacterales;f__Helicobacteraceae;g__	0.00 ± 0.00	0.0	0.00 ± 0.00	0.0	4.10E-01	-	-	-
k__Bacteria;p__Proteobacteria;c__Epsilonproteobacteria;o__Campylobacterales;f__Helicobacteraceae;g__Helicobacter	0.04 ± 0.01	0.0	0.00 ± 0.00	0.0	2.60E-41	2.3150	-	3.96E-41
k__Bacteria;p__Proteobacteria;c__Epsilonproteobacteria;o__Campylobacterales;f__Helicobacteraceae;g__Wolinella	0.01 ± 0.00	0.0	0.00 ± 0.00	0.0	3.09E-19	-	-	3.80E-19
k__Bacteria;p__Proteobacteria;c__Gammaproteobacteria;o__;f__;g__	0.00 ± 0.00	0.0	0.00 ± 0.00	0.0	1.11E-08	-	-	1.21E-08
k__Bacteria;p__Proteobacteria;c__Gammaproteobacteria;o__Aeromonadales;f__Aeromonadaceae;g__Aeromonas	0.00 ± 0.00	0.0	0.00 ± 0.00	0.0	NA	-	-	-
k__Bacteria;p__Proteobacteria;c__Gammaproteobacteria;o__Aeromonadales;f__Succinivibrionaceae;g__Succinivibrio	0.06 ± 0.05	0.9	0.00 ± 0.00	0.0	1.75E-21	2.5580	-	2.21E-21
k__Bacteria;p__Proteobacteria;c__Gammaproteobacteria;o__Alteromonadales;f__Alteromonadaceae;g__Marinobacter	0.00 ± 0.00	0.0	0.00 ± 0.00	0.0	5.74E-04	-	-	5.90E-04
k__Bacteria;p__Proteobacteria;c__Gammaproteobacteria;o__Alteromonadales;f__Idiomarinaceae;g__Aliidiomarina	0.00 ± 0.00	0.0	0.00 ± 0.00	0.0	2.97E-03	-	-	3.02E-03
k__Bacteria;p__Proteobacteria;c__Gammaproteobacteria;o__Cardiobacteriales;f__Cardiobacteriaceae;g__Cardiobacterium	0.02 ± 0.00	0.0	0.01 ± 0.00	0.0	3.32E-14	-	-	4.51E-14
k__Bacteria;p__Proteobacteria;c__Gammaproteobacteria;o__Cardiobacteriales;f__Cardiobacteriaceae;g__Suttonella	0.00 ± 0.00	0.0	0.00 ± 0.00	0.0	NA	-	-	-
k__Bacteria;p__Proteobacteria;c__Gammaproteobacteria;o__Chromatiales;f__Chromatiaceae;g__	0.00 ± 0.00	0.0	0.00 ± 0.00	0.0	1.50E-01	-	-	-
k__Bacteria;p__Proteobacteria;c__Gammaproteobacteria;o__Enterobacterales;f__Enterobacteriaceae;g__	0.00 ± 0.00	0.0	0.01 ± 0.00	0.0	1.55E-05	-	-	1.68E-05
k__Bacteria;p__Proteobacteria;c__Gammaproteobacteria;o__Enterobacterales;f__Enterobacteriaceae;g__Enterobacter	0.01 ± 0.00	0.0	0.01 ± 0.00	0.0	4.19E-03	-	-	4.46E-03
k__Bacteria;p__Proteobacteria;c__Gammaproteobacteria;o__Enterobacterales;f__Enterobacteriaceae;g__Kosakonia	0.00 ± 0.00	0.0	0.00 ± 0.00	0.0	NA	-	-	-
k__Bacteria;p__Proteobacteria;c__Gammaproteobacteria;o__Enterobacterales;f__Erwiniaceae;g__Buchnera	0.00 ± 0.00	0.0	0.00 ± 0.00	0.0	2.32E-01	-	-	-
k__Bacteria;p__Proteobacteria;c__Gammaproteobacteria;o__Enterobacterales;f__Erwiniaceae;g__Pantoea	0.00 ± 0.00	0.0	0.00 ± 0.00	0.0	NA	-	-	-
k__Bacteria;p__Proteobacteria;c__Gammaproteobacteria;o__Enterobacterales;f__Morganellaceae;g__	0.00 ± 0.00	0.0	0.00 ± 0.00	0.0	NA	-	-	-
k__Bacteria;p__Proteobacteria;c__Gammaproteobacteria;o__Enterobacterales;f__Morganellaceae;g__Providencia	0.00 ± 0.00	0.0	0.01 ± 0.00	0.0	6.18E-02	-	-	-
k__Bacteria;p__Proteobacteria;c__Gammaproteobacteria;o__Enterobacterales;f__Pectobacteriaceae;g__	0.00 ± 0.00	0.0	0.00 ± 0.00	0.0	2.32E-01	-	-	-
k__Bacteria;p__Proteobacteria;c__Gammaproteobacteria;o__Enterobacterales;f__Yersiniaceae;g__Ewingella	0.00 ± 0.00	0.0	0.00 ± 0.00	0.0	1.54E-02	-	-	1.57E-02
k__Bacteria;p__Proteobacteria;c__Gammaproteobacteria;o__Legionellales;f__Coxiellaceae;g__Diplorickettsia	0.00 ± 0.00	0.0	0.00 ± 0.00	0.0	8.92E-02	-	-	-
k__Bacteria;p__Proteobacteria;c__Gammaproteobacteria;o__Methylococcales;f__Methylococcaceae;g__	0.00 ± 0.00	0.0	0.00 ± 0.00	0.0	7.73E-07	-	-	8.24E-07
k__Bacteria;p__Proteobacteria;c__Gammaproteobacteria;o__Oceanospirillales;f__Halomonadaceae;g__Chromohalobacter	0.00 ± 0.00	0.0	0.00 ± 0.00	0.0	8.92E-02	-	-	-
k__Bacteria;p__Proteobacteria;c__Gammaproteobacteria;o__Oceanospirillales;f__Halomonadaceae;g__Halomonas	0.59 ± 0.08	11.2	0.00 ± 0.00	0.0	3.37E-58	3.4786	-	5.85E-58
k__Bacteria;p__Proteobacteria;c__Gammaproteobacteria;o__Oceanospirillales;f__Oceanospirillaceae;g__Marinobacterium	0.00 ± 0.00	0.0	0.00 ± 0.00	0.0	2.32E-01	-	-	-
k__Bacteria;p__Proteobacteria;c__Gammaproteobacteria;o__Orbales;f__Orbaceae;g__	0.00 ± 0.00	0.0	0.00 ± 0.00	0.0	NA	-	-	-
k__Bacteria;p__Proteobacteria;c__Gammaproteobacteria;o__Pasteurellales;f__Pasteurellaceae;g__	0.02 ± 0.00	0.0	0.00 ± 0.00	0.0	2.23E-49	2.0280	-	3.63E-49
**k__Bacteria;p__Proteobacteria;c__Gammaproteobacteria;o__Pasteurellales;f__Pasteurellaceae;g__Aggregatibacter**	**1.62 ± 0.12**	**67.2**	**0.64 ± 0.07**	**18.5**	**1.30E-20**	**3.6773**	**-**	**1.91E-20**
**k__Bacteria;p__Proteobacteria;c__Gammaproteobacteria;o__Pasteurellales;f__Pasteurellaceae;g__Haemophilus**	**26.42 ± 1.00**	**100.0**	**12.62 ± 0.60**	**99.4**	**2.04E-23**	**4.8359**	**-**	**2.98E-23**
k__Bacteria;p__Proteobacteria;c__Gammaproteobacteria;o__Pasteurellales;f__Pasteurellaceae;g__Mannheimia	0.01 ± 0.00	0.0	0.00 ± 0.00	0.0	1.08E-27	-	-	1.45E-27
k__Bacteria;p__Proteobacteria;c__Gammaproteobacteria;o__Pseudomonadales;f__Moraxellaceae;g__Acinetobacter	0.01 ± 0.00	0.0	0.00 ± 0.00	0.0	7.65E-11	-	-	8.68E-11
k__Bacteria;p__Proteobacteria;c__Gammaproteobacteria;o__Pseudomonadales;f__Moraxellaceae;g__Moraxella	0.03 ± 0.00	0.0	0.00 ± 0.00	0.0	3.97E-19	2.0991	-	4.99E-19
k__Bacteria;p__Proteobacteria;c__Gammaproteobacteria;o__Pseudomonadales;f__Moraxellaceae;g__Psychrobacter	0.00 ± 0.00	0.0	0.00 ± 0.00	0.0	2.15E-05	-	-	2.25E-05
k__Bacteria;p__Proteobacteria;c__Gammaproteobacteria;o__Pseudomonadales;f__Pseudomonadaceae;g__Pseudomonas	0.00 ± 0.00	0.0	0.01 ± 0.01	0.0	3.58E-04	-	-	3.83E-04
k__Bacteria;p__Proteobacteria;c__Gammaproteobacteria;o__Thiotrichales;f__Francisellaceae;g__Francisella	0.00 ± 0.00	0.0	0.00 ± 0.00	0.0	NA	-	-	-
k__Bacteria;p__Proteobacteria;c__Gammaproteobacteria;o__Xanthomonadales;f__;g__	0.00 ± 0.00	0.0	0.00 ± 0.00	0.0	NA	-	-	-
k__Bacteria;p__Proteobacteria;c__Gammaproteobacteria;o__Xanthomonadales;f__Rhodanobacteraceae;g__Dokdonella	0.00 ± 0.00	0.0	0.00 ± 0.00	0.0	NA	-	-	-
k__Bacteria;p__Proteobacteria;c__Gammaproteobacteria;o__Xanthomonadales;f__Rhodanobacteraceae;g__Rhodanobacter	0.00 ± 0.00	0.0	0.00 ± 0.00	0.0	2.32E-01	-	-	-
k__Bacteria;p__Proteobacteria;c__Gammaproteobacteria;o__Xanthomonadales;f__Xanthomonadaceae;g__	0.00 ± 0.00	0.0	0.00 ± 0.00	0.0	3.15E-07	-	-	3.48E-07
k__Bacteria;p__Proteobacteria;c__Gammaproteobacteria;o__Xanthomonadales;f__Xanthomonadaceae;g__Luteimonas	0.00 ± 0.00	0.0	0.00 ± 0.00	0.0	NA	-	-	-
k__Bacteria;p__Proteobacteria;c__Gammaproteobacteria;o__Xanthomonadales;f__Xanthomonadaceae;g__Lysobacter	0.00 ± 0.00	0.0	0.00 ± 0.00	0.0	NA	-	-	-
k__Bacteria;p__Proteobacteria;c__Gammaproteobacteria;o__Xanthomonadales;f__Xanthomonadaceae;g__Pseudoxanthomonas	0.00 ± 0.00	0.0	0.00 ± 0.00	0.0	4.10E-01	-	-	-
k__Bacteria;p__Proteobacteria;c__Gammaproteobacteria;o__Xanthomonadales;f__Xanthomonadaceae;g__Stenotrophomonas	0.00 ± 0.00	0.0	0.01 ± 0.00	0.0	2.38E-01	-	-	-
k__Bacteria;p__Proteobacteria;c__Gammaproteobacteria;o__Xanthomonadales;f__Xanthomonadaceae;g__Xanthomonas	0.00 ± 0.00	0.0	0.00 ± 0.00	0.0	NA	-	-	-
k__Bacteria;p__Saccharibacteria;c__;o__;f__;g__	0.00 ± 0.00	0.0	0.00 ± 0.00	0.0	5.06E-03	-	-	4.90E-03
k__Bacteria;p__Spirochaetes;c__Spirochaetia;o__Brachyspirales;f__Brachyspiraceae;g__Brachyspira	0.00 ± 0.00	0.0	0.00 ± 0.00	0.0	8.92E-02	-	-	-
k__Bacteria;p__Spirochaetes;c__Spirochaetia;o__Spirochaetales;f__Spirochaetaceae;g__	0.01 ± 0.00	0.0	0.03 ± 0.01	0.0	4.14E-01	-	-	-
k__Bacteria;p__Spirochaetes;c__Spirochaetia;o__Spirochaetales;f__Spirochaetaceae;g__Treponema	0.19 ± 0.04	2.6	0.30 ± 0.05	4.8	5.38E-02	-	2.8461	4.37E-02
k__Bacteria;p__Synergistetes;c__Synergistia;o__Synergistales;f__Synergistaceae;g__	0.00 ± 0.00	0.0	0.00 ± 0.00	0.0	8.92E-02	-	-	-
k__Bacteria;p__Synergistetes;c__Synergistia;o__Synergistales;f__Synergistaceae;g__Fretibacterium	0.02 ± 0.00	0.0	0.03 ± 0.01	0.0	1.33E-06	-	-	1.64E-06
k__Bacteria;p__Synergistetes;c__Synergistia;o__Synergistales;f__Synergistaceae;g__Jonquetella	0.00 ± 0.00	0.0	0.00 ± 0.00	0.0	NA	-	-	-
k__Bacteria;p__Synergistetes;c__Synergistia;o__Synergistales;f__Synergistaceae;g__Pyramidobacter	0.00 ± 0.00	0.0	0.00 ± 0.00	0.0	4.10E-06	-	-	4.33E-06

k__Bacteria;p__Tenericutes;c__Mollicutes;o__Acholeplasmatales;f__Acholeplasmataceae;g__	0.01 ± 0.00	0.0	0.02 ± 0.01	0.6	2.63E-13	-	-	3.23E-13
k__Bacteria;p__Tenericutes;c__Mollicutes;o__Anaeroplasmatales;f__Anaeroplasmataceae;g__	0.00 ± 0.00	0.0	0.00 ± 0.00	0.0	2.53E-04	-	-	2.61E-04
k__Bacteria;p__Tenericutes;c__Mollicutes;o__Anaeroplasmatales;f__Anaeroplasmataceae;g__Anaeroplasma	0.00 ± 0.00	0.0	0.00 ± 0.00	0.0	6.79E-03	-	-	6.89E-03
k__Bacteria;p__Tenericutes;c__Mollicutes;o__Anaeroplasmatales;f__Anaeroplasmataceae;g__Asteroleplasma	0.00 ± 0.00	0.0	0.00 ± 0.00	0.0	NA	-	-	-
k__Bacteria;p__Tenericutes;c__Mollicutes;o__Entomoplasmatales;f__Entomoplasmataceae;g__	0.00 ± 0.00	0.0	0.00 ± 0.00	0.0	NA	-	-	-
k__Bacteria;p__Tenericutes;c__Mollicutes;o__Entomoplasmatales;f__Entomoplasmataceae;g__Entomoplasma	0.00 ± 0.00	0.0	0.00 ± 0.00	0.0	2.32E-01	-	-	-
k__Bacteria;p__Tenericutes;c__Mollicutes;o__Entomoplasmatales;f__Spiroplasmataceae;g__	0.00 ± 0.00	0.0	0.12 ± 0.04	3.0	9.51E-02	-	-	-
k__Bacteria;p__Tenericutes;c__Mollicutes;o__Mollicutes_RF9;f__;g__	0.00 ± 0.00	0.0	0.00 ± 0.00	0.0	9.69E-07	-	-	1.05E-06
k__Bacteria;p__Tenericutes;c__Mollicutes;o__Mycoplasmatales;f__Mycoplasmataceae;g__	0.00 ± 0.00	0.0	0.01 ± 0.00	0.0	1.58E-01	-	-	-
k__Bacteria;p__Tenericutes;c__Mollicutes;o__Mycoplasmatales;f__Mycoplasmataceae;g__Mycoplasma	0.02 ± 0.00	0.0	0.03 ± 0.01	0.0	1.69E-06	-	-	2.10E-06
k__Bacteria;p__Tenericutes;c__Mollicutes;o__Mycoplasmatales;f__Mycoplasmataceae;g__Ureaplasma	0.00 ± 0.00	0.0	0.00 ± 0.00	0.0	NA	-	-	-
k__Bacteria;p__Tenericutes;c__Mollicutes;o__NB1-n;f__;g__	0.00 ± 0.00	0.0	0.00 ± 0.00	0.0	NA	-	-	-
k__Bacteria;p__Verrucomicrobia;c__;o__;f__;g__	0.00 ± 0.00	0.0	0.00 ± 0.00	0.0	7.73E-07	-	-	8.24E-07
k__Bacteria;p__Verrucomicrobia;c__Opitutae;o__Opitutae_vadinHA64;f__;g__	0.00 ± 0.00	0.0	0.00 ± 0.00	0.0	4.10E-01	-	-	-
k__Bacteria;p__Verrucomicrobia;c__Opitutae;o__Puniceicoccales;f__Puniceicoccaceae;g__	0.01 ± 0.00	0.0	0.00 ± 0.00	0.0	6.21E-13	-	-	7.12E-13
k__Bacteria;p__Verrucomicrobia;c__Opitutae;o__Puniceicoccales;f__Puniceicoccaceae;g__Cerasicoccus	0.00 ± 0.00	0.0	0.00 ± 0.00	0.0	NA	-	-	-
k__Bacteria;p__Verrucomicrobia;c__Spartobacteria;o__Chthoniobacterales;f__Chthoniobacteraceae;g__Chthoniobacter	0.00 ± 0.00	0.0	0.00 ± 0.00	0.0	2.32E-01	-	-	-
k__Bacteria;p__Verrucomicrobia;c__Verrucomicrobiae;o__Verrucomicrobiales;f__Akkermansiaceae;g__Akkermansia	0.00 ± 0.00	0.0	0.00 ± 0.00	0.0	4.10E-01	-	-	-

$^{\$}$ mean ± s.e.m

* Individual samples with >1% abundance were counted.

Table S5. Relative abundance of bacterial taxa (L6, at the genus and above levels) at the anal site of macaques and humans.

Anal site	Macaque		Human			LDA score		
	Abundance[$] (%)	Prevalence* (%)	Abundance[$] (%)	Prevalence* (%)	MW test (p)	Macaque	Human	p value
k__Bacteria;p__Acidobacteria;c__Acidobacteriia;o__Acidobacteriales;f__Acidobacteriaceae;g__	0.00 ± 0.00	0.0	0.00 ± 0.00	0.0	NA	-	-	-
k__Bacteria;p__Acidobacteria;c__Acidobacteriia;o__Acidobacteriales;f__Acidobacteriaceae;g__Terriglobus	0.00 ± 0.00	0.0	0.00 ± 0.00	0.0	NA	-	-	-
k__Bacteria;p__Acidobacteria;c__Holophagae;o__;f__;g__	0.00 ± 0.00	0.0	0.00 ± 0.00	0.0	NA	-	-	-
k__Bacteria;p__Acidobacteria;c__Solibacteres;o__Solibacterales;f__Bryobacteraceae;g__Paludibaculum	0.00 ± 0.00	0.0	0.00 ± 0.00	0.0	NA	-	-	-
k__Bacteria;p__Actinobacteria;c__Actinobacteria;o__Actinomycetales;f__Actinomycetaceae;g__	0.00 ± 0.00	0.0	0.00 ± 0.00	0.0	2.44E-01	-	-	-
k__Bacteria;p__Actinobacteria;c__Actinobacteria;o__Actinomycetales;f__Actinomycetaceae;g__Actinobaculum	0.00 ± 0.00	0.0	0.00 ± 0.00	0.0	NA	-	-	-
k__Bacteria;p__Actinobacteria;c__Actinobacteria;o__Actinomycetales;f__Actinomycetaceae;g__Actinomyces	0.00 ± 0.00	0.0	0.00 ± 0.00	0.0	9.28E-01	-	-	-
k__Bacteria;p__Actinobacteria;c__Actinobacteria;o__Actinomycetales;f__Actinomycetaceae;g__Actinotignum	0.00 ± 0.00	0.0	0.00 ± 0.00	0.0	NA	-	-	-
k__Bacteria;p__Actinobacteria;c__Actinobacteria;o__Actinomycetales;f__Actinomycetaceae;g__Arcanobacterium	0.00 ± 0.00	0.0	0.00 ± 0.00	0.0	2.44E-01	-	-	-
k__Bacteria;p__Actinobacteria;c__Actinobacteria;o__Actinomycetales;f__Actinomycetaceae;g__Flaviflexus	0.00 ± 0.00	0.0	0.00 ± 0.00	0.0	NA	-	-	-
k__Bacteria;p__Actinobacteria;c__Actinobacteria;o__Actinomycetales;f__Actinomycetaceae;g__Mobiluncus	0.00 ± 0.00	0.0	0.00 ± 0.00	0.0	1.66E-04	-	-	1.72E-04
k__Bacteria;p__Actinobacteria;c__Actinobacteria;o__Actinomycetales;f__Actinomycetaceae;g__Trueperella	0.00 ± 0.00	0.0	0.00 ± 0.00	0.0	2.44E-01	-	-	-
k__Bacteria;p__Actinobacteria;c__Actinobacteria;o__Actinomycetales;f__Actinomycetaceae;g__Varibaculum	0.00 ± 0.00	0.0	0.00 ± 0.00	0.0	7.57E-05	-	-	7.89E-05
k__Bacteria;p__Actinobacteria;c__Actinobacteria;o__Bifidobacteriales;f__Bifidobacteriaceae;g__Alloscardovia	0.00 ± 0.00	0.0	0.00 ± 0.00	0.0	1.57E-05	-	-	1.65E-05
k__Bacteria;p__Actinobacteria;c__Actinobacteria;o__Bifidobacteriales;f__Bifidobacteriaceae;g__Bifidobacterium	0.00 ± 0.00	0.0	0.06 ± 0.02	1.3	1.08E-03	-	2.4839	9.81E-04
k__Bacteria;p__Actinobacteria;c__Actinobacteria;o__Bifidobacteriales;f__Bifidobacteriaceae;g__Gardnerella	0.00 ± 0.00	0.0	0.00 ± 0.00	0.0	4.20E-02	-	-	4.22E-02
k__Bacteria;p__Actinobacteria;c__Actinobacteria;o__Bifidobacteriales;f__Bifidobacteriaceae;g__Scardovia	0.00 ± 0.00	0.0	0.00 ± 0.00	0.0	NA	-	-	-
k__Bacteria;p__Actinobacteria;c__Actinobacteria;o__Corynebacteriales;f__Corynebacteriaceae;g__Corynebacterium	0.01 ± 0.00	0.0	0.00 ± 0.00	0.0	1.71E-11	-	-	1.97E-11
k__Bacteria;p__Actinobacteria;c__Actinobacteria;o__Corynebacteriales;f__Dietziaceae;g__Dietzia	0.00 ± 0.00	0.0	0.00 ± 0.00	0.0	4.20E-02	-	-	4.22E-02
k__Bacteria;p__Actinobacteria;c__Actinobacteria;o__Corynebacteriales;f__Mycobacteriaceae;g__Mycobacterium	0.00 ± 0.00	0.0	0.00 ± 0.00	0.0	NA	-	-	-
k__Bacteria;p__Actinobacteria;c__Actinobacteria;o__Corynebacteriales;f__Nocardiaceae;g__	0.00 ± 0.00	0.0	0.00 ± 0.00	0.0	1.73E-03	-	-	1.77E-03
k__Bacteria;p__Actinobacteria;c__Actinobacteria;o__Frankiales;f__;g__	0.00 ± 0.00	0.0	0.00 ± 0.00	0.0	NA	-	-	-
k__Bacteria;p__Actinobacteria;c__Actinobacteria;o__Frankiales;f__Antricoccus;g__	0.00 ± 0.00	0.0	0.00 ± 0.00	0.0	NA	-	-	-
k__Bacteria;p__Actinobacteria;c__Actinobacteria;o__Frankiales;f__Frankiaceae;g__Jatrophihabitans	0.00 ± 0.00	0.0	0.00 ± 0.00	0.0	NA	-	-	-
k__Bacteria;p__Actinobacteria;c__Actinobacteria;o__Kineosporiales;f__Kineosporiaceae;g__Kineococcus	0.00 ± 0.00	0.0	0.00 ± 0.00	0.0	NA	-	-	-
k__Bacteria;p__Actinobacteria;c__Actinobacteria;o__Micrococcales;f__Bogoriellaceae;g__Bogoriella	0.00 ± 0.00	0.0	0.00 ± 0.00	0.0	9.78E-02	-	-	-
k__Bacteria;p__Actinobacteria;c__Actinobacteria;o__Micrococcales;f__Brevibacteriaceae;g__Brevibacterium	0.00 ± 0.00	0.0	0.00 ± 0.00	0.0	2.44E-01	-	-	-
k__Bacteria;p__Actinobacteria;c__Actinobacteria;o__Micrococcales;f__Cellulomonadaceae;g__Cellulomonas	0.00 ± 0.00	0.0	0.00 ± 0.00	0.0	NA	-	-	-
k__Bacteria;p__Actinobacteria;c__Actinobacteria;o__Micrococcales;f__Dermabacteraceae;g__Brachybacterium	0.00 ± 0.00	0.0	0.00 ± 0.00	0.0	8.36E-03	-	-	8.49E-03
k__Bacteria;p__Actinobacteria;c__Actinobacteria;o__Micrococcales;f__Dermatophilaceae;g__Dermatophilus	0.00 ± 0.00	0.0	0.00 ± 0.00	0.0	NA	-	-	-
k__Bacteria;p__Actinobacteria;c__Actinobacteria;o__Micrococcales;f__Dermatophilaceae;g__Kineosphaera	0.00 ± 0.00	0.0	0.00 ± 0.00	0.0	NA	-	-	-
k__Bacteria;p__Actinobacteria;c__Actinobacteria;o__Micrococcales;f__Dermatophilaceae;g__Piscicoccus	0.00 ± 0.00	0.0	0.00 ± 0.00	0.0	NA	-	-	-
k__Bacteria;p__Actinobacteria;c__Actinobacteria;o__Micrococcales;f__Intrasporangiaceae;g__Knoellia	0.00 ± 0.00	0.0	0.00 ± 0.00	0.0	NA	-	-	-
k__Bacteria;p__Actinobacteria;c__Actinobacteria;o__Micrococcales;f__Intrasporangiaceae;g__Phycicoccus	0.00 ± 0.00	0.0	0.00 ± 0.00	0.0	NA	-	-	-
k__Bacteria;p__Actinobacteria;c__Actinobacteria;o__Micrococcales;f__Microbacteriaceae;g__	0.00 ± 0.00	0.0	0.00 ± 0.00	0.0	9.78E-02	-	-	-
k__Bacteria;p__Actinobacteria;c__Actinobacteria;o__Micrococcales;f__Microbacteriaceae;g__Amnibacterium	0.00 ± 0.00	0.0	0.00 ± 0.00	0.0	9.78E-02	-	-	-
k__Bacteria;p__Actinobacteria;c__Actinobacteria;o__Micrococcales;f__Microbacteriaceae;g__Leucobacter	0.00 ± 0.00	0.0	0.00 ± 0.00	0.0	NA	-	-	-
k__Bacteria;p__Actinobacteria;c__Actinobacteria;o__Micrococcales;f__Micrococcaceae;g__Arthrobacter	0.00 ± 0.00	0.0	0.00 ± 0.00	0.0	4.20E-02	-	-	4.22E-02
k__Bacteria;p__Actinobacteria;c__Actinobacteria;o__Micrococcales;f__Micrococcaceae;g__Glutamicibacter	0.00 ± 0.00	0.0	0.00 ± 0.00	0.0	2.44E-01	-	-	-
k__Bacteria;p__Actinobacteria;c__Actinobacteria;o__Micrococcales;f__Micrococcaceae;g__Kocuria	0.00 ± 0.00	0.0	0.00 ± 0.00	0.0	8.36E-03	-	-	8.49E-03
k__Bacteria;p__Actinobacteria;c__Actinobacteria;o__Micrococcales;f__Micrococcaceae;g__Micrococcus	0.00 ± 0.00	0.0	0.00 ± 0.00	0.0	1.86E-02	-	-	1.88E-02
k__Bacteria;p__Actinobacteria;c__Actinobacteria;o__Micrococcales;f__Micrococcaceae;g__Paenarthrobacter	0.00 ± 0.00	0.0	0.00 ± 0.00	0.0	NA	-	-	-
k__Bacteria;p__Actinobacteria;c__Actinobacteria;o__Micrococcales;f__Micrococcaceae;g__Rothia	0.00 ± 0.00	0.0	0.00 ± 0.00	0.0	7.57E-05	-	-	7.89E-05
k__Bacteria;p__Actinobacteria;c__Actinobacteria;o__Micromonosporales;f__Micromonosporaceae;g__Catellatospora	0.00 ± 0.00	0.0	0.00 ± 0.00	0.0	NA	-	-	-
k__Bacteria;p__Actinobacteria;c__Actinobacteria;o__Nakamurellales;f__Nakamurellaceae;g__Nakamurella	0.00 ± 0.00	0.0	0.00 ± 0.00	0.0	NA	-	-	-
k__Bacteria;p__Actinobacteria;c__Actinobacteria;o__Propionibacteriales;f__Nocardioidaceae;g__Marmoricola	0.00 ± 0.00	0.0	0.00 ± 0.00	0.0	NA	-	-	-
k__Bacteria;p__Actinobacteria;c__Actinobacteria;o__Propionibacteriales;f__Propionibacteriaceae;g__Cutibacterium	0.00 ± 0.00	0.0	0.00 ± 0.00	0.0	2.29E-01	-	-	-
k__Bacteria;p__Actinobacteria;c__Actinobacteria;o__Propionibacteriales;f__Propionibacteriaceae;g__Propionimicrobium	0.00 ± 0.00	0.0	0.00 ± 0.00	0.0	NA	-	-	-
k__Bacteria;p__Actinobacteria;c__Actinobacteria;o__Propionibacteriales;f__Propionibacteriaceae;g__Pseudopropionibacterium	0.00 ± 0.00	0.0	0.00 ± 0.00	0.0	NA	-	-	-
k__Bacteria;p__Actinobacteria;c__Actinobacteria;o__Pseudonocardiales;f__Pseudonocardiaceae;g__Actinomycetospora	0.00 ± 0.00	0.0	0.00 ± 0.00	0.0	NA	-	-	-
k__Bacteria;p__Actinobacteria;c__Actinobacteria;o__Pseudonocardiales;f__Pseudonocardiaceae;g__Saccharopolyspora	0.00 ± 0.00	0.0	0.00 ± 0.00	0.0	NA	-	-	-
k__Bacteria;p__Actinobacteria;c__Actinobacteria;o__Streptosporangiales;f__Thermomonosporaceae;g__Actinoallomurus	0.00 ± 0.00	0.0	0.00 ± 0.00	0.0	NA	-	-	-
k__Bacteria;p__Actinobacteria;c__Coriobacteriia;o__Coriobacteriales;f__Atopobiaceae;g__	0.00 ± 0.00	0.0	0.00 ± 0.00	0.0	2.44E-01	-	-	-
k__Bacteria;p__Actinobacteria;c__Coriobacteriia;o__Coriobacteriales;f__Atopobiaceae;g__Atopobium	0.00 ± 0.00	0.0	0.00 ± 0.00	0.0	4.23E-05	-	-	4.49E-05
k__Bacteria;p__Actinobacteria;c__Coriobacteriia;o__Coriobacteriales;f__Atopobiaceae;g__Olsenella	0.08 ± 0.01	0.0	0.00 ± 0.00	0.0	8.67E-46	2.5800	-	1.40E-45
k__Bacteria;p__Actinobacteria;c__Coriobacteriia;o__Coriobacteriales;f__Coriobacteriaceae;g__	0.10 ± 0.01	0.0	0.00 ± 0.00	0.0	3.08E-50	2.6954	-	5.15E-50
k__Bacteria;p__Actinobacteria;c__Coriobacteriia;o__Coriobacteriales;f__Coriobacteriaceae;g__Collinsella	0.04 ± 0.01	0.0	0.06 ± 0.01	0.6	3.06E-06	-	2.2240	3.99E-06
k__Bacteria;p__Actinobacteria;c__Coriobacteriia;o__Coriobacteriales;f__Coriobacteriaceae;g__Parvibacter	0.00 ± 0.00	0.0	0.00 ± 0.00	0.0	9.78E-02	-	-	-

k__Bacteria;p__Actinobacteria;c__Coriobacteriia;o__Coriobacteriales;f__Coriobacteriaceae;g__Senegalimassilia	0.03 ± 0.00	0.0	0.00 ± 0.00	0.0	9.48E-39	2.1407	-	1.46E-38
k__Bacteria;p__Actinobacteria;c__Coriobacteriia;o__Eggerthellales;f__Eggerthellaceae;g__	0.07 ± 0.02	0.9	0.00 ± 0.00	0.0	3.07E-22	2.5799	-	1.07E-22
k__Bacteria;p__Actinobacteria;c__Coriobacteriia;o__Eggerthellales;f__Eggerthellaceae;g__Eggerthella	0.01 ± 0.00	0.0	0.00 ± 0.00	0.0	9.18E-24	-	-	1.20E-23
k__Bacteria;p__Actinobacteria;c__Coriobacteriia;o__Eggerthellales;f__Eggerthellaceae;g__Enterorhabdus	0.01 ± 0.00	0.0	0.00 ± 0.00	0.0	1.32E-20	-	-	1.67E-20
k__Bacteria;p__Actinobacteria;c__Coriobacteriia;o__Eggerthellales;f__Eggerthellaceae;g__Gordonibacter	0.00 ± 0.00	0.0	0.00 ± 0.00	0.0	NA	-	-	-
k__Bacteria;p__Actinobacteria;c__Coriobacteriia;o__Eggerthellales;f__Eggerthellaceae;g__Raoultibacter	0.00 ± 0.00	0.0	0.01 ± 0.00	0.0	6.19E-01	-	-	-
k__Bacteria;p__Actinobacteria;c__Coriobacteriia;o__Eggerthellales;f__Eggerthellaceae;g__Slackia	0.01 ± 0.00	0.0	0.00 ± 0.00	0.0	3.07E-24	-	-	4.19E-24
k__Bacteria;p__Actinobacteria;c__Thermoleophilia;o__Solirubrobacterales;f__Solirubrobacteraceae;g__Solirubrobacter	0.00 ± 0.00	0.0	0.00 ± 0.00	0.0	NA	-	-	-
k__Bacteria;p__Bacteroidetes;c__;o__;f__;g__	0.00 ± 0.00	0.0	0.00 ± 0.00	0.0	9.78E-02	-	-	-
k__Bacteria;p__Bacteroidetes;c__Bacteroidia;o__Bacteroidales;f__;g__	0.08 ± 0.02	1.7	0.00 ± 0.00	0.0	7.60E-23	2.6490	-	9.86E-23
k__Bacteria;p__Bacteroidetes;c__Bacteroidia;o__Bacteroidales;f__Bacteroidaceae;g__	0.59 ± 0.10	16.5	0.00 ± 0.00	0.0	1.13E-53	3.4878	-	1.94E-53
k__Bacteria;p__Bacteroidetes;c__Bacteroidia;o__Bacteroidales;f__Bacteroidaceae;g__Anaerorhabdus	0.00 ± 0.00	0.0	0.00 ± 0.00	0.0	NA	-	-	-
**k__Bacteria;p__Bacteroidetes;c__Bacteroidia;o__Bacteroidales;f__Bacteroidaceae;g__Bacteroides**	**0.29 ± 0.12**	**3.5**	**51.73 ± 1.90**	**100.0**	**5.04E-45**	**-**	**5.4040**	**6.53E-45**
k__Bacteria;p__Bacteroidetes;c__Bacteroidia;o__Bacteroidales;f__Bacteroidales_RF16_group;g__	0.00 ± 0.00	0.0	0.00 ± 0.00	0.0	NA	-	-	-
k__Bacteria;p__Bacteroidetes;c__Bacteroidia;o__Bacteroidales;f__Odoribacteraceae;g__Butyricimonas	0.00 ± 0.00	0.0	0.36 ± 0.06	12.0	3.16E-11	-	3.2807	2.45E-11
k__Bacteria;p__Bacteroidetes;c__Bacteroidia;o__Bacteroidales;f__Odoribacteraceae;g__Odoribacter	0.00 ± 0.00	0.0	0.54 ± 0.07	14.6	9.94E-27	-	3.4190	6.22E-27
k__Bacteria;p__Bacteroidetes;c__Bacteroidia;o__Bacteroidales;f__Phocaeicola;g__abscessus	0.00 ± 0.00	0.0	0.00 ± 0.00	0.0	NA	-	-	-
k__Bacteria;p__Bacteroidetes;c__Bacteroidia;o__Bacteroidales;f__Porphyromonadaceae;g__	0.63 ± 0.09	15.7	0.69 ± 0.15	13.3	1.51E-18	-	3.0752	2.41E-18
k__Bacteria;p__Bacteroidetes;c__Bacteroidia;o__Bacteroidales;f__Porphyromonadaceae;g__Barnesiella	0.00 ± 0.00	0.0	0.96 ± 0.18	25.9	1.31E-15	-	3.6780	1.03E-15
k__Bacteria;p__Bacteroidetes;c__Bacteroidia;o__Bacteroidales;f__Porphyromonadaceae;g__Coprobacter	0.00 ± 0.00	0.0	0.09 ± 0.02	2.5	1.74E-08	-	2.6448	2.54E-08
k__Bacteria;p__Bacteroidetes;c__Bacteroidia;o__Bacteroidales;f__Porphyromonadaceae;g__Muribaculum	0.12 ± 0.04	0.9	0.33 ± 0.12	5.1	2.56E-17	-	3.0483	3.52E-17
k__Bacteria;p__Bacteroidetes;c__Bacteroidia;o__Bacteroidales;f__Porphyromonadaceae;g__Paludibacter	0.31 ± 0.06	7.0	0.00 ± 0.00	0.0	2.65E-48	3.1849	-	4.36E-48
**k__Bacteria;p__Bacteroidetes;c__Bacteroidia;o__Bacteroidales;f__Porphyromonadaceae;g__Parabacteroides**	**0.04 ± 0.02**	**0.9**	**3.73 ± 0.38**	**73.4**	**1.73E-31**	**-**	**4.2579**	**2.47E-31**
k__Bacteria;p__Bacteroidetes;c__Bacteroidia;o__Bacteroidales;f__Porphyromonadaceae;g__Petrimonas	0.00 ± 0.00	0.0	0.00 ± 0.00	0.0	2.44E-01	-	-	-
k__Bacteria;p__Bacteroidetes;c__Bacteroidia;o__Bacteroidales;f__Porphyromonadaceae;g__Porphyromonas	0.18 ± 0.09	2.6	0.00 ± 0.00	0.0	1.52E-22	2.9528	-	2.00E-22
k__Bacteria;p__Bacteroidetes;c__Bacteroidia;o__Bacteroidales;f__Porphyromonadaceae;g__Proteiniphilum	0.00 ± 0.00	0.0	0.00 ± 0.00	0.0	NA	-	-	-
k__Bacteria;p__Bacteroidetes;c__Bacteroidia;o__Bacteroidales;f__Porphyromonadaceae;g__Tannerella	0.00 ± 0.00	0.0	0.00 ± 0.00	0.0	NA	-	-	-
k__Bacteria;p__Bacteroidetes;c__Bacteroidia;o__Bacteroidales;f__Prevotellaceae;g__	0.45 ± 0.06	13.9	0.20 ± 0.07	5.7	2.81E-34	3.1272	-	4.72E-34
k__Bacteria;p__Bacteroidetes;c__Bacteroidia;o__Bacteroidales;f__Prevotellaceae;g__Alloprevotella	0.21 ± 0.04	5.2	0.15 ± 0.09	2.5	8.47E-44	2.7558	-	1.41E-43
k__Bacteria;p__Bacteroidetes;c__Bacteroidia;o__Bacteroidales;f__Prevotellaceae;g__Paraprevotella	0.00 ± 0.00	0.0	0.88 ± 0.20	15.8	7.55E-08	-	3.6842	6.77E-08
**k__Bacteria;p__Bacteroidetes;c__Bacteroidia;o__Bacteroidales;f__Prevotellaceae;g__Prevotella**	**18.67 ± 1.15**	**96.5**	**3.17 ± 0.75**	**17.7**	**4.92E-34**	**4.8825**	**-**	**7.82E-34**
**k__Bacteria;p__Bacteroidetes;c__Bacteroidia;o__Bacteroidales;f__Prevotellaceae;g__Prevotellamassilia**	**4.92 ± 0.43**	**74.8**	**0.14 ± 0.09**	**1.9**	**1.83E-49**	**4.3695**	**-**	**3.11E-49**
k__Bacteria;p__Bacteroidetes;c__Bacteroidia;o__Bacteroidales;f__Rikenellaceae;g__	0.06 ± 0.01	0.9	0.34 ± 0.23	1.9	1.49E-28	-	3.2527	2.11E-28
**k__Bacteria;p__Bacteroidetes;c__Bacteroidia;o__Bacteroidales;f__Rikenellaceae;g__Alistipes**	**0.01 ± 0.01**	**0.0**	**5.92 ± 0.49**	**81.6**	**1.94E-35**	**-**	**4.4765**	**2.65E-35**
k__Bacteria;p__Bacteroidetes;c__Bacteroidia;o__Bacteroidales;f__Rikenellaceae;g__Rikenellaceae_RC9_gut_group	0.11 ± 0.02	1.7	0.11 ± 0.08	1.3	2.85E-38	2.6163	-	4.40E-38
k__Bacteria;p__Bacteroidetes;c__Bacteroidia;o__Marinilabiliales;f__Marinilabiliaceae;g__	0.28 ± 0.12	3.5	0.00 ± 0.00	0.0	1.17E-38	3.1074	-	1.77E-38
k__Bacteria;p__Bacteroidetes;c__Bacteroidia;o__Marinilabiliales;f__Prolixibacteraceae;g__Mariniphaga	0.00 ± 0.00	0.0	0.00 ± 0.00	0.0	NA	-	-	-
k__Bacteria;p__Bacteroidetes;c__Chitinophagia;o__Chitinophagales;f__Chitinophagaceae;g__	0.00 ± 0.00	0.0	0.00 ± 0.00	0.0	NA	-	-	-
k__Bacteria;p__Bacteroidetes;c__Chitinophagia;o__Chitinophagales;f__Chitinophagaceae;g__Chitinophaga	0.00 ± 0.00	0.0	0.00 ± 0.00	0.0	NA	-	-	-
k__Bacteria;p__Bacteroidetes;c__Chitinophagia;o__Chitinophagales;f__Chitinophagaceae;g__Filimonas	0.00 ± 0.00	0.0	0.00 ± 0.00	0.0	NA	-	-	-
k__Bacteria;p__Bacteroidetes;c__Chitinophagia;o__Chitinophagales;f__Chitinophagaceae;g__Flaviaesturariibacter	0.00 ± 0.00	0.0	0.00 ± 0.00	0.0	NA	-	-	-
k__Bacteria;p__Bacteroidetes;c__Chitinophagia;o__Chitinophagales;f__Chitinophagaceae;g__Flavihumibacter	0.00 ± 0.00	0.0	0.00 ± 0.00	0.0	2.44E-01	-	-	-
k__Bacteria;p__Bacteroidetes;c__Chitinophagia;o__Chitinophagales;f__Chitinophagaceae;g__Flavisolibacter	0.00 ± 0.00	0.0	0.00 ± 0.00	0.0	NA	-	-	-
k__Bacteria;p__Bacteroidetes;c__Chitinophagia;o__Chitinophagales;f__Chitinophagaceae;g__Heliimonas	0.00 ± 0.00	0.0	0.00 ± 0.00	0.0	NA	-	-	-
k__Bacteria;p__Bacteroidetes;c__Chitinophagia;o__Chitinophagales;f__Chitinophagaceae;g__Niabella	0.00 ± 0.00	0.0	0.00 ± 0.00	0.0	NA	-	-	-
k__Bacteria;p__Bacteroidetes;c__Chitinophagia;o__Chitinophagales;f__Chitinophagaceae;g__Sediminibacterium	0.00 ± 0.00	0.0	0.00 ± 0.00	0.0	NA	-	-	-
k__Bacteria;p__Bacteroidetes;c__Chitinophagia;o__Chitinophagales;f__Chitinophagaceae;g__Taibaiella	0.00 ± 0.00	0.0	0.00 ± 0.00	0.0	NA	-	-	-
k__Bacteria;p__Bacteroidetes;c__Chitinophagia;o__Chitinophagales;f__Chitinophagaceae;g__Terrimonas	0.00 ± 0.00	0.0	0.00 ± 0.00	0.0	NA	-	-	-
k__Bacteria;p__Bacteroidetes;c__Cytophagia;o__Cytophagales;f__Cytophagaceae;g__	0.00 ± 0.00	0.0	0.00 ± 0.00	0.0	NA	-	-	-
k__Bacteria;p__Bacteroidetes;c__Cytophagia;o__Cytophagales;f__Cytophagaceae;g__Spirosoma	0.00 ± 0.00	0.0	0.00 ± 0.00	0.0	NA	-	-	-
k__Bacteria;p__Bacteroidetes;c__Cytophagia;o__Cytophagales;f__Hymenobacteraceae;g__Siccationidurans	0.00 ± 0.00	0.0	0.00 ± 0.00	0.0	NA	-	-	-
k__Bacteria;p__Bacteroidetes;c__Flavobacteriia;o__Flavobacteriales;f__Crocinitomicaceae;g__Fluviicola	0.00 ± 0.00	0.0	0.00 ± 0.00	0.0	NA	-	-	-
k__Bacteria;p__Bacteroidetes;c__Flavobacteriia;o__Flavobacteriales;f__Flavobacteriaceae;g__	0.03 ± 0.01	0.0	0.01 ± 0.01	0.0	2.88E-27	2.1982	-	3.14E-28
k__Bacteria;p__Bacteroidetes;c__Flavobacteriia;o__Flavobacteriales;f__Flavobacteriaceae;g__Bergeyella	0.00 ± 0.00	0.0	0.00 ± 0.00	0.0	NA	-	-	-
k__Bacteria;p__Bacteroidetes;c__Flavobacteriia;o__Flavobacteriales;f__Flavobacteriaceae;g__Capnocytophaga	0.00 ± 0.00	0.0	0.00 ± 0.00	0.0	7.54E-01	-	-	-
k__Bacteria;p__Bacteroidetes;c__Flavobacteriia;o__Flavobacteriales;f__Flavobacteriaceae;g__Chryseobacterium	0.00 ± 0.00	0.0	0.00 ± 0.00	0.0	1.86E-02	-	-	1.88E-02
k__Bacteria;p__Bacteroidetes;c__Flavobacteriia;o__Flavobacteriales;f__Flavobacteriaceae;g__Cloacibacterium	0.00 ± 0.00	0.0	0.00 ± 0.00	0.0	9.78E-02	-	-	-
k__Bacteria;p__Bacteroidetes;c__Flavobacteriia;o__Flavobacteriales;f__Flavobacteriaceae;g__Elizabethkingia	0.00 ± 0.00	0.0	0.00 ± 0.00	0.0	NA	-	-	-
k__Bacteria;p__Bacteroidetes;c__Flavobacteriia;o__Flavobacteriales;f__Flavobacteriaceae;g__Empedobacter	0.00 ± 0.00	0.0	0.00 ± 0.00	0.0	2.44E-01	-	-	-
k__Bacteria;p__Bacteroidetes;c__Flavobacteriia;o__Flavobacteriales;f__Flavobacteriaceae;g__Flavobacterium	0.00 ± 0.00	0.0	0.00 ± 0.00	0.0	1.86E-02	-	-	1.88E-02
k__Bacteria;p__Bacteroidetes;c__Flavobacteriia;o__Flavobacteriales;f__Flavobacteriaceae;g__Riemerella	0.00 ± 0.00	0.0	0.00 ± 0.00	0.0	1.86E-02	-	-	1.88E-02
k__Bacteria;p__Bacteroidetes;c__Flavobacteriia;o__Flavobacteriales;f__Flavobacteriaceae;g__Soonwooa	0.00 ± 0.00	0.0	0.00 ± 0.00	0.0	2.44E-01	-	-	-
k__Bacteria;p__Bacteroidetes;c__Sphingobacteriia;o__Sphingobacteriales;f__Chitinophagaceae;g__Taibaiella	0.00 ± 0.00	0.0	0.00 ± 0.00	0.0	NA	-	-	-

k__Bacteria;p__Bacteroidetes;c__Sphingobacteriia;o__Sphingobacteriales;f__Lentimicrobiaceae;g__	0.00 ± 0.00	0.0	0.00 ± 0.00	0.0	NA	-	-	-
k__Bacteria;p__Bacteroidetes;c__Sphingobacteriia;o__Sphingobacteriales;f__Sphingobacteriaceae;g__	0.14 ± 0.02	1.7	0.00 ± 0.00	0.0	9.62E-43	2.8623	-	1.51E-42
k__Bacteria;p__Bacteroidetes;c__Sphingobacteriia;o__Sphingobacteriales;f__Sphingobacteriaceae;g__Mucilaginibacter	0.00 ± 0.00	0.0	0.00 ± 0.00	0.0	NA	-	-	-
k__Bacteria;p__Bacteroidetes;c__Sphingobacteriia;o__Sphingobacteriales;f__Sphingobacteriaceae;g__Pedobacter	0.00 ± 0.00	0.0	0.00 ± 0.00	0.0	NA	-	-	-
k__Bacteria;p__Bacteroidetes;c__Sphingobacteriia;o__Sphingobacteriales;f__Sphingobacteriaceae;g__Sphingobacterium	0.00 ± 0.00	0.0	0.00 ± 0.00	0.0	4.20E-02	-	-	4.22E-02
k__Bacteria;p__Chlamydiae;c__Chlamydiia;o__Parachlamydiales;f__;g__	0.00 ± 0.00	0.0	0.00 ± 0.00	0.0	2.44E-01	-	-	-
k__Bacteria;p__Chlamydiae;c__Chlamydiia;o__Parachlamydiales;f__Parachlamydiaceae;g__Protochlamydia	0.00 ± 0.00	0.0	0.00 ± 0.00	0.0	NA	-	-	-
k__Bacteria;p__Chlorobi;c__Chlorobia;o__Chlorobiales;f__OPB56;g__	0.00 ± 0.00	0.0	0.00 ± 0.00	0.0	NA	-	-	-
k__Bacteria;p__Chloroflexi;c__Anaerolineae;o__Anaerolineales;f__Anaerolineaceae;g__	0.00 ± 0.00	0.0	0.00 ± 0.00	0.0	NA	-	-	-
k__Bacteria;p__Chloroflexi;c__Thermomicrobia;o__Sphaerobacterales;f__Sphaerobacteraceae;g__	0.00 ± 0.00	0.0	0.00 ± 0.00	0.0	NA	-	-	-
k__Bacteria;p__Cyanobacteria;c__Cyanobacteria;o__SubsectionIII;f__FamilyI;g__	0.00 ± 0.00	0.0	0.00 ± 0.00	0.0	NA	-	-	-
k__Bacteria;p__Cyanobacteria;c__Cyanobacteria;o__SubsectionIV;f__FamilyI;g__Trichormus	0.00 ± 0.00	0.0	0.00 ± 0.00	0.0	2.44E-01	-	-	-
k__Bacteria;p__Cyanobacteria;c__Melainabacteria;o__Gastranaerophilales;f__;g__	0.17 ± 0.03	3.5	0.06 ± 0.03	2.5	3.88E-30	2.6703	-	6.03E-30
k__Bacteria;p__Cyanobacteria;c__Melainabacteria;o__Obscuribacterales;f__;g__	0.00 ± 0.00	0.0	0.00 ± 0.00	0.0	NA	-	-	-
k__Bacteria;p__Deferribacteres;c__Deferribacteres;o__Deferribacterales;f__Deferribacteraceae;g__Seleniivibrio	0.00 ± 0.00	0.0	0.00 ± 0.00	0.0	NA	-	-	-
k__Bacteria;p__Deinococcus-Thermus;c__Deinococci;o__Deinococcales;f__Deinococcaceae;g__Deinococcus	0.00 ± 0.00	0.0	0.00 ± 0.00	0.0	NA	-	-	-
k__Bacteria;p__Elusimicrobia;c__Elusimicrobia;o__Elusimicrobiales;f__Elusimicrobiaceae;g__	0.00 ± 0.00	0.0	0.00 ± 0.00	0.0	NA	-	-	-
k__Bacteria;p__Elusimicrobia;c__Elusimicrobia;o__Elusimicrobiales;f__Elusimicrobiaceae;g__Elusimicrobium	0.08 ± 0.06	1.7	0.00 ± 0.00	0.0	1.64E-10	2.6468	-	1.84E-10
k__Bacteria;p__Fibrobacteres;c__Fibrobacteria;o__Fibrobacterales;f__Fibrobacteraceae;g__Fibrobacter	0.01 ± 0.00	0.0	0.00 ± 0.00	0.0	2.42E-14	-	-	2.85E-14
k__Bacteria;p__Firmicutes;c__Bacilli;o__Bacillales;f__Alicyclobacillaceae;g__Alicyclobacillus	0.00 ± 0.00	0.0	0.00 ± 0.00	0.0	5.69E-08	-	-	6.18E-08
k__Bacteria;p__Firmicutes;c__Bacilli;o__Bacillales;f__Bacillaceae;g__	0.00 ± 0.00	0.0	0.00 ± 0.00	0.0	2.95E-11	-	-	3.34E-11
k__Bacteria;p__Firmicutes;c__Bacilli;o__Bacillales;f__Bacillaceae;g__Anoxybacillus	0.00 ± 0.00	0.0	0.00 ± 0.00	0.0	NA	-	-	-
k__Bacteria;p__Firmicutes;c__Bacilli;o__Bacillales;f__Bacillaceae;g__Bacillus	0.00 ± 0.00	0.0	0.00 ± 0.00	0.0	2.44E-01	-	-	-
k__Bacteria;p__Firmicutes;c__Bacilli;o__Bacillales;f__Bacillaceae;g__Geobacillus	0.00 ± 0.00	0.0	0.00 ± 0.00	0.0	NA	-	-	-
k__Bacteria;p__Firmicutes;c__Bacilli;o__Bacillales;f__Bacillales;g__Thermicanus	0.00 ± 0.00	0.0	0.00 ± 0.00	0.0	NA	-	-	-
k__Bacteria;p__Firmicutes;c__Bacilli;o__Bacillales;f__Exiguobacterium;g__	0.00 ± 0.00	0.0	0.00 ± 0.00	0.0	NA	-	-	-
k__Bacteria;p__Firmicutes;c__Bacilli;o__Bacillales;f__Family_XI;g__Gemella	0.00 ± 0.00	0.0	0.00 ± 0.00	0.0	1.48E-13	-	-	1.73E-13
k__Bacteria;p__Firmicutes;c__Bacilli;o__Bacillales;f__Paenibacillaceae;g__	0.02 ± 0.00	0.0	0.00 ± 0.00	0.0	1.11E-23	-	-	1.47E-23
k__Bacteria;p__Firmicutes;c__Bacilli;o__Bacillales;f__Paenibacillaceae;g__Cohnella	0.00 ± 0.00	0.0	0.00 ± 0.00	0.0	NA	-	-	-
k__Bacteria;p__Firmicutes;c__Bacilli;o__Bacillales;f__Paenibacillaceae;g__Paenibacillus	0.00 ± 0.00	0.0	0.00 ± 0.00	0.0	2.44E-01	-	-	-
k__Bacteria;p__Firmicutes;c__Bacilli;o__Bacillales;f__Planococcaceae;g__Kurthia	0.00 ± 0.00	0.0	0.00 ± 0.00	0.0	2.44E-01	-	-	-
k__Bacteria;p__Firmicutes;c__Bacilli;o__Bacillales;f__Staphylococcaceae;g__Jeotgalicoccus	0.00 ± 0.00	0.0	0.00 ± 0.00	0.0	2.44E-01	-	-	-
k__Bacteria;p__Firmicutes;c__Bacilli;o__Bacillales;f__Staphylococcaceae;g__Macrococcus	0.00 ± 0.00	0.0	0.00 ± 0.00	0.0	4.20E-02	-	-	4.22E-02
k__Bacteria;p__Firmicutes;c__Bacilli;o__Bacillales;f__Staphylococcaceae;g__Staphylococcus	0.01 ± 0.00	0.0	0.00 ± 0.00	0.0	1.76E-06	-	-	1.89E-06
k__Bacteria;p__Firmicutes;c__Bacilli;o__Bacillales;f__Thermoactinomycetaceae;g__	0.00 ± 0.00	0.0	0.00 ± 0.00	0.0	5.69E-08	-	-	6.18E-08
k__Bacteria;p__Firmicutes;c__Bacilli;o__Lactobacillales;f__Aerococcaceae;g__	0.00 ± 0.00	0.0	0.00 ± 0.00	0.0	NA	-	-	-
k__Bacteria;p__Firmicutes;c__Bacilli;o__Lactobacillales;f__Aerococcaceae;g__Abiotrophia	0.00 ± 0.00	0.0	0.00 ± 0.00	0.0	4.20E-02	-	-	4.22E-02
k__Bacteria;p__Firmicutes;c__Bacilli;o__Lactobacillales;f__Aerococcaceae;g__Aerococcus	0.00 ± 0.00	0.0	0.00 ± 0.00	0.0	3.62E-04	-	-	3.74E-04
k__Bacteria;p__Firmicutes;c__Bacilli;o__Lactobacillales;f__Aerococcaceae;g__Dolosicoccus	0.00 ± 0.00	0.0	0.00 ± 0.00	0.0	NA	-	-	-
k__Bacteria;p__Firmicutes;c__Bacilli;o__Lactobacillales;f__Aerococcaceae;g__Eremococcus	0.00 ± 0.00	0.0	0.00 ± 0.00	0.0	9.78E-02	-	-	-
k__Bacteria;p__Firmicutes;c__Bacilli;o__Lactobacillales;f__Aerococcaceae;g__Facklamia	0.00 ± 0.00	0.0	0.00 ± 0.00	0.0	7.11E-06	-	-	7.51E-06
k__Bacteria;p__Firmicutes;c__Bacilli;o__Lactobacillales;f__Carnobacteriaceae;g__Alkalibacterium	0.00 ± 0.00	0.0	0.00 ± 0.00	0.0	NA	-	-	-
k__Bacteria;p__Firmicutes;c__Bacilli;o__Lactobacillales;f__Carnobacteriaceae;g__Atopostipes	0.00 ± 0.00	0.0	0.00 ± 0.00	0.0	2.44E-01	-	-	-
k__Bacteria;p__Firmicutes;c__Bacilli;o__Lactobacillales;f__Carnobacteriaceae;g__Dolosigranulum	0.00 ± 0.00	0.0	0.00 ± 0.00	0.0	NA	-	-	-
k__Bacteria;p__Firmicutes;c__Bacilli;o__Lactobacillales;f__Carnobacteriaceae;g__Granulicatella	0.00 ± 0.00	0.0	0.00 ± 0.00	0.0	1.20E-09	-	-	1.36E-09
k__Bacteria;p__Firmicutes;c__Bacilli;o__Lactobacillales;f__Enterococcaceae;g__Enterococcus	0.00 ± 0.00	0.0	0.00 ± 0.00	0.0	9.10E-01	-	-	-
k__Bacteria;p__Firmicutes;c__Bacilli;o__Lactobacillales;f__Lactobacillaceae;g__Lactobacillus	0.43 ± 0.06	7.0	0.33 ± 0.07	5.7	1.34E-06	2.6879	-	1.21E-06
k__Bacteria;p__Firmicutes;c__Bacilli;o__Lactobacillales;f__Lactobacillaceae;g__Pediococcus	0.00 ± 0.00	0.0	0.00 ± 0.00	0.0	9.78E-02	-	-	-
k__Bacteria;p__Firmicutes;c__Bacilli;o__Lactobacillales;f__Leuconostocaceae;g__Leuconostoc	0.00 ± 0.00	0.0	0.00 ± 0.00	0.0	3.80E-03	-	-	3.87E-03
k__Bacteria;p__Firmicutes;c__Bacilli;o__Lactobacillales;f__Leuconostocaceae;g__Weissella	0.01 ± 0.00	0.0	0.00 ± 0.00	0.0	2.64E-09	-	-	2.94E-09
k__Bacteria;p__Firmicutes;c__Bacilli;o__Lactobacillales;f__Streptococcaceae;g__Lactococcus	0.00 ± 0.00	0.0	0.00 ± 0.00	0.0	1.07E-02	-	-	1.10E-02
k__Bacteria;p__Firmicutes;c__Bacilli;o__Lactobacillales;f__Streptococcaceae;g__Streptococcus	0.18 ± 0.03	2.6	0.03 ± 0.01	0.0	8.57E-21	2.8867	-	1.34E-20
k__Bacteria;p__Firmicutes;c__Clostridia;o__Clostridiales;f__;g__	0.04 ± 0.01	0.0	0.31 ± 0.11	6.3	4.07E-04	-	3.1597	4.88E-04
k__Bacteria;p__Firmicutes;c__Clostridia;o__Clostridiales;f__Bacteroides;g__pectinophilus	0.02 ± 0.00	0.0	0.00 ± 0.00	0.0	1.72E-16	-	-	2.14E-16
k__Bacteria;p__Firmicutes;c__Clostridia;o__Clostridiales;f__Caldicoprobacteraceae;g__	0.01 ± 0.00	0.0	0.02 ± 0.02	0.6	4.45E-14	-	-	5.34E-14
k__Bacteria;p__Firmicutes;c__Clostridia;o__Clostridiales;f__Christensenellaceae;g__	0.09 ± 0.02	0.9	0.07 ± 0.02	1.9	2.76E-08	2.1849	-	3.75E-08
k__Bacteria;p__Firmicutes;c__Clostridia;o__Clostridiales;f__Christensenellaceae;g__Christensenella	0.00 ± 0.00	0.0	0.01 ± 0.01	0.0	2.25E-03	-	-	2.41E-03
k__Bacteria;p__Firmicutes;c__Clostridia;o__Clostridiales;f__Christensenellaceae;g__Christensenellaceae_R-7_group	0.35 ± 0.04	9.6	0.15 ± 0.02	1.9	4.13E-12	3.0314	-	5.86E-12
k__Bacteria;p__Firmicutes;c__Clostridia;o__Clostridiales;f__Clostridiaceae;g__	0.06 ± 0.03	0.9	0.06 ± 0.02	1.3	1.03E-08	-	2.2549	1.31E-08
k__Bacteria;p__Firmicutes;c__Clostridia;o__Clostridiales;f__Clostridiaceae;g__Beduini	0.05 ± 0.01	0.0	0.00 ± 0.00	0.0	1.44E-27	2.3852	-	1.96E-27
k__Bacteria;p__Firmicutes;c__Clostridia;o__Clostridiales;f__Clostridiaceae;g__Butyricicoccus	0.16 ± 0.02	1.7	0.09 ± 0.01	1.3	2.58E-09	2.5856	-	1.33E-09
k__Bacteria;p__Firmicutes;c__Clostridia;o__Clostridiales;f__Clostridiaceae;g__Caloramator	0.00 ± 0.00	0.0	0.00 ± 0.00	0.0	1.86E-02	-	-	1.88E-02
k__Bacteria;p__Firmicutes;c__Clostridia;o__Clostridiales;f__Clostridiaceae;g__Clostridium	0.95 ± 0.21	18.3	0.04 ± 0.01	0.6	3.39E-35	3.6431	-	5.51E-35

k__Bacteria;p__Firmicutes;c__Clostridia;o__Clostridiales;f__Clostridiaceae;g__Hungatella	0.24 ± 0.03	5.2	0.06 ± 0.02	0.6	4.77E-30	2.9961	-	7.72E-30
k__Bacteria;p__Firmicutes;c__Clostridia;o__Clostridiales;f__Clostridiaceae;g__Lactonifactor	0.00 ± 0.00	0.0	0.00 ± 0.00	0.0	2.29E-01	-	-	-
k__Bacteria;p__Firmicutes;c__Clostridia;o__Clostridiales;f__Clostridiaceae;g__Oxobacter	0.00 ± 0.00	0.0	0.00 ± 0.00	0.0	4.20E-02	-	-	4.22E-02
k__Bacteria;p__Firmicutes;c__Clostridia;o__Clostridiales;f__Clostridiales;g__	0.03 ± 0.01	0.0	0.00 ± 0.00	0.0	1.38E-17	2.1337	-	1.71E-17
k__Bacteria;p__Firmicutes;c__Clostridia;o__Clostridiales;f__Clostridiales;g__Anaerovorax	0.06 ± 0.01	0.0	0.00 ± 0.00	0.0	1.19E-32	2.4387	-	5.52E-33
k__Bacteria;p__Firmicutes;c__Clostridia;o__Clostridiales;f__Clostridiales;g__Casaltella	0.00 ± 0.00	0.0	0.00 ± 0.00	0.0	7.57E-05	-	-	7.89E-05
k__Bacteria;p__Firmicutes;c__Clostridia;o__Clostridiales;f__Clostridiales;g__Emergencia	0.02 ± 0.01	0.0	0.02 ± 0.01	0.6	1.54E-14	-	-	1.96E-14
k__Bacteria;p__Firmicutes;c__Clostridia;o__Clostridiales;f__Clostridiales;g__Eubacterium	0.00 ± 0.00	0.0	0.00 ± 0.00	0.0	9.78E-02	-	-	-
k__Bacteria;p__Firmicutes;c__Clostridia;o__Clostridiales;f__Clostridiales;g__Ihubacter	0.00 ± 0.00	0.0	0.01 ± 0.00	0.0	1.14E-01	-	-	-
k__Bacteria;p__Firmicutes;c__Clostridia;o__Clostridiales;f__Clostridiales;g__Mogibacterium	0.00 ± 0.00	0.0	0.00 ± 0.00	0.0	8.97E-10	-	-	9.97E-10
k__Bacteria;p__Firmicutes;c__Clostridia;o__Clostridiales;f__Defluviitaleaceae;g__	0.00 ± 0.00	0.0	0.00 ± 0.00	0.0	2.08E-09	-	-	2.30E-09
k__Bacteria;p__Firmicutes;c__Clostridia;o__Clostridiales;f__Defluviitaleaceae;g__Defluviitalea	0.00 ± 0.00	0.0	0.00 ± 0.00	0.0	9.78E-02	-	-	-
k__Bacteria;p__Firmicutes;c__Clostridia;o__Clostridiales;f__Defluviitaleaceae;g__Vallitalea	0.01 ± 0.00	0.0	0.00 ± 0.00	0.0	5.17E-12	-	-	5.91E-12
k__Bacteria;p__Firmicutes;c__Clostridia;o__Clostridiales;f__Eubacteriaceae;g__	0.00 ± 0.00	0.0	0.00 ± 0.00	0.0	NA	-	-	-
k__Bacteria;p__Firmicutes;c__Clostridia;o__Clostridiales;f__Eubacteriaceae;g__Anaerofustis	0.00 ± 0.00	0.0	0.00 ± 0.00	0.0	2.44E-01	-	-	-
**k__Bacteria;p__Firmicutes;c__Clostridia;o__Clostridiales;f__Eubacteriaceae;g__Eubacterium**	**1.49 ± 0.11**	**61.7**	**1.17 ± 0.10**	**42.4**	**2.25E-03**	**3.1842**	**-**	**2.29E-03**
k__Bacteria;p__Firmicutes;c__Clostridia;o__Clostridiales;f__Family_XI;g__Murdochiella	0.00 ± 0.00	0.0	0.00 ± 0.00	0.0	3.98E-01	-	-	-
k__Bacteria;p__Firmicutes;c__Clostridia;o__Clostridiales;f__Family_XIII;g__Family_XIII_UCG-001	0.00 ± 0.00	0.0	0.00 ± 0.00	0.0	2.44E-01	-	-	-
k__Bacteria;p__Firmicutes;c__Clostridia;o__Clostridiales;f__Fenollaria;g__massiliensis	0.01 ± 0.00	0.0	0.00 ± 0.00	0.0	1.40E-04	-	-	1.48E-04
k__Bacteria;p__Firmicutes;c__Clostridia;o__Clostridiales;f__Flavonifractor;g__	0.01 ± 0.00	0.0	0.08 ± 0.01	0.6	1.84E-04	-	2.5790	2.43E-04
k__Bacteria;p__Firmicutes;c__Clostridia;o__Clostridiales;f__Flavonifractor;g__plautii	0.00 ± 0.00	0.0	0.09 ± 0.02	2.5	3.73E-11	-	2.6646	5.42E-11
k__Bacteria;p__Firmicutes;c__Clostridia;o__Clostridiales;f__Flintibacter;g__	0.15 ± 0.02	0.9	0.00 ± 0.00	0.0	2.64E-50	2.8950	-	4.43E-50
k__Bacteria;p__Firmicutes;c__Clostridia;o__Clostridiales;f__Gracilibacteraceae;g__	0.02 ± 0.00	0.0	0.05 ± 0.02	0.6	2.02E-09	-	2.1836	2.54E-09
k__Bacteria;p__Firmicutes;c__Clostridia;o__Clostridiales;f__Gracilibacteraceae;g__Gracilibacter	0.01 ± 0.00	0.0	0.06 ± 0.02	1.3	6.09E-02	-	2.4309	4.32E-02
k__Bacteria;p__Firmicutes;c__Clostridia;o__Clostridiales;f__Heliobacteriaceae;g__	0.01 ± 0.00	0.0	0.00 ± 0.00	0.0	2.42E-14	-	-	2.85E-14
k__Bacteria;p__Firmicutes;c__Clostridia;o__Clostridiales;f__Howardella;g__	0.00 ± 0.00	0.0	0.00 ± 0.00	0.0	2.44E-01	-	-	-
k__Bacteria;p__Firmicutes;c__Clostridia;o__Clostridiales;f__Howardella;g__ureilytica	0.00 ± 0.00	0.0	0.01 ± 0.00	0.0	4.38E-02	-	-	4.24E-02
k__Bacteria;p__Firmicutes;c__Clostridia;o__Clostridiales;f__Intestinimonas;g__	0.37 ± 0.03	5.2	0.10 ± 0.02	1.9	3.79E-21	3.1423	-	4.56E-21
k__Bacteria;p__Firmicutes;c__Clostridia;o__Clostridiales;f__Lachnospiraceae;g__	0.65 ± 0.05	21.7	0.59 ± 0.08	19.0	2.18E-04	2.6358	-	2.50E-04
k__Bacteria;p__Firmicutes;c__Clostridia;o__Clostridiales;f__Lachnospiraceae;g__Acetatifactor	0.00 ± 0.00	0.0	0.00 ± 0.00	0.0	5.06E-18	-	-	6.22E-18
k__Bacteria;p__Firmicutes;c__Clostridia;o__Clostridiales;f__Lachnospiraceae;g__Acetitomaculum	0.00 ± 0.00	0.0	0.01 ± 0.01	0.0	6.13E-03	-	-	5.93E-03
k__Bacteria;p__Firmicutes;c__Clostridia;o__Clostridiales;f__Lachnospiraceae;g__Anaerobium	0.07 ± 0.02	3.5	0.00 ± 0.00	0.0	2.67E-19	2.5728	-	3.32E-19
k__Bacteria;p__Firmicutes;c__Clostridia;o__Clostridiales;f__Lachnospiraceae;g__Anaerocolumna	0.00 ± 0.00	0.0	0.00 ± 0.00	0.0	1.29E-07	-	-	1.39E-07
k__Bacteria;p__Firmicutes;c__Clostridia;o__Clostridiales;f__Lachnospiraceae;g__Anaerosporobacter	0.15 ± 0.02	3.5	0.00 ± 0.00	0.0	2.07E-46	2.8815	-	3.36E-46
k__Bacteria;p__Firmicutes;c__Clostridia;o__Clostridiales;f__Lachnospiraceae;g__Anaerostipes	0.87 ± 0.13	24.3	0.11 ± 0.02	1.3	1.58E-20	3.5756	-	1.24E-20
**k__Bacteria;p__Firmicutes;c__Clostridia;o__Clostridiales;f__Lachnospiraceae;g__Blautia**	**1.47 ± 0.12**	**49.6**	**0.77 ± 0.10**	**19.0**	**9.82E-10**	**3.5451**	**-**	**8.34E-10**
k__Bacteria;p__Firmicutes;c__Clostridia;o__Clostridiales;f__Lachnospiraceae;g__Butyrivibrio	0.14 ± 0.03	1.7	0.20 ± 0.09	3.2	3.38E-28	-	2.6395	5.07E-28
k__Bacteria;p__Firmicutes;c__Clostridia;o__Clostridiales;f__Lachnospiraceae;g__Catonella	0.00 ± 0.00	0.0	0.00 ± 0.00	0.0	9.78E-02	-	-	-
k__Bacteria;p__Firmicutes;c__Clostridia;o__Clostridiales;f__Lachnospiraceae;g__Cellulosilyticum	0.00 ± 0.00	0.0	0.00 ± 0.00	0.0	3.80E-03	-	-	3.87E-03
**k__Bacteria;p__Firmicutes;c__Clostridia;o__Clostridiales;f__Lachnospiraceae;g__[Clostridium]**	**2.13 ± 0.16**	**68.7**	**0.44 ± 0.05**	**11.4**	**1.25E-24**	**3.9203**	**-**	**1.02E-24**
k__Bacteria;p__Firmicutes;c__Clostridia;o__Clostridiales;f__Lachnospiraceae;g__Coprococcus	0.27 ± 0.03	2.6	0.32 ± 0.05	6.3	2.97E-02	-	2.6062	3.33E-02
k__Bacteria;p__Firmicutes;c__Clostridia;o__Clostridiales;f__Lachnospiraceae;g__Cuneatibacter	0.05 ± 0.01	0.0	0.00 ± 0.00	0.0	5.73E-41	2.3770	-	8.88E-41
k__Bacteria;p__Firmicutes;c__Clostridia;o__Clostridiales;f__Lachnospiraceae;g__Desulfotomaculum	0.54 ± 0.06	12.2	0.08 ± 0.01	0.6	1.30E-29	3.3410	-	1.00E-29
k__Bacteria;p__Firmicutes;c__Clostridia;o__Clostridiales;f__Lachnospiraceae;g__Dorea	0.57 ± 0.05	20.0	0.20 ± 0.02	2.5	2.14E-14	3.2620	-	2.23E-14
k__Bacteria;p__Firmicutes;c__Clostridia;o__Clostridiales;f__Lachnospiraceae;g__Eisenbergiella	0.04 ± 0.01	0.0	0.30 ± 0.04	8.2	1.06E-04	-	3.1164	1.25E-04
**k__Bacteria;p__Firmicutes;c__Clostridia;o__Clostridiales;f__Lachnospiraceae;g__[Eubacterium]**	**1.10 ± 0.12**	**38.3**	**2.29 ± 0.31**	**45.6**	**2.37E-02**	**-**	**3.8016**	**2.34E-02**
k__Bacteria;p__Firmicutes;c__Clostridia;o__Clostridiales;f__Lachnospiraceae;g__Frisingicoccus	0.00 ± 0.00	0.0	0.01 ± 0.00	0.0	6.13E-03	-	-	5.93E-03
k__Bacteria;p__Firmicutes;c__Clostridia;o__Clostridiales;f__Lachnospiraceae;g__Fusicatenibacter	0.09 ± 0.01	0.9	0.20 ± 0.03	3.2	6.08E-01	-	-	-
k__Bacteria;p__Firmicutes;c__Clostridia;o__Clostridiales;f__Lachnospiraceae;g__Hespellia	0.00 ± 0.00	0.0	0.00 ± 0.00	0.0	7.57E-05	-	-	7.89E-05
k__Bacteria;p__Firmicutes;c__Clostridia;o__Clostridiales;f__Lachnospiraceae;g__Johnsonella	0.00 ± 0.00	0.0	0.00 ± 0.00	0.0	NA	-	-	-
k__Bacteria;p__Firmicutes;c__Clostridia;o__Clostridiales;f__Lachnospiraceae;g__Lachnoanaerobaculum	0.00 ± 0.00	0.0	0.00 ± 0.00	0.0	4.20E-02	2.0830	-	4.22E-02
k__Bacteria;p__Firmicutes;c__Clostridia;o__Clostridiales;f__Lachnospiraceae;g__Lachnospira	0.04 ± 0.01	0.0	0.23 ± 0.05	5.7	4.52E-01	-	-	-
k__Bacteria;p__Firmicutes;c__Clostridia;o__Clostridiales;f__Lachnospiraceae;g__Lachnospiraceae_ND3007_group	0.00 ± 0.00	0.0	0.00 ± 0.00	0.0	1.66E-04	-	-	1.72E-04
k__Bacteria;p__Firmicutes;c__Clostridia;o__Clostridiales;f__Lachnospiraceae;g__Moryella	0.00 ± 0.00	0.0	0.00 ± 0.00	0.0	NA	-	-	-
k__Bacteria;p__Firmicutes;c__Clostridia;o__Clostridiales;f__Lachnospiraceae;g__Muricomes	0.00 ± 0.00	0.0	0.00 ± 0.00	0.0	4.20E-02	-	-	4.22E-02
k__Bacteria;p__Firmicutes;c__Clostridia;o__Clostridiales;f__Lachnospiraceae;g__Murimonas	0.05 ± 0.01	0.0	0.08 ± 0.01	0.0	8.03E-02	-	-	-
k__Bacteria;p__Firmicutes;c__Clostridia;o__Clostridiales;f__Lachnospiraceae;g__Oribacterium	0.00 ± 0.00	0.0	0.00 ± 0.00	0.0	8.36E-03	-	-	8.49E-03
k__Bacteria;p__Firmicutes;c__Clostridia;o__Clostridiales;f__Lachnospiraceae;g__Pseudobutyrivibrio	0.02 ± 0.00	0.0	0.00 ± 0.00	0.0	4.05E-32	2.0702	-	5.78E-32
**k__Bacteria;p__Firmicutes;c__Clostridia;o__Clostridiales;f__Lachnospiraceae;g__Roseburia**	**1.30 ± 0.18**	**36.5**	**0.40 ± 0.05**	**12.7**	**3.52E-07**	**3.6267**	**-**	**3.87E-07**
k__Bacteria;p__Firmicutes;c__Clostridia;o__Clostridiales;f__Lachnospiraceae;g__Ruminococcus	0.00 ± 0.00	0.0	0.47 ± 0.10	10.1	9.07E-26	-	3.3785	1.24E-25
k__Bacteria;p__Firmicutes;c__Clostridia;o__Clostridiales;f__Lachnospiraceae;g__Shuttleworthia	0.00 ± 0.00	0.0	0.00 ± 0.00	0.0	NA	-	-	-
k__Bacteria;p__Firmicutes;c__Clostridia;o__Clostridiales;f__Lachnospiraceae;g__Stomatobaculum	0.00 ± 0.00	0.0	0.00 ± 0.00	0.0	2.44E-01	-	-	-
k__Bacteria;p__Firmicutes;c__Clostridia;o__Clostridiales;f__Lachnospiraceae;g__Tyzzerella	0.00 ± 0.00	0.0	0.00 ± 0.00	0.0	9.78E-02	-	-	-

k__Bacteria;p__Firmicutes;c__Clostridia;o__Clostridiales;f__Levyella;g__	0.00 ± 0.00	0.0	0.00 ± 0.00	0.0	9.78E-02	-	-	-
k__Bacteria;p__Firmicutes;c__Clostridia;o__Clostridiales;f__Oscillospiraceae;g__	0.00 ± 0.00	0.0	0.00 ± 0.00	0.0	2.44E-01	-	-	-
**k__Bacteria;p__Firmicutes;c__Clostridia;o__Clostridiales;f__Oscillospiraceae;g__Oscillibacter**	**0.71 ± 0.11**	**14.8**	**3.10 ± 0.35**	**65.2**	**5.86E-15**	**-**	**4.0853**	**8.16E-15**
k__Bacteria;p__Firmicutes;c__Clostridia;o__Clostridiales;f__Peptococcaceae;g__	0.08 ± 0.01	0.9	0.39 ± 0.08	12.7	1.76E-03	-	3.1774	1.78E-03
k__Bacteria;p__Firmicutes;c__Clostridia;o__Clostridiales;f__Peptococcaceae;g__Peptococcus	0.01 ± 0.00	0.0	0.00 ± 0.00	0.0	1.78E-17	-	-	2.19E-17
k__Bacteria;p__Firmicutes;c__Clostridia;o__Clostridiales;f__Peptostreptococcaceae;g__	0.00 ± 0.00	0.0	0.00 ± 0.00	0.0	1.66E-04	-	-	1.72E-04
k__Bacteria;p__Firmicutes;c__Clostridia;o__Clostridiales;f__Peptostreptococcaceae;g__Clostridioides	0.00 ± 0.00	0.0	0.00 ± 0.00	0.0	9.78E-02	-	-	-
k__Bacteria;p__Firmicutes;c__Clostridia;o__Clostridiales;f__Peptostreptococcaceae;g__Eubacterium	0.00 ± 0.00	0.0	0.00 ± 0.00	0.0	2.44E-01	-	-	-
k__Bacteria;p__Firmicutes;c__Clostridia;o__Clostridiales;f__Peptostreptococcaceae;g__Filifactor	0.00 ± 0.00	0.0	0.00 ± 0.00	0.0	2.44E-01	-	-	-
k__Bacteria;p__Firmicutes;c__Clostridia;o__Clostridiales;f__Peptostreptococcaceae;g__Intestinibacter	0.03 ± 0.01	0.0	0.00 ± 0.00	0.0	7.50E-21	2.0620	-	9.67E-21
k__Bacteria;p__Firmicutes;c__Clostridia;o__Clostridiales;f__Peptostreptococcaceae;g__Peptostreptococcus	0.00 ± 0.00	0.0	0.00 ± 0.00	0.0	8.87E-03	-	-	9.07E-03
k__Bacteria;p__Firmicutes;c__Clostridia;o__Clostridiales;f__Peptostreptococcaceae;g__Romboutsia	0.01 ± 0.00	0.0	0.04 ± 0.01	0.6	3.74E-02	-	2.2764	3.51E-02
k__Bacteria;p__Firmicutes;c__Clostridia;o__Clostridiales;f__Peptostreptococcaceae;g__Terrisporobacter	0.02 ± 0.01	0.9	0.00 ± 0.00	0.0	1.10E-08	-	-	1.20E-08
k__Bacteria;p__Firmicutes;c__Clostridia;o__Clostridiales;f__Pseudoflavonifractor;g__	0.01 ± 0.00	0.0	0.00 ± 0.00	0.0	3.91E-02	-	-	3.96E-02
k__Bacteria;p__Firmicutes;c__Clostridia;o__Clostridiales;f__Pseudoflavonifractor;g__capillosus	0.01 ± 0.00	0.0	0.00 ± 0.00	0.0	8.37E-19	-	-	1.09E-18
k__Bacteria;p__Firmicutes;c__Clostridia;o__Clostridiales;f__Ruminococcaceae;g__	1.37 ± 0.16	47.8	1.98 ± 0.26	50.0	9.42E-01	-	-	-
k__Bacteria;p__Firmicutes;c__Clostridia;o__Clostridiales;f__Ruminococcaceae;g__Acetanaerobacterium	0.01 ± 0.01	0.0	0.44 ± 0.13	8.9	5.15E-02	-	3.3306	4.63E-02
k__Bacteria;p__Firmicutes;c__Clostridia;o__Clostridiales;f__Ruminococcaceae;g__Acetivibrio	0.05 ± 0.01	0.9	0.04 ± 0.01	0.0	6.58E-05	-	-	4.06E-05
k__Bacteria;p__Firmicutes;c__Clostridia;o__Clostridiales;f__Ruminococcaceae;g__Acutalibacter	0.00 ± 0.00	0.0	0.00 ± 0.00	0.0	7.11E-06	-	-	7.51E-06
k__Bacteria;p__Firmicutes;c__Clostridia;o__Clostridiales;f__Ruminococcaceae;g__Anaerobacterium	0.01 ± 0.00	0.0	0.01 ± 0.00	0.0	2.65E-10	-	-	3.08E-10
k__Bacteria;p__Firmicutes;c__Clostridia;o__Clostridiales;f__Ruminococcaceae;g__Anaerofilum	0.09 ± 0.01	0.0	0.02 ± 0.01	0.0	5.48E-28	2.5956	-	8.46E-28
k__Bacteria;p__Firmicutes;c__Clostridia;o__Clostridiales;f__Ruminococcaceae;g__Anaeromassilibacillus	0.00 ± 0.00	0.0	0.01 ± 0.00	0.0	1.66E-03	-	-	1.58E-03
k__Bacteria;p__Firmicutes;c__Clostridia;o__Clostridiales;f__Ruminococcaceae;g__Anaerotruncus	0.05 ± 0.01	0.0	0.05 ± 0.01	0.0	3.32E-02	-	-	2.97E-02
k__Bacteria;p__Firmicutes;c__Clostridia;o__Clostridiales;f__Ruminococcaceae;g__Caproiciproducens	0.02 ± 0.00	0.0	0.00 ± 0.00	0.0	4.94E-22	-	-	6.57E-22
k__Bacteria;p__Firmicutes;c__Clostridia;o__Clostridiales;f__Ruminococcaceae;g__Clostridium	0.29 ± 0.03	5.2	0.13 ± 0.02	1.9	4.84E-09	2.9454	-	4.24E-09
k__Bacteria;p__Firmicutes;c__Clostridia;o__Clostridiales;f__Ruminococcaceae;g__Drancourtella	0.00 ± 0.00	0.0	0.00 ± 0.00	0.0	8.69E-02	-	-	-
k__Bacteria;p__Firmicutes;c__Clostridia;o__Clostridiales;f__Ruminococcaceae;g__Ethanoligenens	0.07 ± 0.01	0.9	0.01 ± 0.00	0.0	8.76E-22	2.4346	-	1.27E-21
k__Bacteria;p__Firmicutes;c__Clostridia;o__Clostridiales;f__Ruminococcaceae;g__Eubacterium	0.10 ± 0.02	0.9	0.14 ± 0.03	2.5	1.99E-04	-	2.3687	2.47E-04
k__Bacteria;p__Firmicutes;c__Clostridia;o__Clostridiales;f__Ruminococcaceae;g__Faecalibacterium	4.80 ± 0.40	84.3	4.55 ± 0.42	73.4	8.48E-02	-	-	-
k__Bacteria;p__Firmicutes;c__Clostridia;o__Clostridiales;f__Ruminococcaceae;g__Fastidiosipila	0.00 ± 0.00	0.0	0.00 ± 0.00	0.0	9.78E-02	-	-	-
k__Bacteria;p__Firmicutes;c__Clostridia;o__Clostridiales;f__Ruminococcaceae;g__Gemmiger	0.29 ± 0.03	3.5	0.98 ± 0.15	23.4	9.08E-03	-	3.5429	8.19E-03
k__Bacteria;p__Firmicutes;c__Clostridia;o__Clostridiales;f__Ruminococcaceae;g__Neglecta	0.01 ± 0.00	0.0	0.10 ± 0.03	1.3	2.93E-01	-	-	-
k__Bacteria;p__Firmicutes;c__Clostridia;o__Clostridiales;f__Ruminococcaceae;g__Papillibacter	0.02 ± 0.00	0.0	0.00 ± 0.00	0.0	1.22E-25	-	-	1.63E-25
k__Bacteria;p__Firmicutes;c__Clostridia;o__Clostridiales;f__Ruminococcaceae;g__Phocea	0.00 ± 0.00	0.0	0.02 ± 0.01	0.6	3.57E-06	-	2.0101	3.24E-06
k__Bacteria;p__Firmicutes;c__Clostridia;o__Clostridiales;f__Ruminococcaceae;g__Ruminiclostridium	0.02 ± 0.00	0.0	0.01 ± 0.00	0.0	3.37E-17	-	-	4.49E-17
k__Bacteria;p__Firmicutes;c__Clostridia;o__Clostridiales;f__Ruminococcaceae;g__Ruminococcus	0.98 ± 0.12	31.3	1.86 ± 0.30	46.8	2.95E-01	-	-	-
k__Bacteria;p__Firmicutes;c__Clostridia;o__Clostridiales;f__Ruminococcaceae;g__Saccharofermentans	0.01 ± 0.01	0.0	0.00 ± 0.00	0.0	6.98E-11	-	-	7.87E-11
k__Bacteria;p__Firmicutes;c__Clostridia;o__Clostridiales;f__Ruminococcaceae;g__Sporobacter	0.62 ± 0.08	20.0	0.23 ± 0.06	5.1	2.20E-18	3.2999	-	3.19E-18
k__Bacteria;p__Firmicutes;c__Clostridia;o__Clostridiales;f__Ruminococcaceae;g__Subdoligranulum	0.02 ± 0.00	0.0	0.00 ± 0.00	0.0	1.57E-37	2.0498	-	2.36E-37
k__Bacteria;p__Firmicutes;c__Clostridia;o__Clostridiales;f__Syntrophomonadaceae;g__	0.00 ± 0.00	0.0	0.00 ± 0.00	0.0	NA	-	-	-
k__Bacteria;p__Firmicutes;c__Clostridia;o__Clostridiales;f__Syntrophomonadaceae;g__Pelospora	0.00 ± 0.00	0.0	0.00 ± 0.00	0.0	NA	-	-	-
k__Bacteria;p__Firmicutes;c__Clostridia;o__Clostridiales;f__Syntrophomonadaceae;g__Syntrophomonas	0.00 ± 0.00	0.0	0.00 ± 0.00	0.0	1.73E-03	-	-	1.77E-03
k__Bacteria;p__Firmicutes;c__Clostridia;o__Thermoanaerobacterales;f__Thermoanaerobacteraceae;g__	0.00 ± 0.00	0.0	0.00 ± 0.00	0.0	1.39E-01	-	-	-
k__Bacteria;p__Firmicutes;c__Erysipelotrichia;o__Erysipelotrichales;f__Erysipelotrichaceae;g__	0.16 ± 0.04	2.6	0.04 ± 0.02	1.3	7.08E-34	2.8392	-	3.91E-34
k__Bacteria;p__Firmicutes;c__Erysipelotrichia;o__Erysipelotrichales;f__Erysipelotrichaceae;g__Bulleidia	0.05 ± 0.01	0.0	0.00 ± 0.00	0.0	2.41E-43	2.3910	-	3.82E-43
k__Bacteria;p__Firmicutes;c__Erysipelotrichia;o__Erysipelotrichales;f__Erysipelotrichaceae;g__Catenibacterium	0.44 ± 0.09	12.2	0.03 ± 0.01	0.6	3.15E-34	3.3038	-	4.87E-34
k__Bacteria;p__Firmicutes;c__Erysipelotrichia;o__Erysipelotrichales;f__Erysipelotrichaceae;g__Clostridium	0.26 ± 0.03	2.6	0.24 ± 0.03	5.1	1.06E-01	-	-	-
k__Bacteria;p__Firmicutes;c__Erysipelotrichia;o__Erysipelotrichales;f__Erysipelotrichaceae;g__Coprobacillus	0.00 ± 0.00	0.0	0.00 ± 0.00	0.0	3.52E-02	-	-	3.44E-02
k__Bacteria;p__Firmicutes;c__Erysipelotrichia;o__Erysipelotrichales;f__Erysipelotrichaceae;g__Dielma	0.00 ± 0.00	0.0	0.02 ± 0.01	0.6	1.65E-02	-	-	1.60E-02
k__Bacteria;p__Firmicutes;c__Erysipelotrichia;o__Erysipelotrichales;f__Erysipelotrichaceae;g__Eggerthia	0.00 ± 0.00	0.0	0.00 ± 0.00	0.0	NA	-	-	-
k__Bacteria;p__Firmicutes;c__Erysipelotrichia;o__Erysipelotrichales;f__Erysipelotrichaceae;g__Erysipelatoclostridium	0.00 ± 0.00	0.0	0.00 ± 0.00	0.0	2.08E-01	-	-	-
k__Bacteria;p__Firmicutes;c__Erysipelotrichia;o__Erysipelotrichales;f__Erysipelotrichaceae;g__Erysipelotrichaceae_UCG-004	0.00 ± 0.00	0.0	0.00 ± 0.00	0.0	7.57E-05	-	-	7.89E-05
k__Bacteria;p__Firmicutes;c__Erysipelotrichia;o__Erysipelotrichales;f__Erysipelotrichaceae;g__Eubacterium	0.00 ± 0.00	0.0	0.01 ± 0.01	0.0	2.26E-02	-	-	2.20E-02
k__Bacteria;p__Firmicutes;c__Erysipelotrichia;o__Erysipelotrichales;f__Erysipelotrichaceae;g__Faecalitalea	0.00 ± 0.00	0.0	0.01 ± 0.01	0.6	1.39E-01	-	-	-
k__Bacteria;p__Firmicutes;c__Erysipelotrichia;o__Erysipelotrichales;f__Erysipelotrichaceae;g__Holdemanella	0.04 ± 0.01	0.0	0.08 ± 0.02	3.2	9.34E-19	-	2.3028	6.19E-19
k__Bacteria;p__Firmicutes;c__Erysipelotrichia;o__Erysipelotrichales;f__Erysipelotrichaceae;g__Holdemania	0.00 ± 0.00	0.0	0.01 ± 0.00	0.0	2.94E-05	-	-	2.73E-05
k__Bacteria;p__Firmicutes;c__Erysipelotrichia;o__Erysipelotrichales;f__Erysipelotrichaceae;g__Longibaculum	0.11 ± 0.01	0.0	0.00 ± 0.00	0.0	8.67E-46	2.7313	-	1.40E-45
k__Bacteria;p__Firmicutes;c__Erysipelotrichia;o__Erysipelotrichales;f__Erysipelotrichaceae;g__Longicatena	0.00 ± 0.00	0.0	0.01 ± 0.01	0.0	1.08E-03	-	-	1.04E-03
k__Bacteria;p__Firmicutes;c__Erysipelotrichia;o__Erysipelotrichales;f__Erysipelotrichaceae;g__Massiliomicrobiota	0.00 ± 0.00	0.0	0.00 ± 0.00	0.0	8.69E-02	-	-	-
k__Bacteria;p__Firmicutes;c__Erysipelotrichia;o__Erysipelotrichales;f__Erysipelotrichaceae;g__Solobacterium	0.00 ± 0.00	0.0	0.00 ± 0.00	0.0	4.20E-02	2.1718	-	4.22E-02
k__Bacteria;p__Firmicutes;c__Erysipelotrichia;o__Erysipelotrichales;f__Erysipelotrichaceae;g__Turicibacter	0.00 ± 0.00	0.0	0.02 ± 0.01	0.0	7.34E-06	-	2.0343	6.75E-06
k__Bacteria;p__Firmicutes;c__Negativicutes;o__Acidaminococcales;f__Acidaminococcaceae;g__Acidaminococcus	0.00 ± 0.00	0.0	0.19 ± 0.06	5.7	1.86E-05	-	2.9723	1.72E-05
**k__Bacteria;p__Firmicutes;c__Negativicutes;o__Acidaminococcales;f__Acidaminococcaceae;g__Phascolarctobacterium**	**2.61 ± 0.23**	**73.0**	**1.03 ± 0.16**	**25.9**	**4.48E-18**	**3.8915**	**-**	**6.15E-18**

k__Bacteria;p__Firmicutes;c__Negativicutes;o__Acidaminococcales;f__Acidaminococcaceae;g__Succiniclasticum	0.00 ± 0.00	0.0	0.09 ± 0.07	1.3	8.69E-02	-	-	-
k__Bacteria;p__Firmicutes;c__Negativicutes;o__Selenomonadales;f__Selenomonadaceae;g__	0.06 ± 0.03	2.6	0.00 ± 0.00	0.0	6.50E-21	2.4926	-	8.38E-21
k__Bacteria;p__Firmicutes;c__Negativicutes;o__Selenomonadales;f__Selenomonadaceae;g__Megamonas	0.00 ± 0.00	0.0	0.04 ± 0.03	0.6	5.51E-02	-	-	-
k__Bacteria;p__Firmicutes;c__Negativicutes;o__Selenomonadales;f__Selenomonadaceae;g__Mitsuokella	0.01 ± 0.00	0.0	0.00 ± 0.00	0.0	5.17E-15	-	-	6.18E-15
k__Bacteria;p__Firmicutes;c__Negativicutes;o__Selenomonadales;f__Selenomonadaceae;g__Pectinatus	0.00 ± 0.00	0.0	0.00 ± 0.00	0.0	NA	-	-	-
**k__Bacteria;p__Firmicutes;c__Negativicutes;o__Selenomonadales;f__Selenomonadaceae;g__Propionispira**	**3.37 ± 0.35**	**70.4**	**0.00 ± 0.00**	**0.0**	**1.48E-53**	**4.2199**	**-**	**2.54E-53**
k__Bacteria;p__Firmicutes;c__Negativicutes;o__Selenomonadales;f__Selenomonadaceae;g__Schwartzia	0.00 ± 0.00	0.0	0.00 ± 0.00	0.0	NA	-	-	-
**k__Bacteria;p__Firmicutes;c__Negativicutes;o__Selenomonadales;f__Selenomonadaceae;g__Selenomonas**	**1.09 ± 0.17**	**27.0**	**0.00 ± 0.00**	**0.0**	**3.25E-52**	**3.7064**	**-**	**5.52E-52**
k__Bacteria;p__Firmicutes;c__Negativicutes;o__Veillonellales;f__Veillonellaceae;g__Allisonella	0.01 ± 0.00	0.0	0.00 ± 0.00	0.0	3.47E-04	-	-	3.68E-04
k__Bacteria;p__Firmicutes;c__Negativicutes;o__Veillonellales;f__Veillonellaceae;g__Anaeroglobus	0.00 ± 0.00	0.0	0.00 ± 0.00	0.0	2.44E-01	-	-	-
**k__Bacteria;p__Firmicutes;c__Negativicutes;o__Veillonellales;f__Veillonellaceae;g__Dialister**	**1.29 ± 0.16**	**33.0**	**1.19 ± 0.26**	**22.2**	**6.46E-09**	**3.3307**	**-**	**9.29E-09**
**k__Bacteria;p__Firmicutes;c__Negativicutes;o__Veillonellales;f__Veillonellaceae;g__Megasphaera**	**1.11 ± 0.46**	**20.0**	**0.01 ± 0.01**	**0.0**	**3.57E-38**	**3.7326**	**-**	**5.60E-38**
k__Bacteria;p__Firmicutes;c__Negativicutes;o__Veillonellales;f__Veillonellaceae;g__Veillonella	0.05 ± 0.01	0.9	0.05 ± 0.02	0.6	1.62E-09	2.1949	-	2.12E-09
k__Bacteria;p__Firmicutes;c__Tissierellia;o__Tissierellales;f__Peptoniphilaceae;g__	0.04 ± 0.01	0.9	0.00 ± 0.00	0.0	1.52E-15	2.2840	-	1.82E-15
k__Bacteria;p__Firmicutes;c__Tissierellia;o__Tissierellales;f__Peptoniphilaceae;g__Anaerococcus	0.02 ± 0.01	0.0	0.00 ± 0.00	0.0	4.77E-21	2.0561	-	6.06E-21
k__Bacteria;p__Firmicutes;c__Tissierellia;o__Tissierellales;f__Peptoniphilaceae;g__Anaerosphaera	0.00 ± 0.00	0.0	0.00 ± 0.00	0.0	NA	-	-	-
k__Bacteria;p__Firmicutes;c__Tissierellia;o__Tissierellales;f__Peptoniphilaceae;g__Finegoldia	0.01 ± 0.00	0.0	0.00 ± 0.00	0.0	1.17E-14	-	-	1.40E-14
k__Bacteria;p__Firmicutes;c__Tissierellia;o__Tissierellales;f__Peptoniphilaceae;g__Gallicola	0.00 ± 0.00	0.0	0.00 ± 0.00	0.0	NA	-	-	-
k__Bacteria;p__Firmicutes;c__Tissierellia;o__Tissierellales;f__Peptoniphilaceae;g__Helcococcus	0.00 ± 0.00	0.0	0.00 ± 0.00	0.0	8.36E-03	-	-	8.49E-03
k__Bacteria;p__Firmicutes;c__Tissierellia;o__Tissierellales;f__Peptoniphilaceae;g__Parvimonas	0.00 ± 0.00	0.0	0.00 ± 0.00	0.0	1.96E-04	-	-	2.05E-04
k__Bacteria;p__Firmicutes;c__Tissierellia;o__Tissierellales;f__Peptoniphilaceae;g__Peptoniphilus	0.06 ± 0.03	0.9	0.00 ± 0.00	0.0	3.49E-19	2.4429	-	4.39E-19
k__Bacteria;p__Firmicutes;c__Tissierellia;o__Tissierellales;f__Tissierellaceae;g__	0.00 ± 0.00	0.0	0.00 ± 0.00	0.0	3.21E-06	-	-	3.41E-06
k__Bacteria;p__Firmicutes;c__Tissierellia;o__Tissierellales;f__Tissierellaceae;g__Tissierella	0.00 ± 0.00	0.0	0.00 ± 0.00	0.0	NA	-	-	-
k__Bacteria;p__Fusobacteria;c__Fusobacteriia;o__Fusobacteriales;f__Fusobacteriaceae;g__Cetobacterium	0.09 ± 0.09	0.9	0.00 ± 0.00	0.0	9.78E-02	-	-	-
k__Bacteria;p__Fusobacteria;c__Fusobacteriia;o__Fusobacteriales;f__Fusobacteriaceae;g__Fusobacterium	0.13 ± 0.05	2.6	0.06 ± 0.04	1.9	8.56E-22	2.5793	-	1.14E-21
k__Bacteria;p__Fusobacteria;c__Fusobacteriia;o__Fusobacteriales;f__Leptotrichiaceae;g__Leptotrichia	0.00 ± 0.00	0.0	0.00 ± 0.00	0.0	1.73E-03	-	-	1.80E-03
k__Bacteria;p__Fusobacteria;c__Fusobacteriia;o__Fusobacteriales;f__Leptotrichiaceae;g__Sneathia	0.01 ± 0.01	0.0	0.00 ± 0.00	0.0	2.51E-08	-	-	2.73E-08
k__Bacteria;p__Fusobacteria;c__Fusobacteriia;o__Fusobacteriales;f__Leptotrichiaceae;g__Streptobacillus	0.00 ± 0.00	0.0	0.00 ± 0.00	0.0	4.20E-02	2.1120	-	4.22E-02
k__Bacteria;p__Gemmatimonadetes;c__;o__;f__;g__	0.00 ± 0.00	0.0	0.00 ± 0.00	0.0	NA	-	-	-
k__Bacteria;p__Gemmatimonadetes;c__Gemmatimonadetes;o__Gemmatimonadales;f__Gemmatimonadaceae;g__Gemmatirosa	0.00 ± 0.00	0.0	0.00 ± 0.00	0.0	NA	-	-	-
k__Bacteria;p__Gracilibacteria;c__;o__;f__;g__	0.00 ± 0.00	0.0	0.00 ± 0.00	0.0	NA	-	-	-
k__Bacteria;p__Lentisphaerae;c__Lentisphaeria;o__Victivallales;f__;g__	0.02 ± 0.01	0.0	0.00 ± 0.00	0.0	8.57E-18	-	-	1.08E-17
k__Bacteria;p__Lentisphaerae;c__Lentisphaeria;o__Victivallales;f__Victivallaceae;g__	0.01 ± 0.00	0.0	0.00 ± 0.00	0.0	1.80E-11	-	-	2.08E-11
k__Bacteria;p__Lentisphaerae;c__Lentisphaeria;o__Victivallales;f__Victivallaceae;g__Victivallis	0.00 ± 0.00	0.0	0.02 ± 0.01	0.6	1.46E-02	-	-	1.42E-02
k__Bacteria;p__Lentisphaerae;c__Oligosphaeria;o__Oligosphaerales;f__Oligosphaeraceae;g__	0.01 ± 0.00	0.0	0.00 ± 0.00	0.0	6.98E-11	-	-	7.87E-11
k__Bacteria;p__Planctomycetes;c__Planctomycetia;o__Planctomycetales;f__Gemmataceae;g__	0.00 ± 0.00	0.0	0.00 ± 0.00	0.0	2.44E-01	-	-	-
k__Bacteria;p__Planctomycetes;c__Planctomycetia;o__Planctomycetales;f__Isosphaeraceae;g__Aquisphaera	0.00 ± 0.00	0.0	0.00 ± 0.00	0.0	NA	-	-	-
k__Bacteria;p__Proteobacteria;c__Alphaproteobacteria;o__Caulobacterales;f__Caulobacteraceae;g__	0.00 ± 0.00	0.0	0.00 ± 0.00	0.0	NA	-	-	-
k__Bacteria;p__Proteobacteria;c__Alphaproteobacteria;o__Caulobacterales;f__Caulobacteraceae;g__Brevundimonas	0.00 ± 0.00	0.0	0.00 ± 0.00	0.0	4.20E-02	-	-	4.22E-02
k__Bacteria;p__Proteobacteria;c__Alphaproteobacteria;o__Caulobacterales;f__Caulobacteraceae;g__Caulobacter	0.00 ± 0.00	0.0	0.00 ± 0.00	0.0	NA	-	-	-
k__Bacteria;p__Proteobacteria;c__Alphaproteobacteria;o__Caulobacterales;f__Caulobacteraceae;g__Phenylobacterium	0.00 ± 0.00	0.0	0.00 ± 0.00	0.0	NA	-	-	-
k__Bacteria;p__Proteobacteria;c__Alphaproteobacteria;o__Rhizobiales;f__;g__	0.00 ± 0.00	0.0	0.00 ± 0.00	0.0	2.44E-01	-	-	-
k__Bacteria;p__Proteobacteria;c__Alphaproteobacteria;o__Rhizobiales;f__Aurantimonadaceae;g__Aureimonas	0.00 ± 0.00	0.0	0.00 ± 0.00	0.0	NA	-	-	-
k__Bacteria;p__Proteobacteria;c__Alphaproteobacteria;o__Rhizobiales;f__Beijerinckiaceae;g__Camelimonas	0.00 ± 0.00	0.0	0.00 ± 0.00	0.0	2.44E-01	-	-	-
k__Bacteria;p__Proteobacteria;c__Alphaproteobacteria;o__Rhizobiales;f__Bradyrhizobiaceae;g__Bosea	0.00 ± 0.00	0.0	0.00 ± 0.00	0.0	NA	-	-	-
k__Bacteria;p__Proteobacteria;c__Alphaproteobacteria;o__Rhizobiales;f__Bradyrhizobiaceae;g__Bradyrhizobium	0.00 ± 0.00	0.0	0.00 ± 0.00	0.0	9.78E-02	-	-	-
k__Bacteria;p__Proteobacteria;c__Alphaproteobacteria;o__Rhizobiales;f__Brucellaceae;g__Pseudochrobactrum	0.00 ± 0.00	0.0	0.00 ± 0.00	0.0	NA	-	-	-
k__Bacteria;p__Proteobacteria;c__Alphaproteobacteria;o__Rhizobiales;f__Hyphomicrobiaceae;g__Devosia	0.00 ± 0.00	0.0	0.00 ± 0.00	0.0	9.78E-02	-	-	-
k__Bacteria;p__Proteobacteria;c__Alphaproteobacteria;o__Rhizobiales;f__Hyphomicrobiaceae;g__Hyphomicrobium	0.00 ± 0.00	0.0	0.00 ± 0.00	0.0	NA	-	-	-
k__Bacteria;p__Proteobacteria;c__Alphaproteobacteria;o__Rhizobiales;f__Hyphomicrobiaceae;g__Rhodomicrobium	0.00 ± 0.00	0.0	0.00 ± 0.00	0.0	NA	-	-	-
k__Bacteria;p__Proteobacteria;c__Alphaproteobacteria;o__Rhizobiales;f__Methylobacteriaceae;g__Methylobacterium	0.00 ± 0.00	0.0	0.00 ± 0.00	0.0	1.86E-02	-	-	1.88E-02
k__Bacteria;p__Proteobacteria;c__Alphaproteobacteria;o__Rhizobiales;f__Methylocystaceae;g__Methylosinus	0.00 ± 0.00	0.0	0.00 ± 0.00	0.0	9.78E-02	-	-	-
k__Bacteria;p__Proteobacteria;c__Alphaproteobacteria;o__Rhizobiales;f__Phyllobacteriaceae;g__Mesorhizobium	0.00 ± 0.00	0.0	0.00 ± 0.00	0.0	NA	-	-	-
k__Bacteria;p__Proteobacteria;c__Alphaproteobacteria;o__Rhizobiales;f__Rhizobiaceae;g__	0.00 ± 0.00	0.0	0.00 ± 0.00	0.0	NA	-	-	-
k__Bacteria;p__Proteobacteria;c__Alphaproteobacteria;o__Rhizobiales;f__Rhizobiaceae;g__Rhizobium	0.00 ± 0.00	0.0	0.00 ± 0.00	0.0	NA	-	-	-
k__Bacteria;p__Proteobacteria;c__Alphaproteobacteria;o__Rhodobacterales;f__Hyphomonadaceae;g__	0.00 ± 0.00	0.0	0.00 ± 0.00	0.0	NA	-	-	-
k__Bacteria;p__Proteobacteria;c__Alphaproteobacteria;o__Rhodobacterales;f__Rhodobacteraceae;g__	0.00 ± 0.00	0.0	0.00 ± 0.00	0.0	NA	-	-	-
k__Bacteria;p__Proteobacteria;c__Alphaproteobacteria;o__Rhodobacterales;f__Rhodobacteraceae;g__Amaricoccus	0.00 ± 0.00	0.0	0.00 ± 0.00	0.0	2.44E-01	-	-	-
k__Bacteria;p__Proteobacteria;c__Alphaproteobacteria;o__Rhodobacterales;f__Rhodobacteraceae;g__Aquimixticola	0.00 ± 0.00	0.0	0.00 ± 0.00	0.0	4.20E-02	-	-	4.22E-02
k__Bacteria;p__Proteobacteria;c__Alphaproteobacteria;o__Rhodobacterales;f__Rhodobacteraceae;g__Gemmobacter	0.00 ± 0.00	0.0	0.00 ± 0.00	0.0	9.78E-02	-	-	-
k__Bacteria;p__Proteobacteria;c__Alphaproteobacteria;o__Rhodobacterales;f__Rhodobacteraceae;g__Paracoccus	0.00 ± 0.00	0.0	0.00 ± 0.00	0.0	4.20E-02	-	-	4.22E-02
k__Bacteria;p__Proteobacteria;c__Alphaproteobacteria;o__Rhodobacterales;f__Rhodobacteraceae;g__Rhodobacter	0.00 ± 0.00	0.0	0.00 ± 0.00	0.0	2.44E-01	-	-	-
k__Bacteria;p__Proteobacteria;c__Alphaproteobacteria;o__Rhodobacterales;f__Rhodobacteraceae;g__Rubellimicrobium	0.00 ± 0.00	0.0	0.00 ± 0.00	0.0	NA	-	-	-

k__Bacteria;p__Proteobacteria;c__Alphaproteobacteria;o__Rhodospirillales;f__Acetobacteraceae;g__	0.01 ± 0.00	0.0	0.00 ± 0.00	0.0	1.32E-20	-	-	1.67E-20
k__Bacteria;p__Proteobacteria;c__Alphaproteobacteria;o__Rhodospirillales;f__Acetobacteraceae;g__Acidocella	0.00 ± 0.00	0.0	0.00 ± 0.00	0.0	NA	-	-	-
k__Bacteria;p__Proteobacteria;c__Alphaproteobacteria;o__Rhodospirillales;f__Acetobacteraceae;g__Belnapia	0.00 ± 0.00	0.0	0.00 ± 0.00	0.0	NA	-	-	-
k__Bacteria;p__Proteobacteria;c__Alphaproteobacteria;o__Rhodospirillales;f__Acetobacteraceae;g__Gluconobacter	0.00 ± 0.00	0.0	0.00 ± 0.00	0.0	NA	-	-	-
k__Bacteria;p__Proteobacteria;c__Alphaproteobacteria;o__Rhodospirillales;f__Acetobacteraceae;g__Paracraurococcus	0.00 ± 0.00	0.0	0.00 ± 0.00	0.0	NA	-	-	-
k__Bacteria;p__Proteobacteria;c__Alphaproteobacteria;o__Rhodospirillales;f__Acetobacteraceae;g__Rhodovastum	0.00 ± 0.00	0.0	0.00 ± 0.00	0.0	NA	-	-	-
k__Bacteria;p__Proteobacteria;c__Alphaproteobacteria;o__Rhodospirillales;f__Acetobacteraceae;g__Roseomonas	0.00 ± 0.00	0.0	0.00 ± 0.00	0.0	NA	-	-	-
k__Bacteria;p__Proteobacteria;c__Alphaproteobacteria;o__Rhodospirillales;f__Rhodospirillaceae;g__	0.44 ± 0.08	12.2	0.43 ± 0.14	8.2	3.87E-15	2.9001	-	1.20E-15
k__Bacteria;p__Proteobacteria;c__Alphaproteobacteria;o__Rhodospirillales;f__Rhodospirillaceae;g__Ferrovibrio	0.00 ± 0.00	0.0	0.00 ± 0.00	0.0	NA	-	-	-
k__Bacteria;p__Proteobacteria;c__Alphaproteobacteria;o__Rhodospirillales;f__Rhodospirillaceae;g__Lacibacterium	0.00 ± 0.00	0.0	0.00 ± 0.00	0.0	NA	-	-	-
k__Bacteria;p__Proteobacteria;c__Alphaproteobacteria;o__Rhodospirillales;f__Rhodospirillaceae;g__Niveispirillum	0.00 ± 0.00	0.0	0.00 ± 0.00	0.0	2.44E-01	-	-	-
k__Bacteria;p__Proteobacteria;c__Alphaproteobacteria;o__Rhodospirillales;f__Rhodospirillaceae;g__Tistrella	0.00 ± 0.00	0.0	0.00 ± 0.00	0.0	NA	-	-	-
k__Bacteria;p__Proteobacteria;c__Alphaproteobacteria;o__Rickettsiales;f__;g__	0.03 ± 0.02	0.9	0.00 ± 0.00	0.0	2.51E-08	2.1876	-	2.73E-08
k__Bacteria;p__Proteobacteria;c__Alphaproteobacteria;o__Sphingomonadales;f__Erythrobacteraceae;g__Altererythrobacter	0.00 ± 0.00	0.0	0.00 ± 0.00	0.0	NA	-	-	-
k__Bacteria;p__Proteobacteria;c__Alphaproteobacteria;o__Sphingomonadales;f__Sphingomonadaceae;g__	0.00 ± 0.00	0.0	0.00 ± 0.00	0.0	2.44E-01	-	-	-
k__Bacteria;p__Proteobacteria;c__Alphaproteobacteria;o__Sphingomonadales;f__Sphingomonadaceae;g__Novosphingobium	0.00 ± 0.00	0.0	0.00 ± 0.00	0.0	2.44E-01	-	-	-
k__Bacteria;p__Proteobacteria;c__Alphaproteobacteria;o__Sphingomonadales;f__Sphingomonadaceae;g__Sphingobium	0.00 ± 0.00	0.0	0.00 ± 0.00	0.0	NA	-	-	-
k__Bacteria;p__Proteobacteria;c__Alphaproteobacteria;o__Sphingomonadales;f__Sphingomonadaceae;g__Sphingomonas	0.00 ± 0.00	0.0	0.00 ± 0.00	0.0	1.86E-02	2.1413	-	1.88E-02
k__Bacteria;p__Proteobacteria;c__Alphaproteobacteria;o__Sphingomonadales;f__Sphingomonadaceae;g__Sphingopyxis	0.00 ± 0.00	0.0	0.00 ± 0.00	0.0	NA	-	-	-
k__Bacteria;p__Proteobacteria;c__Alphaproteobacteria;o__Sphingomonadales;f__Sphingomonadaceae;g__Sphingorhabdus	0.00 ± 0.00	0.0	0.00 ± 0.00	0.0	NA	-	-	-
k__Bacteria;p__Proteobacteria;c__Betaproteobacteria;o__;f__;g__	0.00 ± 0.00	0.0	0.00 ± 0.00	0.0	NA	-	-	-
k__Bacteria;p__Proteobacteria;c__Betaproteobacteria;o__Burkholderiales;f__Alcaligenaceae;g__Achromobacter	0.00 ± 0.00	0.0	0.00 ± 0.00	0.0	9.78E-02	-	-	-
k__Bacteria;p__Proteobacteria;c__Betaproteobacteria;o__Burkholderiales;f__Alcaligenaceae;g__Alcaligenes	0.00 ± 0.00	0.0	0.00 ± 0.00	0.0	NA	-	-	-
k__Bacteria;p__Proteobacteria;c__Betaproteobacteria;o__Burkholderiales;f__Alcaligenaceae;g__Derxia	0.00 ± 0.00	0.0	0.00 ± 0.00	0.0	NA	-	-	-
k__Bacteria;p__Proteobacteria;c__Betaproteobacteria;o__Burkholderiales;f__Alcaligenaceae;g__Parapusillimonas	0.00 ± 0.00	0.0	0.00 ± 0.00	0.0	NA	-	-	-
k__Bacteria;p__Proteobacteria;c__Betaproteobacteria;o__Burkholderiales;f__Alcaligenaceae;g__Pelistega	0.00 ± 0.00	0.0	0.00 ± 0.00	0.0	2.44E-01	-	-	-
k__Bacteria;p__Proteobacteria;c__Betaproteobacteria;o__Burkholderiales;f__Burkholderiaceae;g__Burkholderia	0.00 ± 0.00	0.0	0.00 ± 0.00	0.0	NA	-	-	-
k__Bacteria;p__Proteobacteria;c__Betaproteobacteria;o__Burkholderiales;f__Burkholderiaceae;g__Cupriavidus	0.00 ± 0.00	0.0	0.00 ± 0.00	0.0	NA	-	-	-
k__Bacteria;p__Proteobacteria;c__Betaproteobacteria;o__Burkholderiales;f__Burkholderiaceae;g__Lautropia	0.00 ± 0.00	0.0	0.00 ± 0.00	0.0	2.29E-01	-	-	-
k__Bacteria;p__Proteobacteria;c__Betaproteobacteria;o__Burkholderiales;f__Burkholderiaceae;g__Limnobacter	0.00 ± 0.00	0.0	0.00 ± 0.00	0.0	2.44E-01	-	-	-
k__Bacteria;p__Proteobacteria;c__Betaproteobacteria;o__Burkholderiales;f__Burkholderiaceae;g__Paraburkholderia	0.01 ± 0.00	0.0	0.00 ± 0.00	0.0	2.08E-09	-	-	2.30E-09
k__Bacteria;p__Proteobacteria;c__Betaproteobacteria;o__Burkholderiales;f__Burkholderiaceae;g__Ralstonia	0.00 ± 0.00	0.0	0.00 ± 0.00	0.0	4.20E-02	2.2195	-	4.22E-02
k__Bacteria;p__Proteobacteria;c__Betaproteobacteria;o__Burkholderiales;f__Comamonadaceae;g__	0.08 ± 0.01	0.0	0.05 ± 0.03	1.9	6.96E-32	2.2880	-	1.12E-31
k__Bacteria;p__Proteobacteria;c__Betaproteobacteria;o__Burkholderiales;f__Comamonadaceae;g__Comamonas	0.00 ± 0.00	0.0	0.00 ± 0.00	0.0	1.86E-02	-	-	1.88E-02
k__Bacteria;p__Proteobacteria;c__Betaproteobacteria;o__Burkholderiales;f__Comamonadaceae;g__Delftia	0.00 ± 0.00	0.0	0.00 ± 0.00	0.0	NA	-	-	-
k__Bacteria;p__Proteobacteria;c__Betaproteobacteria;o__Burkholderiales;f__Comamonadaceae;g__Pelomonas	0.00 ± 0.00	0.0	0.00 ± 0.00	0.0	1.86E-02	-	-	1.88E-02
k__Bacteria;p__Proteobacteria;c__Betaproteobacteria;o__Burkholderiales;f__Oxalobacteraceae;g__Duganella	0.00 ± 0.00	0.0	0.00 ± 0.00	0.0	NA	-	-	-
k__Bacteria;p__Proteobacteria;c__Betaproteobacteria;o__Burkholderiales;f__Oxalobacteraceae;g__Herbaspirillum	0.03 ± 0.00	0.0	0.00 ± 0.00	0.0	4.05E-32	2.1879	-	5.78E-32
k__Bacteria;p__Proteobacteria;c__Betaproteobacteria;o__Burkholderiales;f__Oxalobacteraceae;g__Janthinobacterium	0.00 ± 0.00	0.0	0.00 ± 0.00	0.0	2.44E-01	-	-	-
k__Bacteria;p__Proteobacteria;c__Betaproteobacteria;o__Burkholderiales;f__Oxalobacteraceae;g__Massilia	0.00 ± 0.00	0.0	0.00 ± 0.00	0.0	NA	-	-	-
k__Bacteria;p__Proteobacteria;c__Betaproteobacteria;o__Burkholderiales;f__Roseateles;g__	0.26 ± 0.07	6.1	0.04 ± 0.03	1.3	1.08E-37	2.9980	-	1.69E-37
k__Bacteria;p__Proteobacteria;c__Betaproteobacteria;o__Burkholderiales;f__Roseateles;g__aquatilis	0.00 ± 0.00	0.0	0.00 ± 0.00	0.0	NA	-	-	-
k__Bacteria;p__Proteobacteria;c__Betaproteobacteria;o__Burkholderiales;f__Rubrivivax;g__gelatinosus	0.00 ± 0.00	0.0	0.00 ± 0.00	0.0	4.20E-02	2.3359	-	4.22E-02
k__Bacteria;p__Proteobacteria;c__Betaproteobacteria;o__Burkholderiales;f__Sutterellaceae;g__Parasutterella	0.00 ± 0.00	0.0	0.83 ± 0.14	21.5	4.62E-20	-	3.6246	3.31E-20
**k__Bacteria;p__Proteobacteria;c__Betaproteobacteria;o__Burkholderiales;f__Sutterellaceae;g__Sutterella**	**0.29 ± 0.04**	**9.6**	**1.09 ± 0.20**	**23.4**	**2.21E-02**	**-**	**3.6299**	**1.66E-02**
k__Bacteria;p__Proteobacteria;c__Betaproteobacteria;o__Burkholderiales;f__Tepidimonas;g__fonticaldi	0.00 ± 0.00	0.0	0.00 ± 0.00	0.0	NA	-	-	-
k__Bacteria;p__Proteobacteria;c__Betaproteobacteria;o__Hydrogenophilales;f__Hydrogenophilaceae;g__	0.00 ± 0.00	0.0	0.00 ± 0.00	0.0	NA	-	-	-
k__Bacteria;p__Proteobacteria;c__Betaproteobacteria;o__Methylophilales;f__Methylophilaceae;g__	0.00 ± 0.00	0.0	0.00 ± 0.00	0.0	NA	-	-	-
k__Bacteria;p__Proteobacteria;c__Betaproteobacteria;o__Neisseriales;f__Neisseriaceae;g__	0.00 ± 0.00	0.0	0.00 ± 0.00	0.0	8.36E-03	-	-	8.49E-03
k__Bacteria;p__Proteobacteria;c__Betaproteobacteria;o__Neisseriales;f__Neisseriaceae;g__Alysiella	0.00 ± 0.00	0.0	0.00 ± 0.00	0.0	NA	-	-	-
k__Bacteria;p__Proteobacteria;c__Betaproteobacteria;o__Neisseriales;f__Neisseriaceae;g__Eikenella	0.00 ± 0.00	0.0	0.00 ± 0.00	0.0	NA	-	-	-
k__Bacteria;p__Proteobacteria;c__Betaproteobacteria;o__Neisseriales;f__Neisseriaceae;g__Kingella	0.00 ± 0.00	0.0	0.00 ± 0.00	0.0	NA	-	-	-
k__Bacteria;p__Proteobacteria;c__Betaproteobacteria;o__Neisseriales;f__Neisseriaceae;g__Neisseria	0.00 ± 0.00	0.0	0.00 ± 0.00	0.0	8.29E-01	-	-	-
k__Bacteria;p__Proteobacteria;c__Betaproteobacteria;o__Neisseriales;f__Neisseriaceae;g__Simonsiella	0.00 ± 0.00	0.0	0.00 ± 0.00	0.0	NA	-	-	-
k__Bacteria;p__Proteobacteria;c__Betaproteobacteria;o__Neisseriales;f__Neisseriaceae;g__Snodgrassella	0.00 ± 0.00	0.0	0.00 ± 0.00	0.0	NA	-	-	-
k__Bacteria;p__Proteobacteria;c__Betaproteobacteria;o__Rhodocyclales;f__Rhodocyclaceae;g__Azoarcus	0.00 ± 0.00	0.0	0.00 ± 0.00	0.0	1.86E-02	-	-	1.88E-02
k__Bacteria;p__Proteobacteria;c__Betaproteobacteria;o__Rhodocyclales;f__Rhodocyclaceae;g__Propionivibrio	0.00 ± 0.00	0.0	0.00 ± 0.00	0.0	2.44E-01	-	-	-
k__Bacteria;p__Proteobacteria;c__Deltaproteobacteria;o__Bradymonadales;f__;g__	0.11 ± 0.02	1.7	0.00 ± 0.00	0.0	4.42E-27	2.7477	-	5.99E-27
k__Bacteria;p__Proteobacteria;c__Deltaproteobacteria;o__Desulfobacterales;f__Desulfobacteraceae;g__	0.00 ± 0.00	0.0	0.00 ± 0.00	0.0	NA	-	-	-
k__Bacteria;p__Proteobacteria;c__Deltaproteobacteria;o__Desulfobacterales;f__Desulfobulbaceae;g__Desulfobulbus	0.00 ± 0.00	0.0	0.00 ± 0.00	0.0	NA	-	-	-
k__Bacteria;p__Proteobacteria;c__Deltaproteobacteria;o__Desulfovibrionales;f__;g__	0.04 ± 0.02	0.9	0.00 ± 0.00	0.0	1.52E-15	2.2975	-	1.82E-15
k__Bacteria;p__Proteobacteria;c__Deltaproteobacteria;o__Desulfovibrionales;f__Desulfohalobiaceae;g__	0.01 ± 0.00	0.0	0.00 ± 0.00	0.0	6.48E-07	-	-	6.94E-07
k__Bacteria;p__Proteobacteria;c__Deltaproteobacteria;o__Desulfovibrionales;f__Desulfomicrobiaceae;g__Desulfomicrobium	0.00 ± 0.00	0.0	0.00 ± 0.00	0.0	NA	-	-	-

k__Bacteria;p__Proteobacteria;c__Deltaproteobacteria;o__Desulfovibrionales;f__Desulfovibrionaceae;g__	0.01 ± 0.01	0.0	0.00 ± 0.00	0.0	1.08E-24	-	-	1.42E-24
k__Bacteria;p__Proteobacteria;c__Deltaproteobacteria;o__Desulfovibrionales;f__Desulfovibrionaceae;g__Desulfovibrio	0.43 ± 0.07	10.4	0.02 ± 0.01	0.0	1.34E-39	3.3142	-	2.15E-39
k__Bacteria;p__Proteobacteria;c__Deltaproteobacteria;o__Desulfuromonadales;f__;g__	0.00 ± 0.00	0.0	0.00 ± 0.00	0.0	2.44E-01	-	-	-
k__Bacteria;p__Proteobacteria;c__Deltaproteobacteria;o__Desulfuromonadales;f__Desulfuromonadaceae;g__	0.02 ± 0.01	0.0	0.00 ± 0.00	0.0	1.64E-10	-	-	1.84E-10
k__Bacteria;p__Proteobacteria;c__Deltaproteobacteria;o__Desulfuromonadales;f__Geobacteraceae;g__Geobacter	0.00 ± 0.00	0.0	0.00 ± 0.00	0.0	9.78E-02	-	-	-
k__Bacteria;p__Proteobacteria;c__Deltaproteobacteria;o__Myxococcales;f__Labilitrichaceae;g__Labilithrix	0.00 ± 0.00	0.0	0.00 ± 0.00	0.0	NA	-	-	-
k__Bacteria;p__Proteobacteria;c__Deltaproteobacteria;o__Myxococcales;f__Phaselicystidaceae;g__Phaselicystis	0.00 ± 0.00	0.0	0.00 ± 0.00	0.0	NA	-	-	-
k__Bacteria;p__Proteobacteria;c__Epsilonproteobacteria;o__Campylobacterales;f__Campylobacteraceae;g__	0.00 ± 0.00	0.0	0.00 ± 0.00	0.0	NA	-	-	-
k__Bacteria;p__Proteobacteria;c__Epsilonproteobacteria;o__Campylobacterales;f__Campylobacteraceae;g__Arcobacter	0.00 ± 0.00	0.0	0.00 ± 0.00	0.0	NA	-	-	-
k__Bacteria;p__Proteobacteria;c__Epsilonproteobacteria;o__Campylobacterales;f__Campylobacteraceae;g__Campylobacter	0.04 ± 0.02	0.9	0.00 ± 0.00	0.0	7.43E-23	2.3600	-	9.70E-23
k__Bacteria;p__Proteobacteria;c__Epsilonproteobacteria;o__Campylobacterales;f__Campylobacteraceae;g__Sulfurospirillum	0.00 ± 0.00	0.0	0.00 ± 0.00	0.0	2.44E-01	-	-	-
k__Bacteria;p__Proteobacteria;c__Epsilonproteobacteria;o__Campylobacterales;f__Helicobacteraceae;g__	0.00 ± 0.00	0.0	0.00 ± 0.00	0.0	2.44E-01	-	-	-
**k__Bacteria;p__Proteobacteria;c__Epsilonproteobacteria;o__Campylobacterales;f__Helicobacteraceae;g__Helicobacter**	**30.74 ± 2.90**	**96.5**	**0.00 ± 0.00**	**0.0**	**1.33E-55**	**5.1906**	**-**	**2.31E-55**
k__Bacteria;p__Proteobacteria;c__Epsilonproteobacteria;o__Campylobacterales;f__Helicobacteraceae;g__Wolinella	0.00 ± 0.00	0.0	0.00 ± 0.00	0.0	NA	-	-	-
k__Bacteria;p__Proteobacteria;c__Gammaproteobacteria;o__;f__;g__	0.22 ± 0.05	4.3	0.00 ± 0.00	0.0	6.06E-49	3.0363	-	1.00E-48
k__Bacteria;p__Proteobacteria;c__Gammaproteobacteria;o__Aeromonadales;f__Aeromonadaceae;g__Aeromonas	0.00 ± 0.00	0.0	0.00 ± 0.00	0.0	2.44E-01	-	-	-
**k__Bacteria;p__Proteobacteria;c__Gammaproteobacteria;o__Aeromonadales;f__Succinivibrionaceae;g__Succinivibrio**	**1.60 ± 0.17**	**47.8**	**0.03 ± 0.03**	**0.6**	**8.84E-53**	**3.8896**	**-**	**1.53E-52**
k__Bacteria;p__Proteobacteria;c__Gammaproteobacteria;o__Alteromonadales;f__Alteromonadaceae;g__Marinobacter	0.00 ± 0.00	0.0	0.00 ± 0.00	0.0	2.44E-01	-	-	-
k__Bacteria;p__Proteobacteria;c__Gammaproteobacteria;o__Alteromonadales;f__Idiomarinaceae;g__Aliidiomarina	0.00 ± 0.00	0.0	0.00 ± 0.00	0.0	1.73E-03	-	-	1.77E-03
k__Bacteria;p__Proteobacteria;c__Gammaproteobacteria;o__Cardiobacteriales;f__Cardiobacteriaceae;g__Cardiobacterium	0.00 ± 0.00	0.0	0.00 ± 0.00	0.0	2.44E-01	-	-	-
k__Bacteria;p__Proteobacteria;c__Gammaproteobacteria;o__Cardiobacteriales;f__Cardiobacteriaceae;g__Suttonella	0.00 ± 0.00	0.0	0.00 ± 0.00	0.0	NA	-	-	-
k__Bacteria;p__Proteobacteria;c__Gammaproteobacteria;o__Chromatiales;f__Chromatiaceae;g__	0.00 ± 0.00	0.0	0.00 ± 0.00	0.0	NA	-	-	-
k__Bacteria;p__Proteobacteria;c__Gammaproteobacteria;o__Enterobacterales;f__Enterobacteriaceae;g__	0.10 ± 0.06	1.7	0.12 ± 0.06	1.9	4.12E-03	-	2.6402	2.96E-03
k__Bacteria;p__Proteobacteria;c__Gammaproteobacteria;o__Enterobacterales;f__Enterobacteriaceae;g__Enterobacter	0.04 ± 0.03	0.9	0.01 ± 0.01	0.0	3.15E-02	2.1794	-	3.28E-02
k__Bacteria;p__Proteobacteria;c__Gammaproteobacteria;o__Enterobacterales;f__Enterobacteriaceae;g__Kosakonia	0.00 ± 0.00	0.0	0.00 ± 0.00	0.0	2.44E-01	-	-	-
k__Bacteria;p__Proteobacteria;c__Gammaproteobacteria;o__Enterobacterales;f__Erwiniaceae;g__Buchnera	0.00 ± 0.00	0.0	0.00 ± 0.00	0.0	NA	-	-	-
k__Bacteria;p__Proteobacteria;c__Gammaproteobacteria;o__Enterobacterales;f__Erwiniaceae;g__Pantoea	0.00 ± 0.00	0.0	0.00 ± 0.00	0.0	1.86E-02	-	-	1.88E-02
k__Bacteria;p__Proteobacteria;c__Gammaproteobacteria;o__Enterobacterales;f__Morganellaceae;g__	0.00 ± 0.00	0.0	0.00 ± 0.00	0.0	NA	-	-	-
k__Bacteria;p__Proteobacteria;c__Gammaproteobacteria;o__Enterobacterales;f__Morganellaceae;g__Providencia	0.00 ± 0.00	0.0	0.00 ± 0.00	0.0	2.44E-01	-	-	-
k__Bacteria;p__Proteobacteria;c__Gammaproteobacteria;o__Enterobacterales;f__Pectobacteriaceae;g__	0.00 ± 0.00	0.0	0.00 ± 0.00	0.0	NA	-	-	-
k__Bacteria;p__Proteobacteria;c__Gammaproteobacteria;o__Enterobacterales;f__Yersiniaceae;g__Ewingella	0.00 ± 0.00	0.0	0.00 ± 0.00	0.0	NA	-	-	-
k__Bacteria;p__Proteobacteria;c__Gammaproteobacteria;o__Legionellales;f__Coxiellaceae;g__Diplorickettsia	0.00 ± 0.00	0.0	0.00 ± 0.00	0.0	2.44E-01	-	-	-
k__Bacteria;p__Proteobacteria;c__Gammaproteobacteria;o__Methylococcales;f__Methylococcaceae;g__	0.00 ± 0.00	0.0	0.00 ± 0.00	0.0	NA	-	-	-
k__Bacteria;p__Proteobacteria;c__Gammaproteobacteria;o__Oceanospirillales;f__Halomonadaceae;g__Chromohalobacter	0.00 ± 0.00	0.0	0.00 ± 0.00	0.0	NA	-	-	-
k__Bacteria;p__Proteobacteria;c__Gammaproteobacteria;o__Oceanospirillales;f__Halomonadaceae;g__Halomonas	0.43 ± 0.08	7.8	0.00 ± 0.00	0.0	1.48E-53	3.3510	-	2.54E-53
k__Bacteria;p__Proteobacteria;c__Gammaproteobacteria;o__Oceanospirillales;f__Oceanospirillaceae;g__Marinobacterium	0.00 ± 0.00	0.0	0.00 ± 0.00	0.0	NA	-	-	-
k__Bacteria;p__Proteobacteria;c__Gammaproteobacteria;o__Orbales;f__Orbaceae;g__	0.00 ± 0.00	0.0	0.00 ± 0.00	0.0	NA	-	-	-
k__Bacteria;p__Proteobacteria;c__Gammaproteobacteria;o__Pasteurellales;f__Pasteurellaceae;g__	0.00 ± 0.00	0.0	0.00 ± 0.00	0.0	3.80E-03	-	-	3.87E-03
k__Bacteria;p__Proteobacteria;c__Gammaproteobacteria;o__Pasteurellales;f__Pasteurellaceae;g__Aggregatibacter	0.01 ± 0.00	0.0	0.00 ± 0.00	0.0	4.03E-17	-	-	4.94E-17
k__Bacteria;p__Proteobacteria;c__Gammaproteobacteria;o__Pasteurellales;f__Pasteurellaceae;g__Haemophilus	0.58 ± 0.11	15.7	0.09 ± 0.03	1.9	1.91E-23	3.4173	-	2.98E-23
k__Bacteria;p__Proteobacteria;c__Gammaproteobacteria;o__Pasteurellales;f__Pasteurellaceae;g__Mannheimia	0.00 ± 0.00	0.0	0.00 ± 0.00	0.0	NA	-	-	-
k__Bacteria;p__Proteobacteria;c__Gammaproteobacteria;o__Pseudomonadales;f__Moraxellaceae;g__Acinetobacter	0.01 ± 0.00	0.0	0.00 ± 0.00	0.0	6.48E-07	-	-	6.94E-07
k__Bacteria;p__Proteobacteria;c__Gammaproteobacteria;o__Pseudomonadales;f__Moraxellaceae;g__Moraxella	0.00 ± 0.00	0.0	0.00 ± 0.00	0.0	1.86E-02	2.5591	-	1.88E-02
k__Bacteria;p__Proteobacteria;c__Gammaproteobacteria;o__Pseudomonadales;f__Moraxellaceae;g__Psychrobacter	0.00 ± 0.00	0.0	0.00 ± 0.00	0.0	9.78E-02	-	-	-
k__Bacteria;p__Proteobacteria;c__Gammaproteobacteria;o__Pseudomonadales;f__Pseudomonadaceae;g__Pseudomonas	0.00 ± 0.00	0.0	0.00 ± 0.00	0.0	5.69E-08	-	-	6.18E-08
k__Bacteria;p__Proteobacteria;c__Gammaproteobacteria;o__Thiotrichales;f__Francisellaceae;g__Francisella	0.00 ± 0.00	0.0	0.00 ± 0.00	0.0	NA	-	-	-
k__Bacteria;p__Proteobacteria;c__Gammaproteobacteria;o__Xanthomonadales;f__;g__	0.00 ± 0.00	0.0	0.00 ± 0.00	0.0	NA	-	-	-
k__Bacteria;p__Proteobacteria;c__Gammaproteobacteria;o__Xanthomonadales;f__Rhodanobacteraceae;g__Dokdonella	0.00 ± 0.00	0.0	0.00 ± 0.00	0.0	NA	-	-	-
k__Bacteria;p__Proteobacteria;c__Gammaproteobacteria;o__Xanthomonadales;f__Rhodanobacteraceae;g__Rhodanobacter	0.00 ± 0.00	0.0	0.00 ± 0.00	0.0	NA	-	-	-
k__Bacteria;p__Proteobacteria;c__Gammaproteobacteria;o__Xanthomonadales;f__Xanthomonadaceae;g__	0.00 ± 0.00	0.0	0.00 ± 0.00	0.0	9.78E-02	-	-	-
k__Bacteria;p__Proteobacteria;c__Gammaproteobacteria;o__Xanthomonadales;f__Xanthomonadaceae;g__Luteimonas	0.00 ± 0.00	0.0	0.00 ± 0.00	0.0	2.44E-01	-	-	-
k__Bacteria;p__Proteobacteria;c__Gammaproteobacteria;o__Xanthomonadales;f__Xanthomonadaceae;g__Lysobacter	0.00 ± 0.00	0.0	0.00 ± 0.00	0.0	2.44E-01	-	-	-
k__Bacteria;p__Proteobacteria;c__Gammaproteobacteria;o__Xanthomonadales;f__Xanthomonadaceae;g__Pseudoxanthomonas	0.00 ± 0.00	0.0	0.00 ± 0.00	0.0	NA	-	-	-
k__Bacteria;p__Proteobacteria;c__Gammaproteobacteria;o__Xanthomonadales;f__Xanthomonadaceae;g__Stenotrophomonas	0.00 ± 0.00	0.0	0.00 ± 0.00	0.0	1.86E-02	-	-	1.88E-02
k__Bacteria;p__Proteobacteria;c__Gammaproteobacteria;o__Xanthomonadales;f__Xanthomonadaceae;g__Xanthomonas	0.00 ± 0.00	0.0	0.00 ± 0.00	0.0	NA	-	-	-
k__Bacteria;p__Saccharibacteria;c__;o__;f__;g__	0.00 ± 0.00	0.0	0.00 ± 0.00	0.0	NA	-	-	-
k__Bacteria;p__Spirochaetes;c__Spirochaetia;o__Brachyspirales;f__Brachyspiraceae;g__Brachyspira	0.37 ± 0.05	13.9	0.00 ± 0.00	0.0	6.06E-49	3.2586	-	1.00E-48
k__Bacteria;p__Spirochaetes;c__Spirochaetia;o__Spirochaetales;f__Spirochaetaceae;g__	0.09 ± 0.02	1.7	0.00 ± 0.00	0.0	2.16E-22	2.6988	-	2.79E-22
k__Bacteria;p__Spirochaetes;c__Spirochaetia;o__Spirochaetales;f__Spirochaetaceae;g__Treponema	0.30 ± 0.06	7.0	0.00 ± 0.00	0.0	3.60E-45	3.1776	-	5.78E-45
k__Bacteria;p__Synergistetes;c__Synergistia;o__Synergistales;f__Synergistaceae;g__	0.00 ± 0.00	0.0	0.00 ± 0.00	0.0	NA	-	-	-
k__Bacteria;p__Synergistetes;c__Synergistia;o__Synergistales;f__Synergistaceae;g__Fretibacterium	0.00 ± 0.00	0.0	0.00 ± 0.00	0.0	NA	-	-	-
k__Bacteria;p__Synergistetes;c__Synergistia;o__Synergistales;f__Synergistaceae;g__Jonquetella	0.00 ± 0.00	0.0	0.00 ± 0.00	0.0	NA	-	-	-
k__Bacteria;p__Synergistetes;c__Synergistia;o__Synergistales;f__Synergistaceae;g__Pyramidobacter	0.00 ± 0.00	0.0	0.00 ± 0.00	0.0	NA	-	-	-

k__Bacteria;p__Tenericutes;c__Mollicutes;o__Acholeplasmatales;f__Acholeplasmataceae;g__	0.02 ± 0.00	0.0	0.03 ± 0.01	0.0	4.48E-20	-	-	6.26E-20
k__Bacteria;p__Tenericutes;c__Mollicutes;o__Anaeroplasmatales;f__Anaeroplasmataceae;g__	0.25 ± 0.05	8.7	0.02 ± 0.01	1.3	2.01E-25	3.1046	-	2.73E-25
k__Bacteria;p__Tenericutes;c__Mollicutes;o__Anaeroplasmatales;f__Anaeroplasmataceae;g__Anaeroplasma	0.14 ± 0.07	3.5	0.00 ± 0.00	0.0	5.98E-16	2.8268	-	7.17E-16
k__Bacteria;p__Tenericutes;c__Mollicutes;o__Anaeroplasmatales;f__Anaeroplasmataceae;g__Asteroleplasma	0.00 ± 0.00	0.0	0.02 ± 0.02	0.6	8.29E-01	-	-	-
k__Bacteria;p__Tenericutes;c__Mollicutes;o__Entomoplasmatales;f__Entomoplasmataceae;g__	0.00 ± 0.00	0.0	0.00 ± 0.00	0.0	2.44E-01	-	-	-
k__Bacteria;p__Tenericutes;c__Mollicutes;o__Entomoplasmatales;f__Entomoplasmataceae;g__Entomoplasma	0.00 ± 0.00	0.0	0.00 ± 0.00	0.0	NA	-	-	-
k__Bacteria;p__Tenericutes;c__Mollicutes;o__Entomoplasmatales;f__Spiroplasmataceae;g__	0.03 ± 0.01	0.0	0.26 ± 0.07	8.2	1.68E-05	-	3.0779	2.04E-05
k__Bacteria;p__Tenericutes;c__Mollicutes;o__Mollicutes_RF9;f__;g__	0.08 ± 0.02	0.9	0.16 ± 0.07	3.2	3.99E-16	-	2.6269	1.10E-16
k__Bacteria;p__Tenericutes;c__Mollicutes;o__Mycoplasmatales;f__Mycoplasmataceae;g__	0.02 ± 0.01	0.0	0.00 ± 0.00	0.0	2.89E-07	-	-	3.11E-07
k__Bacteria;p__Tenericutes;c__Mollicutes;o__Mycoplasmatales;f__Mycoplasmataceae;g__Mycoplasma	0.00 ± 0.00	0.0	0.00 ± 0.00	0.0	1.86E-02	-	-	1.88E-02
k__Bacteria;p__Tenericutes;c__Mollicutes;o__Mycoplasmatales;f__Mycoplasmataceae;g__Ureaplasma	0.00 ± 0.00	0.0	0.00 ± 0.00	0.0	NA	-	-	-
k__Bacteria;p__Tenericutes;c__Mollicutes;o__NB1-n;f__;g__	0.00 ± 0.00	0.0	0.13 ± 0.06	3.8	1.89E-04	-	2.8540	1.78E-04
k__Bacteria;p__Verrucomicrobia;c__;o__;f__;g__	0.15 ± 0.03	4.3	0.00 ± 0.00	0.0	9.62E-43	2.8832	-	1.51E-42
k__Bacteria;p__Verrucomicrobia;c__Opitutae;o__Opitutae_vadinHA64;f__;g__	0.00 ± 0.00	0.0	0.00 ± 0.00	0.0	4.30E-04	-	-	4.47E-04
k__Bacteria;p__Verrucomicrobia;c__Opitutae;o__Puniceicoccales;f__Puniceicoccaceae;g__	0.04 ± 0.01	0.9	0.01 ± 0.01	0.0	3.08E-18	2.1690	-	3.94E-18
k__Bacteria;p__Verrucomicrobia;c__Opitutae;o__Puniceicoccales;f__Puniceicoccaceae;g__Cerasicoccus	0.00 ± 0.00	0.0	0.00 ± 0.00	0.0	7.11E-06	-	-	7.51E-06
k__Bacteria;p__Verrucomicrobia;c__Spartobacteria;o__Chthoniobacterales;f__Chthoniobacteraceae;g__Chthoniobacter	0.00 ± 0.00	0.0	0.00 ± 0.00	0.0	NA	-	-	-
k__Bacteria;p__Verrucomicrobia;c__Verrucomicrobiae;o__Verrucomicrobiales;f__Akkermansiaceae;g__Akkermansia	0.00 ± 0.00	0.0	0.36 ± 0.12	8.9	6.28E-11	-	3.2857	5.34E-11

$ mean ± s.e.m

* Individual samples with >1% abundance were counted.

Table S6. Relative abundance of bacterial taxa (L6, at the genus and above levels) at the vaginal site of macaques and humans.

Vaginal site	Macaque		Human			LDA score		
	Abundance[$] (%)	Prevalence* (%)	Abundance[$] (%)	Prevalence* (%)	MW test (p)	Macaque	Human	p value
k__Bacteria;p__Acidobacteria;c__Acidobacteriia;o__Acidobacteriales;f__Acidobacteriaceae;g__	0.00 ± 0.00	0.0	0.00 ± 0.00	0.0	3.55E-01	-	-	-
k__Bacteria;p__Acidobacteria;c__Acidobacteriia;o__Acidobacteriales;f__Acidobacteriaceae;g__Terriglobus	0.00 ± 0.00	0.0	0.00 ± 0.00	0.0	NA	-	-	-
k__Bacteria;p__Acidobacteria;c__Holophagae;o__;f__;g__	0.00 ± 0.00	0.0	0.00 ± 0.00	0.0	3.55E-01	-	-	-
k__Bacteria;p__Acidobacteria;c__Solibacteres;o__Solibacterales;f__Bryobacteraceae;g__Paludibaculum	0.00 ± 0.00	0.0	0.00 ± 0.00	0.0	NA	-	-	-
k__Bacteria;p__Actinobacteria;c__Actinobacteria;o__Actinomycetales;f__Actinomycetaceae;g__	0.05 ± 0.02	1.2	0.00 ± 0.00	0.0	1.59E-08	2.6319	-	1.92E-08
k__Bacteria;p__Actinobacteria;c__Actinobacteria;o__Actinomycetales;f__Actinomycetaceae;g__Actinobaculum	0.00 ± 0.00	0.0	0.00 ± 0.00	0.0	NA	-	-	-
k__Bacteria;p__Actinobacteria;c__Actinobacteria;o__Actinomycetales;f__Actinomycetaceae;g__Actinomyces	0.01 ± 0.01	0.0	0.04 ± 0.02	0.0	2.18E-02	-	2.6089	1.95E-02
k__Bacteria;p__Actinobacteria;c__Actinobacteria;o__Actinomycetales;f__Actinomycetaceae;g__Actinotignum	0.00 ± 0.00	0.0	0.00 ± 0.00	0.0	NA	-	-	-
k__Bacteria;p__Actinobacteria;c__Actinobacteria;o__Actinomycetales;f__Actinomycetaceae;g__Arcanobacterium	0.04 ± 0.01	0.0	0.01 ± 0.00	0.0	6.99E-11	2.5420	-	9.46E-11
k__Bacteria;p__Actinobacteria;c__Actinobacteria;o__Actinomycetales;f__Actinomycetaceae;g__Flaviflexus	0.00 ± 0.00	0.0	0.00 ± 0.00	0.0	1.04E-01	-	-	-
**k__Bacteria;p__Actinobacteria;c__Actinobacteria;o__Actinomycetales;f__Actinomycetaceae;g__Mobiluncus**	**1.69 ± 0.22**	**57.8**	**0.09 ± 0.05**	**4.1**	**5.60E-15**	**3.8735**	**-**	**8.41E-15**
k__Bacteria;p__Actinobacteria;c__Actinobacteria;o__Actinomycetales;f__Actinomycetaceae;g__Trueperella	0.03 ± 0.02	1.2	0.00 ± 0.00	0.0	8.17E-09	2.5993	-	9.95E-09
k__Bacteria;p__Actinobacteria;c__Actinobacteria;o__Actinomycetales;f__Actinomycetaceae;g__Varibaculum	0.00 ± 0.00	0.0	0.00 ± 0.00	0.0	2.92E-01	-	-	-
k__Bacteria;p__Actinobacteria;c__Actinobacteria;o__Bifidobacteriales;f__Bifidobacteriaceae;g__Alloscardovia	0.00 ± 0.00	0.0	0.00 ± 0.00	0.0	2.48E-01	-	-	-
k__Bacteria;p__Actinobacteria;c__Actinobacteria;o__Bifidobacteriales;f__Bifidobacteriaceae;g__Bifidobacterium	0.01 ± 0.01	0.0	1.18 ± 1.04	4.1	9.37E-01	-	-	-
k__Bacteria;p__Actinobacteria;c__Actinobacteria;o__Bifidobacteriales;f__Bifidobacteriaceae;g__Gardnerella	0.42 ± 0.19	9.6	2.62 ± 0.87	17.8	3.79E-01	-	-	-
k__Bacteria;p__Actinobacteria;c__Actinobacteria;o__Bifidobacteriales;f__Bifidobacteriaceae;g__Scardovia	0.00 ± 0.00	0.0	0.00 ± 0.00	0.0	NA	-	-	-
**k__Bacteria;p__Actinobacteria;c__Actinobacteria;o__Corynebacteriales;f__Corynebacteriaceae;g__Corynebacterium**	**1.81 ± 0.49**	**25.3**	**0.21 ± 0.10**	**1.4**	**7.36E-06**	**3.9218**	**-**	**1.11E-05**
k__Bacteria;p__Actinobacteria;c__Actinobacteria;o__Corynebacteriales;f__Dietziaceae;g__Dietzia	0.01 ± 0.01	0.0	0.01 ± 0.00	0.0	3.14E-01	-	-	-
k__Bacteria;p__Actinobacteria;c__Actinobacteria;o__Corynebacteriales;f__Mycobacteriaceae;g__Mycobacterium	0.00 ± 0.00	0.0	0.07 ± 0.03	1.4	3.67E-06	-	2.7568	3.07E-06
k__Bacteria;p__Actinobacteria;c__Actinobacteria;o__Corynebacteriales;f__Nocardiaceae;g__	0.00 ± 0.00	0.0	0.00 ± 0.00	0.0	NA	-	-	-
k__Bacteria;p__Actinobacteria;c__Actinobacteria;o__Frankiales;f__;g__	0.00 ± 0.00	0.0	0.00 ± 0.00	0.0	6.54E-01	-	-	-
k__Bacteria;p__Actinobacteria;c__Actinobacteria;o__Frankiales;f__Antricoccus;g__	0.00 ± 0.00	0.0	0.00 ± 0.00	0.0	3.55E-01	-	-	-
k__Bacteria;p__Actinobacteria;c__Actinobacteria;o__Frankiales;f__Frankiaceae;g__Jatrophihabitans	0.00 ± 0.00	0.0	0.00 ± 0.00	0.0	NA	-	-	-
k__Bacteria;p__Actinobacteria;c__Actinobacteria;o__Kineosporiales;f__Kineosporiaceae;g__Kineococcus	0.00 ± 0.00	0.0	0.00 ± 0.00	0.0	3.55E-01	-	-	-
k__Bacteria;p__Actinobacteria;c__Actinobacteria;o__Micrococcales;f__Bogoriellaceae;g__Bogoriella	0.00 ± 0.00	0.0	0.00 ± 0.00	0.0	1.86E-01	-	-	-
k__Bacteria;p__Actinobacteria;c__Actinobacteria;o__Micrococcales;f__Brevibacteriaceae;g__Brevibacterium	0.02 ± 0.02	1.2	0.00 ± 0.00	0.0	1.04E-01	-	-	-
k__Bacteria;p__Actinobacteria;c__Actinobacteria;o__Micrococcales;f__Cellulomonadaceae;g__Cellulomonas	0.00 ± 0.00	0.0	0.00 ± 0.00	0.0	NA	-	-	-
k__Bacteria;p__Actinobacteria;c__Actinobacteria;o__Micrococcales;f__Dermabacteraceae;g__Brachybacterium	0.01 ± 0.01	0.0	0.00 ± 0.00	0.0	1.04E-01	-	-	-
k__Bacteria;p__Actinobacteria;c__Actinobacteria;o__Micrococcales;f__Dermatophilaceae;g__Dermatophilus	0.00 ± 0.00	0.0	0.00 ± 0.00	0.0	1.04E-01	-	-	-
k__Bacteria;p__Actinobacteria;c__Actinobacteria;o__Micrococcales;f__Dermatophilaceae;g__Kineosphaera	0.00 ± 0.00	0.0	0.00 ± 0.00	0.0	1.86E-01	-	-	-
k__Bacteria;p__Actinobacteria;c__Actinobacteria;o__Micrococcales;f__Dermatophilaceae;g__Piscicoccus	0.00 ± 0.00	0.0	0.00 ± 0.00	0.0	1.86E-01	-	-	-
k__Bacteria;p__Actinobacteria;c__Actinobacteria;o__Micrococcales;f__Intrasporangiaceae;g__Knoellia	0.00 ± 0.00	0.0	0.00 ± 0.00	0.0	1.04E-01	-	-	-
k__Bacteria;p__Actinobacteria;c__Actinobacteria;o__Micrococcales;f__Intrasporangiaceae;g__Phycicoccus	0.00 ± 0.00	0.0	0.00 ± 0.00	0.0	3.55E-01	-	-	-
k__Bacteria;p__Actinobacteria;c__Actinobacteria;o__Micrococcales;f__Microbacteriaceae;g__	0.01 ± 0.01	0.0	0.00 ± 0.00	0.0	1.98E-02	2.7590	-	2.04E-02
k__Bacteria;p__Actinobacteria;c__Actinobacteria;o__Micrococcales;f__Microbacteriaceae;g__Amnibacterium	0.00 ± 0.00	0.0	0.00 ± 0.00	0.0	1.04E-01	-	-	-
k__Bacteria;p__Actinobacteria;c__Actinobacteria;o__Micrococcales;f__Microbacteriaceae;g__Leucobacter	0.00 ± 0.00	0.0	0.00 ± 0.00	0.0	1.04E-01	-	-	-
k__Bacteria;p__Actinobacteria;c__Actinobacteria;o__Micrococcales;f__Micrococcaceae;g__Arthrobacter	0.01 ± 0.00	0.0	0.00 ± 0.00	0.0	1.04E-01	-	-	-
k__Bacteria;p__Actinobacteria;c__Actinobacteria;o__Micrococcales;f__Micrococcaceae;g__Glutamicibacter	0.00 ± 0.00	0.0	0.00 ± 0.00	0.0	NA	-	-	-
k__Bacteria;p__Actinobacteria;c__Actinobacteria;o__Micrococcales;f__Micrococcaceae;g__Kocuria	0.03 ± 0.02	1.2	0.00 ± 0.00	0.0	1.04E-01	-	-	-
k__Bacteria;p__Actinobacteria;c__Actinobacteria;o__Micrococcales;f__Micrococcaceae;g__Micrococcus	0.01 ± 0.00	0.0	0.00 ± 0.00	0.0	1.04E-01	-	-	-
k__Bacteria;p__Actinobacteria;c__Actinobacteria;o__Micrococcales;f__Micrococcaceae;g__Paenarthrobacter	0.00 ± 0.00	0.0	0.00 ± 0.00	0.0	NA	-	-	-
k__Bacteria;p__Actinobacteria;c__Actinobacteria;o__Micrococcales;f__Micrococcaceae;g__Rothia	0.01 ± 0.00	0.0	0.00 ± 0.00	0.0	1.36E-01	-	-	-
k__Bacteria;p__Actinobacteria;c__Actinobacteria;o__Micromonosporales;f__Micromonosporaceae;g__Catellatospora	0.00 ± 0.00	0.0	0.00 ± 0.00	0.0	NA	-	-	-
k__Bacteria;p__Actinobacteria;c__Actinobacteria;o__Nakamurellales;f__Nakamurellaceae;g__Nakamurella	0.00 ± 0.00	0.0	0.00 ± 0.00	0.0	NA	-	-	-
k__Bacteria;p__Actinobacteria;c__Actinobacteria;o__Propionibacteriales;f__Nocardioidaceae;g__Marmoricola	0.00 ± 0.00	0.0	0.00 ± 0.00	0.0	NA	-	-	-
k__Bacteria;p__Actinobacteria;c__Actinobacteria;o__Propionibacteriales;f__Propionibacteriaceae;g__Cutibacterium	0.00 ± 0.00	0.0	0.01 ± 0.01	0.0	2.63E-04	-	2.5410	2.34E-04
k__Bacteria;p__Actinobacteria;c__Actinobacteria;o__Propionibacteriales;f__Propionibacteriaceae;g__Propionimicrobium	0.00 ± 0.00	0.0	0.02 ± 0.01	0.0	8.07E-03	-	2.6550	7.52E-03
k__Bacteria;p__Actinobacteria;c__Actinobacteria;o__Propionibacteriales;f__Propionibacteriaceae;g__Pseudopropionibacterium	0.00 ± 0.00	0.0	0.00 ± 0.00	0.0	NA	-	-	-
k__Bacteria;p__Actinobacteria;c__Actinobacteria;o__Pseudonocardiales;f__Pseudonocardiaceae;g__Actinomycetospora	0.00 ± 0.00	0.0	0.00 ± 0.00	0.0	NA	-	-	-
k__Bacteria;p__Actinobacteria;c__Actinobacteria;o__Pseudonocardiales;f__Pseudonocardiaceae;g__Saccharopolyspora	0.00 ± 0.00	0.0	0.00 ± 0.00	0.0	3.55E-01	-	-	-
k__Bacteria;p__Actinobacteria;c__Actinobacteria;o__Streptosporangiales;f__Thermomonosporaceae;g__Actinoallomurus	0.00 ± 0.00	0.0	0.00 ± 0.00	0.0	3.55E-01	-	-	-
k__Bacteria;p__Actinobacteria;c__Coriobacteriia;o__Coriobacteriales;f__Atopobiaceae;g__	0.09 ± 0.01	0.0	0.00 ± 0.00	0.0	4.40E-18	2.8160	-	6.59E-18
**k__Bacteria;p__Actinobacteria;c__Coriobacteriia;o__Coriobacteriales;f__Atopobiaceae;g__Atopobium**	**2.13 ± 0.44**	**43.4**	**1.24 ± 0.51**	**13.7**	**1.17E-13**	**3.7299**	**-**	**2.06E-13**
k__Bacteria;p__Actinobacteria;c__Coriobacteriia;o__Coriobacteriales;f__Atopobiaceae;g__Olsenella	0.02 ± 0.01	0.0	0.00 ± 0.00	0.0	8.17E-09	2.6358	-	9.95E-09
k__Bacteria;p__Actinobacteria;c__Coriobacteriia;o__Coriobacteriales;f__Coriobacteriaceae;g__	0.03 ± 0.01	0.0	0.00 ± 0.00	0.0	1.06E-09	2.4463	-	1.32E-09
k__Bacteria;p__Actinobacteria;c__Coriobacteriia;o__Coriobacteriales;f__Coriobacteriaceae;g__Collinsella	0.02 ± 0.01	0.0	0.00 ± 0.00	0.0	1.52E-04	2.6190	-	1.70E-04
k__Bacteria;p__Actinobacteria;c__Coriobacteriia;o__Coriobacteriales;f__Coriobacteriaceae;g__Parvibacter	0.00 ± 0.00	0.0	0.00 ± 0.00	0.0	1.86E-01	-	-	-

k__Bacteria;p__Actinobacteria;c__Coriobacteriia;o__Coriobacteriales;f__Coriobacteriaceae;g__Senegalimassilia	0.01 ± 0.00	0.0	0.00 ± 0.00	0.0	1.47E-05	2.8580	-	1.65E-05
k__Bacteria;p__Actinobacteria;c__Coriobacteriia;o__Eggerthellales;f__Eggerthellaceae;g__	0.03 ± 0.02	1.2	0.00 ± 0.00	0.0	4.52E-06	2.8606	-	5.13E-06
k__Bacteria;p__Actinobacteria;c__Coriobacteriia;o__Eggerthellales;f__Eggerthellaceae;g__Eggerthella	0.00 ± 0.00	0.0	0.00 ± 0.00	0.0	7.71E-04	3.1594	-	8.24E-04
k__Bacteria;p__Actinobacteria;c__Coriobacteriia;o__Eggerthellales;f__Eggerthellaceae;g__Enterorhabdus	0.00 ± 0.00	0.0	0.00 ± 0.00	0.0	2.29E-03	3.1083	-	2.42E-03
k__Bacteria;p__Actinobacteria;c__Coriobacteriia;o__Eggerthellales;f__Eggerthellaceae;g__Gordonibacter	0.00 ± 0.00	0.0	0.14 ± 0.06	5.5	8.07E-03	-	2.8761	7.52E-03
k__Bacteria;p__Actinobacteria;c__Coriobacteriia;o__Eggerthellales;f__Eggerthellaceae;g__Raoultibacter	0.00 ± 0.00	0.0	0.00 ± 0.00	0.0	3.55E-01	-	-	-
k__Bacteria;p__Actinobacteria;c__Coriobacteriia;o__Eggerthellales;f__Eggerthellaceae;g__Slackia	0.01 ± 0.00	0.0	0.00 ± 0.00	0.0	1.46E-04	2.9696	-	1.59E-04
k__Bacteria;p__Actinobacteria;c__Thermoleophilia;o__Solirubrobacterales;f__Solirubrobacteraceae;g__Solirubrobacter	0.00 ± 0.00	0.0	0.00 ± 0.00	0.0	NA	-	-	-
k__Bacteria;p__Bacteroidetes;c__;o__;f__;g__	0.00 ± 0.00	0.0	0.00 ± 0.00	0.0	NA	-	-	-
k__Bacteria;p__Bacteroidetes;c__Bacteroidia;o__Bacteroidales;f__;g__	0.02 ± 0.01	1.2	0.00 ± 0.00	0.0	2.55E-04	2.3872	-	2.76E-04
k__Bacteria;p__Bacteroidetes;c__Bacteroidia;o__Bacteroidales;f__Bacteroidaceae;g__	0.35 ± 0.09	2.4	0.00 ± 0.00	0.0	9.56E-28	3.2743	-	1.71E-27
k__Bacteria;p__Bacteroidetes;c__Bacteroidia;o__Bacteroidales;f__Bacteroidaceae;g__Anaerorhabdus	0.00 ± 0.00	0.0	0.00 ± 0.00	0.0	NA	-	-	-
k__Bacteria;p__Bacteroidetes;c__Bacteroidia;o__Bacteroidales;f__Bacteroidaceae;g__Bacteroides	0.02 ± 0.01	0.0	0.13 ± 0.04	2.7	1.79E-03	-	2.8282	1.40E-03
k__Bacteria;p__Bacteroidetes;c__Bacteroidia;o__Bacteroidales;f__Bacteroidales_RF16_group;g__	0.00 ± 0.00	0.0	0.00 ± 0.00	0.0	NA	-	-	-
k__Bacteria;p__Bacteroidetes;c__Bacteroidia;o__Bacteroidales;f__Odoribacteraceae;g__Butyricimonas	0.00 ± 0.00	0.0	0.00 ± 0.00	0.0	1.86E-01	-	-	-
k__Bacteria;p__Bacteroidetes;c__Bacteroidia;o__Bacteroidales;f__Odoribacteraceae;g__Odoribacter	0.00 ± 0.00	0.0	0.00 ± 0.00	0.0	2.92E-01	-	-	-
k__Bacteria;p__Bacteroidetes;c__Bacteroidia;o__Bacteroidales;f__Phocaeicola;g__abscessus	0.00 ± 0.00	0.0	0.00 ± 0.00	0.0	NA	-	-	-
**k__Bacteria;p__Bacteroidetes;c__Bacteroidia;o__Bacteroidales;f__Porphyromonadaceae;g__**	**1.23 ± 0.27**	**36.1**	**0.02 ± 0.01**	**1.4**	**3.16E-26**	**3.7992**	**-**	**5.66E-26**
k__Bacteria;p__Bacteroidetes;c__Bacteroidia;o__Bacteroidales;f__Porphyromonadaceae;g__Barnesiella	0.00 ± 0.00	0.0	0.00 ± 0.00	0.0	2.92E-01	-	-	-
k__Bacteria;p__Bacteroidetes;c__Bacteroidia;o__Bacteroidales;f__Porphyromonadaceae;g__Coprobacter	0.00 ± 0.00	0.0	0.00 ± 0.00	0.0	NA	-	-	-
k__Bacteria;p__Bacteroidetes;c__Bacteroidia;o__Bacteroidales;f__Porphyromonadaceae;g__Muribaculum	0.03 ± 0.01	0.0	0.00 ± 0.00	0.0	1.40E-04	2.4692	-	1.56E-04
k__Bacteria;p__Bacteroidetes;c__Bacteroidia;o__Bacteroidales;f__Porphyromonadaceae;g__Paludibacter	0.10 ± 0.04	3.6	0.00 ± 0.00	0.0	1.06E-09	2.7022	-	1.32E-09
k__Bacteria;p__Bacteroidetes;c__Bacteroidia;o__Bacteroidales;f__Porphyromonadaceae;g__Parabacteroides	0.00 ± 0.00	0.0	0.01 ± 0.00	0.0	3.61E-01	-	-	-
k__Bacteria;p__Bacteroidetes;c__Bacteroidia;o__Bacteroidales;f__Porphyromonadaceae;g__Petrimonas	0.00 ± 0.00	0.0	0.00 ± 0.00	0.0	1.86E-01	-	-	-
**k__Bacteria;p__Bacteroidetes;c__Bacteroidia;o__Bacteroidales;f__Porphyromonadaceae;g__Porphyromonas**	**9.71 ± 0.92**	**94.0**	**0.16 ± 0.08**	**4.1**	**5.09E-26**	**4.6789**	**-**	**8.62E-26**
k__Bacteria;p__Bacteroidetes;c__Bacteroidia;o__Bacteroidales;f__Porphyromonadaceae;g__Proteiniphilum	0.00 ± 0.00	0.0	0.00 ± 0.00	0.0	3.55E-01	-	-	-
k__Bacteria;p__Bacteroidetes;c__Bacteroidia;o__Bacteroidales;f__Porphyromonadaceae;g__Tannerella	0.00 ± 0.00	0.0	0.00 ± 0.00	0.0	NA	-	-	-
**k__Bacteria;p__Bacteroidetes;c__Bacteroidia;o__Bacteroidales;f__Prevotellaceae;g__**	**2.81 ± 0.36**	**56.6**	**0.00 ± 0.00**	**0.0**	**1.70E-25**	**4.1432**	**-**	**2.92E-25**
k__Bacteria;p__Bacteroidetes;c__Bacteroidia;o__Bacteroidales;f__Prevotellaceae;g__Alloprevotella	0.01 ± 0.00	0.0	0.00 ± 0.00	0.0	2.51E-05	3.0197	-	2.84E-05
k__Bacteria;p__Bacteroidetes;c__Bacteroidia;o__Bacteroidales;f__Prevotellaceae;g__Paraprevotella	0.00 ± 0.00	0.0	0.00 ± 0.00	0.0	NA	-	-	-
**k__Bacteria;p__Bacteroidetes;c__Bacteroidia;o__Bacteroidales;f__Prevotellaceae;g__Prevotella**	**6.40 ± 0.60**	**94.0**	**4.32 ± 1.13**	**32.9**	**1.68E-09**	**4.0628**	**-**	**2.85E-09**
k__Bacteria;p__Bacteroidetes;c__Bacteroidia;o__Bacteroidales;f__Prevotellaceae;g__Prevotellamassilia	0.31 ± 0.08	8.4	0.00 ± 0.00	0.0	4.33E-15	3.2233	-	6.17E-15
k__Bacteria;p__Bacteroidetes;c__Bacteroidia;o__Bacteroidales;f__Rikenellaceae;g__	0.02 ± 0.01	0.0	0.00 ± 0.00	0.0	4.12E-04	2.3056	-	4.50E-04
k__Bacteria;p__Bacteroidetes;c__Bacteroidia;o__Bacteroidales;f__Rikenellaceae;g__Alistipes	0.00 ± 0.00	0.0	0.01 ± 0.00	0.0	4.90E-02	-	2.7053	4.59E-02
k__Bacteria;p__Bacteroidetes;c__Bacteroidia;o__Bacteroidales;f__Rikenellaceae;g__Rikenellaceae_RC9_gut_group	0.02 ± 0.01	0.0	0.00 ± 0.00	0.0	1.47E-05	2.2010	-	1.65E-05
k__Bacteria;p__Bacteroidetes;c__Bacteroidia;o__Marinilabiliales;f__Marinilabiliaceae;g__	0.01 ± 0.01	0.0	0.00 ± 0.00	0.0	4.68E-05	2.4650	-	5.17E-05
k__Bacteria;p__Bacteroidetes;c__Bacteroidia;o__Marinilabiliales;f__Prolixibacteraceae;g__Mariniphaga	0.00 ± 0.00	0.0	0.00 ± 0.00	0.0	3.55E-01	-	-	-
k__Bacteria;p__Bacteroidetes;c__Chitinophagia;o__Chitinophagales;f__Chitinophagaceae;g__	0.00 ± 0.00	0.0	0.00 ± 0.00	0.0	NA	-	-	-
k__Bacteria;p__Bacteroidetes;c__Chitinophagia;o__Chitinophagales;f__Chitinophagaceae;g__Chitinophaga	0.00 ± 0.00	0.0	0.00 ± 0.00	0.0	3.55E-01	-	-	-
k__Bacteria;p__Bacteroidetes;c__Chitinophagia;o__Chitinophagales;f__Chitinophagaceae;g__Filimonas	0.00 ± 0.00	0.0	0.00 ± 0.00	0.0	NA	-	-	-
k__Bacteria;p__Bacteroidetes;c__Chitinophagia;o__Chitinophagales;f__Chitinophagaceae;g__Flaviaesturariibacter	0.00 ± 0.00	0.0	0.00 ± 0.00	0.0	NA	-	-	-
k__Bacteria;p__Bacteroidetes;c__Chitinophagia;o__Chitinophagales;f__Chitinophagaceae;g__Flavihumibacter	0.00 ± 0.00	0.0	0.00 ± 0.00	0.0	NA	-	-	-
k__Bacteria;p__Bacteroidetes;c__Chitinophagia;o__Chitinophagales;f__Chitinophagaceae;g__Flavisolibacter	0.00 ± 0.00	0.0	0.00 ± 0.00	0.0	3.55E-01	-	-	-
k__Bacteria;p__Bacteroidetes;c__Chitinophagia;o__Chitinophagales;f__Chitinophagaceae;g__Heliimonas	0.00 ± 0.00	0.0	0.00 ± 0.00	0.0	NA	-	-	-
k__Bacteria;p__Bacteroidetes;c__Chitinophagia;o__Chitinophagales;f__Chitinophagaceae;g__Niabella	0.00 ± 0.00	0.0	0.00 ± 0.00	0.0	NA	-	-	-
k__Bacteria;p__Bacteroidetes;c__Chitinophagia;o__Chitinophagales;f__Chitinophagaceae;g__Sediminibacterium	0.00 ± 0.00	0.0	0.00 ± 0.00	0.0	NA	-	-	-
k__Bacteria;p__Bacteroidetes;c__Chitinophagia;o__Chitinophagales;f__Chitinophagaceae;g__Taibaiella	0.00 ± 0.00	0.0	0.00 ± 0.00	0.0	NA	-	-	-
k__Bacteria;p__Bacteroidetes;c__Chitinophagia;o__Chitinophagales;f__Chitinophagaceae;g__Terrimonas	0.00 ± 0.00	0.0	0.00 ± 0.00	0.0	NA	-	-	-
k__Bacteria;p__Bacteroidetes;c__Cytophagia;o__Cytophagales;f__Cytophagaceae;g__	0.00 ± 0.00	0.0	0.00 ± 0.00	0.0	NA	-	-	-
k__Bacteria;p__Bacteroidetes;c__Cytophagia;o__Cytophagales;f__Cytophagaceae;g__Spirosoma	0.00 ± 0.00	0.0	0.00 ± 0.00	0.0	3.55E-01	-	-	-
k__Bacteria;p__Bacteroidetes;c__Cytophagia;o__Cytophagales;f__Hymenobacteraceae;g__Siccationidurans	0.00 ± 0.00	0.0	0.00 ± 0.00	0.0	1.04E-01	-	-	-
k__Bacteria;p__Bacteroidetes;c__Flavobacteriia;o__Flavobacteriales;f__Crocinitomicaceae;g__Fluviicola	0.00 ± 0.00	0.0	0.00 ± 0.00	0.0	1.04E-01	-	-	-
k__Bacteria;p__Bacteroidetes;c__Flavobacteriia;o__Flavobacteriales;f__Flavobacteriaceae;g__	0.01 ± 0.01	0.0	0.00 ± 0.00	0.0	7.71E-04	2.1274	-	8.24E-04
k__Bacteria;p__Bacteroidetes;c__Flavobacteriia;o__Flavobacteriales;f__Flavobacteriaceae;g__Bergeyella	0.00 ± 0.00	0.0	0.00 ± 0.00	0.0	NA	-	-	-
k__Bacteria;p__Bacteroidetes;c__Flavobacteriia;o__Flavobacteriales;f__Flavobacteriaceae;g__Capnocytophaga	0.00 ± 0.00	0.0	0.00 ± 0.00	0.0	3.55E-01	-	-	-
k__Bacteria;p__Bacteroidetes;c__Flavobacteriia;o__Flavobacteriales;f__Flavobacteriaceae;g__Chryseobacterium	0.06 ± 0.04	2.4	0.00 ± 0.00	0.0	2.08E-01	-	-	-
k__Bacteria;p__Bacteroidetes;c__Flavobacteriia;o__Flavobacteriales;f__Flavobacteriaceae;g__Cloacibacterium	0.00 ± 0.00	0.0	0.01 ± 0.01	0.0	2.92E-01	-	-	-
k__Bacteria;p__Bacteroidetes;c__Flavobacteriia;o__Flavobacteriales;f__Flavobacteriaceae;g__Elizabethkingia	0.00 ± 0.00	0.0	0.08 ± 0.04	2.7	8.07E-03	-	2.7898	7.52E-03
k__Bacteria;p__Bacteroidetes;c__Flavobacteriia;o__Flavobacteriales;f__Flavobacteriaceae;g__Empedobacter	0.00 ± 0.00	0.0	0.00 ± 0.00	0.0	1.86E-01	-	-	-
k__Bacteria;p__Bacteroidetes;c__Flavobacteriia;o__Flavobacteriales;f__Flavobacteriaceae;g__Flavobacterium	0.01 ± 0.01	0.0	0.00 ± 0.00	0.0	2.15E-01	-	-	-
k__Bacteria;p__Bacteroidetes;c__Flavobacteriia;o__Flavobacteriales;f__Flavobacteriaceae;g__Riemerella	0.01 ± 0.00	0.0	0.00 ± 0.00	0.0	1.04E-01	-	-	-
k__Bacteria;p__Bacteroidetes;c__Flavobacteriia;o__Flavobacteriales;f__Flavobacteriaceae;g__Soonwooa	0.01 ± 0.01	0.0	0.00 ± 0.00	0.0	1.04E-01	-	-	-
k__Bacteria;p__Bacteroidetes;c__Sphingobacteriia;o__Sphingobacteriales;f__Chitinophagaceae;g__Taibaiella	0.00 ± 0.00	0.0	0.00 ± 0.00	0.0	1.04E-01	-	-	-

k__Bacteria;p__Bacteroidetes;c__Sphingobacteriia;o__Sphingobacteriales;f__Lentimicrobiaceae;g__	0.00 ± 0.00	0.0	0.00 ± 0.00	0.0	NA	-	-	-
k__Bacteria;p__Bacteroidetes;c__Sphingobacteriia;o__Sphingobacteriales;f__Sphingobacteriaceae;g__	0.03 ± 0.01	0.0	0.00 ± 0.00	0.0	2.10E-07	2.3644	-	2.47E-07
k__Bacteria;p__Bacteroidetes;c__Sphingobacteriia;o__Sphingobacteriales;f__Sphingobacteriaceae;g__Mucilaginibacter	0.00 ± 0.00	0.0	0.00 ± 0.00	0.0	NA	-	-	-
k__Bacteria;p__Bacteroidetes;c__Sphingobacteriia;o__Sphingobacteriales;f__Sphingobacteriaceae;g__Pedobacter	0.00 ± 0.00	0.0	0.00 ± 0.00	0.0	3.55E-01	-	-	-
k__Bacteria;p__Bacteroidetes;c__Sphingobacteriia;o__Sphingobacteriales;f__Sphingobacteriaceae;g__Sphingobacterium	0.05 ± 0.03	1.2	0.00 ± 0.00	0.0	1.04E-01	-	-	-
k__Bacteria;p__Chlamydiae;c__Chlamydiia;o__Parachlamydiales;f__;g__	0.00 ± 0.00	0.0	0.00 ± 0.00	0.0	NA	-	-	-
k__Bacteria;p__Chlamydiae;c__Chlamydiia;o__Parachlamydiales;f__Parachlamydiaceae;g__Protochlamydia	0.00 ± 0.00	0.0	0.00 ± 0.00	0.0	NA	-	-	-
k__Bacteria;p__Chlorobi;c__Chlorobia;o__Chlorobiales;f__OPB56;g__	0.00 ± 0.00	0.0	0.00 ± 0.00	0.0	NA	-	-	-
k__Bacteria;p__Chloroflexi;c__Anaerolineae;o__Anaerolineales;f__Anaerolineaceae;g__	0.00 ± 0.00	0.0	0.00 ± 0.00	0.0	NA	-	-	-
k__Bacteria;p__Chloroflexi;c__Thermomicrobia;o__Sphaerobacterales;f__Sphaerobacteraceae;g__	0.00 ± 0.00	0.0	0.00 ± 0.00	0.0	5.91E-02	-	-	-
k__Bacteria;p__Cyanobacteria;c__Cyanobacteria;o__SubsectionIII;f__FamilyI;g__	0.00 ± 0.00	0.0	0.00 ± 0.00	0.0	3.55E-01	-	-	-
k__Bacteria;p__Cyanobacteria;c__Cyanobacteria;o__SubsectionIV;f__FamilyI;g__Trichormus	0.00 ± 0.00	0.0	0.00 ± 0.00	0.0	NA	-	-	-
k__Bacteria;p__Cyanobacteria;c__Melainabacteria;o__Gastranaerophilales;f__;g__	0.03 ± 0.01	0.0	0.00 ± 0.00	0.0	4.52E-06	2.6959	-	5.13E-06
k__Bacteria;p__Cyanobacteria;c__Melainabacteria;o__Obscuribacterales;f__;g__	0.00 ± 0.00	0.0	0.08 ± 0.02	1.4	3.20E-05	-	2.7270	2.76E-05
k__Bacteria;p__Deferribacteres;c__Deferribacteres;o__Deferribacterales;f__Deferribacteraceae;g__Seleniivibrio	0.00 ± 0.00	0.0	0.00 ± 0.00	0.0	NA	-	-	-
k__Bacteria;p__Deinococcus-Thermus;c__Deinococci;o__Deinococcales;f__Deinococcaceae;g__Deinococcus	0.00 ± 0.00	0.0	0.00 ± 0.00	0.0	3.55E-01	-	-	-
k__Bacteria;p__Elusimicrobia;c__Elusimicrobia;o__Elusimicrobiales;f__Elusimicrobiaceae;g__	0.00 ± 0.00	0.0	0.00 ± 0.00	0.0	NA	-	-	-
k__Bacteria;p__Elusimicrobia;c__Elusimicrobia;o__Elusimicrobiales;f__Elusimicrobiaceae;g__Elusimicrobium	0.00 ± 0.00	0.0	0.00 ± 0.00	0.0	3.41E-02	3.7711	-	3.49E-02
k__Bacteria;p__Fibrobacteres;c__Fibrobacteria;o__Fibrobacterales;f__Fibrobacteraceae;g__Fibrobacter	0.00 ± 0.00	0.0	0.00 ± 0.00	0.0	3.41E-02	3.6352	-	3.49E-02
k__Bacteria;p__Firmicutes;c__Bacilli;o__Bacillales;f__Alicyclobacillaceae;g__Alicyclobacillus	0.01 ± 0.00	0.0	0.00 ± 0.00	0.0	8.17E-09	2.9018	-	9.95E-09
k__Bacteria;p__Firmicutes;c__Bacilli;o__Bacillales;f__Bacillaceae;g__	0.00 ± 0.00	0.0	0.00 ± 0.00	0.0	1.98E-02	3.4346	-	2.04E-02
k__Bacteria;p__Firmicutes;c__Bacilli;o__Bacillales;f__Bacillaceae;g__Anoxybacillus	0.00 ± 0.00	0.0	0.00 ± 0.00	0.0	NA	-	-	-
k__Bacteria;p__Firmicutes;c__Bacilli;o__Bacillales;f__Bacillaceae;g__Bacillus	0.00 ± 0.00	0.0	0.00 ± 0.00	0.0	9.27E-01	-	-	-
k__Bacteria;p__Firmicutes;c__Bacilli;o__Bacillales;f__Bacillaceae;g__Geobacillus	0.00 ± 0.00	0.0	0.00 ± 0.00	0.0	3.55E-01	-	-	-
k__Bacteria;p__Firmicutes;c__Bacilli;o__Bacillales;f__Bacillales;g__Thermicanus	0.00 ± 0.00	0.0	0.00 ± 0.00	0.0	NA	-	-	-
k__Bacteria;p__Firmicutes;c__Bacilli;o__Bacillales;f__Exiguobacterium;g__	0.00 ± 0.00	0.0	0.00 ± 0.00	0.0	NA	-	-	-
k__Bacteria;p__Firmicutes;c__Bacilli;o__Bacillales;f__Family_XI;g__Gemella	0.00 ± 0.00	0.0	0.07 ± 0.03	2.7	6.31E-02	-	-	-
k__Bacteria;p__Firmicutes;c__Bacilli;o__Bacillales;f__Paenibacillaceae;g__	0.01 ± 0.00	0.0	0.00 ± 0.00	0.0	2.63E-05	2.3391	-	2.93E-05
k__Bacteria;p__Firmicutes;c__Bacilli;o__Bacillales;f__Paenibacillaceae;g__Cohnella	0.00 ± 0.00	0.0	0.00 ± 0.00	0.0	NA	-	-	-
k__Bacteria;p__Firmicutes;c__Bacilli;o__Bacillales;f__Paenibacillaceae;g__Paenibacillus	0.00 ± 0.00	0.0	0.00 ± 0.00	0.0	3.55E-01	-	-	-
k__Bacteria;p__Firmicutes;c__Bacilli;o__Bacillales;f__Planococcaceae;g__Kurthia	0.00 ± 0.00	0.0	0.00 ± 0.00	0.0	3.55E-01	-	-	-
k__Bacteria;p__Firmicutes;c__Bacilli;o__Bacillales;f__Staphylococcaceae;g__Jeotgalicoccus	0.02 ± 0.02	1.2	0.00 ± 0.00	0.0	1.04E-01	-	-	-
k__Bacteria;p__Firmicutes;c__Bacilli;o__Bacillales;f__Staphylococcaceae;g__Macrococcus	0.01 ± 0.00	0.0	0.00 ± 0.00	0.0	1.04E-01	-	-	-
k__Bacteria;p__Firmicutes;c__Bacilli;o__Bacillales;f__Staphylococcaceae;g__Staphylococcus	0.01 ± 0.00	0.0	0.19 ± 0.13	2.7	8.80E-05	-	3.1523	7.01E-05
k__Bacteria;p__Firmicutes;c__Bacilli;o__Bacillales;f__Thermoactinomycetaceae;g__	0.00 ± 0.00	0.0	0.00 ± 0.00	0.0	3.41E-02	3.0720	-	3.49E-02
k__Bacteria;p__Firmicutes;c__Bacilli;o__Lactobacillales;f__Aerococcaceae;g__	0.07 ± 0.02	0.0	0.00 ± 0.00	0.0	2.99E-11	2.7074	-	3.86E-11
k__Bacteria;p__Firmicutes;c__Bacilli;o__Lactobacillales;f__Aerococcaceae;g__Abiotrophia	0.00 ± 0.00	0.0	0.00 ± 0.00	0.0	3.55E-01	-	-	-
k__Bacteria;p__Firmicutes;c__Bacilli;o__Lactobacillales;f__Aerococcaceae;g__Aerococcus	0.97 ± 0.29	15.7	0.08 ± 0.04	1.4	5.22E-12	3.6675	-	7.97E-12
k__Bacteria;p__Firmicutes;c__Bacilli;o__Lactobacillales;f__Aerococcaceae;g__Dolosicoccus	0.06 ± 0.02	1.2	0.00 ± 0.00	0.0	4.17E-09	2.6389	-	5.12E-09
k__Bacteria;p__Firmicutes;c__Bacilli;o__Lactobacillales;f__Aerococcaceae;g__Eremococcus	0.23 ± 0.12	6.0	0.00 ± 0.00	0.0	1.59E-08	3.0293	-	1.92E-08
k__Bacteria;p__Firmicutes;c__Bacilli;o__Lactobacillales;f__Aerococcaceae;g__Facklamia	0.87 ± 0.21	20.5	0.00 ± 0.00	0.0	3.94E-23	3.6349	-	6.57E-23
k__Bacteria;p__Firmicutes;c__Bacilli;o__Lactobacillales;f__Carnobacteriaceae;g__Alkalibacterium	0.00 ± 0.00	0.0	0.00 ± 0.00	0.0	3.55E-01	-	-	-
k__Bacteria;p__Firmicutes;c__Bacilli;o__Lactobacillales;f__Carnobacteriaceae;g__Atopostipes	0.15 ± 0.05	3.6	0.00 ± 0.00	0.0	3.97E-16	2.9099	-	5.71E-16
k__Bacteria;p__Firmicutes;c__Bacilli;o__Lactobacillales;f__Carnobacteriaceae;g__Dolosigranulum	0.00 ± 0.00	0.0	0.00 ± 0.00	0.0	NA	-	-	-
k__Bacteria;p__Firmicutes;c__Bacilli;o__Lactobacillales;f__Carnobacteriaceae;g__Granulicatella	0.00 ± 0.00	0.0	0.00 ± 0.00	0.0	1.37E-01	-	-	-
k__Bacteria;p__Firmicutes;c__Bacilli;o__Lactobacillales;f__Enterococcaceae;g__Enterococcus	0.00 ± 0.00	0.0	0.01 ± 0.01	0.0	1.27E-01	-	-	-
**k__Bacteria;p__Firmicutes;c__Bacilli;o__Lactobacillales;f__Lactobacillaceae;g__Lactobacillus**	**0.35 ± 0.07**	**13.3**	**78.65 ± 3.36**	**98.6**	**8.96E-26**	**-**	**5.5900**	**1.37E-25**
k__Bacteria;p__Firmicutes;c__Bacilli;o__Lactobacillales;f__Lactobacillaceae;g__Pediococcus	0.00 ± 0.00	0.0	0.00 ± 0.00	0.0	NA	-	-	-
k__Bacteria;p__Firmicutes;c__Bacilli;o__Lactobacillales;f__Leuconostocaceae;g__Leuconostoc	0.00 ± 0.00	0.0	0.00 ± 0.00	0.0	NA	-	-	-
k__Bacteria;p__Firmicutes;c__Bacilli;o__Lactobacillales;f__Leuconostocaceae;g__Weissella	0.00 ± 0.00	0.0	0.00 ± 0.00	0.0	7.71E-04	3.3600	-	8.24E-04
k__Bacteria;p__Firmicutes;c__Bacilli;o__Lactobacillales;f__Streptococcaceae;g__Lactococcus	0.00 ± 0.00	0.0	0.00 ± 0.00	0.0	3.55E-01	-	-	-
k__Bacteria;p__Firmicutes;c__Bacilli;o__Lactobacillales;f__Streptococcaceae;g__Streptococcus	1.05 ± 0.57	13.3	0.71 ± 0.26	15.1	1.67E-01	-	-	-
k__Bacteria;p__Firmicutes;c__Clostridia;o__Clostridiales;f__;g__	0.01 ± 0.00	0.0	0.00 ± 0.00	0.0	1.37E-04	3.1767	-	1.52E-04
k__Bacteria;p__Firmicutes;c__Clostridia;o__Clostridiales;f__Bacteroides;g__pectinophilus	0.00 ± 0.00	0.0	0.00 ± 0.00	0.0	1.98E-02	3.5353	-	2.04E-02
k__Bacteria;p__Firmicutes;c__Clostridia;o__Clostridiales;f__Caldicoprobacteraceae;g__	0.00 ± 0.00	0.0	0.00 ± 0.00	0.0	6.75E-03	3.1815	-	7.04E-03
k__Bacteria;p__Firmicutes;c__Clostridia;o__Clostridiales;f__Christensenellaceae;g__	0.02 ± 0.01	0.0	0.00 ± 0.00	0.0	3.06E-08	2.2714	-	3.68E-08
k__Bacteria;p__Firmicutes;c__Clostridia;o__Clostridiales;f__Christensenellaceae;g__Christensenella	0.00 ± 0.00	0.0	0.00 ± 0.00	0.0	3.85E-01	-	-	-
k__Bacteria;p__Firmicutes;c__Clostridia;o__Clostridiales;f__Christensenellaceae;g__Christensenellaceae_R-7_group	0.06 ± 0.01	0.0	0.00 ± 0.00	0.0	7.74E-12	2.6988	-	1.04E-11
k__Bacteria;p__Firmicutes;c__Clostridia;o__Clostridiales;f__Clostridiaceae;g__	0.81 ± 0.22	15.7	0.00 ± 0.00	0.0	2.63E-24	3.5886	-	4.45E-24
k__Bacteria;p__Firmicutes;c__Clostridia;o__Clostridiales;f__Clostridiaceae;g__Beduini	0.01 ± 0.00	0.0	0.00 ± 0.00	0.0	4.68E-05	2.8580	-	5.17E-05
k__Bacteria;p__Firmicutes;c__Clostridia;o__Clostridiales;f__Clostridiaceae;g__Butyricicoccus	0.06 ± 0.01	0.0	0.00 ± 0.00	0.0	3.07E-13	2.6031	-	4.15E-13
k__Bacteria;p__Firmicutes;c__Clostridia;o__Clostridiales;f__Clostridiaceae;g__Caloramator	0.00 ± 0.00	0.0	0.00 ± 0.00	0.0	3.55E-01	-	-	-
k__Bacteria;p__Firmicutes;c__Clostridia;o__Clostridiales;f__Clostridiaceae;g__Clostridium	0.22 ± 0.07	4.8	0.26 ± 0.12	6.8	4.38E-06	-	2.7762	5.78E-06

k__Bacteria;p__Firmicutes;c__Clostridia;o__Clostridiales;f__Clostridiaceae;g__Hungatella	0.04 ± 0.01	0.0	0.00 ± 0.00	0.0	8.17E-09	2.4321	-	9.95E-09
k__Bacteria;p__Firmicutes;c__Clostridia;o__Clostridiales;f__Clostridiaceae;g__Lactonifactor	0.00 ± 0.00	0.0	0.00 ± 0.00	0.0	NA	-	-	-
k__Bacteria;p__Firmicutes;c__Clostridia;o__Clostridiales;f__Clostridiaceae;g__Oxobacter	0.00 ± 0.00	0.0	0.00 ± 0.00	0.0	NA	-	-	-
k__Bacteria;p__Firmicutes;c__Clostridia;o__Clostridiales;f__Clostridiales;g__	0.01 ± 0.00	0.0	0.00 ± 0.00	0.0	2.29E-03	2.0675	-	2.42E-03
k__Bacteria;p__Firmicutes;c__Clostridia;o__Clostridiales;f__Clostridiales;g__Anaerovorax	0.01 ± 0.00	0.0	0.00 ± 0.00	0.0	7.32E-07	2.5179	-	8.48E-07
k__Bacteria;p__Firmicutes;c__Clostridia;o__Clostridiales;f__Clostridiales;g__Casaltella	0.00 ± 0.00	0.0	0.00 ± 0.00	0.0	3.55E-01	-	-	-
k__Bacteria;p__Firmicutes;c__Clostridia;o__Clostridiales;f__Clostridiales;g__Emergencia	0.00 ± 0.00	0.0	0.00 ± 0.00	0.0	1.33E-03	2.9484	-	1.41E-03
k__Bacteria;p__Firmicutes;c__Clostridia;o__Clostridiales;f__Clostridiales;g__Eubacterium	0.00 ± 0.00	0.0	0.00 ± 0.00	0.0	6.75E-03	3.3811	-	7.04E-03
k__Bacteria;p__Firmicutes;c__Clostridia;o__Clostridiales;f__Clostridiales;g__Ihubacter	0.00 ± 0.00	0.0	0.00 ± 0.00	0.0	1.16E-02	2.2065	-	1.20E-02
k__Bacteria;p__Firmicutes;c__Clostridia;o__Clostridiales;f__Clostridiales;g__Mogibacterium	0.00 ± 0.00	0.0	0.00 ± 0.00	0.0	2.29E-03	2.9154	-	2.42E-03
k__Bacteria;p__Firmicutes;c__Clostridia;o__Clostridiales;f__Defluviitaleaceae;g__	0.00 ± 0.00	0.0	0.00 ± 0.00	0.0	5.91E-02	-	-	-
k__Bacteria;p__Firmicutes;c__Clostridia;o__Clostridiales;f__Defluviitaleaceae;g__Defluviitalea	0.00 ± 0.00	0.0	0.00 ± 0.00	0.0	NA	-	-	-
k__Bacteria;p__Firmicutes;c__Clostridia;o__Clostridiales;f__Defluviitaleaceae;g__Vallitalea	0.00 ± 0.00	0.0	0.00 ± 0.00	0.0	1.04E-01	-	-	-
k__Bacteria;p__Firmicutes;c__Clostridia;o__Clostridiales;f__Eubacteriaceae;g__	0.06 ± 0.01	0.0	0.00 ± 0.00	0.0	1.43E-11	2.6991	-	1.86E-11
k__Bacteria;p__Firmicutes;c__Clostridia;o__Clostridiales;f__Eubacteriaceae;g__Anaerofustis	0.00 ± 0.00	0.0	0.00 ± 0.00	0.0	NA	-	-	-
k__Bacteria;p__Firmicutes;c__Clostridia;o__Clostridiales;f__Eubacteriaceae;g__Eubacterium	0.43 ± 0.09	12.0	0.01 ± 0.01	0.0	1.01E-16	3.3263	-	1.55E-16
k__Bacteria;p__Firmicutes;c__Clostridia;o__Clostridiales;f__Family_XI;g__Murdochiella	0.01 ± 0.00	0.0	0.04 ± 0.02	0.0	2.35E-01	-	-	-
k__Bacteria;p__Firmicutes;c__Clostridia;o__Clostridiales;f__Family_XIII;g__Family_XIII_UCG-001	0.06 ± 0.01	0.0	0.00 ± 0.00	0.0	3.07E-13	2.7796	-	4.15E-13
k__Bacteria;p__Firmicutes;c__Clostridia;o__Clostridiales;f__Fenollaria;g__massiliensis	0.59 ± 0.24	13.3	0.10 ± 0.04	2.7	7.22E-04	3.5214	-	8.74E-04
k__Bacteria;p__Firmicutes;c__Clostridia;o__Clostridiales;f__Flavonifractor;g__	0.00 ± 0.00	0.0	0.00 ± 0.00	0.0	1.98E-02	3.5159	-	2.04E-02
k__Bacteria;p__Firmicutes;c__Clostridia;o__Clostridiales;f__Flavonifractor;g__plautii	0.00 ± 0.00	0.0	0.00 ± 0.00	0.0	6.54E-01	-	-	-
k__Bacteria;p__Firmicutes;c__Clostridia;o__Clostridiales;f__Flintibacter;g__	0.04 ± 0.01	0.0	0.00 ± 0.00	0.0	5.86E-08	2.4437	-	6.99E-08
k__Bacteria;p__Firmicutes;c__Clostridia;o__Clostridiales;f__Gracilibacteraceae;g__	0.00 ± 0.00	0.0	0.00 ± 0.00	0.0	2.29E-03	2.8424	-	2.42E-03
k__Bacteria;p__Firmicutes;c__Clostridia;o__Clostridiales;f__Gracilibacteraceae;g__Gracilibacter	0.00 ± 0.00	0.0	0.00 ± 0.00	0.0	3.41E-02	3.9180	-	3.49E-02
k__Bacteria;p__Firmicutes;c__Clostridia;o__Clostridiales;f__Heliobacteriaceae;g__	0.00 ± 0.00	0.0	0.00 ± 0.00	0.0	4.44E-04	2.8156	-	4.78E-04
k__Bacteria;p__Firmicutes;c__Clostridia;o__Clostridiales;f__Howardella;g__	0.01 ± 0.00	0.0	0.13 ± 0.06	2.7	3.51E-01	-	-	-
k__Bacteria;p__Firmicutes;c__Clostridia;o__Clostridiales;f__Howardella;g__ureilytica	0.00 ± 0.00	0.0	0.00 ± 0.00	0.0	1.86E-01	-	-	-
k__Bacteria;p__Firmicutes;c__Clostridia;o__Clostridiales;f__Intestinimonas;g__	0.10 ± 0.02	1.2	0.00 ± 0.00	0.0	6.96E-13	2.7261	-	9.45E-13
**k__Bacteria;p__Firmicutes;c__Clostridia;o__Clostridiales;f__Lachnospiraceae;g__**	**2.62 ± 0.36**	**55.4**	**0.00 ± 0.00**	**0.0**	**1.63E-28**	**4.0975**	**-**	**2.96E-28**
k__Bacteria;p__Firmicutes;c__Clostridia;o__Clostridiales;f__Lachnospiraceae;g__Acetatifactor	0.00 ± 0.00	0.0	0.00 ± 0.00	0.0	1.98E-02	3.4890	-	2.04E-02
k__Bacteria;p__Firmicutes;c__Clostridia;o__Clostridiales;f__Lachnospiraceae;g__Acetitomaculum	0.00 ± 0.00	0.0	0.00 ± 0.00	0.0	NA	-	-	-
k__Bacteria;p__Firmicutes;c__Clostridia;o__Clostridiales;f__Lachnospiraceae;g__Anaerobium	0.00 ± 0.00	0.0	0.00 ± 0.00	0.0	7.71E-04	2.6641	-	8.24E-04
k__Bacteria;p__Firmicutes;c__Clostridia;o__Clostridiales;f__Lachnospiraceae;g__Anaerocolumna	0.00 ± 0.00	0.0	0.00 ± 0.00	0.0	1.86E-01	-	-	-
k__Bacteria;p__Firmicutes;c__Clostridia;o__Clostridiales;f__Lachnospiraceae;g__Anaerosporobacter	0.04 ± 0.03	1.2	0.00 ± 0.00	0.0	1.11E-07	2.6623	-	1.32E-07
k__Bacteria;p__Firmicutes;c__Clostridia;o__Clostridiales;f__Lachnospiraceae;g__Anaerostipes	0.17 ± 0.04	6.0	0.00 ± 0.00	0.0	1.24E-10	2.9636	-	1.63E-10
k__Bacteria;p__Firmicutes;c__Clostridia;o__Clostridiales;f__Lachnospiraceae;g__Blautia	0.41 ± 0.08	19.3	0.02 ± 0.01	0.0	3.35E-12	3.3123	-	4.99E-12
**k__Bacteria;p__Firmicutes;c__Clostridia;o__Clostridiales;f__Lachnospiraceae;g__Butyrivibrio**	**2.08 ± 0.43**	**42.2**	**0.00 ± 0.00**	**0.0**	**3.66E-20**	**4.0086**	**-**	**5.71E-20**
k__Bacteria;p__Firmicutes;c__Clostridia;o__Clostridiales;f__Lachnospiraceae;g__Catonella	0.47 ± 0.13	12.0	0.00 ± 0.00	0.0	1.43E-14	3.3277	-	2.00E-14
k__Bacteria;p__Firmicutes;c__Clostridia;o__Clostridiales;f__Lachnospiraceae;g__Cellulosilyticum	0.00 ± 0.00	0.0	0.00 ± 0.00	0.0	NA	-	-	-
k__Bacteria;p__Firmicutes;c__Clostridia;o__Clostridiales;f__Lachnospiraceae;g__[Clostridium]	0.35 ± 0.08	12.0	0.00 ± 0.00	0.0	3.08E-15	3.2554	-	4.60E-15
k__Bacteria;p__Firmicutes;c__Clostridia;o__Clostridiales;f__Lachnospiraceae;g__Coprococcus	0.08 ± 0.02	0.0	0.00 ± 0.00	0.0	2.07E-10	2.6819	-	2.70E-10
k__Bacteria;p__Firmicutes;c__Clostridia;o__Clostridiales;f__Lachnospiraceae;g__Cuneatibacter	0.01 ± 0.00	0.0	0.00 ± 0.00	0.0	8.28E-05	2.4955	-	9.09E-05
k__Bacteria;p__Firmicutes;c__Clostridia;o__Clostridiales;f__Lachnospiraceae;g__Desulfotomaculum	0.09 ± 0.03	1.2	0.00 ± 0.00	0.0	3.03E-10	2.7993	-	3.92E-10
k__Bacteria;p__Firmicutes;c__Clostridia;o__Clostridiales;f__Lachnospiraceae;g__Dorea	0.20 ± 0.05	6.0	0.00 ± 0.00	0.0	4.44E-15	3.0473	-	6.60E-15
k__Bacteria;p__Firmicutes;c__Clostridia;o__Clostridiales;f__Lachnospiraceae;g__Eisenbergiella	0.01 ± 0.00	0.0	0.00 ± 0.00	0.0	1.14E-04	2.6678	-	1.29E-04
k__Bacteria;p__Firmicutes;c__Clostridia;o__Clostridiales;f__Lachnospiraceae;g__[Eubacterium]	0.21 ± 0.06	6.0	0.02 ± 0.01	0.0	1.88E-08	3.0276	-	2.55E-08
k__Bacteria;p__Firmicutes;c__Clostridia;o__Clostridiales;f__Lachnospiraceae;g__Frisingicoccus	0.00 ± 0.00	0.0	0.00 ± 0.00	0.0	2.92E-01	-	-	-
k__Bacteria;p__Firmicutes;c__Clostridia;o__Clostridiales;f__Lachnospiraceae;g__Fusicatenibacter	0.03 ± 0.01	0.0	0.00 ± 0.00	0.0	3.22E-07	2.9654	-	3.98E-07
k__Bacteria;p__Firmicutes;c__Clostridia;o__Clostridiales;f__Lachnospiraceae;g__Hespellia	0.00 ± 0.00	0.0	0.00 ± 0.00	0.0	5.91E-02	-	-	-
k__Bacteria;p__Firmicutes;c__Clostridia;o__Clostridiales;f__Lachnospiraceae;g__Johnsonella	0.00 ± 0.00	0.0	0.00 ± 0.00	0.0	NA	-	-	-
k__Bacteria;p__Firmicutes;c__Clostridia;o__Clostridiales;f__Lachnospiraceae;g__Lachnoanaerobaculum	0.00 ± 0.00	0.0	0.00 ± 0.00	0.0	1.33E-01	-	-	-
k__Bacteria;p__Firmicutes;c__Clostridia;o__Clostridiales;f__Lachnospiraceae;g__Lachnospira	0.01 ± 0.00	0.0	0.00 ± 0.00	0.0	1.21E-03	3.1573	-	1.31E-03
k__Bacteria;p__Firmicutes;c__Clostridia;o__Clostridiales;f__Lachnospiraceae;g__Lachnospiraceae_ND3007_group	0.00 ± 0.00	0.0	0.00 ± 0.00	0.0	NA	-	-	-
k__Bacteria;p__Firmicutes;c__Clostridia;o__Clostridiales;f__Lachnospiraceae;g__Moryella	0.00 ± 0.00	0.0	0.00 ± 0.00	0.0	NA	-	-	-
k__Bacteria;p__Firmicutes;c__Clostridia;o__Clostridiales;f__Lachnospiraceae;g__Muricomes	0.00 ± 0.00	0.0	0.00 ± 0.00	0.0	NA	-	-	-
k__Bacteria;p__Firmicutes;c__Clostridia;o__Clostridiales;f__Lachnospiraceae;g__Murimonas	0.01 ± 0.00	0.0	0.00 ± 0.00	0.0	1.35E-06	3.0988	-	1.56E-06
k__Bacteria;p__Firmicutes;c__Clostridia;o__Clostridiales;f__Lachnospiraceae;g__Oribacterium	0.00 ± 0.00	0.0	0.00 ± 0.00	0.0	9.27E-01	-	-	-
k__Bacteria;p__Firmicutes;c__Clostridia;o__Clostridiales;f__Lachnospiraceae;g__Pseudobutyrivibrio	0.01 ± 0.00	0.0	0.00 ± 0.00	0.0	2.55E-04	3.0437	-	2.76E-04
k__Bacteria;p__Firmicutes;c__Clostridia;o__Clostridiales;f__Lachnospiraceae;g__Roseburia	0.18 ± 0.06	7.2	0.01 ± 0.00	0.0	1.14E-09	2.9768	-	1.52E-09
k__Bacteria;p__Firmicutes;c__Clostridia;o__Clostridiales;f__Lachnospiraceae;g__Ruminococcus	0.00 ± 0.00	0.0	0.01 ± 0.00	0.0	8.07E-03	-	3.0386	7.52E-03
k__Bacteria;p__Firmicutes;c__Clostridia;o__Clostridiales;f__Lachnospiraceae;g__Shuttleworthia	0.00 ± 0.00	0.0	0.91 ± 0.69	2.7	6.41E-02	-	-	-
k__Bacteria;p__Firmicutes;c__Clostridia;o__Clostridiales;f__Lachnospiraceae;g__Stomatobaculum	0.00 ± 0.00	0.0	0.03 ± 0.02	0.0	4.09E-03	-	2.7677	3.77E-03
k__Bacteria;p__Firmicutes;c__Clostridia;o__Clostridiales;f__Lachnospiraceae;g__Tyzzerella	0.00 ± 0.00	0.0	0.00 ± 0.00	0.0	NA	-	-	-

k__Bacteria;p__Firmicutes;c__Clostridia;o__Clostridiales;f__Levyella;g__	0.00 ± 0.00	0.0	0.00 ± 0.00	0.0	7.71E-04	3.6089	-	8.24E-04
k__Bacteria;p__Firmicutes;c__Clostridia;o__Clostridiales;f__Oscillospiraceae;g__	0.00 ± 0.00	0.0	0.00 ± 0.00	0.0	3.55E-01	-	-	-
k__Bacteria;p__Firmicutes;c__Clostridia;o__Clostridiales;f__Oscillospiraceae;g__Oscillibacter	0.21 ± 0.07	6.0	0.01 ± 0.00	0.0	8.35E-12	3.0112	-	1.17E-11
k__Bacteria;p__Firmicutes;c__Clostridia;o__Clostridiales;f__Peptococcaceae;g__	0.03 ± 0.01	0.0	0.00 ± 0.00	0.0	1.11E-07	2.3778	-	1.32E-07
k__Bacteria;p__Firmicutes;c__Clostridia;o__Clostridiales;f__Peptococcaceae;g__Peptococcus	0.12 ± 0.02	0.0	0.01 ± 0.00	0.0	3.66E-19	2.9416	-	6.12E-19
**k__Bacteria;p__Firmicutes;c__Clostridia;o__Clostridiales;f__Peptostreptococcaceae;g__**	**1.58 ± 0.25**	**43.4**	**0.00 ± 0.00**	**0.0**	**9.99E-25**	**3.8819**	**-**	**1.70E-24**
k__Bacteria;p__Firmicutes;c__Clostridia;o__Clostridiales;f__Peptostreptococcaceae;g__Clostridioides	0.00 ± 0.00	0.0	0.00 ± 0.00	0.0	3.55E-01	-	-	-
k__Bacteria;p__Firmicutes;c__Clostridia;o__Clostridiales;f__Peptostreptococcaceae;g__Eubacterium	0.00 ± 0.00	0.0	0.00 ± 0.00	0.0	3.55E-01	-	-	-
k__Bacteria;p__Firmicutes;c__Clostridia;o__Clostridiales;f__Peptostreptococcaceae;g__Filifactor	0.00 ± 0.00	0.0	0.00 ± 0.00	0.0	NA	-	-	-
k__Bacteria;p__Firmicutes;c__Clostridia;o__Clostridiales;f__Peptostreptococcaceae;g__Intestinibacter	0.03 ± 0.01	0.0	0.00 ± 0.00	0.0	1.47E-05	2.3918	-	1.65E-05
k__Bacteria;p__Firmicutes;c__Clostridia;o__Clostridiales;f__Peptostreptococcaceae;g__Peptostreptococcus	0.17 ± 0.06	4.8	0.06 ± 0.02	0.0	2.41E-03	2.7884	-	2.86E-03
k__Bacteria;p__Firmicutes;c__Clostridia;o__Clostridiales;f__Peptostreptococcaceae;g__Romboutsia	0.01 ± 0.00	0.0	0.00 ± 0.00	0.0	8.14E-02	-	-	-
k__Bacteria;p__Firmicutes;c__Clostridia;o__Clostridiales;f__Peptostreptococcaceae;g__Terrisporobacter	0.01 ± 0.00	0.0	0.00 ± 0.00	0.0	3.94E-03	2.9732	-	4.13E-03
k__Bacteria;p__Firmicutes;c__Clostridia;o__Clostridiales;f__Pseudoflavonifractor;g__	0.00 ± 0.00	0.0	0.00 ± 0.00	0.0	1.04E-01	-	-	-
k__Bacteria;p__Firmicutes;c__Clostridia;o__Clostridiales;f__Pseudoflavonifractor;g__capillosus	0.00 ± 0.00	0.0	0.00 ± 0.00	0.0	3.94E-03	2.8732	-	4.13E-03
**k__Bacteria;p__Firmicutes;c__Clostridia;o__Clostridiales;f__Ruminococcaceae;g__**	**2.15 ± 0.29**	**59.0**	**0.00 ± 0.00**	**0.0**	**5.24E-29**	**4.0350**	**-**	**9.48E-29**
k__Bacteria;p__Firmicutes;c__Clostridia;o__Clostridiales;f__Ruminococcaceae;g__Acetanaerobacterium	0.00 ± 0.00	0.0	0.00 ± 0.00	0.0	3.94E-03	2.9350	-	4.13E-03
k__Bacteria;p__Firmicutes;c__Clostridia;o__Clostridiales;f__Ruminococcaceae;g__Acetivibrio	0.01 ± 0.00	0.0	0.00 ± 0.00	0.0	7.71E-04	2.6381	-	8.24E-04
k__Bacteria;p__Firmicutes;c__Clostridia;o__Clostridiales;f__Ruminococcaceae;g__Acutalibacter	0.00 ± 0.00	0.0	0.00 ± 0.00	0.0	3.55E-01	-	-	-
k__Bacteria;p__Firmicutes;c__Clostridia;o__Clostridiales;f__Ruminococcaceae;g__Anaerobacterium	0.00 ± 0.00	0.0	0.00 ± 0.00	0.0	1.16E-02	3.0336	-	1.20E-02
k__Bacteria;p__Firmicutes;c__Clostridia;o__Clostridiales;f__Ruminococcaceae;g__Anaerofilum	0.02 ± 0.00	0.0	0.00 ± 0.00	0.0	3.06E-08	2.4670	-	3.68E-08
k__Bacteria;p__Firmicutes;c__Clostridia;o__Clostridiales;f__Ruminococcaceae;g__Anaeromassilibacillus	0.00 ± 0.00	0.0	0.00 ± 0.00	0.0	NA	-	-	-
k__Bacteria;p__Firmicutes;c__Clostridia;o__Clostridiales;f__Ruminococcaceae;g__Anaerotruncus	0.01 ± 0.00	0.0	0.00 ± 0.00	0.0	7.32E-07	2.7348	-	8.48E-07
k__Bacteria;p__Firmicutes;c__Clostridia;o__Clostridiales;f__Ruminococcaceae;g__Caproiciproducens	0.00 ± 0.00	0.0	0.00 ± 0.00	0.0	6.75E-03	3.5987	-	7.04E-03
k__Bacteria;p__Firmicutes;c__Clostridia;o__Clostridiales;f__Ruminococcaceae;g__Clostridium	0.09 ± 0.02	1.2	0.00 ± 0.00	0.0	1.47E-12	2.7339	-	1.95E-12
k__Bacteria;p__Firmicutes;c__Clostridia;o__Clostridiales;f__Ruminococcaceae;g__Drancourtella	0.00 ± 0.00	0.0	0.00 ± 0.00	0.0	NA	-	-	-
k__Bacteria;p__Firmicutes;c__Clostridia;o__Clostridiales;f__Ruminococcaceae;g__Ethanoligenens	0.02 ± 0.01	0.0	0.00 ± 0.00	0.0	5.86E-08	2.2033	-	6.99E-08
k__Bacteria;p__Firmicutes;c__Clostridia;o__Clostridiales;f__Ruminococcaceae;g__Eubacterium	0.03 ± 0.01	1.2	0.00 ± 0.00	0.0	1.12E-04	2.5160	-	1.24E-04
k__Bacteria;p__Firmicutes;c__Clostridia;o__Clostridiales;f__Ruminococcaceae;g__Faecalibacterium	0.92 ± 0.25	20.5	0.03 ± 0.01	0.0	9.77E-14	3.6559	-	1.55E-13
k__Bacteria;p__Firmicutes;c__Clostridia;o__Clostridiales;f__Ruminococcaceae;g__Fastidiosipila	0.14 ± 0.03	1.2	0.00 ± 0.00	0.0	4.95E-21	2.9009	-	7.87E-21
k__Bacteria;p__Firmicutes;c__Clostridia;o__Clostridiales;f__Ruminococcaceae;g__Gemmiger	0.09 ± 0.03	2.4	0.00 ± 0.00	0.0	4.54E-08	2.7512	-	5.70E-08
k__Bacteria;p__Firmicutes;c__Clostridia;o__Clostridiales;f__Ruminococcaceae;g__Neglecta	0.00 ± 0.00	0.0	0.00 ± 0.00	0.0	1.33E-03	2.7729	-	1.41E-03
k__Bacteria;p__Firmicutes;c__Clostridia;o__Clostridiales;f__Ruminococcaceae;g__Papillibacter	0.00 ± 0.00	0.0	0.00 ± 0.00	0.0	7.71E-04	3.1236	-	8.24E-04
k__Bacteria;p__Firmicutes;c__Clostridia;o__Clostridiales;f__Ruminococcaceae;g__Phocea	0.00 ± 0.00	0.0	0.00 ± 0.00	0.0	NA	-	-	-
k__Bacteria;p__Firmicutes;c__Clostridia;o__Clostridiales;f__Ruminococcaceae;g__Ruminiclostridium	0.02 ± 0.00	0.0	0.00 ± 0.00	0.0	6.74E-13	3.0892	-	9.04E-13
k__Bacteria;p__Firmicutes;c__Clostridia;o__Clostridiales;f__Ruminococcaceae;g__Ruminococcus	0.28 ± 0.06	10.8	0.01 ± 0.01	0.0	2.02E-11	3.1453	-	2.88E-11
**k__Bacteria;p__Firmicutes;c__Clostridia;o__Clostridiales;f__Ruminococcaceae;g__Saccharofermentans**	**6.91 ± 0.70**	**66.3**	**0.55 ± 0.31**	**6.8**	**1.03E-16**	**4.4911**	**-**	**1.62E-16**
k__Bacteria;p__Firmicutes;c__Clostridia;o__Clostridiales;f__Ruminococcaceae;g__Sporobacter	0.17 ± 0.04	3.6	0.00 ± 0.00	0.0	2.74E-14	2.9446	-	3.79E-14
k__Bacteria;p__Firmicutes;c__Clostridia;o__Clostridiales;f__Ruminococcaceae;g__Subdoligranulum	0.00 ± 0.00	0.0	0.00 ± 0.00	0.0	4.44E-04	2.8483	-	4.78E-04
k__Bacteria;p__Firmicutes;c__Clostridia;o__Clostridiales;f__Syntrophomonadaceae;g__	0.00 ± 0.00	0.0	0.00 ± 0.00	0.0	NA	-	-	-
k__Bacteria;p__Firmicutes;c__Clostridia;o__Clostridiales;f__Syntrophomonadaceae;g__Pelospora	0.00 ± 0.00	0.0	0.00 ± 0.00	0.0	NA	-	-	-
k__Bacteria;p__Firmicutes;c__Clostridia;o__Clostridiales;f__Syntrophomonadaceae;g__Syntrophomonas	0.00 ± 0.00	0.0	0.00 ± 0.00	0.0	3.55E-01	-	-	-
k__Bacteria;p__Firmicutes;c__Clostridia;o__Thermoanaerobacterales;f__Thermoanaerobacteraceae;g__	0.00 ± 0.00	0.0	0.00 ± 0.00	0.0	NA	-	-	-
k__Bacteria;p__Firmicutes;c__Erysipelotrichia;o__Erysipelotrichales;f__Erysipelotrichaceae;g__	0.42 ± 0.07	13.3	0.00 ± 0.00	0.0	9.99E-25	3.3298	-	1.70E-24
k__Bacteria;p__Firmicutes;c__Erysipelotrichia;o__Erysipelotrichales;f__Erysipelotrichaceae;g__Bulleidia	0.02 ± 0.00	0.0	0.00 ± 0.00	0.0	1.11E-07	2.5133	-	1.32E-07
k__Bacteria;p__Firmicutes;c__Erysipelotrichia;o__Erysipelotrichales;f__Erysipelotrichaceae;g__Catenibacterium	0.20 ± 0.06	6.0	0.00 ± 0.00	0.0	2.23E-15	3.0441	-	3.15E-15
k__Bacteria;p__Firmicutes;c__Erysipelotrichia;o__Erysipelotrichales;f__Erysipelotrichaceae;g__Clostridium	0.07 ± 0.02	0.0	0.01 ± 0.00	0.0	9.73E-10	2.8477	-	1.30E-09
k__Bacteria;p__Firmicutes;c__Erysipelotrichia;o__Erysipelotrichales;f__Erysipelotrichaceae;g__Coprobacillus	0.00 ± 0.00	0.0	0.00 ± 0.00	0.0	NA	-	-	-
k__Bacteria;p__Firmicutes;c__Erysipelotrichia;o__Erysipelotrichales;f__Erysipelotrichaceae;g__Dielma	0.00 ± 0.00	0.0	0.00 ± 0.00	0.0	NA	-	-	-
k__Bacteria;p__Firmicutes;c__Erysipelotrichia;o__Erysipelotrichales;f__Erysipelotrichaceae;g__Eggerthia	0.00 ± 0.00	0.0	0.00 ± 0.00	0.0	NA	-	-	-
k__Bacteria;p__Firmicutes;c__Erysipelotrichia;o__Erysipelotrichales;f__Erysipelotrichaceae;g__Erysipelatoclostridium	0.00 ± 0.00	0.0	0.00 ± 0.00	0.0	3.55E-01	-	-	-
k__Bacteria;p__Firmicutes;c__Erysipelotrichia;o__Erysipelotrichales;f__Erysipelotrichaceae;g__Erysipelotrichaceae_UCG-004	0.00 ± 0.00	0.0	0.00 ± 0.00	0.0	1.86E-01	-	-	-
k__Bacteria;p__Firmicutes;c__Erysipelotrichia;o__Erysipelotrichales;f__Erysipelotrichaceae;g__Eubacterium	0.00 ± 0.00	0.0	0.00 ± 0.00	0.0	NA	-	-	-
k__Bacteria;p__Firmicutes;c__Erysipelotrichia;o__Erysipelotrichales;f__Erysipelotrichaceae;g__Faecalitalea	0.00 ± 0.00	0.0	0.00 ± 0.00	0.0	NA	-	-	-
k__Bacteria;p__Firmicutes;c__Erysipelotrichia;o__Erysipelotrichales;f__Erysipelotrichaceae;g__Holdemanella	0.01 ± 0.00	0.0	0.00 ± 0.00	0.0	7.26E-06	3.0500	-	8.32E-06
k__Bacteria;p__Firmicutes;c__Erysipelotrichia;o__Erysipelotrichales;f__Erysipelotrichaceae;g__Holdemania	0.00 ± 0.00	0.0	0.00 ± 0.00	0.0	5.91E-02	-	-	-
k__Bacteria;p__Firmicutes;c__Erysipelotrichia;o__Erysipelotrichales;f__Erysipelotrichaceae;g__Longibaculum	0.02 ± 0.01	0.0	0.00 ± 0.00	0.0	7.32E-07	2.6283	-	8.48E-07
k__Bacteria;p__Firmicutes;c__Erysipelotrichia;o__Erysipelotrichales;f__Erysipelotrichaceae;g__Longicatena	0.00 ± 0.00	0.0	0.00 ± 0.00	0.0	NA	-	-	-
k__Bacteria;p__Firmicutes;c__Erysipelotrichia;o__Erysipelotrichales;f__Erysipelotrichaceae;g__Massiliomicrobiota	0.00 ± 0.00	0.0	0.00 ± 0.00	0.0	NA	-	-	-
k__Bacteria;p__Firmicutes;c__Erysipelotrichia;o__Erysipelotrichales;f__Erysipelotrichaceae;g__Solobacterium	0.00 ± 0.00	0.0	0.00 ± 0.00	0.0	1.86E-01	-	-	-
k__Bacteria;p__Firmicutes;c__Erysipelotrichia;o__Erysipelotrichales;f__Erysipelotrichaceae;g__Turicibacter	0.00 ± 0.00	0.0	0.00 ± 0.00	0.0	NA	-	-	-
k__Bacteria;p__Firmicutes;c__Negativicutes;o__Acidaminococcales;f__Acidaminococcaceae;g__Acidaminococcus	0.00 ± 0.00	0.0	0.00 ± 0.00	0.0	1.33E-01	-	-	-
k__Bacteria;p__Firmicutes;c__Negativicutes;o__Acidaminococcales;f__Acidaminococcaceae;g__Phascolarctobacterium	0.62 ± 0.32	10.8	0.00 ± 0.00	0.0	3.61E-18	3.4773	-	5.58E-18

k__Bacteria;p__Firmicutes;c__Negativicutes;o__Acidaminococcales;f__Acidaminococcaceae;g__Succiniclasticum	0.00 ± 0.00	0.0	0.00 ± 0.00	0.0	NA	-	-	-
k__Bacteria;p__Firmicutes;c__Negativicutes;o__Selenomonadales;f__Selenomonadaceae;g__	0.35 ± 0.08	14.5	0.00 ± 0.00	0.0	1.25E-16	3.2610	-	1.84E-16
k__Bacteria;p__Firmicutes;c__Negativicutes;o__Selenomonadales;f__Selenomonadaceae;g__Megamonas	0.00 ± 0.00	0.0	0.00 ± 0.00	0.0	NA	-	-	-
k__Bacteria;p__Firmicutes;c__Negativicutes;o__Selenomonadales;f__Selenomonadaceae;g__Mitsuokella	0.00 ± 0.00	0.0	0.00 ± 0.00	0.0	6.54E-01	-	-	-
k__Bacteria;p__Firmicutes;c__Negativicutes;o__Selenomonadales;f__Selenomonadaceae;g__Pectinatus	0.00 ± 0.00	0.0	0.00 ± 0.00	0.0	NA	-	-	-
k__Bacteria;p__Firmicutes;c__Negativicutes;o__Selenomonadales;f__Selenomonadaceae;g__Propionispira	0.48 ± 0.15	13.3	0.00 ± 0.00	0.0	2.57E-19	3.3996	-	3.95E-19
k__Bacteria;p__Firmicutes;c__Negativicutes;o__Selenomonadales;f__Selenomonadaceae;g__Schwartzia	0.00 ± 0.00	0.0	0.00 ± 0.00	0.0	NA	-	-	-
**k__Bacteria;p__Firmicutes;c__Negativicutes;o__Selenomonadales;f__Selenomonadaceae;g__Selenomonas**	**1.01 ± 0.15**	**33.7**	**0.00 ± 0.00**	**0.0**	**1.74E-28**	**3.6912**	**-**	**3.15E-28**
k__Bacteria;p__Firmicutes;c__Negativicutes;o__Veillonellales;f__Veillonellaceae;g__Allisonella	0.00 ± 0.00	0.0	0.00 ± 0.00	0.0	5.91E-02	-	-	-
k__Bacteria;p__Firmicutes;c__Negativicutes;o__Veillonellales;f__Veillonellaceae;g__Anaeroglobus	0.00 ± 0.00	0.0	0.07 ± 0.06	1.4	6.41E-02	-	-	-
**k__Bacteria;p__Firmicutes;c__Negativicutes;o__Veillonellales;f__Veillonellaceae;g__Dialister**	**5.53 ± 0.61**	**84.3**	**0.47 ± 0.15**	**12.3**	**1.18E-21**	**4.3828**	**-**	**2.03E-21**
k__Bacteria;p__Firmicutes;c__Negativicutes;o__Veillonellales;f__Veillonellaceae;g__Megasphaera	0.15 ± 0.12	1.2	0.86 ± 0.35	8.2	1.10E-01	-	-	-
k__Bacteria;p__Firmicutes;c__Negativicutes;o__Veillonellales;f__Veillonellaceae;g__Veillonella	0.01 ± 0.00	0.0	0.19 ± 0.12	4.1	9.56E-01	-	-	-
**k__Bacteria;p__Firmicutes;c__Tissierellia;o__Tissierellales;f__Peptoniphilaceae;g__**	**2.25 ± 0.28**	**60.2**	**0.08 ± 0.04**	**4.1**	**1.56E-24**	**4.0407**	**-**	**2.72E-24**
**k__Bacteria;p__Firmicutes;c__Tissierellia;o__Tissierellales;f__Peptoniphilaceae;g__Anaerococcus**	**1.95 ± 0.59**	**27.7**	**1.20 ± 0.50**	**13.7**	**1.24E-04**	**3.7581**	**-**	**1.34E-04**
k__Bacteria;p__Firmicutes;c__Tissierellia;o__Tissierellales;f__Peptoniphilaceae;g__Anaerosphaera	0.22 ± 0.09	6.0	0.00 ± 0.00	0.0	1.59E-08	3.0572	-	1.92E-08
k__Bacteria;p__Firmicutes;c__Tissierellia;o__Tissierellales;f__Peptoniphilaceae;g__Finegoldia	0.51 ± 0.21	8.4	0.55 ± 0.13	12.3	7.67E-02	-	-	-
k__Bacteria;p__Firmicutes;c__Tissierellia;o__Tissierellales;f__Peptoniphilaceae;g__Gallicola	0.00 ± 0.00	0.0	0.00 ± 0.00	0.0	3.55E-01	-	-	-
k__Bacteria;p__Firmicutes;c__Tissierellia;o__Tissierellales;f__Peptoniphilaceae;g__Helcococcus	0.22 ± 0.06	7.2	0.00 ± 0.00	0.0	3.92E-17	3.0723	-	5.87E-17
k__Bacteria;p__Firmicutes;c__Tissierellia;o__Tissierellales;f__Peptoniphilaceae;g__Parvimonas	1.00 ± 0.15	32.5	0.08 ± 0.04	2.7	9.89E-15	3.6899	-	1.50E-14
**k__Bacteria;p__Firmicutes;c__Tissierellia;o__Tissierellales;f__Peptoniphilaceae;g__Peptoniphilus**	**1.97 ± 0.25**	**55.4**	**0.70 ± 0.20**	**16.4**	**3.06E-13**	**3.7732**	**-**	**4.85E-13**
k__Bacteria;p__Firmicutes;c__Tissierellia;o__Tissierellales;f__Tissierellaceae;g__	0.21 ± 0.11	3.6	0.00 ± 0.00	0.0	1.06E-09	3.0969	-	1.32E-09
k__Bacteria;p__Firmicutes;c__Tissierellia;o__Tissierellales;f__Tissierellaceae;g__Tissierella	0.03 ± 0.01	0.0	0.00 ± 0.00	0.0	5.29E-10	2.6156	-	6.63E-10
k__Bacteria;p__Fusobacteria;c__Fusobacteriia;o__Fusobacteriales;f__Fusobacteriaceae;g__Cetobacterium	0.01 ± 0.01	0.0	0.00 ± 0.00	0.0	1.86E-01	-	-	-
**k__Bacteria;p__Fusobacteria;c__Fusobacteriia;o__Fusobacteriales;f__Fusobacteriaceae;g__Fusobacterium**	**5.35 ± 0.89**	**56.6**	**0.08 ± 0.05**	**2.7**	**4.02E-19**	**4.4177**	**-**	**6.52E-19**
k__Bacteria;p__Fusobacteria;c__Fusobacteriia;o__Fusobacteriales;f__Leptotrichiaceae;g__Leptotrichia	0.00 ± 0.00	0.0	0.00 ± 0.00	0.0	9.58E-01	-	-	-
**k__Bacteria;p__Fusobacteria;c__Fusobacteriia;o__Fusobacteriales;f__Leptotrichiaceae;g__Sneathia**	**4.98 ± 0.66**	**67.5**	**0.19 ± 0.13**	**2.7**	**8.96E-21**	**4.3698**	**-**	**1.48E-20**
k__Bacteria;p__Fusobacteria;c__Fusobacteriia;o__Fusobacteriales;f__Leptotrichiaceae;g__Streptobacillus	0.00 ± 0.00	0.0	0.00 ± 0.00	0.0	1.04E-01	-	-	-
k__Bacteria;p__Gemmatimonadetes;c__;o__;f__;g__	0.00 ± 0.00	0.0	0.00 ± 0.00	0.0	3.55E-01	-	-	-
k__Bacteria;p__Gemmatimonadetes;c__Gemmatimonadetes;o__Gemmatimonadales;f__Gemmatimonadaceae;g__Gemmatirosa	0.00 ± 0.00	0.0	0.00 ± 0.00	0.0	1.86E-01	-	-	-
k__Bacteria;p__Gracilibacteria;c__;o__;f__;g__	0.00 ± 0.00	0.0	0.00 ± 0.00	0.0	NA	-	-	-
k__Bacteria;p__Lentisphaerae;c__Lentisphaeria;o__Victivallales;f__;g__	0.00 ± 0.00	0.0	0.00 ± 0.00	0.0	6.75E-03	2.6475	-	7.04E-03
k__Bacteria;p__Lentisphaerae;c__Lentisphaeria;o__Victivallales;f__Victivallaceae;g__	0.00 ± 0.00	0.0	0.00 ± 0.00	0.0	1.98E-02	2.9569	-	2.04E-02
k__Bacteria;p__Lentisphaerae;c__Lentisphaeria;o__Victivallales;f__Victivallaceae;g__Victivallis	0.00 ± 0.00	0.0	0.00 ± 0.00	0.0	NA	-	-	-
k__Bacteria;p__Lentisphaerae;c__Oligosphaeria;o__Oligosphaerales;f__Oligosphaeraceae;g__	0.00 ± 0.00	0.0	0.00 ± 0.00	0.0	3.41E-02	2.6689	-	3.49E-02
k__Bacteria;p__Planctomycetes;c__Planctomycetia;o__Planctomycetales;f__Gemmataceae;g__	0.00 ± 0.00	0.0	0.00 ± 0.00	0.0	NA	-	-	-
k__Bacteria;p__Planctomycetes;c__Planctomycetia;o__Planctomycetales;f__Isosphaeraceae;g__Aquisphaera	0.00 ± 0.00	0.0	0.00 ± 0.00	0.0	3.55E-01	-	-	-
k__Bacteria;p__Proteobacteria;c__Alphaproteobacteria;o__Caulobacterales;f__Caulobacteraceae;g__	0.00 ± 0.00	0.0	0.00 ± 0.00	0.0	NA	-	-	-
k__Bacteria;p__Proteobacteria;c__Alphaproteobacteria;o__Caulobacterales;f__Caulobacteraceae;g__Brevundimonas	0.02 ± 0.01	0.0	0.01 ± 0.00	0.0	3.27E-01	-	-	-
k__Bacteria;p__Proteobacteria;c__Alphaproteobacteria;o__Caulobacterales;f__Caulobacteraceae;g__Caulobacter	0.00 ± 0.00	0.0	0.00 ± 0.00	0.0	3.55E-01	-	-	-
k__Bacteria;p__Proteobacteria;c__Alphaproteobacteria;o__Caulobacterales;f__Caulobacteraceae;g__Phenylobacterium	0.00 ± 0.00	0.0	0.00 ± 0.00	0.0	2.92E-01	-	-	-
k__Bacteria;p__Proteobacteria;c__Alphaproteobacteria;o__Rhizobiales;f__;g__	0.00 ± 0.00	0.0	0.00 ± 0.00	0.0	NA	-	-	-
k__Bacteria;p__Proteobacteria;c__Alphaproteobacteria;o__Rhizobiales;f__Aurantimonadaceae;g__Aureimonas	0.00 ± 0.00	0.0	0.00 ± 0.00	0.0	3.55E-01	-	-	-
k__Bacteria;p__Proteobacteria;c__Alphaproteobacteria;o__Rhizobiales;f__Beijerinckiaceae;g__Camelimonas	0.00 ± 0.00	0.0	0.00 ± 0.00	0.0	NA	-	-	-
k__Bacteria;p__Proteobacteria;c__Alphaproteobacteria;o__Rhizobiales;f__Bradyrhizobiaceae;g__Bosea	0.00 ± 0.00	0.0	0.00 ± 0.00	0.0	8.65E-01	-	-	-
k__Bacteria;p__Proteobacteria;c__Alphaproteobacteria;o__Rhizobiales;f__Bradyrhizobiaceae;g__Bradyrhizobium	0.00 ± 0.00	0.0	0.02 ± 0.01	0.0	3.41E-02	-	3.1096	3.20E-02
k__Bacteria;p__Proteobacteria;c__Alphaproteobacteria;o__Rhizobiales;f__Brucellaceae;g__Pseudochrobactrum	0.00 ± 0.00	0.0	0.00 ± 0.00	0.0	1.86E-01	-	-	-
k__Bacteria;p__Proteobacteria;c__Alphaproteobacteria;o__Rhizobiales;f__Hyphomicrobiaceae;g__Devosia	0.00 ± 0.00	0.0	0.00 ± 0.00	0.0	1.04E-01	-	-	-
k__Bacteria;p__Proteobacteria;c__Alphaproteobacteria;o__Rhizobiales;f__Hyphomicrobiaceae;g__Hyphomicrobium	0.00 ± 0.00	0.0	0.00 ± 0.00	0.0	3.55E-01	-	-	-
k__Bacteria;p__Proteobacteria;c__Alphaproteobacteria;o__Rhizobiales;f__Hyphomicrobiaceae;g__Rhodomicrobium	0.00 ± 0.00	0.0	0.00 ± 0.00	0.0	NA	-	-	-
k__Bacteria;p__Proteobacteria;c__Alphaproteobacteria;o__Rhizobiales;f__Methylobacteriaceae;g__Methylobacterium	0.00 ± 0.00	0.0	0.00 ± 0.00	0.0	3.42E-03	3.2433	-	3.65E-03
k__Bacteria;p__Proteobacteria;c__Alphaproteobacteria;o__Rhizobiales;f__Methylocystaceae;g__Methylosinus	0.00 ± 0.00	0.0	0.00 ± 0.00	0.0	NA	-	-	-
k__Bacteria;p__Proteobacteria;c__Alphaproteobacteria;o__Rhizobiales;f__Phyllobacteriaceae;g__Mesorhizobium	0.00 ± 0.00	0.0	0.00 ± 0.00	0.0	3.55E-01	-	-	-
k__Bacteria;p__Proteobacteria;c__Alphaproteobacteria;o__Rhizobiales;f__Rhizobiaceae;g__	0.00 ± 0.00	0.0	0.00 ± 0.00	0.0	NA	-	-	-
k__Bacteria;p__Proteobacteria;c__Alphaproteobacteria;o__Rhizobiales;f__Rhizobiaceae;g__Rhizobium	0.00 ± 0.00	0.0	0.00 ± 0.00	0.0	NA	-	-	-
k__Bacteria;p__Proteobacteria;c__Alphaproteobacteria;o__Rhodobacterales;f__Hyphomonadaceae;g__	0.00 ± 0.00	0.0	0.00 ± 0.00	0.0	NA	-	-	-
k__Bacteria;p__Proteobacteria;c__Alphaproteobacteria;o__Rhodobacterales;f__Rhodobacteraceae;g__	0.00 ± 0.00	0.0	0.00 ± 0.00	0.0	1.04E-01	-	-	-
k__Bacteria;p__Proteobacteria;c__Alphaproteobacteria;o__Rhodobacterales;f__Rhodobacteraceae;g__Amaricoccus	0.05 ± 0.05	1.2	0.00 ± 0.00	0.0	3.55E-01	-	-	-
k__Bacteria;p__Proteobacteria;c__Alphaproteobacteria;o__Rhodobacterales;f__Rhodobacteraceae;g__Aquimixticola	0.01 ± 0.01	0.0	0.00 ± 0.00	0.0	1.04E-01	-	-	-
k__Bacteria;p__Proteobacteria;c__Alphaproteobacteria;o__Rhodobacterales;f__Rhodobacteraceae;g__Gemmobacter	0.00 ± 0.00	0.0	0.00 ± 0.00	0.0	1.04E-01	-	-	-
k__Bacteria;p__Proteobacteria;c__Alphaproteobacteria;o__Rhodobacterales;f__Rhodobacteraceae;g__Paracoccus	0.01 ± 0.01	0.0	0.00 ± 0.00	0.0	5.91E-02	-	-	-
k__Bacteria;p__Proteobacteria;c__Alphaproteobacteria;o__Rhodobacterales;f__Rhodobacteraceae;g__Rhodobacter	0.00 ± 0.00	0.0	0.00 ± 0.00	0.0	NA	-	-	-
k__Bacteria;p__Proteobacteria;c__Alphaproteobacteria;o__Rhodobacterales;f__Rhodobacteraceae;g__Rubellimicrobium	0.00 ± 0.00	0.0	0.00 ± 0.00	0.0	3.55E-01	-	-	-

k__Bacteria;p__Proteobacteria;c__Alphaproteobacteria;o__Rhodospirillales;f__Acetobacteraceae;g__	0.00 ± 0.00	0.0	0.00 ± 0.00	0.0	1.16E-02	2.7787	-	1.20E-02
k__Bacteria;p__Proteobacteria;c__Alphaproteobacteria;o__Rhodospirillales;f__Acetobacteraceae;g__Acidocella	0.00 ± 0.00	0.0	0.00 ± 0.00	0.0	NA	-	-	-
k__Bacteria;p__Proteobacteria;c__Alphaproteobacteria;o__Rhodospirillales;f__Acetobacteraceae;g__Belnapia	0.00 ± 0.00	0.0	0.00 ± 0.00	0.0	3.55E-01	-	-	-
k__Bacteria;p__Proteobacteria;c__Alphaproteobacteria;o__Rhodospirillales;f__Acetobacteraceae;g__Gluconobacter	0.00 ± 0.00	0.0	0.00 ± 0.00	0.0	NA	-	-	-
k__Bacteria;p__Proteobacteria;c__Alphaproteobacteria;o__Rhodospirillales;f__Acetobacteraceae;g__Paracraurococcus	0.00 ± 0.00	0.0	0.00 ± 0.00	0.0	NA	-	-	-
k__Bacteria;p__Proteobacteria;c__Alphaproteobacteria;o__Rhodospirillales;f__Acetobacteraceae;g__Rhodovastum	0.00 ± 0.00	0.0	0.00 ± 0.00	0.0	3.55E-01	-	-	-
k__Bacteria;p__Proteobacteria;c__Alphaproteobacteria;o__Rhodospirillales;f__Acetobacteraceae;g__Roseomonas	0.00 ± 0.00	0.0	0.01 ± 0.01	0.0	2.92E-01	-	-	-
k__Bacteria;p__Proteobacteria;c__Alphaproteobacteria;o__Rhodospirillales;f__Rhodospirillaceae;g__	0.07 ± 0.03	1.2	0.00 ± 0.00	0.0	1.89E-07	2.5737	-	2.26E-07
k__Bacteria;p__Proteobacteria;c__Alphaproteobacteria;o__Rhodospirillales;f__Rhodospirillaceae;g__Ferrovibrio	0.00 ± 0.00	0.0	0.00 ± 0.00	0.0	3.55E-01	-	-	-
k__Bacteria;p__Proteobacteria;c__Alphaproteobacteria;o__Rhodospirillales;f__Rhodospirillaceae;g__Lacibacterium	0.00 ± 0.00	0.0	0.00 ± 0.00	0.0	2.92E-01	-	-	-
k__Bacteria;p__Proteobacteria;c__Alphaproteobacteria;o__Rhodospirillales;f__Rhodospirillaceae;g__Niveispirillum	0.00 ± 0.00	0.0	0.00 ± 0.00	0.0	3.55E-01	-	-	-
k__Bacteria;p__Proteobacteria;c__Alphaproteobacteria;o__Rhodospirillales;f__Rhodospirillaceae;g__Tistrella	0.00 ± 0.00	0.0	0.00 ± 0.00	0.0	1.04E-01	-	-	-
k__Bacteria;p__Proteobacteria;c__Alphaproteobacteria;o__Rickettsiales;f__;g__	0.00 ± 0.00	0.0	0.00 ± 0.00	0.0	3.55E-01	-	-	-
k__Bacteria;p__Proteobacteria;c__Alphaproteobacteria;o__Sphingomonadales;f__Erythrobacteraceae;g__Altererythrobacter	0.00 ± 0.00	0.0	0.00 ± 0.00	0.0	NA	-	-	-
k__Bacteria;p__Proteobacteria;c__Alphaproteobacteria;o__Sphingomonadales;f__Sphingomonadaceae;g__	0.00 ± 0.00	0.0	0.00 ± 0.00	0.0	1.86E-01	-	-	-
k__Bacteria;p__Proteobacteria;c__Alphaproteobacteria;o__Sphingomonadales;f__Sphingomonadaceae;g__Novosphingobium	0.00 ± 0.00	0.0	0.00 ± 0.00	0.0	1.98E-02	3.2111	-	2.04E-02
k__Bacteria;p__Proteobacteria;c__Alphaproteobacteria;o__Sphingomonadales;f__Sphingomonadaceae;g__Sphingobium	0.00 ± 0.00	0.0	0.02 ± 0.01	0.0	2.49E-03	-	2.9165	2.25E-03
k__Bacteria;p__Proteobacteria;c__Alphaproteobacteria;o__Sphingomonadales;f__Sphingomonadaceae;g__Sphingomonas	0.00 ± 0.00	0.0	0.01 ± 0.00	0.0	9.13E-01	-	-	-
k__Bacteria;p__Proteobacteria;c__Alphaproteobacteria;o__Sphingomonadales;f__Sphingomonadaceae;g__Sphingopyxis	0.00 ± 0.00	0.0	0.02 ± 0.01	0.0	6.89E-03	-	2.6686	6.28E-03
k__Bacteria;p__Proteobacteria;c__Alphaproteobacteria;o__Sphingomonadales;f__Sphingomonadaceae;g__Sphingorhabdus	0.00 ± 0.00	0.0	0.00 ± 0.00	0.0	3.55E-01	-	-	-
k__Bacteria;p__Proteobacteria;c__Betaproteobacteria;o__;f__;g__	0.00 ± 0.00	0.0	0.00 ± 0.00	0.0	NA	-	-	-
k__Bacteria;p__Proteobacteria;c__Betaproteobacteria;o__Burkholderiales;f__Alcaligenaceae;g__Achromobacter	0.00 ± 0.00	0.0	0.28 ± 0.22	4.1	4.59E-02	-	3.2102	4.22E-02
k__Bacteria;p__Proteobacteria;c__Betaproteobacteria;o__Burkholderiales;f__Alcaligenaceae;g__Alcaligenes	0.00 ± 0.00	0.0	0.00 ± 0.00	0.0	1.16E-02	3.2080	-	1.20E-02
k__Bacteria;p__Proteobacteria;c__Betaproteobacteria;o__Burkholderiales;f__Alcaligenaceae;g__Derxia	0.00 ± 0.00	0.0	0.00 ± 0.00	0.0	NA	-	-	-
k__Bacteria;p__Proteobacteria;c__Betaproteobacteria;o__Burkholderiales;f__Alcaligenaceae;g__Parapusillimonas	0.00 ± 0.00	0.0	0.00 ± 0.00	0.0	3.55E-01	-	-	-
k__Bacteria;p__Proteobacteria;c__Betaproteobacteria;o__Burkholderiales;f__Alcaligenaceae;g__Pelistega	0.02 ± 0.02	1.2	0.00 ± 0.00	0.0	5.91E-02	-	-	-
k__Bacteria;p__Proteobacteria;c__Betaproteobacteria;o__Burkholderiales;f__Burkholderiaceae;g__Burkholderia	0.00 ± 0.00	0.0	0.45 ± 0.16	9.6	4.26E-05	-	3.3758	3.51E-05
k__Bacteria;p__Proteobacteria;c__Betaproteobacteria;o__Burkholderiales;f__Burkholderiaceae;g__Cupriavidus	0.00 ± 0.00	0.0	0.01 ± 0.00	0.0	3.41E-02	-	2.7361	3.20E-02
k__Bacteria;p__Proteobacteria;c__Betaproteobacteria;o__Burkholderiales;f__Burkholderiaceae;g__Lautropia	0.00 ± 0.00	0.0	0.00 ± 0.00	0.0	NA	-	-	-
k__Bacteria;p__Proteobacteria;c__Betaproteobacteria;o__Burkholderiales;f__Burkholderiaceae;g__Limnobacter	0.00 ± 0.00	0.0	0.00 ± 0.00	0.0	3.55E-01	-	-	-
k__Bacteria;p__Proteobacteria;c__Betaproteobacteria;o__Burkholderiales;f__Burkholderiaceae;g__Paraburkholderia	0.00 ± 0.00	0.0	0.00 ± 0.00	0.0	1.04E-01	-	-	-
k__Bacteria;p__Proteobacteria;c__Betaproteobacteria;o__Burkholderiales;f__Burkholderiaceae;g__Ralstonia	0.00 ± 0.00	0.0	0.00 ± 0.00	0.0	2.06E-01	-	-	-
k__Bacteria;p__Proteobacteria;c__Betaproteobacteria;o__Burkholderiales;f__Comamonadaceae;g__	0.01 ± 0.00	0.0	0.01 ± 0.00	0.0	1.44E-02	2.7579	-	1.57E-02
k__Bacteria;p__Proteobacteria;c__Betaproteobacteria;o__Burkholderiales;f__Comamonadaceae;g__Comamonas	0.01 ± 0.00	0.0	0.01 ± 0.01	0.0	2.35E-01	-	-	-
k__Bacteria;p__Proteobacteria;c__Betaproteobacteria;o__Burkholderiales;f__Comamonadaceae;g__Delftia	0.00 ± 0.00	0.0	0.00 ± 0.00	0.0	1.33E-01	-	-	-
k__Bacteria;p__Proteobacteria;c__Betaproteobacteria;o__Burkholderiales;f__Comamonadaceae;g__Pelomonas	0.00 ± 0.00	0.0	0.00 ± 0.00	0.0	7.58E-01	-	-	-
k__Bacteria;p__Proteobacteria;c__Betaproteobacteria;o__Burkholderiales;f__Oxalobacteraceae;g__Duganella	0.00 ± 0.00	0.0	0.00 ± 0.00	0.0	NA	-	-	-
k__Bacteria;p__Proteobacteria;c__Betaproteobacteria;o__Burkholderiales;f__Oxalobacteraceae;g__Herbaspirillum	0.01 ± 0.00	0.0	0.44 ± 0.15	12.3	2.76E-01	-	-	-
k__Bacteria;p__Proteobacteria;c__Betaproteobacteria;o__Burkholderiales;f__Oxalobacteraceae;g__Janthinobacterium	0.00 ± 0.00	0.0	0.00 ± 0.00	0.0	1.04E-01	-	-	-
k__Bacteria;p__Proteobacteria;c__Betaproteobacteria;o__Burkholderiales;f__Oxalobacteraceae;g__Massilia	0.00 ± 0.00	0.0	0.00 ± 0.00	0.0	3.55E-01	-	-	-
k__Bacteria;p__Proteobacteria;c__Betaproteobacteria;o__Burkholderiales;f__Roseateles;g__	0.04 ± 0.02	1.2	0.00 ± 0.00	0.0	1.47E-05	2.3838	-	1.65E-05
k__Bacteria;p__Proteobacteria;c__Betaproteobacteria;o__Burkholderiales;f__Roseateles;g__aquatilis	0.00 ± 0.00	0.0	0.00 ± 0.00	0.0	NA	-	-	-
k__Bacteria;p__Proteobacteria;c__Betaproteobacteria;o__Burkholderiales;f__Rubrivivax;g__gelatinosus	0.03 ± 0.02	1.2	0.00 ± 0.00	0.0	5.91E-02	-	-	-
k__Bacteria;p__Proteobacteria;c__Betaproteobacteria;o__Burkholderiales;f__Sutterellaceae;g__Parasutterella	0.00 ± 0.00	0.0	0.00 ± 0.00	0.0	2.92E-01	-	-	-
k__Bacteria;p__Proteobacteria;c__Betaproteobacteria;o__Burkholderiales;f__Sutterellaceae;g__Sutterella	0.01 ± 0.01	0.0	0.03 ± 0.03	1.4	3.50E-03	-	3.1579	3.86E-03
k__Bacteria;p__Proteobacteria;c__Betaproteobacteria;o__Burkholderiales;f__Tepidimonas;g__fonticaldi	0.00 ± 0.00	0.0	0.00 ± 0.00	0.0	NA	-	-	-
k__Bacteria;p__Proteobacteria;c__Betaproteobacteria;o__Hydrogenophilales;f__Hydrogenophilaceae;g__	0.00 ± 0.00	0.0	0.00 ± 0.00	0.0	NA	-	-	-
k__Bacteria;p__Proteobacteria;c__Betaproteobacteria;o__Methylophilales;f__Methylophilaceae;g__	0.00 ± 0.00	0.0	0.00 ± 0.00	0.0	3.55E-01	-	-	-
k__Bacteria;p__Proteobacteria;c__Betaproteobacteria;o__Neisseriales;f__Neisseriaceae;g__	0.00 ± 0.00	0.0	0.00 ± 0.00	0.0	3.41E-02	3.3822	-	3.49E-02
k__Bacteria;p__Proteobacteria;c__Betaproteobacteria;o__Neisseriales;f__Neisseriaceae;g__Alysiella	0.00 ± 0.00	0.0	0.00 ± 0.00	0.0	NA	-	-	-
k__Bacteria;p__Proteobacteria;c__Betaproteobacteria;o__Neisseriales;f__Neisseriaceae;g__Eikenella	0.00 ± 0.00	0.0	0.00 ± 0.00	0.0	NA	-	-	-
k__Bacteria;p__Proteobacteria;c__Betaproteobacteria;o__Neisseriales;f__Neisseriaceae;g__Kingella	0.00 ± 0.00	0.0	0.00 ± 0.00	0.0	NA	-	-	-
k__Bacteria;p__Proteobacteria;c__Betaproteobacteria;o__Neisseriales;f__Neisseriaceae;g__Neisseria	0.00 ± 0.00	0.0	0.00 ± 0.00	0.0	2.48E-01	-	-	-
k__Bacteria;p__Proteobacteria;c__Betaproteobacteria;o__Neisseriales;f__Neisseriaceae;g__Simonsiella	0.00 ± 0.00	0.0	0.00 ± 0.00	0.0	NA	-	-	-
k__Bacteria;p__Proteobacteria;c__Betaproteobacteria;o__Neisseriales;f__Neisseriaceae;g__Snodgrassella	0.00 ± 0.00	0.0	0.06 ± 0.04	2.7	6.41E-02	-	-	-
k__Bacteria;p__Proteobacteria;c__Betaproteobacteria;o__Rhodocyclales;f__Rhodocyclaceae;g__Azoarcus	0.00 ± 0.00	0.0	0.00 ± 0.00	0.0	3.55E-01	-	-	-
k__Bacteria;p__Proteobacteria;c__Betaproteobacteria;o__Rhodocyclales;f__Rhodocyclaceae;g__Propionivibrio	0.00 ± 0.00	0.0	0.00 ± 0.00	0.0	NA	-	-	-
k__Bacteria;p__Proteobacteria;c__Deltaproteobacteria;o__Bradymonadales;f__;g__	0.03 ± 0.01	1.2	0.00 ± 0.00	0.0	1.47E-05	2.2177	-	1.65E-05
k__Bacteria;p__Proteobacteria;c__Deltaproteobacteria;o__Desulfobacterales;f__Desulfobacteraceae;g__	0.00 ± 0.00	0.0	0.00 ± 0.00	0.0	NA	-	-	-
k__Bacteria;p__Proteobacteria;c__Deltaproteobacteria;o__Desulfobacterales;f__Desulfobulbaceae;g__Desulfobulbus	0.00 ± 0.00	0.0	0.00 ± 0.00	0.0	NA	-	-	-
k__Bacteria;p__Proteobacteria;c__Deltaproteobacteria;o__Desulfovibrionales;f__;g__	0.00 ± 0.00	0.0	0.00 ± 0.00	0.0	3.55E-01	-	-	-
k__Bacteria;p__Proteobacteria;c__Deltaproteobacteria;o__Desulfovibrionales;f__Desulfohalobiaceae;g__	0.00 ± 0.00	0.0	0.00 ± 0.00	0.0	1.86E-01	-	-	-
k__Bacteria;p__Proteobacteria;c__Deltaproteobacteria;o__Desulfovibrionales;f__Desulfomicrobiaceae;g__Desulfomicrobium	0.00 ± 0.00	0.0	0.00 ± 0.00	0.0	NA	-	-	-

k__Bacteria;p__Proteobacteria;c__Deltaproteobacteria;o__Desulfovibrionales;f__Desulfovibrionaceae;g__	0.00 ± 0.00	0.0	0.00 ± 0.00	0.0	1.98E-02	3.4246	-	2.04E-02
k__Bacteria;p__Proteobacteria;c__Deltaproteobacteria;o__Desulfovibrionales;f__Desulfovibrionaceae;g__Desulfovibrio	0.04 ± 0.02	1.2	0.00 ± 0.00	0.0	3.06E-08	2.4538	-	3.68E-08
k__Bacteria;p__Proteobacteria;c__Deltaproteobacteria;o__Desulfuromonadales;f__;g__	0.00 ± 0.00	0.0	0.00 ± 0.00	0.0	NA	-	-	-
k__Bacteria;p__Proteobacteria;c__Deltaproteobacteria;o__Desulfuromonadales;f__Desulfuromonadaceae;g__	0.00 ± 0.00	0.0	0.00 ± 0.00	0.0	NA	-	-	-
k__Bacteria;p__Proteobacteria;c__Deltaproteobacteria;o__Desulfuromonadales;f__Geobacteraceae;g__Geobacter	0.00 ± 0.00	0.0	0.00 ± 0.00	0.0	3.41E-02	3.7311	-	3.49E-02
k__Bacteria;p__Proteobacteria;c__Deltaproteobacteria;o__Myxococcales;f__Labilitrichaceae;g__Labilithrix	0.00 ± 0.00	0.0	0.00 ± 0.00	0.0	NA	-	-	-
k__Bacteria;p__Proteobacteria;c__Deltaproteobacteria;o__Myxococcales;f__Phaselicystidaceae;g__Phaselicystis	0.00 ± 0.00	0.0	0.00 ± 0.00	0.0	3.55E-01	-	-	-
k__Bacteria;p__Proteobacteria;c__Epsilonproteobacteria;o__Campylobacterales;f__Campylobacteraceae;g__	0.00 ± 0.00	0.0	0.00 ± 0.00	0.0	3.55E-01	-	-	-
k__Bacteria;p__Proteobacteria;c__Epsilonproteobacteria;o__Campylobacterales;f__Campylobacteraceae;g__Arcobacter	0.00 ± 0.00	0.0	0.00 ± 0.00	0.0	1.86E-01	-	-	-
**k__Bacteria;p__Proteobacteria;c__Epsilonproteobacteria;o__Campylobacterales;f__Campylobacteraceae;g__Campylobacter**	**8.47 ± 0.75**	**85.5**	**0.28 ± 0.16**	**5.5**	**8.52E-25**	**4.6046**	**-**	**1.48E-24**
k__Bacteria;p__Proteobacteria;c__Epsilonproteobacteria;o__Campylobacterales;f__Campylobacteraceae;g__Sulfurospirillum	0.00 ± 0.00	0.0	0.00 ± 0.00	0.0	NA	-	-	-
k__Bacteria;p__Proteobacteria;c__Epsilonproteobacteria;o__Campylobacterales;f__Helicobacteraceae;g__	0.00 ± 0.00	0.0	0.00 ± 0.00	0.0	NA	-	-	-
k__Bacteria;p__Proteobacteria;c__Epsilonproteobacteria;o__Campylobacterales;f__Helicobacteraceae;g__Helicobacter	0.48 ± 0.14	10.8	0.00 ± 0.00	0.0	4.95E-21	3.3922	-	7.87E-21
k__Bacteria;p__Proteobacteria;c__Epsilonproteobacteria;o__Campylobacterales;f__Helicobacteraceae;g__Wolinella	0.00 ± 0.00	0.0	0.00 ± 0.00	0.0	NA	-	-	-
k__Bacteria;p__Proteobacteria;c__Gammaproteobacteria;o__;f__;g__	0.05 ± 0.02	1.2	0.00 ± 0.00	0.0	1.06E-09	2.3953	-	1.32E-09
k__Bacteria;p__Proteobacteria;c__Gammaproteobacteria;o__Aeromonadales;f__Aeromonadaceae;g__Aeromonas	0.00 ± 0.00	0.0	0.00 ± 0.00	0.0	3.55E-01	-	-	-
k__Bacteria;p__Proteobacteria;c__Gammaproteobacteria;o__Aeromonadales;f__Succinivibrionaceae;g__Succinivibrio	0.25 ± 0.07	6.0	0.00 ± 0.00	0.0	3.07E-13	3.1329	-	4.15E-13
k__Bacteria;p__Proteobacteria;c__Gammaproteobacteria;o__Alteromonadales;f__Alteromonadaceae;g__Marinobacter	0.00 ± 0.00	0.0	0.00 ± 0.00	0.0	6.75E-03	3.6324	-	7.04E-03
k__Bacteria;p__Proteobacteria;c__Gammaproteobacteria;o__Alteromonadales;f__Idiomarinaceae;g__Aliidiomarina	0.01 ± 0.00	0.0	0.00 ± 0.00	0.0	3.94E-03	3.4153	-	4.13E-03
k__Bacteria;p__Proteobacteria;c__Gammaproteobacteria;o__Cardiobacteriales;f__Cardiobacteriaceae;g__Cardiobacterium	0.00 ± 0.00	0.0	0.00 ± 0.00	0.0	NA	-	-	-
k__Bacteria;p__Proteobacteria;c__Gammaproteobacteria;o__Cardiobacteriales;f__Cardiobacteriaceae;g__Suttonella	0.02 ± 0.02	1.2	0.00 ± 0.00	0.0	1.04E-01	-	-	-
k__Bacteria;p__Proteobacteria;c__Gammaproteobacteria;o__Chromatiales;f__Chromatiaceae;g__	0.00 ± 0.00	0.0	0.00 ± 0.00	0.0	2.92E-01	-	-	-
k__Bacteria;p__Proteobacteria;c__Gammaproteobacteria;o__Enterobacterales;f__Enterobacteriaceae;g__	0.00 ± 0.00	0.0	0.01 ± 0.00	0.0	2.30E-01	-	-	-
k__Bacteria;p__Proteobacteria;c__Gammaproteobacteria;o__Enterobacterales;f__Enterobacteriaceae;g__Enterobacter	0.03 ± 0.03	1.2	0.01 ± 0.01	0.0	5.62E-01	-	-	-
k__Bacteria;p__Proteobacteria;c__Gammaproteobacteria;o__Enterobacterales;f__Enterobacteriaceae;g__Kosakonia	0.00 ± 0.00	0.0	0.00 ± 0.00	0.0	NA	-	-	-
k__Bacteria;p__Proteobacteria;c__Gammaproteobacteria;o__Enterobacterales;f__Erwiniaceae;g__Buchnera	0.00 ± 0.00	0.0	0.00 ± 0.00	0.0	NA	-	-	-
k__Bacteria;p__Proteobacteria;c__Gammaproteobacteria;o__Enterobacterales;f__Erwiniaceae;g__Pantoea	0.00 ± 0.00	0.0	0.00 ± 0.00	0.0	NA	-	-	-
k__Bacteria;p__Proteobacteria;c__Gammaproteobacteria;o__Enterobacterales;f__Morganellaceae;g__	0.01 ± 0.01	0.0	0.00 ± 0.00	0.0	1.86E-01	-	-	-
k__Bacteria;p__Proteobacteria;c__Gammaproteobacteria;o__Enterobacterales;f__Morganellaceae;g__Providencia	0.00 ± 0.00	0.0	0.00 ± 0.00	0.0	1.04E-01	-	-	-
k__Bacteria;p__Proteobacteria;c__Gammaproteobacteria;o__Enterobacterales;f__Pectobacteriaceae;g__	0.00 ± 0.00	0.0	0.00 ± 0.00	0.0	NA	-	-	-
k__Bacteria;p__Proteobacteria;c__Gammaproteobacteria;o__Enterobacterales;f__Yersiniaceae;g__Ewingella	0.00 ± 0.00	0.0	0.00 ± 0.00	0.0	NA	-	-	-
k__Bacteria;p__Proteobacteria;c__Gammaproteobacteria;o__Legionellales;f__Coxiellaceae;g__Diplorickettsia	0.00 ± 0.00	0.0	0.00 ± 0.00	0.0	3.55E-01	-	-	-
k__Bacteria;p__Proteobacteria;c__Gammaproteobacteria;o__Methylococcales;f__Methylococcaceae;g__	0.00 ± 0.00	0.0	0.00 ± 0.00	0.0	NA	-	-	-
k__Bacteria;p__Proteobacteria;c__Gammaproteobacteria;o__Oceanospirillales;f__Halomonadaceae;g__Chromohalobacter	0.00 ± 0.00	0.0	0.00 ± 0.00	0.0	1.86E-01	-	-	-
**k__Bacteria;p__Proteobacteria;c__Gammaproteobacteria;o__Oceanospirillales;f__Halomonadaceae;g__Halomonas**	**2.44 ± 0.63**	**27.7**	**0.00 ± 0.00**	**0.0**	**6.95E-30**	**4.1239**	**-**	**1.29E-29**
k__Bacteria;p__Proteobacteria;c__Gammaproteobacteria;o__Oceanospirillales;f__Oceanospirillaceae;g__Marinobacterium	0.00 ± 0.00	0.0	0.00 ± 0.00	0.0	3.55E-01	-	-	-
k__Bacteria;p__Proteobacteria;c__Gammaproteobacteria;o__Orbales;f__Orbaceae;g__	0.00 ± 0.00	0.0	0.00 ± 0.00	0.0	3.55E-01	-	-	-
k__Bacteria;p__Proteobacteria;c__Gammaproteobacteria;o__Pasteurellales;f__Pasteurellaceae;g__	0.00 ± 0.00	0.0	0.00 ± 0.00	0.0	NA	-	-	-
k__Bacteria;p__Proteobacteria;c__Gammaproteobacteria;o__Pasteurellales;f__Pasteurellaceae;g__Aggregatibacter	0.00 ± 0.00	0.0	0.00 ± 0.00	0.0	4.44E-04	3.1493	-	4.78E-04
**k__Bacteria;p__Proteobacteria;c__Gammaproteobacteria;o__Pasteurellales;f__Pasteurellaceae;g__Haemophilus**	**1.11 ± 0.55**	**8.4**	**0.02 ± 0.01**	**0.0**	**3.55E-11**	**3.7460**	**-**	**5.24E-11**
k__Bacteria;p__Proteobacteria;c__Gammaproteobacteria;o__Pasteurellales;f__Pasteurellaceae;g__Mannheimia	0.00 ± 0.00	0.0	0.00 ± 0.00	0.0	NA	-	-	-
k__Bacteria;p__Proteobacteria;c__Gammaproteobacteria;o__Pseudomonadales;f__Moraxellaceae;g__Acinetobacter	0.12 ± 0.07	3.6	0.03 ± 0.02	1.4	3.21E-03	2.7780	-	3.51E-03
k__Bacteria;p__Proteobacteria;c__Gammaproteobacteria;o__Pseudomonadales;f__Moraxellaceae;g__Moraxella	0.01 ± 0.01	0.0	0.00 ± 0.00	0.0	3.71E-01	-	-	-
k__Bacteria;p__Proteobacteria;c__Gammaproteobacteria;o__Pseudomonadales;f__Moraxellaceae;g__Psychrobacter	0.00 ± 0.00	0.0	0.00 ± 0.00	0.0	1.04E-01	-	-	-
k__Bacteria;p__Proteobacteria;c__Gammaproteobacteria;o__Pseudomonadales;f__Pseudomonadaceae;g__Pseudomonas	0.02 ± 0.01	0.0	0.69 ± 0.22	15.1	9.41E-01	-	-	-
k__Bacteria;p__Proteobacteria;c__Gammaproteobacteria;o__Thiotrichales;f__Francisellaceae;g__Francisella	0.00 ± 0.00	0.0	0.00 ± 0.00	0.0	3.55E-01	-	-	-
k__Bacteria;p__Proteobacteria;c__Gammaproteobacteria;o__Xanthomonadales;f__;g__	0.00 ± 0.00	0.0	0.00 ± 0.00	0.0	3.55E-01	-	-	-
k__Bacteria;p__Proteobacteria;c__Gammaproteobacteria;o__Xanthomonadales;f__Rhodanobacteraceae;g__Dokdonella	0.00 ± 0.00	0.0	0.00 ± 0.00	0.0	1.86E-01	-	-	-
k__Bacteria;p__Proteobacteria;c__Gammaproteobacteria;o__Xanthomonadales;f__Rhodanobacteraceae;g__Rhodanobacter	0.00 ± 0.00	0.0	0.00 ± 0.00	0.0	NA	-	-	-
k__Bacteria;p__Proteobacteria;c__Gammaproteobacteria;o__Xanthomonadales;f__Xanthomonadaceae;g__	0.00 ± 0.00	0.0	0.00 ± 0.00	0.0	1.04E-01	-	-	-
k__Bacteria;p__Proteobacteria;c__Gammaproteobacteria;o__Xanthomonadales;f__Xanthomonadaceae;g__Luteimonas	0.01 ± 0.01	0.0	0.00 ± 0.00	0.0	1.86E-01	-	-	-
k__Bacteria;p__Proteobacteria;c__Gammaproteobacteria;o__Xanthomonadales;f__Xanthomonadaceae;g__Lysobacter	0.00 ± 0.00	0.0	0.00 ± 0.00	0.0	1.86E-01	-	-	-
k__Bacteria;p__Proteobacteria;c__Gammaproteobacteria;o__Xanthomonadales;f__Xanthomonadaceae;g__Pseudoxanthomonas	0.00 ± 0.00	0.0	0.00 ± 0.00	0.0	NA	-	-	-
k__Bacteria;p__Proteobacteria;c__Gammaproteobacteria;o__Xanthomonadales;f__Xanthomonadaceae;g__Stenotrophomonas	0.00 ± 0.00	0.0	0.26 ± 0.14	5.5	5.73E-08	-	3.1959	4.29E-08
k__Bacteria;p__Proteobacteria;c__Gammaproteobacteria;o__Xanthomonadales;f__Xanthomonadaceae;g__Xanthomonas	0.00 ± 0.00	0.0	0.00 ± 0.00	0.0	3.55E-01	-	-	-
k__Bacteria;p__Saccharibacteria;c__;o__;f__;g__	0.00 ± 0.00	0.0	0.00 ± 0.00	0.0	NA	-	-	-
k__Bacteria;p__Spirochaetes;c__Spirochaetia;o__Brachyspirales;f__Brachyspiraceae;g__Brachyspira	0.01 ± 0.00	0.0	0.00 ± 0.00	0.0	8.18E-06	3.0162	-	9.22E-06
**k__Bacteria;p__Spirochaetes;c__Spirochaetia;o__Spirochaetales;f__Spirochaetaceae;g__**	**3.59 ± 0.48**	**57.8**	**0.00 ± 0.00**	**0.0**	**7.81E-23**	**4.2443**	**-**	**1.28E-22**
k__Bacteria;p__Spirochaetes;c__Spirochaetia;o__Spirochaetales;f__Spirochaetaceae;g__Treponema	0.97 ± 0.19	25.3	0.00 ± 0.00	0.0	6.71E-19	3.6760	-	1.02E-18
k__Bacteria;p__Synergistetes;c__Synergistia;o__Synergistales;f__Synergistaceae;g__	0.00 ± 0.00	0.0	0.00 ± 0.00	0.0	NA	-	-	-
k__Bacteria;p__Synergistetes;c__Synergistia;o__Synergistales;f__Synergistaceae;g__Fretibacterium	0.00 ± 0.00	0.0	0.00 ± 0.00	0.0	NA	-	-	-
k__Bacteria;p__Synergistetes;c__Synergistia;o__Synergistales;f__Synergistaceae;g__Jonquetella	0.00 ± 0.00	0.0	0.01 ± 0.01	1.4	2.92E-01	-	-	-
k__Bacteria;p__Synergistetes;c__Synergistia;o__Synergistales;f__Synergistaceae;g__Pyramidobacter	0.00 ± 0.00	0.0	0.00 ± 0.00	0.0	NA	-	-	-

k__Bacteria;p__Tenericutes;c__Mollicutes;o__Acholeplasmatales;f__Acholeplasmataceae;g__	0.01 ± 0.00	0.0	0.00 ± 0.00	0.0	1.47E-05	2.4066	-	1.65E-05
k__Bacteria;p__Tenericutes;c__Mollicutes;o__Anaeroplasmatales;f__Anaeroplasmataceae;g__	0.02 ± 0.01	0.0	0.00 ± 0.00	0.0	2.63E-05	2.4397	-	2.93E-05
k__Bacteria;p__Tenericutes;c__Mollicutes;o__Anaeroplasmatales;f__Anaeroplasmataceae;g__Anaeroplasma	0.00 ± 0.00	0.0	0.00 ± 0.00	0.0	1.98E-02	3.1221	-	2.04E-02
k__Bacteria;p__Tenericutes;c__Mollicutes;o__Anaeroplasmatales;f__Anaeroplasmataceae;g__Asteroleplasma	0.00 ± 0.00	0.0	0.00 ± 0.00	0.0	NA	-	-	-
k__Bacteria;p__Tenericutes;c__Mollicutes;o__Entomoplasmatales;f__Entomoplasmataceae;g__	0.00 ± 0.00	0.0	0.00 ± 0.00	0.0	3.55E-01	-	-	-
k__Bacteria;p__Tenericutes;c__Mollicutes;o__Entomoplasmatales;f__Entomoplasmataceae;g__Entomoplasma	0.00 ± 0.00	0.0	0.00 ± 0.00	0.0	NA	-	-	-
k__Bacteria;p__Tenericutes;c__Mollicutes;o__Entomoplasmatales;f__Spiroplasmataceae;g__	0.01 ± 0.01	0.0	0.00 ± 0.00	0.0	4.68E-05	2.3300	-	5.17E-05
k__Bacteria;p__Tenericutes;c__Mollicutes;o__Mollicutes_RF9;f__;g__	0.03 ± 0.01	0.0	0.00 ± 0.00	0.0	1.11E-07	2.3622	-	1.32E-07
k__Bacteria;p__Tenericutes;c__Mollicutes;o__Mycoplasmatales;f__Mycoplasmataceae;g__	0.00 ± 0.00	0.0	0.00 ± 0.00	0.0	5.91E-02	-	-	-
k__Bacteria;p__Tenericutes;c__Mollicutes;o__Mycoplasmatales;f__Mycoplasmataceae;g__Mycoplasma	0.07 ± 0.02	2.4	0.00 ± 0.00	0.0	7.45E-07	2.6882	-	8.88E-07
k__Bacteria;p__Tenericutes;c__Mollicutes;o__Mycoplasmatales;f__Mycoplasmataceae;g__Ureaplasma	0.00 ± 0.00	0.0	0.40 ± 0.19	6.8	8.53E-08	-	3.3534	1.47E-07
k__Bacteria;p__Tenericutes;c__Mollicutes;o__NB1-n;f__;g__	0.00 ± 0.00	0.0	0.00 ± 0.00	0.0	2.92E-01	-	-	-
k__Bacteria;p__Verrucomicrobia;c__;o__;f__;g__	0.03 ± 0.01	0.0	0.00 ± 0.00	0.0	8.18E-06	2.2769	-	9.22E-06
k__Bacteria;p__Verrucomicrobia;c__Opitutae;o__Opitutae_vadinHA64;f__;g__	0.00 ± 0.00	0.0	0.00 ± 0.00	0.0	3.55E-01	-	-	-
k__Bacteria;p__Verrucomicrobia;c__Opitutae;o__Puniceicoccales;f__Puniceicoccaceae;g__	0.00 ± 0.00	0.0	0.00 ± 0.00	0.0	3.41E-02	3.0872	-	3.49E-02
k__Bacteria;p__Verrucomicrobia;c__Opitutae;o__Puniceicoccales;f__Puniceicoccaceae;g__Cerasicoccus	0.00 ± 0.00	0.0	0.00 ± 0.00	0.0	3.55E-01	-	-	-
k__Bacteria;p__Verrucomicrobia;c__Spartobacteria;o__Chthoniobacterales;f__Chthoniobacteraceae;g__Chthoniobacter	0.00 ± 0.00	0.0	0.00 ± 0.00	0.0	NA	-	-	-
k__Bacteria;p__Verrucomicrobia;c__Verrucomicrobiae;o__Verrucomicrobiales;f__Akkermansiaceae;g__Akkermansia	0.00 ± 0.00	0.0	0.00 ± 0.00	0.0	NA	-	-	-

$ mean ± s.e.m

* Individual samples with >1% abundance were counted.

Table S7. Relative abundance of opportinistic pathogens as defined by PATRIC in the surveyed samples.

	Body site	Macaque			Human		
		Abundance$ (%)	(Min-Max)	Prevalence* (%)	Abundance$ (%)	(Min-Max)	Prevalence* (%)
Acinetobacter baumannii	Vaginal	0.01 ± 0.01	(0.00-0.33)	3.6			
Actinomyces odontolyticus	Oral				1.82 ± 0.14	(0.05-9.49)	97.6
Actinomyces odontolyticus	Vaginal				0.01 ± 0.01	(0.09-0.35)	1.4
Aggregatibacter actinomycetemcomitans	Oral	0.75 ± 0.09	(0.02-8.65)	85.3	0.00 ± 0.00	(0.08-0.13)	1.8
Alistipes putredinis	Anal				2.43 ± 0.27	(0.02-25.21)	64.6
Alistipes putredinis	Oral				0.01 ± 0.00	(0.02-0.63)	3.6
Alistipes putredinis	Vaginal				0.00 ± 0.00	(0.07-0.11)	1.4
Bacteroides caccae	Anal				2.22 ± 0.24	(0.02-23.05)	91.8
Bacteroides caccae	Oral				0.01 ± 0.00	(0.02-0.54)	3.6
Bacteroides caccae	Vaginal				0.01 ± 0.00	(0.04-0.18)	1.4
Bacteroides fragilis	Anal				0.74 ± 0.20	(0.03-23.50)	39.9
Bacteroides fragilis	Oral				0.01 ± 0.00	(0.03-0.50)	1.8
Bacteroides massiliensis	Anal				2.87 ± 0.61	(0.34-63.70)	30.4
Bacteroides massiliensis	Oral				0.04 ± 0.02	(0.03-3.56)	2.4
Bacteroides massiliensis	Vaginal				0.00 ± 0.00	(0.08-0.25)	1.4
Bacteroides vulgatus	Anal	0.09 ± 0.07	(0.00-7.58)	3.5	19.53 ± 1.31	(0.60-87.11)	93.7
Bacteroides vulgatus	Oral				0.09 ± 0.02	(0.03-2.98)	20.8
Bacteroides vulgatus	Vaginal				0.06 ± 0.02	(0.03-1.22)	15.1
Bifidobacterium dentium	Anal				0.02 ± 0.01	(0.02-0.62)	5.7
Bifidobacterium dentium	Oral				0.01 ± 0.00	(0.04-0.44)	1.8
Bifidobacterium dentium	Vaginal				0.02 ± 0.02	(0.03-1.51)	1.4
Campylobacter showae	Oral				0.20 ± 0.03	(0.03-2.17)	44
Campylobacter ureolyticus	Anal	0.00 ± 0.00	(0.00-0.26)	1.7			
Campylobacter ureolyticus	Vaginal	1.38 ± 0.28	(0.01-11.76)	63.9	0.28 ± 0.16	(0.03-10.95)	12.3
Capnocytophaga gingivalis	Oral	0.03 ± 0.01	(0.00-0.41)	7.8	0.47 ± 0.04	(0.04-2.12)	82.7
Capnocytophaga sputigena	Oral				0.54 ± 0.05	(0.04-3.10)	77.4
Clostridium perfringens	Vaginal				0.26 ± 0.12	(0.07-7.70)	12.3
Corynebacterium amycolatum	Vaginal				0.08 ± 0.06	(0.03-4.69)	11
Corynebacterium camporealensis	Vaginal	0.08 ± 0.06	(0.31-4.72)	3.6			
Corynebacterium matruchotii	Oral	0.03 ± 0.01	(0.00-0.42)	5.2			
Corynebacterium matruchotii	Vaginal	0.00 ± 0.00	(0.17-0.17)	1.2			
Eikenella corrodens	Oral				0.06 ± 0.01	(0.02-0.42)	18.5
Elizabethkingia miricola	Vaginal				0.08 ± 0.04	(0.04-2.14)	6.8
Fusobacterium necrophorum	Oral				0.01 ± 0.00	(0.01-0.36)	2.4
Fusobacterium nucleatum	Oral				0.43 ± 0.04	(0.02-2.39)	75.6
Fusobacterium nucleatum	Vaginal				0.02 ± 0.01	(0.03-0.49)	4.1
Fusobacterium periodonticum	Oral				2.92 ± 0.23	(0.14-22.95)	99.4
Fusobacterium periodonticum	Vaginal				0.06 ± 0.04	(0.03-2.38)	6.8
Gardnerella vaginalis	Vaginal	0.36 ± 0.17	(0.01-12.87)	21.7	2.62 ± 0.87	(0.01-37.22)	27.4
Leptotrichia goodfellowii	Oral				0.02 ± 0.00	(0.02-0.30)	6.5
Mobiluncus mulieris	Vaginal				0.07 ± 0.04	(0.03-3.02)	8.2
Morganella morganii	Vaginal	0.00 ± 0.00	(0.04-0.14)	1.2			
Mycoplasma hominis	Vaginal				0.00 ± 0.00	(0.05-0.20)	1.4
Parabacteroides distasonis	Anal				1.39 ± 0.25	(0.03-29.17)	72.8
Parabacteroides distasonis	Oral				0.01 ± 0.00	(0.03-0.36)	3.6
Parabacteroides distasonis	Vaginal				0.00 ± 0.00	(0.10-0.23)	1.4
Parvimonas micra	Oral				0.16 ± 0.02	(0.02-1.12)	45.2
Parvimonas micra	Vaginal				0.08 ± 0.04	(0.06-2.45)	9.6
Porphyromonas gingivalis	Oral	0.18 ± 0.03	(0.00-1.78)	30.2	0.03 ± 0.01	(0.04-1.58)	4.8
Porphyromonas gingivalis	Vaginal	0.00 ± 0.00	(0.20-0.20)	1.2			
Prevotella bivia	Vaginal				0.77 ± 0.34	(0.03-22.05)	32.9
Prevotella intermedia	Oral				0.08 ± 0.01	(0.03-1.05)	19.6
Rothia mucilaginosa	Oral	0.18 ± 0.03	(0.00-2.72)	44.8	0.34 ± 0.04	(0.02-3.83)	61.3
Rothia mucilaginosa	Vaginal	0.00 ± 0.00	(0.02-0.17)	1.2			
Streptococcus agalactiae	Anal	0.01 ± 0.01	(0.00-1.18)	1.7			
Streptococcus agalactiae	Vaginal	0.02 ± 0.01	(0.00-0.48)	4.8	0.20 ± 0.14	(0.04-9.79)	6.8
Streptococcus anginosus	Oral	-	-	-	0.05±0.01	(0.02-1.28)	15
Streptococcus anginosus	Vaginal	-	-	-	0.20±0.08	(0.04-3.62)	18.9
Streptococcus mutans	Oral	0.00 ± 0.00	(Inf--Inf)	0	0.03 ± 0.01	(0.02-0.44)	8.9
Streptococcus pneumoniae	Oral	0.94 ± 0.13	(0.01-7.06)	81.9	0.00 ± 0.00	(Inf--Inf)	0
Streptococcus pseudopneumoniae	Oral	0.93±0.13	(0.01-7.06)	82.1	-	-	-
Streptococcus pseudopneumoniae	Vaginal	0.01±0.00	(0.01-0.37)	2.4	-	-	-
Tannerella forsythia	Oral	0.02 ± 0.00	(0.00-0.30)	4.3	0.02 ± 0.01	(0.02-0.62)	8.9
Treponema denticola	Oral	0.04 ± 0.01	(0.00-0.69)	4.3	0.04 ± 0.01	(0.02-1.40)	12.5

$ mean ± s.e.m

* Individual samples with >0.01% abundance were counted.

Table S8. Sparse partial least squares discriminant analysis (sPLSDA) of predicted metabolic pathways discriminative between macaque and human oral microbiota.

annotation	baseMean	log2FoldChange _macaque	log2FoldChange _human	lfcSE	stat	pvalue	padj
1,1,1-Trichloro-2,2-bis(4-chlorophenyl)ethane (DDT) degradation	48.9736		**2.7112**	0.2120	12.7865	1.95E-37	4.88E-37
ABC transporters	170961.4387	**-0.2974**		0.0153	-19.4435	3.31E-84	2.24E-83
Adipocytokine signaling pathway	2903.3926		**0.9729**	0.0509	19.1271	1.50E-81	9.48E-81
Alanine, aspartate and glutamate metabolism	42648.9121		**0.1927**	0.0072	26.7821	5.23E-158	1.04E-156
alpha-Linolenic acid metabolism	291.8813		**1.2003**	0.1115	10.7680	4.88E-27	1.00E-26
Alzheimer's disease	3709.1640		**0.2502**	0.0547	4.5718	4.84E-06	6.69E-06
Amino acid metabolism	11039.4434	**-1.0902**		0.0261	-41.7692	0.00E+00	0.00E+00
Amino acid related enzymes	78862.5777	**-0.0361**		0.0101	-3.5707	3.56E-04	4.71E-04
Amino sugar and nucleotide sugar metabolism	72588.7727	**-0.3284**		0.0160	-20.5191	1.45E-93	1.16E-92
Aminoacyl-tRNA biosynthesis	68398.8738	**-0.0914**		0.0133	-6.8779	6.07E-12	9.99E-12
Aminobenzoate degradation	6898.9244	**-0.1641**		0.0238	-6.8955	5.37E-12	8.88E-12
Amoebiasis	153.2018		**2.6381**	0.1355	19.4674	2.08E-84	1.44E-83
Amyotrophic lateral sclerosis (ALS)	824.6734		**0.7716**	0.0763	10.1164	4.68E-24	9.03E-24
Antigen processing and presentation	944.8638		**0.9012**	0.0840	10.7330	7.13E-27	1.44E-26
Apoptosis	772.9362	**-1.6835**		0.0908	-18.5341	1.10E-76	6.35E-76
Arachidonic acid metabolism	2239.4012		**0.5348**	0.0301	17.7699	1.21E-70	6.47E-70
Arginine and proline metabolism	41936.0578		**0.1236**	0.0100	12.3339	5.95E-35	1.43E-34
Ascorbate and aldarate metabolism	6294.0875	**-0.9236**		0.0418	-22.0706	6.05E-108	6.01E-107
Atrazine degradation	938.8117	**-2.3533**		0.0992	-23.7326	1.66E-124	1.93E-123
Bacterial chemotaxis	8849.1740		**0.4525**	0.0763	5.9281	3.07E-09	4.68E-09
Bacterial invasion of epithelial cells	328.9031		**1.3941**	0.1016	13.7275	6.95E-43	1.93E-42
Bacterial motility proteins	21822.3576		**0.8003**	0.0668	11.9771	4.68E-33	1.07E-32
Bacterial secretion system	36116.7889	**-0.1501**		0.0227	-6.6163	3.68E-11	6.02E-11
Bacterial toxins	6892.3261	**-0.2427**		0.0268	-9.0514	1.41E-19	2.62E-19
Basal transcription factors	60.6761		**0.3586**	0.1475	2.4308	1.51E-02	1.88E-02
Base excision repair	24720.4119	**-0.1667**		0.0094	-17.7638	1.35E-70	7.07E-70
Benzoate degradation	9851.6236	**-0.0578**		0.0243	-2.3761	1.75E-02	2.16E-02
beta-Alanine metabolism	7215.1266		**0.6742**	0.0352	19.1755	5.93E-82	3.83E-81
beta-Lactam resistance	1052.3544	**-0.8440**		0.0653	-12.9315	2.99E-38	7.56E-38
Bile secretion	5.6655		**0.5379**	0.2191	2.4552	1.41E-02	1.77E-02
Biosynthesis and biodegradation of secondary metabolites	1973.4304		**0.5871**	0.0401	14.6341	1.70E-48	5.38E-48
Biosynthesis of ansamycins	3564.5033		**0.2236**	0.0264	8.4660	2.54E-17	4.58E-17
Biosynthesis of siderophore group nonribosomal peptides	2126.2679	**-0.1738**		0.0362	-4.8034	1.56E-06	2.19E-06
Biosynthesis of type II polyketide products	13.6461	**-4.4968**		0.2836	-15.8576	1.24E-56	5.32E-56
Biosynthesis of vancomycin group antibiotics	3168.2031	**-0.4255**		0.0197	-21.6125	1.37E-103	1.31E-102
Biotin metabolism	7601.9556		**0.3082**	0.0284	10.8405	2.21E-27	4.59E-27
Bisphenol degradation	2082.1820	**-0.2264**		0.0412	-5.4945	3.92E-08	5.76E-08
Bladder cancer	81.1975		**1.0096**	0.1450	6.9609	3.38E-12	5.63E-12
Butanoate metabolism	29659.3875	**-0.1759**		0.0103	-17.0708	2.45E-65	1.19E-64
Butirosin and neomycin biosynthesis	2284.9146		**0.1040**	0.0242	4.3043	1.68E-05	2.31E-05
C5-Branched dibasic acid metabolism	14276.0483		**0.1209**	0.0161	7.5012	6.32E-14	1.09E-13
Caffeine metabolism	13.1444	**-4.7012**		0.3139	-14.9771	1.04E-50	3.43E-50
Caprolactam degradation	1658.7177	**-0.9356**		0.0464	-20.1851	1.32E-90	9.94E-90
Carbohydrate digestion and absorption	1624.7593	**-1.3630**		0.0486	-28.0334	6.36E-173	1.97E-171
Carbohydrate metabolism	5849.6248	**-0.5792**		0.0313	-18.4865	2.65E-76	1.50E-75
Carbon fixation in photosynthetic organisms	29228.9521		**0.1148**	0.0105	10.8920	1.26E-27	2.65E-27
Carbon fixation pathways in prokaryotes	47518.1778		**0.1647**	0.0130	12.6669	9.02E-37	2.22E-36
Cardiac muscle contraction	894.5265		**0.6794**	0.1654	4.1073	4.00E-05	5.40E-05
Carotenoid biosynthesis	255.4368		**1.1743**	0.1277	9.1962	3.71E-20	6.92E-20
Cell division	3712.6773		**0.9936**	0.0489	20.3130	9.87E-92	7.62E-91
Cell motility and secretion	11478.3786		**0.1300**	0.0205	6.3317	2.43E-10	3.90E-10
Cellular antigens	2462.7547		**0.5336**	0.0317	16.8419	1.20E-63	5.67E-63
Chloroalkane and chloroalkene degradation	6600.5453	**-0.7220**		0.0261	-27.6977	7.45E-169	2.07E-167
Chlorocyclohexane and chlorobenzene degradation	396.2888	**-0.7334**		0.1259	-5.8227	5.79E-09	8.80E-09
Citrate cycle (TCA cycle)	32383.0624		**0.1221**	0.0229	5.3339	9.61E-08	1.38E-07
Colorectal cancer	48.9304	**-4.9720**		0.3499	-14.2080	8.18E-46	2.42E-45
Cyanoamino acid metabolism	8945.3983		**0.3749**	0.0156	23.9874	3.76E-127	4.55E-126
Cysteine and methionine metabolism	46449.6968		**0.0472**	0.0094	4.9996	5.75E-07	8.11E-07
Cytoskeleton proteins	10642.4890		**0.4791**	0.0285	16.8304	1.46E-63	6.77E-63
D-Alanine metabolism	7330.1743	**-0.2763**		0.0181	-15.2586	1.44E-52	5.35E-52
D-Arginine and D-ornithine metabolism	291.4026		**1.5646**	0.1037	15.0818	2.13E-51	7.51E-51
D-Glutamine and D-glutamate metabolism	8130.0937		**0.0904**	0.0092	9.8520	6.72E-23	1.28E-22
Dioxin degradation	3976.1982	**-0.8751**		0.0338	-25.8920	8.19E-148	1.20E-146
DNA repair and recombination proteins	157444.1608	**-0.0562**		0.0092	-6.1154	9.63E-10	1.51E-09
DNA replication	37137.2679		**0.0278**	0.0132	2.1122	3.47E-02	4.21E-02
Drug metabolism - cytochrome P450	2448.7007	**-0.4307**		0.0776	-5.5505	2.85E-08	4.24E-08
Drug metabolism - other enzymes	16908.4580	**-0.1350**		0.0164	-8.2507	1.57E-16	2.75E-16
Electron transfer carriers	1951.8837	**-1.0970**		0.0517	-21.2113	7.51E-100	6.73E-99
Energy metabolism	38351.0025		**0.2699**	0.0243	11.1261	9.37E-29	2.03E-28
Epithelial cell signaling in Helicobacter pylori infection	3845.4659		**0.0651**	0.0233	2.7986	5.13E-03	6.52E-03
Ether lipid metabolism	484.6422	**-5.0814**		0.0975	-52.1365	0.00E+00	0.00E+00
Ethylbenzene degradation	1153.7047		**1.0081**	0.0672	15.0112	6.20E-51	2.10E-50
Fatty acid biosynthesis	27698.7456	**-0.2018**		0.0136	-14.7925	1.64E-49	5.24E-49

Fatty acid metabolism	11409.1729	**-0.2408**		0.0192	-12.5210	5.73E-36	1.40E-35
Flagellar assembly	6272.1745		**2.1611**	0.1235	17.4983	1.48E-68	7.32E-68
Flavone and flavonol biosynthesis	151.3501		**0.2517**	0.1173	2.1463	3.19E-02	3.88E-02
Flavonoid biosynthesis	641.3687	**-2.4092**		0.0811	-29.6980	8.15E-194	4.53E-192
Folate biosynthesis	25381.6950		**0.0445**	0.0109	4.0871	4.37E-05	5.87E-05
Fructose and mannose metabolism	39792.4095	**-0.5107**		0.0203	-25.1694	8.67E-140	1.15E-138
Function unknown	78180.6185		**0.0939**	0.0158	5.9599	2.52E-09	3.88E-09
Galactose metabolism	32172.8545	**-0.4700**		0.0284	-16.5216	2.57E-61	1.15E-60
General function prediction only	173866.0525	**-0.1461**		0.0057	-25.5780	2.68E-144	3.73E-143
Geraniol degradation	2447.6054	**-0.3402**		0.0320	-10.6437	1.87E-26	3.73E-26
Germination	94.4546		**1.1555**	0.1595	7.2443	4.35E-13	7.32E-13
Glutamatergic synapse	2871.7577		**0.2800**	0.0250	11.2098	3.65E-29	8.05E-29
Glutathione metabolism	15246.2653	**-0.1079**		0.0179	-6.0155	1.79E-09	2.77E-09
Glycan biosynthesis and metabolism	3017.9370		**0.2003**	0.0571	3.5066	4.54E-04	5.92E-04
Glycerolipid metabolism	19010.3084	**-0.6364**		0.0211	-30.1161	2.98E-199	2.07E-197
Glycerophospholipid metabolism	29395.5776	**-0.2000**		0.0143	-14.0126	1.31E-44	3.70E-44
Glycine, serine and threonine metabolism	42438.6127	**-0.0886**		0.0068	-13.0692	4.94E-39	1.28E-38
Glycolysis / Gluconeogenesis	57477.3341	**-0.3543**		0.0134	-26.3717	2.89E-153	5.36E-152
Glycosaminoglycan degradation	2345.3240		**0.4999**	0.0422	11.8515	2.11E-32	4.78E-32
Glycosphingolipid biosynthesis - ganglio series	1893.3289		**0.4352**	0.0422	10.3133	6.14E-25	1.20E-24
Glycosphingolipid biosynthesis - globo series	3073.2292		**0.0789**	0.0385	2.0503	4.03E-02	4.88E-02
Glycosphingolipid biosynthesis - lacto and neolacto series	16.7996		**0.3370**	0.1670	2.0177	4.36E-02	5.25E-02
Glycosyltransferases	24133.6121	**-0.2132**		0.0135	-15.7687	5.11E-56	2.12E-55
Glyoxylate and dicarboxylate metabolism	19141.7655		**0.3301**	0.0214	15.4484	7.74E-54	2.95E-53
Histidine metabolism	25425.9498	**-0.6833**		0.0249	-27.4043	2.44E-165	6.16E-164
Homologous recombination	52546.4347		**0.0474**	0.0117	4.0413	5.32E-05	7.10E-05
Hypertrophic cardiomyopathy (HCM)	0.4542		**2.7987**	0.8486	3.2980	9.74E-04	1.26E-03
Influenza A	48.9304	**-4.9720**		0.3499	-14.2080	8.18E-46	2.42E-45
Inorganic ion transport and metabolism	10771.8759	**-0.1132**		0.0318	-3.5581	3.74E-04	4.90E-04
Inositol phosphate metabolism	6030.6470	**-0.1489**		0.0314	-4.7456	2.08E-06	2.89E-06
Insulin signaling pathway	4454.5409	**-0.5419**		0.0222	-24.4506	4.96E-132	6.26E-131
Ion channels	2868.3990	**-0.6061**		0.0441	-13.7539	4.82E-43	1.35E-42
Isoquinoline alkaloid biosynthesis	2282.4239		**0.7173**	0.0454	15.8102	2.65E-56	1.12E-55
Limonene and pinene degradation	3360.4811	**-0.1902**		0.0348	-5.4686	4.54E-08	6.64E-08
Linoleic acid metabolism	1580.4124	**-0.2413**		0.0435	-5.5417	3.00E-08	4.43E-08
Lipid biosynthesis proteins	30749.4154		**0.0724**	0.0099	7.3218	2.45E-13	4.20E-13
Lipoic acid metabolism	3001.6423	**-0.1612**		0.0452	-3.5645	3.65E-04	4.80E-04
Lipopolysaccharide biosynthesis	21727.9600		**0.7966**	0.0468	17.0091	7.03E-65	3.37E-64
Lipopolysaccharide biosynthesis proteins	28675.1814		**0.4754**	0.0288	16.5349	2.06E-61	9.38E-61
Lysine biosynthesis	34680.8426	**-0.0464**		0.0115	-4.0307	5.56E-05	7.40E-05
Lysine degradation	6964.6655		**0.3622**	0.0314	11.5384	8.45E-31	1.89E-30
Lysosome	3144.1086		**0.6575**	0.0409	16.0931	2.85E-58	1.24E-57
Meiosis - yeast	192.0116		**2.2931**	0.1214	18.8949	1.26E-79	7.44E-79
Membrane and intracellular structural molecules	34274.9722		**0.1770**	0.0206	8.6086	7.39E-18	1.36E-17
Metabolism of cofactors and vitamins	7992.9764	**-0.2348**		0.0185	-12.7023	5.74E-37	1.43E-36
Metabolism of xenobiotics by cytochrome P450	2450.4428	**-0.4303**		0.0772	-5.5756	2.47E-08	3.69E-08
Methane metabolism	50283.4947	**-0.2226**		0.0115	-19.2929	6.16E-83	4.08E-82
Mineral absorption	683.3228		**1.2784**	0.0715	17.8804	1.68E-71	9.14E-71
Mismatch repair	44342.9608		**0.0384**	0.0129	2.9661	3.02E-03	3.88E-03
N-Glycan biosynthesis	1138.2217		**1.6206**	0.0818	19.8094	2.47E-87	1.81E-86
Naphthalene degradation	6657.1138	**-0.2912**		0.0264	-11.0286	2.78E-28	5.99E-28
Nicotinate and nicotinamide metabolism	21539.5935		**0.1954**	0.0143	13.7128	8.51E-43	2.34E-42
Nitrogen metabolism	33370.5237		**0.3047**	0.0160	19.1006	2.49E-81	1.54E-80
Nitrotoluene degradation	1883.0563		**1.0798**	0.0616	17.5408	7.00E-69	3.54E-68
NOD-like receptor signaling pathway	1259.9797		**1.2082**	0.0802	15.0583	3.04E-51	1.06E-50
Non-homologous end-joining	462.1806		**0.9130**	0.0878	10.4008	2.46E-25	4.85E-25
Novobiocin biosynthesis	6254.5746		**0.0796**	0.0191	4.1679	3.07E-05	4.17E-05
Nucleotide excision repair	20062.6862		**0.1532**	0.0126	12.2007	3.08E-34	7.32E-34
Nucleotide metabolism	5160.2115	**-0.9184**		0.0327	-28.1132	6.75E-174	2.35E-172
One carbon pool by folate	33665.1543	**-0.0254**		0.0107	-2.3873	1.70E-02	2.11E-02
Other glycan degradation	7212.3804		**0.6454**	0.0358	18.0227	1.29E-72	7.19E-72
Other ion-coupled transporters	56924.2908		**0.2246**	0.0143	15.7579	6.06E-56	2.48E-55
Other transporters	10804.4755		**0.1005**	0.0176	5.7194	1.07E-08	1.61E-08
Others	46882.9091	**-0.4349**		0.0168	-25.9381	2.48E-148	3.82E-147
Oxidative phosphorylation	52358.0568		**0.0592**	0.0275	2.1490	3.16E-02	3.87E-02
p53 signaling pathway	48.9837	**-4.9736**		0.3495	-14.2308	5.90E-46	1.84E-45
Pantothenate and CoA biosynthesis	29923.6155		**0.1095**	0.0056	19.7318	1.15E-86	8.19E-86
Parkinson's disease	899.2329		**0.6704**	0.1593	4.2073	2.58E-05	3.54E-05
Pathways in cancer	2136.8265		**0.3483**	0.0611	5.7029	1.18E-08	1.77E-08
Penicillin and cephalosporin biosynthesis	1760.6067	**-1.1326**		0.0535	-21.1712	1.76E-99	1.53E-98
Pentose and glucuronate interconversions	12826.6829	**-0.0833**		0.0288	-2.8910	3.84E-03	4.90E-03
Pentose phosphate pathway	36490.5547		**0.0335**	0.0114	2.9413	3.27E-03	4.19E-03
Peptidases	92923.0658	**-0.1050**		0.0074	-14.1899	1.06E-45	3.06E-45
Peptidoglycan biosynthesis	49975.8608	**-0.2808**		0.0108	-26.0365	1.91E-149	3.13E-148
Pertussis	1472.9269	**-0.7329**		0.0665	-11.0251	2.89E-28	6.18E-28
Phenylalanine metabolism	5981.7853		**0.3051**	0.0277	11.0119	3.35E-28	7.10E-28
Phenylalanine, tyrosine and tryptophan biosynthesis	42377.3289	**-0.0641**		0.0152	-4.2044	2.62E-05	3.57E-05

Phosphatidylinositol signaling system	6200.1655	**-0.2628**		0.0256	-10.2672	9.91E-25	1.93E-24
Phosphonate and phosphinate metabolism	3454.3708	**-0.7382**		0.0257	-28.7575	7.29E-182	3.38E-180
Phosphotransferase system (PTS)	40939.8598	**-1.4952**		0.0480	-31.1307	9.27E-213	8.59E-211
Photosynthesis	20658.9110	**-0.1366**		0.0189	-7.2322	4.75E-13	7.96E-13
Photosynthesis proteins	21163.2588	**-0.1579**		0.0185	-8.5181	1.62E-17	2.97E-17
Plant-pathogen interaction	5951.2858		**0.2891**	0.0191	15.1516	7.39E-52	2.70E-51
Polycyclic aromatic hydrocarbon degradation	6319.9704	**-0.3055**		0.0231	-13.2509	4.46E-40	1.17E-39
Polyketide sugar unit biosynthesis	8992.8239	**-0.2456**		0.0220	-11.1472	7.39E-29	1.62E-28
Pores ion channels	25729.5617		**0.4341**	0.0320	13.5459	8.38E-42	2.26E-41
Porphyrin and chlorophyll metabolism	44357.9106	**-0.3636**		0.0242	-15.0505	3.43E-51	1.18E-50
PPAR signaling pathway	4458.7315		**0.3763**	0.0252	14.9235	2.32E-50	7.58E-50
Prenyltransferases	18558.9580		**0.0709**	0.0140	5.0526	4.36E-07	6.18E-07
Primary bile acid biosynthesis	71.8943	**-0.7988**		0.1322	-6.0402	1.54E-09	2.39E-09
Primary immunodeficiency	2653.4124		**0.3289**	0.0212	15.4957	3.71E-54	1.45E-53
Prion diseases	309.7551		**0.6339**	0.0755	8.3994	4.49E-17	8.05E-17
Progesterone-mediated oocyte maturation	944.8638		**0.9012**	0.0840	10.7330	7.13E-27	1.44E-26
Propanoate metabolism	25639.6445	**-0.1396**		0.0108	-12.9695	1.82E-38	4.69E-38
Prostate cancer	946.8102		**0.9027**	0.0840	10.7476	6.08E-27	1.24E-26
Proteasome	1192.0623		**0.9255**	0.0772	11.9836	4.33E-33	9.94E-33
Protein digestion and absorption	955.1947		**1.4529**	0.1096	13.2534	4.31E-40	1.14E-39
Protein export	37091.6983	**-0.1899**		0.0134	-14.1975	9.49E-46	2.78E-45
Protein folding and associated processing	37125.5250		**0.1733**	0.0075	22.9934	5.43E-117	5.59E-116
Protein kinases	11619.8136	**-0.4257**		0.0286	-14.8709	5.09E-50	1.65E-49
Protein processing in endoplasmic reticulum	1459.0317		**1.1941**	0.0874	13.6560	1.86E-42	5.07E-42
Proximal tubule bicarbonate reclamation	559.0303		**0.9863**	0.0819	12.0466	2.02E-33	4.68E-33
Purine metabolism	128237.4552	**-0.0207**		0.0061	-3.4148	6.38E-04	8.29E-04
Pyrimidine metabolism	104883.5320	**-0.0720**		0.0086	-8.3530	6.66E-17	1.18E-16
Pyruvate metabolism	54564.9315	**-0.3678**		0.0131	-28.1615	1.73E-174	6.88E-173
Renal cell carcinoma	1127.7318		**0.1407**	0.0524	2.6861	7.23E-03	9.14E-03
Replication, recombination and repair proteins	36950.1554		**0.0400**	0.0084	4.7726	1.82E-06	2.54E-06
Restriction enzyme	8164.0302		**0.2961**	0.0348	8.4995	1.90E-17	3.46E-17
Retinol metabolism	2373.1510	**-0.2952**		0.0550	-5.3658	8.06E-08	1.17E-07
Riboflavin metabolism	13006.8694		**0.2993**	0.0241	12.4188	2.07E-35	4.99E-35
Ribosome	140079.6947	**-0.0848**		0.0130	-6.5200	7.03E-11	1.14E-10
Ribosome Biogenesis	86260.2640	**-0.1483**		0.0098	-15.1225	1.15E-51	4.16E-51
RIG-I-like receptor signaling pathway	141.8272		**1.1521**	0.1377	8.3655	5.98E-17	1.07E-16
RNA degradation	24402.4995		**0.0945**	0.0123	7.6913	1.46E-14	2.53E-14
RNA polymerase	10840.4713	**-0.2972**		0.0143	-20.7667	8.67E-96	7.09E-95
RNA transport	6802.3372	**-0.3795**		0.0242	-15.6584	2.91E-55	1.17E-54
Secondary bile acid biosynthesis	57.0911	**-0.2715**		0.1363	-1.9921	4.64E-02	5.56E-02
Secretion system	74578.7313	**-0.3062**		0.0200	-15.2849	9.64E-53	3.62E-52
Selenocompound metabolism	20144.1743	**-0.0395**		0.0078	-5.0854	3.67E-07	5.26E-07
Signal transduction mechanisms	21715.4309	**-0.3153**		0.0150	-21.0773	1.29E-98	1.08E-97
Small cell lung cancer	48.9304	**-4.9720**		0.3499	-14.2080	8.18E-46	2.42E-45
Sporulation	3555.4265		**0.3388**	0.0666	5.0843	3.69E-07	5.26E-07
Staphylococcus aureus infection	4233.3229	**-1.4056**		0.0661	-21.2753	1.92E-100	1.78E-99
Starch and sucrose metabolism	39564.5061	**-0.3983**		0.0210	-18.9248	7.12E-80	4.30E-79
Steroid hormone biosynthesis	170.1005		**2.7585**	0.1571	17.5631	4.73E-69	2.43E-68
Stilbenoid, diarylheptanoid and gingerol biosynthesis	312.3335	**-0.9091**		0.0836	-10.8762	1.50E-27	3.13E-27
Streptomycin biosynthesis	13912.4508	**-0.1156**		0.0159	-7.2523	4.10E-13	6.99E-13
Styrene degradation	957.9025		**0.4551**	0.0699	6.5133	7.35E-11	1.19E-10
Sulfur metabolism	12920.2300		**0.1633**	0.0168	9.7348	2.14E-22	4.05E-22
Sulfur relay system	13840.6674		**0.1151**	0.0184	6.2603	3.84E-10	6.14E-10
Synthesis and degradation of ketone bodies	2284.4026	**-0.4644**		0.0381	-12.1848	3.75E-34	8.83E-34
Systemic lupus erythematosus	143.4051		**2.7498**	0.1820	15.1056	1.49E-51	5.30E-51
Taurine and hypotaurine metabolism	6397.7852	**-0.2169**		0.0190	-11.4136	3.57E-30	7.95E-30
Terpenoid backbone biosynthesis	30279.2443		**0.1800**	0.0110	16.3563	3.92E-60	1.73E-59
Tetracycline biosynthesis	8098.9356	**-0.5391**		0.0345	-15.6473	3.46E-55	1.38E-54
Thiamine metabolism	22220.8319		**0.3247**	0.0139	23.4342	1.92E-121	2.13E-120
Toluene degradation	5604.9828		**0.8313**	0.0554	15.0052	6.79E-51	2.27E-50
Toxoplasmosis	48.9304	**-4.9720**		0.3499	-14.2080	8.18E-46	2.42E-45
Transcription factors	66942.7130	**-0.4439**		0.0164	-27.1420	3.15E-162	7.30E-161
Transcription machinery	35369.7830		**0.0966**	0.0158	6.0960	1.09E-09	1.70E-09
Transcription related proteins	250.9683		**2.4458**	0.1050	23.2914	5.41E-120	5.79E-119
Translation factors	30300.1246		**0.0648**	0.0120	5.4185	6.01E-08	8.75E-08
Translation proteins	54118.4726	**-0.1142**		0.0081	-14.0691	5.88E-45	1.68E-44
Transporters	322102.0786	**-0.5017**		0.0190	-26.3420	6.34E-153	1.10E-151
Tropane, piperidine and pyridine alkaloid biosynthesis	4802.2401		**0.3268**	0.0244	13.3964	6.35E-41	1.70E-40
Tuberculosis	7017.2995	**-0.1898**		0.0191	-9.9404	2.78E-23	5.32E-23
Two-component system	50687.9172	**-0.0405**		0.0173	-2.3394	1.93E-02	2.38E-02
Type I diabetes mellitus	2838.0300	**-0.1091**		0.0150	-7.2486	4.21E-13	7.14E-13
Tyrosine metabolism	16708.4899	**-0.1997**		0.0154	-12.9348	2.87E-38	7.31E-38
Ubiquinone and other terpenoid-quinone biosynthesis	12742.5381		**0.4104**	0.0391	10.4973	8.89E-26	1.77E-25
Ubiquitin system	548.0966		**1.0727**	0.0694	15.4588	6.58E-54	2.54E-53
Valine, leucine and isoleucine biosynthesis	37042.0508	**-0.1952**		0.0161	-12.0965	1.10E-33	2.57E-33
Valine, leucine and isoleucine degradation	13229.0417	**-0.1621**		0.0194	-8.3487	6.90E-17	1.21E-16
Various types of N-glycan biosynthesis	58.9935		**2.0658**	0.2166	9.5355	1.49E-21	2.80E-21

Vibrio cholerae infection	0.0956		**1.9892**	0.8106	2.4540	1.41E-02	1.77E-02
Vibrio cholerae pathogenic cycle	4180.5170	**-0.1702**		0.0272	-6.2581	3.90E-10	6.19E-10
Viral myocarditis	48.9304	**-4.9720**		0.3499	-14.2080	8.18E-46	2.42E-45
Vitamin B6 metabolism	9706.1012		**0.0802**	0.0129	6.2031	5.54E-10	8.75E-10
Xylene degradation	2514.425248	**-1.179250994**		0.0436	-27.0552	3.3E-161	7.1E-160

Table S9. Sparse partial least squares discriminant analysis (sPLSDA) of predicted metabolic pathways discriminative between macaque and human anal microbiota.

annotation	baseMean	log2FoldChange _macaque	log2FoldChange _human	lfcSE	stat	pvalue	padj
1,1,1-Trichloro-2,2-bis(4-chlorophenyl)ethane (DDT) degradation	700.3469	**-4.6707**		0.2276	-20.5204	1.42E-93	9.52E-93
ABC transporters	184567.8434	**-0.3510**		0.0311	-11.2927	1.43E-29	3.07E-29
Adipocytokine signaling pathway	6451.1144		**1.2878**	0.0788	16.3380	5.30E-60	1.93E-59
Alanine, aspartate and glutamate metabolism	87179.0452		**0.2281**	0.0093	24.4110	1.31E-131	2.70E-130
alpha-Linolenic acid metabolism	804.1287	**-3.5815**		0.1599	-22.4040	3.60E-111	3.73E-110
Alzheimer's disease	6493.7830	**-1.5521**		0.0728	-21.3260	6.52E-101	5.48E-100
Amino acid metabolism	12643.4788		**0.4373**	0.0376	11.6286	2.95E-31	6.78E-31
Amino acid related enzymes	116166.7853	**-0.1802**		0.0073	-24.5690	2.71E-133	6.07E-132
Amino sugar and nucleotide sugar metabolism	113306.4784		**0.2932**	0.0153	19.1761	5.86E-82	3.35E-81
Aminoacyl-tRNA biosynthesis	92766.5069	**-0.4277**		0.0201	-21.2692	2.19E-100	1.79E-99
Aminobenzoate degradation	7779.1131		**0.3318**	0.0288	11.5051	1.24E-30	2.80E-30
Amoebiasis	793.3505		**2.0999**	0.1153	18.2161	3.84E-74	1.75E-73
Amyotrophic lateral sclerosis (ALS)	2078.3164	**-1.7666**		0.1191	-14.8278	9.69E-50	2.83E-49
Antigen processing and presentation	3647.2092	**-0.1067**		0.0183	-5.8387	5.26E-09	7.69E-09
Apoptosis	146.2025		**2.5743**	0.2220	11.5975	4.24E-31	9.67E-31
Arachidonic acid metabolism	1907.2929	**-0.4943**		0.1059	-4.6655	3.08E-06	4.20E-06
Arginine and proline metabolism	92347.1841		**0.1518**	0.0095	15.9941	1.40E-57	4.72E-57
Ascorbate and aldarate metabolism	7112.3577		**0.2116**	0.0354	5.9734	2.32E-09	3.47E-09
Atrazine degradation	1259.7919	**-1.7534**		0.1144	-15.3326	4.63E-53	1.47E-52
Bacterial chemotaxis	34156.5098	**-1.4614**		0.0847	-17.2533	1.06E-66	4.37E-66
Bacterial invasion of epithelial cells	15.0579	**-3.1375**		0.2847	-11.0192	3.09E-28	6.48E-28
Bacterial motility proteins	76766.3009	**-1.6578**		0.1001	-16.5547	1.48E-61	5.61E-61
Bacterial secretion system	49347.4125	**-0.5423**		0.0320	-16.9299	2.71E-64	1.06E-63
Bacterial toxins	8702.7901		**0.6931**	0.0267	25.9350	2.68E-148	7.22E-147
Basal transcription factors	23.7953	**-6.8043**		0.2267	-30.0197	5.43E-198	3.65E-196
Base excision repair	32687.1950	**-0.1161**		0.0090	-12.8805	5.79E-38	1.51E-37
Benzoate degradation	12344.4439	**-0.1010**		0.0313	-3.2295	1.24E-03	1.59E-03
beta-Alanine metabolism	14949.6246	**-0.2442**		0.0154	-15.8767	9.19E-57	3.05E-56
beta-Lactam resistance	1849.5475		**0.9992**	0.0903	11.0636	1.88E-28	3.99E-28
Bile secretion	7.0380	**-2.9911**		0.4199	-7.1225	1.06E-12	1.72E-12
Biosynthesis and biodegradation of secondary metabolites	5026.0623	**-0.1025**		0.0513	-1.9985	4.57E-02	5.36E-02
Biosynthesis of ansamycins	8271.4818	**-0.2563**		0.0280	-9.1495	5.72E-20	1.02E-19
Biosynthesis of siderophore group nonribosomal peptides	1481.6355		**0.7909**	0.0937	8.4385	3.21E-17	5.58E-17
Biosynthesis of type II polyketide products	0.0471		**1.8584**	0.9081	2.0465	4.07E-02	4.82E-02
Biosynthesis of unsaturated fatty acids	7905.2585	**-0.3205**		0.0189	-16.9501	1.92E-64	7.60E-64
Biosynthesis of vancomycin group antibiotics	5617.7448		**0.3340**	0.0211	15.8507	1.39E-56	4.51E-56
Biotin metabolism	15311.6386	**-0.4991**		0.0454	-10.9870	4.41E-28	9.13E-28
Bisphenol degradation	5600.9672		**0.9700**	0.0823	11.7838	4.73E-32	1.12E-31
Bladder cancer	12.4530	**-3.2491**		0.3253	-9.9871	1.74E-23	3.33E-23
Butanoate metabolism	42894.4038	**-0.0352**		0.0153	-2.3030	2.13E-02	2.57E-02
Butirosin and neomycin biosynthesis	4997.3940		**0.8525**	0.0818	10.4254	1.90E-25	3.78E-25
Caffeine metabolism	8.4749		**7.0493**	0.5922	11.9029	1.14E-32	2.75E-32
Caprolactam degradation	629.0502	**-0.9324**		0.1180	-7.9026	2.73E-15	4.65E-15
Carbohydrate metabolism	12407.1424		**1.2092**	0.0814	14.8600	5.99E-50	1.77E-49
Carbon fixation pathways in prokaryotes	87249.6672	**-0.1591**		0.0216	-7.3836	1.54E-13	2.54E-13
Cardiac muscle contraction	2100.2905	**-5.0926**		0.4678	-10.8853	1.35E-27	2.76E-27
Carotenoid biosynthesis	131.9074		**0.7896**	0.1824	4.3285	1.50E-05	2.03E-05
Cell cycle - Caulobacter	38557.3789	**-0.1422**		0.0107	-13.2768	3.15E-40	8.40E-40
Cell division	6129.0261		**0.3627**	0.0214	16.9759	1.24E-64	4.97E-64
Cell motility and secretion	16489.0132	**-0.4879**		0.0409	-11.9370	7.59E-33	1.84E-32
Cellular antigens	4394.2245		**1.3153**	0.1032	12.7434	3.39E-37	8.78E-37
Chagas disease (American trypanosomiasis)	21.1549		**0.6538**	0.3007	2.1740	2.97E-02	3.55E-02
Chaperones and folding catalysts	85736.2813	**-0.0452**		0.0117	-3.8754	1.06E-04	1.40E-04
Chloroalkane and chloroalkene degradation	10641.0994		**0.0610**	0.0269	2.2641	2.36E-02	2.83E-02
Chlorocyclohexane and chlorobenzene degradation	1140.1716	**-1.7339**		0.1151	-15.0583	3.05E-51	9.21E-51
Chromosome	120605.0401	**-0.1086**		0.0086	-12.6346	1.36E-36	3.45E-36
Circadian rhythm - plant	8.5007		**6.3635**	0.6370	9.9898	1.69E-23	3.27E-23
Citrate cycle (TCA cycle)	56127.9217	**-0.1210**		0.0293	-4.1274	3.67E-05	4.91E-05
Cyanoamino acid metabolism	23631.9180		**0.9911**	0.0545	18.1800	7.43E-74	3.33E-73
Cysteine and methionine metabolism	70991.2776	**-0.1004**		0.0065	-15.5147	2.76E-54	8.84E-54
Cytoskeleton proteins	27768.6364	**-0.1004**		0.0201	-5.0056	5.57E-07	7.80E-07
D-Alanine metabolism	7351.9604	**-0.3688**		0.0198	-18.6028	3.05E-77	1.55E-76
D-Arginine and D-ornithine metabolism	123.3385	**-0.9768**		0.1757	-5.5590	2.71E-08	3.93E-08
D-Glutamine and D-glutamate metabolism	12157.7047	**-0.0492**		0.0104	-4.7432	2.10E-06	2.89E-06
Dioxin degradation	2867.3249		**0.6796**	0.0864	7.8609	3.81E-15	6.45E-15
DNA repair and recombination proteins	212365.5270	**-0.0867**		0.0082	-10.5343	6.00E-26	1.20E-25
DNA replication	51808.5895	**-0.1511**		0.0109	-13.8054	2.37E-43	6.56E-43
DNA replication proteins	95145.8666	**-0.0497**		0.0100	-4.9444	7.64E-07	1.06E-06
Drug metabolism - cytochrome P450	1711.9319	**-0.7470**		0.0952	-7.8422	4.43E-15	7.44E-15
Drug metabolism - other enzymes	23548.7826		**0.4871**	0.0518	9.4003	5.44E-21	9.89E-21
Electron transfer carriers	2021.9861	**-2.6841**		0.1107	-24.2526	6.20E-130	1.11E-128
Energy metabolism	70162.0212		**0.1336**	0.0072	18.5637	6.32E-77	3.09E-76

Ether lipid metabolism	81.3202	**-0.3253**		0.1592	-2.0436	4.10E-02	4.84E-02
Fatty acid biosynthesis	38426.3881	**-0.1936**		0.0217	-8.9017	5.50E-19	9.73E-19
Fatty acid elongation in mitochondria	9.4343		**5.3768**	0.5373	10.0063	1.43E-23	2.79E-23
Fatty acid metabolism	13663.2394		**0.2714**	0.0381	7.1208	1.07E-12	1.73E-12
Flagellar assembly	40832.4428	**-2.1081**		0.1139	-18.5073	1.80E-76	8.66E-76
Flavone and flavonol biosynthesis	660.7606		**2.3420**	0.1356	17.2715	7.71E-67	3.24E-66
Flavonoid biosynthesis	261.2891		**1.2731**	0.1467	8.6785	4.01E-18	7.00E-18
Folate biosynthesis	32849.2827	**-0.2346**		0.0215	-10.9295	8.33E-28	1.71E-27
Fructose and mannose metabolism	66762.8021		**0.4661**	0.0410	11.3716	5.79E-30	1.28E-29
Function unknown	98197.5368	**-0.2640**		0.0247	-10.6940	1.09E-26	2.20E-26
G protein-coupled receptors	0.2913		**3.8650**	1.1437	3.3795	7.26E-04	9.35E-04
Galactose metabolism	57623.6664		**0.9502**	0.0566	16.8012	2.39E-63	9.20E-63
General function prediction only	270871.8933		**0.0620**	0.0052	11.9847	4.27E-33	1.05E-32
Geraniol degradation	2936.5936		**0.9928**	0.0863	11.5048	1.25E-30	2.80E-30
Glutamatergic synapse	8480.8207		**0.6569**	0.0241	27.2213	3.63E-163	1.40E-161
Glutathione metabolism	16045.7599	**-0.1904**		0.0291	-6.5544	5.59E-11	8.63E-11
Glycan biosynthesis and metabolism	4316.5177	**-1.2545**		0.0728	-17.2324	1.52E-66	6.19E-66
Glycerolipid metabolism	23703.0148		**0.0796**	0.0332	2.3952	1.66E-02	2.04E-02
Glycerophospholipid metabolism	42077.5546	**-0.1660**		0.0189	-8.7621	1.92E-18	3.37E-18
Glycine, serine and threonine metabolism	63148.7261	**-0.0263**		0.0061	-4.3162	1.59E-05	2.14E-05
Glycolysis / Gluconeogenesis	78619.3065		**0.0957**	0.0129	7.4156	1.21E-13	2.01E-13
Glycosaminoglycan degradation	10539.5831		**2.6988**	0.1112	24.2796	3.22E-130	6.18E-129
Glycosphingolipid biosynthesis - ganglio series	7510.2510		**2.5571**	0.1128	22.6760	7.72E-114	8.97E-113
Glycosphingolipid biosynthesis - globo series	11980.9120		**1.8746**	0.0902	20.7907	5.25E-96	3.72E-95
Glyoxylate and dicarboxylate metabolism	40825.1343	**-0.0652**		0.0180	-3.6108	3.05E-04	3.97E-04
Histidine metabolism	50440.4927		**0.2960**	0.0122	24.1683	4.80E-129	8.07E-128
Homologous recombination	72447.0153	**-0.0938**		0.0093	-10.0881	6.24E-24	1.22E-23
Huntington's disease	5233.7325	**-1.7930**		0.1087	-16.5017	3.56E-61	1.33E-60
Inorganic ion transport and metabolism	16366.6700		**0.1286**	0.0368	3.4965	4.71E-04	6.10E-04
Inositol phosphate metabolism	7403.3207		**0.4540**	0.0257	17.6960	4.50E-70	1.92E-69
Insulin signaling pathway	6540.1169		**0.3075**	0.0272	11.3075	1.21E-29	2.61E-29
Ion channels	841.8987		**1.1335**	0.1211	9.3617	7.84E-21	1.42E-20
Isoflavonoid biosynthesis	8.6010	**-5.4627**		0.3407	-16.0326	7.56E-58	2.58E-57
Limonene and pinene degradation	4986.9810	**-0.1121**		0.0284	-3.9532	7.71E-05	1.03E-04
Linoleic acid metabolism	4744.3795		**1.0543**	0.0840	12.5473	4.11E-36	1.02E-35
Lipid biosynthesis proteins	46764.4209		**0.0948**	0.0075	12.6153	1.74E-36	4.37E-36
Lipid metabolism	9593.8179	**-0.1496**		0.0233	-6.4213	1.35E-10	2.08E-10
Lipoic acid metabolism	2872.4171		**2.2785**	0.0925	24.6423	4.45E-134	1.09E-132
Lipopolysaccharide biosynthesis	35006.5273	**-0.6763**		0.0701	-9.6413	5.35E-22	9.85E-22
Lipopolysaccharide biosynthesis proteins	44319.6854	**-0.4171**		0.0625	-6.6728	2.51E-11	3.92E-11
Lysine biosynthesis	62010.6154	**-0.0750**		0.0092	-8.1120	4.98E-16	8.53E-16
Lysine degradation	9254.2651	**-0.3941**		0.0272	-14.4942	1.32E-47	3.73E-47
Lysosome	14836.9362		**2.7078**	0.1030	26.2877	2.65E-152	7.92E-151
MAPK signaling pathway - yeast	3375.8742		**1.2220**	0.0814	15.0188	5.53E-51	1.65E-50
Meiosis - yeast	223.1356	**-0.7387**		0.1508	-4.8987	9.65E-07	1.33E-06
Membrane and intracellular structural molecules	54303.7596		**0.2567**	0.0374	6.8651	6.64E-12	1.05E-11
Metabolism of cofactors and vitamins	9023.5355	**-0.6935**		0.0388	-17.8619	2.34E-71	1.01E-70
Metabolism of xenobiotics by cytochrome P450	1682.3940	**-0.7890**		0.0968	-8.1463	3.75E-16	6.47E-16
Methane metabolism	90538.7630	**-0.0223**		0.0096	-2.3197	2.04E-02	2.47E-02
Mineral absorption	400.5948	**-1.2238**		0.1676	-7.3030	2.82E-13	4.59E-13
Mismatch repair	63375.5000	**-0.2030**		0.0095	-21.4353	6.26E-102	5.43E-101
N-Glycan biosynthesis	2571.7713	**-0.3608**		0.0489	-7.3763	1.63E-13	2.67E-13
Naphthalene degradation	9426.7374		**0.2498**	0.0200	12.4856	8.95E-36	2.21E-35
Nicotinate and nicotinamide metabolism	33248.0458	**-0.0438**		0.0080	-5.5050	3.69E-08	5.31E-08
Nitrogen metabolism	61270.0773	**-0.1563**		0.0264	-5.9190	3.24E-09	4.79E-09
Nitrotoluene degradation	6261.4974	**-1.5862**		0.0812	-19.5229	7.01E-85	4.10E-84
NOD-like receptor signaling pathway	4029.1933		**0.0474**	0.0186	2.5541	1.06E-02	1.32E-02
Non-homologous end-joining	1066.5620	**-1.6710**		0.1436	-11.6331	2.80E-31	6.48E-31
Novobiocin biosynthesis	10777.3396	**-0.2465**		0.0118	-20.9167	3.77E-97	2.74E-96
Nucleotide excision repair	31260.2347	**-0.3022**		0.0132	-22.8283	2.40E-115	3.07E-114
Other glycan degradation	32910.2165		**2.1808**	0.0959	22.7480	1.50E-114	1.84E-113
Other ion-coupled transporters	104296.7430		**0.1225**	0.0161	7.6062	2.82E-14	4.72E-14
Other transporters	19806.8231		**0.3290**	0.0157	20.9241	3.23E-97	2.42E-96
Others	65964.3879		**0.1559**	0.0161	9.7045	2.88E-22	5.39E-22
Oxidative phosphorylation	92628.0958	**-0.1059**		0.0149	-7.1190	1.09E-12	1.74E-12
Pantothenate and CoA biosynthesis	49832.7620	**-0.2451**		0.0132	-18.6293	1.86E-77	9.63E-77
Parkinson's disease	2139.9437	**-4.6827**		0.4538	-10.3197	5.74E-25	1.13E-24
Pathways in cancer	4707.9020	**-0.5742**		0.0376	-15.2691	1.23E-52	3.80E-52
Penicillin and cephalosporin biosynthesis	1984.4920		**1.7668**	0.0854	20.6973	3.66E-95	2.52E-94
Pentose and glucuronate interconversions	39466.5583		**0.9752**	0.0371	26.3184	1.18E-152	3.97E-151
Pentose phosphate pathway	61459.7586		**0.2264**	0.0194	11.6448	2.44E-31	5.70E-31
Peptidases	141048.8588		**0.0681**	0.0103	6.6389	3.16E-11	4.91E-11
Peptidoglycan biosynthesis	60967.4413	**-0.1585**		0.0119	-13.3246	1.66E-40	4.48E-40
Peroxisome	15973.3074		**0.2347**	0.0247	9.5017	2.06E-21	3.78E-21
Pertussis	2091.7835		**2.3316**	0.1108	21.0360	3.07E-98	2.36E-97
Phenylalanine metabolism	14289.3956		**0.4470**	0.0212	21.1180	5.43E-99	4.30E-98

Phenylalanine, tyrosine and tryptophan biosynthesis	65867.3857	**-0.2136**		0.0130	-16.4074	1.69E-60	6.24E-60
Phenylpropanoid biosynthesis	13913.4390		**1.4965**	0.0930	16.0984	2.62E-58	9.15E-58
Phosphatidylinositol signaling system	6719.6941		**0.6653**	0.0179	37.1325	8.41E-302	1.13E-299
Phosphonate and phosphinate metabolism	3936.1518		**1.4905**	0.0787	18.9281	6.69E-80	3.53E-79
Phosphotransferase system (PTS)	17560.4710	**-0.6444**		0.1041	-6.1910	5.98E-10	9.04E-10
Photosynthesis	31456.0873	**-0.4344**		0.0201	-21.6396	7.61E-104	7.06E-103
Photosynthesis proteins	31570.2311	**-0.4334**		0.0200	-21.6652	4.37E-104	4.20E-103
Plant-pathogen interaction	13319.7607	**-0.6124**		0.0322	-19.0141	1.30E-80	7.02E-80
Polycyclic aromatic hydrocarbon degradation	8476.5878		**0.0370**	0.0132	2.8102	4.95E-03	6.19E-03
Polyketide sugar unit biosynthesis	17845.1530		**0.3451**	0.0186	18.5790	4.76E-77	2.37E-76
Pores ion channels	36308.5567		**0.5240**	0.0399	13.1253	2.36E-39	6.22E-39
Porphyrin and chlorophyll metabolism	64187.6279	**-0.0656**		0.0222	-2.9523	3.15E-03	3.96E-03
PPAR signaling pathway	8541.0583		**1.0034**	0.0741	13.5419	8.84E-42	2.40E-41
Prenyltransferases	26270.1055	**-0.0599**		0.0116	-5.1658	2.39E-07	3.39E-07
Primary bile acid biosynthesis	2782.7435		**1.9044**	0.0973	19.5675	2.92E-85	1.79E-84
Prion diseases	870.6628	**-3.1485**		0.1339	-23.5138	2.94E-122	3.96E-121
Progesterone-mediated oocyte maturation	3647.2092	**-0.1067**		0.0183	-5.8387	5.26E-09	7.69E-09
Propanoate metabolism	36993.3228	**-0.3714**		0.0207	-17.9550	4.39E-72	1.93E-71
Prostate cancer	4000.8667		**0.1220**	0.0242	5.0457	4.52E-07	6.37E-07
Proteasome	3651.2431	**-0.1050**		0.0182	-5.7691	7.97E-09	1.16E-08
Protein digestion and absorption	2877.5273		**1.4914**	0.1172	12.7301	4.02E-37	1.03E-36
Protein export	49227.7414	**-0.2756**		0.0170	-16.1977	5.24E-59	1.88E-58
Protein folding and associated processing	52126.6379	**-0.3400**		0.0247	-13.7911	2.88E-43	7.92E-43
Protein kinases	18649.9537	**-0.0464**		0.0200	-2.3228	2.02E-02	2.46E-02
Protein processing in endoplasmic reticulum	6868.6866		**0.0617**	0.0312	1.9761	4.81E-02	5.63E-02
Proximal tubule bicarbonate reclamation	1748.6997		**2.5318**	0.0874	28.9555	2.40E-184	1.29E-182
Purine metabolism	170301.4779	**-0.1205**		0.0063	-19.1151	1.89E-81	1.04E-80
Pyrimidine metabolism	141147.9338	**-0.0876**		0.0090	-9.7612	1.65E-22	3.13E-22
Renal cell carcinoma	1021.3304	**-2.6118**		0.1336	-19.5473	4.35E-85	2.60E-84
Renin-angiotensin system	1.1143	**-2.5179**		0.3681	-6.8401	7.91E-12	1.24E-11
Replication, recombination and repair proteins	51425.1576	**-0.1841**		0.0167	-11.0187	3.11E-28	6.48E-28
Restriction enzyme	19681.9588		**0.3164**	0.0282	11.2149	3.45E-29	7.36E-29
Retinol metabolism	2361.4118	**-0.2660**		0.0709	-3.7536	1.74E-04	2.29E-04
Riboflavin metabolism	21547.8057	**-0.3660**		0.0231	-15.8660	1.09E-56	3.58E-56
Ribosome	189318.0805	**-0.2968**		0.0147	-20.2132	7.49E-91	4.91E-90
Ribosome Biogenesis	102636.9473	**-0.1325**		0.0136	-9.7252	2.35E-22	4.43E-22
Ribosome biogenesis in eukaryotes	3709.0169	**-0.3036**		0.0136	-22.3012	3.60E-110	3.58E-109
RIG-I-like receptor signaling pathway	92.5671	**-1.0646**		0.1736	-6.1342	8.56E-10	1.29E-09
RNA degradation	38591.8992	**-0.0583**		0.0118	-4.9561	7.19E-07	1.00E-06
RNA polymerase	11955.6986	**-0.3064**		0.0160	-19.1301	1.42E-81	7.95E-81
RNA transport	9788.1065	**-0.4062**		0.0265	-15.3198	5.64E-53	1.76E-52
Secondary bile acid biosynthesis	2763.7469		**1.9505**	0.0975	20.0116	4.36E-89	2.79E-88
Secretion system	89808.8247	**-0.7647**		0.0388	-19.7156	1.58E-86	9.92E-86
Selenocompound metabolism	28545.7807	**-0.2731**		0.0115	-23.7900	4.23E-125	6.70E-124
Sphingolipid metabolism	23628.8192		**1.9878**	0.0921	21.5824	2.63E-103	2.36E-102
Staphylococcus aureus infection	79.1311	**-1.6896**		0.1702	-9.9252	3.23E-23	6.17E-23
Starch and sucrose metabolism	75743.0178		**0.7296**	0.0480	15.2128	2.91E-52	8.89E-52
Steroid biosynthesis	35.7927		**0.8395**	0.4012	2.0927	3.64E-02	4.33E-02
Steroid hormone biosynthesis	2961.0028		**3.4298**	0.1192	28.7834	3.46E-182	1.55E-180
Stilbenoid, diarylheptanoid and gingerol biosynthesis	85.5824		**2.0549**	0.2237	9.1875	4.02E-20	7.21E-20
Streptomycin biosynthesis	26121.3522		**0.5576**	0.0247	22.5519	1.29E-112	1.39E-111
Styrene degradation	766.0815	**-0.6842**		0.1074	-6.3706	1.88E-10	2.88E-10
Sulfur metabolism	23027.4712		**0.1745**	0.0180	9.6848	3.50E-22	6.49E-22
Sulfur relay system	19453.6300	**-0.5942**		0.0370	-16.0438	6.32E-58	2.18E-57
Synthesis and degradation of ketone bodies	1564.3421	**-0.3989**		0.1065	-3.7473	1.79E-04	2.33E-04
Systemic lupus erythematosus	8.4547		**7.5588**	0.6357	11.8912	1.31E-32	3.13E-32
Taurine and hypotaurine metabolism	8980.7341		**0.2082**	0.0181	11.4884	1.51E-30	3.35E-30
Terpenoid backbone biosynthesis	45365.3694	**-0.2039**		0.0090	-22.6745	8.00E-114	8.97E-113
Tetracycline biosynthesis	10748.2910	**-0.5696**		0.0502	-11.3460	7.76E-30	1.70E-29
Thiamine metabolism	37716.6049	**-0.0576**		0.0107	-5.3856	7.22E-08	1.03E-07
Toluene degradation	8757.0864		**0.1678**	0.0533	3.1494	1.64E-03	2.08E-03
Transcription machinery	79313.8767		**0.3866**	0.0164	23.6203	2.38E-123	3.37E-122
Transcription related proteins	187.2968	**-0.3850**		0.1569	-2.4536	1.41E-02	1.75E-02
Translation factors	42778.8749	**-0.2070**		0.0114	-18.2310	2.93E-74	1.36E-73
Translation proteins	66903.2737	**-0.1855**		0.0115	-16.1669	8.64E-59	3.06E-58
Transporters	369430.0207	**-0.1130**		0.0352	-3.2108	1.32E-03	1.69E-03
Tropane, piperidine and pyridine alkaloid biosynthesis	9374.5242		**0.0324**	0.0105	3.0820	2.06E-03	2.60E-03
Tryptophan metabolism	8976.6491	**-0.4975**		0.0338	-14.7073	5.79E-49	1.67E-48
Tuberculosis	11388.2532		**0.0543**	0.0140	3.8893	1.01E-04	1.33E-04
Two-component system	104176.1231	**-0.4520**		0.0310	-14.5903	3.24E-48	9.26E-48
Type I diabetes mellitus	4621.5573		**0.2660**	0.0144	18.4608	4.27E-76	2.01E-75
Type II diabetes mellitus	3279.8666		**0.4810**	0.0755	6.3684	1.91E-10	2.90E-10
Tyrosine metabolism	23120.6344		**0.0399**	0.0088	4.5279	5.96E-06	8.09E-06
Ubiquitin system	739.3226		**4.2735**	0.1327	32.2124	1.18E-227	1.06E-225
Valine, leucine and isoleucine biosynthesis	53011.5814	**-0.0835**		0.0155	-5.3722	7.78E-08	1.11E-07
Valine, leucine and isoleucine degradation	14748.1503		**0.2860**	0.0412	6.9398	3.93E-12	6.25E-12

Various types of N-glycan biosynthesis	663.8285	**-11.5847**		0.2684	-43.1602	0.00E+00	0.00E+00
Vasopressin-regulated water reabsorption	0.0249		**2.5008**	1.0644	2.3495	1.88E-02	2.30E-02
Vibrio cholerae pathogenic cycle	6551.5342	**-0.8657**		0.0366	-23.6252	2.12E-123	3.17E-122
Vitamin B6 metabolism	15550.4852		**0.3170**	0.0228	13.8796	8.42E-44	2.36E-43
Xylene degradation	2642.5725		**0.5539**	0.0935	5.9231	3.16E-09	4.69E-09
Zeatin biosynthesis	4833.660469		**0.048570689**	0.0173	2.80179	5.08E-03	6.33E-03

Table S10. Sparse partial least squares discriminant analysis (sPLSDA) of predicted metabolic pathways discriminative between macaque and human vaginal microbiota.

annotation	baseMean	log2FoldChange _macaque	log2FoldChange _human	lfcSE	stat	pvalue	padj
1,1,1-Trichloro-2,2-bis(4-chlorophenyl)ethane (DDT) degradation	249.2986	**-3.9420**		0.3667	-10.7501	5.92E-27	2.32E-26
ABC transporters	196882.0431		**0.0969**	0.0266	3.6441	2.68E-04	3.75E-04
Adipocytokine signaling pathway	3101.7061	**-1.7798**		0.2220	-8.0190	1.07E-15	2.42E-15
African trypanosomiasis	175.4859		**0.7440**	0.3351	2.2198	2.64E-02	3.14E-02
Alanine, aspartate and glutamate metabolism	51115.7579	**-0.0447**		0.0158	-2.8293	4.67E-03	5.86E-03
Alzheimer's disease	4187.6704	**-0.4179**		0.0407	-10.2645	1.02E-24	3.62E-24
Amino acid metabolism	7296.4749	**-0.8682**		0.0636	-13.6497	2.03E-42	1.83E-41
Amino acid related enzymes	87272.4356	**-0.0498**		0.0164	-3.0413	2.36E-03	3.01E-03
Amino sugar and nucleotide sugar metabolism	82445.9433		**0.3822**	0.0260	14.6883	7.66E-49	9.00E-48
Aminoacyl-tRNA biosynthesis	85135.9054		**0.2151**	0.0227	9.4887	2.34E-21	6.51E-21
Aminobenzoate degradation	8281.1662		**0.6608**	0.0921	7.1725	7.36E-13	1.51E-12
Amoebiasis	537.0482	**-2.3087**		0.2476	-9.3234	1.13E-20	3.01E-20
Amyotrophic lateral sclerosis (ALS)	584.1475	**-1.6427**		0.2306	-7.1247	1.04E-12	2.12E-12
Antigen processing and presentation	1439.1920	**-2.1264**		0.2099	-10.1290	4.11E-24	1.35E-23
Apoptosis	468.2917		**3.9977**	0.4139	9.6581	4.54E-22	1.33E-21
Arachidonic acid metabolism	1547.6648	**-0.9833**		0.1829	-5.3758	7.63E-08	1.28E-07
Arginine and proline metabolism	43674.2916	**-0.6329**		0.0482	-13.1387	1.97E-39	1.44E-38
Ascorbate and aldarate metabolism	5991.0112	**-1.2100**		0.2023	-5.9817	2.21E-09	3.92E-09
Atrazine degradation	997.6583	**-1.4582**		0.2110	-6.9100	4.85E-12	9.48E-12
Bacterial chemotaxis	20514.3145	**-1.5935**		0.1325	-12.0271	2.56E-33	1.44E-32
Bacterial invasion of epithelial cells	218.6178	**-4.1377**		0.3920	-10.5566	4.74E-26	1.78E-25
Bacterial motility proteins	45311.5466	**-2.3559**		0.2107	-11.1829	4.94E-29	2.19E-28
Bacterial secretion system	38037.5948	**-0.3170**		0.0284	-11.1427	7.77E-29	3.38E-28
Bacterial toxins	7708.4229		**0.6347**	0.0482	13.1681	1.34E-39	1.06E-38
Basal transcription factors	90.9510	**-2.1537**		0.2869	-7.5076	6.02E-14	1.29E-13
Base excision repair	28463.1570		**0.3159**	0.0189	16.7227	8.95E-63	1.73E-61
Benzoate degradation	16017.3959		**0.5860**	0.0574	10.2162	1.68E-24	5.89E-24
beta-Alanine metabolism	7736.4324	**-1.2797**		0.1834	-6.9781	2.99E-12	5.94E-12
beta-Lactam resistance	1635.1816		**2.8912**	0.2382	12.1364	6.78E-34	3.98E-33
Betalain biosynthesis	8.4949		**6.2461**	0.6568	9.5105	1.90E-21	5.45E-21
Bile secretion	4.5070		**1.7929**	0.6646	2.6978	6.98E-03	8.65E-03
Biosynthesis and biodegradation of secondary metabolites	1611.3915	**-1.7207**		0.2081	-8.2667	1.38E-16	3.26E-16
Biosynthesis of 12-, 14- and 16-membered macrolides	4.6287		**6.2478**	1.0418	5.9972	2.01E-09	3.59E-09
Biosynthesis of ansamycins	4558.8248	**-1.6633**		0.1722	-9.6564	4.62E-22	1.34E-21
Biosynthesis of siderophore group nonribosomal peptides	741.7037	**-0.9176**		0.2228	-4.1188	3.81E-05	5.56E-05
Biosynthesis of type II polyketide products	8.3845		**2.8489**	0.5307	5.3683	7.95E-08	1.32E-07
Biosynthesis of unsaturated fatty acids	6153.4631		**0.2215**	0.0684	3.2361	1.21E-03	1.58E-03
Biosynthesis of vancomycin group antibiotics	2627.1010	**-0.4286**		0.1492	-2.8721	4.08E-03	5.14E-03
Biotin metabolism	6717.7987	**-1.8730**		0.1372	-13.6524	1.95E-42	1.82E-41
Bisphenol degradation	2377.6187	**-1.3684**		0.1889	-7.2449	4.33E-13	8.92E-13
Bladder cancer	55.0449	**-1.5379**		0.3078	-4.9961	5.85E-07	9.40E-07
Butanoate metabolism	31774.9797	**-0.2197**		0.0386	-5.6881	1.28E-08	2.20E-08
Butirosin and neomycin biosynthesis	2611.2464		**0.2749**	0.0923	2.9791	2.89E-03	3.67E-03
C5-Branched dibasic acid metabolism	7559.1875	**-1.7381**		0.1830	-9.4980	2.14E-21	6.02E-21
Carbohydrate metabolism	5960.9165		**0.3341**	0.0649	5.1451	2.67E-07	4.37E-07
Carbon fixation in photosynthetic organisms	36011.8509	**-0.0799**		0.0284	-2.8129	4.91E-03	6.11E-03
Carbon fixation pathways in prokaryotes	52753.6789	**-0.1047**		0.0209	-5.0194	5.18E-07	8.38E-07
Cardiac muscle contraction	932.4767	**-2.5358**		0.3227	-7.8578	3.91E-15	8.79E-15
Carotenoid biosynthesis	103.1801		**1.6251**	0.3914	4.1524	3.29E-05	4.83E-05
Cell cycle - Caulobacter	32489.8724		**0.0668**	0.0195	3.4286	6.07E-04	8.36E-04
Cell division	2299.5829	**-1.8463**		0.1834	-10.0682	7.64E-24	2.48E-23
Cellular antigens	2750.9582		**1.5284**	0.1297	11.7842	4.71E-32	2.36E-31
Chloroalkane and chloroalkene degradation	8371.6928	**-0.3993**		0.0632	-6.3179	2.65E-10	4.94E-10
Chromosome	93838.4943		**0.2162**	0.0165	13.1050	3.08E-39	2.13E-38
Circadian rhythm - plant	3.0409		**2.1887**	0.6931	3.1580	1.59E-03	2.05E-03
Citrate cycle (TCA cycle)	28719.3328	**-0.1526**		0.0426	-3.5812	3.42E-04	4.74E-04
Colorectal cancer	22.2968		**1.9104**	0.5757	3.3183	9.06E-04	1.21E-03
Cyanoamino acid metabolism	11287.7747		**0.1135**	0.0457	2.4823	1.31E-02	1.58E-02
Cysteine and methionine metabolism	46258.3312		**0.0922**	0.0231	3.9918	6.56E-05	9.32E-05
Cytochrome P450	2.2995		**3.5089**	1.1010	3.1870	1.44E-03	1.87E-03
Cytoskeleton proteins	18518.4410		**0.2078**	0.0293	7.0950	1.29E-12	2.61E-12
D-Alanine metabolism	9418.0337		**0.7534**	0.0384	19.6149	1.15E-85	7.79E-84
D-Arginine and D-ornithine metabolism	375.1035	**-2.9004**		0.2435	-11.9090	1.06E-32	5.52E-32
D-Glutamine and D-glutamate metabolism	10225.8888		**0.3016**	0.0293	10.2914	7.71E-25	2.77E-24
Dioxin degradation	6357.8949		**0.5809**	0.0760	7.6423	2.13E-14	4.61E-14
DNA repair and recombination proteins	184966.5389		**0.2849**	0.0182	15.6367	4.09E-55	5.82E-54
DNA replication	45144.7055		**0.2533**	0.0232	10.9274	8.52E-28	3.60E-27
DNA replication proteins	75150.4178		**0.2497**	0.0199	12.5296	5.14E-36	3.23E-35
Drug metabolism - cytochrome P450	1332.4536		**0.5927**	0.2466	2.4029	1.63E-02	1.95E-02
Drug metabolism - other enzymes	18392.7378		**0.2511**	0.0302	8.3228	8.59E-17	2.07E-16
Electron transfer carriers	1865.9453	**-2.9136**		0.2468	-11.8045	3.70E-32	1.88E-31
Endocytosis	6.6907		**6.0943**	0.9701	6.2821	3.34E-10	6.13E-10
Energy metabolism	37101.3987	**-0.6301**		0.0401	-15.7161	1.17E-55	1.76E-54

Epithelial cell signaling in Helicobacter pylori infection	3037.7651	**-2.0870**		0.1953	-10.6841	1.21E-26	4.66E-26
Ethylbenzene degradation	2158.2189		**0.6568**	0.1379	4.7631	1.91E-06	3.03E-06
Fatty acid metabolism	14470.7253	**-0.1875**		0.0738	-2.5424	1.10E-02	1.36E-02
Fc gamma R-mediated phagocytosis	6.7138		**5.9576**	0.9505	6.2679	3.66E-10	6.68E-10
Flagellar assembly	20566.2710	**-2.6943**		0.2335	-11.5399	8.30E-31	3.93E-30
Flavone and flavonol biosynthesis	265.4098		**1.5492**	0.3429	4.5180	6.24E-06	9.36E-06
Flavonoid biosynthesis	280.2592	**-2.1806**		0.2424	-8.9960	2.34E-19	5.85E-19
Fluorobenzoate degradation	265.3818		**3.3913**	0.5244	6.4668	1.00E-10	1.92E-10
Folate biosynthesis	20657.3481	**-0.6226**		0.0365	-17.0641	2.74E-65	5.70E-64
Fructose and mannose metabolism	48320.6472		**0.5060**	0.0394	12.8416	9.59E-38	6.32E-37
Function unknown	77105.4493		**0.1426**	0.0235	6.0624	1.34E-09	2.41E-09
Galactose metabolism	37091.9474		**0.8834**	0.0529	16.7105	1.10E-62	1.98E-61
Germination	1300.3151	**-3.6785**		0.3625	-10.1463	3.44E-24	1.16E-23
Glutamatergic synapse	4663.0208		**0.3891**	0.0472	8.2398	1.73E-16	4.05E-16
Glutathione metabolism	13941.5417		**0.3349**	0.0339	9.8722	5.49E-23	1.71E-22
Glycan biosynthesis and metabolism	1844.6685	**-2.1103**		0.1955	-10.7930	3.71E-27	1.47E-26
Glycerolipid metabolism	21166.7234		**0.4751**	0.0362	13.1106	2.86E-39	2.03E-38
Glycerophospholipid metabolism	32634.0568		**0.1858**	0.0240	7.7409	9.87E-15	2.17E-14
Glycine, serine and threonine metabolism	35741.9202	**-0.3627**		0.0317	-11.4341	2.83E-30	1.27E-29
Glycolysis / Gluconeogenesis	70299.3951		**0.6908**	0.0281	24.6249	6.84E-134	1.85E-131
Glycosphingolipid biosynthesis - ganglio series	513.6414	**-1.0213**		0.2434	-4.1964	2.71E-05	4.00E-05
Glycosphingolipid biosynthesis - globo series	2372.3340		**0.6641**	0.1250	5.3116	1.09E-07	1.80E-07
Glycosphingolipid biosynthesis - lacto and neolacto series	0.4714		**3.4642**	1.5665	2.2114	2.70E-02	3.20E-02
Glycosyltransferases	24259.3328		**0.4769**	0.0415	11.4904	1.47E-30	6.86E-30
Glyoxylate and dicarboxylate metabolism	20769.3526	**-0.5753**		0.0585	-9.8360	7.88E-23	2.42E-22
GnRH signaling pathway	6.6907		**6.0943**	0.9701	6.2821	3.34E-10	6.13E-10
Hematopoietic cell lineage	2.2266		**4.1322**	1.2622	3.2737	1.06E-03	1.40E-03
Histidine metabolism	20815.0865	**-1.1727**		0.0687	-17.0690	2.52E-65	5.68E-64
Homologous recombination	62290.2802		**0.1933**	0.0239	8.0963	5.67E-16	1.30E-15
Huntington's disease	2396.2995	**-2.3703**		0.1962	-12.0816	1.32E-33	7.59E-33
Hypertrophic cardiomyopathy (HCM)	6.4324		**3.6551**	0.7854	4.6539	3.26E-06	5.09E-06
Indole alkaloid biosynthesis	4.5914		**5.6073**	0.9403	5.9630	2.48E-09	4.37E-09
Influenza A	22.2968		**1.9104**	0.5757	3.3183	9.06E-04	1.21E-03
Inorganic ion transport and metabolism	7929.9323	**-0.5896**		0.0716	-8.2347	1.80E-16	4.19E-16
Inositol phosphate metabolism	5306.8592	**-0.2367**		0.0699	-3.3881	7.04E-04	9.64E-04
Insulin signaling pathway	3066.2989	**-1.1535**		0.1554	-7.4240	1.14E-13	2.38E-13
Ion channels	2339.3268		**1.6290**	0.1203	13.5412	8.94E-42	7.78E-41
Isoflavonoid biosynthesis	3.6637		**1.9129**	0.5875	3.2561	1.13E-03	1.48E-03
Isoquinoline alkaloid biosynthesis	1971.8969	**-1.7594**		0.2044	-8.6094	7.35E-18	1.82E-17
Linoleic acid metabolism	1648.7461	**-1.7591**		0.1877	-9.3709	7.19E-21	1.94E-20
Lipid metabolism	9821.4034		**0.6975**	0.0753	9.2650	1.95E-20	5.16E-20
Lipoic acid metabolism	1164.0466	**-1.5348**		0.1809	-8.4824	2.21E-17	5.37E-17
Lipopolysaccharide biosynthesis	13759.4911	**-2.3069**		0.1981	-11.6432	2.49E-31	1.22E-30
Lipopolysaccharide biosynthesis proteins	18806.5912	**-1.5824**		0.0918	-17.2316	1.54E-66	3.78E-65
Lysine biosynthesis	37504.3947		**0.2824**	0.0461	6.1219	9.25E-10	1.68E-09
Lysine degradation	8016.8868	**-0.6332**		0.0989	-6.4002	1.55E-10	2.91E-10
Lysosome	1509.3393	**-0.6812**		0.2077	-3.2795	1.04E-03	1.38E-03
MAPK signaling pathway - yeast	1260.8601	**-2.1982**		0.1837	-11.9667	5.31E-33	2.81E-32
Meiosis - yeast	230.4089	**-0.7333**		0.2955	-2.4819	1.31E-02	1.58E-02
Melanogenesis	1.8052		**5.3957**	1.2787	4.2196	2.45E-05	3.63E-05
Membrane and intracellular structural molecules	23049.9670	**-0.6621**		0.0552	-11.9923	3.90E-33	2.11E-32
Metabolism of xenobiotics by cytochrome P450	1299.3802		**0.5481**	0.2451	2.2366	2.53E-02	3.02E-02
Methane metabolism	63010.6708	**-0.0357**		0.0176	-2.0330	4.21E-02	4.92E-02
Mineral absorption	230.8990	**-1.7049**		0.2587	-6.5903	4.39E-11	8.46E-11
Mismatch repair	54569.3228		**0.2145**	0.0214	10.0379	1.04E-23	3.34E-23
mRNA surveillance pathway	2.0983		**5.9021**	1.2683	4.6535	3.26E-06	5.09E-06
N-Glycan biosynthesis	1417.5369	**-2.8634**		0.2346	-12.2065	2.87E-34	1.72E-33
Naphthalene degradation	7791.1254		**0.3617**	0.0519	6.9714	3.14E-12	6.19E-12
Nitrogen metabolism	35677.5945	**-0.4666**		0.0325	-14.3526	1.03E-46	1.11E-45
Nitrotoluene degradation	2414.6842	**-2.3840**		0.2196	-10.8549	1.89E-27	7.73E-27
NOD-like receptor signaling pathway	1564.1758	**-1.7434**		0.2146	-8.1245	4.49E-16	1.04E-15
Non-homologous end-joining	393.8287	**-1.2099**		0.2860	-4.2301	2.34E-05	3.48E-05
Novobiocin biosynthesis	4568.8695	**-1.7526**		0.1860	-9.4218	4.43E-21	1.21E-20
Nucleotide excision repair	27059.5306		**0.2422**	0.0223	10.8644	1.70E-27	7.08E-27
Nucleotide metabolism	4766.0830		**0.5258**	0.0612	8.5941	8.39E-18	2.06E-17
One carbon pool by folate	34999.0478	**-0.0898**		0.0223	-4.0323	5.52E-05	7.89E-05
Other glycan degradation	5345.2256		**0.4131**	0.1253	3.2966	9.79E-04	1.30E-03
Other ion-coupled transporters	67454.6871		**0.2197**	0.0281	7.8075	5.83E-15	1.29E-14
Other transporters	11130.4761	**-0.7204**		0.0378	-19.0703	4.46E-81	2.01E-79
Others	55591.9762		**0.3625**	0.0370	9.7962	1.17E-22	3.55E-22
Oxidative phosphorylation	54972.9372	**-0.1587**		0.0246	-6.4369	1.22E-10	2.32E-10
p53 signaling pathway	23.3664		**1.6398**	0.5810	2.8221	4.77E-03	5.96E-03
Pantothenate and CoA biosynthesis	30098.5085	**-0.4451**		0.0255	-17.4241	5.41E-68	1.62E-66
Parkinson's disease	960.9605	**-2.3678**		0.3200	-7.4000	1.36E-13	2.83E-13
Penicillin and cephalosporin biosynthesis	1923.2351		**1.8586**	0.1070	17.3712	1.36E-67	3.68E-66
Pentose and glucuronate interconversions	15698.0554	**-0.2729**		0.0504	-5.4158	6.10E-08	1.03E-07
Pentose phosphate pathway	46644.1707		**0.2998**	0.0234	12.8115	1.41E-37	9.08E-37

Peptidases	113405.3092		**0.2750**	0.0168	16.4103	1.61E-60	2.56E-59
Peptidoglycan biosynthesis	52486.4723		**0.1033**	0.0221	4.6647	3.09E-06	4.88E-06
Peroxisome	7651.9918	**-0.7587**		0.0661	-11.4800	1.66E-30	7.61E-30
Pertussis	902.8811	**-1.0014**		0.2620	-3.8227	1.32E-04	1.87E-04
Phenylalanine metabolism	6385.4016	**-1.2012**		0.1802	-6.6674	2.60E-11	5.06E-11
Phenylalanine, tyrosine and tryptophan biosynthesis	27733.1232	**-1.6840**		0.1772	-9.5014	2.07E-21	5.88E-21
Phenylpropanoid biosynthesis	2971.6744	**-0.4449**		0.1803	-2.4679	1.36E-02	1.64E-02
Phosphatidylinositol signaling system	4732.4937	**-0.3655**		0.0406	-9.0140	1.99E-19	5.02E-19
Phosphonate and phosphinate metabolism	1006.3621	**-0.9911**		0.2069	-4.7911	1.66E-06	2.65E-06
Phosphotransferase system (PTS)	51881.6452		**1.5455**	0.1101	14.0373	9.21E-45	9.21E-44
Photosynthesis	23258.4618		**0.3947**	0.0382	10.3228	5.56E-25	2.03E-24
Photosynthesis proteins	23359.5436		**0.3908**	0.0376	10.3799	3.06E-25	1.13E-24
Plant-pathogen interaction	8400.0904	**-0.3698**		0.0392	-9.4248	4.31E-21	1.19E-20
Pores ion channels	15912.5331	**-0.9121**		0.0652	-13.9862	1.89E-44	1.83E-43
Porphyrin and chlorophyll metabolism	32826.3261	**-2.4063**		0.1615	-14.9017	3.21E-50	3.94E-49
PPAR signaling pathway	5702.3020	**-0.4034**		0.0718	-5.6211	1.90E-08	3.22E-08
Prenyltransferases	22481.1725		**0.3171**	0.0311	10.1974	2.04E-24	7.05E-24
Primary bile acid biosynthesis	1495.0117		**3.0483**	0.2286	13.3328	1.49E-40	1.26E-39
Primary immunodeficiency	4297.1463		**0.9838**	0.0517	19.0382	8.24E-81	3.18E-79
Prion diseases	345.4689	**-2.7353**		0.2533	-10.7989	3.49E-27	1.40E-26
Progesterone-mediated oocyte maturation	1439.1920	**-2.1264**		0.2099	-10.1290	4.11E-24	1.35E-23
Propanoate metabolism	28964.5312		**0.2146**	0.0471	4.5551	5.24E-06	7.99E-06
Prostate cancer	1439.9848	**-2.1223**		0.2089	-10.1614	2.95E-24	1.01E-23
Proteasome	1668.4476	**-2.0003**		0.2068	-9.6740	3.89E-22	1.15E-21
Protein digestion and absorption	631.5176	**-1.3161**		0.2863	-4.5978	4.27E-06	6.55E-06
Protein folding and associated processing	35257.0887	**-0.8409**		0.0440	-19.1164	1.85E-81	9.97E-80
Protein kinases	13433.9513	**-0.2686**		0.0472	-5.6935	1.24E-08	2.14E-08
Protein processing in endoplasmic reticulum	3452.7937	**-0.2993**		0.0734	-4.0757	4.59E-05	6.66E-05
Proximal tubule bicarbonate reclamation	1205.9908		**0.9182**	0.1994	4.6053	4.12E-06	6.35E-06
Purine metabolism	149746.3174		**0.2185**	0.0221	9.9063	3.91E-23	1.23E-22
Pyrimidine metabolism	120659.8541		**0.2525**	0.0218	11.5863	4.83E-31	2.33E-30
Pyruvate metabolism	53477.5959		**0.1527**	0.0262	5.8390	5.25E-09	9.15E-09
Renal cell carcinoma	1787.5175		**1.4594**	0.0956	15.2628	1.35E-52	1.83E-51
Renin-angiotensin system	9.7726		**2.6411**	0.6529	4.0453	5.23E-05	7.51E-05
Replication, recombination and repair proteins	50139.8014		**0.1161**	0.0251	4.6197	3.84E-06	5.96E-06
Riboflavin metabolism	14286.7731	**-0.1724**		0.0578	-2.9814	2.87E-03	3.65E-03
Ribosome	165710.6286		**0.1577**	0.0246	6.4068	1.49E-10	2.81E-10
Ribosome Biogenesis	89396.5648		**0.1400**	0.0179	7.8320	4.80E-15	1.07E-14
Ribosome biogenesis in eukaryotes	3447.6581		**0.1209**	0.0266	4.5437	5.53E-06	8.38E-06
RIG-I-like receptor signaling pathway	238.0842	**-1.0243**		0.3251	-3.1503	1.63E-03	2.10E-03
RNA degradation	26216.0005	**-0.1396**		0.0188	-7.4316	1.07E-13	2.26E-13
RNA polymerase	13512.9123		**0.4228**	0.0326	12.9855	1.48E-38	9.98E-38
RNA transport	9687.7594		**0.5180**	0.0523	9.9067	3.89E-23	1.23E-22
Secondary bile acid biosynthesis	1427.1812		**3.0621**	0.2329	13.1494	1.72E-39	1.29E-38
Secretion system	82406.4489	**-0.1829**		0.0350	-5.2208	1.78E-07	2.93E-07
Signal transduction mechanisms	29978.1736		**0.6362**	0.0347	18.3146	6.33E-75	2.13E-73
Small cell lung cancer	22.2968		**1.9104**	0.5757	3.3183	9.06E-04	1.21E-03
Sphingolipid metabolism	5395.5972		**1.2070**	0.1327	9.0973	9.26E-20	2.36E-19
Sporulation	14845.4903	**-2.6465**		0.2718	-9.7368	2.10E-22	6.30E-22
Staphylococcus aureus infection	5171.2927		**4.2828**	0.1930	22.1882	4.46E-109	6.02E-107
Starch and sucrose metabolism	48917.2820		**0.6684**	0.0404	16.5555	1.46E-61	2.46E-60
Steroid biosynthesis	23.4957		**2.1816**	0.5932	3.6779	2.35E-04	3.31E-04
Steroid hormone biosynthesis	167.2007		**0.6568**	0.3150	2.0853	3.70E-02	4.35E-02
Styrene degradation	1181.6842	**-0.8926**		0.2482	-3.5965	3.23E-04	4.49E-04
Sulfur relay system	15550.4975	**-0.2480**		0.0332	-7.4705	7.99E-14	1.70E-13
Synthesis and degradation of ketone bodies	4409.3382		**1.4705**	0.1118	13.1563	1.57E-39	1.21E-38
Systemic lupus erythematosus	2.0391		**3.7581**	0.8315	4.5199	6.19E-06	9.33E-06
Taurine and hypotaurine metabolism	7820.9787		**0.8100**	0.0390	20.7540	1.13E-95	1.02E-93
Terpenoid backbone biosynthesis	38210.3404		**0.2237**	0.0186	12.0055	3.32E-33	1.83E-32
Thiamine metabolism	29855.9122	**-0.1480**		0.0250	-5.9310	3.01E-09	5.28E-09
Toluene degradation	6775.2348		**0.6822**	0.0744	9.1691	4.77E-20	1.23E-19
Toxoplasmosis	22.2968		**1.9104**	0.5757	3.3183	9.06E-04	1.21E-03
Transcription factors	81527.9922		**0.3965**	0.0321	12.3339	5.95E-35	3.65E-34
Transcription machinery	46138.9457	**-0.1613**		0.0209	-7.7139	1.22E-14	2.66E-14
Translation factors	36607.6886		**0.2022**	0.0244	8.2849	1.18E-16	2.82E-16
Translation proteins	52712.3585	**-0.2101**		0.0191	-11.0167	3.18E-28	1.36E-27
Transporters	394511.2901		**0.4044**	0.0276	14.6536	1.28E-48	1.44E-47
Tropane, piperidine and pyridine alkaloid biosynthesis	3877.3685	**-1.6440**		0.1782	-9.2234	2.88E-20	7.47E-20
Tuberculosis	9130.1199		**0.3070**	0.0216	14.2259	6.33E-46	6.57E-45
Two-component system	71906.3533	**-0.1301**		0.0518	-2.5108	1.20E-02	1.48E-02
Type I diabetes mellitus	3251.6970		**0.0862**	0.0213	4.0514	5.09E-05	7.35E-05
Type II diabetes mellitus	2938.4184		**0.3754**	0.0353	10.6310	2.14E-26	8.13E-26
Tyrosine metabolism	19190.2193		**0.0583**	0.0272	2.1441	3.20E-02	3.78E-02
Ubiquinone and other terpenoid-quinone biosynthesis	9706.5120		**0.2170**	0.0867	2.5016	1.24E-02	1.51E-02
Ubiquitin system	418.8692	**-1.2449**		0.2480	-5.0194	5.18E-07	8.38E-07
Valine, leucine and isoleucine biosynthesis	24918.7581	**-0.8605**		0.0575	-14.9545	1.46E-50	1.87E-49
Various types of N-glycan biosynthesis	294.3353	**-5.2925**		0.3978	-13.3047	2.17E-40	1.78E-39

Vibrio cholerae pathogenic cycle	3982.9419	**-0.3885**		0.0549	-7.0717	1.53E-12	3.06E-12
Viral myocarditis	22.2968		**1.9104**	0.5757	3.3183	9.06E-04	1.21E-03
Vitamin B6 metabolism	8767.8823	**-0.5016**		0.0542	-9.2593	2.06E-20	5.39E-20
Xylene degradation	4211.4364		**0.4515**	0.0791	5.7047	1.17E-08	2.02E-08

Table S11. NSTI score of each sample predicted using PICRUSt.

#Sample	NSTI Value	Host	Site
PM001S2M1	0.0951141984995	Macaque	Anal
PM002S2M1	0.0969671122208	Macaque	Anal
PM003S2M1	0.0969934026514	Macaque	Anal
PM004S2M1	0.0908952967922	Macaque	Anal
PM005S2M1	0.0547937876961	Macaque	Anal
PM006S2M1	0.0625174149946	Macaque	Anal
PM007S2M1	0.0469267161951	Macaque	Anal
PM008S2M1	0.0873646435398	Macaque	Anal
PM009S2M1	0.0572835812280	Macaque	Anal
PM010S2M1	0.0994358347061	Macaque	Anal
PM011S2M1	0.0546193227085	Macaque	Anal
PM012S2M1	0.0912925669967	Macaque	Anal
PM013S2M1	0.0911383763400	Macaque	Anal
PM014S2M1	0.0663773215677	Macaque	Anal
PM015S2M1	0.0556658912098	Macaque	Anal
PM016S2M1	0.0876184982896	Macaque	Anal
PM017S2M1	0.0551516303421	Macaque	Anal
PM018S2M1	0.1178078943420	Macaque	Anal
PM019S2M1	0.1904718505810	Macaque	Anal
PM020S2M1	0.0472581824076	Macaque	Anal
PM021S2M1	0.0500212582560	Macaque	Anal
PM022S2M1	0.0601327228476	Macaque	Anal
PM023S2M1	0.1736130888150	Macaque	Anal
PM024S2M1	0.0643832039241	Macaque	Anal
PM025S2M1	0.0497193351886	Macaque	Anal
PM026S2M1	0.0506260902951	Macaque	Anal
PM027S2M1	0.0498137470788	Macaque	Anal
PM028S2M1	0.1137135563440	Macaque	Anal
PM029S2M1	0.0547605809505	Macaque	Anal
PM030S2M1	0.0527783605024	Macaque	Anal
PM031S2M1	0.0674984750009	Macaque	Anal
PM032S2M1	0.1243455735750	Macaque	Anal
PM033S2M1	0.0740172473354	Macaque	Anal
PM034S2M1	0.0508941824457	Macaque	Anal
PM035S2M1	0.0519566733240	Macaque	Anal
PM036S2M1	0.0549623226855	Macaque	Anal
PM037S2M1	0.0880563406401	Macaque	Anal
PM038S2M1	0.0593588697116	Macaque	Anal
PM039S2M1	0.0488386463783	Macaque	Anal
PM040S2M1	0.0995168804807	Macaque	Anal
PM041S2M1	0.1158075624860	Macaque	Anal
PM042S2M1	0.0801135339879	Macaque	Anal
PM043S2M1	0.0473347314581	Macaque	Anal
PM044S2M1	0.0798127169676	Macaque	Anal
PM045S2M1	0.0759816668700	Macaque	Anal

PM046S2M1	0.1472600477630	Macaque	Anal
PM047S2M1	0.0912910508648	Macaque	Anal
PM048S2M1	0.0575220648844	Macaque	Anal
PM049S2M1	0.0513337370726	Macaque	Anal
PM050S2M1	0.0808634698503	Macaque	Anal
PM051S2M1	0.1411963703340	Macaque	Anal
PM052S2M1	0.0852188689451	Macaque	Anal
PM053S2M1	0.0923599373293	Macaque	Anal
PM054S2M1	0.1342469254810	Macaque	Anal
PM055S2M1	0.0495443636965	Macaque	Anal
PM056S2M1	0.0767262807828	Macaque	Anal
PM057S2M1	0.0968266988546	Macaque	Anal
PM058S2M1	0.0843098111606	Macaque	Anal
PM059S2M1	0.1260509109670	Macaque	Anal
PM060S2M1	0.1303557916230	Macaque	Anal
PM061S2M1	0.0494583831954	Macaque	Anal
PM062S2M1	0.0974095735023	Macaque	Anal
PM063S2M1	0.0959163812150	Macaque	Anal
PM064S2M1	0.1100720783540	Macaque	Anal
PM065S2M1	0.0636786335664	Macaque	Anal
PM066S2M1	0.0754774010021	Macaque	Anal
PM067S2M1	0.0989609936609	Macaque	Anal
PM068S2M1	0.0883670913658	Macaque	Anal
PM069S2M1	0.1620364382750	Macaque	Anal
PM070S2M1	0.0390245439827	Macaque	Anal
PM071S2M1	0.0713957801156	Macaque	Anal
PM072S2M1	0.0944942024382	Macaque	Anal
PM073S2M1	0.1070606383180	Macaque	Anal
PM074S2M1	0.0889987566889	Macaque	Anal
PM075S2M1	0.1452754003040	Macaque	Anal
PM076S2M1	0.0897341460758	Macaque	Anal
PM077S2M1	0.1163098327190	Macaque	Anal
PM078S2M1	0.1171858891730	Macaque	Anal
PM079S2M1	0.0625710798525	Macaque	Anal
PM080S2M1	0.1390586155000	Macaque	Anal
PM081S2M1	0.1481160136860	Macaque	Anal
PM082S2M1	0.1027456952090	Macaque	Anal
PM083S2M1	0.0951402953724	Macaque	Anal
PM084S2M1	0.1630740482900	Macaque	Anal
PM085S2M1	0.0692182229515	Macaque	Anal
PM086S2M1	0.0898839878021	Macaque	Anal
PM087S2M1	0.0650352963632	Macaque	Anal
PM088S2M1	0.0604824267988	Macaque	Anal
PM089S2M1	0.1202386465730	Macaque	Anal
PM090S2M1	0.0567697799788	Macaque	Anal
PM091S2M1	0.0571033383282	Macaque	Anal
PM092S2M1	0.0676028216490	Macaque	Anal
PM093S2M1	0.0649840190455	Macaque	Anal

PM094S2M1	0.0660040235537	Macaque	Anal
PM095S2M1	0.0988091740361	Macaque	Anal
PM096S2M1	0.0592924824787	Macaque	Anal
PM097S2M1	0.0583837320380	Macaque	Anal
PM098S2M1	0.0848337669045	Macaque	Anal
PM099S2M1	0.0679654580712	Macaque	Anal
PM100S2M1	0.0971694744419	Macaque	Anal
PM101S2M1	0.0506383951579	Macaque	Anal
PM102S2M1	0.0799332941518	Macaque	Anal
PM103S2M1	0.0888403378623	Macaque	Anal
PM104S2M1	0.0879562066607	Macaque	Anal
PM105S2M1	0.0998178704174	Macaque	Anal
PM106S2M1	0.0970221140561	Macaque	Anal
PM107S2M1	0.0676668570085	Macaque	Anal
PM108S2M1	0.0850649626131	Macaque	Anal
PM109S2M1	0.0798980445819	Macaque	Anal
PM110S2M1	0.0822787262621	Macaque	Anal
PM111S2M1	0.1008770587090	Macaque	Anal
PM112S2M1	0.1007699727270	Macaque	Anal
PM113S2M1	0.0827830236911	Macaque	Anal
PM114S2M1	0.1405311329020	Macaque	Anal
PM115S2M1	0.0843927017258	Macaque	Anal
PM116S2M1	0.1137816728870	Macaque	Anal
PM117S2M1	0.0988767625371	Macaque	Anal
PM001S1M1	0.0240105559301	Macaque	Oral
PM002S1M1	0.0406021335587	Macaque	Oral
PM003S1M1	0.0832476466620	Macaque	Oral
PM004S1M1	0.0427054907027	Macaque	Oral
PM005S1M1	0.2739393641520	Macaque	Oral
PM006S1M1	0.0323021252275	Macaque	Oral
PM007S1M1	0.0488953618389	Macaque	Oral
PM008S1M1	0.0321159540646	Macaque	Oral
PM009S1M1	0.0463458374086	Macaque	Oral
PM010S1M1	0.0274470352927	Macaque	Oral
PM011S1M1	0.0227051419150	Macaque	Oral
PM012S1M1	0.0553309094023	Macaque	Oral
PM013S1M1	0.0300563206025	Macaque	Oral
PM014S1M1	0.0419230216207	Macaque	Oral
PM015S1M1	0.1050568896340	Macaque	Oral
PM016S1M1	0.0596893429143	Macaque	Oral
PM017S1M1	0.0630312251927	Macaque	Oral
PM018S1M1	0.0381270738062	Macaque	Oral
PM019S1M1	0.0462241207456	Macaque	Oral
PM020S1M1	0.0474211144174	Macaque	Oral
PM021S1M1	0.0408012946285	Macaque	Oral
PM022S1M1	0.0273158915189	Macaque	Oral
PM023S1M1	0.0407425382352	Macaque	Oral
PM024S1M1	0.0267092305450	Macaque	Oral

PM025S1M1	0.0318499929825	Macaque	Oral
PM026S1M1	0.0304256938239	Macaque	Oral
PM027S1M1	0.0228074208414	Macaque	Oral
PM028S1M1	0.0197509148968	Macaque	Oral
PM029S1M1	0.0200408368655	Macaque	Oral
PM030S1M1	0.0375812254075	Macaque	Oral
PM031S1M1	0.0365608246924	Macaque	Oral
PM032S1M1	0.0252085027497	Macaque	Oral
PM033S1M1	0.0363409107836	Macaque	Oral
PM034S1M1	0.0199961061651	Macaque	Oral
PM035S1M1	0.0348156276629	Macaque	Oral
PM036S1M1	0.0264178726914	Macaque	Oral
PM037S1M1	0.0410905462550	Macaque	Oral
PM038S1M1	0.0251874381546	Macaque	Oral
PM039S1M1	0.0277906310880	Macaque	Oral
PM040S1M1	0.0286372576167	Macaque	Oral
PM041S1M1	0.0320921383402	Macaque	Oral
PM042S1M1	0.0270852099638	Macaque	Oral
PM043S1M1	0.0294841048998	Macaque	Oral
PM044S1M1	0.1289750269750	Macaque	Oral
PM045S1M1	0.0530441413810	Macaque	Oral
PM046S1M1	0.0220725881457	Macaque	Oral
PM047S1M1	0.0497312204028	Macaque	Oral
PM048S1M1	0.0390618735027	Macaque	Oral
PM049S1M1	0.0360372651178	Macaque	Oral
PM050S1M1	0.0412264667211	Macaque	Oral
PM051S1M1	0.0336797661427	Macaque	Oral
PM052S1M1	0.0202548629867	Macaque	Oral
PM053S1M1	0.0288116123145	Macaque	Oral
PM054S1M1	0.0453302983557	Macaque	Oral
PM055S1M1	0.0261451079768	Macaque	Oral
PM056S1M1	0.0371010935068	Macaque	Oral
PM057S1M1	0.2579386546670	Macaque	Oral
PM058S1M1	0.0206227182404	Macaque	Oral
PM059S1M1	0.0305740091541	Macaque	Oral
PM060S1M1	0.0294120149739	Macaque	Oral
PM061S1M1	0.0328429225921	Macaque	Oral
PM062S1M1	0.0394215187110	Macaque	Oral
PM063S1M1	0.0549916557530	Macaque	Oral
PM064S1M1	0.0555960467899	Macaque	Oral
PM065S1M1	0.0653834552412	Macaque	Oral
PM066S1M1	0.0402487681180	Macaque	Oral
PM067S1M1	0.0242496713401	Macaque	Oral
PM068S1M1	0.0251881274475	Macaque	Oral
PM069S1M1	0.0408538875836	Macaque	Oral
PM070S1M1	0.0191034315415	Macaque	Oral
PM071S1M1	0.0399659221536	Macaque	Oral
PM072S1M1	0.0319518844445	Macaque	Oral

PM073S1M1	0.0200919313244	Macaque	Oral
PM074S1M1	0.0147987594738	Macaque	Oral
PM075S1M1	0.0335395030409	Macaque	Oral
PM076S1M1	0.0351976551901	Macaque	Oral
PM077S1M1	0.0468477925027	Macaque	Oral
PM078S1M1	0.0246943781484	Macaque	Oral
PM079S1M1	0.0226454884592	Macaque	Oral
PM080S1M1	0.0233085585524	Macaque	Oral
PM081S1M1	0.0319158567430	Macaque	Oral
PM082S1M1	0.0723855076890	Macaque	Oral
PM083S1M1	0.0470011146125	Macaque	Oral
PM084S1M1	0.0254448309976	Macaque	Oral
PM085S1M1	0.2436342864860	Macaque	Oral
PM086S1M1	0.0673395198911	Macaque	Oral
PM087S1M1	0.0331258638556	Macaque	Oral
PM088S1M1	0.0507134481721	Macaque	Oral
PM089S1M1	0.0346708959615	Macaque	Oral
PM090S1M1	0.0245999609476	Macaque	Oral
PM091S1M1	0.0552611655482	Macaque	Oral
PM092S1M1	0.0314207588169	Macaque	Oral
PM093S1M1	0.0529944848948	Macaque	Oral
PM094S1M1	0.0647165772266	Macaque	Oral
PM095S1M1	0.0397466547937	Macaque	Oral
PM096S1M1	0.0889030320489	Macaque	Oral
PM097S1M1	0.0240157729075	Macaque	Oral
PM098S1M1	0.0683806177950	Macaque	Oral
PM099S1M1	0.0542043473738	Macaque	Oral
PM100S1M1	0.0560263176158	Macaque	Oral
PM101S1M1	0.0453606725356	Macaque	Oral
PM102S1M1	0.0310876219967	Macaque	Oral
PM103S1M1	0.0299448142435	Macaque	Oral
PM104S1M1	0.0616764991272	Macaque	Oral
PM105S1M1	0.0237331698852	Macaque	Oral
PM106S1M1	0.0172199488824	Macaque	Oral
PM107S1M1	0.0755909859204	Macaque	Oral
PM108S1M1	0.1117966084970	Macaque	Oral
PM109S1M1	0.3715983008070	Macaque	Oral
PM110S1M1	0.1395016269620	Macaque	Oral
PM111S1M1	0.0344972666977	Macaque	Oral
PM112S1M1	0.0233633920993	Macaque	Oral
PM113S1M1	0.0434869387191	Macaque	Oral
PM114S1M1	0.0371082712973	Macaque	Oral
PM115S1M1	0.0390174569274	Macaque	Oral
PM116S1M1	0.0309832675765	Macaque	Oral
PM117S1M1	0.0356407552976	Macaque	Oral
SRS011159	0.0501743041973	Human	Saliva
SRS011273	0.0301808441869	Human	Saliva
SRS011407	0.0208663044871	Human	Saliva

SRS011417	0.0259780450012	Human	Saliva
SRS011454	0.0307280032451	Human	Saliva
SRS011482	0.0306999615130	Human	Saliva
SRS011504	0.0525552312854	Human	Saliva
SRS011531	0.0368225702502	Human	Saliva
SRS011588	0.0500232396396	Human	Saliva
SRS011623	0.0675793336130	Human	Saliva
SRS011655	0.0507948683998	Human	Saliva
SRS013185	0.0605180075204	Human	Saliva
SRS013228	0.0177097175853	Human	Saliva
SRS013456	0.0465236122811	Human	Saliva
SRS013498	0.0244938400486	Human	Saliva
SRS013551	0.0443227712751	Human	Saliva
SRS013699	0.0928381017141	Human	Saliva
SRS014556	0.0317212360907	Human	Saliva
SRS014803	0.0293877402896	Human	Saliva
SRS014857	0.0450330362546	Human	Saliva
SRS014950	0.0291648817718	Human	Saliva
SRS015113	0.0736809729201	Human	Saliva
SRS015170	0.0764729806269	Human	Saliva
SRS015227	0.0248292131287	Human	Saliva
SRS015293	0.0217696603255	Human	Saliva
SRS015454	0.1359032541670	Human	Saliva
SRS015556	0.0227975024891	Human	Saliva
SRS015665	0.0423455451341	Human	Saliva
SRS015704	0.0396040746843	Human	Saliva
SRS015758	0.0568789131624	Human	Saliva
SRS015817	0.0408320294661	Human	Saliva
SRS015998	0.0332924333520	Human	Saliva
SRS016170	0.0382643956513	Human	Saliva
SRS016221	0.0780538830108	Human	Saliva
SRS016285	0.1352126962030	Human	Saliva
SRS016408	0.0298414218888	Human	Saliva
SRS016449	0.0367001824548	Human	Saliva
SRS016642	0.0343307771912	Human	Saliva
SRS016708	0.0422658679784	Human	Saliva
SRS016853	0.0241605486198	Human	Saliva
SRS016924	0.0306440455486	Human	Saliva
SRS017072	0.0293804819101	Human	Saliva
SRS017115	0.0409269800340	Human	Saliva
SRS017203	0.0711907278326	Human	Saliva
SRS017402	0.0528121583431	Human	Saliva
SRS017649	0.0227728721977	Human	Saliva
SRS017772	0.0614872185498	Human	Saliva
SRS017829	0.1340200008730	Human	Saliva
SRS017886	0.0175566491321	Human	Saliva
SRS017943	0.0300537776140	Human	Saliva
SRS018005	0.0182799517320	Human	Saliva

SRS018141	0.0450678479499	Human	Saliva
SRS018258	0.0278083394328	Human	Saliva
SRS018497	0.0347517442359	Human	Saliva
SRS018587	0.0250617027216	Human	Saliva
SRS018735	0.0642492916815	Human	Saliva
SRS018787	0.0200353638169	Human	Saliva
SRS019011	0.0398437526457	Human	Saliva
SRS019263	0.0295819556572	Human	Saliva
SRS019890	0.0256603493814	Human	Saliva
SRS020127	0.0180756627546	Human	Saliva
SRS020184	0.0439809938211	Human	Saliva
SRS020298	0.0336413149562	Human	Saliva
SRS020421	0.0414890444461	Human	Saliva
SRS020592	0.0386253747894	Human	Saliva
SRS020819	0.0287162665469	Human	Saliva
SRS020934	0.0472503185142	Human	Saliva
SRS021117	0.0282268179984	Human	Saliva
SRS021183	0.0270729940554	Human	Saliva
SRS021249	0.0200503082175	Human	Saliva
SRS021312	0.0152471221045	Human	Saliva
SRS021372	0.0323098086102	Human	Saliva
SRS021492	0.0692151643526	Human	Saliva
SRS021549	0.0637270072819	Human	Saliva
SRS021609	0.0392526926096	Human	Saliva
SRS021672	0.0353643786661	Human	Saliva
SRS021732	0.0398452526371	Human	Saliva
SRS021798	0.0343886208691	Human	Saliva
SRS021861	0.0262462737630	Human	Saliva
SRS021918	0.0237225417303	Human	Saliva
SRS021978	0.0233820512082	Human	Saliva
SRS022041	0.0306687655057	Human	Saliva
SRS022101	0.0513058533098	Human	Saliva
SRS022167	0.0550257999169	Human	Saliva
SRS022299	0.0447557251383	Human	Saliva
SRS022362	0.0437561234933	Human	Saliva
SRS022422	0.0296244425246	Human	Saliva
SRS022488	0.0291692041324	Human	Saliva
SRS022554	0.0312555554515	Human	Saliva
SRS022617	0.0486544893106	Human	Saliva
SRS022677	0.0327287675132	Human	Saliva
SRS022743	0.0322947672652	Human	Saliva
SRS022806	0.0410034438647	Human	Saliva
SRS022866	0.0581689499472	Human	Saliva
SRS022932	0.0236390708974	Human	Saliva
SRS022995	0.0403125998774	Human	Saliva
SRS023055	0.0337875523229	Human	Saliva
SRS023114	0.0253168472638	Human	Saliva
SRS023184	0.0236750670487	Human	Saliva

SRS023250	0.0382098965145	Human	Saliva
SRS023310	0.0420677026633	Human	Saliva
SRS023373	0.0711404592487	Human	Saliva
SRS023430	0.0235567793542	Human	Saliva
SRS023742	0.0383030726099	Human	Saliva
SRS023799	0.0276229834869	Human	Saliva
SRS023859	0.0308062785482	Human	Saliva
SRS024102	0.0820463885072	Human	Saliva
SRS024273	0.0326043087202	Human	Saliva
SRS024462	0.0587601391081	Human	Saliva
SRS024519	0.0249983765822	Human	Saliva
SRS024690	0.0224021710434	Human	Saliva
SRS042258	0.0300487791239	Human	Saliva
SRS042441	0.0259438098589	Human	Saliva
SRS042483	0.0399446648702	Human	Saliva
SRS042504	0.1040185846580	Human	Saliva
SRS042607	0.0464117844662	Human	Saliva
SRS043192	0.0559541544492	Human	Saliva
SRS044606	0.0208993582904	Human	Saliva
SRS044938	0.0446862177550	Human	Saliva
SRS045138	0.0255628974225	Human	Saliva
SRS045145	0.0205534715823	Human	Saliva
SRS045158	0.0330915127595	Human	Saliva
SRS045314	0.0152444750730	Human	Saliva
SRS045517	0.0553806853145	Human	Saliva
SRS046269	0.0279025559949	Human	Saliva
SRS046488	0.0461740593563	Human	Saliva
SRS046893	0.0380447971159	Human	Saliva
SRS047001	0.0282311304051	Human	Saliva
SRS047094	0.0527606262445	Human	Saliva
SRS047701	0.0771153997903	Human	Saliva
SRS048043	0.0525395138961	Human	Saliva
SRS048044	0.0438853152965	Human	Saliva
SRS049063	0.0735923611091	Human	Saliva
SRS049248	0.0365412776853	Human	Saliva
SRS050005	0.0547007184262	Human	Saliva
SRS050119	0.0295579305818	Human	Saliva
SRS050388	0.0322521225102	Human	Saliva
SRS050871	0.0561791070036	Human	Saliva
SRS050971	0.0426385731992	Human	Saliva
SRS052982	0.0360417363720	Human	Saliva
SRS053635	0.0325905246535	Human	Saliva
SRS054258	0.0326934716258	Human	Saliva
SRS054953	0.0315302396065	Human	Saliva
SRS055003	0.0175409039256	Human	Saliva
SRS055295	0.0318442354347	Human	Saliva
SRS055808	0.0856763779068	Human	Saliva
SRS055847	0.0299536219795	Human	Saliva

SRS055987	0.0619252904598	Human	Saliva
SRS056331	0.0236010611988	Human	Saliva
SRS056362	0.0781294391120	Human	Saliva
SRS056421	0.0258398196682	Human	Saliva
SRS056509	0.0487490014708	Human	Saliva
SRS056606	0.0616178273446	Human	Saliva
SRS057473	0.0730549455730	Human	Saliva
SRS057603	0.0244224375705	Human	Saliva
SRS058293	0.0320844787741	Human	Saliva
SRS058543	0.0224024593038	Human	Saliva
SRS062410	0.0585757938516	Human	Saliva
SRS062500	0.0500210849244	Human	Saliva
SRS062620	0.0168315835130	Human	Saliva
SRS062708	0.0365602758708	Human	Saliva
SRS062784	0.0398021667426	Human	Saliva
SRS063388	0.0352985179614	Human	Saliva
SRS063416	0.0375782315419	Human	Saliva
SRS063697	0.0185535983537	Human	Saliva
SRS063891	0.0297123445557	Human	Saliva
SRS063965	0.0392509855119	Human	Saliva
SRS064038	0.0249297562558	Human	Saliva
SRS065061	0.0348933531890	Human	Saliva
SRS065156	0.0322474270228	Human	Saliva
SRS065424	0.0345769809354	Human	Saliva
SRS065577	0.0169518953201	Human	Saliva
SRS065675	0.0157063450208	Human	Saliva
SRS011157	0.1182423055010	Human	Stool
SRS011271	0.0964212443317	Human	Stool
SRS011405	0.0950912867414	Human	Stool
SRS011413	0.0944949718729	Human	Stool
SRS011415	0.1688535845510	Human	Stool
SRS011452	0.0613780511400	Human	Stool
SRS011529	0.1253371410720	Human	Stool
SRS011586	0.1083842533020	Human	Stool
SRS011621	0.0457640776238	Human	Stool
SRS011653	0.1333362053760	Human	Stool
SRS012191	0.0765152935388	Human	Stool
SRS013177	0.1101904934030	Human	Stool
SRS013216	0.0424681099762	Human	Stool
SRS013386	0.0761845118374	Human	Stool
SRS013490	0.1221063397490	Human	Stool
SRS013543	0.0795056529540	Human	Stool
SRS013687	0.1158652555650	Human	Stool
SRS014572	0.0457234382824	Human	Stool
SRS014823	0.0635584817446	Human	Stool
SRS014885	0.0595161891555	Human	Stool
SRS014978	0.0985839029054	Human	Stool
SRS015133	0.0813405375758	Human	Stool

SRS015190	0.0668031900650	Human	Stool
SRS015247	0.0528594048708	Human	Stool
SRS015281	0.0486511846811	Human	Stool
SRS015452	0.0755646067319	Human	Stool
SRS015578	0.0597775216684	Human	Stool
SRS015724	0.0892427604506	Human	Stool
SRS015782	0.0793007027470	Human	Stool
SRS015815	0.0502182973852	Human	Stool
SRS016018	0.0580615407741	Human	Stool
SRS016152	0.0466511889004	Human	Stool
SRS016203	0.0737451357491	Human	Stool
SRS016267	0.0356382153604	Human	Stool
SRS016400	0.1515122544910	Human	Stool
SRS016437	0.0447151470539	Human	Stool
SRS016630	0.0954354480860	Human	Stool
SRS016841	0.0649212474949	Human	Stool
SRS016916	0.0601811080219	Human	Stool
SRS016954	0.0655520123954	Human	Stool
SRS017103	0.0694918696890	Human	Stool
SRS017191	0.0899872740484	Human	Stool
SRS017394	0.0830098431208	Human	Stool
SRS017641	0.0718153236875	Human	Stool
SRS017764	0.0314229361813	Human	Stool
SRS017821	0.1005118434820	Human	Stool
SRS017878	0.0406812416622	Human	Stool
SRS017935	0.0620512281456	Human	Stool
SRS017997	0.0967654068109	Human	Stool
SRS018068	0.0822051576881	Human	Stool
SRS018133	0.0718289289724	Human	Stool
SRS018250	0.0514217611001	Human	Stool
SRS018427	0.0789498991978	Human	Stool
SRS018489	0.1072848262530	Human	Stool
SRS018607	0.0538815946658	Human	Stool
SRS018733	0.0652319301457	Human	Stool
SRS018817	0.0732715829964	Human	Stool
SRS019013	0.0505852185944	Human	Stool
SRS019267	0.0540722412213	Human	Stool
SRS019910	0.0785201871408	Human	Stool
SRS020119	0.1085342823870	Human	Stool
SRS020176	0.0616263737647	Human	Stool
SRS020290	0.0495308872630	Human	Stool
SRS020413	0.0930094138098	Human	Stool
SRS020584	0.1105428120640	Human	Stool
SRS020811	0.0769529693411	Human	Stool
SRS020926	0.0472103966736	Human	Stool
SRS021109	0.0395931589629	Human	Stool
SRS021175	0.0691430989310	Human	Stool
SRS021241	0.0422779707495	Human	Stool

SRS021304	0.0524322838699	Human	Stool
SRS021364	0.1295741756420	Human	Stool
SRS021484	0.0659849642230	Human	Stool
SRS021541	0.0585312781448	Human	Stool
SRS021664	0.0469562435975	Human	Stool
SRS021724	0.0669187926694	Human	Stool
SRS021790	0.0978524186506	Human	Stool
SRS021853	0.1018682155120	Human	Stool
SRS021910	0.1738958739870	Human	Stool
SRS021970	0.1073496149010	Human	Stool
SRS022033	0.1088917284410	Human	Stool
SRS022093	0.1197747127800	Human	Stool
SRS022159	0.0584867717389	Human	Stool
SRS022291	0.1185999724360	Human	Stool
SRS022354	0.0688232924325	Human	Stool
SRS022414	0.0779068628555	Human	Stool
SRS022480	0.0823792478587	Human	Stool
SRS022546	0.0807443425712	Human	Stool
SRS022609	0.1379244577850	Human	Stool
SRS022669	0.0462539806519	Human	Stool
SRS022735	0.0455366295699	Human	Stool
SRS022798	0.0559829207397	Human	Stool
SRS022858	0.0611503397871	Human	Stool
SRS022924	0.0563896716138	Human	Stool
SRS022987	0.0485626964221	Human	Stool
SRS023047	0.1942350076470	Human	Stool
SRS023106	0.0604303454053	Human	Stool
SRS023176	0.0537222841525	Human	Stool
SRS023242	0.0392758661828	Human	Stool
SRS023302	0.0434669830238	Human	Stool
SRS023365	0.0415945479902	Human	Stool
SRS023422	0.0438480301049	Human	Stool
SRS023488	0.0606255292663	Human	Stool
SRS023734	0.0792985894823	Human	Stool
SRS023791	0.0475480734632	Human	Stool
SRS023851	0.0464114089240	Human	Stool
SRS024265	0.1455249511490	Human	Stool
SRS024454	0.1356653053400	Human	Stool
SRS024511	0.0547744206544	Human	Stool
SRS024682	0.1265893290570	Human	Stool
SRS042387	0.1006952969510	Human	Stool
SRS042415	0.0666754791047	Human	Stool
SRS042843	0.0509801424140	Human	Stool
SRS043769	0.0531579202099	Human	Stool
SRS044088	0.2219261913520	Human	Stool
SRS044146	0.1574457237310	Human	Stool
SRS045350	0.0710854897440	Human	Stool
SRS045414	0.0482129841839	Human	Stool

SRS045493	0.0527215236194	Human	Stool
SRS045526	0.0627605804060	Human	Stool
SRS045613	0.0407336321348	Human	Stool
SRS045627	0.0452558368704	Human	Stool
SRS045910	0.0574974675962	Human	Stool
SRS046216	0.0719201214263	Human	Stool
SRS046349	0.1243631090680	Human	Stool
SRS047561	0.1339042311090	Human	Stool
SRS047642	0.0696557176669	Human	Stool
SRS047967	0.0967079704711	Human	Stool
SRS048008	0.0420689249172	Human	Stool
SRS048299	0.0457561528419	Human	Stool
SRS048722	0.0527665892872	Human	Stool
SRS048853	0.1133006845940	Human	Stool
SRS049157	0.0435322092726	Human	Stool
SRS049949	0.0751230357471	Human	Stool
SRS049959	0.1641288861700	Human	Stool
SRS049982	0.0614300732507	Human	Stool
SRS050141	0.0835349641100	Human	Stool
SRS050374	0.1736013170070	Human	Stool
SRS051031	0.0815807915453	Human	Stool
SRS051086	0.0460778162284	Human	Stool
SRS051609	0.1268699044510	Human	Stool
SRS052326	0.0562410527061	Human	Stool
SRS052471	0.1218185809250	Human	Stool
SRS053301	0.0550153425269	Human	Stool
SRS054488	0.1143620907750	Human	Stool
SRS055137	0.0687388380797	Human	Stool
SRS055482	0.1392358752480	Human	Stool
SRS055697	0.0747014998108	Human	Stool
SRS055934	0.1486234478000	Human	Stool
SRS056255	0.0522309813475	Human	Stool
SRS056505	0.0529715567291	Human	Stool
SRS056656	0.0654920036172	Human	Stool
SRS057258	0.0713964662760	Human	Stool
SRS057447	0.0762556200555	Human	Stool
SRS057901	0.0585762907503	Human	Stool
SRS058416	0.0733202660241	Human	Stool
SRS062464	0.0915932876815	Human	Stool
SRS062610	0.0648825418066	Human	Stool
SRS062847	0.0660382386921	Human	Stool
SRS063068	0.0279910743080	Human	Stool
SRS063138	0.0383698189933	Human	Stool
SRS063214	0.1248611213200	Human	Stool
SRS063275	0.1020417578880	Human	Stool
SRS063307	0.1033381787390	Human	Stool
SRS063524	0.0682919253920	Human	Stool
SRS063797	0.0577473618007	Human	Stool

SRS063827	0.0610878177631	Human	Stool
SRS063921	0.0573089120854	Human	Stool
SRS063961	0.1093511528060	Human	Stool
SRS064276	0.0660275533676	Human	Stool
SRS065263	0.0562201955702	Human	Stool
SRS065466	0.0551000488964	Human	Stool
SRS065500	0.1278466884370	Human	Stool
PM002S3M1	0.0786378962025	Macaque	Vaginal
PM003S3M1	0.0434675748733	Macaque	Vaginal
PM004S3M1	0.0782936556719	Macaque	Vaginal
PM006S3M1	0.0737936552251	Macaque	Vaginal
PM008S3M1	0.0740683983235	Macaque	Vaginal
PM009S3M1	0.0891808968024	Macaque	Vaginal
PM010S3M1	0.0927927422663	Macaque	Vaginal
PM012S3M1	0.0752038121451	Macaque	Vaginal
PM014S3M1	0.0606154421160	Macaque	Vaginal
PM015S3M1	0.0728555713753	Macaque	Vaginal
PM016S3M1	0.1094499328490	Macaque	Vaginal
PM017S3M1	0.0827622036977	Macaque	Vaginal
PM022S3M1	0.0580725400351	Macaque	Vaginal
PM024S3M1	0.0905289391240	Macaque	Vaginal
PM025S3M1	0.0707679529544	Macaque	Vaginal
PM026S3M1	0.0794485361568	Macaque	Vaginal
PM027S3M1	0.0859177968168	Macaque	Vaginal
PM030S3M1	0.0884355451643	Macaque	Vaginal
PM031S3M1	0.0855077786336	Macaque	Vaginal
PM033S3M1	0.0642533762165	Macaque	Vaginal
PM039S3M1	0.0889305125549	Macaque	Vaginal
PM041S3M1	0.0632253977665	Macaque	Vaginal
PM042S3M1	0.1017672142280	Macaque	Vaginal
PM043S3M1	0.0987656235139	Macaque	Vaginal
PM044S3M1	0.0669192164378	Macaque	Vaginal
PM045S3M1	0.1036168355470	Macaque	Vaginal
PM047S3M1	0.0685706417268	Macaque	Vaginal
PM048S3M1	0.0711381013235	Macaque	Vaginal
PM049S3M1	0.0764617094609	Macaque	Vaginal
PM050S3M1	0.0097931484569	Macaque	Vaginal
PM051S3M1	0.0916058055677	Macaque	Vaginal
PM052S3M1	0.0678826811124	Macaque	Vaginal
PM053S3M1	0.0966266041287	Macaque	Vaginal
PM054S3M1	0.0606855880444	Macaque	Vaginal
PM056S3M1	0.0868010985230	Macaque	Vaginal
PM057S3M1	0.0810399705883	Macaque	Vaginal
PM058S3M1	0.0702924450355	Macaque	Vaginal
PM059S3M1	0.1413930274660	Macaque	Vaginal
PM060S3M1	0.0809164469859	Macaque	Vaginal
PM061S3M1	0.0878404197731	Macaque	Vaginal
PM062S3M1	0.0810838953973	Macaque	Vaginal

PM063S3M1	0.0682020449457	Macaque	Vaginal
PM064S3M1	0.1026991686530	Macaque	Vaginal
PM065S3M1	0.1134140945630	Macaque	Vaginal
PM066S3M1	0.1004749922860	Macaque	Vaginal
PM067S3M1	0.0731529772958	Macaque	Vaginal
PM069S3M1	0.1275405423740	Macaque	Vaginal
PM070S3M1	0.0800554103273	Macaque	Vaginal
PM071S3M1	0.1129998577030	Macaque	Vaginal
PM072S3M1	0.0394880017809	Macaque	Vaginal
PM073S3M1	0.1090503641880	Macaque	Vaginal
PM075S3M1	0.0964653411136	Macaque	Vaginal
PM076S3M1	0.0643579943528	Macaque	Vaginal
PM077S3M1	0.0885339297928	Macaque	Vaginal
PM078S3M1	0.1197653422140	Macaque	Vaginal
PM079S3M1	0.0777644753182	Macaque	Vaginal
PM080S3M1	0.1174887670040	Macaque	Vaginal
PM081S3M1	0.1280805803290	Macaque	Vaginal
PM082S3M1	0.0719398319663	Macaque	Vaginal
PM085S3M1	0.1047533694460	Macaque	Vaginal
PM086S3M1	0.0756005001137	Macaque	Vaginal
PM087S3M1	0.0985951037342	Macaque	Vaginal
PM089S3M1	0.0298954286108	Macaque	Vaginal
PM090S3M1	0.0778235777158	Macaque	Vaginal
PM092S3M1	0.0915958594178	Macaque	Vaginal
PM093S3M1	0.1076928618880	Macaque	Vaginal
PM094S3M1	0.1096116254540	Macaque	Vaginal
PM095S3M1	0.0756258027106	Macaque	Vaginal
PM096S3M1	0.0417487375368	Macaque	Vaginal
PM097S3M1	0.0898139092234	Macaque	Vaginal
PM098S3M1	0.0488175430751	Macaque	Vaginal
PM099S3M1	0.0986582667099	Macaque	Vaginal
PM103S3M1	0.1040448844670	Macaque	Vaginal
PM104S3M1	0.0930257731954	Macaque	Vaginal
PM105S3M1	0.1032649583470	Macaque	Vaginal
PM106S3M1	0.0997075272941	Macaque	Vaginal
PM107S3M1	0.0791217138198	Macaque	Vaginal
PM108S3M1	0.0836648795458	Macaque	Vaginal
PM109S3M1	0.0625876645098	Macaque	Vaginal
PM110S3M1	0.0881902946667	Macaque	Vaginal
PM112S3M1	0.0694060580559	Macaque	Vaginal
PM113S3M1	0.0692854653911	Macaque	Vaginal
PM115S3M1	0.0857737106647	Macaque	Vaginal
PM117S3M1	0.0790174714692	Macaque	Vaginal
SRS011446	0.0183042729752	Human	Vaginal
SRS011523	0.0154058225865	Human	Vaginal
SRS011580	0.0128603985738	Human	Vaginal
SRS011615	0.0224078356413	Human	Vaginal
SRS011682	0.0261690931088	Human	Vaginal

SRS013581	0.0159561766971	Human	Vaginal
SRS014546	0.0151878484952	Human	Vaginal
SRS014835	0.0174456273729	Human	Vaginal
SRS015145	0.0150833910264	Human	Vaginal
SRS015202	0.0215416508956	Human	Vaginal
SRS015259	0.0176619335068	Human	Vaginal
SRS015736	0.0267323242070	Human	Vaginal
SRS016215	0.0232339808071	Human	Vaginal
SRS016687	0.0149080594198	Human	Vaginal
SRS016728	0.0146863269600	Human	Vaginal
SRS018288	0.0154649755705	Human	Vaginal
SRS018527	0.0160487769340	Human	Vaginal
SRS018765	0.0216700389566	Human	Vaginal
SRS019241	0.0227681379984	Human	Vaginal
SRS021147	0.0158738493599	Human	Vaginal
SRS021213	0.0172946589194	Human	Vaginal
SRS021279	0.0153691594192	Human	Vaginal
SRS021402	0.0150328187725	Human	Vaginal
SRS021639	0.0463790230941	Human	Vaginal
SRS021762	0.0166922154616	Human	Vaginal
SRS021828	0.0452931872109	Human	Vaginal
SRS022008	0.0166215791520	Human	Vaginal
SRS022131	0.0173587835266	Human	Vaginal
SRS022197	0.0374240723861	Human	Vaginal
SRS022329	0.0219154678594	Human	Vaginal
SRS022452	0.0320338717895	Human	Vaginal
SRS022518	0.0173649790359	Human	Vaginal
SRS022584	0.0152617918143	Human	Vaginal
SRS022707	0.0218304548029	Human	Vaginal
SRS022773	0.0496742429309	Human	Vaginal
SRS022896	0.0481625490714	Human	Vaginal
SRS022962	0.0198893569694	Human	Vaginal
SRS023085	0.0179675586523	Human	Vaginal
SRS023145	0.0167901729765	Human	Vaginal
SRS023217	0.0247562595750	Human	Vaginal
SRS023340	0.0215171342822	Human	Vaginal
SRS023461	0.1128770489420	Human	Vaginal
SRS023889	0.0333897007838	Human	Vaginal
SRS024306	0.0163067824673	Human	Vaginal
SRS042961	0.0150446731538	Human	Vaginal
SRS043066	0.0208948590522	Human	Vaginal
SRS044715	0.0157820415690	Human	Vaginal
SRS044750	0.0383118825849	Human	Vaginal
SRS044945	0.0202311349008	Human	Vaginal
SRS044948	0.0220803840922	Human	Vaginal
SRS045920	0.0684555713503	Human	Vaginal
SRS048275	0.0146081811768	Human	Vaginal
SRS049964	0.0162188317475	Human	Vaginal

SRS050081	0.0150953254900	Human	Vaginal
SRS051407	0.0169363795392	Human	Vaginal
SRS051970	0.0147862500398	Human	Vaginal
SRS052468	0.0166912530509	Human	Vaginal
SRS053469	0.0153633466163	Human	Vaginal
SRS053877	0.0393434979405	Human	Vaginal
SRS054549	0.0447930919973	Human	Vaginal
SRS056514	0.0158002231852	Human	Vaginal
SRS056561	0.0151929159041	Human	Vaginal
SRS056693	0.0174289088694	Human	Vaginal
SRS056854	0.0163432780900	Human	Vaginal
SRS057898	0.0353679683234	Human	Vaginal
SRS062445	0.0119042116226	Human	Vaginal
SRS062896	0.0173023750958	Human	Vaginal
SRS063522	0.0213890381518	Human	Vaginal
SRS064618	0.0519601067702	Human	Vaginal
SRS064648	0.0177060309115	Human	Vaginal
SRS064833	0.0290766120430	Human	Vaginal
SRS064882	0.0337295185205	Human	Vaginal
SRS065165	0.0156537090428	Human	Vaginal
SRS065619	0.0180347109457	Human	Vaginal

Table S12. Relative abundance of anal bacterial taxa (L6, at the genus and above levels) between juvenile and adult male macaques.

Anal microbiota in male macaque animals	Adult		Juvenile			LDA score		
	Abundance$^{\$}$ (%)	Prevalence* (%)	Abundance$^{\$}$ (%)	Prevalence* (%)	MW test (p)	Adult	Juvenile	p value
k__Bacteria;p__Actinobacteria;c__Actinobacteria;o__Actinomycetales;f__Actinomycetaceae;g__Actinomyces	0.00 ± 0.00	0	0.00 ± 0.00	0	0.3164	-	-	-
k__Bacteria;p__Actinobacteria;c__Actinobacteria;o__Actinomycetales;f__Actinomycetaceae;g__Arcanobacterium	0.00 ± 0.00	0	0.00 ± 0.00	0	0.3806	-	-	-
k__Bacteria;p__Actinobacteria;c__Actinobacteria;o__Actinomycetales;f__Actinomycetaceae;g__Mobiluncus	0.00 ± 0.00	0	0.00 ± 0.00	0	0.3806	-	-	-
k__Bacteria;p__Actinobacteria;c__Actinobacteria;o__Actinomycetales;f__Actinomycetaceae;g__Varibaculum	0.00 ± 0.00	0	0.00 ± 0.00	0	0.4304	-	-	-
k__Bacteria;p__Actinobacteria;c__Actinobacteria;o__Bifidobacteriales;f__Bifidobacteriaceae;g__Alloscardovia	0.00 ± 0.00	0	0.00 ± 0.00	0	0.4553	-	-	-
k__Bacteria;p__Actinobacteria;c__Actinobacteria;o__Bifidobacteriales;f__Bifidobacteriaceae;g__Bifidobacterium	0.00 ± 0.00	0	0.00 ± 0.00	0	0.6013	-	-	-
k__Bacteria;p__Actinobacteria;c__Actinobacteria;o__Corynebacteriales;f__Corynebacteriaceae;g__Corynebacterium	0.02 ± 0.01	0	0.01 ± 0.01	0	0.3232	-	-	-
k__Bacteria;p__Actinobacteria;c__Actinobacteria;o__Corynebacteriales;f__Nocardiaceae;g__	0.01 ± 0.00	0	0.00 ± 0.00	0	0.3576	-	-	-
k__Bacteria;p__Actinobacteria;c__Actinobacteria;o__Micrococcales;f__Bogoriellaceae;g__Bogoriella	0.00 ± 0.00	0	0.00 ± 0.00	0	0.3164	-	-	-
k__Bacteria;p__Actinobacteria;c__Actinobacteria;o__Micrococcales;f__Dermabacteraceae;g__Brachybacterium	0.00 ± 0.00	0	0.00 ± 0.00	0	0.3164	-	-	-
k__Bacteria;p__Actinobacteria;c__Actinobacteria;o__Micrococcales;f__Microbacteriaceae;g__	0.00 ± 0.00	0	0.00 ± 0.00	0	0.3164	-	-	-
k__Bacteria;p__Actinobacteria;c__Actinobacteria;o__Micrococcales;f__Micrococcaceae;g__Arthrobacter	0.00 ± 0.00	0	0.00 ± 0.00	0	0.1376	-	-	-
k__Bacteria;p__Actinobacteria;c__Actinobacteria;o__Micrococcales;f__Micrococcaceae;g__Glutamicibacter	0.00 ± 0.00	0	0.00 ± 0.00	0	0.3164	-	-	-
k__Bacteria;p__Actinobacteria;c__Actinobacteria;o__Micrococcales;f__Micrococcaceae;g__Kocuria	0.00 ± 0.00	0	0.00 ± 0.00	0	0.1376	-	-	-
k__Bacteria;p__Actinobacteria;c__Actinobacteria;o__Micrococcales;f__Micrococcaceae;g__Micrococcus	0.00 ± 0.00	0	0.00 ± 0.00	0	0.1376	-	-	-
k__Bacteria;p__Actinobacteria;c__Actinobacteria;o__Micrococcales;f__Micrococcaceae;g__Rothia	0.00 ± 0.00	0	0.00 ± 0.00	0	0.6542	-	-	-
k__Bacteria;p__Actinobacteria;c__Coriobacteriia;o__Coriobacteriales;f__Atopobiaceae;g__Atopobium	0.00 ± 0.00	0	0.00 ± 0.00	0	0.192	-	-	-
k__Bacteria;p__Actinobacteria;c__Coriobacteriia;o__Coriobacteriales;f__Atopobiaceae;g__Olsenella	0.09 ± 0.02	0	0.07 ± 0.03	0	0.0726	-	-	-
k__Bacteria;p__Actinobacteria;c__Coriobacteriia;o__Coriobacteriales;f__Coriobacteriaceae;g__	0.11 ± 0.04	0	0.11 ± 0.06	0	0.6503	-	-	-
k__Bacteria;p__Actinobacteria;c__Coriobacteriia;o__Coriobacteriales;f__Coriobacteriaceae;g__Collinsella	0.04 ± 0.01	0	0.03 ± 0.01	0	0.3826	-	-	-
k__Bacteria;p__Actinobacteria;c__Coriobacteriia;o__Coriobacteriales;f__Coriobacteriaceae;g__Senegalimassilia	0.02 ± 0.01	0	0.03 ± 0.01	0	0.2233	-	-	-
k__Bacteria;p__Actinobacteria;c__Coriobacteriia;o__Eggerthellales;f__Eggerthellaceae;g__	0.02 ± 0.01	0	0.02 ± 0.01	0	0.5871	-	-	-
k__Bacteria;p__Actinobacteria;c__Coriobacteriia;o__Eggerthellales;f__Eggerthellaceae;g__Eggerthella	0.01 ± 0.00	0	0.00 ± 0.00	0	0.046	3.4358	-	0.0308
k__Bacteria;p__Actinobacteria;c__Coriobacteriia;o__Eggerthellales;f__Eggerthellaceae;g__Enterorhabdus	0.01 ± 0.00	0	0.00 ± 0.00	0	0.0581	3.4922	-	0.0379
k__Bacteria;p__Actinobacteria;c__Coriobacteriia;o__Eggerthellales;f__Eggerthellaceae;g__Raoultibacter	0.00 ± 0.00	0	0.00 ± 0.00	0	0.1376	-	-	-
k__Bacteria;p__Actinobacteria;c__Coriobacteriia;o__Eggerthellales;f__Eggerthellaceae;g__Slackia	0.01 ± 0.00	0	0.02 ± 0.01	0	0.3095	-	-	-
k__Bacteria;p__Bacteroidetes;c__Bacteroidia;o__Bacteroidales;f__;g__	0.15 ± 0.08	5.9	0.00 ± 0.00	0	0.1034	-	-	-
**k__Bacteria;p__Bacteroidetes;c__Bacteroidia;o__Bacteroidales;f__Bacteroidaceae;g__**	**1.00 ± 0.46**	**23.5**	**0.09 ± 0.02**	**0**	**0.0223**	**3.6954**	**-**	**0.0344**
k__Bacteria;p__Bacteroidetes;c__Bacteroidia;o__Bacteroidales;f__Bacteroidaceae;g__Bacteroides	0.82 ± 0.51	11.8	0.10 ± 0.06	0	0.0069	3.5866	-	0.0072
k__Bacteria;p__Bacteroidetes;c__Bacteroidia;o__Bacteroidales;f__Odoribacteraceae;g__Butyricimonas	0.03 ± 0.02	0	0.00 ± 0.00	0	0.2096	-	-	-
**k__Bacteria;p__Bacteroidetes;c__Bacteroidia;o__Bacteroidales;f__Porphyromonadaceae;g__**	**1.22 ± 0.37**	**29.4**	**0.82 ± 0.42**	**20**	**0.0141**	**3.5080**	**-**	**0.0159**
k__Bacteria;p__Bacteroidetes;c__Bacteroidia;o__Bacteroidales;f__Porphyromonadaceae;g__Muribaculum	0.37 ± 0.28	5.9	0.05 ± 0.03	0	0.1481	-	-	-
k__Bacteria;p__Bacteroidetes;c__Bacteroidia;o__Bacteroidales;f__Porphyromonadaceae;g__Paludibacter	0.50 ± 0.21	17.6	0.14 ± 0.08	6.7	0.1456	-	-	-
k__Bacteria;p__Bacteroidetes;c__Bacteroidia;o__Bacteroidales;f__Porphyromonadaceae;g__Parabacteroides	0.07 ± 0.02	0	0.12 ± 0.10	6.7	0.0483	-	-	-
k__Bacteria;p__Bacteroidetes;c__Bacteroidia;o__Bacteroidales;f__Porphyromonadaceae;g__Porphyromonas	0.14 ± 0.12	5.9	0.12 ± 0.06	0	0.4488	-	-	-
k__Bacteria;p__Bacteroidetes;c__Bacteroidia;o__Bacteroidales;f__Prevotellaceae;g__	0.76 ± 0.25	29.4	0.30 ± 0.11	6.7	0.0177	3.4732	-	0.0197
k__Bacteria;p__Bacteroidetes;c__Bacteroidia;o__Bacteroidales;f__Prevotellaceae;g__Alloprevotella	0.45 ± 0.15	17.6	0.39 ± 0.21	13.3	0.0177	3.1396	-	0.0219
**k__Bacteria;p__Bacteroidetes;c__Bacteroidia;o__Bacteroidales;f__Prevotellaceae;g__Prevotella**	**24.47 ± 3.01**	**100**	**10.02 ± 3.12**	**93.3**	**0.001**	**4.7564**	**-**	**0.0023**
**k__Bacteria;p__Bacteroidetes;c__Bacteroidia;o__Bacteroidales;f__Prevotellaceae;g__Prevotellamassilia**	**7.47 ± 1.31**	**94.1**	**2.67 ± 1.18**	**33.3**	**0.0011**	**4.3293**	**-**	**0.0020**
k__Bacteria;p__Bacteroidetes;c__Bacteroidia;o__Bacteroidales;f__Rikenellaceae;g__	0.10 ± 0.06	5.9	0.06 ± 0.03	0	0.833	-	-	-
k__Bacteria;p__Bacteroidetes;c__Bacteroidia;o__Bacteroidales;f__Rikenellaceae;g__Alistipes	0.01 ± 0.00	0	0.02 ± 0.01	0	0.2288	-	-	-
k__Bacteria;p__Bacteroidetes;c__Bacteroidia;o__Bacteroidales;f__Rikenellaceae;g__Rikenellaceae_RC9_gut_group	0.22 ± 0.08	5.9	0.09 ± 0.05	0	0.1667	-	-	-
k__Bacteria;p__Bacteroidetes;c__Bacteroidia;o__Marinilabiliales;f__Marinilabiliaceae;g__	0.32 ± 0.16	5.9	0.77 ± 0.76	6.7	0.0179	-	3.7022	0.0250
k__Bacteria;p__Bacteroidetes;c__Flavobacteriia;o__Flavobacteriales;f__Flavobacteriaceae;g__	0.05 ± 0.02	0	0.04 ± 0.02	0	0.0974	-	-	-
k__Bacteria;p__Bacteroidetes;c__Flavobacteriia;o__Flavobacteriales;f__Flavobacteriaceae;g__Chryseobacterium	0.00 ± 0.00	0	0.00 ± 0.00	0	0.9283	-	-	-
k__Bacteria;p__Bacteroidetes;c__Flavobacteriia;o__Flavobacteriales;f__Flavobacteriaceae;g__Cloacibacterium	0.00 ± 0.00	0	0.00 ± 0.00	0	0.3164	-	-	-
k__Bacteria;p__Bacteroidetes;c__Flavobacteriia;o__Flavobacteriales;f__Flavobacteriaceae;g__Flavobacterium	0.00 ± 0.00	0	0.00 ± 0.00	0	0.9283	-	-	-
k__Bacteria;p__Bacteroidetes;c__Flavobacteriia;o__Flavobacteriales;f__Flavobacteriaceae;g__Riemerella	0.00 ± 0.00	0	0.00 ± 0.00	0	0.9283	-	-	-
k__Bacteria;p__Bacteroidetes;c__Sphingobacteriia;o__Sphingobacteriales;f__Sphingobacteriaceae;g__	0.19 ± 0.04	0	0.16 ± 0.08	6.7	0.1297	-	-	-
k__Bacteria;p__Cyanobacteria;c__Melainabacteria;o__Gastranaerophilales;f__;g__	0.26 ± 0.11	5.9	0.09 ± 0.05	0	0.0145	3.0483	-	0.0203
k__Bacteria;p__Elusimicrobia;c__Elusimicrobia;o__Elusimicrobiales;f__Elusimicrobiaceae;g__Elusimicrobium	0.12 ± 0.10	5.9	0.44 ± 0.44	6.7	0.9827	-	-	-

k__Bacteria;p__Fibrobacteres;c__Fibrobacteria;o__Fibrobacterales;f__Fibrobacteraceae;g__Fibrobacter	0.01 ± 0.00	0	0.00 ± 0.00	0	0.029	3.0412	-	0.0456
k__Bacteria;p__Firmicutes;c__Bacilli;o__Bacillales;f__Alicyclobacillaceae;g__Alicyclobacillus	0.00 ± 0.00	0	0.01 ± 0.00	0	0.1801	-	-	-
k__Bacteria;p__Firmicutes;c__Bacilli;o__Bacillales;f__Bacillaceae;g__	0.00 ± 0.00	0	0.01 ± 0.00	0	0.1748	-	-	-
k__Bacteria;p__Firmicutes;c__Bacilli;o__Bacillales;f__Family_XI;g__Gemella	0.00 ± 0.00	0	0.01 ± 0.00	0	0.4473	-	-	-
k__Bacteria;p__Firmicutes;c__Bacilli;o__Bacillales;f__Paenibacillaceae;g__	0.02 ± 0.01	0	0.03 ± 0.03	0	0.6416	-	-	-
k__Bacteria;p__Firmicutes;c__Bacilli;o__Bacillales;f__Staphylococcaceae;g__Jeotgalicoccus	0.00 ± 0.00	0	0.00 ± 0.00	0	0.3164	-	-	-
k__Bacteria;p__Firmicutes;c__Bacilli;o__Bacillales;f__Staphylococcaceae;g__Macrococcus	0.00 ± 0.00	0	0.00 ± 0.00	0	0.1376	-	-	-
k__Bacteria;p__Firmicutes;c__Bacilli;o__Bacillales;f__Staphylococcaceae;g__Staphylococcus	0.00 ± 0.00	0	0.01 ± 0.00	0	0.0121	-	3.4969	0.0136
k__Bacteria;p__Firmicutes;c__Bacilli;o__Bacillales;f__Thermoactinomycetaceae;g__	0.00 ± 0.00	0	0.00 ± 0.00	0	0.655	-	-	-
k__Bacteria;p__Firmicutes;c__Bacilli;o__Lactobacillales;f__Aerococcaceae;g__Abiotrophia	0.00 ± 0.00	0	0.00 ± 0.00	0	0.3806	-	-	-
k__Bacteria;p__Firmicutes;c__Bacilli;o__Lactobacillales;f__Aerococcaceae;g__Aerococcus	0.00 ± 0.00	0	0.00 ± 0.00	0	0.9283	-	-	-
k__Bacteria;p__Firmicutes;c__Bacilli;o__Lactobacillales;f__Aerococcaceae;g__Facklamia	0.00 ± 0.00	0	0.00 ± 0.00	0	0.1376	-	-	-
k__Bacteria;p__Firmicutes;c__Bacilli;o__Lactobacillales;f__Carnobacteriaceae;g__Granulicatella	0.00 ± 0.00	0	0.00 ± 0.00	0	0.0523	-	-	-
k__Bacteria;p__Firmicutes;c__Bacilli;o__Lactobacillales;f__Lactobacillaceae;g__Lactobacillus	0.48 ± 0.09	11.8	0.28 ± 0.07	0	0.1227	-	-	-
k__Bacteria;p__Firmicutes;c__Bacilli;o__Lactobacillales;f__Lactobacillaceae;g__Pediococcus	0.00 ± 0.00	0	0.00 ± 0.00	0	0.1376	-	-	-
k__Bacteria;p__Firmicutes;c__Bacilli;o__Lactobacillales;f__Leuconostocaceae;g__Leuconostoc	0.02 ± 0.02	0	0.00 ± 0.00	0	0.3806	-	-	-
k__Bacteria;p__Firmicutes;c__Bacilli;o__Lactobacillales;f__Leuconostocaceae;g__Weissella	0.02 ± 0.02	0	0.00 ± 0.00	0	0.3986	-	-	-
k__Bacteria;p__Firmicutes;c__Bacilli;o__Lactobacillales;f__Streptococcaceae;g__Lactococcus	0.00 ± 0.00	0	0.00 ± 0.00	0	0.2931	-	-	-
k__Bacteria;p__Firmicutes;c__Bacilli;o__Lactobacillales;f__Streptococcaceae;g__Streptococcus	0.16 ± 0.04	0	0.09 ± 0.04	0	0.067	-	-	-
k__Bacteria;p__Firmicutes;c__Clostridia;o__Clostridiales;f__;g__	0.05 ± 0.02	0	0.09 ± 0.05	0	0.9691	-	-	-
k__Bacteria;p__Firmicutes;c__Clostridia;o__Clostridiales;f__Bacteroides;g__pectinophilus	0.02 ± 0.01	0	0.00 ± 0.00	0	0.0716	2.8914	-	0.0455
k__Bacteria;p__Firmicutes;c__Clostridia;o__Clostridiales;f__Caldicoprobacteraceae;g__	0.02 ± 0.01	0	0.01 ± 0.01	0	0.5186	-	-	-
k__Bacteria;p__Firmicutes;c__Clostridia;o__Clostridiales;f__Christensenellaceae;g__	0.12 ± 0.05	0	0.16 ± 0.10	6.7	0.4615	-	-	-
k__Bacteria;p__Firmicutes;c__Clostridia;o__Clostridiales;f__Christensenellaceae;g__Christensenella	0.01 ± 0.00	0	0.00 ± 0.00	0	0.0205	3.2687	-	0.0134
k__Bacteria;p__Firmicutes;c__Clostridia;o__Clostridiales;f__Christensenellaceae;g__Christensenellaceae_R-7_group	0.33 ± 0.08	11.8	0.28 ± 0.13	6.7	0.1227	-	-	-
k__Bacteria;p__Firmicutes;c__Clostridia;o__Clostridiales;f__Clostridiaceae;g__	0.03 ± 0.01	0	0.03 ± 0.02	0	0.1538	-	-	-
k__Bacteria;p__Firmicutes;c__Clostridia;o__Clostridiales;f__Clostridiaceae;g__Beduini	0.01 ± 0.01	0	0.01 ± 0.00	0	0.8621	-	-	-
k__Bacteria;p__Firmicutes;c__Clostridia;o__Clostridiales;f__Clostridiaceae;g__Butyricicoccus	0.27 ± 0.09	11.8	0.11 ± 0.05	0	0.0453	3.0510	-	0.0398
k__Bacteria;p__Firmicutes;c__Clostridia;o__Clostridiales;f__Clostridiaceae;g__Caloramator	0.00 ± 0.00	0	0.00 ± 0.00	0	0.3806	-	-	-
k__Bacteria;p__Firmicutes;c__Clostridia;o__Clostridiales;f__Clostridiaceae;g__Clostridium	1.10 ± 0.46	17.6	0.48 ± 0.39	6.7	0.0433	-	-	-
k__Bacteria;p__Firmicutes;c__Clostridia;o__Clostridiales;f__Clostridiaceae;g__Hungatella	0.26 ± 0.08	5.9	0.09 ± 0.03	0	0.0259	3.0349	-	0.0297
k__Bacteria;p__Firmicutes;c__Clostridia;o__Clostridiales;f__Clostridiaceae;g__Oxobacter	0.00 ± 0.00	0	0.00 ± 0.00	0	1	-	-	-
k__Bacteria;p__Firmicutes;c__Clostridia;o__Clostridiales;f__Clostridiales;g__	0.07 ± 0.04	0	0.02 ± 0.01	0	0.1827	-	-	-
k__Bacteria;p__Firmicutes;c__Clostridia;o__Clostridiales;f__Clostridiales;g__Anaerovorax	0.05 ± 0.01	0	0.04 ± 0.01	0	0.143	-	-	-
k__Bacteria;p__Firmicutes;c__Clostridia;o__Clostridiales;f__Clostridiales;g__Casaltella	0.00 ± 0.00	0	0.01 ± 0.00	0	0.062	-	-	-
k__Bacteria;p__Firmicutes;c__Clostridia;o__Clostridiales;f__Clostridiales;g__Emergencia	0.06 ± 0.03	0	0.04 ± 0.03	0	0.0503	-	-	-
k__Bacteria;p__Firmicutes;c__Clostridia;o__Clostridiales;f__Clostridiales;g__Ihubacter	0.01 ± 0.01	0	0.00 ± 0.00	0	0.075	-	-	-
k__Bacteria;p__Firmicutes;c__Clostridia;o__Clostridiales;f__Clostridiales;g__Mogibacterium	0.01 ± 0.00	0	0.00 ± 0.00	0	0.1883	-	-	-
k__Bacteria;p__Firmicutes;c__Clostridia;o__Clostridiales;f__Defluviitaleaceae;g__	0.00 ± 0.00	0	0.00 ± 0.00	0	0.585	-	-	-
k__Bacteria;p__Firmicutes;c__Clostridia;o__Clostridiales;f__Defluviitaleaceae;g__Vallitalea	0.01 ± 0.01	0	0.00 ± 0.00	0	0.4564	-	-	-
**k__Bacteria;p__Firmicutes;c__Clostridia;o__Clostridiales;f__Eubacteriaceae;g__Eubacterium**	**1.71 ± 0.30**	**64.7**	**1.03 ± 0.33**	**33.3**	**0.0244**	**3.5257**	**-**	**0.0243**
k__Bacteria;p__Firmicutes;c__Clostridia;o__Clostridiales;f__Fenollaria;g__massiliensis	0.01 ± 0.01	0	0.02 ± 0.01	0	0.8181	-	-	-
k__Bacteria;p__Firmicutes;c__Clostridia;o__Clostridiales;f__Flavonifractor;g__	0.01 ± 0.00	0	0.00 ± 0.00	0	0.0332	3.3644	-	0.0379
k__Bacteria;p__Firmicutes;c__Clostridia;o__Clostridiales;f__Flavonifractor;g__plautii	0.00 ± 0.00	0	0.00 ± 0.00	0	0.5503	-	-	-
k__Bacteria;p__Firmicutes;c__Clostridia;o__Clostridiales;f__Flintibacter;g__	0.19 ± 0.05	0	0.15 ± 0.08	6.7	0.0235	2.6349	-	0.0268
k__Bacteria;p__Firmicutes;c__Clostridia;o__Clostridiales;f__Gracilibacteraceae;g__	0.04 ± 0.01	0	0.03 ± 0.01	0	0.2616	-	-	-
k__Bacteria;p__Firmicutes;c__Clostridia;o__Clostridiales;f__Gracilibacteraceae;g__Gracilibacter	0.01 ± 0.00	0	0.01 ± 0.01	0	0.9644	-	-	-
k__Bacteria;p__Firmicutes;c__Clostridia;o__Clostridiales;f__Heliobacteriaceae;g__	0.01 ± 0.01	0	0.01 ± 0.01	0	0.0058	2.7672	-	0.0079
k__Bacteria;p__Firmicutes;c__Clostridia;o__Clostridiales;f__Howardella;g__ureilytica	0.00 ± 0.00	0	0.00 ± 0.00	0	0.192	-	-	-
k__Bacteria;p__Firmicutes;c__Clostridia;o__Clostridiales;f__Intestinimonas;g__	0.55 ± 0.14	11.8	0.21 ± 0.09	6.7	0.0031	3.2504	-	0.0039
k__Bacteria;p__Firmicutes;c__Clostridia;o__Clostridiales;f__Lachnospiraceae;g__	0.71 ± 0.13	17.6	0.30 ± 0.09	6.7	0.0013	3.2880	-	0.0023
k__Bacteria;p__Firmicutes;c__Clostridia;o__Clostridiales;f__Lachnospiraceae;g__Acetatifactor	0.01 ± 0.00	0	0.01 ± 0.01	0	0.2116	-	-	-
k__Bacteria;p__Firmicutes;c__Clostridia;o__Clostridiales;f__Lachnospiraceae;g__Anaerobium	0.03 ± 0.02	0	0.01 ± 0.00	0	0.5052	-	-	-
k__Bacteria;p__Firmicutes;c__Clostridia;o__Clostridiales;f__Lachnospiraceae;g__Anaerocolumna	0.00 ± 0.00	0	0.00 ± 0.00	0	0.9215	-	-	-
k__Bacteria;p__Firmicutes;c__Clostridia;o__Clostridiales;f__Lachnospiraceae;g__Anaerosporobacter	0.07 ± 0.01	0	0.04 ± 0.02	0	0.0368	-	-	-

k__Bacteria;p__Firmicutes;c__Clostridia;o__Clostridiales;f__Lachnospiraceae;g__Anaerostipes	0.50 ± 0.09	17.6	0.20 ± 0.09	6.7	0.0025	3.1984	-	0.0027
**k__Bacteria;p__Firmicutes;c__Clostridia;o__Clostridiales;f__Lachnospiraceae;g__Blautia**	**1.23 ± 0.22**	**47.1**	**0.74 ± 0.23**	**26.7**	**0.0299**	**3.3976**	**-**	**0.0243**
k__Bacteria;p__Firmicutes;c__Clostridia;o__Clostridiales;f__Lachnospiraceae;g__Butyrivibrio	0.27 ± 0.17	5.9	0.02 ± 0.01	0	0.0149	3.2358	-	0.0134
k__Bacteria;p__Firmicutes;c__Clostridia;o__Clostridiales;f__Lachnospiraceae;g__Cellulosilyticum	0.00 ± 0.00	0	0.00 ± 0.00	0	0.3164	-	-	-
**k__Bacteria;p__Firmicutes;c__Clostridia;o__Clostridiales;f__Lachnospiraceae;g__Clostridium**	**2.56 ± 0.49**	**88.2**	**0.91 ± 0.28**	**33.3**	**0.0024**	**3.9013**	**-**	**0.0039**
k__Bacteria;p__Firmicutes;c__Clostridia;o__Clostridiales;f__Lachnospiraceae;g__Coprococcus	0.33 ± 0.08	5.9	0.24 ± 0.11	6.7	0.0244	2.8657	-	0.0219
k__Bacteria;p__Firmicutes;c__Clostridia;o__Clostridiales;f__Lachnospiraceae;g__Cuneatibacter	0.05 ± 0.01	0	0.03 ± 0.02	0	0.003	2.9686	-	0.0032
k__Bacteria;p__Firmicutes;c__Clostridia;o__Clostridiales;f__Lachnospiraceae;g__Desulfotomaculum	0.77 ± 0.21	29.4	0.32 ± 0.11	6.7	0.0244	3.3406	-	0.0362
k__Bacteria;p__Firmicutes;c__Clostridia;o__Clostridiales;f__Lachnospiraceae;g__Dorea	0.49 ± 0.09	17.6	0.35 ± 0.14	13.3	0.0219	2.9085	-	0.0219
k__Bacteria;p__Firmicutes;c__Clostridia;o__Clostridiales;f__Lachnospiraceae;g__Eisenbergiella	0.05 ± 0.02	0	0.02 ± 0.01	0	0.0193	2.6447	-	0.0197
**k__Bacteria;p__Firmicutes;c__Clostridia;o__Clostridiales;f__Lachnospiraceae;g__Eubacterium**	**1.33 ± 0.40**	**41.2**	**0.38 ± 0.15**	**13.3**	**0.001**	**3.6485**	**-**	**0.0018**
k__Bacteria;p__Firmicutes;c__Clostridia;o__Clostridiales;f__Lachnospiraceae;g__Fusicatenibacter	0.15 ± 0.05	0	0.03 ± 0.01	0	0.0208	2.7882	-	0.0214
k__Bacteria;p__Firmicutes;c__Clostridia;o__Clostridiales;f__Lachnospiraceae;g__Hespellia	0.00 ± 0.00	0	0.00 ± 0.00	0	0.3806	-	-	-
k__Bacteria;p__Firmicutes;c__Clostridia;o__Clostridiales;f__Lachnospiraceae;g__Lachnospira	0.06 ± 0.02	0	0.02 ± 0.01	0	0.3352	-	-	-
k__Bacteria;p__Firmicutes;c__Clostridia;o__Clostridiales;f__Lachnospiraceae;g__Lachnospiraceae_ND3007_group	0.00 ± 0.00	0	0.00 ± 0.00	0	0.9215	-	-	-
k__Bacteria;p__Firmicutes;c__Clostridia;o__Clostridiales;f__Lachnospiraceae;g__Murimonas	0.06 ± 0.02	0	0.06 ± 0.05	0	0.0596	-	-	-
k__Bacteria;p__Firmicutes;c__Clostridia;o__Clostridiales;f__Lachnospiraceae;g__Oribacterium	0.00 ± 0.00	0	0.00 ± 0.00	0	0.192	-	-	-
k__Bacteria;p__Firmicutes;c__Clostridia;o__Clostridiales;f__Lachnospiraceae;g__Pseudobutyrivibrio	0.02 ± 0.01	0	0.01 ± 0.00	0	0.0422	3.0298	-	0.0488
**k__Bacteria;p__Firmicutes;c__Clostridia;o__Clostridiales;f__Lachnospiraceae;g__Roseburia**	**1.72 ± 0.57**	**35.3**	**0.32 ± 0.14**	**6.7**	**0.0032**	**3.7809**	**-**	**0.0050**
k__Bacteria;p__Firmicutes;c__Clostridia;o__Clostridiales;f__Lachnospiraceae;g__Tyzzerella	0.01 ± 0.01	0	0.00 ± 0.00	0	1	-	-	-
**k__Bacteria;p__Firmicutes;c__Clostridia;o__Clostridiales;f__Oscillospiraceae;g__Oscillibacter**	**1.17 ± 0.38**	**23.5**	**0.66 ± 0.36**	**20**	**0.0069**	**3.5434**	**-**	**0.0091**
k__Bacteria;p__Firmicutes;c__Clostridia;o__Clostridiales;f__Peptococcaceae;g__	0.08 ± 0.02	0	0.04 ± 0.01	0	0.0719	-	-	-
k__Bacteria;p__Firmicutes;c__Clostridia;o__Clostridiales;f__Peptococcaceae;g__Peptococcus	0.00 ± 0.00	0	0.02 ± 0.01	0	0.2041	-	-	-
k__Bacteria;p__Firmicutes;c__Clostridia;o__Clostridiales;f__Peptostreptococcaceae;g__	0.00 ± 0.00	0	0.00 ± 0.00	0	0.192	-	-	-
k__Bacteria;p__Firmicutes;c__Clostridia;o__Clostridiales;f__Peptostreptococcaceae;g__Intestinibacter	0.02 ± 0.01	0	0.05 ± 0.04	0	0.2069	-	-	-
k__Bacteria;p__Firmicutes;c__Clostridia;o__Clostridiales;f__Peptostreptococcaceae;g__Peptostreptococcus	0.00 ± 0.00	0	0.00 ± 0.00	0	0.062	-	-	-
k__Bacteria;p__Firmicutes;c__Clostridia;o__Clostridiales;f__Peptostreptococcaceae;g__Romboutsia	0.03 ± 0.02	0	0.00 ± 0.00	0	0.0526	-	-	-
k__Bacteria;p__Firmicutes;c__Clostridia;o__Clostridiales;f__Peptostreptococcaceae;g__Terrisporobacter	0.00 ± 0.00	0	0.00 ± 0.00	0	0.1494	-	-	-
k__Bacteria;p__Firmicutes;c__Clostridia;o__Clostridiales;f__Pseudoflavonifractor;g__	0.01 ± 0.01	0	0.01 ± 0.01	0	0.9283	-	-	-
k__Bacteria;p__Firmicutes;c__Clostridia;o__Clostridiales;f__Pseudoflavonifractor;g__capillosus	0.02 ± 0.01	0	0.02 ± 0.01	0	0.1353	-	-	-
k__Bacteria;p__Firmicutes;c__Clostridia;o__Clostridiales;f__Ruminococcaceae;g__	1.27 ± 0.23	52.9	1.15 ± 0.40	26.7	0.1136	-	-	-
k__Bacteria;p__Firmicutes;c__Clostridia;o__Clostridiales;f__Ruminococcaceae;g__Acetanaerobacterium	0.02 ± 0.01	0	0.00 ± 0.00	0	0.0977	-	-	-
k__Bacteria;p__Firmicutes;c__Clostridia;o__Clostridiales;f__Ruminococcaceae;g__Acetivibrio	0.08 ± 0.06	5.9	0.01 ± 0.01	0	0.0599	-	-	-
k__Bacteria;p__Firmicutes;c__Clostridia;o__Clostridiales;f__Ruminococcaceae;g__Acutalibacter	0.00 ± 0.00	0	0.00 ± 0.00	0	0.1887	-	-	-
k__Bacteria;p__Firmicutes;c__Clostridia;o__Clostridiales;f__Ruminococcaceae;g__Anaerobacterium	0.00 ± 0.00	0	0.01 ± 0.00	0	0.5359	-	-	-
k__Bacteria;p__Firmicutes;c__Clostridia;o__Clostridiales;f__Ruminococcaceae;g__Anaerofilum	0.08 ± 0.02	0	0.05 ± 0.03	0	0.0081	2.5161	-	0.0090
k__Bacteria;p__Firmicutes;c__Clostridia;o__Clostridiales;f__Ruminococcaceae;g__Anaeromassilibacillus	0.00 ± 0.00	0	0.00 ± 0.00	0	0.3164	-	-	-
k__Bacteria;p__Firmicutes;c__Clostridia;o__Clostridiales;f__Ruminococcaceae;g__Anaerotruncus	0.07 ± 0.02	0	0.04 ± 0.02	0	0.0203	2.5041	-	0.0187
k__Bacteria;p__Firmicutes;c__Clostridia;o__Clostridiales;f__Ruminococcaceae;g__Caproiciproducens	0.01 ± 0.01	0	0.04 ± 0.02	0	0.3532	-	-	-
k__Bacteria;p__Firmicutes;c__Clostridia;o__Clostridiales;f__Ruminococcaceae;g__Clostridium	0.36 ± 0.10	5.9	0.15 ± 0.04	0	0.1323	-	-	-
k__Bacteria;p__Firmicutes;c__Clostridia;o__Clostridiales;f__Ruminococcaceae;g__Ethanoligenens	0.12 ± 0.05	0	0.10 ± 0.07	6.7	0.0876	-	-	-
k__Bacteria;p__Firmicutes;c__Clostridia;o__Clostridiales;f__Ruminococcaceae;g__Eubacterium	0.15 ± 0.04	0	0.02 ± 0.01	0	0.0069	2.9400	-	0.0027
**k__Bacteria;p__Firmicutes;c__Clostridia;o__Clostridiales;f__Ruminococcaceae;g__Faecalibacterium**	**5.28 ± 0.90**	**88.2**	**2.69 ± 0.86**	**60**	**0.027**	**4.0883**	**-**	**0.0328**
k__Bacteria;p__Firmicutes;c__Clostridia;o__Clostridiales;f__Ruminococcaceae;g__Gemmiger	0.24 ± 0.05	0	0.12 ± 0.05	0	0.0212	2.7421	-	0.0296
k__Bacteria;p__Firmicutes;c__Clostridia;o__Clostridiales;f__Ruminococcaceae;g__Neglecta	0.02 ± 0.01	0	0.01 ± 0.00	0	0.3934	-	-	-
k__Bacteria;p__Firmicutes;c__Clostridia;o__Clostridiales;f__Ruminococcaceae;g__Papillibacter	0.02 ± 0.01	0	0.02 ± 0.01	0	0.0203	2.4401	-	0.0228
k__Bacteria;p__Firmicutes;c__Clostridia;o__Clostridiales;f__Ruminococcaceae;g__Phocea	0.00 ± 0.00	0	0.00 ± 0.00	0	0.3806	-	-	-
k__Bacteria;p__Firmicutes;c__Clostridia;o__Clostridiales;f__Ruminococcaceae;g__Ruminiclostridium	0.02 ± 0.01	0	0.03 ± 0.02	0	0.6696	-	-	-
**k__Bacteria;p__Firmicutes;c__Clostridia;o__Clostridiales;f__Ruminococcaceae;g__Ruminococcus**	**1.65 ± 0.47**	**47.1**	**0.39 ± 0.14**	**13.3**	**4.00E-04**	**3.8949**	**-**	**0.0005**
k__Bacteria;p__Firmicutes;c__Clostridia;o__Clostridiales;f__Ruminococcaceae;g__Saccharofermentans	0.00 ± 0.00	0	0.00 ± 0.00	0	0.8695	-	-	-
k__Bacteria;p__Firmicutes;c__Clostridia;o__Clostridiales;f__Ruminococcaceae;g__Sporobacter	0.81 ± 0.20	35.3	0.67 ± 0.31	20	0.216	-	-	-
k__Bacteria;p__Firmicutes;c__Clostridia;o__Clostridiales;f__Ruminococcaceae;g__Subdoligranulum	0.02 ± 0.01	0	0.02 ± 0.01	0	0.1492	-	-	-
k__Bacteria;p__Firmicutes;c__Erysipelotrichia;o__Erysipelotrichales;f__Erysipelotrichaceae;g__	0.42 ± 0.26	11.8	0.13 ± 0.06	0	0.105	-	-	-
k__Bacteria;p__Firmicutes;c__Erysipelotrichia;o__Erysipelotrichales;f__Erysipelotrichaceae;g__Bulleidia	0.07 ± 0.02	0	0.03 ± 0.01	0	0.0284	2.7606	-	0.0296
k__Bacteria;p__Firmicutes;c__Erysipelotrichia;o__Erysipelotrichales;f__Erysipelotrichaceae;g__Catenibacterium	0.76 ± 0.38	17.6	0.26 ± 0.10	6.7	0.2413	-	-	-

k__Bacteria;p__Firmicutes;c__Erysipelotrichia;o__Erysipelotrichales;f__Erysipelotrichaceae;g__Clostridium	0.19 ± 0.04	0	0.20 ± 0.08	6.7	0.2304	-	-	-
k__Bacteria;p__Firmicutes;c__Erysipelotrichia;o__Erysipelotrichales;f__Erysipelotrichaceae;g__Dielma	0.02 ± 0.02	0	0.00 ± 0.00	0	0.3806	-	-	-
k__Bacteria;p__Firmicutes;c__Erysipelotrichia;o__Erysipelotrichales;f__Erysipelotrichaceae;g__Erysipelatoclostridium	0.00 ± 0.00	0	0.00 ± 0.00	0	0.3806	-	-	-
k__Bacteria;p__Firmicutes;c__Erysipelotrichia;o__Erysipelotrichales;f__Erysipelotrichaceae;g__Erysipelotrichaceae_UCG-004	0.00 ± 0.00	0	0.00 ± 0.00	0	0.3806	-	-	-
k__Bacteria;p__Firmicutes;c__Erysipelotrichia;o__Erysipelotrichales;f__Erysipelotrichaceae;g__Holdemanella	0.06 ± 0.02	0	0.04 ± 0.02	0	0.2193	-	-	-
k__Bacteria;p__Firmicutes;c__Erysipelotrichia;o__Erysipelotrichales;f__Erysipelotrichaceae;g__Holdemania	0.00 ± 0.00	0	0.00 ± 0.00	0	0.3806	-	-	-
k__Bacteria;p__Firmicutes;c__Erysipelotrichia;o__Erysipelotrichales;f__Erysipelotrichaceae;g__Longibaculum	0.07 ± 0.02	0	0.02 ± 0.01	0	0.0392	-	-	-
k__Bacteria;p__Firmicutes;c__Erysipelotrichia;o__Erysipelotrichales;f__Erysipelotrichaceae;g__Solobacterium	0.00 ± 0.00	0	0.00 ± 0.00	0	0.3806	-	-	-
**k__Bacteria;p__Firmicutes;c__Negativicutes;o__Acidaminococcales;f__Acidaminococcaceae;g__Phascolarctobacterium**	**4.52 ± 0.90**	**100**	**1.18 ± 0.31**	**33.3**	**0**	**4.2230**	**-**	**0.0001**
k__Bacteria;p__Firmicutes;c__Negativicutes;o__Selenomonadales;f__Selenomonadaceae;g__	0.01 ± 0.00	0	0.01 ± 0.01	0	0.1766	-	-	-
k__Bacteria;p__Firmicutes;c__Negativicutes;o__Selenomonadales;f__Selenomonadaceae;g__Mitsuokella	0.01 ± 0.00	0	0.00 ± 0.00	0	0.2339	-	-	-
**k__Bacteria;p__Firmicutes;c__Negativicutes;o__Selenomonadales;f__Selenomonadaceae;g__Propionispira**	**3.98 ± 0.96**	**82.4**	**0.86 ± 0.20**	**46.7**	**0.0027**	**4.1403**	**-**	**0.0057**
k__Bacteria;p__Firmicutes;c__Negativicutes;o__Selenomonadales;f__Selenomonadaceae;g__Selenomonas	1.19 ± 0.40	29.4	0.43 ± 0.19	13.3	0.0857	-	-	-
k__Bacteria;p__Firmicutes;c__Negativicutes;o__Veillonellales;f__Veillonellaceae;g__Allisonella	0.02 ± 0.01	0	0.00 ± 0.00	0	0.6005	-	-	-
k__Bacteria;p__Firmicutes;c__Negativicutes;o__Veillonellales;f__Veillonellaceae;g__Dialister	1.70 ± 0.50	41.2	1.24 ± 0.47	33.3	0.331	-	-	-
k__Bacteria;p__Firmicutes;c__Negativicutes;o__Veillonellales;f__Veillonellaceae;g__Megasphaera	0.10 ± 0.02	0	0.28 ± 0.09	6.7	0.3733	-	-	-
k__Bacteria;p__Firmicutes;c__Negativicutes;o__Veillonellales;f__Veillonellaceae;g__Veillonella	0.02 ± 0.00	0	0.09 ± 0.07	6.7	0.9693	-	-	-
k__Bacteria;p__Firmicutes;c__Tissierellia;o__Tissierellales;f__Peptoniphilaceae;g__	0.01 ± 0.00	0	0.12 ± 0.09	6.7	0.3914	-	-	-
k__Bacteria;p__Firmicutes;c__Tissierellia;o__Tissierellales;f__Peptoniphilaceae;g__Anaerococcus	0.00 ± 0.00	0	0.04 ± 0.02	0	0.1508	-	-	-
k__Bacteria;p__Firmicutes;c__Tissierellia;o__Tissierellales;f__Peptoniphilaceae;g__Finegoldia	0.00 ± 0.00	0	0.03 ± 0.01	0	0.0499	-	-	-
k__Bacteria;p__Firmicutes;c__Tissierellia;o__Tissierellales;f__Peptoniphilaceae;g__Helcococcus	0.00 ± 0.00	0	0.01 ± 0.01	0	0.3164	-	-	-
k__Bacteria;p__Firmicutes;c__Tissierellia;o__Tissierellales;f__Peptoniphilaceae;g__Peptoniphilus	0.01 ± 0.00	0	0.07 ± 0.03	0	0.4218	-	-	-
k__Bacteria;p__Firmicutes;c__Tissierellia;o__Tissierellales;f__Tissierellaceae;g__	0.00 ± 0.00	0	0.01 ± 0.01	0	0.7089	-	-	-
k__Bacteria;p__Fusobacteria;c__Fusobacteriia;o__Fusobacteriales;f__Fusobacteriaceae;g__Fusobacterium	0.02 ± 0.01	0	0.21 ± 0.16	6.7	0.4075	-	-	-
k__Bacteria;p__Fusobacteria;c__Fusobacteriia;o__Fusobacteriales;f__Leptotrichiaceae;g__Leptotrichia	0.00 ± 0.00	0	0.00 ± 0.00	0	0.3164	-	-	-
k__Bacteria;p__Fusobacteria;c__Fusobacteriia;o__Fusobacteriales;f__Leptotrichiaceae;g__Sneathia	0.00 ± 0.00	0	0.00 ± 0.00	0	0.5017	-	-	-
k__Bacteria;p__Lentisphaerae;c__Lentisphaeria;o__Victivallales;f__;g__	0.01 ± 0.01	0	0.04 ± 0.02	0	0.4093	-	-	-
k__Bacteria;p__Lentisphaerae;c__Lentisphaeria;o__Victivallales;f__Victivallaceae;g__	0.01 ± 0.01	0	0.00 ± 0.00	0	0.0031	2.9663	-	0.0046
k__Bacteria;p__Lentisphaerae;c__Oligosphaeria;o__Oligosphaerales;f__Oligosphaeraceae;g__	0.02 ± 0.01	0	0.02 ± 0.02	0	0.1494	-	-	-
k__Bacteria;p__Proteobacteria;c__Alphaproteobacteria;o__Rhizobiales;f__Methylobacteriaceae;g__Methylobacterium	0.00 ± 0.00	0	0.00 ± 0.00	0	0.3806	-	-	-
k__Bacteria;p__Proteobacteria;c__Alphaproteobacteria;o__Rhodobacterales;f__Rhodobacteraceae;g__Paracoccus	0.00 ± 0.00	0	0.00 ± 0.00	0	0.3164	-	-	-
k__Bacteria;p__Proteobacteria;c__Alphaproteobacteria;o__Rhodobacterales;f__Rhodobacteraceae;g__Rhodobacter	0.00 ± 0.00	0	0.00 ± 0.00	0	0.3806	-	-	-
k__Bacteria;p__Proteobacteria;c__Alphaproteobacteria;o__Rhodospirillales;f__Acetobacteraceae;g__	0.03 ± 0.02	0	0.00 ± 0.00	0	0.0053	2.8187	-	0.0082
k__Bacteria;p__Proteobacteria;c__Alphaproteobacteria;o__Rhodospirillales;f__Rhodospirillaceae;g__	0.31 ± 0.15	11.8	0.16 ± 0.05	0	0.7189	-	-	-
k__Bacteria;p__Proteobacteria;c__Alphaproteobacteria;o__Rickettsiales;f__;g__	0.11 ± 0.10	5.9	0.06 ± 0.04	0	0.634	-	-	-
k__Bacteria;p__Proteobacteria;c__Alphaproteobacteria;o__Sphingomonadales;f__Sphingomonadaceae;g__	0.00 ± 0.00	0	0.00 ± 0.00	0	0.3806	-	-	-
k__Bacteria;p__Proteobacteria;c__Betaproteobacteria;o__Burkholderiales;f__Alcaligenaceae;g__Achromobacter	0.00 ± 0.00	0	0.00 ± 0.00	0	0.1376	-	-	-
k__Bacteria;p__Proteobacteria;c__Betaproteobacteria;o__Burkholderiales;f__Burkholderiaceae;g__Paraburkholderia	0.03 ± 0.02	0	0.01 ± 0.00	0	0.7024	-	-	-
k__Bacteria;p__Proteobacteria;c__Betaproteobacteria;o__Burkholderiales;f__Burkholderiaceae;g__Ralstonia	0.00 ± 0.00	0	0.00 ± 0.00	0	0.3164	-	-	-
k__Bacteria;p__Proteobacteria;c__Betaproteobacteria;o__Burkholderiales;f__Comamonadaceae;g__	0.16 ± 0.05	0	0.04 ± 0.01	0	0.0054	2.8654	-	0.0102
k__Bacteria;p__Proteobacteria;c__Betaproteobacteria;o__Burkholderiales;f__Comamonadaceae;g__Comamonas	0.00 ± 0.00	0	0.00 ± 0.00	0	0.3164	-	-	-
k__Bacteria;p__Proteobacteria;c__Betaproteobacteria;o__Burkholderiales;f__Oxalobacteraceae;g__Herbaspirillum	0.02 ± 0.00	0	0.01 ± 0.01	0	0.0088	2.9593	-	0.0109
k__Bacteria;p__Proteobacteria;c__Betaproteobacteria;o__Burkholderiales;f__Roseateles;g__	0.41 ± 0.25	5.9	0.13 ± 0.10	6.7	0.069	-	-	-
k__Bacteria;p__Proteobacteria;c__Betaproteobacteria;o__Burkholderiales;f__Rubrivivax;g__gelatinosus	0.00 ± 0.00	0	0.00 ± 0.00	0	0.3164	-	-	-
k__Bacteria;p__Proteobacteria;c__Betaproteobacteria;o__Burkholderiales;f__Sutterellaceae;g__Sutterella	0.36 ± 0.14	17.6	0.09 ± 0.05	0	0.211	-	-	-
k__Bacteria;p__Proteobacteria;c__Betaproteobacteria;o__Neisseriales;f__Neisseriaceae;g__	0.00 ± 0.00	0	0.00 ± 0.00	0	0.192	-	-	-
k__Bacteria;p__Proteobacteria;c__Betaproteobacteria;o__Neisseriales;f__Neisseriaceae;g__Neisseria	0.00 ± 0.00	0	0.00 ± 0.00	0	0.3806	-	-	-
k__Bacteria;p__Proteobacteria;c__Betaproteobacteria;o__Rhodocyclales;f__Rhodocyclaceae;g__Azoarcus	0.00 ± 0.00	0	0.00 ± 0.00	0	0.3806	-	-	-
k__Bacteria;p__Proteobacteria;c__Deltaproteobacteria;o__Bradymonadales;f__;g__	0.18 ± 0.09	5.9	0.03 ± 0.02	0	0.2689	-	-	-
k__Bacteria;p__Proteobacteria;c__Deltaproteobacteria;o__Desulfovibrionales;f__;g__	0.10 ± 0.09	5.9	0.02 ± 0.01	0	0.4757	-	-	-
k__Bacteria;p__Proteobacteria;c__Deltaproteobacteria;o__Desulfovibrionales;f__Desulfohalobiaceae;g__	0.00 ± 0.00	0	0.00 ± 0.00	0	0.3806	-	-	-
k__Bacteria;p__Proteobacteria;c__Deltaproteobacteria;o__Desulfovibrionales;f__Desulfovibrionaceae;g__	0.02 ± 0.01	0	0.04 ± 0.04	0	0.0077	-	2.6731	0.0083
k__Bacteria;p__Proteobacteria;c__Deltaproteobacteria;o__Desulfovibrionales;f__Desulfovibrionaceae;g__Desulfovibrio	0.47 ± 0.22	5.9	0.69 ± 0.36	13.3	0.9699	-	-	-
k__Bacteria;p__Proteobacteria;c__Deltaproteobacteria;o__Desulfuromonadales;f__Desulfuromonadaceae;g__	0.05 ± 0.05	0	0.03 ± 0.01	0	0.2971	-	-	-
k__Bacteria;p__Proteobacteria;c__Epsilonproteobacteria;o__Campylobacterales;f__Campylobacteraceae;g__Campylobacter	0.02 ± 0.02	0	0.02 ± 0.01	0	0.5599	-	-	-

**k__Bacteria;p__Proteobacteria;c__Epsilonproteobacteria;o__Campylobacterales;f__Helicobacteraceae;g__Helicobacter**	**12.49 ± 5.31**	**88.2**	**59.02 ± 9.03**	**93.3**	**7.00E-04**	**-**	**5.3083**	**0.0016**
k__Bacteria;p__Proteobacteria;c__Gammaproteobacteria;o__;f__;g__	0.32 ± 0.13	11.8	0.25 ± 0.16	6.7	0.0589	-	-	-
**k__Bacteria;p__Proteobacteria;c__Gammaproteobacteria;o__Aeromonadales;f__Succinivibrionaceae;g__Succinivibrio**	**3.25 ± 0.74**	**70.6**	**0.71 ± 0.27**	**20**	**0.0011**	**4.0840**	**-**	**0.0020**
k__Bacteria;p__Proteobacteria;c__Gammaproteobacteria;o__Alteromonadales;f__Idiomarinaceae;g__Aliidiomarina	0.00 ± 0.00	0	0.00 ± 0.00	0	0.4553	-	-	-
k__Bacteria;p__Proteobacteria;c__Gammaproteobacteria;o__Enterobacterales;f__Enterobacteriaceae;g__	0.05 ± 0.04	0	0.19 ± 0.15	6.7	0.6043	-	-	-
k__Bacteria;p__Proteobacteria;c__Gammaproteobacteria;o__Enterobacterales;f__Enterobacteriaceae;g__Enterobacter	0.00 ± 0.00	0	0.00 ± 0.00	0	0.0277	-	3.7545	0.0299
k__Bacteria;p__Proteobacteria;c__Gammaproteobacteria;o__Enterobacterales;f__Erwiniaceae;g__Pantoea	0.00 ± 0.00	0	0.00 ± 0.00	0	0.3164	-	-	-
k__Bacteria;p__Proteobacteria;c__Gammaproteobacteria;o__Enterobacterales;f__Morganellaceae;g__Providencia	0.00 ± 0.00	0	0.00 ± 0.00	0	0.3164	-	-	-
k__Bacteria;p__Proteobacteria;c__Gammaproteobacteria;o__Oceanospirillales;f__Halomonadaceae;g__Halomonas	0.13 ± 0.04	0	0.54 ± 0.17	13.3	0.0235	-	3.2800	0.0268
k__Bacteria;p__Proteobacteria;c__Gammaproteobacteria;o__Pasteurellales;f__Pasteurellaceae;g__	0.00 ± 0.00	0	0.00 ± 0.00	0	0.9283	-	-	-
k__Bacteria;p__Proteobacteria;c__Gammaproteobacteria;o__Pasteurellales;f__Pasteurellaceae;g__Aggregatibacter	0.00 ± 0.00	0	0.03 ± 0.02	0	0.6792	-	-	-
k__Bacteria;p__Proteobacteria;c__Gammaproteobacteria;o__Pasteurellales;f__Pasteurellaceae;g__Haemophilus	0.17 ± 0.08	5.9	0.87 ± 0.41	20	0.1043	-	-	-
k__Bacteria;p__Proteobacteria;c__Gammaproteobacteria;o__Pseudomonadales;f__Moraxellaceae;g__Acinetobacter	0.00 ± 0.00	0	0.00 ± 0.00	0	0.3603	-	-	-
k__Bacteria;p__Proteobacteria;c__Gammaproteobacteria;o__Pseudomonadales;f__Moraxellaceae;g__Moraxella	0.00 ± 0.00	0	0.00 ± 0.00	0	0.3164	-	-	-
k__Bacteria;p__Proteobacteria;c__Gammaproteobacteria;o__Pseudomonadales;f__Moraxellaceae;g__Psychrobacter	0.00 ± 0.00	0	0.00 ± 0.00	0	0.3806	-	-	-
k__Bacteria;p__Proteobacteria;c__Gammaproteobacteria;o__Pseudomonadales;f__Pseudomonadaceae;g__Pseudomonas	0.00 ± 0.00	0	0.00 ± 0.00	0	0.5017	-	-	-
k__Bacteria;p__Proteobacteria;c__Gammaproteobacteria;o__Xanthomonadales;f__Xanthomonadaceae;g__	0.00 ± 0.00	0	0.00 ± 0.00	0	0.3806	-	-	-
k__Bacteria;p__Proteobacteria;c__Gammaproteobacteria;o__Xanthomonadales;f__Xanthomonadaceae;g__Stenotrophomonas	0.00 ± 0.00	0	0.00 ± 0.00	0	0.3806	-	-	-
k__Bacteria;p__Spirochaetes;c__Spirochaetia;o__Brachyspirales;f__Brachyspiraceae;g__Brachyspira	0.13 ± 0.06	0	0.79 ± 0.17	40	0.0019	-	3.4831	0.0026
k__Bacteria;p__Spirochaetes;c__Spirochaetia;o__Spirochaetales;f__Spirochaetaceae;g__	0.08 ± 0.05	0	0.08 ± 0.04	0	0.661	-	-	-
k__Bacteria;p__Spirochaetes;c__Spirochaetia;o__Spirochaetales;f__Spirochaetaceae;g__Treponema	0.35 ± 0.11	11.8	0.06 ± 0.02	0	0.0282	3.2472	-	0.0434
k__Bacteria;p__Tenericutes;c__Mollicutes;o__Acholeplasmatales;f__Acholeplasmataceae;g__	0.04 ± 0.01	0	0.01 ± 0.00	0	0.6277	-	-	-
k__Bacteria;p__Tenericutes;c__Mollicutes;o__Anaeroplasmatales;f__Anaeroplasmataceae;g__	0.23 ± 0.11	5.9	0.06 ± 0.03	0	0.4253	-	-	-
k__Bacteria;p__Tenericutes;c__Mollicutes;o__Anaeroplasmatales;f__Anaeroplasmataceae;g__Anaeroplasma	0.01 ± 0.00	0	0.01 ± 0.01	0	0.8936	-	-	-
k__Bacteria;p__Tenericutes;c__Mollicutes;o__Entomoplasmatales;f__Entomoplasmataceae;g__	0.00 ± 0.00	0	0.00 ± 0.00	0	0.3806	-	-	-
k__Bacteria;p__Tenericutes;c__Mollicutes;o__Entomoplasmatales;f__Spiroplasmataceae;g__	0.04 ± 0.02	0	0.03 ± 0.02	0	0.261	-	-	-
k__Bacteria;p__Tenericutes;c__Mollicutes;o__Mollicutes_RF9;f__;g__	0.11 ± 0.04	0	0.12 ± 0.09	6.7	0.4117	-	-	-
k__Bacteria;p__Tenericutes;c__Mollicutes;o__Mycoplasmatales;f__Mycoplasmataceae;g__	0.01 ± 0.01	0	0.01 ± 0.00	0	0.6758	-	-	-
k__Bacteria;p__Tenericutes;c__Mollicutes;o__Mycoplasmatales;f__Mycoplasmataceae;g__Mycoplasma	0.00 ± 0.00	0	0.00 ± 0.00	0	1	-	-	-
k__Bacteria;p__Verrucomicrobia;c__;o__;f__;g__	0.33 ± 0.16	11.8	0.13 ± 0.10	6.7	0.027	3.1091	-	0.0378
k__Bacteria;p__Verrucomicrobia;c__Opitutae;o__Opitutae_vadinHA64;f__;g__	0.00 ± 0.00	0	0.00 ± 0.00	0	0.192	-	-	-
k__Bacteria;p__Verrucomicrobia;c__Opitutae;o__Puniceicoccales;f__Puniceicoccaceae;g__	0.01 ± 0.00	0	0.02 ± 0.01	0	0.479	-	-	-
k__Bacteria;p__Verrucomicrobia;c__Opitutae;o__Puniceicoccales;f__Puniceicoccaceae;g__Cerasicoccus	0.00 ± 0.00	0	0.01 ± 0.01	0	0.1376	-	-	-

$ mean ± s.e.m

* Individual samples with >1% abundance were counted.

Table S13. Relative abundance of vagnial bacterial taxa (L6, at the genus and above levels) between juvenile and adult macaques.

Vaginal microbiota in macaque animals	Adult		Juvenile					
	Abundance[$] (%)	Prevalence* (%)	Abundance[$] (%)	Prevalence* (%)	MW test (p)	Adult	Juvenile	p value
k__Bacteria;p__Acidobacteria;c__Acidobacteriia;o__Acidobacteriales;f__Acidobacteriaceae;g__	0.00 ± 0.00	0	0.00 ± 0.00	0	0.7391	-	-	-
k__Bacteria;p__Acidobacteria;c__Holophagae;o__;f__;g__	0.00 ± 0.00	0	0.00 ± 0.00	0	0.7391	-	-	-
k__Bacteria;p__Actinobacteria;c__Actinobacteria;o__Actinomycetales;f__Actinomycetaceae;g__	0.04 ± 0.03	1.4	0.08 ± 0.04	0	0.0324	-	2.3317	0.0342
k__Bacteria;p__Actinobacteria;c__Actinobacteria;o__Actinomycetales;f__Actinomycetaceae;g__Actinomyces	0.01 ± 0.01	0	0.01 ± 0.01	0	7.00E-04	-	-	0.0007
k__Bacteria;p__Actinobacteria;c__Actinobacteria;o__Actinomycetales;f__Actinomycetaceae;g__Arcanobacterium	0.02 ± 0.01	0	0.20 ± 0.10	0	0.0355	-	2.9039	0.0374
k__Bacteria;p__Actinobacteria;c__Actinobacteria;o__Actinomycetales;f__Actinomycetaceae;g__Flaviflexus	0.00 ± 0.00	0	0.00 ± 0.00	0	0.004	-	-	0.0040
**k__Bacteria;p__Actinobacteria;c__Actinobacteria;o__Actinomycetales;f__Actinomycetaceae;g__Mobiluncus**	**1.92 ± 0.24**	**65.8**	**0.02 ± 0.02**	**0**	**0**	**3.9849**	**-**	**0.0000**
k__Bacteria;p__Actinobacteria;c__Actinobacteria;o__Actinomycetales;f__Actinomycetaceae;g__Trueperella	0.02 ± 0.01	0	0.16 ± 0.13	10	0.0355	-	2.7644	0.0374
k__Bacteria;p__Actinobacteria;c__Actinobacteria;o__Bifidobacteriales;f__Bifidobacteriaceae;g__Alloscardovia	0.00 ± 0.00	0	0.00 ± 0.00	0	0.7391	-	-	-
k__Bacteria;p__Actinobacteria;c__Actinobacteria;o__Bifidobacteriales;f__Bifidobacteriaceae;g__Bifidobacterium	0.01 ± 0.01	0	0.01 ± 0.01	0	0.4987	-	-	-
k__Bacteria;p__Actinobacteria;c__Actinobacteria;o__Bifidobacteriales;f__Bifidobacteriaceae;g__Gardnerella	0.48 ± 0.22	11	0.00 ± 0.00	0	0.0238	3.3635	-	0.0257
**k__Bacteria;p__Actinobacteria;c__Actinobacteria;o__Corynebacteriales;f__Corynebacteriaceae;g__Corynebacterium**	**1.26 ± 0.38**	**17.8**	**5.78 ± 2.75**	**80**	**1.00E-04**	**-**	**4.2729**	**0.0001**
k__Bacteria;p__Actinobacteria;c__Actinobacteria;o__Corynebacteriales;f__Dietziaceae;g__Dietzia	0.01 ± 0.01	0	0.03 ± 0.03	0	0.4072	-	-	-
k__Bacteria;p__Actinobacteria;c__Actinobacteria;o__Frankiales;f__;g__	0.00 ± 0.00	0	0.00 ± 0.00	0	1.00E-04	-	-	0.0001
k__Bacteria;p__Actinobacteria;c__Actinobacteria;o__Frankiales;f__Antricoccus;g__	0.00 ± 0.00	0	0.00 ± 0.00	0	0.0077	-	2.6470	0.0073
k__Bacteria;p__Actinobacteria;c__Actinobacteria;o__Kineosporiales;f__Kineosporiaceae;g__Kineococcus	0.00 ± 0.00	0	0.00 ± 0.00	0	0.0077	-	2.1519	0.0073
k__Bacteria;p__Actinobacteria;c__Actinobacteria;o__Micrococcales;f__Bogoriellaceae;g__Bogoriella	0.00 ± 0.00	0	0.00 ± 0.00	0	0.1082	-	-	-
k__Bacteria;p__Actinobacteria;c__Actinobacteria;o__Micrococcales;f__Brevibacteriaceae;g__Brevibacterium	0.02 ± 0.02	1.4	0.01 ± 0.00	0	0.004	2.0126	-	0.0040
k__Bacteria;p__Actinobacteria;c__Actinobacteria;o__Micrococcales;f__Dermabacteraceae;g__Brachybacterium	0.00 ± 0.00	0	0.04 ± 0.03	0	0.004	-	2.3772	0.0040
k__Bacteria;p__Actinobacteria;c__Actinobacteria;o__Micrococcales;f__Dermatophilaceae;g__Dermatophilus	0.00 ± 0.00	0	0.00 ± 0.00	0	0.004	-	-	0.0040
k__Bacteria;p__Actinobacteria;c__Actinobacteria;o__Micrococcales;f__Dermatophilaceae;g__Kineosphaera	0.00 ± 0.00	0	0.00 ± 0.00	0	1.00E-04	-	-	0.0001
k__Bacteria;p__Actinobacteria;c__Actinobacteria;o__Micrococcales;f__Dermatophilaceae;g__Piscicoccus	0.00 ± 0.00	0	0.00 ± 0.00	0	0.1082	-	-	-
k__Bacteria;p__Actinobacteria;c__Actinobacteria;o__Micrococcales;f__Intrasporangiaceae;g__Knoellia	0.00 ± 0.00	0	0.01 ± 0.01	0	0.003	-	-	0.0031
k__Bacteria;p__Actinobacteria;c__Actinobacteria;o__Micrococcales;f__Intrasporangiaceae;g__Phycicoccus	0.00 ± 0.00	0	0.00 ± 0.00	0	0.0077	-	2.5595	0.0073
k__Bacteria;p__Actinobacteria;c__Actinobacteria;o__Micrococcales;f__Microbacteriaceae;g__	0.01 ± 0.01	0	0.03 ± 0.02	0	0.0029	-	2.2828	0.0030
k__Bacteria;p__Actinobacteria;c__Actinobacteria;o__Micrococcales;f__Microbacteriaceae;g__Amnibacterium	0.00 ± 0.00	0	0.01 ± 0.01	0	0.003	-	-	0.0031
k__Bacteria;p__Actinobacteria;c__Actinobacteria;o__Micrococcales;f__Microbacteriaceae;g__Leucobacter	0.01 ± 0.01	0	0.00 ± 0.00	0	0.004	-	-	0.0040
k__Bacteria;p__Actinobacteria;c__Actinobacteria;o__Micrococcales;f__Micrococcaceae;g__Arthrobacter	0.00 ± 0.00	0	0.03 ± 0.02	0	0.004	-	2.1426	0.0040
k__Bacteria;p__Actinobacteria;c__Actinobacteria;o__Micrococcales;f__Micrococcaceae;g__Kocuria	0.03 ± 0.03	1.4	0.05 ± 0.04	0	0.004	-	2.3857	0.0040
k__Bacteria;p__Actinobacteria;c__Actinobacteria;o__Micrococcales;f__Micrococcaceae;g__Micrococcus	0.00 ± 0.00	0	0.03 ± 0.03	0	0.0035	-	2.3297	0.0035
k__Bacteria;p__Actinobacteria;c__Actinobacteria;o__Micrococcales;f__Micrococcaceae;g__Rothia	0.00 ± 0.00	0	0.03 ± 0.02	0	7.00E-04	-	2.1667	0.0007
k__Bacteria;p__Actinobacteria;c__Actinobacteria;o__Pseudonocardiales;f__Pseudonocardiaceae;g__Saccharopolyspora	0.00 ± 0.00	0	0.00 ± 0.00	0	0.7391	-	-	-
k__Bacteria;p__Actinobacteria;c__Actinobacteria;o__Streptosporangiales;f__Thermomonosporaceae;g__Actinoallomurus	0.00 ± 0.00	0	0.00 ± 0.00	0	0.7391	-	-	-
k__Bacteria;p__Actinobacteria;c__Coriobacteriia;o__Coriobacteriales;f__Atopobiaceae;g__	0.10 ± 0.02	0	0.04 ± 0.01	0	0.2906	-	-	-
**k__Bacteria;p__Actinobacteria;c__Coriobacteriia;o__Coriobacteriales;f__Atopobiaceae;g__Atopobium**	**2.39 ± 0.49**	**49.3**	**0.21 ± 0.08**	**0**	**2.00E-04**	**4.0528**	**-**	**0.0002**
k__Bacteria;p__Actinobacteria;c__Coriobacteriia;o__Coriobacteriales;f__Atopobiaceae;g__Olsenella	0.02 ± 0.01	0	0.08 ± 0.02	0	0	-	2.5386	0.0000
k__Bacteria;p__Actinobacteria;c__Coriobacteriia;o__Coriobacteriales;f__Coriobacteriaceae;g__	0.01 ± 0.00	0	0.11 ± 0.05	0	6.00E-04	-	2.7104	0.0006
k__Bacteria;p__Actinobacteria;c__Coriobacteriia;o__Coriobacteriales;f__Coriobacteriaceae;g__Collinsella	0.01 ± 0.00	0	0.10 ± 0.05	0	1.00E-04	-	2.6914	0.0001
k__Bacteria;p__Actinobacteria;c__Coriobacteriia;o__Coriobacteriales;f__Coriobacteriaceae;g__Parvibacter	0.00 ± 0.00	0	0.00 ± 0.00	0	0.6169	-	-	-
k__Bacteria;p__Actinobacteria;c__Coriobacteriia;o__Coriobacteriales;f__Coriobacteriaceae;g__Senegalimassilia	0.00 ± 0.00	0	0.05 ± 0.02	0	0	-	2.4243	0.0000
k__Bacteria;p__Actinobacteria;c__Coriobacteriia;o__Eggerthellales;f__Eggerthellaceae;g__	0.01 ± 0.00	0	0.21 ± 0.17	10	0.0016	-	3.0229	0.0017
k__Bacteria;p__Actinobacteria;c__Coriobacteriia;o__Eggerthellales;f__Eggerthellaceae;g__Eggerthella	0.00 ± 0.00	0	0.01 ± 0.01	0	0.0082	-	-	0.0086
k__Bacteria;p__Actinobacteria;c__Coriobacteriia;o__Eggerthellales;f__Eggerthellaceae;g__Enterorhabdus	0.00 ± 0.00	0	0.01 ± 0.01	0	0.0063	-	-	0.0066
k__Bacteria;p__Actinobacteria;c__Coriobacteriia;o__Eggerthellales;f__Eggerthellaceae;g__Raoultibacter	0.00 ± 0.00	0	0.00 ± 0.00	0	0.7391	-	-	-
k__Bacteria;p__Actinobacteria;c__Coriobacteriia;o__Eggerthellales;f__Eggerthellaceae;g__Slackia	0.00 ± 0.00	0	0.02 ± 0.01	0	0.0035	-	-	0.0037
k__Bacteria;p__Bacteroidetes;c__Bacteroidia;o__Bacteroidales;f__;g__	0.01 ± 0.00	0	0.11 ± 0.11	10	0.2256	-	-	-
k__Bacteria;p__Bacteroidetes;c__Bacteroidia;o__Bacteroidales;f__Bacteroidaceae;g__	0.38 ± 0.10	2.7	0.14 ± 0.03	0	0.0638	-	-	-
k__Bacteria;p__Bacteroidetes;c__Bacteroidia;o__Bacteroidales;f__Bacteroidaceae;g__Bacteroides	0.01 ± 0.01	0	0.08 ± 0.04	0	0.0231	-	2.5866	0.0242
k__Bacteria;p__Bacteroidetes;c__Bacteroidia;o__Bacteroidales;f__Odoribacteraceae;g__Butyricimonas	0.00 ± 0.00	0	0.00 ± 0.00	0	1.00E-04	-	-	0.0001
k__Bacteria;p__Bacteroidetes;c__Bacteroidia;o__Bacteroidales;f__Porphyromonadaceae;g__	1.33 ± 0.30	37	0.54 ± 0.17	30	0.4712	-	-	-
k__Bacteria;p__Bacteroidetes;c__Bacteroidia;o__Bacteroidales;f__Porphyromonadaceae;g__Muribaculum	0.02 ± 0.01	0	0.08 ± 0.05	0	0.0141	-	2.6571	0.0149
k__Bacteria;p__Bacteroidetes;c__Bacteroidia;o__Bacteroidales;f__Porphyromonadaceae;g__Paludibacter	0.05 ± 0.02	1.4	0.47 ± 0.27	20	0.0123	-	3.4664	0.0131

k__Bacteria;p__Bacteroidetes;c__Bacteroidia;o__Bacteroidales;f__Porphyromonadaceae;g__Parabacteroides	0.00 ± 0.00	0	0.02 ± 0.01	0	0.0021	-	2.0524	0.0022
k__Bacteria;p__Bacteroidetes;c__Bacteroidia;o__Bacteroidales;f__Porphyromonadaceae;g__Petrimonas	0.00 ± 0.00	0	0.00 ± 0.00	0	0.0972	-	-	-
k__Bacteria;p__Bacteroidetes;c__Bacteroidia;o__Bacteroidales;f__Porphyromonadaceae;g__Porphyromonas	10.20 ± 1.00	94.5	6.11 ± 1.88	90	0.0892	-	-	-
k__Bacteria;p__Bacteroidetes;c__Bacteroidia;o__Bacteroidales;f__Porphyromonadaceae;g__Proteiniphilum	0.00 ± 0.00	0	0.00 ± 0.00	0	0.7391	-	-	-
**k__Bacteria;p__Bacteroidetes;c__Bacteroidia;o__Bacteroidales;f__Prevotellaceae;g__**	**3.15 ± 0.39**	**63**	**0.28 ± 0.12**	**10**	**0.0014**	**4.1413**	**-**	**0.0015**
k__Bacteria;p__Bacteroidetes;c__Bacteroidia;o__Bacteroidales;f__Prevotellaceae;g__Alloprevotella	0.01 ± 0.00	0	0.01 ± 0.00	0	0.2758	-	-	-
k__Bacteria;p__Bacteroidetes;c__Bacteroidia;o__Bacteroidales;f__Prevotellaceae;g__Prevotella	6.37 ± 0.63	94.5	6.63 ± 2.01	90	0.7423	-	-	-
k__Bacteria;p__Bacteroidetes;c__Bacteroidia;o__Bacteroidales;f__Prevotellaceae;g__Prevotellamassilia	0.23 ± 0.08	5.5	0.95 ± 0.38	30	2.00E-04	-	3.6609	0.0002
k__Bacteria;p__Bacteroidetes;c__Bacteroidia;o__Bacteroidales;f__Rikenellaceae;g__	0.01 ± 0.00	0	0.08 ± 0.05	0	0.0043	-	2.7181	0.0046
k__Bacteria;p__Bacteroidetes;c__Bacteroidia;o__Bacteroidales;f__Rikenellaceae;g__Alistipes	0.00 ± 0.00	0	0.00 ± 0.00	0	1.00E-04	-	-	0.0001
k__Bacteria;p__Bacteroidetes;c__Bacteroidia;o__Bacteroidales;f__Rikenellaceae;g__Rikenellaceae_RC9_gut_group	0.01 ± 0.00	0	0.12 ± 0.06	0	0.0755	-	-	-
k__Bacteria;p__Bacteroidetes;c__Bacteroidia;o__Marinilabiliales;f__Marinilabiliaceae;g__	0.01 ± 0.00	0	0.06 ± 0.04	0	8.00E-04	-	2.5399	0.0008
k__Bacteria;p__Bacteroidetes;c__Bacteroidia;o__Marinilabiliales;f__Prolixibacteraceae;g__Mariniphaga	0.00 ± 0.00	0	0.00 ± 0.00	0	0.7391	-	-	-
k__Bacteria;p__Bacteroidetes;c__Chitinophagia;o__Chitinophagales;f__Chitinophagaceae;g__Chitinophaga	0.00 ± 0.00	0	0.00 ± 0.00	0	0.7391	-	-	-
k__Bacteria;p__Bacteroidetes;c__Chitinophagia;o__Chitinophagales;f__Chitinophagaceae;g__Flavisolibacter	0.00 ± 0.00	0	0.01 ± 0.01	0	0.0077	-	2.3027	0.0073
k__Bacteria;p__Bacteroidetes;c__Cytophagia;o__Cytophagales;f__Cytophagaceae;g__Spirosoma	0.00 ± 0.00	0	0.00 ± 0.00	0	0.0077	-	2.2732	0.0073
k__Bacteria;p__Bacteroidetes;c__Cytophagia;o__Cytophagales;f__Hymenobacteraceae;g__Siccationidurans	0.00 ± 0.00	0	0.00 ± 0.00	0	0.0035	-	-	0.0035
k__Bacteria;p__Bacteroidetes;c__Flavobacteriia;o__Flavobacteriales;f__Crocinitomicaceae;g__Fluviicola	0.00 ± 0.00	0	0.01 ± 0.00	0	0.004	-	-	0.0040
k__Bacteria;p__Bacteroidetes;c__Flavobacteriia;o__Flavobacteriales;f__Flavobacteriaceae;g__	0.00 ± 0.00	0	0.10 ± 0.05	0	0.0055	-	2.8000	0.0057
k__Bacteria;p__Bacteroidetes;c__Flavobacteriia;o__Flavobacteriales;f__Flavobacteriaceae;g__Capnocytophaga	0.00 ± 0.00	0	0.01 ± 0.01	0	0.0077	-	2.1966	0.0073
k__Bacteria;p__Bacteroidetes;c__Flavobacteriia;o__Flavobacteriales;f__Flavobacteriaceae;g__Chryseobacterium	0.01 ± 0.01	0	0.39 ± 0.28	20	0.0783	-	3.4037	0.0404
k__Bacteria;p__Bacteroidetes;c__Flavobacteriia;o__Flavobacteriales;f__Flavobacteriaceae;g__Empedobacter	0.00 ± 0.00	0	0.00 ± 0.00	0	1.00E-04	-	-	0.0001
k__Bacteria;p__Bacteroidetes;c__Flavobacteriia;o__Flavobacteriales;f__Flavobacteriaceae;g__Flavobacterium	0.00 ± 0.00	0	0.08 ± 0.06	0	0	-	2.5658	0.0000
k__Bacteria;p__Bacteroidetes;c__Flavobacteriia;o__Flavobacteriales;f__Flavobacteriaceae;g__Riemerella	0.00 ± 0.00	0	0.04 ± 0.03	0	0.003	-	2.3388	0.0031
k__Bacteria;p__Bacteroidetes;c__Flavobacteriia;o__Flavobacteriales;f__Flavobacteriaceae;g__Soonwooa	0.01 ± 0.01	0	0.02 ± 0.02	0	0.004	-	2.1314	0.0040
k__Bacteria;p__Bacteroidetes;c__Sphingobacteriia;o__Sphingobacteriales;f__Chitinophagaceae;g__Taibaiella	0.00 ± 0.00	0	0.00 ± 0.00	0	0.004	-	-	0.0040
k__Bacteria;p__Bacteroidetes;c__Sphingobacteriia;o__Sphingobacteriales;f__Sphingobacteriaceae;g__	0.01 ± 0.00	0	0.13 ± 0.06	0	0.0012	-	2.8784	0.0013
k__Bacteria;p__Bacteroidetes;c__Sphingobacteriia;o__Sphingobacteriales;f__Sphingobacteriaceae;g__Pedobacter	0.00 ± 0.00	0	0.00 ± 0.00	0	0.0077	-	2.2608	0.0073
k__Bacteria;p__Bacteroidetes;c__Sphingobacteriia;o__Sphingobacteriales;f__Sphingobacteriaceae;g__Sphingobacterium	0.04 ± 0.04	1.4	0.15 ± 0.10	0	0.004	-	2.8635	0.0040
k__Bacteria;p__Chloroflexi;c__Thermomicrobia;o__Sphaerobacterales;f__Sphaerobacteraceae;g__	0.00 ± 0.00	0	0.01 ± 0.01	0	0.015	-	-	0.0151
k__Bacteria;p__Cyanobacteria;c__Cyanobacteria;o__SubsectionIII;f__FamilyI;g__	0.00 ± 0.00	0	0.00 ± 0.00	0	0.7391	-	-	-
k__Bacteria;p__Cyanobacteria;c__Melainabacteria;o__Gastranaerophilales;f__;g__	0.01 ± 0.00	0	0.16 ± 0.08	0	0.0181	-	2.9471	0.0190
k__Bacteria;p__Deinococcus-Thermus;c__Deinococci;o__Deinococcales;f__Deinococcaceae;g__Deinococcus	0.00 ± 0.00	0	0.00 ± 0.00	0	0.0077	-	2.0966	0.0073
k__Bacteria;p__Elusimicrobia;c__Elusimicrobia;o__Elusimicrobiales;f__Elusimicrobiaceae;g__Elusimicrobium	0.00 ± 0.00	0	0.00 ± 0.00	0	0.5415	-	-	-
k__Bacteria;p__Fibrobacteres;c__Fibrobacteria;o__Fibrobacterales;f__Fibrobacteraceae;g__Fibrobacter	0.00 ± 0.00	0	0.00 ± 0.00	0	0.0401	-	-	0.0404
k__Bacteria;p__Firmicutes;c__Bacilli;o__Bacillales;f__Alicyclobacillaceae;g__Alicyclobacillus	0.01 ± 0.00	0	0.04 ± 0.02	0	8.00E-04	-	2.2256	0.0009
k__Bacteria;p__Firmicutes;c__Bacilli;o__Bacillales;f__Bacillaceae;g__	0.00 ± 0.00	0	0.01 ± 0.01	0	0	-	-	0.0000
k__Bacteria;p__Firmicutes;c__Bacilli;o__Bacillales;f__Bacillaceae;g__Bacillus	0.00 ± 0.00	0	0.00 ± 0.00	0	0.7391	-	-	-
k__Bacteria;p__Firmicutes;c__Bacilli;o__Bacillales;f__Bacillaceae;g__Geobacillus	0.00 ± 0.00	0	0.00 ± 0.00	0	0.0077	-	2.0689	0.0073
k__Bacteria;p__Firmicutes;c__Bacilli;o__Bacillales;f__Family_XI;g__Gemella	0.00 ± 0.00	0	0.01 ± 0.00	0	0	-	-	0.0000
k__Bacteria;p__Firmicutes;c__Bacilli;o__Bacillales;f__Paenibacillaceae;g__	0.00 ± 0.00	0	0.06 ± 0.04	0	0.0068	-	2.5905	0.0071
k__Bacteria;p__Firmicutes;c__Bacilli;o__Bacillales;f__Paenibacillaceae;g__Paenibacillus	0.00 ± 0.00	0	0.00 ± 0.00	0	0.7391	-	-	-
k__Bacteria;p__Firmicutes;c__Bacilli;o__Bacillales;f__Planococcaceae;g__Kurthia	0.00 ± 0.00	0	0.01 ± 0.01	0	0.0077	-	2.1025	0.0073
k__Bacteria;p__Firmicutes;c__Bacilli;o__Bacillales;f__Staphylococcaceae;g__Jeotgalicoccus	0.02 ± 0.02	1.4	0.01 ± 0.01	0	0.004	-	-	0.0040
k__Bacteria;p__Firmicutes;c__Bacilli;o__Bacillales;f__Staphylococcaceae;g__Macrococcus	0.00 ± 0.00	0	0.03 ± 0.03	0	0.0035	-	2.3743	0.0035
k__Bacteria;p__Firmicutes;c__Bacilli;o__Bacillales;f__Staphylococcaceae;g__Staphylococcus	0.00 ± 0.00	0	0.04 ± 0.02	0	0	-	2.3329	0.0000
k__Bacteria;p__Firmicutes;c__Bacilli;o__Bacillales;f__Thermoactinomycetaceae;g__	0.00 ± 0.00	0	0.01 ± 0.00	0	0.0511	-	-	-
k__Bacteria;p__Firmicutes;c__Bacilli;o__Lactobacillales;f__Aerococcaceae;g__	0.05 ± 0.01	0	0.22 ± 0.09	0	0.0035	-	2.8970	0.0038
k__Bacteria;p__Firmicutes;c__Bacilli;o__Lactobacillales;f__Aerococcaceae;g__Abiotrophia	0.00 ± 0.00	0	0.01 ± 0.01	0	0.0077	-	2.4391	0.0073
**k__Bacteria;p__Firmicutes;c__Bacilli;o__Lactobacillales;f__Aerococcaceae;g__Aerococcus**	**0.75 ± 0.23**	**13.7**	**2.59 ± 1.77**	**30**	**0.0088**	**-**	**3.9139**	**0.0092**
k__Bacteria;p__Firmicutes;c__Bacilli;o__Lactobacillales;f__Aerococcaceae;g__Dolosicoccus	0.04 ± 0.01	0	0.22 ± 0.14	10	0.0013	-	2.9225	0.0014
**k__Bacteria;p__Firmicutes;c__Bacilli;o__Lactobacillales;f__Aerococcaceae;g__Eremococcus**	**0.11 ± 0.04**	**5.5**	**1.10 ± 0.90**	**10**	**6.00E-04**	**-**	**3.6596**	**0.0006**
**k__Bacteria;p__Firmicutes;c__Bacilli;o__Lactobacillales;f__Aerococcaceae;g__Facklamia**	**0.71 ± 0.16**	**17.8**	**2.06 ± 1.24**	**40**	**0.0094**	**-**	**3.7859**	**0.0102**
k__Bacteria;p__Firmicutes;c__Bacilli;o__Lactobacillales;f__Carnobacteriaceae;g__Alkalibacterium	0.00 ± 0.00	0	0.00 ± 0.00	0	0.7391	-	-	-
k__Bacteria;p__Firmicutes;c__Bacilli;o__Lactobacillales;f__Carnobacteriaceae;g__Atopostipes	0.12 ± 0.03	2.7	0.40 ± 0.29	10	0.0441	-	3.1287	0.0471
k__Bacteria;p__Firmicutes;c__Bacilli;o__Lactobacillales;f__Carnobacteriaceae;g__Granulicatella	0.00 ± 0.00	0	0.02 ± 0.01	0	0	-	-	0.0000

k__Bacteria;p__Firmicutes;c__Bacilli;o__Lactobacillales;f__Enterococcaceae;g__Enterococcus	0.00 ± 0.00	0	0.00 ± 0.00	0	0.0077	-	-	0.0073
k__Bacteria;p__Firmicutes;c__Bacilli;o__Lactobacillales;f__Lactobacillaceae;g__Lactobacillus	0.27 ± 0.07	9.6	0.94 ± 0.27	40	7.00E-04	-	3.5453	0.0008
k__Bacteria;p__Firmicutes;c__Bacilli;o__Lactobacillales;f__Leuconostocaceae;g__Weissella	0.00 ± 0.00	0	0.00 ± 0.00	0	0.5909	-	-	-
k__Bacteria;p__Firmicutes;c__Bacilli;o__Lactobacillales;f__Streptococcaceae;g__Lactococcus	0.00 ± 0.00	0	0.00 ± 0.00	0	0.0077	-	2.1494	0.0073
k__Bacteria;p__Firmicutes;c__Bacilli;o__Lactobacillales;f__Streptococcaceae;g__Streptococcus	1.15 ± 0.65	13.7	0.29 ± 0.13	10	0.0996	-	-	-
k__Bacteria;p__Firmicutes;c__Clostridia;o__Clostridiales;f__;g__	0.00 ± 0.00	0	0.02 ± 0.01	0	0.0611	-	-	-
k__Bacteria;p__Firmicutes;c__Clostridia;o__Clostridiales;f__Bacteroides;g__pectinophilus	0.00 ± 0.00	0	0.00 ± 0.00	0	0.7436	-	-	-
k__Bacteria;p__Firmicutes;c__Clostridia;o__Clostridiales;f__Caldicoprobacteraceae;g__	0.00 ± 0.00	0	0.01 ± 0.00	0	0.0225	-	-	0.0232
k__Bacteria;p__Firmicutes;c__Clostridia;o__Clostridiales;f__Christensenellaceae;g__	0.01 ± 0.00	0	0.10 ± 0.05	0	0.0047	-	2.7874	0.0051
k__Bacteria;p__Firmicutes;c__Clostridia;o__Clostridiales;f__Christensenellaceae;g__Christensenella	0.00 ± 0.00	0	0.00 ± 0.00	0	0.2607	-	-	-
k__Bacteria;p__Firmicutes;c__Clostridia;o__Clostridiales;f__Christensenellaceae;g__Christensenellaceae_R-7_group	0.04 ± 0.01	0	0.20 ± 0.07	0	0.0028	-	2.9936	0.0027
k__Bacteria;p__Firmicutes;c__Clostridia;o__Clostridiales;f__Clostridiaceae;g__	0.88 ± 0.25	17.8	0.25 ± 0.07	0	0.9331	-	-	-
k__Bacteria;p__Firmicutes;c__Clostridia;o__Clostridiales;f__Clostridiaceae;g__Beduini	0.01 ± 0.01	0	0.01 ± 0.01	0	0.0608	-	-	0.0493
k__Bacteria;p__Firmicutes;c__Clostridia;o__Clostridiales;f__Clostridiaceae;g__Butyricicoccus	0.04 ± 0.01	0	0.18 ± 0.05	0	1.00E-04	-	2.8446	0.0001
k__Bacteria;p__Firmicutes;c__Clostridia;o__Clostridiales;f__Clostridiaceae;g__Caloramator	0.00 ± 0.00	0	0.00 ± 0.00	0	0.0077	-	-	0.0073
k__Bacteria;p__Firmicutes;c__Clostridia;o__Clostridiales;f__Clostridiaceae;g__Clostridium	0.15 ± 0.05	4.1	0.75 ± 0.44	10	2.00E-04	-	3.3187	0.0002
k__Bacteria;p__Firmicutes;c__Clostridia;o__Clostridiales;f__Clostridiaceae;g__Hungatella	0.03 ± 0.01	0	0.09 ± 0.04	0	0.0091	-	2.6428	0.0097
k__Bacteria;p__Firmicutes;c__Clostridia;o__Clostridiales;f__Clostridiales;g__	0.00 ± 0.00	0	0.05 ± 0.03	0	0.0016	-	2.5324	0.0017
k__Bacteria;p__Firmicutes;c__Clostridia;o__Clostridiales;f__Clostridiales;g__Anaerovorax	0.01 ± 0.00	0	0.05 ± 0.02	0	0.0095	-	2.3866	0.0101
k__Bacteria;p__Firmicutes;c__Clostridia;o__Clostridiales;f__Clostridiales;g__Casaltella	0.00 ± 0.00	0	0.00 ± 0.00	0	0.7391	-	-	-
k__Bacteria;p__Firmicutes;c__Clostridia;o__Clostridiales;f__Clostridiales;g__Emergencia	0.00 ± 0.00	0	0.03 ± 0.02	0	1.00E-04	-	2.3296	0.0001
k__Bacteria;p__Firmicutes;c__Clostridia;o__Clostridiales;f__Clostridiales;g__Eubacterium	0.00 ± 0.00	0	0.00 ± 0.00	0	0.2805	-	-	-
k__Bacteria;p__Firmicutes;c__Clostridia;o__Clostridiales;f__Clostridiales;g__Ihubacter	0.00 ± 0.00	0	0.03 ± 0.02	0	2.00E-04	-	2.2589	0.0002
k__Bacteria;p__Firmicutes;c__Clostridia;o__Clostridiales;f__Clostridiales;g__Mogibacterium	0.00 ± 0.00	0	0.01 ± 0.01	0	0	-	-	0.0000
k__Bacteria;p__Firmicutes;c__Clostridia;o__Clostridiales;f__Defluviitaleaceae;g__	0.00 ± 0.00	0	0.00 ± 0.00	0	0.0185	-	-	0.0186
k__Bacteria;p__Firmicutes;c__Clostridia;o__Clostridiales;f__Defluviitaleaceae;g__Vallitalea	0.00 ± 0.00	0	0.01 ± 0.01	0	0.004	-	-	0.0040
k__Bacteria;p__Firmicutes;c__Clostridia;o__Clostridiales;f__Eubacteriaceae;g__	0.06 ± 0.02	0	0.00 ± 0.00	0	0.0026	2.5441	-	0.0023
**k__Bacteria;p__Firmicutes;c__Clostridia;o__Clostridiales;f__Eubacteriaceae;g__Eubacterium**	**0.30 ± 0.08**	**6.8**	**1.37 ± 0.34**	**50**	**0**	**-**	**3.8044**	**0.0000**
k__Bacteria;p__Firmicutes;c__Clostridia;o__Clostridiales;f__Family_XI;g__Murdochiella	0.01 ± 0.00	0	0.00 ± 0.00	0	0.358	-	-	-
k__Bacteria;p__Firmicutes;c__Clostridia;o__Clostridiales;f__Family_XIII;g__Family_XIII_UCG-001	0.07 ± 0.01	0	0.00 ± 0.00	0	0.001	2.5400	-	0.0010
k__Bacteria;p__Firmicutes;c__Clostridia;o__Clostridiales;f__Fenollaria;g__massiliensis	0.66 ± 0.28	15.1	0.05 ± 0.05	0	0.059	-	-	-
k__Bacteria;p__Firmicutes;c__Clostridia;o__Clostridiales;f__Flavonifractor;g__	0.00 ± 0.00	0	0.00 ± 0.00	0	0.1156	-	-	-
k__Bacteria;p__Firmicutes;c__Clostridia;o__Clostridiales;f__Flavonifractor;g__plautii	0.00 ± 0.00	0	0.00 ± 0.00	0	1.00E-04	-	-	0.0001
k__Bacteria;p__Firmicutes;c__Clostridia;o__Clostridiales;f__Flintibacter;g__	0.03 ± 0.01	0	0.14 ± 0.06	0	0.0018	-	2.8535	0.0020
k__Bacteria;p__Firmicutes;c__Clostridia;o__Clostridiales;f__Gracilibacteraceae;g__	0.00 ± 0.00	0	0.02 ± 0.01	0	0.0017	-	2.0824	0.0018
k__Bacteria;p__Firmicutes;c__Clostridia;o__Clostridiales;f__Gracilibacteraceae;g__Gracilibacter	0.00 ± 0.00	0	0.00 ± 0.00	0	0.5873	-	-	-
k__Bacteria;p__Firmicutes;c__Clostridia;o__Clostridiales;f__Heliobacteriaceae;g__	0.00 ± 0.00	0	0.02 ± 0.01	0	0	-	2.0377	0.0000
k__Bacteria;p__Firmicutes;c__Clostridia;o__Clostridiales;f__Howardella;g__	0.02 ± 0.00	0	0.00 ± 0.00	0	0.0092	-	-	0.0084
k__Bacteria;p__Firmicutes;c__Clostridia;o__Clostridiales;f__Howardella;g__ureilytica	0.00 ± 0.00	0	0.00 ± 0.00	0	1.00E-04	-	-	0.0001
k__Bacteria;p__Firmicutes;c__Clostridia;o__Clostridiales;f__Intestinimonas;g__	0.07 ± 0.02	0	0.30 ± 0.11	10	4.00E-04	-	3.1614	0.0004
**k__Bacteria;p__Firmicutes;c__Clostridia;o__Clostridiales;f__Lachnospiraceae;g__**	**2.92 ± 0.40**	**58.9**	**0.43 ± 0.15**	**30**	**0.0091**	**4.0664**	**-**	**0.0099**
k__Bacteria;p__Firmicutes;c__Clostridia;o__Clostridiales;f__Lachnospiraceae;g__Acetatifactor	0.00 ± 0.00	0	0.01 ± 0.01	0	0.0895	-	-	-
k__Bacteria;p__Firmicutes;c__Clostridia;o__Clostridiales;f__Lachnospiraceae;g__Anaerobium	0.00 ± 0.00	0	0.02 ± 0.01	0	0.0842	-	-	-
k__Bacteria;p__Firmicutes;c__Clostridia;o__Clostridiales;f__Lachnospiraceae;g__Anaerocolumna	0.00 ± 0.00	0	0.00 ± 0.00	0	0.6169	-	-	-
k__Bacteria;p__Firmicutes;c__Clostridia;o__Clostridiales;f__Lachnospiraceae;g__Anaerosporobacter	0.02 ± 0.01	0	0.23 ± 0.20	10	0.0474	-	3.0580	0.0492
k__Bacteria;p__Firmicutes;c__Clostridia;o__Clostridiales;f__Lachnospiraceae;g__Anaerostipes	0.09 ± 0.03	2.7	0.79 ± 0.24	30	1.00E-04	-	3.5971	0.0001
**k__Bacteria;p__Firmicutes;c__Clostridia;o__Clostridiales;f__Lachnospiraceae;g__Blautia**	**0.29 ± 0.08**	**12.3**	**1.23 ± 0.27**	**70**	**2.00E-04**	**-**	**3.6983**	**0.0002**
**k__Bacteria;p__Firmicutes;c__Clostridia;o__Clostridiales;f__Lachnospiraceae;g__Butyrivibrio**	**2.36 ± 0.48**	**47.9**	**0.03 ± 0.01**	**0**	**0.0052**	**4.0837**	**-**	**0.0040**
k__Bacteria;p__Firmicutes;c__Clostridia;o__Clostridiales;f__Lachnospiraceae;g__Catonella	0.53 ± 0.14	13.7	0.00 ± 0.00	0	3.00E-04	3.4125	-	0.0003
**k__Bacteria;p__Firmicutes;c__Clostridia;o__Clostridiales;f__Lachnospiraceae;g__Clostridium**	**0.24 ± 0.07**	**6.8**	**1.22 ± 0.34**	**50**	**0**	**-**	**3.7765**	**0.0000**
k__Bacteria;p__Firmicutes;c__Clostridia;o__Clostridiales;f__Lachnospiraceae;g__Coprococcus	0.05 ± 0.01	0	0.25 ± 0.07	0	2.00E-04	-	3.0591	0.0002
k__Bacteria;p__Firmicutes;c__Clostridia;o__Clostridiales;f__Lachnospiraceae;g__Cuneatibacter	0.01 ± 0.00	0	0.02 ± 0.01	0	0.0121	-	-	0.0128
k__Bacteria;p__Firmicutes;c__Clostridia;o__Clostridiales;f__Lachnospiraceae;g__Desulfotomaculum	0.07 ± 0.03	1.4	0.21 ± 0.05	0	0	-	2.8376	0.0000
k__Bacteria;p__Firmicutes;c__Clostridia;o__Clostridiales;f__Lachnospiraceae;g__Dorea	0.16 ± 0.05	5.5	0.49 ± 0.14	10	5.00E-04	-	3.2599	0.0005
k__Bacteria;p__Firmicutes;c__Clostridia;o__Clostridiales;f__Lachnospiraceae;g__Eisenbergiella	0.01 ± 0.00	0	0.03 ± 0.02	0	0.0336	-	2.0961	0.0352
k__Bacteria;p__Firmicutes;c__Clostridia;o__Clostridiales;f__Lachnospiraceae;g__Eubacterium	0.16 ± 0.05	5.5	0.61 ± 0.27	10	2.00E-04	-	3.4122	0.0002

k__Bacteria;p__Firmicutes;c__Clostridia;o__Clostridiales;f__Lachnospiraceae;g__Fusicatenibacter	0.02 ± 0.01	0	0.05 ± 0.02	0	0.0015	-	2.1602	0.0017
k__Bacteria;p__Firmicutes;c__Clostridia;o__Clostridiales;f__Lachnospiraceae;g__Hespellia	0.00 ± 0.00	0	0.00 ± 0.00	0	0.3964	-	-	-
k__Bacteria;p__Firmicutes;c__Clostridia;o__Clostridiales;f__Lachnospiraceae;g__Lachnospira	0.01 ± 0.00	0	0.02 ± 0.01	0	0.0279	-	-	0.0290
k__Bacteria;p__Firmicutes;c__Clostridia;o__Clostridiales;f__Lachnospiraceae;g__Murimonas	0.01 ± 0.00	0	0.03 ± 0.01	0	0.0024	-	2.0187	0.0027
k__Bacteria;p__Firmicutes;c__Clostridia;o__Clostridiales;f__Lachnospiraceae;g__Oribacterium	0.00 ± 0.00	0	0.00 ± 0.00	0	0.0077	-	2.4075	0.0073
k__Bacteria;p__Firmicutes;c__Clostridia;o__Clostridiales;f__Lachnospiraceae;g__Pseudobutyrivibrio	0.01 ± 0.00	0	0.01 ± 0.01	0	0.2425	-	-	-
k__Bacteria;p__Firmicutes;c__Clostridia;o__Clostridiales;f__Lachnospiraceae;g__Roseburia	0.15 ± 0.06	5.5	0.38 ± 0.13	20	2.00E-04	-	3.1157	0.0002
k__Bacteria;p__Firmicutes;c__Clostridia;o__Clostridiales;f__Levyella;g__	0.00 ± 0.00	0	0.00 ± 0.00	0	0.1735	-	-	-
k__Bacteria;p__Firmicutes;c__Clostridia;o__Clostridiales;f__Oscillospiraceae;g__	0.00 ± 0.00	0	0.00 ± 0.00	0	0.0077	-	-	0.0073
k__Bacteria;p__Firmicutes;c__Clostridia;o__Clostridiales;f__Oscillospiraceae;g__Oscillibacter	0.12 ± 0.05	2.7	0.89 ± 0.41	30	1.00E-04	-	3.7014	0.0001
k__Bacteria;p__Firmicutes;c__Clostridia;o__Clostridiales;f__Peptococcaceae;g__	0.02 ± 0.01	0	0.11 ± 0.04	0	0.0014	-	2.7347	0.0015
k__Bacteria;p__Firmicutes;c__Clostridia;o__Clostridiales;f__Peptococcaceae;g__Peptococcus	0.13 ± 0.03	0	0.03 ± 0.01	0	0.0665	-	-	-
**k__Bacteria;p__Firmicutes;c__Clostridia;o__Clostridiales;f__Peptostreptococcaceae;g__**	**1.76 ± 0.27**	**47.9**	**0.26 ± 0.13**	**10**	**0.001**	**3.8404**	**-**	**0.0011**
k__Bacteria;p__Firmicutes;c__Clostridia;o__Clostridiales;f__Peptostreptococcaceae;g__Clostridioides	0.00 ± 0.00	0	0.00 ± 0.00	0	0.0077	-	-	0.0073
k__Bacteria;p__Firmicutes;c__Clostridia;o__Clostridiales;f__Peptostreptococcaceae;g__Eubacterium	0.00 ± 0.00	0	0.00 ± 0.00	0	0.0077	-	-	0.0073
k__Bacteria;p__Firmicutes;c__Clostridia;o__Clostridiales;f__Peptostreptococcaceae;g__Intestinibacter	0.01 ± 0.00	0	0.16 ± 0.07	0	0	-	2.8532	0.0000
k__Bacteria;p__Firmicutes;c__Clostridia;o__Clostridiales;f__Peptostreptococcaceae;g__Peptostreptococcus	0.19 ± 0.07	5.5	0.00 ± 0.00	0	0.0692	-	-	-
k__Bacteria;p__Firmicutes;c__Clostridia;o__Clostridiales;f__Peptostreptococcaceae;g__Romboutsia	0.01 ± 0.00	0	0.00 ± 0.00	0	0.358	-	-	-
k__Bacteria;p__Firmicutes;c__Clostridia;o__Clostridiales;f__Peptostreptococcaceae;g__Terrisporobacter	0.00 ± 0.00	0	0.02 ± 0.01	0	0.0018	-	-	0.0019
k__Bacteria;p__Firmicutes;c__Clostridia;o__Clostridiales;f__Pseudoflavonifractor;g__	0.00 ± 0.00	0	0.00 ± 0.00	0	0.0035	-	-	0.0035
k__Bacteria;p__Firmicutes;c__Clostridia;o__Clostridiales;f__Pseudoflavonifractor;g__capillosus	0.00 ± 0.00	0	0.01 ± 0.01	0	0.001	-	-	0.0011
**k__Bacteria;p__Firmicutes;c__Clostridia;o__Clostridiales;f__Ruminococcaceae;g__**	**1.72 ± 0.22**	**54.8**	**5.29 ± 1.52**	**90**	**0.0055**	**-**	**4.2342**	**0.0057**
k__Bacteria;p__Firmicutes;c__Clostridia;o__Clostridiales;f__Ruminococcaceae;g__Acetanaerobacterium	0.00 ± 0.00	0	0.02 ± 0.01	0	0.0295	-	-	0.0302
k__Bacteria;p__Firmicutes;c__Clostridia;o__Clostridiales;f__Ruminococcaceae;g__Acetivibrio	0.01 ± 0.00	0	0.03 ± 0.02	0	4.00E-04	-	2.1611	0.0004
k__Bacteria;p__Firmicutes;c__Clostridia;o__Clostridiales;f__Ruminococcaceae;g__Acutalibacter	0.00 ± 0.00	0	0.00 ± 0.00	0	0.0077	-	-	0.0073
k__Bacteria;p__Firmicutes;c__Clostridia;o__Clostridiales;f__Ruminococcaceae;g__Anaerobacterium	0.00 ± 0.00	0	0.02 ± 0.01	0	1.00E-04	-	2.0605	0.0001
k__Bacteria;p__Firmicutes;c__Clostridia;o__Clostridiales;f__Ruminococcaceae;g__Anaerofilum	0.01 ± 0.00	0	0.04 ± 0.02	0	0.0333	-	2.3255	0.0306
k__Bacteria;p__Firmicutes;c__Clostridia;o__Clostridiales;f__Ruminococcaceae;g__Anaerotruncus	0.01 ± 0.00	0	0.05 ± 0.03	0	9.00E-04	-	2.4695	0.0010
k__Bacteria;p__Firmicutes;c__Clostridia;o__Clostridiales;f__Ruminococcaceae;g__Caproiciproducens	0.00 ± 0.00	0	0.00 ± 0.00	0	0.1944	-	-	-
k__Bacteria;p__Firmicutes;c__Clostridia;o__Clostridiales;f__Ruminococcaceae;g__Clostridium	0.07 ± 0.02	1.4	0.29 ± 0.08	0	0	-	3.1270	0.0000
k__Bacteria;p__Firmicutes;c__Clostridia;o__Clostridiales;f__Ruminococcaceae;g__Ethanoligenens	0.01 ± 0.00	0	0.12 ± 0.07	0	0.0037	-	2.8412	0.0040
k__Bacteria;p__Firmicutes;c__Clostridia;o__Clostridiales;f__Ruminococcaceae;g__Eubacterium	0.01 ± 0.00	0	0.19 ± 0.11	10	8.00E-04	-	3.0222	0.0008
**k__Bacteria;p__Firmicutes;c__Clostridia;o__Clostridiales;f__Ruminococcaceae;g__Faecalibacterium**	**0.76 ± 0.27**	**13.7**	**2.11 ± 0.56**	**70**	**1.00E-04**	**-**	**3.9009**	**0.0001**
k__Bacteria;p__Firmicutes;c__Clostridia;o__Clostridiales;f__Ruminococcaceae;g__Fastidiosipila	0.14 ± 0.03	1.4	0.13 ± 0.05	0	0.8771	-	-	-
k__Bacteria;p__Firmicutes;c__Clostridia;o__Clostridiales;f__Ruminococcaceae;g__Gemmiger	0.05 ± 0.02	1.4	0.39 ± 0.17	10	5.00E-04	-	3.3325	0.0005
k__Bacteria;p__Firmicutes;c__Clostridia;o__Clostridiales;f__Ruminococcaceae;g__Neglecta	0.00 ± 0.00	0	0.01 ± 0.01	0	0.0115	-	-	0.0120
k__Bacteria;p__Firmicutes;c__Clostridia;o__Clostridiales;f__Ruminococcaceae;g__Papillibacter	0.00 ± 0.00	0	0.01 ± 0.01	0	0.5294	-	-	-
k__Bacteria;p__Firmicutes;c__Clostridia;o__Clostridiales;f__Ruminococcaceae;g__Ruminiclostridium	0.01 ± 0.00	0	0.02 ± 0.01	0	0.2975	-	-	-
k__Bacteria;p__Firmicutes;c__Clostridia;o__Clostridiales;f__Ruminococcaceae;g__Ruminococcus	0.20 ± 0.06	8.2	0.89 ± 0.27	30	1.00E-04	-	3.6077	0.0001
**k__Bacteria;p__Firmicutes;c__Clostridia;o__Clostridiales;f__Ruminococcaceae;g__Saccharofermentans**	**7.85 ± 0.73**	**75.3**	**0.01 ± 0.01**	**0**	**0**	**4.6021**	**-**	**0.0000**
k__Bacteria;p__Firmicutes;c__Clostridia;o__Clostridiales;f__Ruminococcaceae;g__Sporobacter	0.11 ± 0.03	1.4	0.58 ± 0.23	20	1.00E-04	-	3.4795	0.0001
k__Bacteria;p__Firmicutes;c__Clostridia;o__Clostridiales;f__Ruminococcaceae;g__Subdoligranulum	0.00 ± 0.00	0	0.01 ± 0.01	0	0.0016	-	-	0.0017
k__Bacteria;p__Firmicutes;c__Clostridia;o__Clostridiales;f__Syntrophomonadaceae;g__Syntrophomonas	0.00 ± 0.00	0	0.00 ± 0.00	0	0.0077	-	2.5057	0.0073
k__Bacteria;p__Firmicutes;c__Erysipelotrichia;o__Erysipelotrichales;f__Erysipelotrichaceae;g__	0.46 ± 0.08	15.1	0.20 ± 0.09	0	0.1305	-	-	-
k__Bacteria;p__Firmicutes;c__Erysipelotrichia;o__Erysipelotrichales;f__Erysipelotrichaceae;g__Bulleidia	0.01 ± 0.00	0	0.08 ± 0.02	0	1.00E-04	-	2.5285	0.0001
k__Bacteria;p__Firmicutes;c__Erysipelotrichia;o__Erysipelotrichales;f__Erysipelotrichaceae;g__Catenibacterium	0.13 ± 0.04	4.1	0.76 ± 0.29	20	1.00E-04	-	3.5026	0.0001
k__Bacteria;p__Firmicutes;c__Erysipelotrichia;o__Erysipelotrichales;f__Erysipelotrichaceae;g__Clostridium	0.05 ± 0.02	0	0.21 ± 0.07	0	0	-	2.8639	0.0000
k__Bacteria;p__Firmicutes;c__Erysipelotrichia;o__Erysipelotrichales;f__Erysipelotrichaceae;g__Erysipelatoclostridium	0.00 ± 0.00	0	0.00 ± 0.00	0	0.0077	-	2.2205	0.0073
k__Bacteria;p__Firmicutes;c__Erysipelotrichia;o__Erysipelotrichales;f__Erysipelotrichaceae;g__Erysipelotrichaceae_UCG-004	0.00 ± 0.00	0	0.01 ± 0.01	0	0.0972	-	-	-
k__Bacteria;p__Firmicutes;c__Erysipelotrichia;o__Erysipelotrichales;f__Erysipelotrichaceae;g__Holdemanella	0.01 ± 0.00	0	0.04 ± 0.01	0	1.00E-04	-	2.1778	0.0001
k__Bacteria;p__Firmicutes;c__Erysipelotrichia;o__Erysipelotrichales;f__Erysipelotrichaceae;g__Holdemania	0.00 ± 0.00	0	0.00 ± 0.00	0	0.4623	-	-	-
k__Bacteria;p__Firmicutes;c__Erysipelotrichia;o__Erysipelotrichales;f__Erysipelotrichaceae;g__Longibaculum	0.02 ± 0.01	0	0.08 ± 0.04	0	8.00E-04	-	2.4997	0.0008
k__Bacteria;p__Firmicutes;c__Erysipelotrichia;o__Erysipelotrichales;f__Erysipelotrichaceae;g__Solobacterium	0.00 ± 0.00	0	0.00 ± 0.00	0	0.0972	-	-	-
k__Bacteria;p__Firmicutes;c__Negativicutes;o__Acidaminococcales;f__Acidaminococcaceae;g__Phascolarctobacterium	0.58 ± 0.36	6.8	0.96 ± 0.24	40	2.00E-04	-	3.4378	0.0002
k__Bacteria;p__Firmicutes;c__Negativicutes;o__Selenomonadales;f__Selenomonadaceae;g__	0.40 ± 0.09	16.4	0.03 ± 0.02	0	0.1406	-	-	-
k__Bacteria;p__Firmicutes;c__Negativicutes;o__Selenomonadales;f__Selenomonadaceae;g__Mitsuokella	0.00 ± 0.00	0	0.00 ± 0.00	0	0.6169	-	-	-

k__Bacteria;p__Firmicutes;c__Negativicutes;o__Selenomonadales;f__Selenomonadaceae;g__Propionispira	0.41 ± 0.17	9.6	0.96 ± 0.29	40	4.00E-04	-	3.4916	0.0005
k__Bacteria;p__Firmicutes;c__Negativicutes;o__Selenomonadales;f__Selenomonadaceae;g__Selenomonas	1.10 ± 0.17	37	0.31 ± 0.11	10	0.0756	-	-	-
k__Bacteria;p__Firmicutes;c__Negativicutes;o__Veillonellales;f__Veillonellaceae;g__Allisonella	0.00 ± 0.00	0	0.02 ± 0.01	0	0.0185	-	2.0564	0.0186
**k__Bacteria;p__Firmicutes;c__Negativicutes;o__Veillonellales;f__Veillonellaceae;g__Dialister**	**6.15 ± 0.66**	**91.8**	**1.01 ± 0.27**	**30**	**0**	**4.3941**	**-**	**0.0000**
**k__Bacteria;p__Firmicutes;c__Negativicutes;o__Veillonellales;f__Veillonellaceae;g__Megasphaera**	**0.03 ± 0.01**	**0**	**1.09 ± 0.98**	**10**	**0.0359**	**-**	**3.7575**	**0.0375**
k__Bacteria;p__Firmicutes;c__Negativicutes;o__Veillonellales;f__Veillonellaceae;g__Veillonella	0.01 ± 0.00	0	0.02 ± 0.02	0	0.3145	-	-	-
k__Bacteria;p__Firmicutes;c__Tissierellia;o__Tissierellales;f__Peptoniphilaceae;g__	2.22 ± 0.30	60.3	2.48 ± 0.91	60	0.9275	-	-	-
**k__Bacteria;p__Firmicutes;c__Tissierellia;o__Tissierellales;f__Peptoniphilaceae;g__Anaerococcus**	**1.46 ± 0.39**	**24.7**	**5.53 ± 3.95**	**50**	**0.0141**	**-**	**4.2677**	**0.0131**
k__Bacteria;p__Firmicutes;c__Tissierellia;o__Tissierellales;f__Peptoniphilaceae;g__Anaerosphaera	0.11 ± 0.07	2.7	0.98 ± 0.53	30	1.00E-04	-	3.5811	0.0001
k__Bacteria;p__Firmicutes;c__Tissierellia;o__Tissierellales;f__Peptoniphilaceae;g__Finegoldia	0.30 ± 0.14	5.5	2.03 ± 1.39	30	0.0518	-	-	-
k__Bacteria;p__Firmicutes;c__Tissierellia;o__Tissierellales;f__Peptoniphilaceae;g__Gallicola	0.00 ± 0.00	0	0.00 ± 0.00	0	0.7391	-	-	-
k__Bacteria;p__Firmicutes;c__Tissierellia;o__Tissierellales;f__Peptoniphilaceae;g__Helcococcus	0.22 ± 0.07	6.8	0.16 ± 0.11	10	0.6146	-	-	-
**k__Bacteria;p__Firmicutes;c__Tissierellia;o__Tissierellales;f__Peptoniphilaceae;g__Parvimonas**	**1.14 ± 0.16**	**37**	**0.00 ± 0.00**	**0**	**1.00E-04**	**3.7205**	**-**	**0.0001**
k__Bacteria;p__Firmicutes;c__Tissierellia;o__Tissierellales;f__Peptoniphilaceae;g__Peptoniphilus	2.06 ± 0.28	56.2	1.34 ± 0.41	50	0.4542	-	-	-
k__Bacteria;p__Firmicutes;c__Tissierellia;o__Tissierellales;f__Tissierellaceae;g__	0.16 ± 0.12	1.4	0.56 ± 0.27	20	0	-	3.3071	0.0000
k__Bacteria;p__Firmicutes;c__Tissierellia;o__Tissierellales;f__Tissierellaceae;g__Tissierella	0.04 ± 0.01	0	0.01 ± 0.00	0	0.1633	-	-	-
k__Bacteria;p__Fusobacteria;c__Fusobacteriia;o__Fusobacteriales;f__Fusobacteriaceae;g__Cetobacterium	0.00 ± 0.00	0	0.05 ± 0.04	0	1.00E-04	-	2.2767	0.0001
k__Bacteria;p__Fusobacteria;c__Fusobacteriia;o__Fusobacteriales;f__Fusobacteriaceae;g__Fusobacterium	5.14 ± 0.89	57.5	6.89 ± 3.54	50	0.8886	-	-	-
k__Bacteria;p__Fusobacteria;c__Fusobacteriia;o__Fusobacteriales;f__Leptotrichiaceae;g__Leptotrichia	0.00 ± 0.00	0	0.00 ± 0.00	0	6.00E-04	-	-	0.0006
**k__Bacteria;p__Fusobacteria;c__Fusobacteriia;o__Fusobacteriales;f__Leptotrichiaceae;g__Sneathia**	**5.65 ± 0.71**	**76.7**	**0.12 ± 0.07**	**0**	**0**	**4.4507**	**-**	**0.0001**
k__Bacteria;p__Fusobacteria;c__Fusobacteriia;o__Fusobacteriales;f__Leptotrichiaceae;g__Streptobacillus	0.00 ± 0.00	0	0.00 ± 0.00	0	0.5305	-	-	-
k__Bacteria;p__Gemmatimonadetes;c__;o__;f__;g__	0.00 ± 0.00	0	0.00 ± 0.00	0	0.7391	-	-	-
k__Bacteria;p__Gemmatimonadetes;c__Gemmatimonadetes;o__Gemmatimonadales;f__Gemmatimonadaceae;g__Gemmatirosa	0.00 ± 0.00	0	0.01 ± 0.01	0	0.0972	-	-	-
k__Bacteria;p__Lentisphaerae;c__Lentisphaeria;o__Victivallales;f__;g__	0.00 ± 0.00	0	0.03 ± 0.01	0	4.00E-04	-	2.1827	0.0004
k__Bacteria;p__Lentisphaerae;c__Lentisphaeria;o__Victivallales;f__Victivallaceae;g__	0.00 ± 0.00	0	0.00 ± 0.00	0	0.6969	-	-	-
k__Bacteria;p__Lentisphaerae;c__Oligosphaeria;o__Oligosphaerales;f__Oligosphaeraceae;g__	0.00 ± 0.00	0	0.01 ± 0.01	0	0	-	-	0.0000
k__Bacteria;p__Planctomycetes;c__Planctomycetia;o__Planctomycetales;f__Isosphaeraceae;g__Aquisphaera	0.00 ± 0.00	0	0.00 ± 0.00	0	0.0077	-	2.2218	0.0073
k__Bacteria;p__Proteobacteria;c__Alphaproteobacteria;o__Caulobacterales;f__Caulobacteraceae;g__Brevundimonas	0.01 ± 0.01	0	0.05 ± 0.03	0	8.00E-04	-	2.3895	0.0008
k__Bacteria;p__Proteobacteria;c__Alphaproteobacteria;o__Caulobacterales;f__Caulobacteraceae;g__Caulobacter	0.00 ± 0.00	0	0.00 ± 0.00	0	0.0077	-	2.4707	0.0073
k__Bacteria;p__Proteobacteria;c__Alphaproteobacteria;o__Rhizobiales;f__Aurantimonadaceae;g__Aureimonas	0.00 ± 0.00	0	0.01 ± 0.01	0	0.0077	-	2.4511	0.0073
k__Bacteria;p__Proteobacteria;c__Alphaproteobacteria;o__Rhizobiales;f__Bradyrhizobiaceae;g__Bosea	0.00 ± 0.00	0	0.00 ± 0.00	0	0.2795	-	-	-
k__Bacteria;p__Proteobacteria;c__Alphaproteobacteria;o__Rhizobiales;f__Bradyrhizobiaceae;g__Bradyrhizobium	0.00 ± 0.00	0	0.01 ± 0.01	0	0.0077	-	2.1054	0.0073
k__Bacteria;p__Proteobacteria;c__Alphaproteobacteria;o__Rhizobiales;f__Brucellaceae;g__Pseudochrobactrum	0.00 ± 0.00	0	0.00 ± 0.00	0	0.1082	-	-	-
k__Bacteria;p__Proteobacteria;c__Alphaproteobacteria;o__Rhizobiales;f__Hyphomicrobiaceae;g__Devosia	0.00 ± 0.00	0	0.01 ± 0.00	0	0.004	-	-	0.0040
k__Bacteria;p__Proteobacteria;c__Alphaproteobacteria;o__Rhizobiales;f__Hyphomicrobiaceae;g__Hyphomicrobium	0.00 ± 0.00	0	0.00 ± 0.00	0	0.0077	-	2.3110	0.0073
k__Bacteria;p__Proteobacteria;c__Alphaproteobacteria;o__Rhizobiales;f__Methylobacteriaceae;g__Methylobacterium	0.00 ± 0.00	0	0.01 ± 0.01	0	0.0131	-	-	0.0136
k__Bacteria;p__Proteobacteria;c__Alphaproteobacteria;o__Rhizobiales;f__Phyllobacteriaceae;g__Mesorhizobium	0.00 ± 0.00	0	0.00 ± 0.00	0	0.7391	-	-	-
k__Bacteria;p__Proteobacteria;c__Alphaproteobacteria;o__Rhodobacterales;f__Rhodobacteraceae;g__	0.00 ± 0.00	0	0.00 ± 0.00	0	0.004	-	-	0.0040
k__Bacteria;p__Proteobacteria;c__Alphaproteobacteria;o__Rhodobacterales;f__Rhodobacteraceae;g__Amaricoccus	0.00 ± 0.00	0	0.40 ± 0.40	10	0.0077	-	3.3090	0.0073
k__Bacteria;p__Proteobacteria;c__Alphaproteobacteria;o__Rhodobacterales;f__Rhodobacteraceae;g__Aquimixticola	0.01 ± 0.01	0	0.01 ± 0.01	0	0.004	-	-	0.0040
k__Bacteria;p__Proteobacteria;c__Alphaproteobacteria;o__Rhodobacterales;f__Rhodobacteraceae;g__Gemmobacter	0.00 ± 0.00	0	0.01 ± 0.01	0	0.0035	-	-	0.0035
k__Bacteria;p__Proteobacteria;c__Alphaproteobacteria;o__Rhodobacterales;f__Rhodobacteraceae;g__Paracoccus	0.01 ± 0.01	0	0.02 ± 0.02	0	1.00E-04	-	2.0853	0.0001
k__Bacteria;p__Proteobacteria;c__Alphaproteobacteria;o__Rhodobacterales;f__Rhodobacteraceae;g__Rubellimicrobium	0.00 ± 0.00	0	0.00 ± 0.00	0	0.0077	-	-	0.0073
k__Bacteria;p__Proteobacteria;c__Alphaproteobacteria;o__Rhodospirillales;f__Acetobacteraceae;g__	0.00 ± 0.00	0	0.02 ± 0.01	0	0.0058	-	2.0849	0.0060
k__Bacteria;p__Proteobacteria;c__Alphaproteobacteria;o__Rhodospirillales;f__Acetobacteraceae;g__Belnapia	0.00 ± 0.00	0	0.00 ± 0.00	0	0.0077	-	-	0.0073
k__Bacteria;p__Proteobacteria;c__Alphaproteobacteria;o__Rhodospirillales;f__Acetobacteraceae;g__Rhodovastum	0.00 ± 0.00	0	0.00 ± 0.00	0	0.0077	-	2.3434	0.0073
k__Bacteria;p__Proteobacteria;c__Alphaproteobacteria;o__Rhodospirillales;f__Rhodospirillaceae;g__	0.01 ± 0.01	0	0.45 ± 0.20	10	0	-	3.4317	0.0000
k__Bacteria;p__Proteobacteria;c__Alphaproteobacteria;o__Rhodospirillales;f__Rhodospirillaceae;g__Ferrovibrio	0.00 ± 0.00	0	0.00 ± 0.00	0	0.7391	-	-	-
k__Bacteria;p__Proteobacteria;c__Alphaproteobacteria;o__Rhodospirillales;f__Rhodospirillaceae;g__Niveispirillum	0.00 ± 0.00	0	0.00 ± 0.00	0	0.7391	-	-	-
k__Bacteria;p__Proteobacteria;c__Alphaproteobacteria;o__Rhodospirillales;f__Rhodospirillaceae;g__Tistrella	0.00 ± 0.00	0	0.00 ± 0.00	0	0.5305	-	-	-
k__Bacteria;p__Proteobacteria;c__Alphaproteobacteria;o__Rickettsiales;f__;g__	0.00 ± 0.00	0	0.00 ± 0.00	0	0.7391	-	-	-
k__Bacteria;p__Proteobacteria;c__Alphaproteobacteria;o__Sphingomonadales;f__Sphingomonadaceae;g__	0.00 ± 0.00	0	0.00 ± 0.00	0	0.1082	-	-	-
k__Bacteria;p__Proteobacteria;c__Alphaproteobacteria;o__Sphingomonadales;f__Sphingomonadaceae;g__Novosphingobium	0.00 ± 0.00	0	0.00 ± 0.00	0	0.7436	-	-	-
k__Bacteria;p__Proteobacteria;c__Alphaproteobacteria;o__Sphingomonadales;f__Sphingomonadaceae;g__Sphingobium	0.00 ± 0.00	0	0.00 ± 0.00	0	0.7391	-	-	-
k__Bacteria;p__Proteobacteria;c__Alphaproteobacteria;o__Sphingomonadales;f__Sphingomonadaceae;g__Sphingomonas	0.00 ± 0.00	0	0.03 ± 0.01	0	6.00E-04	-	2.0805	0.0006
k__Bacteria;p__Proteobacteria;c__Alphaproteobacteria;o__Sphingomonadales;f__Sphingomonadaceae;g__Sphingopyxis	0.00 ± 0.00	0	0.00 ± 0.00	0	0.0972	-	-	-

k__Bacteria;p__Proteobacteria;c__Alphaproteobacteria;o__Sphingomonadales;f__Sphingomonadaceae;g__Sphingorhabdus	0.00 ± 0.00	0	0.00 ± 0.00	0	0.0077	-	2.0954	0.0073
k__Bacteria;p__Proteobacteria;c__Betaproteobacteria;o__Burkholderiales;f__Alcaligenaceae;g__Achromobacter	0.00 ± 0.00	0	0.01 ± 0.01	0	0.5642	-	-	-
k__Bacteria;p__Proteobacteria;c__Betaproteobacteria;o__Burkholderiales;f__Alcaligenaceae;g__Alcaligenes	0.00 ± 0.00	0	0.01 ± 0.00	0	2.00E-04	-	-	0.0003
k__Bacteria;p__Proteobacteria;c__Betaproteobacteria;o__Burkholderiales;f__Alcaligenaceae;g__Parapusillimonas	0.00 ± 0.00	0	0.00 ± 0.00	0	0.0077	-	2.4573	0.0073
k__Bacteria;p__Proteobacteria;c__Betaproteobacteria;o__Burkholderiales;f__Alcaligenaceae;g__Pelistega	0.02 ± 0.02	1.4	0.01 ± 0.01	0	1.00E-04	-	-	0.0001
k__Bacteria;p__Proteobacteria;c__Betaproteobacteria;o__Burkholderiales;f__Burkholderiaceae;g__Burkholderia	0.00 ± 0.00	0	0.00 ± 0.00	0	0.3964	-	-	-
k__Bacteria;p__Proteobacteria;c__Betaproteobacteria;o__Burkholderiales;f__Burkholderiaceae;g__Cupriavidus	0.00 ± 0.00	0	0.00 ± 0.00	0	0.7391	-	-	-
k__Bacteria;p__Proteobacteria;c__Betaproteobacteria;o__Burkholderiales;f__Burkholderiaceae;g__Limnobacter	0.00 ± 0.00	0	0.00 ± 0.00	0	0.7391	-	-	-
k__Bacteria;p__Proteobacteria;c__Betaproteobacteria;o__Burkholderiales;f__Burkholderiaceae;g__Paraburkholderia	0.00 ± 0.00	0	0.00 ± 0.00	0	0.2428	-	-	-
k__Bacteria;p__Proteobacteria;c__Betaproteobacteria;o__Burkholderiales;f__Burkholderiaceae;g__Ralstonia	0.00 ± 0.00	0	0.01 ± 0.00	0	0.0181	-	-	0.0186
k__Bacteria;p__Proteobacteria;c__Betaproteobacteria;o__Burkholderiales;f__Comamonadaceae;g__	0.01 ± 0.00	0	0.03 ± 0.02	0	0.0015	-	2.2252	0.0016
k__Bacteria;p__Proteobacteria;c__Betaproteobacteria;o__Burkholderiales;f__Comamonadaceae;g__Comamonas	0.01 ± 0.01	0	0.02 ± 0.02	0	0.0185	-	2.1297	0.0186
k__Bacteria;p__Proteobacteria;c__Betaproteobacteria;o__Burkholderiales;f__Comamonadaceae;g__Pelomonas	0.00 ± 0.00	0	0.00 ± 0.00	0	0.1771	-	-	-
k__Bacteria;p__Proteobacteria;c__Betaproteobacteria;o__Burkholderiales;f__Oxalobacteraceae;g__Herbaspirillum	0.01 ± 0.00	0	0.01 ± 0.01	0	0.1945	-	-	-
k__Bacteria;p__Proteobacteria;c__Betaproteobacteria;o__Burkholderiales;f__Oxalobacteraceae;g__Janthinobacterium	0.00 ± 0.00	0	0.00 ± 0.00	0	0.004	-	-	0.0040
k__Bacteria;p__Proteobacteria;c__Betaproteobacteria;o__Burkholderiales;f__Oxalobacteraceae;g__Massilia	0.00 ± 0.00	0	0.00 ± 0.00	0	0.7391	-	-	-
k__Bacteria;p__Proteobacteria;c__Betaproteobacteria;o__Burkholderiales;f__Roseateles;g__	0.01 ± 0.00	0	0.28 ± 0.14	10	0	-	3.2189	0.0000
k__Bacteria;p__Proteobacteria;c__Betaproteobacteria;o__Burkholderiales;f__Rubrivivax;g__gelatinosus	0.02 ± 0.02	1.4	0.06 ± 0.04	0	0.0185	-	2.4503	0.0186
k__Bacteria;p__Proteobacteria;c__Betaproteobacteria;o__Burkholderiales;f__Sutterellaceae;g__Sutterella	0.01 ± 0.01	0	0.03 ± 0.02	0	0.1407	-	-	-
k__Bacteria;p__Proteobacteria;c__Betaproteobacteria;o__Methylophilales;f__Methylophilaceae;g__	0.00 ± 0.00	0	0.00 ± 0.00	0	0.7391	-	-	-
k__Bacteria;p__Proteobacteria;c__Betaproteobacteria;o__Neisseriales;f__Neisseriaceae;g__	0.00 ± 0.00	0	0.03 ± 0.02	0	6.00E-04	-	2.0066	0.0006
k__Bacteria;p__Proteobacteria;c__Betaproteobacteria;o__Neisseriales;f__Neisseriaceae;g__Neisseria	0.00 ± 0.00	0	0.00 ± 0.00	0	0.0077	-	-	0.0073
k__Bacteria;p__Proteobacteria;c__Betaproteobacteria;o__Rhodocyclales;f__Rhodocyclaceae;g__Azoarcus	0.00 ± 0.00	0	0.00 ± 0.00	0	0.7391	-	-	-
k__Bacteria;p__Proteobacteria;c__Deltaproteobacteria;o__Bradymonadales;f__;g__	0.02 ± 0.01	1.4	0.09 ± 0.06	0	0.0175	-	2.7194	0.0184
k__Bacteria;p__Proteobacteria;c__Deltaproteobacteria;o__Desulfovibrionales;f__;g__	0.00 ± 0.00	0	0.00 ± 0.00	0	0.0077	-	2.6313	0.0073
k__Bacteria;p__Proteobacteria;c__Deltaproteobacteria;o__Desulfovibrionales;f__Desulfohalobiaceae;g__	0.00 ± 0.00	0	0.00 ± 0.00	0	1.00E-04	-	-	0.0001
k__Bacteria;p__Proteobacteria;c__Deltaproteobacteria;o__Desulfovibrionales;f__Desulfovibrionaceae;g__	0.00 ± 0.00	0	0.00 ± 0.00	0	0.674	-	-	-
k__Bacteria;p__Proteobacteria;c__Deltaproteobacteria;o__Desulfovibrionales;f__Desulfovibrionaceae;g__Desulfovibrio	0.01 ± 0.00	0	0.29 ± 0.14	10	0.0018	-	3.2216	0.0019
k__Bacteria;p__Proteobacteria;c__Deltaproteobacteria;o__Desulfuromonadales;f__Geobacteraceae;g__Geobacter	0.00 ± 0.00	0	0.00 ± 0.00	0	0.6109	-	-	-
k__Bacteria;p__Proteobacteria;c__Deltaproteobacteria;o__Myxococcales;f__Phaselicystidaceae;g__Phaselicystis	0.00 ± 0.00	0	0.00 ± 0.00	0	0.7391	-	-	-
k__Bacteria;p__Proteobacteria;c__Epsilonproteobacteria;o__Campylobacterales;f__Campylobacteraceae;g__	0.00 ± 0.00	0	0.01 ± 0.01	0	0.0077	-	2.1395	0.0073
k__Bacteria;p__Proteobacteria;c__Epsilonproteobacteria;o__Campylobacterales;f__Campylobacteraceae;g__Arcobacter	0.00 ± 0.00	0	0.00 ± 0.00	0	0.1082	-	-	-
**k__Bacteria;p__Proteobacteria;c__Epsilonproteobacteria;o__Campylobacterales;f__Campylobacteraceae;g__Campylobacter**	**9.08 ± 0.82**	**86.3**	**4.06 ± 1.08**	**80**	**0.0353**	**4.4232**	**-**	**0.0302**
**k__Bacteria;p__Proteobacteria;c__Epsilonproteobacteria;o__Campylobacterales;f__Helicobacteraceae;g__Helicobacter**	**0.33 ± 0.11**	**8.2**	**1.54 ± 0.79**	**30**	**2.00E-04**	**-**	**3.6497**	**0.0002**
k__Bacteria;p__Proteobacteria;c__Gammaproteobacteria;o__;f__;g__	0.01 ± 0.00	0	0.29 ± 0.14	10	0.0044	-	3.2643	0.0047
k__Bacteria;p__Proteobacteria;c__Gammaproteobacteria;o__Aeromonadales;f__Aeromonadaceae;g__Aeromonas	0.00 ± 0.00	0	0.00 ± 0.00	0	0.7391	-	-	-
**k__Bacteria;p__Proteobacteria;c__Gammaproteobacteria;o__Aeromonadales;f__Succinivibrionaceae;g__Succinivibrio**	**0.14 ± 0.05**	**2.7**	**1.05 ± 0.42**	**30**	**0.0025**	**-**	**3.7647**	**0.0027**
k__Bacteria;p__Proteobacteria;c__Gammaproteobacteria;o__Alteromonadales;f__Alteromonadaceae;g__Marinobacter	0.00 ± 0.00	0	0.00 ± 0.00	0	0.9238	-	-	-
k__Bacteria;p__Proteobacteria;c__Gammaproteobacteria;o__Alteromonadales;f__Idiomarinaceae;g__Aliidiomarina	0.01 ± 0.00	0	0.00 ± 0.00	0	0.2939	-	-	-
k__Bacteria;p__Proteobacteria;c__Gammaproteobacteria;o__Cardiobacteriales;f__Cardiobacteriaceae;g__Suttonella	0.02 ± 0.02	1.4	0.00 ± 0.00	0	0.2795	-	-	-
k__Bacteria;p__Proteobacteria;c__Gammaproteobacteria;o__Enterobacterales;f__Enterobacteriaceae;g__	0.00 ± 0.00	0	0.01 ± 0.00	0	0.0311	-	-	0.0324
k__Bacteria;p__Proteobacteria;c__Gammaproteobacteria;o__Enterobacterales;f__Enterobacteriaceae;g__Enterobacter	0.04 ± 0.04	1.4	0.00 ± 0.00	0	0.4397	-	-	-
k__Bacteria;p__Proteobacteria;c__Gammaproteobacteria;o__Enterobacterales;f__Morganellaceae;g__	0.00 ± 0.00	0	0.03 ± 0.03	0	0.1082	-	-	-
k__Bacteria;p__Proteobacteria;c__Gammaproteobacteria;o__Enterobacterales;f__Morganellaceae;g__Providencia	0.00 ± 0.00	0	0.01 ± 0.01	0	0.004	-	-	0.0040
k__Bacteria;p__Proteobacteria;c__Gammaproteobacteria;o__Legionellales;f__Coxiellaceae;g__Diplorickettsia	0.00 ± 0.00	0	0.00 ± 0.00	0	0.0077	-	-	0.0073
k__Bacteria;p__Proteobacteria;c__Gammaproteobacteria;o__Oceanospirillales;f__Halomonadaceae;g__Chromohalobacter	0.00 ± 0.00	0	0.00 ± 0.00	0	0.6169	-	-	-
**k__Bacteria;p__Proteobacteria;c__Gammaproteobacteria;o__Oceanospirillales;f__Halomonadaceae;g__Halomonas**	**1.77 ± 0.57**	**20.5**	**7.33 ± 2.77**	**80**	**3.00E-04**	**-**	**4.4050**	**0.0003**
k__Bacteria;p__Proteobacteria;c__Gammaproteobacteria;o__Oceanospirillales;f__Oceanospirillaceae;g__Marinobacterium	0.00 ± 0.00	0	0.00 ± 0.00	0	0.7391	-	-	-
k__Bacteria;p__Proteobacteria;c__Gammaproteobacteria;o__Orbales;f__Orbaceae;g__	0.00 ± 0.00	0	0.00 ± 0.00	0	0.7391	-	-	-
k__Bacteria;p__Proteobacteria;c__Gammaproteobacteria;o__Pasteurellales;f__Pasteurellaceae;g__Aggregatibacter	0.00 ± 0.00	0	0.02 ± 0.01	0	0.0113	-	-	0.0118
**k__Bacteria;p__Proteobacteria;c__Gammaproteobacteria;o__Pasteurellales;f__Pasteurellaceae;g__Haemophilus**	**1.02 ± 0.62**	**6.8**	**1.77 ± 0.99**	**20**	**7.00E-04**	**-**	**3.6541**	**0.0007**
k__Bacteria;p__Proteobacteria;c__Gammaproteobacteria;o__Pseudomonadales;f__Moraxellaceae;g__Acinetobacter	0.04 ± 0.04	1.4	0.74 ± 0.48	20	0.0031	-	3.5971	0.0033
k__Bacteria;p__Proteobacteria;c__Gammaproteobacteria;o__Pseudomonadales;f__Moraxellaceae;g__Moraxella	0.00 ± 0.00	0	0.11 ± 0.09	0	0.003	-	2.8536	0.0031
k__Bacteria;p__Proteobacteria;c__Gammaproteobacteria;o__Pseudomonadales;f__Moraxellaceae;g__Psychrobacter	0.00 ± 0.00	0	0.03 ± 0.02	0	0.003	-	2.1904	0.0031
k__Bacteria;p__Proteobacteria;c__Gammaproteobacteria;o__Pseudomonadales;f__Pseudomonadaceae;g__Pseudomonas	0.01 ± 0.01	0	0.06 ± 0.03	0	0.0625	-	-	-
k__Bacteria;p__Proteobacteria;c__Gammaproteobacteria;o__Thiotrichales;f__Francisellaceae;g__Francisella	0.00 ± 0.00	0	0.01 ± 0.01	0	0.0077	-	2.3550	0.0073

k__Bacteria;p__Proteobacteria;c__Gammaproteobacteria;o__Xanthomonadales;f__;g__	0.00 ± 0.00	0	0.01 ± 0.01	0	0.0077	-	2.1566	0.0073
k__Bacteria;p__Proteobacteria;c__Gammaproteobacteria;o__Xanthomonadales;f__Rhodanobacteraceae;g__Dokdonella	0.00 ± 0.00	0	0.00 ± 0.00	0	0.6169	-	-	-
k__Bacteria;p__Proteobacteria;c__Gammaproteobacteria;o__Xanthomonadales;f__Xanthomonadaceae;g__	0.00 ± 0.00	0	0.00 ± 0.00	0	0.004	-	-	0.0040
k__Bacteria;p__Proteobacteria;c__Gammaproteobacteria;o__Xanthomonadales;f__Xanthomonadaceae;g__Luteimonas	0.01 ± 0.01	0	0.00 ± 0.00	0	0.1082	-	-	-
k__Bacteria;p__Proteobacteria;c__Gammaproteobacteria;o__Xanthomonadales;f__Xanthomonadaceae;g__Lysobacter	0.01 ± 0.01	0	0.00 ± 0.00	0	0.1082	-	-	-
k__Bacteria;p__Proteobacteria;c__Gammaproteobacteria;o__Xanthomonadales;f__Xanthomonadaceae;g__Stenotrophomonas	0.00 ± 0.00	0	0.00 ± 0.00	0	0.2795	-	-	-
k__Bacteria;p__Proteobacteria;c__Gammaproteobacteria;o__Xanthomonadales;f__Xanthomonadaceae;g__Xanthomonas	0.00 ± 0.00	0	0.00 ± 0.00	0	0.7391	-	-	-
k__Bacteria;p__Spirochaetes;c__Spirochaetia;o__Brachyspirales;f__Brachyspiraceae;g__Brachyspira	0.00 ± 0.00	0	0.03 ± 0.01	0	1.00E-04	-	2.1774	0.0001
**k__Bacteria;p__Spirochaetes;c__Spirochaetia;o__Spirochaetales;f__Spirochaetaceae;g__**	**4.00 ± 0.53**	**63**	**0.61 ± 0.40**	**20**	**0.0099**	**4.2539**	**-**	**0.0081**
k__Bacteria;p__Spirochaetes;c__Spirochaetia;o__Spirochaetales;f__Spirochaetaceae;g__Treponema	1.09 ± 0.21	28.8	0.12 ± 0.07	0	0.0783	-	-	-
k__Bacteria;p__Tenericutes;c__Mollicutes;o__Acholeplasmatales;f__Acholeplasmataceae;g__	0.00 ± 0.00	0	0.06 ± 0.02	0	4.00E-04	-	2.4456	0.0005
k__Bacteria;p__Tenericutes;c__Mollicutes;o__Anaeroplasmatales;f__Anaeroplasmataceae;g__	0.01 ± 0.01	0	0.05 ± 0.04	0	0.0134	-	2.3472	0.0141
k__Bacteria;p__Tenericutes;c__Mollicutes;o__Anaeroplasmatales;f__Anaeroplasmataceae;g__Anaeroplasma	0.00 ± 0.00	0	0.00 ± 0.00	0	0.358	-	-	-
k__Bacteria;p__Tenericutes;c__Mollicutes;o__Entomoplasmatales;f__Entomoplasmataceae;g__	0.00 ± 0.00	0	0.00 ± 0.00	0	0.0077	-	2.0879	0.0073
k__Bacteria;p__Tenericutes;c__Mollicutes;o__Entomoplasmatales;f__Spiroplasmataceae;g__	0.01 ± 0.00	0	0.06 ± 0.03	0	0.0507	-	-	-
k__Bacteria;p__Tenericutes;c__Mollicutes;o__Mollicutes_RF9;f__;g__	0.01 ± 0.01	0	0.11 ± 0.08	0	0.002	-	2.8353	0.0021
k__Bacteria;p__Tenericutes;c__Mollicutes;o__Mycoplasmatales;f__Mycoplasmataceae;g__	0.00 ± 0.00	0	0.00 ± 0.00	0	0.4177	-	-	-
k__Bacteria;p__Tenericutes;c__Mollicutes;o__Mycoplasmatales;f__Mycoplasmataceae;g__Mycoplasma	0.08 ± 0.03	2.7	0.01 ± 0.00	0	0.24	-	-	-
k__Bacteria;p__Verrucomicrobia;c__;o__;f__;g__	0.01 ± 0.00	0	0.15 ± 0.08	0	0.0128	-	2.9799	0.0134
k__Bacteria;p__Verrucomicrobia;c__Opitutae;o__Opitutae_vadinHA64;f__;g__	0.00 ± 0.00	0	0.00 ± 0.00	0	0.7391	-	-	-
k__Bacteria;p__Verrucomicrobia;c__Opitutae;o__Puniceicoccales;f__Puniceicoccaceae;g__	0.00 ± 0.00	0	0.02 ± 0.02	0	0.0472	-	2.0950	0.0475
k__Bacteria;p__Verrucomicrobia;c__Opitutae;o__Puniceicoccales;f__Puniceicoccaceae;g__Cerasicoccus	0.00 ± 0.00	0	0.00 ± 0.00	0	0.0077	-	-	0.0073

[$] mean ± s.e.m

* Individual samples with >1% abundance were counted.

Table S14. Relative abundance of vaginal bacterial taxa (L6, at the genus and above levels) between adult female macaques with (Yes) and without (No) tubectomy

Vaginal microbiota in adult female macaques with or without tubectomy	Yes		No					
	Abundance[$] (%)	Prevalence* (%)	Abundance[$] (%)	Prevalence* (%)	MW test (p)	Yes	No	p value
k__Bacteria;p__Acidobacteria;c__Acidobacteriia;o__Acidobacteriales;f__Acidobacteriaceae;g__	0.00 ± 0.00	0.0	0.00 ± 0.00	0.0	0.0533	-	-	-
k__Bacteria;p__Acidobacteria;c__Holophagae;o__;f__;g__	0.00 ± 0.00	0.0	0.00 ± 0.00	0.0	0.6350	-	-	-
k__Bacteria;p__Actinobacteria;c__Actinobacteria;o__Actinomycetales;f__Actinomycetaceae;g__	0.04 ± 0.03	1.7	0.06 ± 0.03	0.0	0.0846	-	-	-
k__Bacteria;p__Actinobacteria;c__Actinobacteria;o__Actinomycetales;f__Actinomycetaceae;g__Actinomyces	0.00 ± 0.00	0.0	0.07 ± 0.06	0.0	0.1321	-	-	-
k__Bacteria;p__Actinobacteria;c__Actinobacteria;o__Actinomycetales;f__Actinomycetaceae;g__Arcanobacterium	0.01 ± 0.00	0.0	0.07 ± 0.03	0.0	0.3643	-	-	-
k__Bacteria;p__Actinobacteria;c__Actinobacteria;o__Actinomycetales;f__Actinomycetaceae;g__Flaviflexus	0.00 ± 0.00	0.0	0.01 ± 0.01	0.0	0.0533	-	-	-
**k__Bacteria;p__Actinobacteria;c__Actinobacteria;o__Actinomycetales;f__Actinomycetaceae;g__Mobiluncus**	**2.02 ± 0.25**	**72.4**	**1.54 ± 0.67**	**40.0**	**0.0282**	**3.4955**	**-**	**0.0267**
k__Bacteria;p__Actinobacteria;c__Actinobacteria;o__Actinomycetales;f__Actinomycetaceae;g__Trueperella	0.01 ± 0.00	0.0	0.05 ± 0.03	0.0	0.0219	-	2.3537	0.0238
k__Bacteria;p__Actinobacteria;c__Actinobacteria;o__Bifidobacteriales;f__Bifidobacteriaceae;g__Alloscardovia	0.00 ± 0.00	0.0	0.00 ± 0.00	0.0	0.0533	-	-	-
k__Bacteria;p__Actinobacteria;c__Actinobacteria;o__Bifidobacteriales;f__Bifidobacteriaceae;g__Bifidobacterium	0.00 ± 0.00	0.0	0.05 ± 0.03	0.0	0.2274	-	-	-
k__Bacteria;p__Actinobacteria;c__Actinobacteria;o__Bifidobacteriales;f__Bifidobacteriaceae;g__Gardnerella	0.31 ± 0.10	10.3	1.14 ± 0.98	13.3	0.5811	-	-	-
k__Bacteria;p__Actinobacteria;c__Actinobacteria;o__Corynebacteriales;f__Corynebacteriaceae;g__Corynebacterium	0.83 ± 0.30	13.8	2.95 ± 1.38	33.3	0.2427	-	-	-
k__Bacteria;p__Actinobacteria;c__Actinobacteria;o__Corynebacteriales;f__Dietziaceae;g__Dietzia	0.00 ± 0.00	0.0	0.03 ± 0.03	0.0	0.6235	-	-	-
k__Bacteria;p__Actinobacteria;c__Actinobacteria;o__Micrococcales;f__Bogoriellaceae;g__Bogoriella	0.00 ± 0.00	0.0	0.00 ± 0.00	0.0	0.0533	-	-	-
k__Bacteria;p__Actinobacteria;c__Actinobacteria;o__Micrococcales;f__Brevibacteriaceae;g__Brevibacterium	0.00 ± 0.00	0.0	0.12 ± 0.12	6.7	0.0533	-	-	-
k__Bacteria;p__Actinobacteria;c__Actinobacteria;o__Micrococcales;f__Dermabacteraceae;g__Brachybacterium	0.00 ± 0.00	0.0	0.02 ± 0.02	0.0	0.0533	-	-	-
k__Bacteria;p__Actinobacteria;c__Actinobacteria;o__Micrococcales;f__Dermatophilaceae;g__Dermatophilus	0.00 ± 0.00	0.0	0.01 ± 0.01	0.0	0.0533	-	-	-
k__Bacteria;p__Actinobacteria;c__Actinobacteria;o__Micrococcales;f__Dermatophilaceae;g__Piscicoccus	0.00 ± 0.00	0.0	0.00 ± 0.00	0.0	0.0533	-	-	-
k__Bacteria;p__Actinobacteria;c__Actinobacteria;o__Micrococcales;f__Intrasporangiaceae;g__Knoellia	0.00 ± 0.00	0.0	0.00 ± 0.00	0.0	0.0533	-	-	-
k__Bacteria;p__Actinobacteria;c__Actinobacteria;o__Micrococcales;f__Microbacteriaceae;g__	0.00 ± 0.00	0.0	0.03 ± 0.03	0.0	0.0006	-	2.3224	0.0006
k__Bacteria;p__Actinobacteria;c__Actinobacteria;o__Micrococcales;f__Microbacteriaceae;g__Amnibacterium	0.00 ± 0.00	0.0	0.00 ± 0.00	0.0	0.0533	-	-	-
k__Bacteria;p__Actinobacteria;c__Actinobacteria;o__Micrococcales;f__Microbacteriaceae;g__Leucobacter	0.00 ± 0.00	0.0	0.02 ± 0.02	0.0	0.0533	-	-	-
k__Bacteria;p__Actinobacteria;c__Actinobacteria;o__Micrococcales;f__Micrococcaceae;g__Arthrobacter	0.00 ± 0.00	0.0	0.02 ± 0.02	0.0	0.0533	-	-	-
k__Bacteria;p__Actinobacteria;c__Actinobacteria;o__Micrococcales;f__Micrococcaceae;g__Kocuria	0.00 ± 0.00	0.0	0.13 ± 0.13	6.7	0.0533	-	-	-
k__Bacteria;p__Actinobacteria;c__Actinobacteria;o__Micrococcales;f__Micrococcaceae;g__Micrococcus	0.00 ± 0.00	0.0	0.02 ± 0.02	0.0	0.0533	-	-	-
k__Bacteria;p__Actinobacteria;c__Actinobacteria;o__Micrococcales;f__Micrococcaceae;g__Rothia	0.00 ± 0.00	0.0	0.01 ± 0.01	0.0	0.0055	-	2.1397	0.0055
k__Bacteria;p__Actinobacteria;c__Actinobacteria;o__Pseudonocardiales;f__Pseudonocardiaceae;g__Saccharopolyspora	0.00 ± 0.00	0.0	0.00 ± 0.00	0.0	0.0533	-	-	-
k__Bacteria;p__Actinobacteria;c__Actinobacteria;o__Streptosporangiales;f__Thermomonosporaceae;g__Actinoallomurus	0.00 ± 0.00	0.0	0.00 ± 0.00	0.0	0.0533	-	-	-
k__Bacteria;p__Actinobacteria;c__Coriobacteriia;o__Coriobacteriales;f__Atopobiaceae;g__	0.10 ± 0.02	0.0	0.09 ± 0.05	0.0	0.0506	-	-	-
k__Bacteria;p__Actinobacteria;c__Coriobacteriia;o__Coriobacteriales;f__Atopobiaceae;g__Atopobium	1.89 ± 0.31	50.0	4.31 ± 2.03	46.7	0.8538	-	-	-
k__Bacteria;p__Actinobacteria;c__Coriobacteriia;o__Coriobacteriales;f__Atopobiaceae;g__Olsenella	0.01 ± 0.01	0.0	0.05 ± 0.02	0.0	0.0103	-	2.3369	0.0113
k__Bacteria;p__Actinobacteria;c__Coriobacteriia;o__Coriobacteriales;f__Coriobacteriaceae;g__	0.01 ± 0.00	0.0	0.01 ± 0.01	0.0	0.1831	-	-	-
k__Bacteria;p__Actinobacteria;c__Coriobacteriia;o__Coriobacteriales;f__Coriobacteriaceae;g__Collinsella	0.01 ± 0.00	0.0	0.03 ± 0.01	0.0	0.0506	-	-	-
k__Bacteria;p__Actinobacteria;c__Coriobacteriia;o__Coriobacteriales;f__Coriobacteriaceae;g__Parvibacter	0.00 ± 0.00	0.0	0.00 ± 0.00	0.0	0.0055	-	2.4199	0.0055
k__Bacteria;p__Actinobacteria;c__Coriobacteriia;o__Coriobacteriales;f__Coriobacteriaceae;g__Senegalimassilia	0.00 ± 0.00	0.0	0.01 ± 0.00	0.0	0.3669	-	-	-
k__Bacteria;p__Actinobacteria;c__Coriobacteriia;o__Eggerthellales;f__Eggerthellaceae;g__	0.01 ± 0.00	0.0	0.01 ± 0.01	0.0	0.4864	-	-	-
k__Bacteria;p__Actinobacteria;c__Coriobacteriia;o__Eggerthellales;f__Eggerthellaceae;g__Eggerthella	0.00 ± 0.00	0.0	0.00 ± 0.00	0.0	0.0259	-	2.3194	0.0270
k__Bacteria;p__Actinobacteria;c__Coriobacteriia;o__Eggerthellales;f__Eggerthellaceae;g__Enterorhabdus	0.00 ± 0.00	0.0	0.01 ± 0.01	0.0	0.3820	-	-	-
k__Bacteria;p__Actinobacteria;c__Coriobacteriia;o__Eggerthellales;f__Eggerthellaceae;g__Raoultibacter	0.00 ± 0.00	0.0	0.00 ± 0.00	0.0	0.6350	-	-	-
k__Bacteria;p__Actinobacteria;c__Coriobacteriia;o__Eggerthellales;f__Eggerthellaceae;g__Slackia	0.00 ± 0.00	0.0	0.01 ± 0.00	0.0	0.3669	-	-	-
k__Bacteria;p__Bacteroidetes;c__Bacteroidia;o__Bacteroidales;f__;g__	0.01 ± 0.01	0.0	0.00 ± 0.00	0.0	0.6216	-	-	-
k__Bacteria;p__Bacteroidetes;c__Bacteroidia;o__Bacteroidales;f__Bacteroidaceae;g__	0.45 ± 0.13	3.4	0.11 ± 0.04	0.0	0.0001	3.2578	-	0.0001
k__Bacteria;p__Bacteroidetes;c__Bacteroidia;o__Bacteroidales;f__Bacteroidaceae;g__Bacteroides	0.00 ± 0.00	0.0	0.04 ± 0.02	0.0	0.1290	-	-	-
k__Bacteria;p__Bacteroidetes;c__Bacteroidia;o__Bacteroidales;f__Porphyromonadaceae;g__	1.44 ± 0.35	43.1	0.88 ± 0.53	13.3	0.0517	3.5872	-	0.0369
k__Bacteria;p__Bacteroidetes;c__Bacteroidia;o__Bacteroidales;f__Porphyromonadaceae;g__Muribaculum	0.02 ± 0.02	0.0	0.01 ± 0.01	0.0	0.2871	-	-	-
k__Bacteria;p__Bacteroidetes;c__Bacteroidia;o__Bacteroidales;f__Porphyromonadaceae;g__Paludibacter	0.04 ± 0.02	0.0	0.10 ± 0.08	6.7	0.0761	-	-	-
k__Bacteria;p__Bacteroidetes;c__Bacteroidia;o__Bacteroidales;f__Porphyromonadaceae;g__Parabacteroides	0.00 ± 0.00	0.0	0.00 ± 0.00	0.0	0.5649	-	-	-
k__Bacteria;p__Bacteroidetes;c__Bacteroidia;o__Bacteroidales;f__Porphyromonadaceae;g__Petrimonas	0.00 ± 0.00	0.0	0.00 ± 0.00	0.0	0.0533	-	-	-
k__Bacteria;p__Bacteroidetes;c__Bacteroidia;o__Bacteroidales;f__Porphyromonadaceae;g__Porphyromonas	10.45 ± 1.18	96.6	9.26 ± 1.83	86.7	0.7691	-	-	-
k__Bacteria;p__Bacteroidetes;c__Bacteroidia;o__Bacteroidales;f__Porphyromonadaceae;g__Proteiniphilum	0.00 ± 0.00	0.0	0.00 ± 0.00	0.0	0.0533	-	-	-
**k__Bacteria;p__Bacteroidetes;c__Bacteroidia;o__Bacteroidales;f__Prevotellaceae;g__**	**3.82 ± 0.45**	**75.9**	**0.57 ± 0.28**	**13.3**	**0.0000**	**4.2259**	**-**	**0.0000**
k__Bacteria;p__Bacteroidetes;c__Bacteroidia;o__Bacteroidales;f__Prevotellaceae;g__Alloprevotella	0.01 ± 0.00	0.0	0.03 ± 0.02	0.0	0.4722	-	-	-

**k__Bacteria;p__Bacteroidetes;c__Bacteroidia;o__Bacteroidales;f__Prevotellaceae;g__Prevotella**	**6.51 ± 0.62**	**98.3**	**5.83 ± 1.99**	**80.0**	**0.0412**	**3.9487**	**-**	**0.0451**
k__Bacteria;p__Bacteroidetes;c__Bacteroidia;o__Bacteroidales;f__Prevotellaceae;g__Prevotellamassilia	0.19 ± 0.07	3.4	0.37 ± 0.25	13.3	0.2834	-	-	-
k__Bacteria;p__Bacteroidetes;c__Bacteroidia;o__Bacteroidales;f__Rikenellaceae;g__	0.01 ± 0.00	0.0	0.02 ± 0.02	0.0	0.8607	-	-	-
k__Bacteria;p__Bacteroidetes;c__Bacteroidia;o__Bacteroidales;f__Rikenellaceae;g__Rikenellaceae_RC9_gut_group	0.00 ± 0.00	0.0	0.02 ± 0.01	0.0	0.1340	-	-	-
k__Bacteria;p__Bacteroidetes;c__Bacteroidia;o__Marinilabiliales;f__Marinilabiliaceae;g__	0.01 ± 0.00	0.0	0.01 ± 0.01	0.0	0.3627	-	-	-
k__Bacteria;p__Bacteroidetes;c__Bacteroidia;o__Marinilabiliales;f__Prolixibacteraceae;g__Mariniphaga	0.00 ± 0.00	0.0	0.00 ± 0.00	0.0	0.0533	-	-	-
k__Bacteria;p__Bacteroidetes;c__Chitinophagia;o__Chitinophagales;f__Chitinophagaceae;g__Chitinophaga	0.00 ± 0.00	0.0	0.00 ± 0.00	0.0	0.0533	-	-	-
k__Bacteria;p__Bacteroidetes;c__Cytophagia;o__Cytophagales;f__Hymenobacteraceae;g__Siccationidurans	0.00 ± 0.00	0.0	0.00 ± 0.00	0.0	0.6350	-	-	-
k__Bacteria;p__Bacteroidetes;c__Flavobacteriia;o__Flavobacteriales;f__Crocinitomicaceae;g__Fluviicola	0.00 ± 0.00	0.0	0.01 ± 0.01	0.0	0.0533	-	-	-
k__Bacteria;p__Bacteroidetes;c__Flavobacteriia;o__Flavobacteriales;f__Flavobacteriaceae;g__	0.00 ± 0.00	0.0	0.00 ± 0.00	0.0	0.6964	-	-	-
k__Bacteria;p__Bacteroidetes;c__Flavobacteriia;o__Flavobacteriales;f__Flavobacteriaceae;g__Chryseobacterium	0.00 ± 0.00	0.0	0.05 ± 0.05	0.0	0.1413	-	2.7558	0.0474
k__Bacteria;p__Bacteroidetes;c__Flavobacteriia;o__Flavobacteriales;f__Flavobacteriaceae;g__Flavobacterium	0.00 ± 0.00	0.0	0.01 ± 0.01	0.0	0.0533	-	-	-
k__Bacteria;p__Bacteroidetes;c__Flavobacteriia;o__Flavobacteriales;f__Flavobacteriaceae;g__Riemerella	0.00 ± 0.00	0.0	0.01 ± 0.01	0.0	0.0533	-	-	-
k__Bacteria;p__Bacteroidetes;c__Flavobacteriia;o__Flavobacteriales;f__Flavobacteriaceae;g__Soonwooa	0.00 ± 0.00	0.0	0.05 ± 0.05	0.0	0.0533	-	-	-
k__Bacteria;p__Bacteroidetes;c__Sphingobacteriia;o__Sphingobacteriales;f__Chitinophagaceae;g__Taibaiella	0.00 ± 0.00	0.0	0.00 ± 0.00	0.0	0.0533	-	-	-
k__Bacteria;p__Bacteroidetes;c__Sphingobacteriia;o__Sphingobacteriales;f__Sphingobacteriaceae;g__	0.01 ± 0.00	0.0	0.01 ± 0.01	0.0	0.4736	-	-	-
k__Bacteria;p__Bacteroidetes;c__Sphingobacteriia;o__Sphingobacteriales;f__Sphingobacteriaceae;g__Sphingobacterium	0.00 ± 0.00	0.0	0.18 ± 0.18	6.7	0.0533	-	-	-
k__Bacteria;p__Chloroflexi;c__Thermomicrobia;o__Sphaerobacterales;f__Sphaerobacteraceae;g__	0.00 ± 0.00	0.0	0.00 ± 0.00	0.0	0.4839	-	-	-
k__Bacteria;p__Cyanobacteria;c__Cyanobacteria;o__SubsectionIII;f__FamilyI;g__	0.00 ± 0.00	0.0	0.00 ± 0.00	0.0	0.6350	-	-	-
k__Bacteria;p__Cyanobacteria;c__Melainabacteria;o__Gastranaerophilales;f__;g__	0.01 ± 0.00	0.0	0.00 ± 0.00	0.0	0.3505	-	-	-
k__Bacteria;p__Elusimicrobia;c__Elusimicrobia;o__Elusimicrobiales;f__Elusimicrobiaceae;g__Elusimicrobium	0.00 ± 0.00	0.0	0.00 ± 0.00	0.0	0.3072	-	-	-
k__Bacteria;p__Fibrobacteres;c__Fibrobacteria;o__Fibrobacterales;f__Fibrobacteraceae;g__Fibrobacter	0.00 ± 0.00	0.0	0.00 ± 0.00	0.0	0.3825	-	-	-
k__Bacteria;p__Firmicutes;c__Bacilli;o__Bacillales;f__Alicyclobacillaceae;g__Alicyclobacillus	0.01 ± 0.00	0.0	0.02 ± 0.01	0.0	0.0776	-	-	-
k__Bacteria;p__Firmicutes;c__Bacilli;o__Bacillales;f__Bacillaceae;g__	0.00 ± 0.00	0.0	0.00 ± 0.00	0.0	0.4839	-	-	-
k__Bacteria;p__Firmicutes;c__Bacilli;o__Bacillales;f__Bacillaceae;g__Bacillus	0.00 ± 0.00	0.0	0.00 ± 0.00	0.0	0.0533	-	-	-
k__Bacteria;p__Firmicutes;c__Bacilli;o__Bacillales;f__Family_XI;g__Gemella	0.00 ± 0.00	0.0	0.01 ± 0.01	0.0	0.0007	-	2.1445	0.0007
k__Bacteria;p__Firmicutes;c__Bacilli;o__Bacillales;f__Paenibacillaceae;g__	0.00 ± 0.00	0.0	0.01 ± 0.00	0.0	0.2779	-	-	-
k__Bacteria;p__Firmicutes;c__Bacilli;o__Bacillales;f__Paenibacillaceae;g__Paenibacillus	0.00 ± 0.00	0.0	0.01 ± 0.01	0.0	0.0533	-	-	-
k__Bacteria;p__Firmicutes;c__Bacilli;o__Bacillales;f__Staphylococcaceae;g__Jeotgalicoccus	0.00 ± 0.00	0.0	0.10 ± 0.10	6.7	0.0533	-	-	-
k__Bacteria;p__Firmicutes;c__Bacilli;o__Bacillales;f__Staphylococcaceae;g__Macrococcus	0.00 ± 0.00	0.0	0.01 ± 0.01	0.0	0.0533	-	-	-
k__Bacteria;p__Firmicutes;c__Bacilli;o__Bacillales;f__Staphylococcaceae;g__Staphylococcus	0.00 ± 0.00	0.0	0.02 ± 0.02	0.0	0.5649	-	-	-
k__Bacteria;p__Firmicutes;c__Bacilli;o__Bacillales;f__Thermoactinomycetaceae;g__	0.00 ± 0.00	0.0	0.00 ± 0.00	0.0	0.0518	-	-	-
k__Bacteria;p__Firmicutes;c__Bacilli;o__Lactobacillales;f__Aerococcaceae;g__	0.02 ± 0.01	0.0	0.15 ± 0.06	0.0	0.0215	-	2.8370	0.0235
k__Bacteria;p__Firmicutes;c__Bacilli;o__Lactobacillales;f__Aerococcaceae;g__Aerococcus	0.61 ± 0.22	10.3	1.30 ± 0.69	26.7	0.1500	-	-	-
k__Bacteria;p__Firmicutes;c__Bacilli;o__Lactobacillales;f__Aerococcaceae;g__Dolosicoccus	0.02 ± 0.01	0.0	0.09 ± 0.05	0.0	0.0236	-	2.5839	0.0257
k__Bacteria;p__Firmicutes;c__Bacilli;o__Lactobacillales;f__Aerococcaceae;g__Eremococcus	0.06 ± 0.03	3.4	0.29 ± 0.17	13.3	0.0030	-	3.0410	0.0034
k__Bacteria;p__Firmicutes;c__Bacilli;o__Lactobacillales;f__Aerococcaceae;g__Facklamia	0.68 ± 0.20	15.5	0.81 ± 0.27	26.7	0.2131	-	-	-
k__Bacteria;p__Firmicutes;c__Bacilli;o__Lactobacillales;f__Carnobacteriaceae;g__Alkalibacterium	0.00 ± 0.00	0.0	0.00 ± 0.00	0.0	0.0533	-	-	-
k__Bacteria;p__Firmicutes;c__Bacilli;o__Lactobacillales;f__Carnobacteriaceae;g__Atopostipes	0.09 ± 0.03	1.7	0.20 ± 0.09	6.7	0.7043	-	-	-
k__Bacteria;p__Firmicutes;c__Bacilli;o__Lactobacillales;f__Carnobacteriaceae;g__Granulicatella	0.00 ± 0.00	0.0	0.01 ± 0.01	0.0	0.0055	-	2.2000	0.0055
k__Bacteria;p__Firmicutes;c__Bacilli;o__Lactobacillales;f__Lactobacillaceae;g__Lactobacillus	0.17 ± 0.06	5.2	0.64 ± 0.25	26.7	0.0090	-	3.3067	0.0101
k__Bacteria;p__Firmicutes;c__Bacilli;o__Lactobacillales;f__Leuconostocaceae;g__Weissella	0.00 ± 0.00	0.0	0.01 ± 0.01	0.0	0.0932	-	-	-
**k__Bacteria;p__Firmicutes;c__Bacilli;o__Lactobacillales;f__Streptococcaceae;g__Streptococcus**	**1.38 ± 0.81**	**13.8**	**0.29 ± 0.11**	**13.3**	**0.0479**	**3.7296**	**-**	**0.0491**
k__Bacteria;p__Firmicutes;c__Clostridia;o__Clostridiales;f__;g__	0.00 ± 0.00	0.0	0.01 ± 0.00	0.0	0.3352	-	-	-
k__Bacteria;p__Firmicutes;c__Clostridia;o__Clostridiales;f__Bacteroides;g__pectinophilus	0.00 ± 0.00	0.0	0.00 ± 0.00	0.0	0.2487	-	-	-
k__Bacteria;p__Firmicutes;c__Clostridia;o__Clostridiales;f__Caldicoprobacteraceae;g__	0.00 ± 0.00	0.0	0.00 ± 0.00	0.0	0.0258	-	2.2256	0.0265
k__Bacteria;p__Firmicutes;c__Clostridia;o__Clostridiales;f__Christensenellaceae;g__	0.01 ± 0.00	0.0	0.02 ± 0.01	0.0	0.3170	-	-	-
k__Bacteria;p__Firmicutes;c__Clostridia;o__Clostridiales;f__Christensenellaceae;g__Christensenella	0.00 ± 0.00	0.0	0.00 ± 0.00	0.0	0.3223	-	-	-
k__Bacteria;p__Firmicutes;c__Clostridia;o__Clostridiales;f__Christensenellaceae;g__Christensenellaceae_R-7_group	0.03 ± 0.01	0.0	0.05 ± 0.02	0.0	0.1953	-	-	-
k__Bacteria;p__Firmicutes;c__Clostridia;o__Clostridiales;f__Clostridiaceae;g__	0.79 ± 0.22	17.2	1.23 ± 0.89	20.0	0.4007	-	-	-
k__Bacteria;p__Firmicutes;c__Clostridia;o__Clostridiales;f__Clostridiaceae;g__Beduini	0.01 ± 0.01	0.0	0.00 ± 0.00	0.0	0.0471	-	-	-
k__Bacteria;p__Firmicutes;c__Clostridia;o__Clostridiales;f__Clostridiaceae;g__Butyricicoccus	0.03 ± 0.01	0.0	0.08 ± 0.03	0.0	0.2956	-	-	-
k__Bacteria;p__Firmicutes;c__Clostridia;o__Clostridiales;f__Clostridiaceae;g__Clostridium	0.13 ± 0.05	1.7	0.23 ± 0.10	13.3	0.0147	-	2.7955	0.0130
k__Bacteria;p__Firmicutes;c__Clostridia;o__Clostridiales;f__Clostridiaceae;g__Hungatella	0.03 ± 0.01	0.0	0.02 ± 0.01	0.0	0.3730	-	-	-
k__Bacteria;p__Firmicutes;c__Clostridia;o__Clostridiales;f__Clostridiales;g__	0.00 ± 0.00	0.0	0.00 ± 0.00	0.0	0.3820	-	-	-
k__Bacteria;p__Firmicutes;c__Clostridia;o__Clostridiales;f__Clostridiales;g__Anaerovorax	0.01 ± 0.00	0.0	0.01 ± 0.01	0.0	0.7936	-	-	-

k__Bacteria;p__Firmicutes;c__Clostridia;o__Clostridiales;f__Clostridiales;g__Casaltella	0.00 ± 0.00	0.0	0.00 ± 0.00	0.0	0.6350	-	-	-
k__Bacteria;p__Firmicutes;c__Clostridia;o__Clostridiales;f__Clostridiales;g__Emergencia	0.00 ± 0.00	0.0	0.00 ± 0.00	0.0	0.8747	-	-	-
k__Bacteria;p__Firmicutes;c__Clostridia;o__Clostridiales;f__Clostridiales;g__Eubacterium	0.00 ± 0.00	0.0	0.01 ± 0.01	0.0	0.7151	-	-	-
k__Bacteria;p__Firmicutes;c__Clostridia;o__Clostridiales;f__Clostridiales;g__Ihubacter	0.00 ± 0.00	0.0	0.00 ± 0.00	0.0	0.0429	-	2.2911	0.0431
k__Bacteria;p__Firmicutes;c__Clostridia;o__Clostridiales;f__Clostridiales;g__Mogibacterium	0.00 ± 0.00	0.0	0.00 ± 0.00	0.0	0.0202	-	2.5899	0.0208
k__Bacteria;p__Firmicutes;c__Clostridia;o__Clostridiales;f__Defluviitaleaceae;g__	0.00 ± 0.00	0.0	0.00 ± 0.00	0.0	0.2993	-	-	-
k__Bacteria;p__Firmicutes;c__Clostridia;o__Clostridiales;f__Defluviitaleaceae;g__Vallitalea	0.00 ± 0.00	0.0	0.01 ± 0.01	0.0	0.0533	-	-	-
k__Bacteria;p__Firmicutes;c__Clostridia;o__Clostridiales;f__Eubacteriaceae;g__	0.08 ± 0.02	0.0	0.02 ± 0.01	0.0	0.0522	2.4812	-	0.0444
k__Bacteria;p__Firmicutes;c__Clostridia;o__Clostridiales;f__Eubacteriaceae;g__Eubacterium	0.24 ± 0.09	5.2	0.50 ± 0.23	13.3	0.0474	-	-	-
k__Bacteria;p__Firmicutes;c__Clostridia;o__Clostridiales;f__Family_XI;g__Murdochiella	0.01 ± 0.01	0.0	0.00 ± 0.00	0.0	0.7635	-	-	-
k__Bacteria;p__Firmicutes;c__Clostridia;o__Clostridiales;f__Family_XIII;g__Family_XIII_UCG-001	0.08 ± 0.01	0.0	0.03 ± 0.02	0.0	0.0056	2.4141	-	0.0058
k__Bacteria;p__Firmicutes;c__Clostridia;o__Clostridiales;f__Fenollaria;g__massiliensis	0.81 ± 0.35	17.2	0.09 ± 0.07	6.7	0.0324	3.5743	-	0.0340
k__Bacteria;p__Firmicutes;c__Clostridia;o__Clostridiales;f__Flavonifractor;g__	0.00 ± 0.00	0.0	0.00 ± 0.00	0.0	0.8220	-	-	-
k__Bacteria;p__Firmicutes;c__Clostridia;o__Clostridiales;f__Flintibacter;g__	0.02 ± 0.01	0.0	0.04 ± 0.03	0.0	0.0770	-	-	-
k__Bacteria;p__Firmicutes;c__Clostridia;o__Clostridiales;f__Gracilibacteraceae;g__	0.00 ± 0.00	0.0	0.00 ± 0.00	0.0	0.0036	-	2.4905	0.0038
k__Bacteria;p__Firmicutes;c__Clostridia;o__Clostridiales;f__Gracilibacteraceae;g__Gracilibacter	0.00 ± 0.00	0.0	0.00 ± 0.00	0.0	0.1413	-	-	-
k__Bacteria;p__Firmicutes;c__Clostridia;o__Clostridiales;f__Heliobacteriaceae;g__	0.00 ± 0.00	0.0	0.01 ± 0.01	0.0	0.1003	-	-	-
k__Bacteria;p__Firmicutes;c__Clostridia;o__Clostridiales;f__Howardella;g__	0.02 ± 0.01	0.0	0.02 ± 0.01	0.0	0.3823	-	-	-
k__Bacteria;p__Firmicutes;c__Clostridia;o__Clostridiales;f__Intestinimonas;g__	0.05 ± 0.02	0.0	0.13 ± 0.06	0.0	0.3368	-	-	-
**k__Bacteria;p__Firmicutes;c__Clostridia;o__Clostridiales;f__Lachnospiraceae;g__**	**3.34 ± 0.47**	**67.2**	**1.30 ± 0.55**	**26.7**	**0.0086**	**3.9724**	**-**	**0.0101**
k__Bacteria;p__Firmicutes;c__Clostridia;o__Clostridiales;f__Lachnospiraceae;g__Acetatifactor	0.00 ± 0.00	0.0	0.00 ± 0.00	0.0	0.8762	-	-	-
k__Bacteria;p__Firmicutes;c__Clostridia;o__Clostridiales;f__Lachnospiraceae;g__Anaerobium	0.00 ± 0.00	0.0	0.00 ± 0.00	0.0	0.0769	-	-	-
k__Bacteria;p__Firmicutes;c__Clostridia;o__Clostridiales;f__Lachnospiraceae;g__Anaerocolumna	0.00 ± 0.00	0.0	0.00 ± 0.00	0.0	0.4839	-	-	-
k__Bacteria;p__Firmicutes;c__Clostridia;o__Clostridiales;f__Lachnospiraceae;g__Anaerosporobacter	0.02 ± 0.01	0.0	0.01 ± 0.01	0.0	0.7944	-	-	-
k__Bacteria;p__Firmicutes;c__Clostridia;o__Clostridiales;f__Lachnospiraceae;g__Anaerostipes	0.07 ± 0.03	1.7	0.17 ± 0.08	6.7	0.0220	-	2.6753	0.0220
k__Bacteria;p__Firmicutes;c__Clostridia;o__Clostridiales;f__Lachnospiraceae;g__Blautia	0.24 ± 0.09	8.6	0.50 ± 0.19	26.7	0.0321	-	3.1102	0.0305
**k__Bacteria;p__Firmicutes;c__Clostridia;o__Clostridiales;f__Lachnospiraceae;g__Butyrivibrio**	**2.80 ± 0.58**	**56.9**	**0.67 ± 0.45**	**13.3**	**0.0013**	**4.0765**	**-**	**0.0009**
k__Bacteria;p__Firmicutes;c__Clostridia;o__Clostridiales;f__Lachnospiraceae;g__Catonella	0.61 ± 0.18	13.8	0.20 ± 0.11	13.3	0.0093	3.2735	-	0.0097
k__Bacteria;p__Firmicutes;c__Clostridia;o__Clostridiales;f__Lachnospiraceae;g__Clostridium	0.23 ± 0.09	5.2	0.27 ± 0.11	13.3	0.1099	-	-	-
k__Bacteria;p__Firmicutes;c__Clostridia;o__Clostridiales;f__Lachnospiraceae;g__Coprococcus	0.04 ± 0.02	0.0	0.08 ± 0.03	0.0	0.4472	-	-	-
k__Bacteria;p__Firmicutes;c__Clostridia;o__Clostridiales;f__Lachnospiraceae;g__Cuneatibacter	0.01 ± 0.01	0.0	0.00 ± 0.00	0.0	0.8264	-	-	-
k__Bacteria;p__Firmicutes;c__Clostridia;o__Clostridiales;f__Lachnospiraceae;g__Desulfotomaculum	0.07 ± 0.04	1.7	0.10 ± 0.06	0.0	0.5557	-	-	-
k__Bacteria;p__Firmicutes;c__Clostridia;o__Clostridiales;f__Lachnospiraceae;g__Dorea	0.11 ± 0.04	3.4	0.36 ± 0.17	13.3	0.0162	-	3.0433	0.0186
k__Bacteria;p__Firmicutes;c__Clostridia;o__Clostridiales;f__Lachnospiraceae;g__Eisenbergiella	0.00 ± 0.00	0.0	0.03 ± 0.02	0.0	0.1438	-	-	-
k__Bacteria;p__Firmicutes;c__Clostridia;o__Clostridiales;f__Lachnospiraceae;g__Eubacterium	0.15 ± 0.06	5.2	0.22 ± 0.11	6.7	0.1947	-	-	-
k__Bacteria;p__Firmicutes;c__Clostridia;o__Clostridiales;f__Lachnospiraceae;g__Fusicatenibacter	0.01 ± 0.00	0.0	0.06 ± 0.03	0.0	0.1934	-	-	-
k__Bacteria;p__Firmicutes;c__Clostridia;o__Clostridiales;f__Lachnospiraceae;g__Hespellia	0.00 ± 0.00	0.0	0.00 ± 0.00	0.0	0.5921	-	-	-
k__Bacteria;p__Firmicutes;c__Clostridia;o__Clostridiales;f__Lachnospiraceae;g__Lachnospira	0.01 ± 0.01	0.0	0.01 ± 0.01	0.0	0.0124	-	-	0.0132
k__Bacteria;p__Firmicutes;c__Clostridia;o__Clostridiales;f__Lachnospiraceae;g__Murimonas	0.01 ± 0.00	0.0	0.02 ± 0.02	0.0	1.0000	-	-	-
k__Bacteria;p__Firmicutes;c__Clostridia;o__Clostridiales;f__Lachnospiraceae;g__Pseudobutyrivibrio	0.00 ± 0.00	0.0	0.01 ± 0.01	0.0	0.9475	-	-	-
k__Bacteria;p__Firmicutes;c__Clostridia;o__Clostridiales;f__Lachnospiraceae;g__Roseburia	0.14 ± 0.07	5.2	0.19 ± 0.15	6.7	0.0835	-	-	-
k__Bacteria;p__Firmicutes;c__Clostridia;o__Clostridiales;f__Levyella;g__	0.00 ± 0.00	0.0	0.00 ± 0.00	0.0	0.6341	-	-	-
k__Bacteria;p__Firmicutes;c__Clostridia;o__Clostridiales;f__Oscillospiraceae;g__Oscillibacter	0.07 ± 0.03	1.7	0.33 ± 0.19	6.7	0.1417	-	-	-
k__Bacteria;p__Firmicutes;c__Clostridia;o__Clostridiales;f__Peptococcaceae;g__	0.02 ± 0.01	0.0	0.03 ± 0.01	0.0	0.1468	-	-	-
k__Bacteria;p__Firmicutes;c__Clostridia;o__Clostridiales;f__Peptococcaceae;g__Peptococcus	0.13 ± 0.03	0.0	0.12 ± 0.06	0.0	0.6570	-	-	-
**k__Bacteria;p__Firmicutes;c__Clostridia;o__Clostridiales;f__Peptostreptococcaceae;g__**	**2.03 ± 0.33**	**55.2**	**0.73 ± 0.26**	**20.0**	**0.0055**	**3.7792**	**-**	**0.0064**
k__Bacteria;p__Firmicutes;c__Clostridia;o__Clostridiales;f__Peptostreptococcaceae;g__Intestinibacter	0.01 ± 0.00	0.0	0.02 ± 0.02	0.0	0.0260	-	-	0.0275
k__Bacteria;p__Firmicutes;c__Clostridia;o__Clostridiales;f__Peptostreptococcaceae;g__Peptostreptococcus	0.20 ± 0.08	5.2	0.15 ± 0.12	6.7	0.4812	-	-	-
k__Bacteria;p__Firmicutes;c__Clostridia;o__Clostridiales;f__Peptostreptococcaceae;g__Romboutsia	0.01 ± 0.00	0.0	0.00 ± 0.00	0.0	0.4825	-	-	-
k__Bacteria;p__Firmicutes;c__Clostridia;o__Clostridiales;f__Peptostreptococcaceae;g__Terrisporobacter	0.00 ± 0.00	0.0	0.01 ± 0.01	0.0	0.2752	-	-	-
k__Bacteria;p__Firmicutes;c__Clostridia;o__Clostridiales;f__Pseudoflavonifractor;g__	0.00 ± 0.00	0.0	0.00 ± 0.00	0.0	0.6350	-	-	-
k__Bacteria;p__Firmicutes;c__Clostridia;o__Clostridiales;f__Pseudoflavonifractor;g__capillosus	0.00 ± 0.00	0.0	0.00 ± 0.00	0.0	0.2617	-	-	-
k__Bacteria;p__Firmicutes;c__Clostridia;o__Clostridiales;f__Ruminococcaceae;g__	1.65 ± 0.22	55.2	1.97 ± 0.68	53.3	0.6087	-	-	-
k__Bacteria;p__Firmicutes;c__Clostridia;o__Clostridiales;f__Ruminococcaceae;g__Acetanaerobacterium	0.00 ± 0.00	0.0	0.01 ± 0.00	0.0	0.3820	-	-	-
k__Bacteria;p__Firmicutes;c__Clostridia;o__Clostridiales;f__Ruminococcaceae;g__Acetivibrio	0.01 ± 0.01	0.0	0.00 ± 0.00	0.0	0.6984	-	-	-
k__Bacteria;p__Firmicutes;c__Clostridia;o__Clostridiales;f__Ruminococcaceae;g__Anaerobacterium	0.00 ± 0.00	0.0	0.00 ± 0.00	0.0	0.0006	-	2.3692	0.0006

k__Bacteria;p__Firmicutes;c__Clostridia;o__Clostridiales;f__Ruminococcaceae;g__Anaerofilum	0.01 ± 0.01	0.0	0.01 ± 0.01	0.0	0.6908	-	-	-
k__Bacteria;p__Firmicutes;c__Clostridia;o__Clostridiales;f__Ruminococcaceae;g__Anaerotruncus	0.01 ± 0.00	0.0	0.02 ± 0.01	0.0	0.1755	-	-	-
k__Bacteria;p__Firmicutes;c__Clostridia;o__Clostridiales;f__Ruminococcaceae;g__Caproiciproducens	0.00 ± 0.00	0.0	0.00 ± 0.00	0.0	0.4475	-	-	-
k__Bacteria;p__Firmicutes;c__Clostridia;o__Clostridiales;f__Ruminococcaceae;g__Clostridium	0.07 ± 0.03	1.7	0.05 ± 0.02	0.0	0.1671	-	-	-
k__Bacteria;p__Firmicutes;c__Clostridia;o__Clostridiales;f__Ruminococcaceae;g__Ethanoligenens	0.00 ± 0.00	0.0	0.02 ± 0.01	0.0	0.0844	-	-	-
k__Bacteria;p__Firmicutes;c__Clostridia;o__Clostridiales;f__Ruminococcaceae;g__Eubacterium	0.01 ± 0.00	0.0	0.00 ± 0.00	0.0	0.2854	-	-	-
**k__Bacteria;p__Firmicutes;c__Clostridia;o__Clostridiales;f__Ruminococcaceae;g__Faecalibacterium**	**0.56 ± 0.26**	**10.3**	**1.53 ± 0.84**	**26.7**	**0.0308**	**-**	**3.6680**	**0.0314**
k__Bacteria;p__Firmicutes;c__Clostridia;o__Clostridiales;f__Ruminococcaceae;g__Fastidiosipila	0.11 ± 0.02	0.0	0.28 ± 0.13	6.7	0.2818	-	-	-
k__Bacteria;p__Firmicutes;c__Clostridia;o__Clostridiales;f__Ruminococcaceae;g__Gemmiger	0.04 ± 0.02	1.7	0.06 ± 0.02	0.0	0.1187	-	-	-
k__Bacteria;p__Firmicutes;c__Clostridia;o__Clostridiales;f__Ruminococcaceae;g__Neglecta	0.00 ± 0.00	0.0	0.00 ± 0.00	0.0	0.5747	-	-	-
k__Bacteria;p__Firmicutes;c__Clostridia;o__Clostridiales;f__Ruminococcaceae;g__Papillibacter	0.00 ± 0.00	0.0	0.00 ± 0.00	0.0	0.9727	-	-	-
k__Bacteria;p__Firmicutes;c__Clostridia;o__Clostridiales;f__Ruminococcaceae;g__Ruminiclostridium	0.01 ± 0.00	0.0	0.02 ± 0.01	0.0	0.4135	-	-	-
k__Bacteria;p__Firmicutes;c__Clostridia;o__Clostridiales;f__Ruminococcaceae;g__Ruminococcus	0.12 ± 0.04	5.2	0.49 ± 0.20	20.0	0.0304	-	3.2414	0.0309
**k__Bacteria;p__Firmicutes;c__Clostridia;o__Clostridiales;f__Ruminococcaceae;g__Saccharofermentans**	**8.68 ± 0.76**	**82.8**	**4.62 ± 1.79**	**46.7**	**0.0167**	**4.2990**	**-**	**0.0169**
k__Bacteria;p__Firmicutes;c__Clostridia;o__Clostridiales;f__Ruminococcaceae;g__Sporobacter	0.10 ± 0.04	1.7	0.13 ± 0.04	0.0	0.1341	-	-	-
k__Bacteria;p__Firmicutes;c__Clostridia;o__Clostridiales;f__Ruminococcaceae;g__Subdoligranulum	0.00 ± 0.00	0.0	0.01 ± 0.00	0.0	0.2036	-	-	-
k__Bacteria;p__Firmicutes;c__Erysipelotrichia;o__Erysipelotrichales;f__Erysipelotrichaceae;g__	0.53 ± 0.09	17.2	0.16 ± 0.07	6.7	0.0022	3.2887	-	0.0024
k__Bacteria;p__Firmicutes;c__Erysipelotrichia;o__Erysipelotrichales;f__Erysipelotrichaceae;g__Bulleidia	0.01 ± 0.00	0.0	0.02 ± 0.01	0.0	0.5070	-	-	-
k__Bacteria;p__Firmicutes;c__Erysipelotrichia;o__Erysipelotrichales;f__Erysipelotrichaceae;g__Catenibacterium	0.05 ± 0.02	0.0	0.41 ± 0.18	20.0	0.0793	-	-	-
k__Bacteria;p__Firmicutes;c__Erysipelotrichia;o__Erysipelotrichales;f__Erysipelotrichaceae;g__Clostridium	0.04 ± 0.02	0.0	0.09 ± 0.04	0.0	0.0436	-	2.4357	0.0412
k__Bacteria;p__Firmicutes;c__Erysipelotrichia;o__Erysipelotrichales;f__Erysipelotrichaceae;g__Erysipelotrichaceae_UCG-004	0.00 ± 0.00	0.0	0.00 ± 0.00	0.0	0.6350	-	-	-
k__Bacteria;p__Firmicutes;c__Erysipelotrichia;o__Erysipelotrichales;f__Erysipelotrichaceae;g__Holdemanella	0.01 ± 0.00	0.0	0.02 ± 0.01	0.0	0.3737	-	-	-
k__Bacteria;p__Firmicutes;c__Erysipelotrichia;o__Erysipelotrichales;f__Erysipelotrichaceae;g__Holdemania	0.00 ± 0.00	0.0	0.00 ± 0.00	0.0	0.3072	-	-	-
k__Bacteria;p__Firmicutes;c__Erysipelotrichia;o__Erysipelotrichales;f__Erysipelotrichaceae;g__Longibaculum	0.02 ± 0.01	0.0	0.02 ± 0.01	0.0	0.7751	-	-	-
k__Bacteria;p__Firmicutes;c__Erysipelotrichia;o__Erysipelotrichales;f__Erysipelotrichaceae;g__Solobacterium	0.00 ± 0.00	0.0	0.00 ± 0.00	0.0	0.6350	-	-	-
k__Bacteria;p__Firmicutes;c__Negativicutes;o__Acidaminococcales;f__Acidaminococcaceae;g__Phascolarctobacterium	0.22 ± 0.07	6.9	1.94 ± 1.73	6.7	0.1916	-	-	-
k__Bacteria;p__Firmicutes;c__Negativicutes;o__Selenomonadales;f__Selenomonadaceae;g__	0.48 ± 0.11	19.0	0.10 ± 0.08	6.7	0.0105	3.2876	-	0.0080
k__Bacteria;p__Firmicutes;c__Negativicutes;o__Selenomonadales;f__Selenomonadaceae;g__Mitsuokella	0.00 ± 0.00	0.0	0.00 ± 0.00	0.0	0.4839	-	-	-
k__Bacteria;p__Firmicutes;c__Negativicutes;o__Selenomonadales;f__Selenomonadaceae;g__Propionispira	0.43 ± 0.21	8.6	0.33 ± 0.15	13.3	0.0926	-	-	-
**k__Bacteria;p__Firmicutes;c__Negativicutes;o__Selenomonadales;f__Selenomonadaceae;g__Selenomonas**	**1.24 ± 0.20**	**39.7**	**0.59 ± 0.21**	**26.7**	**0.0485**	**3.5017**	**-**	**0.0408**
k__Bacteria;p__Firmicutes;c__Negativicutes;o__Veillonellales;f__Veillonellaceae;g__Allisonella	0.00 ± 0.00	0.0	0.00 ± 0.00	0.0	0.0055	-	2.5657	0.0055
k__Bacteria;p__Firmicutes;c__Negativicutes;o__Veillonellales;f__Veillonellaceae;g__Dialister	6.11 ± 0.60	94.8	6.28 ± 2.30	80.0	0.1923	-	-	-
k__Bacteria;p__Firmicutes;c__Negativicutes;o__Veillonellales;f__Veillonellaceae;g__Megasphaera	0.01 ± 0.01	0.0	0.07 ± 0.05	0.0	0.2493	-	-	-
k__Bacteria;p__Firmicutes;c__Negativicutes;o__Veillonellales;f__Veillonellaceae;g__Veillonella	0.00 ± 0.00	0.0	0.02 ± 0.01	0.0	0.2433	-	-	-
k__Bacteria;p__Firmicutes;c__Tissierellia;o__Tissierellales;f__Peptoniphilaceae;g__	2.19 ± 0.32	63.8	2.34 ± 0.75	46.7	0.4324	-	-	-
k__Bacteria;p__Firmicutes;c__Tissierellia;o__Tissierellales;f__Peptoniphilaceae;g__Anaerococcus	1.35 ± 0.45	20.7	1.90 ± 0.72	40.0	0.1787	-	-	-
k__Bacteria;p__Firmicutes;c__Tissierellia;o__Tissierellales;f__Peptoniphilaceae;g__Anaerosphaera	0.08 ± 0.07	1.7	0.23 ± 0.17	6.7	0.0166	-	2.9836	0.0181
k__Bacteria;p__Firmicutes;c__Tissierellia;o__Tissierellales;f__Peptoniphilaceae;g__Finegoldia	0.28 ± 0.17	3.4	0.36 ± 0.15	13.3	0.1839	-	-	-
k__Bacteria;p__Firmicutes;c__Tissierellia;o__Tissierellales;f__Peptoniphilaceae;g__Gallicola	0.00 ± 0.00	0.0	0.00 ± 0.00	0.0	0.0533	-	-	-
k__Bacteria;p__Firmicutes;c__Tissierellia;o__Tissierellales;f__Peptoniphilaceae;g__Helcococcus	0.17 ± 0.07	3.4	0.42 ± 0.21	20.0	0.9395	-	-	-
**k__Bacteria;p__Firmicutes;c__Tissierellia;o__Tissierellales;f__Peptoniphilaceae;g__Parvimonas**	**1.21 ± 0.18**	**39.7**	**0.85 ± 0.39**	**26.7**	**0.0420**	**3.3452**	**-**	**0.0491**
k__Bacteria;p__Firmicutes;c__Tissierellia;o__Tissierellales;f__Peptoniphilaceae;g__Peptoniphilus	2.10 ± 0.33	58.6	1.90 ± 0.44	46.7	0.8324	-	-	-
k__Bacteria;p__Firmicutes;c__Tissierellia;o__Tissierellales;f__Tissierellaceae;g__	0.04 ± 0.02	0.0	0.60 ± 0.59	6.7	0.6056	-	-	-
k__Bacteria;p__Firmicutes;c__Tissierellia;o__Tissierellales;f__Tissierellaceae;g__Tissierella	0.02 ± 0.01	0.0	0.11 ± 0.06	0.0	0.3742	-	-	-
k__Bacteria;p__Fusobacteria;c__Fusobacteriia;o__Fusobacteriales;f__Fusobacteriaceae;g__Fusobacterium	4.40 ± 0.82	60.3	8.00 ± 2.96	46.7	1.0000	-	-	-
k__Bacteria;p__Fusobacteria;c__Fusobacteriia;o__Fusobacteriales;f__Leptotrichiaceae;g__Leptotrichia	0.00 ± 0.00	0.0	0.00 ± 0.00	0.0	0.3223	-	-	-
k__Bacteria;p__Fusobacteria;c__Fusobacteriia;o__Fusobacteriales;f__Leptotrichiaceae;g__Sneathia	6.02 ± 0.82	82.8	4.21 ± 1.37	53.3	0.1210	-	-	-
k__Bacteria;p__Fusobacteria;c__Fusobacteriia;o__Fusobacteriales;f__Leptotrichiaceae;g__Streptobacillus	0.00 ± 0.00	0.0	0.00 ± 0.00	0.0	0.5649	-	-	-
k__Bacteria;p__Gemmatimonadetes;c__;o__;f__;g__	0.00 ± 0.00	0.0	0.00 ± 0.00	0.0	0.0533	-	-	-
k__Bacteria;p__Gemmatimonadetes;c__Gemmatimonadetes;o__Gemmatimonadales;f__Gemmatimonadaceae;g__Gemmatirosa	0.00 ± 0.00	0.0	0.00 ± 0.00	0.0	0.6350	-	-	-
k__Bacteria;p__Lentisphaerae;c__Lentisphaeria;o__Victivallales;f__;g__	0.00 ± 0.00	0.0	0.01 ± 0.01	0.0	0.7952	-	-	-
k__Bacteria;p__Lentisphaerae;c__Lentisphaeria;o__Victivallales;f__Victivallaceae;g__	0.00 ± 0.00	0.0	0.01 ± 0.00	0.0	0.2361	-	-	-
k__Bacteria;p__Lentisphaerae;c__Oligosphaeria;o__Oligosphaerales;f__Oligosphaeraceae;g__	0.00 ± 0.00	0.0	0.00 ± 0.00	0.0	0.0533	-	-	-
k__Bacteria;p__Proteobacteria;c__Alphaproteobacteria;o__Caulobacterales;f__Caulobacteraceae;g__Brevundimonas	0.00 ± 0.00	0.0	0.07 ± 0.06	0.0	0.0055	-	2.6661	0.0055
k__Bacteria;p__Proteobacteria;c__Alphaproteobacteria;o__Rhizobiales;f__Bradyrhizobiaceae;g__Bosea	0.00 ± 0.00	0.0	0.01 ± 0.01	0.0	0.0055	-	2.0731	0.0055
k__Bacteria;p__Proteobacteria;c__Alphaproteobacteria;o__Rhizobiales;f__Brucellaceae;g__Pseudochrobactrum	0.00 ± 0.00	0.0	0.01 ± 0.01	0.0	0.0533	-	-	-

k__Bacteria;p__Proteobacteria;c__Alphaproteobacteria;o__Rhizobiales;f__Hyphomicrobiaceae;g__Devosia	0.00 ± 0.00	0.0	0.00 ± 0.00	0.0	0.0533	-	-	-
k__Bacteria;p__Proteobacteria;c__Alphaproteobacteria;o__Rhizobiales;f__Methylobacteriaceae;g__Methylobacterium	0.00 ± 0.00	0.0	0.01 ± 0.01	0.0	0.0227	-	2.3471	0.0237
k__Bacteria;p__Proteobacteria;c__Alphaproteobacteria;o__Rhizobiales;f__Phyllobacteriaceae;g__Mesorhizobium	0.00 ± 0.00	0.0	0.00 ± 0.00	0.0	0.0533	-	-	-
k__Bacteria;p__Proteobacteria;c__Alphaproteobacteria;o__Rhodobacterales;f__Rhodobacteraceae;g__	0.00 ± 0.00	0.0	0.01 ± 0.01	0.0	0.0533	-	-	-
k__Bacteria;p__Proteobacteria;c__Alphaproteobacteria;o__Rhodobacterales;f__Rhodobacteraceae;g__Aquimixticola	0.00 ± 0.00	0.0	0.04 ± 0.04	0.0	0.0533	-	-	-
k__Bacteria;p__Proteobacteria;c__Alphaproteobacteria;o__Rhodobacterales;f__Rhodobacteraceae;g__Gemmobacter	0.00 ± 0.00	0.0	0.00 ± 0.00	0.0	0.0533	-	-	-
k__Bacteria;p__Proteobacteria;c__Alphaproteobacteria;o__Rhodobacterales;f__Rhodobacteraceae;g__Paracoccus	0.00 ± 0.00	0.0	0.04 ± 0.04	0.0	0.0533	-	-	-
k__Bacteria;p__Proteobacteria;c__Alphaproteobacteria;o__Rhodospirillales;f__Acetobacteraceae;g__	0.00 ± 0.00	0.0	0.00 ± 0.00	0.0	0.0059	-	2.2870	0.0061
k__Bacteria;p__Proteobacteria;c__Alphaproteobacteria;o__Rhodospirillales;f__Rhodospirillaceae;g__	0.01 ± 0.00	0.0	0.04 ± 0.02	0.0	0.1562	-	-	-
k__Bacteria;p__Proteobacteria;c__Alphaproteobacteria;o__Rhodospirillales;f__Rhodospirillaceae;g__Ferrovibrio	0.00 ± 0.00	0.0	0.00 ± 0.00	0.0	0.0533	-	-	-
k__Bacteria;p__Proteobacteria;c__Alphaproteobacteria;o__Rhodospirillales;f__Rhodospirillaceae;g__Niveispirillum	0.00 ± 0.00	0.0	0.00 ± 0.00	0.0	0.0533	-	-	-
k__Bacteria;p__Proteobacteria;c__Alphaproteobacteria;o__Rhodospirillales;f__Rhodospirillaceae;g__Tistrella	0.00 ± 0.00	0.0	0.00 ± 0.00	0.0	0.5921	-	-	-
k__Bacteria;p__Proteobacteria;c__Alphaproteobacteria;o__Rickettsiales;f__;g__	0.00 ± 0.00	0.0	0.00 ± 0.00	0.0	0.0533	-	-	-
k__Bacteria;p__Proteobacteria;c__Alphaproteobacteria;o__Sphingomonadales;f__Sphingomonadaceae;g__	0.00 ± 0.00	0.0	0.00 ± 0.00	0.0	0.0533	-	-	-
k__Bacteria;p__Proteobacteria;c__Alphaproteobacteria;o__Sphingomonadales;f__Sphingomonadaceae;g__Novosphingobium	0.00 ± 0.00	0.0	0.00 ± 0.00	0.0	0.2487	-	-	-
k__Bacteria;p__Proteobacteria;c__Alphaproteobacteria;o__Sphingomonadales;f__Sphingomonadaceae;g__Sphingobium	0.00 ± 0.00	0.0	0.01 ± 0.01	0.0	0.0533	-	-	-
k__Bacteria;p__Proteobacteria;c__Alphaproteobacteria;o__Sphingomonadales;f__Sphingomonadaceae;g__Sphingomonas	0.00 ± 0.00	0.0	0.00 ± 0.00	0.0	0.0276	-	2.7108	0.0289
k__Bacteria;p__Proteobacteria;c__Alphaproteobacteria;o__Sphingomonadales;f__Sphingomonadaceae;g__Sphingopyxis	0.00 ± 0.00	0.0	0.00 ± 0.00	0.0	0.0533	-	-	-
k__Bacteria;p__Proteobacteria;c__Betaproteobacteria;o__Burkholderiales;f__Alcaligenaceae;g__Achromobacter	0.00 ± 0.00	0.0	0.01 ± 0.00	0.0	0.1321	-	-	-
k__Bacteria;p__Proteobacteria;c__Betaproteobacteria;o__Burkholderiales;f__Alcaligenaceae;g__Alcaligenes	0.00 ± 0.00	0.0	0.00 ± 0.00	0.0	0.0472	-	2.1338	0.0474
k__Bacteria;p__Proteobacteria;c__Betaproteobacteria;o__Burkholderiales;f__Alcaligenaceae;g__Pelistega	0.00 ± 0.00	0.0	0.09 ± 0.09	6.7	0.0533	-	-	-
k__Bacteria;p__Proteobacteria;c__Betaproteobacteria;o__Burkholderiales;f__Burkholderiaceae;g__Burkholderia	0.00 ± 0.00	0.0	0.00 ± 0.00	0.0	0.3825	-	-	-
k__Bacteria;p__Proteobacteria;c__Betaproteobacteria;o__Burkholderiales;f__Burkholderiaceae;g__Cupriavidus	0.00 ± 0.00	0.0	0.00 ± 0.00	0.0	0.0533	-	-	-
k__Bacteria;p__Proteobacteria;c__Betaproteobacteria;o__Burkholderiales;f__Burkholderiaceae;g__Limnobacter	0.00 ± 0.00	0.0	0.00 ± 0.00	0.0	0.6350	-	-	-
k__Bacteria;p__Proteobacteria;c__Betaproteobacteria;o__Burkholderiales;f__Burkholderiaceae;g__Paraburkholderia	0.00 ± 0.00	0.0	0.00 ± 0.00	0.0	0.2993	-	-	-
k__Bacteria;p__Proteobacteria;c__Betaproteobacteria;o__Burkholderiales;f__Burkholderiaceae;g__Ralstonia	0.00 ± 0.00	0.0	0.00 ± 0.00	0.0	0.2487	-	-	-
k__Bacteria;p__Proteobacteria;c__Betaproteobacteria;o__Burkholderiales;f__Comamonadaceae;g__	0.00 ± 0.00	0.0	0.02 ± 0.02	0.0	0.5896	-	-	-
k__Bacteria;p__Proteobacteria;c__Betaproteobacteria;o__Burkholderiales;f__Comamonadaceae;g__Comamonas	0.00 ± 0.00	0.0	0.02 ± 0.02	0.0	0.0055	-	2.5426	0.0055
k__Bacteria;p__Proteobacteria;c__Betaproteobacteria;o__Burkholderiales;f__Comamonadaceae;g__Pelomonas	0.00 ± 0.00	0.0	0.01 ± 0.01	0.0	0.9876	-	-	-
k__Bacteria;p__Proteobacteria;c__Betaproteobacteria;o__Burkholderiales;f__Oxalobacteraceae;g__Herbaspirillum	0.01 ± 0.01	0.0	0.01 ± 0.01	0.0	0.1194	-	-	-
k__Bacteria;p__Proteobacteria;c__Betaproteobacteria;o__Burkholderiales;f__Oxalobacteraceae;g__Janthinobacterium	0.00 ± 0.00	0.0	0.00 ± 0.00	0.0	0.0533	-	-	-
k__Bacteria;p__Proteobacteria;c__Betaproteobacteria;o__Burkholderiales;f__Oxalobacteraceae;g__Massilia	0.00 ± 0.00	0.0	0.00 ± 0.00	0.0	0.0533	-	-	-
k__Bacteria;p__Proteobacteria;c__Betaproteobacteria;o__Burkholderiales;f__Roseateles;g__	0.00 ± 0.00	0.0	0.02 ± 0.02	0.0	0.8739	-	-	-
k__Bacteria;p__Proteobacteria;c__Betaproteobacteria;o__Burkholderiales;f__Rubrivivax;g__gelatinosus	0.00 ± 0.00	0.0	0.11 ± 0.11	6.7	0.0055	-	2.9539	0.0055
k__Bacteria;p__Proteobacteria;c__Betaproteobacteria;o__Burkholderiales;f__Sutterellaceae;g__Sutterella	0.01 ± 0.01	0.0	0.02 ± 0.01	0.0	0.4743	-	-	-
k__Bacteria;p__Proteobacteria;c__Betaproteobacteria;o__Methylophilales;f__Methylophilaceae;g__	0.00 ± 0.00	0.0	0.00 ± 0.00	0.0	0.0533	-	-	-
k__Bacteria;p__Proteobacteria;c__Betaproteobacteria;o__Neisseriales;f__Neisseriaceae;g__	0.00 ± 0.00	0.0	0.00 ± 0.00	0.0	0.4839	-	-	-
k__Bacteria;p__Proteobacteria;c__Betaproteobacteria;o__Rhodocyclales;f__Rhodocyclaceae;g__Azoarcus	0.00 ± 0.00	0.0	0.00 ± 0.00	0.0	0.6350	-	-	-
k__Bacteria;p__Proteobacteria;c__Deltaproteobacteria;o__Bradymonadales;f__;g__	0.02 ± 0.02	1.7	0.01 ± 0.01	0.0	0.5445	-	-	-
k__Bacteria;p__Proteobacteria;c__Deltaproteobacteria;o__Desulfovibrionales;f__Desulfovibrionaceae;g__	0.00 ± 0.00	0.0	0.00 ± 0.00	0.0	0.9627	-	-	-
k__Bacteria;p__Proteobacteria;c__Deltaproteobacteria;o__Desulfovibrionales;f__Desulfovibrionaceae;g__Desulfovibrio	0.01 ± 0.00	0.0	0.02 ± 0.01	0.0	0.2292	-	-	-
k__Bacteria;p__Proteobacteria;c__Deltaproteobacteria;o__Desulfuromonadales;f__Geobacteraceae;g__Geobacter	0.00 ± 0.00	0.0	0.00 ± 0.00	0.0	0.1413	-	-	-
k__Bacteria;p__Proteobacteria;c__Deltaproteobacteria;o__Myxococcales;f__Phaselicystidaceae;g__Phaselicystis	0.00 ± 0.00	0.0	0.00 ± 0.00	0.0	0.6350	-	-	-
k__Bacteria;p__Proteobacteria;c__Epsilonproteobacteria;o__Campylobacterales;f__Campylobacteraceae;g__Arcobacter	0.00 ± 0.00	0.0	0.01 ± 0.01	0.0	0.0533	-	-	-
k__Bacteria;p__Proteobacteria;c__Epsilonproteobacteria;o__Campylobacterales;f__Campylobacteraceae;g__Campylobacter	9.30 ± 0.95	87.9	8.24 ± 1.63	80.0	0.7380	-	-	-
k__Bacteria;p__Proteobacteria;c__Epsilonproteobacteria;o__Campylobacterales;f__Helicobacteraceae;g__Helicobacter	0.26 ± 0.10	6.9	0.60 ± 0.33	13.3	0.1251	-	-	-
k__Bacteria;p__Proteobacteria;c__Gammaproteobacteria;o__;f__;g__	0.01 ± 0.00	0.0	0.02 ± 0.01	0.0	0.2732	-	-	-
k__Bacteria;p__Proteobacteria;c__Gammaproteobacteria;o__Aeromonadales;f__Aeromonadaceae;g__Aeromonas	0.00 ± 0.00	0.0	0.00 ± 0.00	0.0	0.6350	-	-	-
k__Bacteria;p__Proteobacteria;c__Gammaproteobacteria;o__Aeromonadales;f__Succinivibrionaceae;g__Succinivibrio	0.10 ± 0.03	1.7	0.32 ± 0.19	6.7	0.1806	-	-	-
k__Bacteria;p__Proteobacteria;c__Gammaproteobacteria;o__Alteromonadales;f__Alteromonadaceae;g__Marinobacter	0.00 ± 0.00	0.0	0.00 ± 0.00	0.0	0.0095	-	2.7401	0.0100
k__Bacteria;p__Proteobacteria;c__Gammaproteobacteria;o__Alteromonadales;f__Idiomarinaceae;g__Aliidiomarina	0.01 ± 0.01	0.0	0.01 ± 0.01	0.0	0.5566	-	-	-
k__Bacteria;p__Proteobacteria;c__Gammaproteobacteria;o__Cardiobacteriales;f__Cardiobacteriaceae;g__Suttonella	0.03 ± 0.03	1.7	0.00 ± 0.00	0.0	0.4839	-	-	-
k__Bacteria;p__Proteobacteria;c__Gammaproteobacteria;o__Enterobacterales;f__Enterobacteriaceae;g__	0.00 ± 0.00	0.0	0.01 ± 0.01	0.0	0.2663	-	-	-
k__Bacteria;p__Proteobacteria;c__Gammaproteobacteria;o__Enterobacterales;f__Enterobacteriaceae;g__Enterobacter	0.05 ± 0.05	1.7	0.00 ± 0.00	0.0	0.5921	-	-	-
k__Bacteria;p__Proteobacteria;c__Gammaproteobacteria;o__Enterobacterales;f__Morganellaceae;g__	0.00 ± 0.00	0.0	0.02 ± 0.02	0.0	0.0533	-	-	-
k__Bacteria;p__Proteobacteria;c__Gammaproteobacteria;o__Enterobacterales;f__Morganellaceae;g__Providencia	0.00 ± 0.00	0.0	0.01 ± 0.01	0.0	0.0533	-	-	-

k__Bacteria;p__Proteobacteria;c__Gammaproteobacteria;o__Oceanospirillales;f__Halomonadaceae;g__Chromohalobacter	0.00 ± 0.00	0.0	0.00 ± 0.00	0.0	0.3223	-	-	-
**k__Bacteria;p__Proteobacteria;c__Gammaproteobacteria;o__Oceanospirillales;f__Halomonadaceae;g__Halomonas**	**1.43 ± 0.64**	**13.8**	**3.05 ± 1.21**	**46.7**	**0.0127**	**-**	**3.9054**	**0.0138**
k__Bacteria;p__Proteobacteria;c__Gammaproteobacteria;o__Oceanospirillales;f__Oceanospirillaceae;g__Marinobacterium	0.00 ± 0.00	0.0	0.00 ± 0.00	0.0	0.0533	-	-	-
k__Bacteria;p__Proteobacteria;c__Gammaproteobacteria;o__Orbales;f__Orbaceae;g__	0.00 ± 0.00	0.0	0.00 ± 0.00	0.0	0.6350	-	-	-
k__Bacteria;p__Proteobacteria;c__Gammaproteobacteria;o__Pasteurellales;f__Pasteurellaceae;g__Aggregatibacter	0.00 ± 0.00	0.0	0.01 ± 0.00	0.0	0.0422	-	2.2246	0.0439
k__Bacteria;p__Proteobacteria;c__Gammaproteobacteria;o__Pasteurellales;f__Pasteurellaceae;g__Haemophilus	0.63 ± 0.53	3.4	2.50 ± 2.22	20.0	0.1114	-	-	-
k__Bacteria;p__Proteobacteria;c__Gammaproteobacteria;o__Pseudomonadales;f__Moraxellaceae;g__Acinetobacter	0.00 ± 0.00	0.0	0.17 ± 0.17	6.7	0.5751	-	-	-
k__Bacteria;p__Proteobacteria;c__Gammaproteobacteria;o__Pseudomonadales;f__Moraxellaceae;g__Moraxella	0.00 ± 0.00	0.0	0.00 ± 0.00	0.0	0.0533	-	-	-
k__Bacteria;p__Proteobacteria;c__Gammaproteobacteria;o__Pseudomonadales;f__Moraxellaceae;g__Psychrobacter	0.00 ± 0.00	0.0	0.00 ± 0.00	0.0	0.0533	-	-	-
k__Bacteria;p__Proteobacteria;c__Gammaproteobacteria;o__Pseudomonadales;f__Pseudomonadaceae;g__Pseudomonas	0.01 ± 0.01	0.0	0.01 ± 0.01	0.0	0.2001	-	-	-
k__Bacteria;p__Proteobacteria;c__Gammaproteobacteria;o__Xanthomonadales;f__Rhodanobacteraceae;g__Dokdonella	0.00 ± 0.00	0.0	0.00 ± 0.00	0.0	0.4839	-	-	-
k__Bacteria;p__Proteobacteria;c__Gammaproteobacteria;o__Xanthomonadales;f__Xanthomonadaceae;g__	0.00 ± 0.00	0.0	0.01 ± 0.01	0.0	0.0533	-	-	-
k__Bacteria;p__Proteobacteria;c__Gammaproteobacteria;o__Xanthomonadales;f__Xanthomonadaceae;g__Luteimonas	0.00 ± 0.00	0.0	0.03 ± 0.03	0.0	0.0533	-	-	-
k__Bacteria;p__Proteobacteria;c__Gammaproteobacteria;o__Xanthomonadales;f__Xanthomonadaceae;g__Lysobacter	0.00 ± 0.00	0.0	0.03 ± 0.03	0.0	0.0533	-	-	-
k__Bacteria;p__Proteobacteria;c__Gammaproteobacteria;o__Xanthomonadales;f__Xanthomonadaceae;g__Stenotrophomonas	0.00 ± 0.00	0.0	0.00 ± 0.00	0.0	0.0055	-	2.8871	0.0055
k__Bacteria;p__Proteobacteria;c__Gammaproteobacteria;o__Xanthomonadales;f__Xanthomonadaceae;g__Xanthomonas	0.00 ± 0.00	0.0	0.00 ± 0.00	0.0	0.0533	-	-	-
k__Bacteria;p__Spirochaetes;c__Spirochaetia;o__Brachyspirales;f__Brachyspiraceae;g__Brachyspira	0.00 ± 0.00	0.0	0.00 ± 0.00	0.0	0.0611	-	-	-
k__Bacteria;p__Spirochaetes;c__Spirochaetia;o__Spirochaetales;f__Spirochaetaceae;g__	4.31 ± 0.61	67.2	2.79 ± 1.02	46.7	0.1174	-	-	-
**k__Bacteria;p__Spirochaetes;c__Spirochaetia;o__Spirochaetales;f__Spirochaetaceae;g__Treponema**	**1.19 ± 0.24**	**31.0**	**0.69 ± 0.43**	**20.0**	**0.0203**	**3.5369**	**-**	**0.0191**
k__Bacteria;p__Tenericutes;c__Mollicutes;o__Acholeplasmatales;f__Acholeplasmataceae;g__	0.01 ± 0.00	0.0	0.00 ± 0.00	0.0	0.6088	-	-	-
k__Bacteria;p__Tenericutes;c__Mollicutes;o__Anaeroplasmatales;f__Anaeroplasmataceae;g__	0.02 ± 0.01	0.0	0.00 ± 0.00	0.0	0.5946	-	-	-
k__Bacteria;p__Tenericutes;c__Mollicutes;o__Anaeroplasmatales;f__Anaeroplasmataceae;g__Anaeroplasma	0.00 ± 0.00	0.0	0.00 ± 0.00	0.0	0.7635	-	-	-
k__Bacteria;p__Tenericutes;c__Mollicutes;o__Entomoplasmatales;f__Spiroplasmataceae;g__	0.00 ± 0.00	0.0	0.02 ± 0.01	0.0	0.0438	-	2.3082	0.0460
k__Bacteria;p__Tenericutes;c__Mollicutes;o__Mollicutes_RF9;f__;g__	0.01 ± 0.01	0.0	0.02 ± 0.02	0.0	0.4709	-	-	-
k__Bacteria;p__Tenericutes;c__Mollicutes;o__Mycoplasmatales;f__Mycoplasmataceae;g__	0.00 ± 0.00	0.0	0.00 ± 0.00	0.0	0.6198	-	-	-
k__Bacteria;p__Tenericutes;c__Mollicutes;o__Mycoplasmatales;f__Mycoplasmataceae;g__Mycoplasma	0.10 ± 0.04	3.4	0.01 ± 0.01	0.0	0.1061	-	-	-
k__Bacteria;p__Verrucomicrobia;c__;o__;f__;g__	0.01 ± 0.00	0.0	0.02 ± 0.01	0.0	0.4279	-	-	-
k__Bacteria;p__Verrucomicrobia;c__Opitutae;o__Opitutae_vadinHA64;f__;g__	0.00 ± 0.00	0.0	0.00 ± 0.00	0.0	0.6350	-	-	-
k__Bacteria;p__Verrucomicrobia;c__Opitutae;o__Puniceicoccales;f__Puniceicoccaceae;g__	0.00 ± 0.00	0.0	0.01 ± 0.01	0.0	0.5649	-	-	-

$ mean ± s.e.m

* Individual samples with >1% abundance were counted.
